# Geometagenomics illuminates the impact of agriculture on the distribution and prevalence of plant viruses at the ecosystem scale

**DOI:** 10.1038/ismej.2017.155

**Published:** 2017-10-20

**Authors:** Pauline Bernardo, Tristan Charles-Dominique, Mohamed Barakat, Philippe Ortet, Emmanuel Fernandez, Denis Filloux, Penelope Hartnady, Tony A Rebelo, Stephen R Cousins, François Mesleard, Damien Cohez, Nicole Yavercovski, Arvind Varsani, Gordon W Harkins, Michel Peterschmitt, Carolyn M Malmstrom, Darren P Martin, Philippe Roumagnac

**Affiliations:** 1 CIRAD, UMR BGPI, Montpellier, France; 2 BGPI, CIRAD, INRA, Montpellier SupAgro, Univ Montpellier, Montpellier, France; 3 Department of Plant Biology, Michigan State University, East Lansing, Michigan, USA; 4 Department of Plant Pathology, Ohio State University, OARDC, Wooster, OH, USA; 5 Xishuangbanna Tropical Botanical Garden, Chinese Academy of Sciences, Center for Integrative Conservation, Community Ecology and Conservation, Menglun, Yunnan, China; 6 South African National Biodiversity Institute, Kirstenbosch Research Centre, Cape Town, South Africa; 7 CEA, CNRS, Aix-Marseille Université, UMR 7265, LEMIRE, Saint-Paul-lez-Durance, France; 8 Computational Biology Division, Department of Integrative Biomedical Sciences, Institute of Infectious Diseases and Molecular Medicine. University of Cape Town, Observatory, South Africa; 9 Tour du Valat, Institut de recherche pour la conservation des zones humides méditerranéennes, Le Sambuc-Arles, France; 10 Institut Méditerranéen de Biodiversité et Ecologie (IMBE), UMR CNRS 7263-IRD 237, Université d'Avignon et des pays du Vaucluse, Aix-Marseille Université, IUT d’Avignon, Avignon, France; 11 The Biodesign Center for Fundamental and Applied Microbiomics, Center for Evolution and Medicine, School of Life Sciences, Arizona State University, Tempe, AZ 85287-5001, USA; 12 School of Biological Sciences and Biomolecular Interaction Centre, University of Canterbury, Christchurch, New Zealand; 13 Structural Biology Research Unit, University of Cape Town, Observatory, South Africa; 14 South African Medical Research Council Bioinformatics Unit, South African National Bioinformatics Institute, University of the Western Cape, South Africa; 15 Graduate Program in Ecology, Evolutionary Biology and Behavior, Michigan State University, East Lansing, Michigan, USA

## Abstract

Disease emergence events regularly result from human activities such as agriculture, which frequently brings large populations of genetically uniform hosts into contact with potential pathogens. Although viruses cause nearly 50% of emerging plant diseases, there is little systematic information about virus distribution across agro-ecological interfaces and large gaps in understanding of virus diversity in nature. Here we applied a novel landscape-scale geometagenomics approach to examine relationships between agricultural land use and distributions of plant-associated viruses in two Mediterranean-climate biodiversity hotspots (Western Cape region of South Africa and Rhône river delta region of France). In total, we analysed 1725 geo-referenced plant samples collected over two years from 4.5 × 4.5 km^2^ grids spanning farmlands and adjacent uncultivated vegetation. We found substantial virus prevalence (25.8–35.7%) in all ecosystems, but prevalence and identified family-level virus diversity were greatest in cultivated areas, with some virus families displaying strong agricultural associations. Our survey revealed 94 previously unknown virus species, primarily from uncultivated plants. This is the first effort to systematically evaluate plant-associated viromes across broad agro-ecological interfaces. Our findings indicate that agriculture substantially influences plant virus distributions and highlight the extent of current ignorance about the diversity and roles of viruses in nature.

## Introduction

Over the next 30 years the world’s human population is expected to increase by 33%, reaching 9.7 billion by 2050 (Department of Economic and Social Affairs, 2015). Increasing food demands that accompany this population growth will continue to drive the conversion of natural areas into intensively managed farmlands (Scherr and Mcneely, 2008). Such land cover change will create numerous opportunities for novel interactions between exotic crop species and resident microbial communities (Burdon *et al.*, 2006; Jones, 2009; Alexander *et al.*, 2014), occasionally leading to outbreaks of previously unknown microbial pathogens (Thresh, 1981; Varsani *et al.*, 2008; Jones, 2009).

Although almost 50% of the microbes responsible for emerging plant diseases are viruses (Anderson *et al.*, 2004), there are major gaps to understand plant virus pathogenesis. One crucial missing component is comprehensive information about the spatial and temporal distributions of plant virus populations existing within different vegetation compartments of agro-ecological landscapes. While crops are occasionally surveyed for suites of specific viruses, little is known about the identities of viruses that inhabit interfaces between managed and natural areas (Roossinck and Garcia-Arenal, 2015). For example, ~1200 plant virus species are currently recognised by the International Committee on Taxonomy of Viruses (King *et al.*, 2012), fewer than 10% have been isolated from uncultivated plant species (Wren *et al.*, 2006; Roossinck *et al.*, 2015).

To develop mechanistic understanding of how viral pathogens might emerge in crops, it is essential to identify the parameters that determine viral diversity and prevalence across agro-ecological interfaces. Initial work in other situations indicates, for example, that reductions in plant diversity can increase prevalence of plant pathogens, including some viruses (Mitchell *et al.*, 2002; Allan *et al.*, 2003; Pagan *et al.*, 2012; Lacroix *et al.*, 2014). A central issue is thus the degree to which anthropogenic perturbations of natural ecosystems—such as the ecological simplification and changes in host species resulting from agricultural conversion—favor the appearance of new or specific viruses (Pagan *et al.*, 2012). We therefore ask: (1) Are plant-associated virus communities more prevalent, but less diverse in cultivated areas? (2) Are particular families of viruses significantly associated with cultivated areas? (3) Are novel viruses more likely to be discovered in native uncultivated vegetation? A comprehensive assessment of such relationships across agro-ecological landscapes has never previously been made, but recent methodological developments in spatial plant virus metagenomics provide new means of investigation (Muthukumar *et al.*, 2007; Roossinck *et al.*, 2010). While such approaches have discovered novel viruses within uncultivated plants in several unmanaged ecosystems (Muthukumar *et al.*, 2009; Bernardo *et al.*, 2013), they have yet to be applied across agro-ecological gradients.

Here we use a new geometagenomics approach to assess the spatial and temporal distributions of plant viruses at the landscape scale within two Mediterranean-climate ecosystems: the Western Cape region of South Africa and the Rhône delta river region of France. We examine relationships between land use history at both locations and the distributions of 511 plant samples containing single reads and contigs with detectable similarity to plant-associated viruses identified within 1725 location-tagged plant samples. We find that (1) virus prevalence is greater in cultivated areas in both locations, but that plant diversity is negatively associated with family-level diversity of plant-associated viruses, contrary to expectations; (2) some virus families show strong associations with agriculture; and (3) the novel viruses identified (94 putative species) are primarily from uncultivated plants.

## Materials and methods

### Study sites and geometagenomics sampling grid

**Figure 1:**
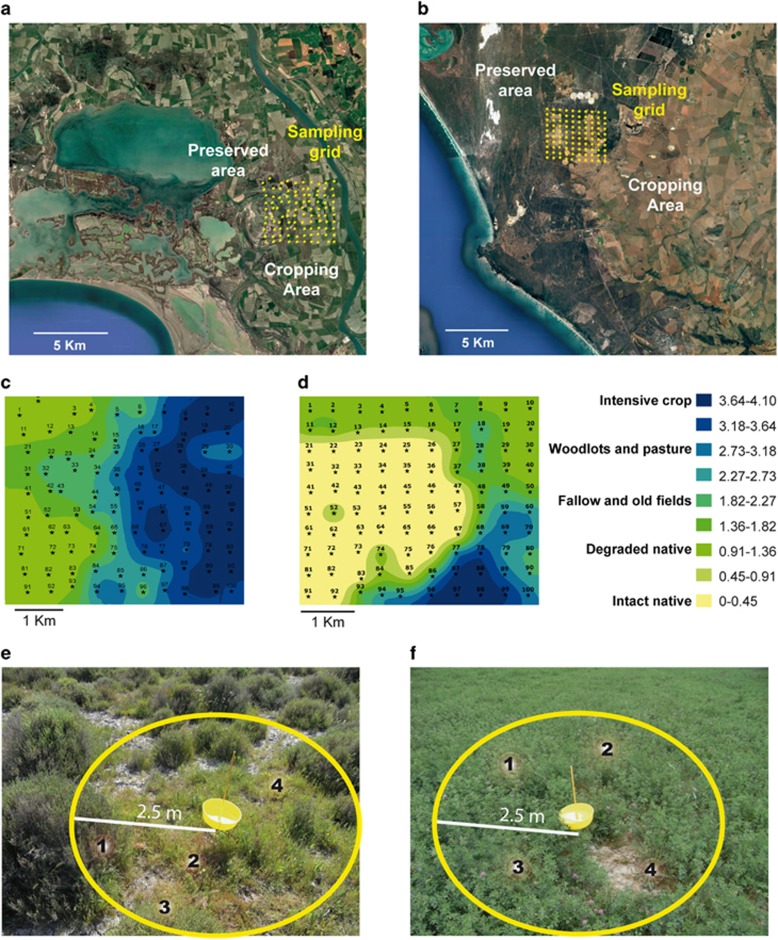
French and South African sampling designs. (**a**) French and (**b**) South African sampling sites. Both 4.5 × 4.5 km^2^ sampling grids contained 100 GPS-nodes with 500 m spacing and were located across agro-ecological interfaces between cultivated and uncultivated areas. Spatial interpolation of degrees of human-mediated disturbance at the (**c**) French and (**d**) South African sampling sites. Empirical Bayesian Kriging was performed based on scores depicting the level of intensity of agriculture using ArcGIS to visualise interfaces between uncultivated and cultivated areas. Every sampling point was ranked as follows: (0) intact native communities; (1) native communities degraded by disturbance or invasion; (2) fallow and old fields; (3) low-intensity polyculture (woodlots and pasture); and (4) intensive crop monoculture. (**e**, **f**) Examples of two geo-nodes at the French sampling site. (**e**) exemplifies an uncultivated sampling point at which four plants (numbers 1–4), each within 2.5 m of the geo-node and with a biomass >10 g, were considered ‘dominant’ and sampled. (**f**) exemplifies a cultivated sampling point, in this case where the vegetation is dominated by alfalfa, at which we collected three separate 5- g alfalfa samples (numbers 1–3) and one (number 4) from a dominant (that is, >10 g biomass) weed.

To quantify landscape-scale patterns of virus distribution, we established permanent 4.5  × 4.5 km^2^ sampling grids across agro-ecological interfaces in two different Mediterranean-climate areas: the Rhône river delta in Southern France and the Cape Floristic Region in South Africa. Both regions represent unique vegetation and have been designated as UNESCO World Heritage Sites (Supplementary Information, Expanded Site Descriptions and Supplementary Table S1). Each grid contained 100 geo-nodes at 500-m spacing (10 nodes × 10 nodes, Figure 1), which were pre-determined using GIS (ArcGIS 10.1, ESRI, Redlands, CA, USA).

In France, the sampling grid spanned an interface between winter wheat, rice and alfalfa fields and the Tour du Valat reserve, which includes a 2600-ha patchwork of seasonal marshes, saline steppes and xero-halophitic meadows. In South Africa, the sampling grid spanned an interface between barley and winter wheat fields to the east and the privately-owned Buffelsfontein Game and Nature Reserve to the west, adjoining the West Coast National Park. This private reserve contains 1600 ha of native strandveld and renosterveld shrublands that are part of the fire-adapted fynbos flora of the Cape Floristic Region—a global biodiversity hotspot (Myers *et al.*, 2000).

### Sampling and measurements at each geo-node

Geo-nodes were sampled in the spring (May–June in France; September–October in South Africa) of 2010 and 2012. We navigated to each geo-node with a Trimble Geo XT V6 (50 cm precision). At each visit, we photographed the geo-node area, rated land use conditions, and collected 5 g of leaf and stem tissue from each dominant plant species for virus analysis. Land use types were rated on a five-point scale: (0) intact native communities; (1) native communities degraded by disturbance or invasion; (2) fallow and old fields; (3) low-intensity polyculture (woodlots and pasture); and (4) intensive crop monoculture. Types 0–1 were considered to be uncultivated (non-agricultural), and types 2–4, cultivated (agricultural).

For virus sampling, we assessed all vegetation within 2.5 m of the geo-node (Figure 1). In multi-strata vegetation, we surveyed all canopy layers. Species for which there was at least 10 g of biomass present were considered ‘dominant’ we generally identified 1–13 dominant species at each geo-node (Figure 1). For each species, we collected a separate sample from a single individual, selected haphazardly without regard to symptoms. To most fully capture virus dynamics, we also sampled small-statured plant species (almost exclusively *Poaceae*); for these, it was necessary to sample from multiple (2–10) individuals to reach the required tissue quantity. In crop monocultures, in which there was little other vegetation, we collected three separate 5-g samples of the crop species (Figure 1). All samples were immediately refrigerated at 4 °C in the field and transported with 4 °C refrigeration to Montpellier, France, where they were kept at −80 °C until processed. Local botanical experts (Yavercovski and Rebelo) confirmed the identities of samples, and categorized them as either crop or non-crop (wild or weedy) species. In 2012, the second set of samples from South Africa was unexpectedly delayed in the middle of air transit and warmed. Thus, these samples are omitted in further analysis.

### Virus extraction, library preparation, and 454 pyrosequencing

To identify known and novel viruses in all 1725 plant samples, we used 454 pyrosequencing of both DNA and RNA extracted from semi-purifications of virion-associated nucleic acids (VANA; Palanga *et al.*, 2016). In this process, each plant species sample from each geo-node was individually bar-coded to allow analysis of relationships between plant and virus species in geographic context. For the VANA semi-purification, 1 g of leaf and stem material from each plant sample was ground and centrifuged twice at low speed (3200*g* for 5 min and 8228*g* for 3 min), filtered through a 0.45 μm sterile syringe filter and centrifuged at 148000*g* for 2.5 h at 4 °C to concentrate viral particles. Unencapsidated nucleic acids were then eliminated by DNase I and RNase A (Euromedex, France). Total nucleic acids were extracted as a mixed RNA/DNA solution from resuspended virus-particles using a NucleoSpin 96 Virus Core Kit (Macherey-Nagel, Germany). In total 23 samples were extracted in each VANA batch along with a dual extraction control: sugarcane tissue infected with a unique, known sugarcane bacilliform badnavirus (from the CIRAD sugarcane quarantine station in Montpellier, France). This dual control served both as a positive with known sequence (the badnavirus) and a negative for the detection of potential contamination (any virus other than the known badnavirus). Before field samples were extracted, sensitivity tests were conducted with 62 different viruses from eleven viral families with a range of genomic nucleic acid types (RNA and DNA), in both single and co-infections; this unique collection of test viruses was available *in vivo* from the CIRAD quarantine station collection (Supplementary Table S2).

For library preparation, complementary DNA (cDNA) synthesis was performed on extracted RNA/DNA solutions using the primer, DoDec (5′-CCT TCG GAT CCT CCN NNN NNN NNN NN-3′). Additional controls of nucleic acid-free water (blanks) were added to each sample group at the reverse transcription step and carried through to sequencing. Priming and extension were then performed with Large (Klenow) Fragment DNA polymerase (Promega, Charbonnières, France) on all cDNA and genomic DNA in each tube. Next, PCR amplification was carried out using one of the 96 multiplex identifier (MID) tagged primers listed in Palanga *et al.* (2016). Finally, libraries were run on a GS FLX Titanium (454 Life Sciences, Branford, CT, USA) by the Beckman Coulter Genomics company (Danvers, MA, USA).

### Processing of 454 reads

MID-tags and primers were identified in each raw read using *agrep* (Wu and Manber, 1992) and assigned to the particular samples from which they originated. Separated raw reads were processed to eliminate MID-tags, primers and low quality regions (Phred quality score threshold of 25) using *cutadapt* (Martin, 2011). Cleaned reads have been deposited in the sequence read archive of GenBank (accession numbers: SAMN05933069–SAMN05933092). We used *BlastN* and *BlastX* to compare contigs assembled with *CAP3* (Huang and Madan, 1999) and non-assembled reads (minimum length 45 bp) to GenBank sequences (Altschul *et al.*, 1990). Whenever query sequences matched a plant-associated virus with an e-value <0.001, we used the *open reading frames Finder* NCBI analysis tool (http://www.ncbi.nlm.nih.gov/gorf/gorf.html) to identify open reading frames that were >20 codons long. For each potential open reading frames, a protein sequence translation was aligned using *MUSCLE 3.7* (default settings; Edgar, 2004) to homologous viral protein sequences identified using *Blast* searches. On the basis of the hosts of their nearest known relatives identified by *BlastN* or *BlastX*, reads and contigs were categorized as likely to represent either plant-infecting viruses (henceforth, plant viruses) or viruses infecting plant-associated fungi (henceforth, mycoviruses). We refer to these plant viruses and mycoviruses collectively as plant-associated viruses (PLAV). Initial classifications may be revised in the future based on further characterization and mycoviruses that are here classified as viruses that probably infect fungi may in fact infect plant hosts and be re-classified as plant viruses. About half of known partitiviruses infect plants, while others infect fungi (Nibert *et al.*, 2014); we classified the partitivirus sequences we found as mycoviruses because pairwise sequence similarity (*BlastN* or *BlastX*) and phylogenetic analyses did not indicate that these reads could be confidently assigned to either one of the two main plant-infecting partitivirus clades (Nibert *et al.*, 2014).

### Estimates of virus prevalence and diversity

To render the most informative ecological snapshot of virus dynamics, we collected tissue from both large- and small-statured plant species; the latter are often overlooked but warrant attention. To reach tissue quantity requirements, several individuals of each small-statured plant species were collected together and combined into a single bulked sample. We therefore define individual prevalence as the number of samples that contained at least one PLAV out of the total number of samples collected from single individuals. We define bulked prevalence as the proportion of PLAV-containing samples among the collection of bulked samples. We define sample prevalence as the prevalence of PLAV in individual and bulked samples considered together. The majority of samples (65.2%) were individual samples. Bulked samples of smaller-statured species were 94% *Poaceae*, 5% *Trifolium spp.* and 1% *Schizaeaceae*. Sample prevalence, bulked prevalence and individual prevalence of cultivated and uncultivated plants were compared using two-tailed Z tests.

To evaluate virus diversity, we first enumerated virus family richness per plant sample. In each sample, we counted only the number of virus families represented (if any), as determined by *Blast* matches, and did not attempt to enumerate genera or species. We adopted this conservative approach because we wanted to avoid ‘over-counting’ different reads or contigs from recombinant viruses as if these were an indication of two separate viruses (a co-infection). To estimate virus diversity at each geo-node, we next calculated the Shannon–Wiener index (*H*), where species richness (*s)* was conservatively estimated by virus family richness and the proportion of individuals belonging to each virus family *i* (*p*_*i*_) was estimated as the number of samples at that geo-node in which the family was represented. Mann–Whitney *U*-tests were used to evaluate differences between cultivated areas and uncultivated areas with respect to viral prevalence and diversity.

### Associations between land use and virus communities

To evaluate potential associations between land use and the identity of virus families at geo-nodes, we used RLQ analysis with the fourth corner method (Dray and Legendre, 2008). With this method, we linked three matrices describing land use variables (R), per-site plant family abundances (L), and virus families detected in each plant family (Q). We used the Hill and Smith approach (Hill and Smith, 1976) to ordinate sites in the R matrix by land-use (Legendre and Legendre, 2012), and principal component analysis (PCA) to analyse the plant family (L) and virus (Q) matrices. Each table was analyzed separately and then compared with results of the three-table RLQ ordination. Statistical significance of the co-structure between land use (R) and virus families (Q) was assessed by comparing total inertia in the RLQ analysis to total inertia after 9999 Monte-Carlo permutations of the rows of the R and Q tables (Doledec *et al.*, 1996). Significance of the association between virus families and land use in the fourth corner analysis (habitat filtering) was evaluated with a two-step testing procedure (Dray and Legendre, 2008). We used 9999 permutations in all randomization procedures and the false discovery rate method (Benjamini and Hochberg, 1995) to adjust *p*-values for multiple testing. All analyses were processed using the *VEGAN* (Dixon, 2003) and *ade4* packages within the *R* statistical computing environment (version 3.0.2).

### Identifying potentially novel viruses

It can be challenging to determine which viral reads and contigs represent novel viruses because of the uneven and crop-biased distribution of GenBank accessions available for comparison, and family-specific differences in how plant virus species are differentiated. We therefore used an approach that combined *BlastN* and *BlastX* determination of sequence similarity with detailed expert review of each taxon identified. On the basis of pairwise sequence similarity, related groups of virus-like sequences (operational taxonomic units, OTUs) were tentatively assigned to known plant virus families. We then generated maximum likelihood phylogenetic trees from alignments of OTU protein sequences with homologous GenBank accessions using *PhyML 3.1* implemented in *MEGA version 6.06* (Tamura *et al.*, 2013) with a JTT amino acid substitution model and 100 bootstrap replicates to quantify branch support. Each OTU-specific tree was individually evaluated to determine whether the OTU-sequences nested within clades containing sequences from the candidate family or appeared to be distinct. Once the most appropriate family was determined, then the OTU was evaluated to determine whether it represented a known species within that family or a potentially novel one. Traditionally, species identities within plant virus families are determined by pairwise sequence identity; viruses are classified as belonging to the same species if their sequence identity is greater than a specific threshold that is particular to each family. To ensure that our *ad hoc* OTU classification system aligned with the diverse classification systems of known virus families, viral OTUs were classified as known species when they shared >75% pairwise amino acid sequence identity with recognized species in GenBank—75% is the approximate consensus of such species identity thresholds in these plant virus families. More divergent OTUs that phylogenetically clustered within known plant virus families, but shared <75% aa sequence identity with any of their known members, were classified as potentially novel species within these families. This OTU classification approach was not used for the mycoviruses because the reads for these viruses were so abundant, divergent and derived from so many different genomic regions that we could not meaningfully align them.

Finally, we tested whether plant virus reads identified in uncultivated plants were less similar to known viruses than those found in cultivated plants. To do this, we portioned plant virus reads according to whether they were obtained from cultivated or uncultivated plants. For each read we took the highest percentage identity *Blast* search result and compared lists of these identities for read from cultivated or uncultivated plants using a Mann–Whitney *U*-test.

## Results and discussion

### Geometagenomics to examine plant virus distributions across the agro-ecological interface

It is well known that viruses cause substantial crop loss and may sometimes be transmitted between crop and non-crop vegetation. We combined the power of next generation sequencing with best practices in ecological sampling to reveal for the first time the broad sweep of virus infections across agro-ecological landscapes. In total, we analysed 1725 plant samples from France (2010, 2012) and South Africa (2010), using VANA semi-purification and 454 pyrosequencing (Table 1), and detected evidence of 757 plant-associated viruses (PLAVs, both plant viruses and mycoviruses) in 29.6% (511 out of 1725) of the samples (Supplementary Table S3). On the basis of BLAST identities to known viruses and phylogenetic analysis, 42% (318 out of 757) of the detected viruses were found to be most similar to plant viruses and 58% (439 out of 757) most similar to mycoviruses. Plant virus sequences proved easier to classify than those that appeared to be mycoviral. Initial classification of the putative mycoviral reads/contigs suggested that they were most similar to known viruses in the families *Partitiviridae* (166 out of 439), *Chrysoviridae* (57 out of 439), and *Totiviridae* (88 out of 439). An additional 128 reads/contigs appeared to represent single stranded DNA (ssDNA) viruses that might infect fungi (Supplementary Table S3 and Supplementary Material).

In sensitivity tests where we analysed plants with known virus infections, test viruses were successfully detected in 75.9% (104 out of 137) of cases. As 7 out of 40 of the test viruses were detected by only a single VANA-read (average length 246–301 nt; Table 1 and Supplementary Table S2), we considered a single VANA-read produced from individual field samples to be credible indication of the presence of a plant-associated virus. The rate of cross-contamination (as assessed with negative controls) was 4.2%. Rates of unanticipated discovery were somewhat higher (8.3%) in positive control plants, suggesting the real presence of previously unrecognized infections of these plants.

### Significantly higher viral prevalence in agricultural areas

Smaller-scale metagenomics surveys of plant viromes in natural environments have indicated that ∼70% of plant samples in Costa Rican forests (Roossinck *et al.*, 2010) and ∼25% of individual plants on an Oklahoma prairie (Muthukumar *et al.*, 2009) harbour identifiable plant viruses. Our estimates of PLAVs prevalence in France (2010: 25.8%, 2012: 35.7%) and South Africa (27.6%) were closer to that of the Oklahoma prairie (Table 1).

However, one issue with our sampling procedure that may have impacted our prevalence estimates is that some samples represented individual plants and others represented multiple plants that had been bulked prior to processing. When we excluded the bulked samples from our data, overall virus prevalence was 18.2% for France in 2010 and 25.7% in 2012 and 21.7% for South Africa in 2010. Although this indicated that the presence of bulked plant samples may have indeed yielded overestimates of prevalence, the prevalence estimates remained similar to those seen in the Oklahoma prairie ecosystem.

**Figure 2:**
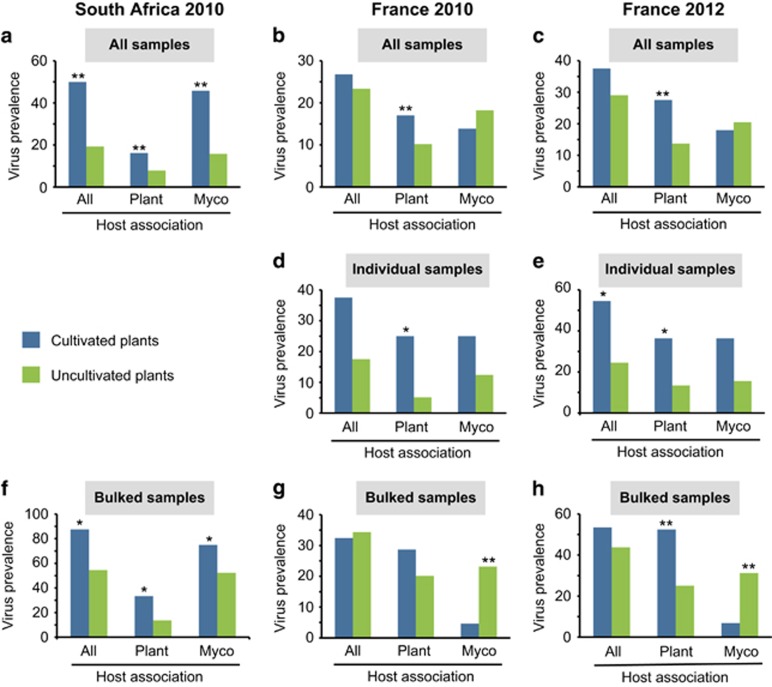
Virus prevalence associated with cultivated and uncultivated plants. Plant virus and mycovirus prevalence within cultivated and uncultivated plants are indicated in blue and light green, respectively. Significant differences in virus prevalence between cultivated and uncultivated plants are indicated by **=p-value <0.01 (two-tailed Z test for two population proportions). In (**a**–**c**) sample infection prevalence is defined as the proportion of plant samples that contained at least one plant-associated virus read or contig (PLAVs). In (**d**, **e**) individual prevalence is defined as the proportion of samples taken from individual plants that contained at least one PLAVs. Note that this comparison could not be made at the South African site because all cultivated plants that were sampled had <5 g of biomass and, as a consequence of this, multiple plants had to be bulked to obtain enough biomass for analysis. In (**f**–**h**) bulked prevalence is defined as the proportion of samples consisting of bulked material from multiple individual plants that contained at least one PLAVs.

We evaluated the extent to which virus prevalence differed between samples collected from cultivated and uncultivated host species. We found that in all surveys the overall proportions of plant samples containing plant viruses were significantly higher (*p*-value <0.01, two-tailed *Z*-test for two population proportions) in cultivated plants relative to uncultivated plants (Figures 2a–c). Irrespective of whether we considered either only bulked samples or only individual plants samples, plant virus prevalence was higher in cultivated plants than in uncultivated plants (Figures 2d–h). This result is consistent with hypotheses relating to host abundance and pathogen prevalence that generally predict increased pathogen prevalence as host abundance increases (Agrawal *et al.*, 2006; Keesing *et al.*, 2010), as it does in many cropping systems.

We also evaluated the extent to which total virus prevalence differed between samples collected from cultivated and uncultivated areas. We found for two out of the three sampling surveys (South Africa, *p*-value <0.01, Mann–Whitney *U*-test, and France 2012, *p*=0.0114, Table 2) that the overall prevalence of plant viruses was significantly higher (*p*-value <0.01, two-tailed *Z*-test for two population proportions) in cultivated areas than it was in uncultivated areas (Table 2).

When the prevalence of plant viruses and mycoviruses were considered separately, a slightly different picture emerged. In France, mycovirus prevalence was highest in both uncultivated plants (Figures 2b and c) and uncultivated areas (Table 2). Interestingly, the mycovirus bulked samples prevalence was higher than the mycovirus individual samples prevalence in uncultivated plants in France (2010, 2012). This suggests that mycoviruses are more concentrated in uncultivated areas than in cultivated ones, which may be attributable to these viruses having different transmission processes to those of plant viruses (insect transmission for plant viruses vs restricted aerial dispersal for mycoviruses).

By contrast, in South Africa, the prevalence of both plant viruses and mycoviruses was highest in both cultivated plants (Figures 2a–f) and cultivated areas (Table 2). It is possible that these differences between the South African and French sampling sites are a consequence of the different disturbance at these sites. In South Africa, the native ‘fynbos’ vegetation burns naturally about every 15 years (van Wilgen, 2009), with the vegetation primarily regrowing from deposited seed. During the last burn at our sampling site in 2000, only the cropping areas remained unburned. It is possible that regular burning of the fynbos might both reduce the prevalence of viruses that infect fynbos species, which are not seedbourne within this vegetation and constrain the dissemination of plant viruses and mycoviruses, although such phenomena have not yet been studied. At the French site, while no extensive burning has occurred within the last 50 years, agriculture-related ecological disturbances have shifted since the 1940s (Supplementary Figure S1) and locations, which, although presently uncultivated, were cultivated between the 1970s and 1990s.

### Virus diversity does not reflect host diversity

The well-known Janzen–Connell hypothesis in ecology posits that pathogens enhance the genetic diversity and structure of host populations in natural ecosystems (Gilbert, 2002). Here we ask the important reciprocal question: Do increases in the diversity of plant hosts drive increases in the diversity of plant viruses (Rottstock *et al.*, 2014)? Our data suggests that this might not be the case for at least one of the three sampling surveys. Specifically, we found at the South African site that the diversity (Shannon–Wiener index) of virus families represented at individual sampling locations was not significantly associated with the diversity of host genera at these locations (Table 2). Further, at the French site, the diversity of virus families was actually greater in cultivated areas (which have lower host diversity) than it was in uncultivated areas in both the 2010 and 2012 sampling surveys (Table 2).

It must, however, be emphasised that the family-level partitioning of virus populations within these analyses limited their power to resolve differences in virus diversity between cultivated and uncultivated areas. Further, it is possible that the proportion of identifiable viruses from uncultivated plant species was lower than that from cultivated species due to biases within GenBank. Our inability to identify highly divergent plant viruses may have been particularly pronounced for the large numbers of indigenous plant species sampled at the South African site since, to our knowledge, prior to our study only a single virus species infecting any of these plants had ever been characterised. Consistent with this possibility, we detected no viruses at the South African site within indigenous plant species in the families *Ebenaceae, Proteaceae* and *Rhamnaceae*. Similar difficulties with the identification of virus-related sequencing reads either from the environment or from uncultivated plant species have been reported elsewhere (Rosario and Breitbart, 2011; Brum *et al.*, 2016). For example, up to 70% of sequence reads generated during some environmental viral metagenomic studies have no detectable homology to sequences within public databases (Rosario and Breitbart, 2011). Here we found that 30.9% of single reads and contigs (Table 1) were not obviously related to any previously submitted GenBank sequences.

Finally, the most obvious bias in any viral metagenomics study is that viral nucleic acids cannot all be isolated with the same efficiency from all environments or hosts. Extreme anatomical and physiological variations between different plant species can strongly impact the ease with which viral nucleic acids can be isolated from different hosts which could in turn bias apparent viral prevalence estimates in favour of the hosts from which nucleic acids are easiest to isolate. Similarly, using isolation procedures that we have employed, the genomic sequences of viruses with more labile capsids should have been more difficult to isolate than those of viruses with stable capsids.

These current limitations of viral metagenomics studies emphasise the possibility that inherent sampling biases, both during the isolation of virus genomic sequences, and within the databases that are used to identify virus-related sequence reads, are likely to result in the underestimation of viral prevalence and diversity.

### Particular virus families significantly associated with agriculture

**Figure 3:**
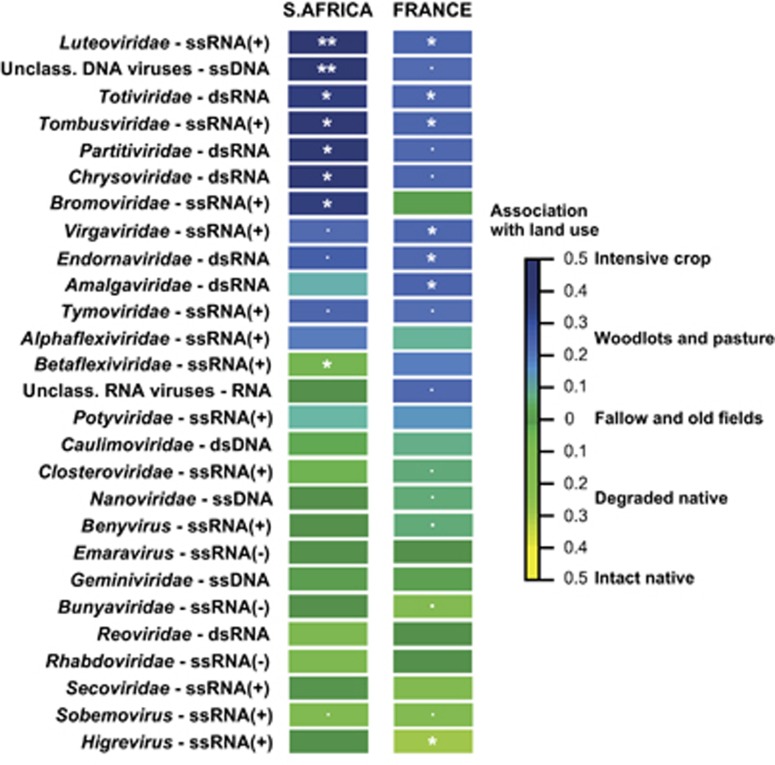
Spatial associations of virus family communities with degrees of land usage. Association of virus communities with land use. The colour gradient represents the Pearson correlation coefficient in the fourth-corner analyses (testing virus vs environment relationships). Significance is indicated by: *p*-value<0.1, *p-value<0.05 and **p-value<0.01.

We investigated the relationships between virus distribution and environmental variables (land use and inter-year effects) in France and South Africa. These analyses indicated that, despite some variability over time, the members of several virus families tended to be found significantly more frequently in cultivated areas than in uncultivated areas (Figure 3). In France, viruses similar to endornaviruses, luteoviruses, virgaviruses, amalgaviruses, tombusviruses and totiviruses were most prevalent in cultivated areas (Figure 3), with the tendency being evident for the first three groups in both sampling years (2010, 2012; Supplementary Figure S2). In South Africa, luteoviruses, tombusviruses and totiviruses were likewise most prevalent in cultivated areas along with bromoviruses, partiviruses, chrysoviruses and unclassified ssDNA viruses.

### Agriculturally important viruses also infect uncultivated plant species

The prevalence of endornavirus-, luteovirus- and virgavirus-like viruses in cultivated areas at the French site suggest that *Poaceae* crops (rice and wheat) there were experiencing recurrent infection with these virus families, including with viruses such as *Barley yellow dwarf virus* (BYDV) and *Barley stripe mosaic virus* (BSMV). Were agricultural pathogens like these persisting between crop cycles within nearby uncultivated plants, and/or spilling over from crops into uncultivated (non-crop) hosts? We found OTUs closely related to 18 known crop pathogens in 37 uncultivated plant hosts (59.5% of which were located in uncultivated areas) at the French site and 10 (20% in uncultivated areas) at the South African site (Supplementary Table S3). These uncultivated plants might act as crop pathogen reservoirs (Cooper and Jones, 2006), or alternatively, these crop-derived infections may detrimentally impact the uncultivated hosts (Jones, 2009, Alexander *et al.*, 2014; Jones and Coutts, 2015). For example, in the Mediterranean-climate regions of Australia, the introduction of the potyvirus, *Bean yellow mosaic virus*, has seriously impacted the indigenous legume, *Kennedia prostata* (Webster *et al.*, 2007).

Interestingly, 7 out of 10 uncultivated plant samples from the South African site that contained OTUs closely related to known plant virus species were from exotic plants (Supplementary Table S3). Likewise, exotic plants in South Africa had a greater prevalence of PLAVs than did indigenous plants (*Z*-Score=7.466, *p*-value=<0.01; Supplementary Figure S3). This difference may be important because in other Mediterranean-climate ecosystem (California, Australia), there has been notable concern about virus exchange between exotic species and indigenous uncultivated plants (Malmstrom *et al.*, 2005a; Webster *et al.*, 2007). In addition, the success of exotic plant species invading new ecosystems can be influenced by their capacity to increase the pathogen loads of the indigenous species with which they compete (Malmstrom *et al.*, 2005b; Borer *et al.*, 2007). On the other hand, plant virus accumulation may, over time, cause declines in the density and distribution of exotic plants and facilitate the recovery of native species (Flory and Clay, 2013).

### Identification of novel viruses

We adopted an approach based on pairwise sequence similarity (*BlastN* or *BlastX*) and phylogenetic analyses to assign related groups of virus-like sequences (OTUs) to known plant virus families (Supplementary Figure S4). Collectively, across all three sampling surveys, 120 plant virus OTUs were identified from 255 of the 1725 analysed plant samples (Supplementary Table S3 and Supplementary Material). Ninety-four of these 120 OTUs share 27–75% identity (median=49%) with known plant virus species and might represent novel species within 19 of the 22 plant virus families currently recognized by the ICTV (Roossinck, 2011, King *et al.*, 2012) or 4 of the 12 recognised but unassigned virus genera (Supplementary Table S3). Furthermore, of the OTUs representing putative novel species, nearly half (45 out of 94) could plausibly represent novel genera within 16 different families (inferred aa sequence identity <50% with any known members of those families). Whereas, 40 out of 45 of these OTUs were found within uncultivated species at the French and South African sites, five were obtained from cultivated species at the French site (Supplementary Table S3).

### Novel plant virus OTUs were mostly from native plants in uncultivated communities

Are viruses found in uncultivated hosts likely to be more dissimilar to known viruses than are viruses detected in cultivated hosts (Roossinck, 2011, 2012)? Whereas 80.9% (76/94) of OTUs representing potential new species were found within uncultivated plants, only 8.6%, (8 out of 94) were found within cultivated plants (Supplementary Table S3). This finding supports the hypothesis that the known plant-infecting virus species are but a tiny fraction of the total occurring in terrestrial environments. It also suggests that our present view of plant-infecting virus diversity is heavily biased in favour of viruses causing recognisable diseases in domesticated plant species (Wren *et al.*, 2006; Roossinck *et al.*, 2015). Although OTUs recovered from uncultivated plants were on average less closely related to known viruses than those recovered from cultivated plants (respectively displaying median identities to a most closely related known virus of 54.8% and 66.8%), this difference was not significant (median identity= *p*-value=0.1187; Mann Whitney *U*-test). This suggests that even in well-studied cultivated host species, there likely remain large numbers of undiscovered plant viruses.

## Conclusion

Our findings reveal the breadth and abundance of plant-infecting viruses in agro-ecological landscapes, where infection is found throughout cultivated and uncultivated plant communities alike. In matched surveys in French and South African sites, we found more than 120 plant virus OTUs representing 19 of the 22 currently recognised plant-infecting virus families. Ninety-four of these OTUs likely represent novel virus species or genera, with potential, when fully characterized, to enlarge the list of known plant virus species by as much as 7.2%. Besides confirming that currently known plant-infecting virus species are likely a tiny fraction of the total occurring in terrestrial environments, our spatially-informed metagenomics-based approach has provided the most convincing evidence yet of the impact of agriculture on the distribution, prevalence and diversity of plant viruses in the environment. It remains to be determined whether preferential associations of specific virus groups with ecologically disturbed areas, or increased plant virus prevalence within such areas, increase the probability of pathogen emergence.

## Figures and Tables

**Table 1:** Characteristics of geo-metagenomics samples from grids of 100 geo-nodes and subsequent VANA-based 454 pyrosequencing of extracted and tagged nucleic acids

*Parameter*	*South Africa 2010*	*France 2010*	*France 2012*
Percentage of geo-nodes in cultivated areas	34	72	74
No. of plant samples (from 100 geo-nodes)	706	484	535
No. of plant samples containing multiple individuals of same species	112	242	247
Total no. of VANA-based 454 pyrosequencing reads	1332624	1092351	1282799
No. of reads removed during quality control (%)	208675 (15.7)	160118 (14.7)	135390 (10.6)
No. of good reads	1123949	932233	1147409
Mean no. of good reads per plant sample	1592	1926	2145
Mean length of good reads (bp)	246	301	260
No. of plant-associated virus reads (% of good reads)	18353 (1.9)	21247 (2.3)	29612 (2.4)
No. of plant-associated virus contigs	3175	2185	2450
No. of samples containing plant-associated virus reads or contigs (%)	195 (27.6)	125 (25.8)	191 (35.7)
Percentage of non-identified reads	35.9	31.0	26.0
Percentage of non-identified contigs	43.1	37.4	30.5

**Table 2:** Average Shannon–Wiener index based estimates of diversity of family-level PLAVs (for plant-associated virus sequences) and genus-level plant samples and average prevalence of PLAVs, plant viruses and mycoviruses calculated from the 100 sampling points scored either as uncultivated (72 in France in 2010, 74 in France in 2010 and 34 in South Africa) or cultivated (28 in France in 2010, 26 in France in 2010 and 66 in South Africa)

*Survey*	*PLAVs Shannon–Wiener Index*			*Plants Shannon–Wiener Index*			*PLAVs prevalence*			*Plant virus prevalence*			*Mycovirus prevalence*		
	*Uncult. average*	*Cult. average*	*Z*-*score*	*Uncult. average*	*Cult. average*	*Z*-*score*	*Uncult. average*	*Cult. average*	*Z*-*score*	*Uncult. average*	*Cult. average*	*Z*-*score*	*Uncult. average*	*Cult. average*	*Z*-*score*
C2010	0.27	0.28	−0.21866 (*p*=0.82588)	1.31	0.72	**−5.7568 (*p*<0.01)**	0.22	0.26	0.1471 (*p*=0.88076)	0.07	0.16	1.26029 (*p*=0.20766)	0.16	0.13	−1.28017 (p=0.20054)
C2012	0.52	0.62	0.53043 (*p*=0.59612)	1.15	0.95	**−2.03529 (*p*=0.04136)**	0.30	0.36	1.19446 (*p*=0.23404)	0.15	0.27	**2.52644 (*p*=0.0114)**	0.20	0.18	−1.11195 (p=0.267)
F2010	0.54	0.90	**−2.71775 (*p*<0.01)**	1.67	1.03	**5.93029 (*p*<0.01)**	0.19	0.53	**−5.69745 (*p*<0.01)**	0.08	0.19	**−3.05974 (*p*<0.01)**	0.16	0.47	**−5.2936 (p<0.01)**

Bold text indicates a statistically significant difference with a *P*-value less than 0.05.
