# Supplementary material for: Geometagenomics illuminates the impact of agriculture on the distribution and prevalence of plant viruses at the ecosystem scale
Source: ISME J. 2017 Oct 20;12(1):173–84. doi: 10.1038/ismej.2017.155 (PMC5739011; doi:10.1038/ismej.2017.155)
Supplement: Supplementary Information [file ismej2017155x1.docx]

**Supplemental Information**

**Expanded Site Description**

In France, the Rhône delta sampling grid spanned an interface between winter wheat, rice, and alfalfa fields planted on soils mostly composed of silts and clays. These soils are characterized by a north-south gradient in both salinity (presence of a saline water-table) and particles' size. Agricultural development began in the 16^th^–17^th^ centuries and intensified in the 1940s with rice cultivation. This agricultural development led to the disappearance of some 40,000 ha of natural habitat (40% of the total area) between 1942 and 1984 (Mesleard, 1994). Wheat is planted in fall and harvested in June–July, while rice is planted in May and harvested in September. Alfalfa fields are perennial (3–4 yrs) and cut 4–5 times annually. The Tour du Valat reserve (founded 1954) includes a 2600-ha patchwork of seasonal marshes, saline steppes and xero-halophitic meadows, which are vegetated with *Juncus*, *Salicornia* (Chenopodiaceae), grasses, forbs (*Limonium*, *Trifolium*), small-statured shrubs, and occasional trees (*Tamarix)*. These plant communities are characteristic of Rhône delta landscapes prior to agricultural expansion in the 1950’s. The area was seasonally grazed by sheep in the 19^th^ century and subsequently grazed at low-intensity by Camargue breeds of cattle and horses. Most of the Tour du Valat lies within the Parc Natural Régional de Camargue and the Réserve Naturelle de Camargue, which is recognized as an UNESCO Biosphere Reserve and World Heritage Site for its ecological significance.

In South Africa, the sampling grid spanned an interface between barley and winter wheat fields to the east and the privately-owned Buffelsfontein Game and Nature Reserve to the west, adjoining the West Coast National Park. The agricultural area has been farmed since the 17^th^ century, but soils are frequently shallow with high proportions of stone and gravel and limited water holding capacity; cereals are grown under dryland cultivation or with center-pivot irrigation. Wheat is planted in May–June and harvested in November–December. Adjacent to these croplands, the Buffelsfontein Reserve contains 1600 ha of native strandveld (beach scrub) and renosterveld (rhinocerous bush) shrublands that represent some of the renowned flora of the Cape Floristic Region, which is a global biodiversity hotspot (Myers et al., 2000) and partially protected as an UNESCO World Heritage Site. Strandveld and renosterveld are both endangered components of the broader Fynbos biome, which is characterized by scleropherous, fire-adapted vegetation known for spectacular spring floral displays. Strandveld inhabits sand dunes and is dominated by shrubs, succulents, *Restio* (endemic South African genus), and other forbs. Renosterveld grows on more fertile soils and is dominated by *Elytropappus rhinocerotis* (Asteraceae), other shrubs, and geophytes. In the past, both communities supported large native herbivores, which were surplanted by cattle during European settlement. Currently, the Preserve is grazed by buffalo, antelopes, white rhinoceros, and giraffe; it burned in 2000.

**References**

MESLEARD, F. 1994. Agricultural Abandonment in a Wetland Area - Abandoned Ricefields in the Camargue, France - Can They Be of Value for Conservation. *Environmental Conservation,* 21**,** 354-357.

MYERS, N., MITTERMEIER, R. A., MITTERMEIER, C. G., DA FONSECA, G. A. & KENT, J. 2000. Biodiversity hotspots for conservation priorities. *Nature,* 403**,** 853-858.
