# Supplementary figures and images for: Geometagenomics illuminates the impact of agriculture on the distribution and prevalence of plant viruses at the ecosystem scale
Source: ISME J. 2017 Oct 20;12(1):173–84. doi: 10.1038/ismej.2017.155 (PMC5739011; doi:10.1038/ismej.2017.155)

Supplementary Figure 1

1942

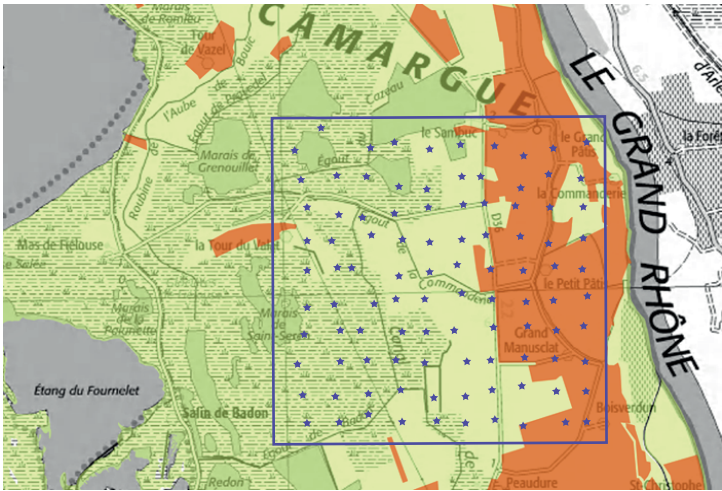

1970

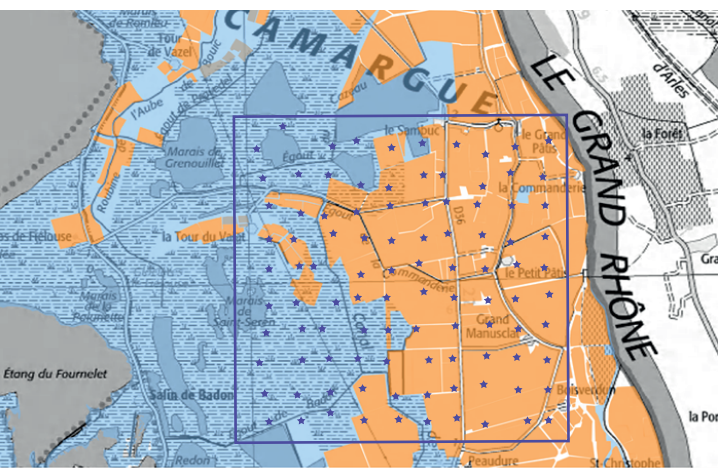

1991

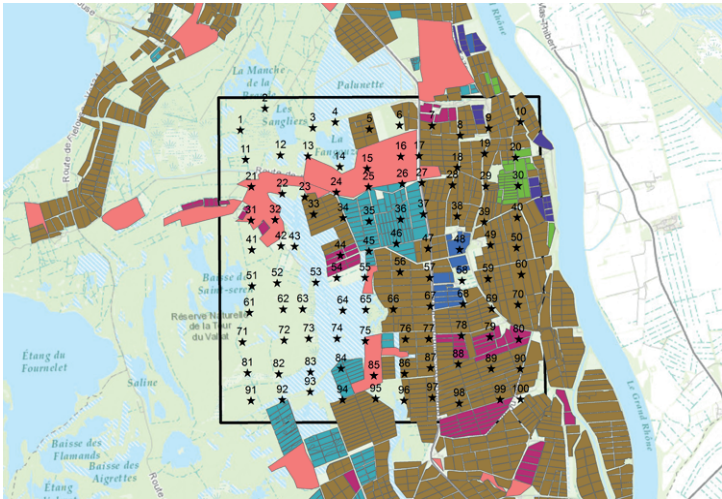

2001

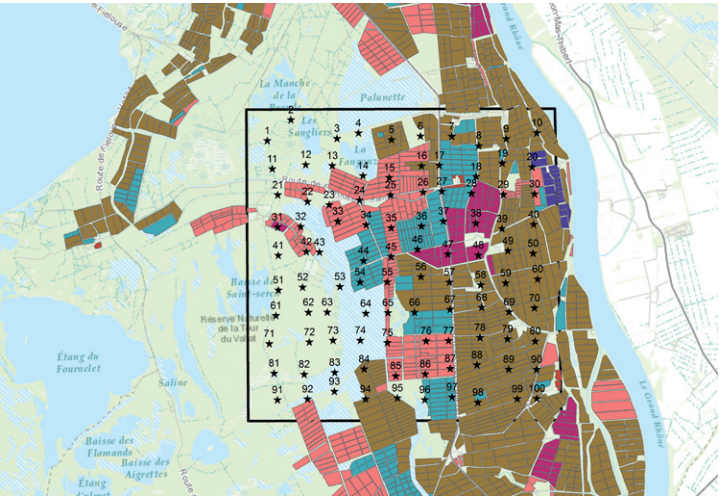

2006

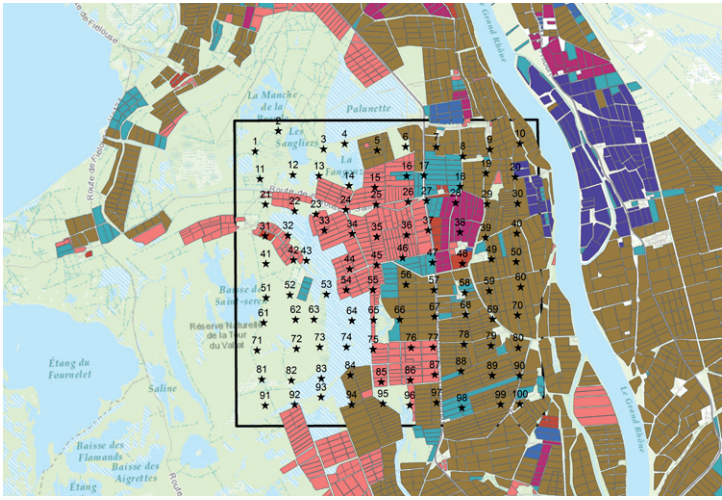

2011

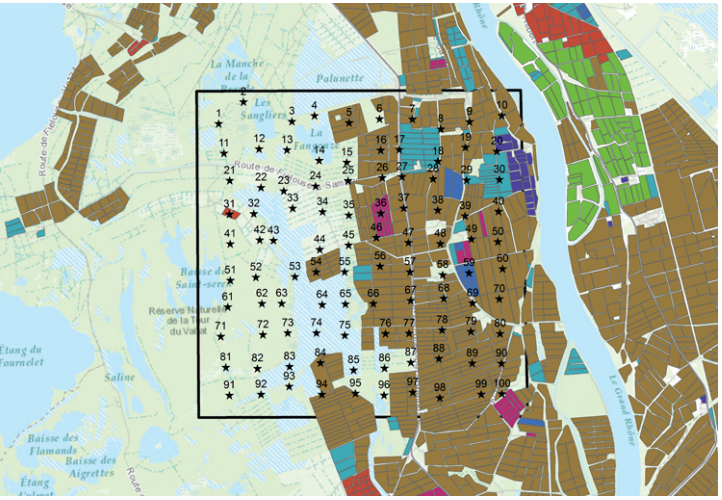

Supplement: Supplementary Figure 1 [file ismej2017155x2.pdf]

Supplementary Figure 2

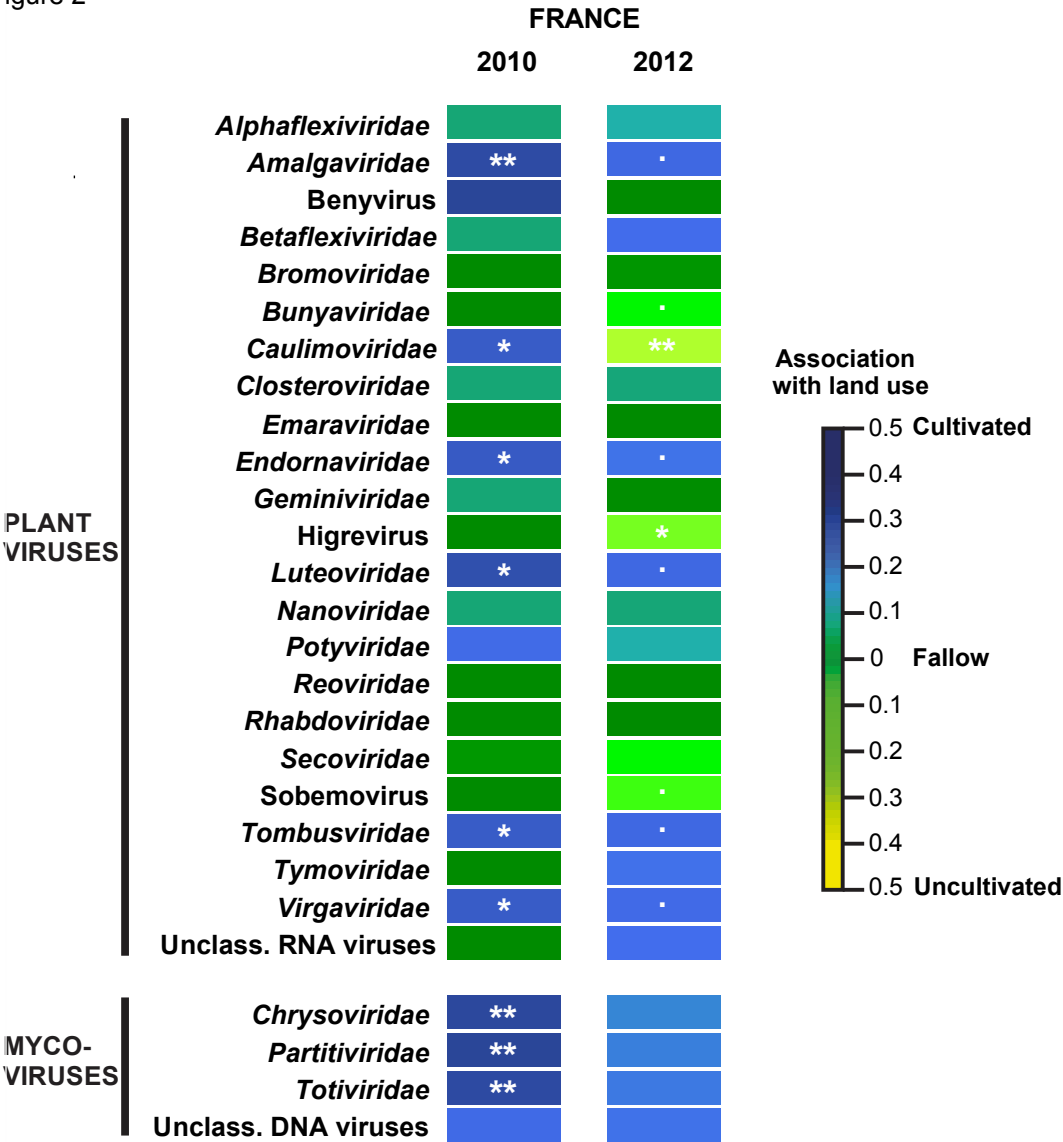

Supplement: Supplementary Figure 2 [file ismej2017155x3.pdf]

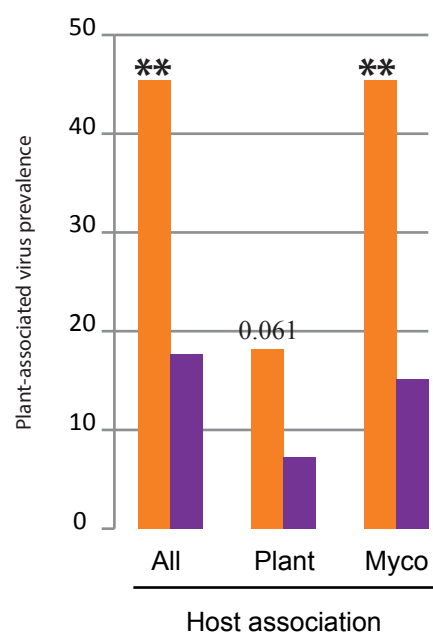

Supplement: Supplementary Figure 3 [file ismej2017155x4.pdf]
