# Supplementary material for: Geometagenomics illuminates the impact of agriculture on the distribution and prevalence of plant viruses at the ecosystem scale
Source: ISME J. 2017 Oct 20;12(1):173–84. doi: 10.1038/ismej.2017.155 (PMC5739011; doi:10.1038/ismej.2017.155)
Supplement: Supplementary Figure 4 [file ismej2017155x5.pdf]

Supplementary Figure 4

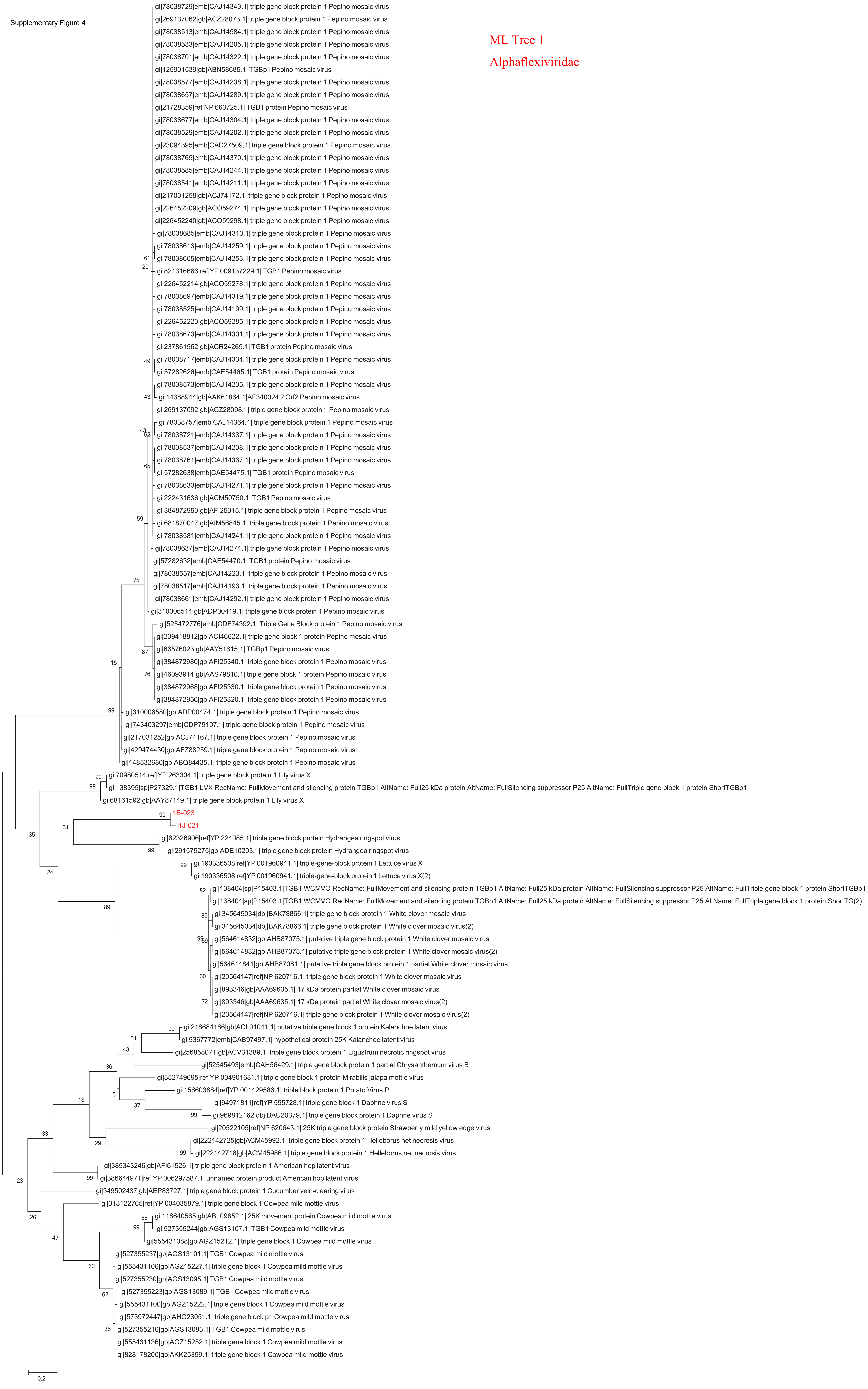

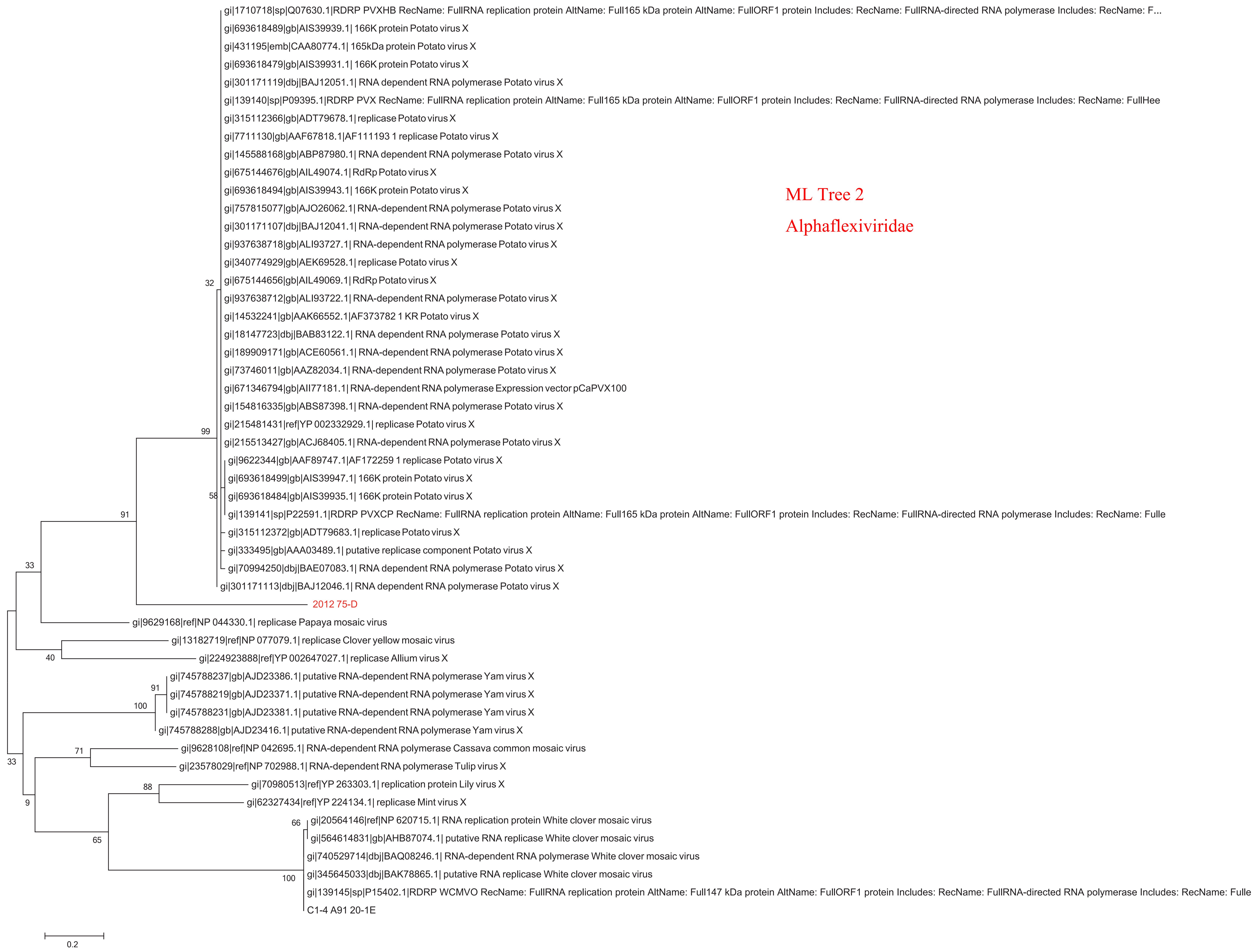
ML Tree 2
Alphaflexiviridae
gi|1710718|sp|Q07630.1|RDRP PVXHB RecName: FullRNA replication protein AltName: Full165 kDa protein AltName: FullORF1 protein Includes: RecName: FullRNA-directed RNA polymerase Includes: RecName: F...
gi|693618489|gb|AIS39939.1| 166K protein Potato virus X
gi|431195|emb|CAA80774.1| 165kDa protein Potato virus X
gi|693618479|gb|AIS39931.1| 166K protein Potato virus X
gi|301171119|dbj|BAJ12051.1| RNA dependent RNA polymerase Potato virus X
gi|139140|sp|P09395.1|RDRP PVX RecName: FullRNA replication protein AltName: Full165 kDa protein AltName: FullORF1 protein Includes: RecName: FullRNA-directed RNA polymerase Includes: RecName: FullHee
gi|315112366|gb|ADT79678.1| replicase Potato virus X
gi|7711130|gb|AAF67818.1|AF111193 1 replicase Potato virus X
gi|145588168|gb|ABP87980.1| RNA dependent RNA polymerase Potato virus X
gi|675144676|gb|AIL49074.1| RdRp Potato virus X
gi|693618494|gb|AIS39943.1| 166K protein Potato virus X
gi|757815077|gb|AJO26062.1| RNA-dependent RNA polymerase Potato virus X
gi|301171107|dbj|BAJ12041.1| RNA-dependent RNA polymerase Potato virus X
gi|937638718|gb|ALI93727.1| RNA-dependent RNA polymerase Potato virus X
gi|340774929|gb|AEK69528.1| replicase Potato virus X
32
gi|675144656|gb|AIL49069.1| RdRp Potato virus X
gi|937638712|gb|ALI93722.1| RNA-dependent RNA polymerase Potato virus X
gi|14532241|gb|AAK66552.1|AF373782 1 KR Potato virus X
gi|18147723|dbj|BAB83122.1| RNA dependent RNA polymerase Potato virus X
gi|189909171|gb|ACE60561.1| RNA-dependent RNA polymerase Potato virus X
gi|73746011|gb|AAZ82034.1| RNA-dependent RNA polymerase Potato virus X
gi|671346794|gb|AII77181.1| RNA-dependent RNA polymerase Expression vector pCaPVX100
gi|154816335|gb|ABS87398.1| RNA-dependent RNA polymerase Potato virus X
gi|215481431|ref|YP 002332929.1| replicase Potato virus X
99
gi|215513427|gb|ACJ68405.1| RNA-dependent RNA polymerase Potato virus X
gi|9622344|gb|AAF89747.1|AF172259 1 replicase Potato virus X
gi|693618499|gb|AIS39947.1| 166K protein Potato virus X
58
gi|693618484|gb|AIS39935.1| 166K protein Potato virus X
91
gi|139141|sp|P22591.1|RDRP PVXCP RecName: FullRNA replication protein AltName: Full165 kDa protein AltName: FullORF1 protein Includes: RecName: FullRNA-directed RNA polymerase Includes: RecName: Fulle
gi|315112372|gb|ADT79683.1| replicase Potato virus X
gi|333495|gb|AAA03489.1| putative replicase component Potato virus X
33
gi|70994250|dbj|BAE07083.1| RNA dependent RNA polymerase Potato virus X
gi|301171113|dbj|BAJ12046.1| RNA dependent RNA polymerase Potato virus X
2012 75-D
gi|9629168|ref|NP 044330.1| replicase Papaya mosaic virus
gi|13182719|ref|NP 077079.1| replicase Clover yellow mosaic virus
40
gi|224923888|ref|YP 002647027.1| replicase Allium virus X
gi|745788237|gb|AJD23386.1| putative RNA-dependent RNA polymerase Yam virus X
91
gi|745788219|gb|AJD23371.1| putative RNA-dependent RNA polymerase Yam virus X
100
gi|745788231|gb|AJD23381.1| putative RNA-dependent RNA polymerase Yam virus X
gi|745788288|gb|AJD23416.1| putative RNA-dependent RNA polymerase Yam virus X
71
gi|9628108|ref|NP 042695.1| RNA-dependent RNA polymerase Cassava common mosaic virus
33
gi|23578029|ref|NP 702988.1| RNA-dependent RNA polymerase Tulip virus X
88
gi|70980513|ref|YP 263303.1| replication protein Lily virus X
9
gi|62327434|ref|YP 224134.1| replicase Mint virus X
66
gi|20564146|ref|NP 620715.1| RNA replication protein White clover mosaic virus
65
gi|564614831|gb|AHB87074.1| putative RNA replicase White clover mosaic virus
gi|740529714|dbj|BAQ08246.1| RNA-dependent RNA polymerase White clover mosaic virus
gi|345645033|dbj|BAK78865.1| putative RNA replicase White clover mosaic virus
100
gi|139145|sp|P15402.1|RDRP WCMVO RecName: FullRNA replication protein AltName: Full147 kDa protein AltName: FullORF1 protein Includes: RecName: FullRNA-directed RNA polymerase Includes: RecName: Fulle
C1-4 A91 20-1E
0.2

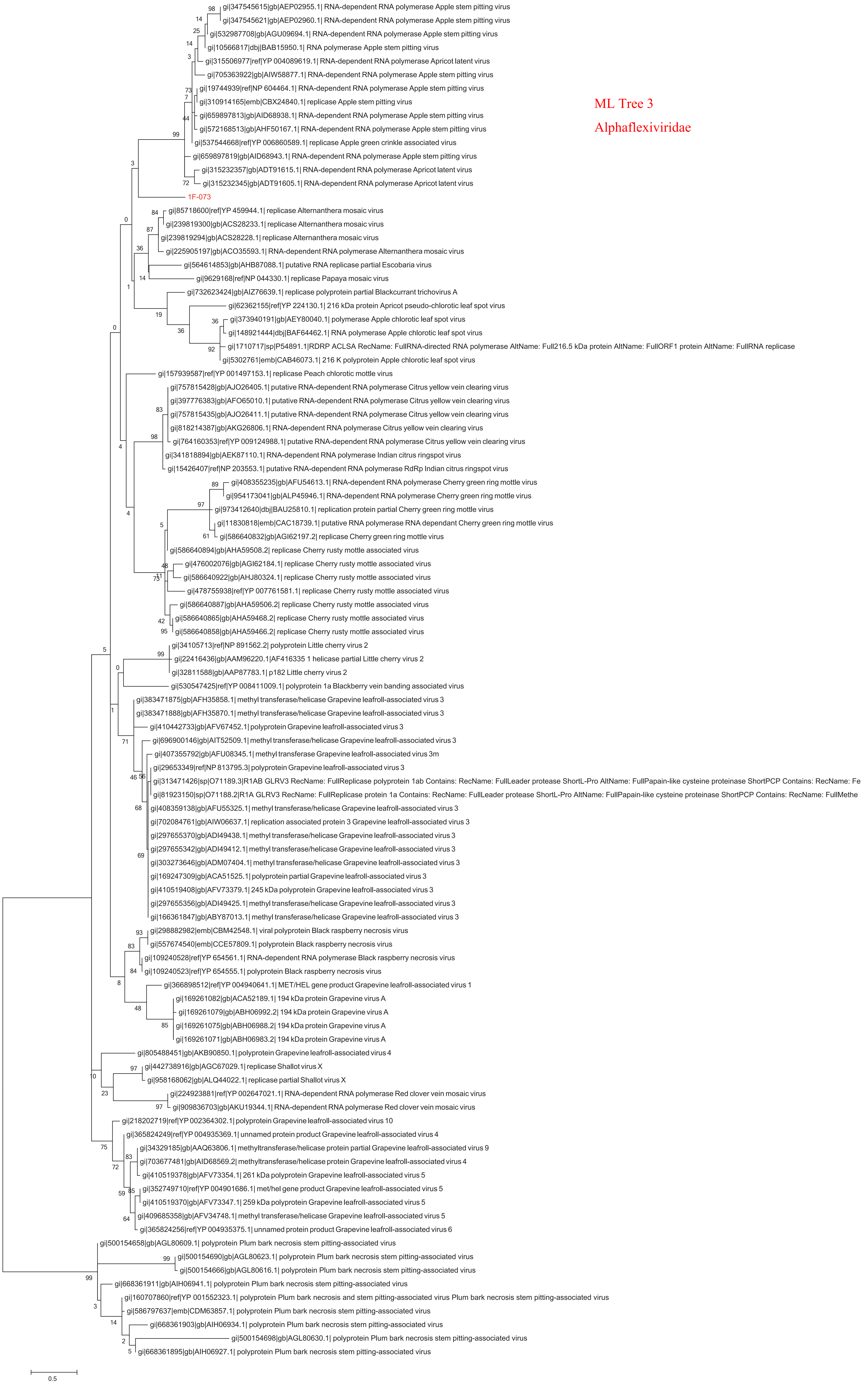

ML Tree 3
Alphaflexiviridae
gi|347545615|gb|AEP02955.1| RNA-dependent RNA polymerase Apple stem pitting virus
gi|347545621|gb|AEP02960.1| RNA-dependent RNA polymerase Apple stem pitting virus
gi|532987708|gb|AGU09694.1| RNA-dependent RNA polymerase Apple stem pitting virus
gi|10566817|dbj|BAB15950.1| RNA polymerase Apple stem pitting virus
gi|315506977|ref|YP 004089619.1| RNA-dependent RNA polymerase Apricot latent virus
gi|705363922|gb|AIW58877.1| RNA-dependent RNA polymerase Apple stem pitting virus
gi|19744939|ref|NP 604464.1| RNA-dependent RNA polymerase Apple stem pitting virus
gi|310914165|emb|CBX24840.1| replicase Apple stem pitting virus
gi|659897813|gb|AID68938.1| RNA-dependent RNA polymerase Apple stem pitting virus
gi|572168513|gb|AHF50167.1| RNA-dependent RNA polymerase Apple stem pitting virus
gi|537544668|ref|YP 006860589.1| replicase Apple green crinkle associated virus
gi|659897819|gb|AID68943.1| RNA-dependent RNA polymerase Apple stem pitting virus
gi|315232357|gb|ADT91615.1| RNA-dependent RNA polymerase Apricot latent virus
gi|315232345|gb|ADT91605.1| RNA-dependent RNA polymerase Apricot latent virus
1F-073
gi|85718600|ref|YP 459944.1| replicase Alternanthera mosaic virus
gi|239819300|gb|ACS28233.1| replicase Alternanthera mosaic virus
gi|239819294|gb|ACS28228.1| replicase Alternanthera mosaic virus
gi|225905197|gb|ACO35593.1| RNA-dependent RNA polymerase Alternanthera mosaic virus
gi|564614853|gb|AHB87088.1| putative RNA replicase partial Escobaria virus
gi|9629168|ref|NP 044330.1| replicase Papaya mosaic virus
gi|732623424|gb|AIZ76639.1| replicase polyprotein partial Blackcurrant trichovirus A
gi|62362155|ref|YP 224130.1| 216 kDa protein Apricot pseudo-chlorotic leaf spot virus
gi|373940191|gb|AEY80040.1| polymerase Apple chlorotic leaf spot virus
gi|148921444|dbj|BAF64462.1| RNA polymerase Apple chlorotic leaf spot virus
gi|1710717|sp|P54891.1|RDRP ACLSA RecName: FullRNA-directed RNA polymerase AltName: Full216.5 kDa protein AltName: FullORF1 protein AltName: FullRNA replicase
gi|5302761|emb|CAB46073.1| 216 K polyprotein Apple chlorotic leaf spot virus
gi|157939587|ref|YP 001497153.1| replicase Peach chlorotic mottle virus
gi|757815428|gb|AJO26405.1| putative RNA-dependent RNA polymerase Citrus yellow vein clearing virus
gi|397776383|gb|AFO65010.1| putative RNA-dependent RNA polymerase Citrus yellow vein clearing virus
gi|757815435|gb|AJO26411.1| putative RNA-dependent RNA polymerase Citrus yellow vein clearing virus
gi|818214387|gb|AKG26806.1| RNA-dependent RNA polymerase Citrus yellow vein clearing virus
gi|764160353|ref|YP 009124988.1| putative RNA-dependent RNA polymerase Citrus yellow vein clearing virus
gi|341818894|gb|AEK87110.1| RNA-dependent RNA polymerase Indian citrus ringspot virus
gi|15426407|ref|NP 203553.1| putative RNA-dependent RNA polymerase RdRp Indian citrus ringspot virus
gi|408355235|gb|AFU54613.1| RNA-dependent RNA polymerase Cherry green ring mottle virus
gi|954173041|gb|ALP45946.1| RNA-dependent RNA polymerase Cherry green ring mottle virus
gi|973412640|dbj|BAU25810.1| replication protein partial Cherry green ring mottle virus
gi|11830818|emb|CAC18739.1| putative RNA polymerase RNA dependant Cherry green ring mottle virus
gi|586640832|gb|AGI62197.2| replicase Cherry green ring mottle virus
gi|586640894|gb|AHA59508.2| replicase Cherry rusty mottle associated virus
gi|476002076|gb|AGI62184.1| replicase Cherry rusty mottle associated virus
gi|586640922|gb|AHJ80324.1| replicase Cherry rusty mottle associated virus
gi|478755938|ref|YP 007761581.1| replicase Cherry rusty mottle associated virus
gi|586640887|gb|AHA59506.2| replicase Cherry rusty mottle associated virus
gi|586640865|gb|AHA59468.2| replicase Cherry rusty mottle associated virus
gi|586640858|gb|AHA59466.2| replicase Cherry rusty mottle associated virus
gi|34105713|ref|NP 891562.2| polyprotein Little cherry virus 2
gi|22416436|gb|AAM96220.1|AF416335 1 helicase partial Little cherry virus 2
gi|32811588|gb|AAP87783.1| p182 Little cherry virus 2
gi|530547425|ref|YP 008411009.1| polyprotein 1a Blackberry vein banding associated virus
gi|383471875|gb|AFH35858.1| methyl transferase/helicase Grapevine leafroll-associated virus 3
gi|383471888|gb|AFH35870.1| methyl transferase/helicase Grapevine leafroll-associated virus 3
gi|410442733|gb|AFV67452.1| polyprotein Grapevine leafroll-associated virus 3
gi|696900146|gb|AIT52509.1| methyl transferase/helicase Grapevine leafroll-associated virus 3
gi|407355792|gb|AFU08345.1| methyl transferase Grapevine leafroll-associated virus 3m
gi|29653349|ref|NP 813795.3| polyprotein Grapevine leafroll-associated virus 3
gi|313471426|sp|O71189.3|R1AB GLRV3 RecName: FullReplicase polyprotein 1ab Contains: RecName: FullLeader protease ShortL-Pro AltName: FullPapain-like cysteine proteinase ShortPCP Contains: RecName: Fe
gi|81923150|sp|O71188.2|R1A GLRV3 RecName: FullReplicase protein 1a Contains: RecName: FullLeader protease ShortL-Pro AltName: FullPapain-like cysteine proteinase ShortPCP Contains: RecName: FullMethe
gi|408359138|gb|AFU55325.1| methyl transferase/helicase Grapevine leafroll-associated virus 3
gi|702084761|gb|AIW06637.1| replication associated protein 3 Grapevine leafroll-associated virus 3
gi|297655370|gb|ADI49438.1| methyl transferase/helicase Grapevine leafroll-associated virus 3
gi|297655342|gb|ADI49412.1| methyl transferase/helicase Grapevine leafroll-associated virus 3
gi|303273646|gb|ADM07404.1| methyl transferase/helicase Grapevine leafroll-associated virus 3
gi|169247309|gb|ACA51525.1| polyprotein partial Grapevine leafroll-associated virus 3
gi|410519408|gb|AFV73379.1| 245 kDa polyprotein Grapevine leafroll-associated virus 3
gi|297655356|gb|ADI49425.1| methyl transferase/helicase Grapevine leafroll-associated virus 3
gi|166361847|gb|ABY87013.1| methyl transferase/helicase Grapevine leafroll-associated virus 3
gi|298882982|emb|CBM42548.1| viral polyprotein Black raspberry necrosis virus
gi|557674540|emb|CCE57809.1| polyprotein Black raspberry necrosis virus
gi|109240528|ref|YP 654561.1| RNA-dependent RNA polymerase Black raspberry necrosis virus
gi|109240523|ref|YP 654555.1| polyprotein Black raspberry necrosis virus
gi|366898512|ref|YP 004940641.1| MET/HEL gene product Grapevine leafroll-associated virus 1
gi|169261082|gb|ACA52189.1| 194 kDa protein Grapevine virus A
gi|169261079|gb|ABH06992.2| 194 kDa protein Grapevine virus A
gi|169261075|gb|ABH06988.2| 194 kDa protein Grapevine virus A
gi|169261071|gb|ABH06983.2| 194 kDa protein Grapevine virus A
gi|805488451|gb|AKB90850.1| polyprotein Grapevine leafroll-associated virus 4
gi|442738916|gb|AGC67029.1| replicase Shallot virus X
gi|958168062|gb|ALQ44022.1| replicase partial Shallot virus X
gi|224923881|ref|YP 002647021.1| RNA-dependent RNA polymerase Red clover vein mosaic virus
gi|909836703|gb|AKU19344.1| RNA-dependent RNA polymerase Red clover vein mosaic virus
gi|218202719|ref|YP 002364302.1| polyprotein Grapevine leafroll-associated virus 10
gi|365824249|ref|YP 004935369.1| unnamed protein product Grapevine leafroll-associated virus 4
gi|34329185|gb|AAQ63806.1| methyltransferase/helicase protein partial Grapevine leafroll-associated virus 9
gi|703677481|gb|AID68569.2| methyltransferase/helicase protein Grapevine leafroll-associated virus 4
gi|410519378|gb|AFV73354.1| 261 kDa polyprotein Grapevine leafroll-associated virus 5
gi|352749710|ref|YP 004901686.1| met/hel gene product Grapevine leafroll-associated virus 5
gi|410519370|gb|AFV73347.1| 259 kDa polyprotein Grapevine leafroll-associated virus 5
gi|409685358|gb|AFV34748.1| methyl transferase/helicase Grapevine leafroll-associated virus 5
gi|365824256|ref|YP 004935375.1| unnamed protein product Grapevine leafroll-associated virus 6
gi|500154658|gb|AGL80609.1| polyprotein Plum bark necrosis stem pitting-associated virus
gi|500154690|gb|AGL80623.1| polyprotein Plum bark necrosis stem pitting-associated virus
gi|500154666|gb|AGL80616.1| polyprotein Plum bark necrosis stem pitting-associated virus
gi|668361911|gb|AIH06941.1| polyprotein Plum bark necrosis stem pitting-associated virus
gi|160707860|ref|YP 001552323.1| polyprotein Plum bark necrosis and stem pitting-associated virus Plum bark necrosis stem pitting-associated virus
gi|586797637|emb|CDM63857.1| polyprotein Plum bark necrosis stem pitting-associated virus
gi|668361903|gb|AIH06934.1| polyprotein Plum bark necrosis stem pitting-associated virus
gi|500154698|gb|AGL80630.1| polyprotein Plum bark necrosis stem pitting-associated virus
gi|668361895|gb|AIH06927.1| polyprotein Plum bark necrosis stem pitting-associated virus
0.5

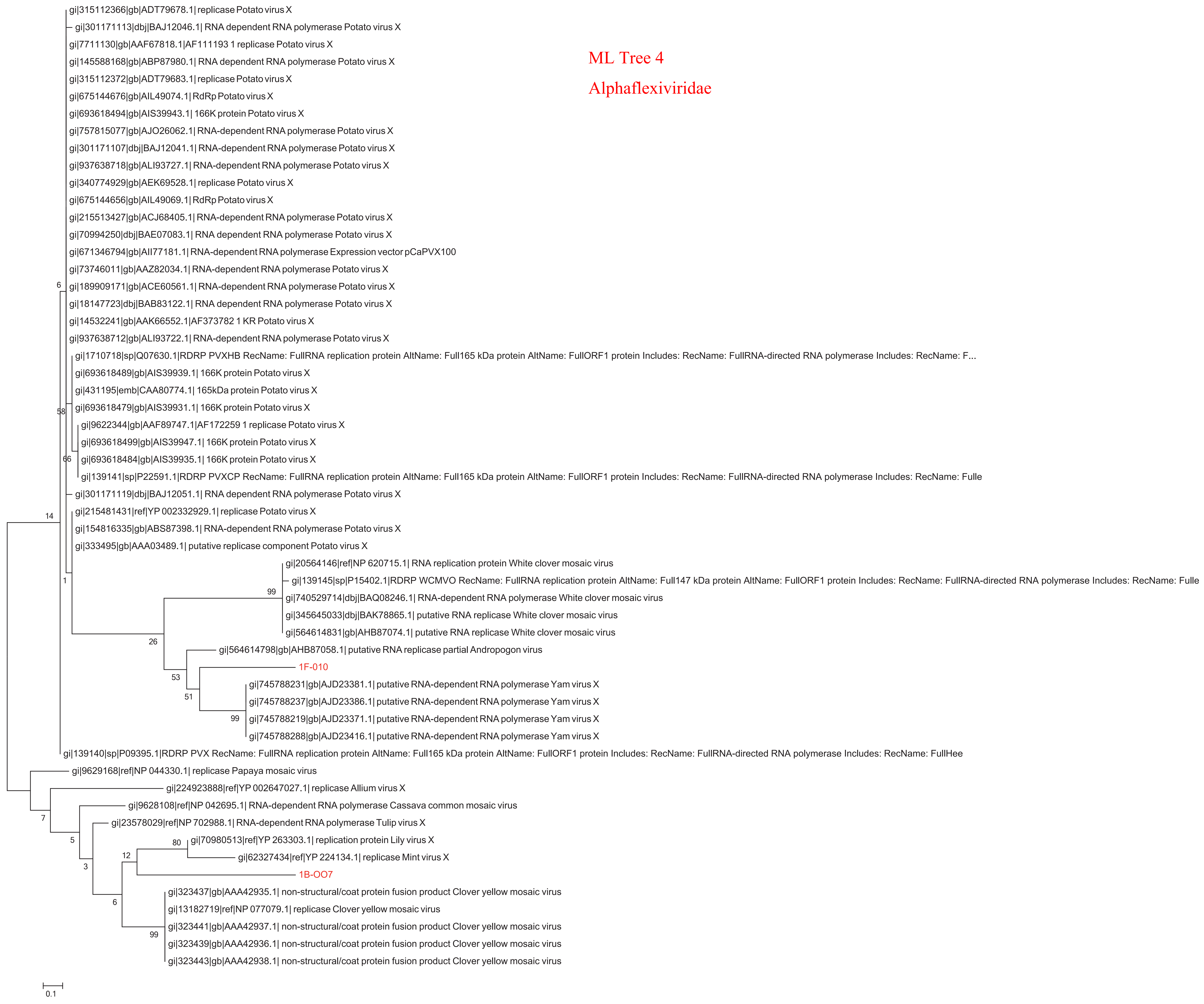
ML Tree 4
Alphaflexiviridae
gi|315112366|gb|ADT79678.1| replicase Potato virus X
gi|301171113|dbj|BAJ12046.1| RNA dependent RNA polymerase Potato virus X
gi|7711130|gb|AAF67818.1|AF111193 1 replicase Potato virus X
gi|145588168|gb|ABP87980.1| RNA dependent RNA polymerase Potato virus X
gi|315112372|gb|ADT79683.1| replicase Potato virus X
gi|675144676|gb|AIL49074.1| RdRp Potato virus X
gi|693618494|gb|AIS39943.1| 166K protein Potato virus X
gi|757815077|gb|AJO26062.1| RNA-dependent RNA polymerase Potato virus X
gi|301171107|dbj|BAJ12041.1| RNA-dependent RNA polymerase Potato virus X
gi|937638718|gb|ALI93727.1| RNA-dependent RNA polymerase Potato virus X
gi|340774929|gb|AEK69528.1| replicase Potato virus X
gi|675144656|gb|AIL49069.1| RdRp Potato virus X
gi|215513427|gb|ACJ68405.1| RNA-dependent RNA polymerase Potato virus X
gi|70994250|dbj|BAE07083.1| RNA dependent RNA polymerase Potato virus X
gi|671346794|gb|AII77181.1| RNA-dependent RNA polymerase Expression vector pCaPVX100
gi|73746011|gb|AAZ82034.1| RNA-dependent RNA polymerase Potato virus X
gi|189909171|gb|ACE60561.1| RNA-dependent RNA polymerase Potato virus X
gi|18147723|dbj|BAB83122.1| RNA dependent RNA polymerase Potato virus X
gi|14532241|gb|AAK66552.1|AF373782 1 KR Potato virus X
gi|937638712|gb|ALI93722.1| RNA-dependent RNA polymerase Potato virus X
gi|1710718|sp|Q07630.1|RDRP PVXHB RecName: FullRNA replication protein AltName: Full165 kDa protein AltName: FullORF1 protein Includes: RecName: FullRNA-directed RNA polymerase Includes: RecName: F...
gi|693618489|gb|AIS39939.1| 166K protein Potato virus X
gi|431195|emb|CAA80774.1| 165kDa protein Potato virus X
gi|693618479|gb|AIS39931.1| 166K protein Potato virus X
gi|9622344|gb|AAF89747.1|AF172259 1 replicase Potato virus X
gi|693618499|gb|AIS39947.1| 166K protein Potato virus X
gi|693618484|gb|AIS39935.1| 166K protein Potato virus X
gi|139141|sp|P22591.1|RDRP PVXCP RecName: FullRNA replication protein AltName: Full165 kDa protein AltName: FullORF1 protein Includes: RecName: FullRNA-directed RNA polymerase Includes: RecName: Fulle
gi|301171119|dbj|BAJ12051.1| RNA dependent RNA polymerase Potato virus X
gi|215481431|ref|YP 002332929.1| replicase Potato virus X
gi|154816335|gb|ABS87398.1| RNA-dependent RNA polymerase Potato virus X
gi|333495|gb|AAA03489.1| putative replicase component Potato virus X
gi|20564146|ref|NP 620715.1| RNA replication protein White clover mosaic virus
gi|139145|sp|P15402.1|RDRP WCMVO RecName: FullRNA replication protein AltName: Full147 kDa protein AltName: FullORF1 protein Includes: RecName: FullRNA-directed RNA polymerase Includes: RecName: Fulle
gi|740529714|dbj|BAQ08246.1| RNA-dependent RNA polymerase White clover mosaic virus
gi|345645033|dbj|BAK78865.1| putative RNA replicase White clover mosaic virus
gi|564614831|gb|AHB87074.1| putative RNA replicase White clover mosaic virus
gi|564614798|gb|AHB87058.1| putative RNA replicase partial Andropogon virus
1F-010
gi|745788231|gb|AJD23381.1| putative RNA-dependent RNA polymerase Yam virus X
gi|745788237|gb|AJD23386.1| putative RNA-dependent RNA polymerase Yam virus X
gi|745788219|gb|AJD23371.1| putative RNA-dependent RNA polymerase Yam virus X
gi|745788288|gb|AJD23416.1| putative RNA-dependent RNA polymerase Yam virus X
gi|139140|sp|P09395.1|RDRP PVX RecName: FullRNA replication protein AltName: Full165 kDa protein AltName: FullORF1 protein Includes: RecName: FullRNA-directed RNA polymerase Includes: RecName: FullHee
gi|9629168|ref|NP 044330.1| replicase Papaya mosaic virus
gi|224923888|ref|YP 002647027.1| replicase Allium virus X
gi|9628108|ref|NP 042695.1| RNA-dependent RNA polymerase Cassava common mosaic virus
gi|23578029|ref|NP 702988.1| RNA-dependent RNA polymerase Tulip virus X
gi|70980513|ref|YP 263303.1| replication protein Lily virus X
gi|62327434|ref|YP 224134.1| replicase Mint virus X
1B-OO7
gi|323437|gb|AAA42935.1| non-structural/coat protein fusion product Clover yellow mosaic virus
gi|13182719|ref|NP 077079.1| replicase Clover yellow mosaic virus
gi|323441|gb|AAA42937.1| non-structural/coat protein fusion product Clover yellow mosaic virus
gi|323439|gb|AAA42936.1| non-structural/coat protein fusion product Clover yellow mosaic virus
gi|323443|gb|AAA42938.1| non-structural/coat protein fusion product Clover yellow mosaic virus
0.1

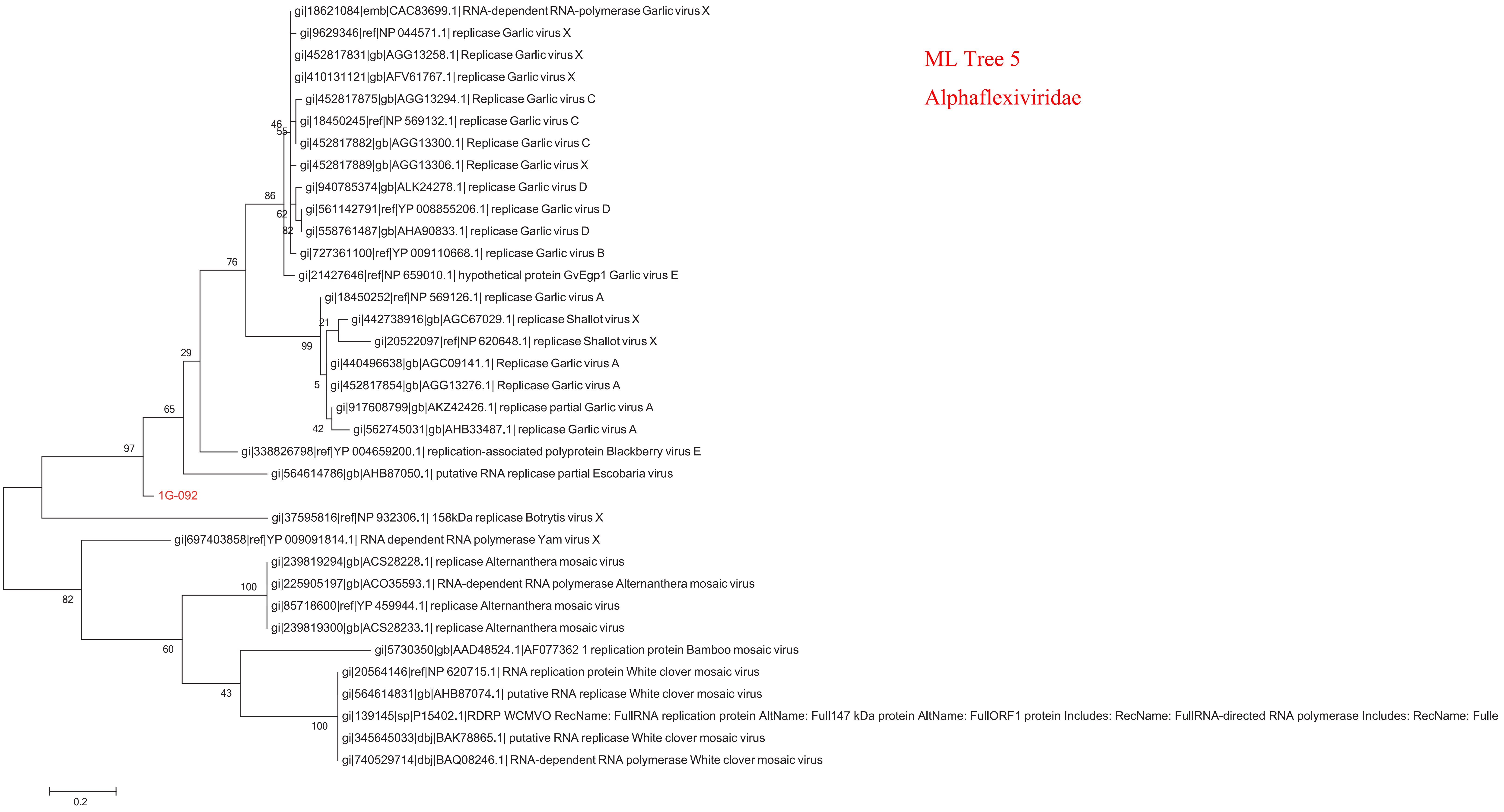
ML Tree 5
Alphaflexiviridae
gi|18621084|emb|CAC83699.1| RNA-dependent RNA-polymerase Garlic virus X
gi|9629346|ref|NP 044571.1| replicase Garlic virus X
gi|452817831|gb|AGG13258.1| Replicase Garlic virus X
gi|410131121|gb|AFV61767.1| replicase Garlic virus X
gi|452817875|gb|AGG13294.1| Replicase Garlic virus C
gi|18450245|ref|NP 569132.1| replicase Garlic virus C
gi|452817882|gb|AGG13300.1| Replicase Garlic virus C
gi|452817889|gb|AGG13306.1| Replicase Garlic virus X
gi|940785374|gb|ALK24278.1| replicase Garlic virus D
gi|561142791|ref|YP 008855206.1| replicase Garlic virus D
gi|558761487|gb|AHA90833.1| replicase Garlic virus D
gi|727361100|ref|YP 009110668.1| replicase Garlic virus B
gi|21427646|ref|NP 659010.1| hypothetical protein GvEgp1 Garlic virus E
gi|18450252|ref|NP 569126.1| replicase Garlic virus A
gi|442738916|gb|AGC67029.1| replicase Shallot virus X
gi|20522097|ref|NP 620648.1| replicase Shallot virus X
gi|440496638|gb|AGC09141.1| Replicase Garlic virus A
gi|452817854|gb|AGG13276.1| Replicase Garlic virus A
gi|917608799|gb|AKZ42426.1| replicase partial Garlic virus A
gi|562745031|gb|AHB33487.1| replicase Garlic virus A
gi|338826798|ref|YP 004659200.1| replication-associated polyprotein Blackberry virus E
gi|564614786|gb|AHB87050.1| putative RNA replicase partial Escobaria virus
1G-092
gi|37595816|ref|NP 932306.1| 158kDa replicase Botrytis virus X
gi|697403858|ref|YP 009091814.1| RNA dependent RNA polymerase Yam virus X
gi|239819294|gb|ACS28228.1| replicase Alternanthera mosaic virus
gi|225905197|gb|ACO35593.1| RNA-dependent RNA polymerase Alternanthera mosaic virus
gi|85718600|ref|YP 459944.1| replicase Alternanthera mosaic virus
gi|239819300|gb|ACS28233.1| replicase Alternanthera mosaic virus
gi|5730350|gb|AAD48524.1|AF077362 1 replication protein Bamboo mosaic virus
gi|20564146|ref|NP 620715.1| RNA replication protein White clover mosaic virus
gi|564614831|gb|AHB87074.1| putative RNA replicase White clover mosaic virus
gi|139145|sp|P15402.1|RDRP WCMVO RecName: FullRNA replication protein AltName: Full147 kDa protein AltName: FullORF1 protein Includes: RecName: FullRNA-directed RNA polymerase Includes: RecName: Fulle
gi|345645033|dbj|BAK78865.1| putative RNA replicase White clover mosaic virus
gi|740529714|dbj|BAQ08246.1| RNA-dependent RNA polymerase White clover mosaic virus
46
55
86
62
82
76
21
99
5
42
29
65
97
82
100
60
43
100
0.2

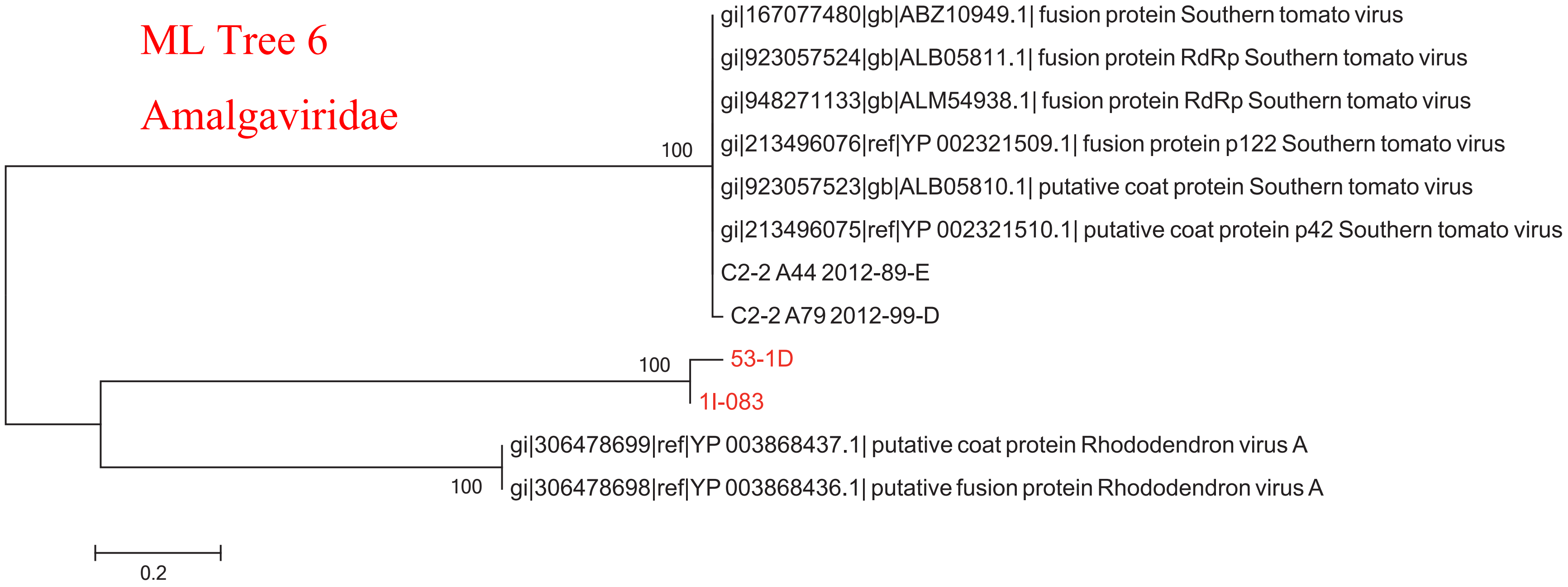
ML Tree 6
Amalgaviridae
gi|167077480|gb|ABZ10949.1| fusion protein Southern tomato virus
gi|923057524|gb|ALB05811.1| fusion protein RdRp Southern tomato virus
gi|948271133|gb|ALM54938.1| fusion protein RdRp Southern tomato virus
gi|213496076|ref|YP 002321509.1| fusion protein p122 Southern tomato virus
gi|923057523|gb|ALB05810.1| putative coat protein Southern tomato virus
gi|213496075|ref|YP 002321510.1| putative coat protein p42 Southern tomato virus
C2-2 A44 2012-89-E
C2-2 A79 2012-99-D
53-1D
1I-083
gi|306478699|ref|YP 003868437.1| putative coat protein Rhododendron virus A
gi|306478698|ref|YP 003868436.1| putative fusion protein Rhododendron virus A
100
100
100
100
0.2

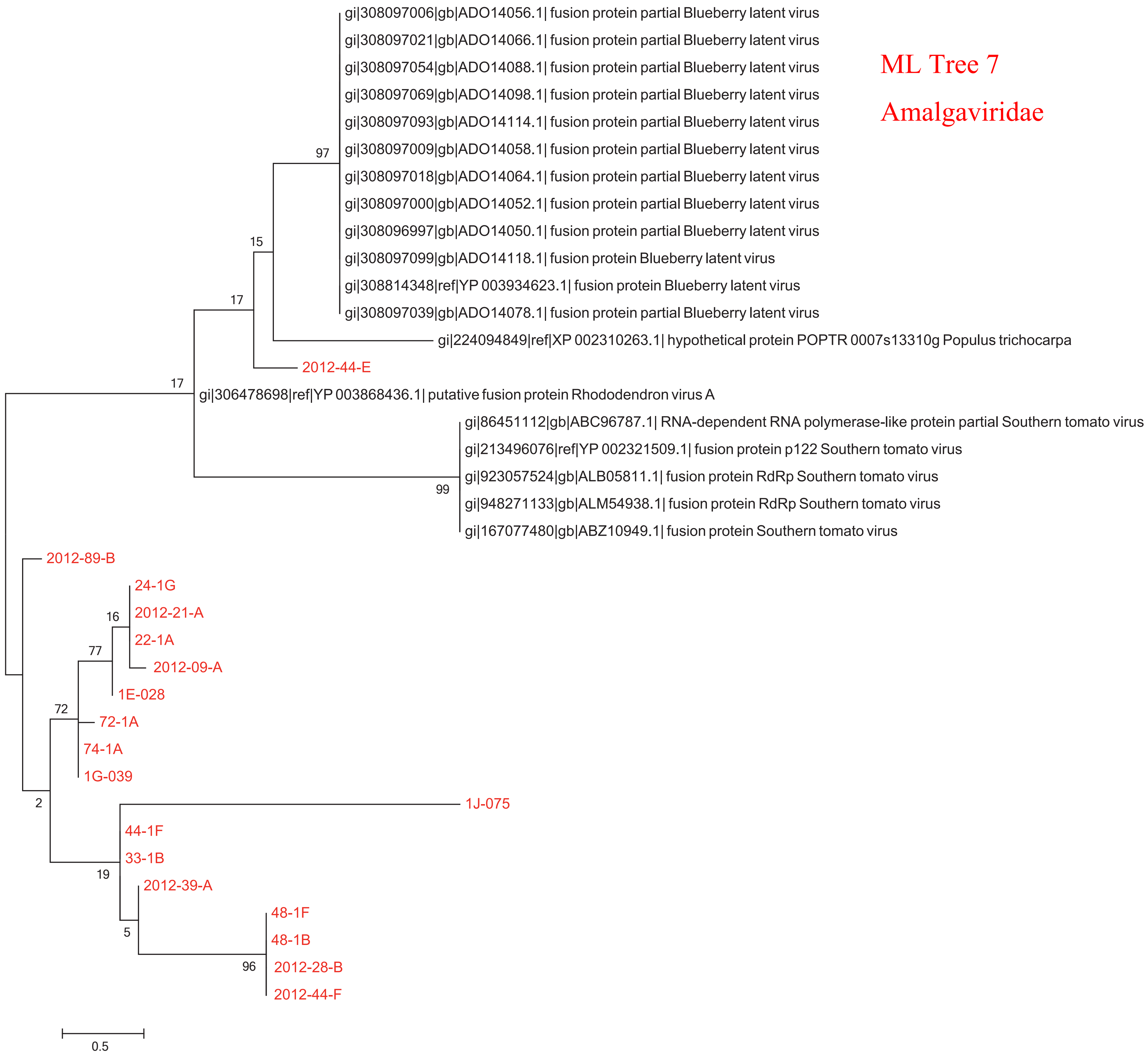
ML Tree 7
Amalgaviridae
gi|308097006|gb|ADO14056.1| fusion protein partial Blueberry latent virus
gi|308097021|gb|ADO14066.1| fusion protein partial Blueberry latent virus
gi|308097054|gb|ADO14088.1| fusion protein partial Blueberry latent virus
gi|308097069|gb|ADO14098.1| fusion protein partial Blueberry latent virus
gi|308097093|gb|ADO14114.1| fusion protein partial Blueberry latent virus
gi|308097009|gb|ADO14058.1| fusion protein partial Blueberry latent virus
gi|308097018|gb|ADO14064.1| fusion protein partial Blueberry latent virus
gi|308097000|gb|ADO14052.1| fusion protein partial Blueberry latent virus
gi|308096997|gb|ADO14050.1| fusion protein partial Blueberry latent virus
gi|308097099|gb|ADO14118.1| fusion protein Blueberry latent virus
gi|308814348|ref|YP 003934623.1| fusion protein Blueberry latent virus
gi|308097039|gb|ADO14078.1| fusion protein partial Blueberry latent virus
97
15
17
17
gi|224094849|ref|XP 002310263.1| hypothetical protein POPTR 0007s13310g Populus trichocarpa
2012-44-E
gi|306478698|ref|YP 003868436.1| putative fusion protein Rhododendron virus A
gi|86451112|gb|ABC96787.1| RNA-dependent RNA polymerase-like protein partial Southern tomato virus
gi|213496076|ref|YP 002321509.1| fusion protein p122 Southern tomato virus
gi|923057524|gb|ALB05811.1| fusion protein RdRp Southern tomato virus
gi|948271133|gb|ALM54938.1| fusion protein RdRp Southern tomato virus
gi|167077480|gb|ABZ10949.1| fusion protein Southern tomato virus
99
2012-89-B
24-1G
2012-21-A
22-1A
2012-09-A
1E-028
16
77
72
72-1A
74-1A
1G-039
2
1J-075
44-1F
33-1B
19
2012-39-A
5
48-1F
48-1B
2012-28-B
2012-44-F
96
0.5

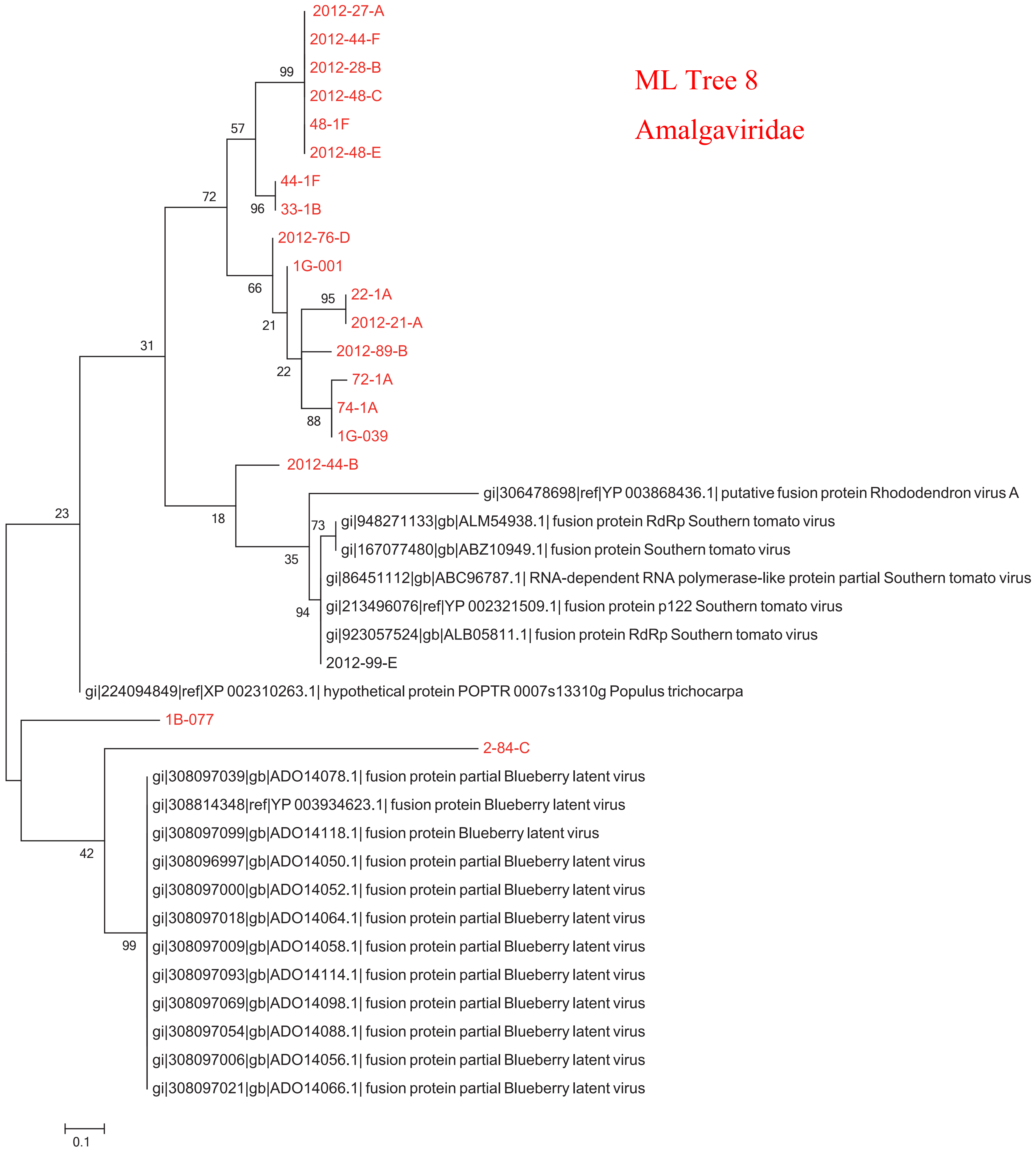
ML Tree 8
Amalgaviridae
2012-27-A
2012-44-F
2012-28-B
2012-48-C
48-1F
2012-48-E
44-1F
33-1B
2012-76-D
1G-001
22-1A
2012-21-A
2012-89-B
72-1A
74-1A
1G-039
2012-44-B
gi|306478698|ref|YP 003868436.1| putative fusion protein Rhododendron virus A
gi|948271133|gb|ALM54938.1| fusion protein RdRp Southern tomato virus
gi|167077480|gb|ABZ10949.1| fusion protein Southern tomato virus
gi|86451112|gb|ABC96787.1| RNA-dependent RNA polymerase-like protein partial Southern tomato virus
gi|213496076|ref|YP 002321509.1| fusion protein p122 Southern tomato virus
gi|923057524|gb|ALB05811.1| fusion protein RdRp Southern tomato virus
2012-99-E
gi|224094849|ref|XP 002310263.1| hypothetical protein POPTR 0007s13310g Populus trichocarpa
1B-077
2-84-C
gi|308097039|gb|ADO14078.1| fusion protein partial Blueberry latent virus
gi|308814348|ref|YP 003934623.1| fusion protein Blueberry latent virus
gi|308097099|gb|ADO14118.1| fusion protein Blueberry latent virus
gi|308096997|gb|ADO14050.1| fusion protein partial Blueberry latent virus
gi|308097000|gb|ADO14052.1| fusion protein partial Blueberry latent virus
gi|308097018|gb|ADO14064.1| fusion protein partial Blueberry latent virus
gi|308097009|gb|ADO14058.1| fusion protein partial Blueberry latent virus
gi|308097093|gb|ADO14114.1| fusion protein partial Blueberry latent virus
gi|308097069|gb|ADO14098.1| fusion protein partial Blueberry latent virus
gi|308097054|gb|ADO14088.1| fusion protein partial Blueberry latent virus
gi|308097006|gb|ADO14056.1| fusion protein partial Blueberry latent virus
gi|308097021|gb|ADO14066.1| fusion protein partial Blueberry latent virus
99
57
96
72
66
21
95
22
88
31
18
73
35
94
23
42
99
0.1

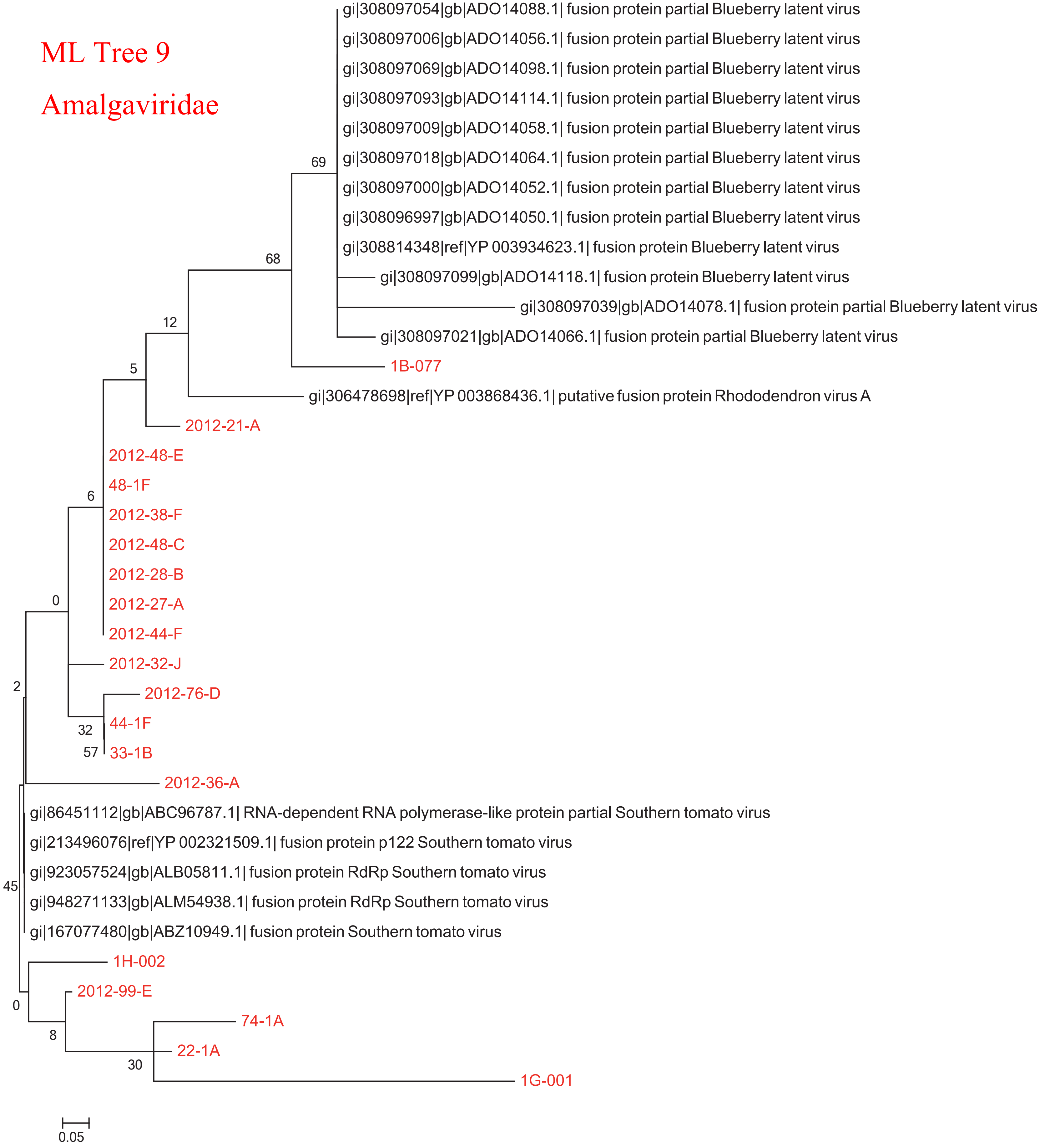

ML Tree 9
Amalgaviridae
gi|308097054|gb|ADO14088.1| fusion protein partial Blueberry latent virus
gi|308097006|gb|ADO14056.1| fusion protein partial Blueberry latent virus
gi|308097069|gb|ADO14098.1| fusion protein partial Blueberry latent virus
gi|308097093|gb|ADO14114.1| fusion protein partial Blueberry latent virus
gi|308097009|gb|ADO14058.1| fusion protein partial Blueberry latent virus
gi|308097018|gb|ADO14064.1| fusion protein partial Blueberry latent virus
gi|308097000|gb|ADO14052.1| fusion protein partial Blueberry latent virus
gi|308096997|gb|ADO14050.1| fusion protein partial Blueberry latent virus
gi|308814348|ref|YP 003934623.1| fusion protein Blueberry latent virus
gi|308097099|gb|ADO14118.1| fusion protein Blueberry latent virus
gi|308097039|gb|ADO14078.1| fusion protein partial Blueberry latent virus
gi|308097021|gb|ADO14066.1| fusion protein partial Blueberry latent virus
1B-077
gi|306478698|ref|YP 003868436.1| putative fusion protein Rhododendron virus A
2012-21-A
2012-48-E
48-1F
2012-38-F
2012-48-C
2012-28-B
2012-27-A
2012-44-F
2012-32-J
2012-76-D
44-1F
33-1B
2012-36-A
gi|86451112|gb|ABC96787.1| RNA-dependent RNA polymerase-like protein partial Southern tomato virus
gi|213496076|ref|YP 002321509.1| fusion protein p122 Southern tomato virus
gi|923057524|gb|ALB05811.1| fusion protein RdRp Southern tomato virus
gi|948271133|gb|ALM54938.1| fusion protein RdRp Southern tomato virus
gi|167077480|gb|ABZ10949.1| fusion protein Southern tomato virus
1H-002
2012-99-E
74-1A
22-1A
1G-001
69
68
12
5
6
0
2
32
57
45
0
8
30
0.05

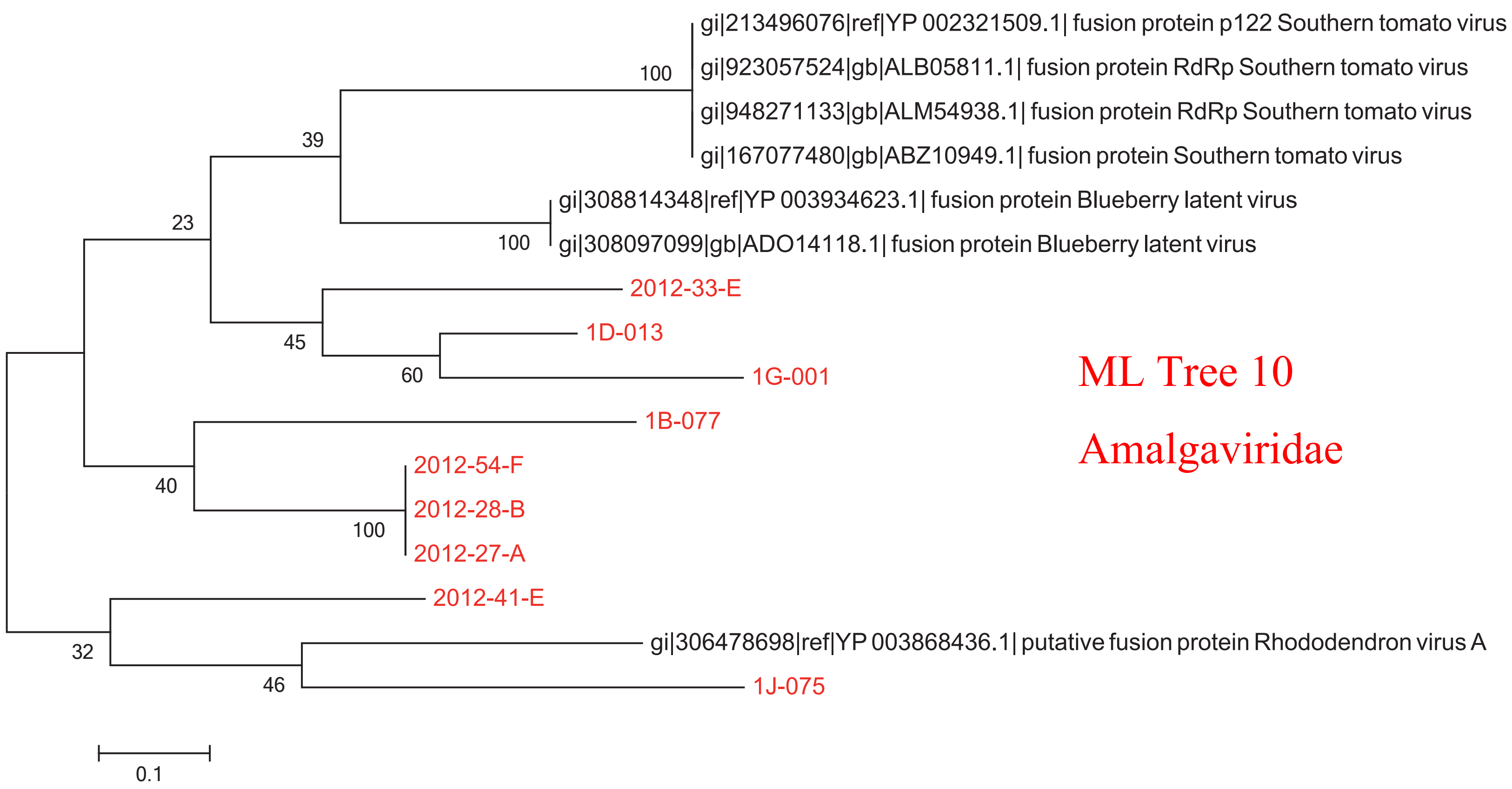
gi|213496076|ref|YP 002321509.1| fusion protein p122 Southern tomato virus
gi|923057524|gb|ALB05811.1| fusion protein RdRp Southern tomato virus
gi|948271133|gb|ALM54938.1| fusion protein RdRp Southern tomato virus
gi|167077480|gb|ABZ10949.1| fusion protein Southern tomato virus
gi|308814348|ref|YP 003934623.1| fusion protein Blueberry latent virus
gi|308097099|gb|ADO14118.1| fusion protein Blueberry latent virus
2012-33-E
1D-013
1G-001
1B-077
2012-54-F
2012-28-B
2012-27-A
2012-41-E
gi|306478698|ref|YP 003868436.1| putative fusion protein Rhododendron virus A
1J-075
100
39
23
100
45
60
40
100
32
46
0.1
ML Tree 10
Amalgaviridae

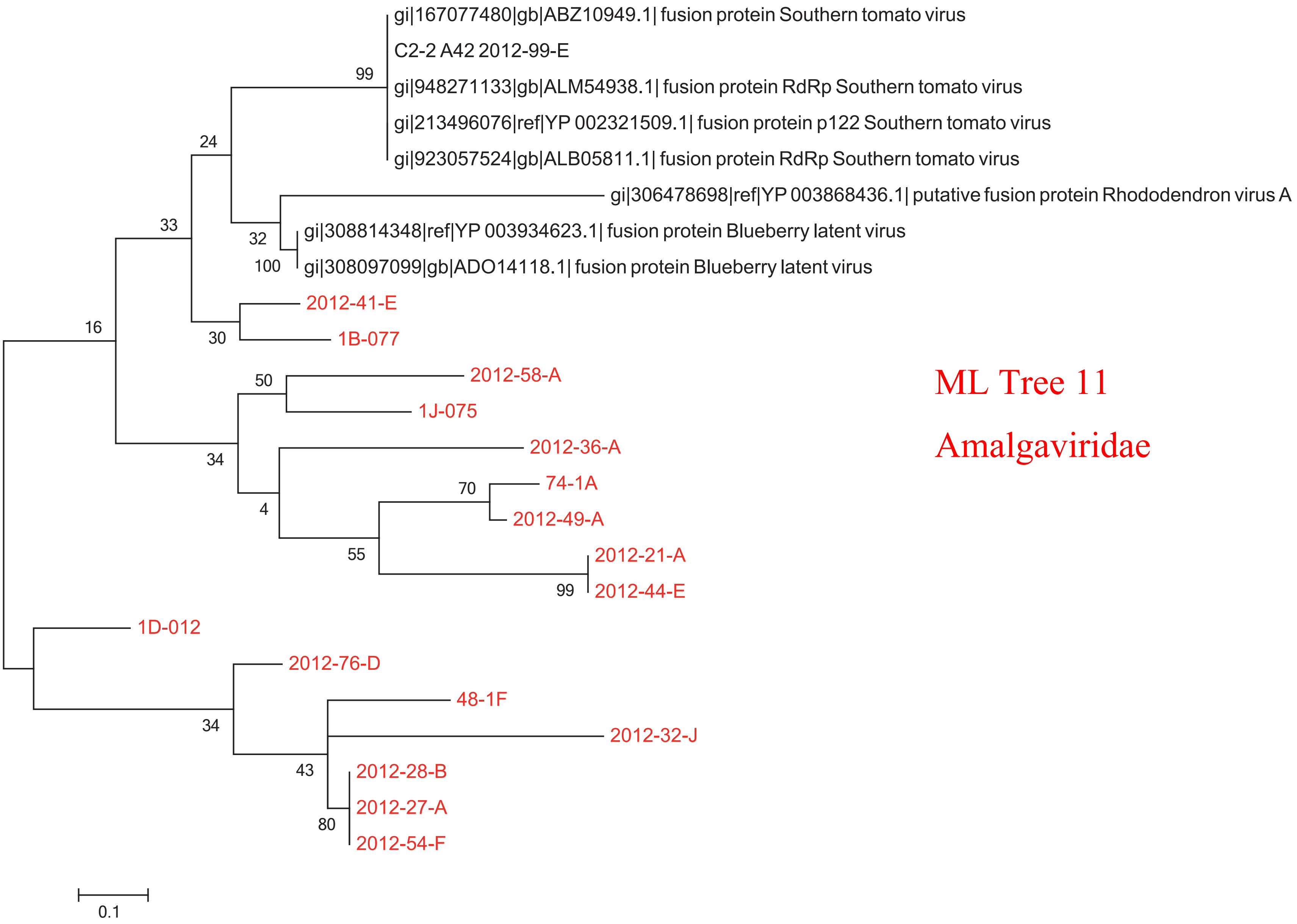
gi|167077480|gb|ABZ10949.1| fusion protein Southern tomato virus
C2-2 A42 2012-99-E
gi|948271133|gb|ALM54938.1| fusion protein RdRp Southern tomato virus
gi|213496076|ref|YP 002321509.1| fusion protein p122 Southern tomato virus
gi|923057524|gb|ALB05811.1| fusion protein RdRp Southern tomato virus
gi|306478698|ref|YP 003868436.1| putative fusion protein Rhododendron virus A
gi|308814348|ref|YP 003934623.1| fusion protein Blueberry latent virus
gi|308097099|gb|ADO14118.1| fusion protein Blueberry latent virus
2012-41-E
1B-077
2012-58-A
1J-075
2012-36-A
74-1A
2012-49-A
2012-21-A
2012-44-E
1D-012
2012-76-D
48-1F
2012-32-J
2012-28-B
2012-27-A
2012-54-F
99
24
33
32
100
16
30
50
34
70
4
55
99
34
43
80
0.1
ML Tree 11
Amalgaviridae

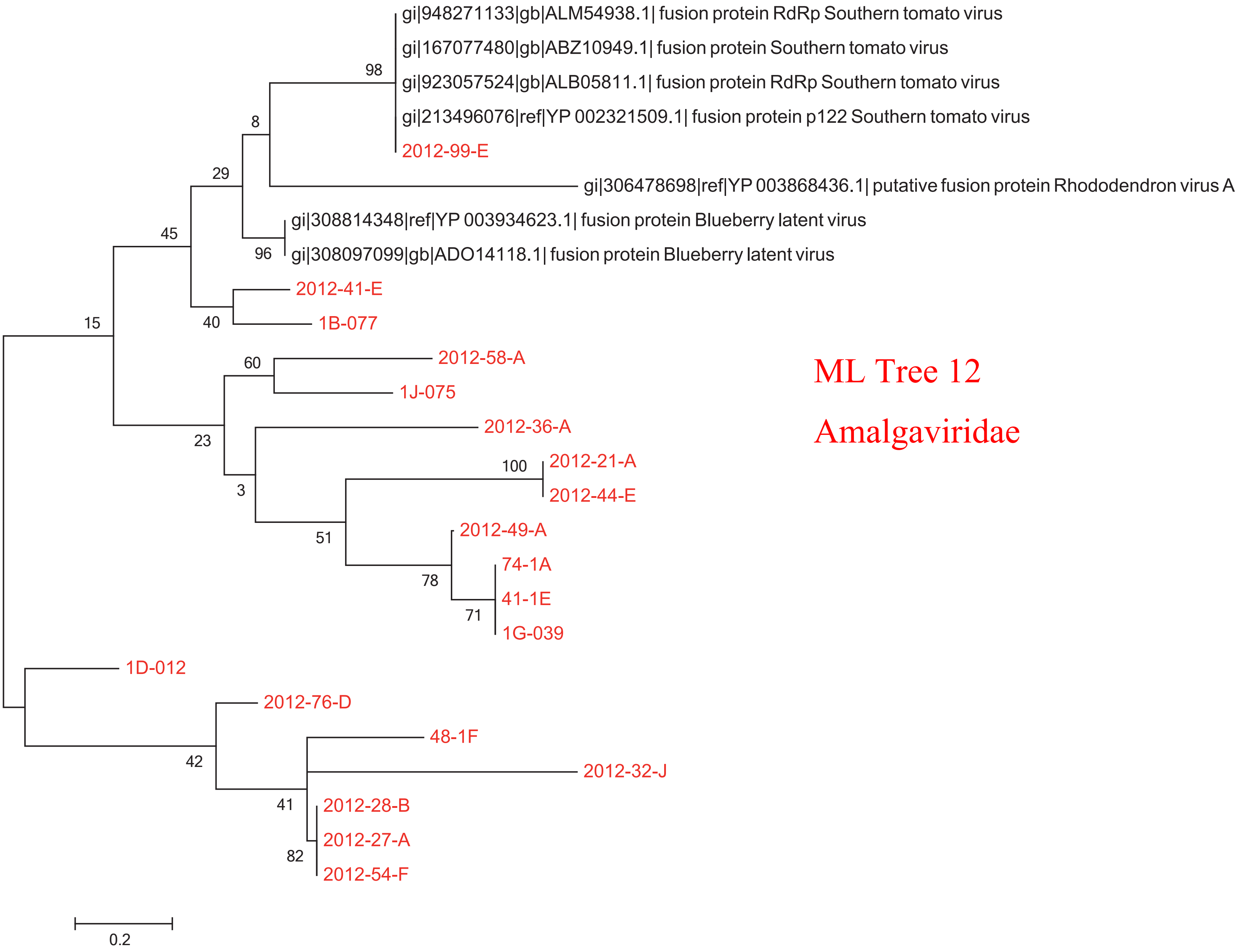

gi|948271133|gb|ALM54938.1| fusion protein RdRp Southern tomato virus
gi|167077480|gb|ABZ10949.1| fusion protein Southern tomato virus
gi|923057524|gb|ALB05811.1| fusion protein RdRp Southern tomato virus
gi|213496076|ref|YP 002321509.1| fusion protein p122 Southern tomato virus
2012-99-E
gi|306478698|ref|YP 003868436.1| putative fusion protein Rhododendron virus A
gi|308814348|ref|YP 003934623.1| fusion protein Blueberry latent virus
gi|308097099|gb|ADO14118.1| fusion protein Blueberry latent virus
2012-41-E
1B-077
2012-58-A
1J-075
2012-36-A
2012-21-A
2012-44-E
2012-49-A
74-1A
41-1E
1G-039
1D-012
2012-76-D
48-1F
2012-32-J
2012-28-B
2012-27-A
2012-54-F
98
8
29
96
45
40
15
60
23
3
100
51
78
71
42
41
82
0.2
ML Tree 12
Amalgaviridae

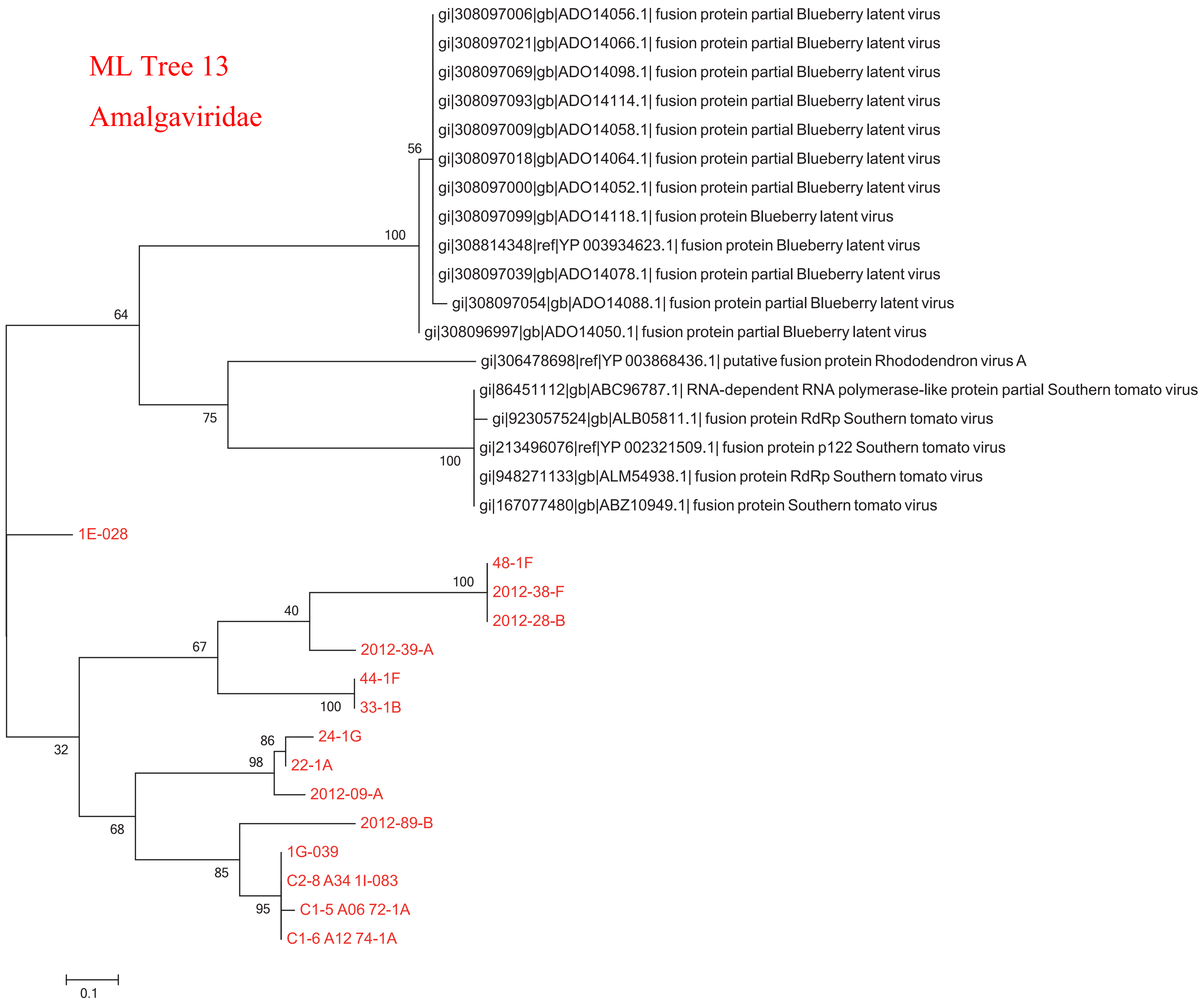
ML Tree 13
Amalgaviridae
gi|308097006|gb|ADO14056.1| fusion protein partial Blueberry latent virus
gi|308097021|gb|ADO14066.1| fusion protein partial Blueberry latent virus
gi|308097069|gb|ADO14098.1| fusion protein partial Blueberry latent virus
gi|308097093|gb|ADO14114.1| fusion protein partial Blueberry latent virus
gi|308097009|gb|ADO14058.1| fusion protein partial Blueberry latent virus
gi|308097018|gb|ADO14064.1| fusion protein partial Blueberry latent virus
gi|308097000|gb|ADO14052.1| fusion protein partial Blueberry latent virus
gi|308097099|gb|ADO14118.1| fusion protein Blueberry latent virus
gi|308814348|ref|YP 003934623.1| fusion protein Blueberry latent virus
gi|308097039|gb|ADO14078.1| fusion protein partial Blueberry latent virus
gi|308097054|gb|ADO14088.1| fusion protein partial Blueberry latent virus
gi|308096997|gb|ADO14050.1| fusion protein partial Blueberry latent virus
gi|306478698|ref|YP 003868436.1| putative fusion protein Rhododendron virus A
gi|86451112|gb|ABC96787.1| RNA-dependent RNA polymerase-like protein partial Southern tomato virus
gi|923057524|gb|ALB05811.1| fusion protein RdRp Southern tomato virus
gi|213496076|ref|YP 002321509.1| fusion protein p122 Southern tomato virus
gi|948271133|gb|ALM54938.1| fusion protein RdRp Southern tomato virus
gi|167077480|gb|ABZ10949.1| fusion protein Southern tomato virus
1E-028
48-1F
2012-38-F
2012-28-B
2012-39-A
44-1F
33-1B
24-1G
22-1A
2012-09-A
2012-89-B
1G-039
C2-8 A34 1I-083
C1-5 A06 72-1A
C1-6 A12 74-1A
56
100
64
75
100
100
40
67
100
32
86
98
68
85
95
0.1

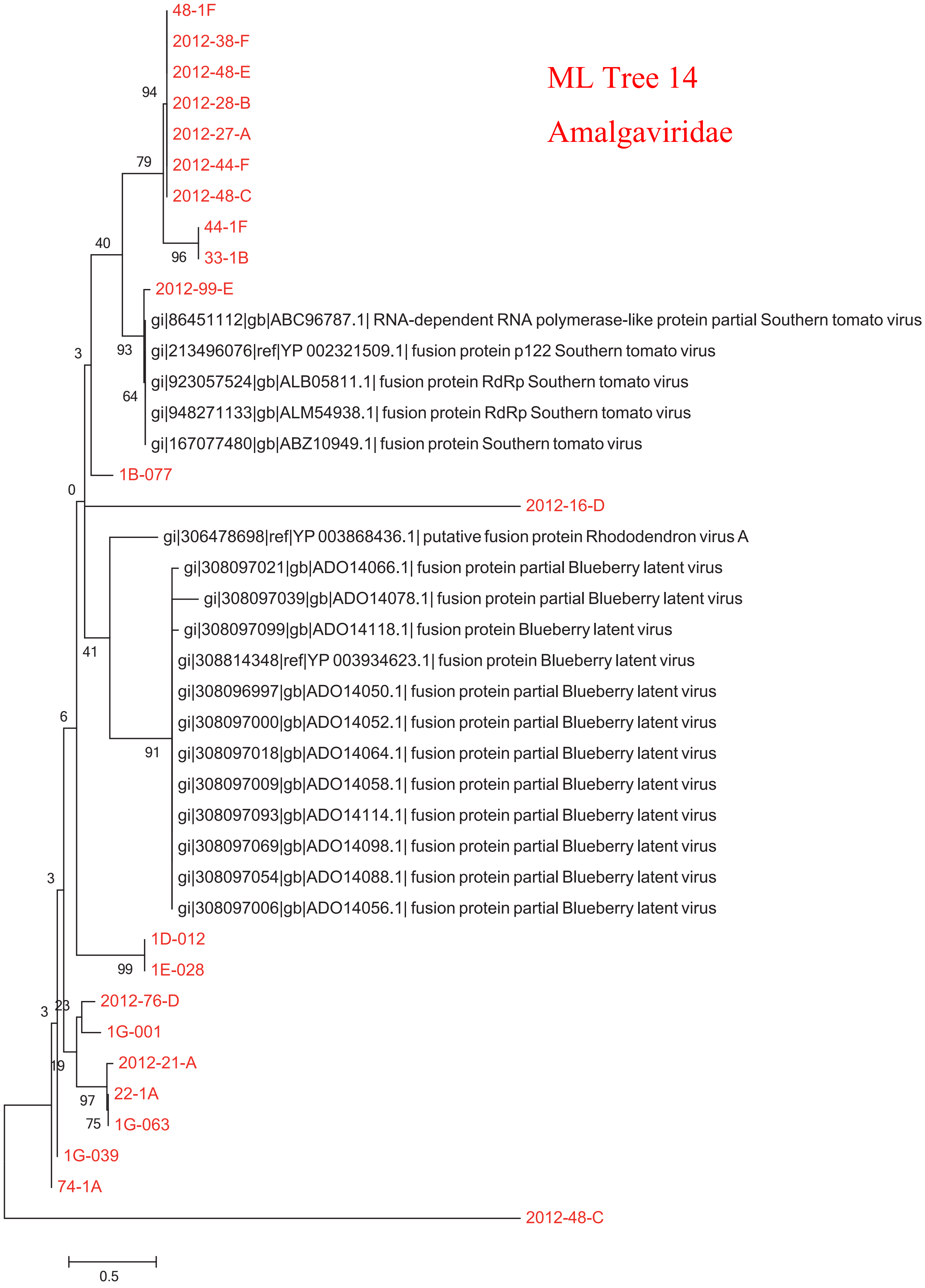
ML Tree 14
Amalgaviridae
48-1F
2012-38-F
2012-48-E
2012-28-B
2012-27-A
2012-44-F
2012-48-C
44-1F
33-1B
2012-99-E
gi|86451112|gb|ABC96787.1| RNA-dependent RNA polymerase-like protein partial Southern tomato virus
gi|213496076|ref|YP 002321509.1| fusion protein p122 Southern tomato virus
gi|923057524|gb|ALB05811.1| fusion protein RdRp Southern tomato virus
gi|948271133|gb|ALM54938.1| fusion protein RdRp Southern tomato virus
gi|167077480|gb|ABZ10949.1| fusion protein Southern tomato virus
1B-077
2012-16-D
gi|306478698|ref|YP 003868436.1| putative fusion protein Rhododendron virus A
gi|308097021|gb|ADO14066.1| fusion protein partial Blueberry latent virus
gi|308097039|gb|ADO14078.1| fusion protein partial Blueberry latent virus
gi|308097099|gb|ADO14118.1| fusion protein Blueberry latent virus
gi|308814348|ref|YP 003934623.1| fusion protein Blueberry latent virus
gi|308096997|gb|ADO14050.1| fusion protein partial Blueberry latent virus
gi|308097000|gb|ADO14052.1| fusion protein partial Blueberry latent virus
gi|308097018|gb|ADO14064.1| fusion protein partial Blueberry latent virus
gi|308097009|gb|ADO14058.1| fusion protein partial Blueberry latent virus
gi|308097093|gb|ADO14114.1| fusion protein partial Blueberry latent virus
gi|308097069|gb|ADO14098.1| fusion protein partial Blueberry latent virus
gi|308097054|gb|ADO14088.1| fusion protein partial Blueberry latent virus
gi|308097006|gb|ADO14056.1| fusion protein partial Blueberry latent virus
1D-012
1E-028
2012-76-D
1G-001
2012-21-A
22-1A
1G-063
1G-039
74-1A
2012-48-C
94
79
96
40
3
93
64
0
41
91
6
3
99
3
23
19
97
75
0.5

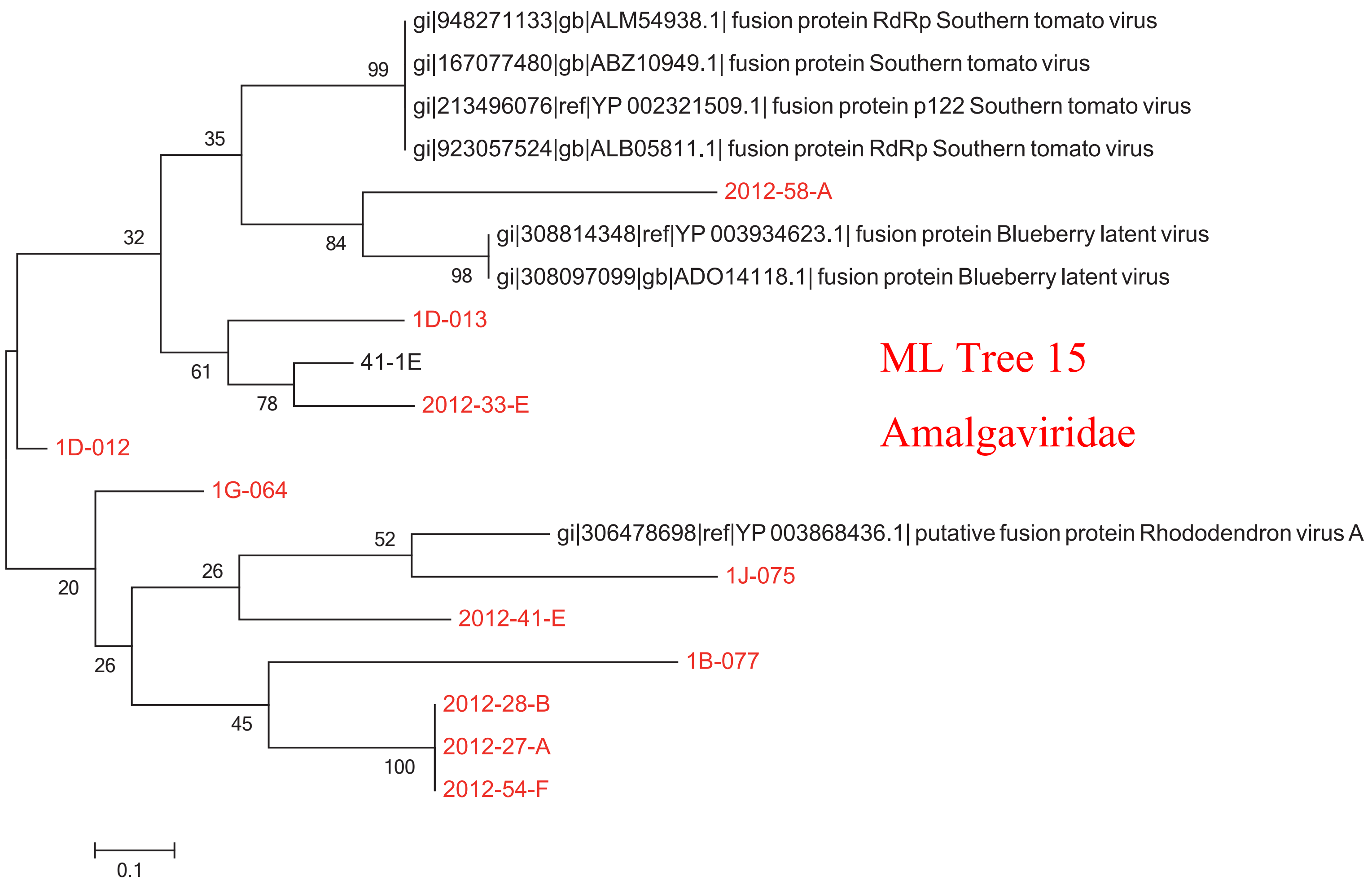
gi|948271133|gb|ALM54938.1| fusion protein RdRp Southern tomato virus
gi|167077480|gb|ABZ10949.1| fusion protein Southern tomato virus
gi|213496076|ref|YP 002321509.1| fusion protein p122 Southern tomato virus
gi|923057524|gb|ALB05811.1| fusion protein RdRp Southern tomato virus
2012-58-A
gi|308814348|ref|YP 003934623.1| fusion protein Blueberry latent virus
gi|308097099|gb|ADO14118.1| fusion protein Blueberry latent virus
1D-013
41-1E
2012-33-E
1D-012
1G-064
gi|306478698|ref|YP 003868436.1| putative fusion protein Rhododendron virus A
1J-075
2012-41-E
1B-077
2012-28-B
2012-27-A
2012-54-F
99
35
84
98
32
61
78
52
26
20
26
45
100
0.1
ML Tree 15
Amalgaviridae

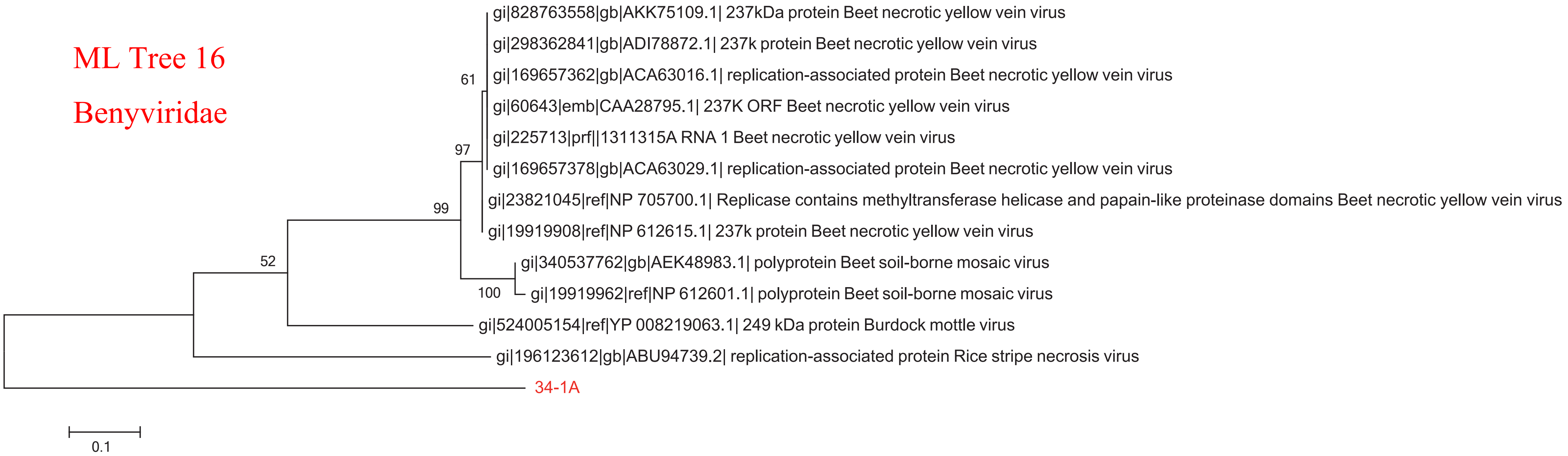
ML Tree 16
Benyviridae
gi|828763558|gb|AKK75109.1| 237kDa protein Beet necrotic yellow vein virus
gi|298362841|gb|ADI78872.1| 237k protein Beet necrotic yellow vein virus
gi|169657362|gb|ACA63016.1| replication-associated protein Beet necrotic yellow vein virus
gi|60643|emb|CAA28795.1| 237K ORF Beet necrotic yellow vein virus
gi|225713|prf||1311315A RNA 1 Beet necrotic yellow vein virus
gi|169657378|gb|ACA63029.1| replication-associated protein Beet necrotic yellow vein virus
gi|23821045|ref|NP 705700.1| Replicase contains methyltransferase helicase and papain-like proteinase domains Beet necrotic yellow vein virus
gi|19919908|ref|NP 612615.1| 237k protein Beet necrotic yellow vein virus
gi|340537762|gb|AEK48983.1| polyprotein Beet soil-borne mosaic virus
gi|19919962|ref|NP 612601.1| polyprotein Beet soil-borne mosaic virus
gi|524005154|ref|YP 008219063.1| 249 kDa protein Burdock mottle virus
gi|196123612|gb|ABU94739.2| replication-associated protein Rice stripe necrosis virus
34-1A
61
97
99
52
100
0.1

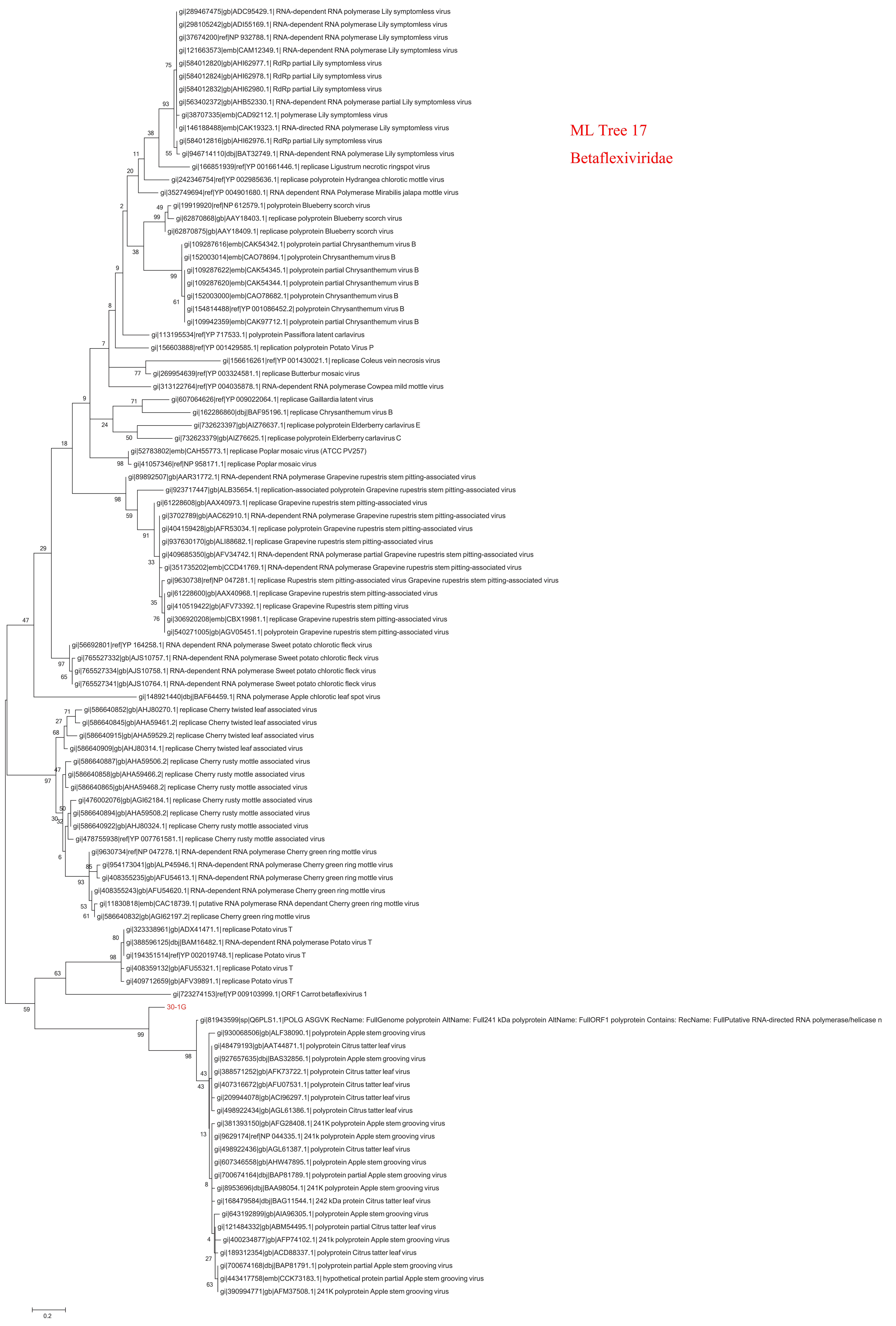
ML Tree 17
Betaflexiviridae
gi|289467475|gb|ADC95429.1| RNA-dependent RNA polymerase Lily symptomless virus
gi|298105242|gb|ADI55169.1| RNA-dependent RNA polymerase Lily symptomless virus
gi|37674200|ref|NP 932788.1| RNA-dependent RNA polymerase Lily symptomless virus
gi|121663573|emb|CAM12349.1| RNA-dependent RNA polymerase Lily symptomless virus
gi|584012820|gb|AHI62977.1| RdRp partial Lily symptomless virus
gi|584012824|gb|AHI62978.1| RdRp partial Lily symptomless virus
gi|584012832|gb|AHI62980.1| RdRp partial Lily symptomless virus
gi|563402372|gb|AHB52330.1| RNA-dependent RNA polymerase partial Lily symptomless virus
gi|38707335|emb|CAD92112.1| polymerase Lily symptomless virus
gi|146188488|emb|CAK19323.1| RNA-directed RNA polymerase Lily symptomless virus
gi|584012816|gb|AHI62976.1| RdRp partial Lily symptomless virus
gi|946714110|dbj|BAT32749.1| RNA-dependent RNA polymerase Lily symptomless virus
gi|166851939|ref|YP 001661446.1| replicase Ligustrum necrotic ringspot virus
gi|242346754|ref|YP 002985636.1| replicase polyprotein Hydrangea chlorotic mottle virus
gi|352749694|ref|YP 004901680.1| RNA dependent RNA Polymerase Mirabilis jalapa mottle virus
gi|19919920|ref|NP 612579.1| polyprotein Blueberry scorch virus
gi|62870868|gb|AAY18403.1| replicase polyprotein Blueberry scorch virus
gi|62870875|gb|AAY18409.1| replicase polyprotein Blueberry scorch virus
gi|109287616|emb|CAK54342.1| polyprotein partial Chrysanthemum virus B
gi|152003014|emb|CAO78694.1| polyprotein Chrysanthemum virus B
gi|109287622|emb|CAK54345.1| polyprotein partial Chrysanthemum virus B
gi|109287620|emb|CAK54344.1| polyprotein partial Chrysanthemum virus B
gi|152003000|emb|CAO78682.1| polyprotein Chrysanthemum virus B
gi|154814488|ref|YP 001086452.2| polyprotein Chrysanthemum virus B
gi|109942359|emb|CAK97712.1| polyprotein partial Chrysanthemum virus B
gi|113195534|ref|YP 717533.1| polyprotein Passiflora latent carlavirus
gi|156603888|ref|YP 001429585.1| replication polyprotein Potato Virus P
gi|156616261|ref|YP 001430021.1| replicase Coleus vein necrosis virus
gi|269954639|ref|YP 003324581.1| replicase Butterbur mosaic virus
gi|313122764|ref|YP 004035878.1| RNA-dependent RNA polymerase Cowpea mild mottle virus
gi|607064626|ref|YP 009022064.1| replicase Gaillardia latent virus
gi|162286860|dbj|BAF95196.1| replicase Chrysanthemum virus B
gi|732623397|gb|AIZ76637.1| replicase polyprotein Elderberry carlavirus E
gi|732623379|gb|AIZ76625.1| replicase polyprotein Elderberry carlavirus C
gi|52783802|emb|CAH55773.1| replicase Poplar mosaic virus (ATCC PV257)
gi|41057346|ref|NP 958171.1| replicase Poplar mosaic virus
gi|89892507|gb|AAR31772.1| RNA-dependent RNA polymerase Grapevine rupestris stem pitting-associated virus
gi|923717447|gb|ALB35654.1| replication-associated polyprotein Grapevine rupestris stem pitting-associated virus
gi|61228608|gb|AAX40973.1| replicase Grapevine rupestris stem pitting-associated virus
gi|3702789|gb|AAC62910.1| RNA-dependent RNA polymerase Grapevine rupestris stem pitting-associated virus
gi|404159428|gb|AFR53034.1| replicase polyprotein Grapevine rupestris stem pitting-associated virus
gi|937630170|gb|ALI88682.1| replicase Grapevine rupestris stem pitting-associated virus
gi|409685350|gb|AFV34742.1| RNA-dependent RNA polymerase partial Grapevine rupestris stem pitting-associated virus
gi|351735202|emb|CCD41769.1| RNA-dependent RNA polymerase Grapevine rupestris stem pitting-associated virus
gi|9630738|ref|NP 047281.1| replicase Rupestris stem pitting-associated virus Grapevine rupestris stem pitting-associated virus
gi|61228600|gb|AAX40968.1| replicase Grapevine rupestris stem pitting-associated virus
gi|410519422|gb|AFV73392.1| replicase Grapevine Rupestris stem pitting virus
gi|306920208|emb|CBX19981.1| replicase Grapevine rupestris stem pitting-associated virus
gi|540271005|gb|AGV05451.1| polyprotein Grapevine rupestris stem pitting-associated virus
gi|56692801|ref|YP 164258.1| RNA dependent RNA polymerase Sweet potato chlorotic fleck virus
gi|765527332|gb|AJS10757.1| RNA-dependent RNA polymerase Sweet potato chlorotic fleck virus
gi|765527334|gb|AJS10758.1| RNA-dependent RNA polymerase Sweet potato chlorotic fleck virus
gi|765527341|gb|AJS10764.1| RNA-dependent RNA polymerase Sweet potato chlorotic fleck virus
gi|148921440|dbj|BAF64459.1| RNA polymerase Apple chlorotic leaf spot virus
gi|586640852|gb|AHJ80270.1| replicase Cherry twisted leaf associated virus
gi|586640845|gb|AHA59461.2| replicase Cherry twisted leaf associated virus
gi|586640915|gb|AHA59529.2| replicase Cherry twisted leaf associated virus
gi|586640909|gb|AHJ80314.1| replicase Cherry twisted leaf associated virus
gi|586640887|gb|AHA59506.2| replicase Cherry rusty mottle associated virus
gi|586640858|gb|AHA59466.2| replicase Cherry rusty mottle associated virus
gi|586640865|gb|AHA59468.2| replicase Cherry rusty mottle associated virus
gi|476002076|gb|AGI62184.1| replicase Cherry rusty mottle associated virus
gi|586640894|gb|AHA59508.2| replicase Cherry rusty mottle associated virus
gi|586640922|gb|AHJ80324.1| replicase Cherry rusty mottle associated virus
gi|478755938|ref|YP 007761581.1| replicase Cherry rusty mottle associated virus
gi|9630734|ref|NP 047278.1| RNA-dependent RNA polymerase Cherry green ring mottle virus
gi|954173041|gb|ALP45946.1| RNA-dependent RNA polymerase Cherry green ring mottle virus
gi|408355235|gb|AFU54613.1| RNA-dependent RNA polymerase Cherry green ring mottle virus
gi|408355243|gb|AFU54620.1| RNA-dependent RNA polymerase Cherry green ring mottle virus
gi|11830818|emb|CAC18739.1| putative RNA polymerase RNA dependant Cherry green ring mottle virus
gi|586640832|gb|AGI62197.2| replicase Cherry green ring mottle virus
gi|323338961|gb|ADX41471.1| replicase Potato virus T
gi|388596125|dbj|BAM16482.1| RNA-dependent RNA polymerase Potato virus T
gi|194351514|ref|YP 002019748.1| replicase Potato virus T
gi|408359132|gb|AFU55321.1| replicase Potato virus T
gi|409712659|gb|AFV39891.1| replicase Potato virus T
gi|723274153|ref|YP 009103999.1| ORF1 Carrot betaflexivirus 1
30-1G
gi|81943599|sp|Q6PLS1.1|POLG ASGVK RecName: FullGenome polyprotein AltName: Full241 kDa polyprotein AltName: FullORF1 polyprotein Contains: RecName: FullPutative RNA-directed RNA polymerase/helicase n
gi|930068506|gb|ALF38090.1| polyprotein Apple stem grooving virus
gi|48479193|gb|AAT44871.1| polyprotein Citrus tatter leaf virus
gi|927657635|dbj|BAS32856.1| polyprotein Apple stem grooving virus
gi|388571252|gb|AFK73722.1| polyprotein Citrus tatter leaf virus
gi|407316672|gb|AFU07531.1| polyprotein Citrus tatter leaf virus
gi|209944078|gb|ACI96297.1| polyprotein Citrus tatter leaf virus
gi|498922434|gb|AGL61386.1| polyprotein Citrus tatter leaf virus
gi|381393150|gb|AFG28408.1| 241K polyprotein Apple stem grooving virus
gi|9629174|ref|NP 044335.1| 241k polyprotein Apple stem grooving virus
gi|498922436|gb|AGL61387.1| polyprotein Citrus tatter leaf virus
gi|607346558|gb|AHW47895.1| polyprotein Apple stem grooving virus
gi|700674164|dbj|BAP81789.1| polyprotein partial Apple stem grooving virus
gi|8953696|dbj|BAA98054.1| 241K polyprotein Apple stem grooving virus
gi|168479584|dbj|BAG11544.1| 242 kDa protein Citrus tatter leaf virus
gi|643192899|gb|AIA96305.1| polyprotein Apple stem grooving virus
gi|121484332|gb|ABM54495.1| polyprotein partial Citrus tatter leaf virus
gi|400234877|gb|AFP74102.1| 241k polyprotein Apple stem grooving virus
gi|189312354|gb|ACD88337.1| polyprotein Citrus tatter leaf virus
gi|700674168|dbj|BAP81791.1| polyprotein partial Apple stem grooving virus
gi|443417758|emb|CCK73183.1| hypothetical protein partial Apple stem grooving virus
gi|390994771|gb|AFM37508.1| 241K polyprotein Apple stem grooving virus
0.2

ML Tree 18
Betaflexiviridae

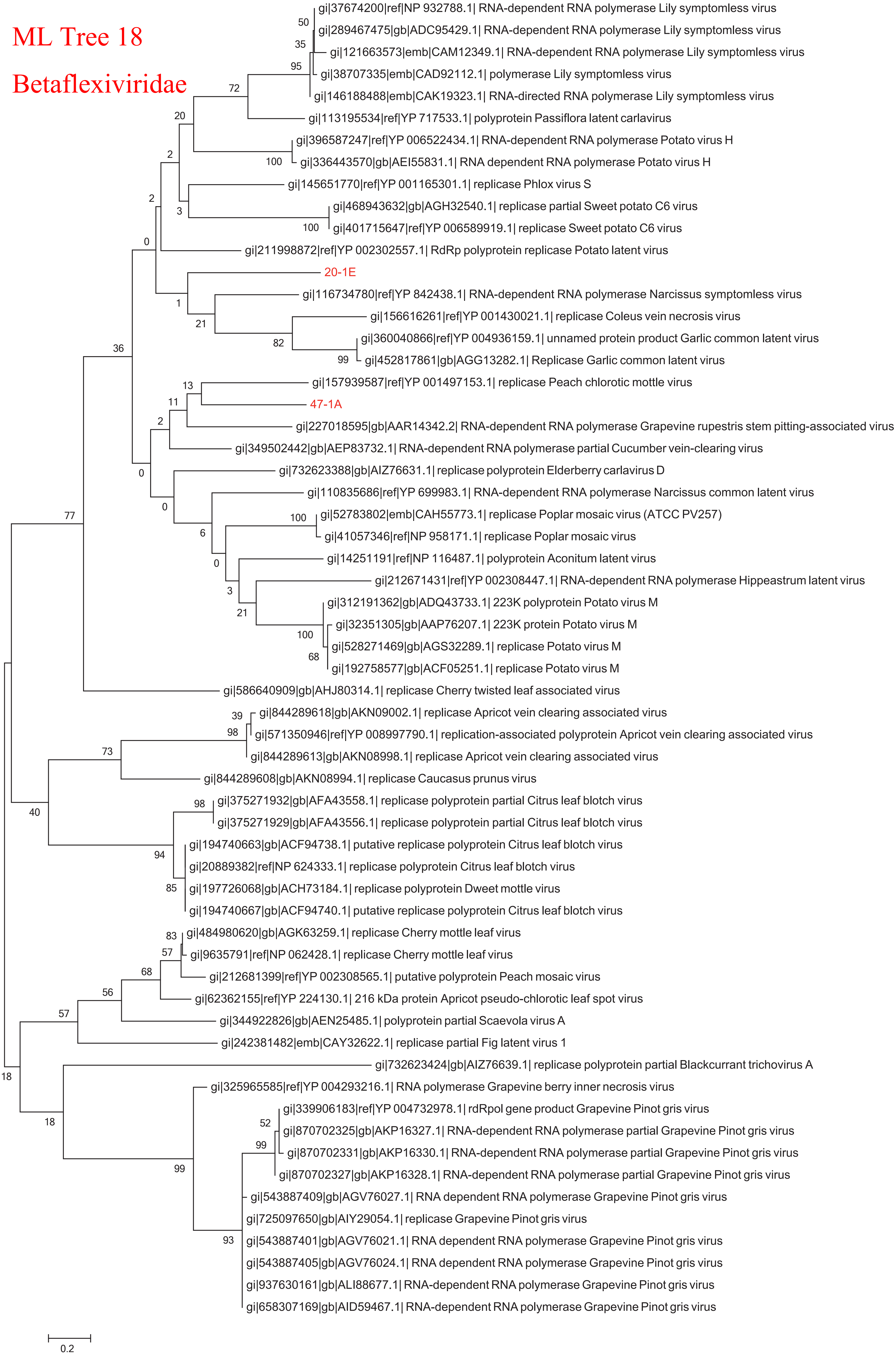

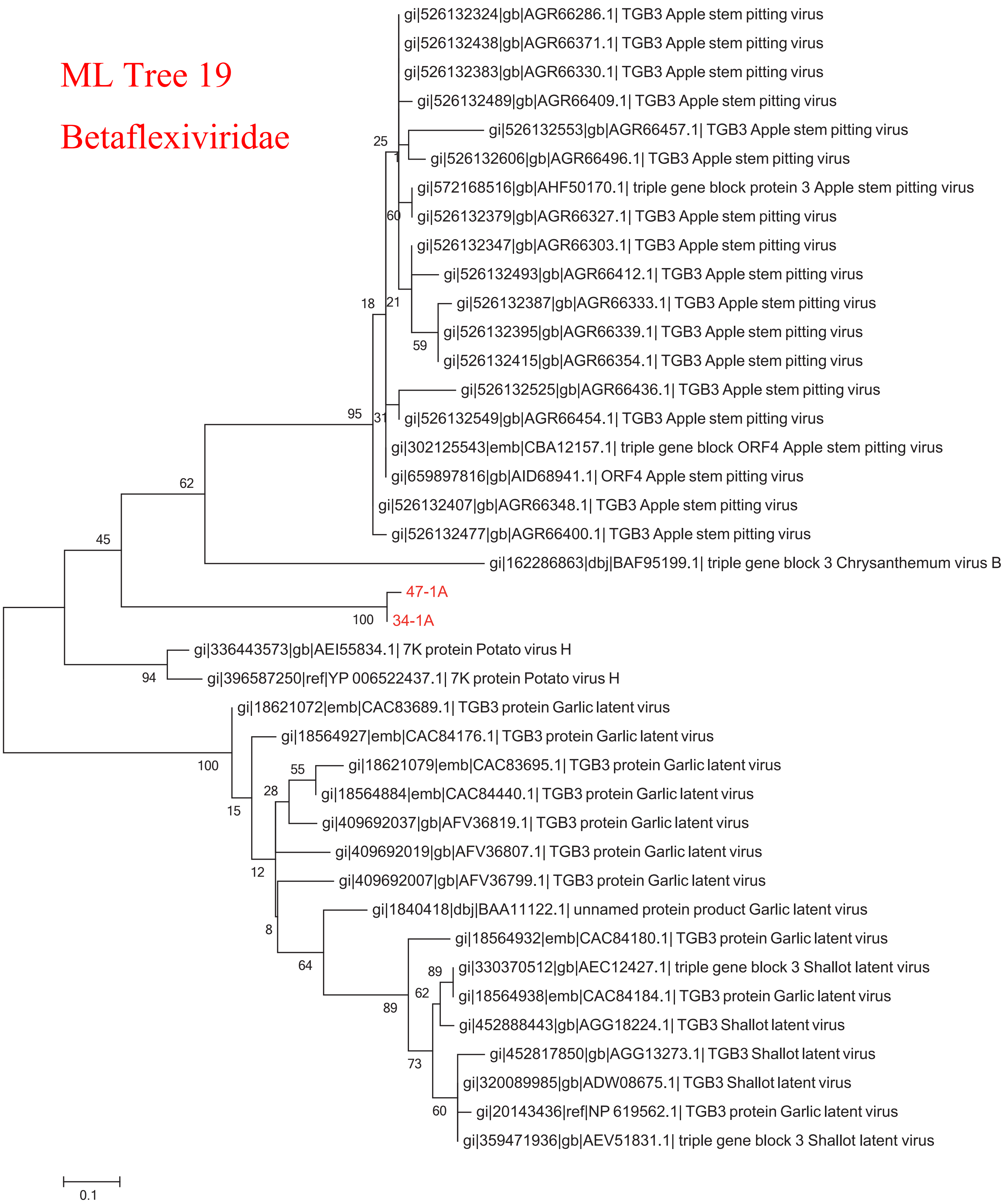

ML Tree 19
Betaflexiviridae
gi|526132324|gb|AGR66286.1| TGB3 Apple stem pitting virus
gi|526132438|gb|AGR66371.1| TGB3 Apple stem pitting virus
gi|526132383|gb|AGR66330.1| TGB3 Apple stem pitting virus
gi|526132489|gb|AGR66409.1| TGB3 Apple stem pitting virus
gi|526132553|gb|AGR66457.1| TGB3 Apple stem pitting virus
gi|526132606|gb|AGR66496.1| TGB3 Apple stem pitting virus
gi|572168516|gb|AHF50170.1| triple gene block protein 3 Apple stem pitting virus
gi|526132379|gb|AGR66327.1| TGB3 Apple stem pitting virus
gi|526132347|gb|AGR66303.1| TGB3 Apple stem pitting virus
gi|526132493|gb|AGR66412.1| TGB3 Apple stem pitting virus
gi|526132387|gb|AGR66333.1| TGB3 Apple stem pitting virus
gi|526132395|gb|AGR66339.1| TGB3 Apple stem pitting virus
gi|526132415|gb|AGR66354.1| TGB3 Apple stem pitting virus
gi|526132525|gb|AGR66436.1| TGB3 Apple stem pitting virus
gi|526132549|gb|AGR66454.1| TGB3 Apple stem pitting virus
gi|302125543|emb|CBA12157.1| triple gene block ORF4 Apple stem pitting virus
gi|659897816|gb|AID68941.1| ORF4 Apple stem pitting virus
gi|526132407|gb|AGR66348.1| TGB3 Apple stem pitting virus
gi|526132477|gb|AGR66400.1| TGB3 Apple stem pitting virus
gi|162286863|dbj|BAF95199.1| triple gene block 3 Chrysanthemum virus B
47-1A
34-1A
gi|336443573|gb|AEI55834.1| 7K protein Potato virus H
gi|396587250|ref|YP 006522437.1| 7K protein Potato virus H
gi|18621072|emb|CAC83689.1| TGB3 protein Garlic latent virus
gi|18564927|emb|CAC84176.1| TGB3 protein Garlic latent virus
gi|18621079|emb|CAC83695.1| TGB3 protein Garlic latent virus
gi|18564884|emb|CAC84440.1| TGB3 protein Garlic latent virus
gi|409692037|gb|AFV36819.1| TGB3 protein Garlic latent virus
gi|409692019|gb|AFV36807.1| TGB3 protein Garlic latent virus
gi|409692007|gb|AFV36799.1| TGB3 protein Garlic latent virus
gi|1840418|dbj|BAA11122.1| unnamed protein product Garlic latent virus
gi|18564932|emb|CAC84180.1| TGB3 protein Garlic latent virus
gi|330370512|gb|AEC12427.1| triple gene block 3 Shallot latent virus
gi|18564938|emb|CAC84184.1| TGB3 protein Garlic latent virus
gi|452888443|gb|AGG18224.1| TGB3 Shallot latent virus
gi|452817850|gb|AGG13273.1| TGB3 Shallot latent virus
gi|320089985|gb|ADW08675.1| TGB3 Shallot latent virus
gi|20143436|ref|NP 619562.1| TGB3 protein Garlic latent virus
gi|359471936|gb|AEV51831.1| triple gene block 3 Shallot latent virus
25
1
60
18
21
59
95
31
62
45
100
94
100
55
28
15
12
8
64
89
89
62
73
60
0.1

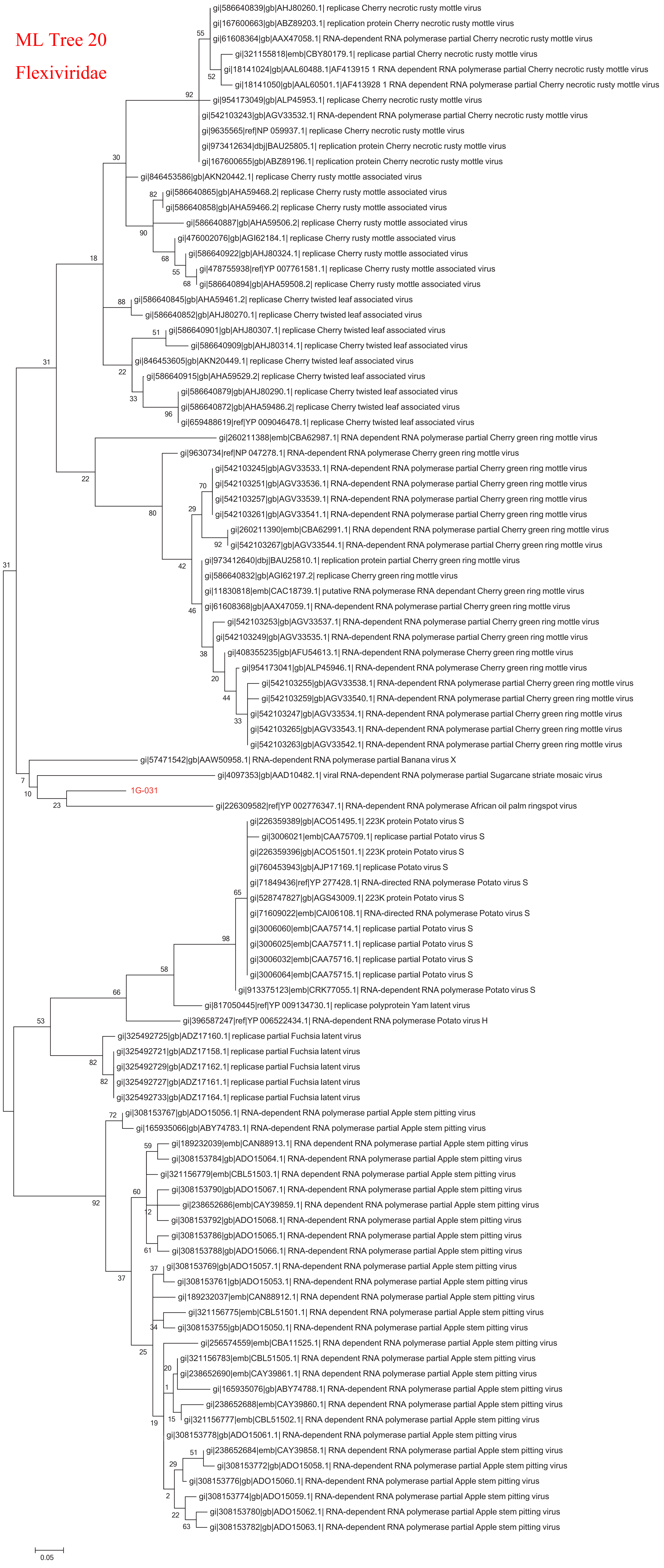
ML Tree 20
Flexiviridae
gi|586640839|gb|AHJ80260.1| replicase Cherry necrotic rusty mottle virus
gi|167600663|gb|ABZ89203.1| replication protein Cherry necrotic rusty mottle virus
gi|61608364|gb|AAX47058.1| RNA-dependent RNA polymerase partial Cherry necrotic rusty mottle virus
gi|321155818|emb|CBY80179.1| replicase partial Cherry necrotic rusty mottle virus
gi|18141024|gb|AAL60488.1|AF413915 1 RNA dependent RNA polymerase partial Cherry necrotic rusty mottle virus
gi|18141050|gb|AAL60501.1|AF413928 1 RNA dependent RNA polymerase partial Cherry necrotic rusty mottle virus
gi|954173049|gb|ALP45953.1| replicase Cherry necrotic rusty mottle virus
gi|542103243|gb|AGV33532.1| RNA-dependent RNA polymerase partial Cherry necrotic rusty mottle virus
gi|9635565|ref|NP 059937.1| replicase Cherry necrotic rusty mottle virus
gi|973412634|dbj|BAU25805.1| replication protein Cherry necrotic rusty mottle virus
gi|167600655|gb|ABZ89196.1| replication protein Cherry necrotic rusty mottle virus
gi|846453586|gb|AKN20442.1| replicase Cherry rusty mottle associated virus
gi|586640865|gb|AHA59468.2| replicase Cherry rusty mottle associated virus
gi|586640858|gb|AHA59466.2| replicase Cherry rusty mottle associated virus
gi|586640887|gb|AHA59506.2| replicase Cherry rusty mottle associated virus
gi|476002076|gb|AGI62184.1| replicase Cherry rusty mottle associated virus
gi|586640922|gb|AHJ80324.1| replicase Cherry rusty mottle associated virus
gi|478755938|ref|YP 007761581.1| replicase Cherry rusty mottle associated virus
gi|586640894|gb|AHA59508.2| replicase Cherry rusty mottle associated virus
gi|586640845|gb|AHA59461.2| replicase Cherry twisted leaf associated virus
gi|586640852|gb|AHJ80270.1| replicase Cherry twisted leaf associated virus
gi|586640901|gb|AHJ80307.1| replicase Cherry twisted leaf associated virus
gi|586640909|gb|AHJ80314.1| replicase Cherry twisted leaf associated virus
gi|846453605|gb|AKN20449.1| replicase Cherry twisted leaf associated virus
gi|586640915|gb|AHA59529.2| replicase Cherry twisted leaf associated virus
gi|586640879|gb|AHJ80290.1| replicase Cherry twisted leaf associated virus
gi|586640872|gb|AHA59486.2| replicase Cherry twisted leaf associated virus
gi|659488619|ref|YP 009046478.1| replicase Cherry twisted leaf associated virus
gi|260211388|emb|CBA62987.1| RNA dependent RNA polymerase partial Cherry green ring mottle virus
gi|9630734|ref|NP 047278.1| RNA-dependent RNA polymerase Cherry green ring mottle virus
gi|542103245|gb|AGV33533.1| RNA-dependent RNA polymerase partial Cherry green ring mottle virus
gi|542103251|gb|AGV33536.1| RNA-dependent RNA polymerase partial Cherry green ring mottle virus
gi|542103257|gb|AGV33539.1| RNA-dependent RNA polymerase partial Cherry green ring mottle virus
gi|542103261|gb|AGV33541.1| RNA-dependent RNA polymerase partial Cherry green ring mottle virus
gi|260211390|emb|CBA62991.1| RNA dependent RNA polymerase partial Cherry green ring mottle virus
gi|542103267|gb|AGV33544.1| RNA-dependent RNA polymerase partial Cherry green ring mottle virus
gi|973412640|dbj|BAU25810.1| replication protein partial Cherry green ring mottle virus
gi|586640832|gb|AGI62197.2| replicase Cherry green ring mottle virus
gi|11830818|emb|CAC18739.1| putative RNA polymerase RNA dependant Cherry green ring mottle virus
gi|61608368|gb|AAX47059.1| RNA-dependent RNA polymerase partial Cherry green ring mottle virus
gi|542103253|gb|AGV33537.1| RNA-dependent RNA polymerase partial Cherry green ring mottle virus
gi|542103249|gb|AGV33535.1| RNA-dependent RNA polymerase partial Cherry green ring mottle virus
gi|408355235|gb|AFU54613.1| RNA-dependent RNA polymerase Cherry green ring mottle virus
gi|954173041|gb|ALP45946.1| RNA-dependent RNA polymerase Cherry green ring mottle virus
gi|542103255|gb|AGV33538.1| RNA-dependent RNA polymerase partial Cherry green ring mottle virus
gi|542103259|gb|AGV33540.1| RNA-dependent RNA polymerase partial Cherry green ring mottle virus
gi|542103247|gb|AGV33534.1| RNA-dependent RNA polymerase partial Cherry green ring mottle virus
gi|542103265|gb|AGV33543.1| RNA-dependent RNA polymerase partial Cherry green ring mottle virus
gi|542103263|gb|AGV33542.1| RNA-dependent RNA polymerase partial Cherry green ring mottle virus
gi|57471542|gb|AAW50958.1| RNA-dependent RNA polymerase partial Banana virus X
gi|4097353|gb|AAD10482.1| viral RNA-dependent RNA polymerase partial Sugarcane striate mosaic virus
1G-031
gi|226309582|ref|YP 002776347.1| RNA-dependent RNA polymerase African oil palm ringspot virus
gi|226359389|gb|ACO51495.1| 223K protein Potato virus S
gi|3006021|emb|CAA75709.1| replicase partial Potato virus S
gi|226359396|gb|ACO51501.1| 223K protein Potato virus S
gi|760453943|gb|AJP17169.1| replicase Potato virus S
gi|71849436|ref|YP 277428.1| RNA-directed RNA polymerase Potato virus S
gi|528747827|gb|AGS43009.1| 223K protein Potato virus S
gi|71609022|emb|CAI06108.1| RNA-directed RNA polymerase Potato virus S
gi|3006060|emb|CAA75714.1| replicase partial Potato virus S
gi|3006025|emb|CAA75711.1| replicase partial Potato virus S
gi|3006032|emb|CAA75716.1| replicase partial Potato virus S
gi|3006064|emb|CAA75715.1| replicase partial Potato virus S
gi|913375123|emb|CRK77055.1| RNA-dependent RNA polymerase Potato virus S
gi|817050445|ref|YP 009134730.1| replicase polyprotein Yam latent virus
gi|396587247|ref|YP 006522434.1| RNA-dependent RNA polymerase Potato virus H
gi|325492725|gb|ADZ17160.1| replicase partial Fuchsia latent virus
gi|325492721|gb|ADZ17158.1| replicase partial Fuchsia latent virus
gi|325492729|gb|ADZ17162.1| replicase partial Fuchsia latent virus
gi|325492727|gb|ADZ17161.1| replicase partial Fuchsia latent virus
gi|325492733|gb|ADZ17164.1| replicase partial Fuchsia latent virus
gi|308153767|gb|ADO15056.1| RNA-dependent RNA polymerase partial Apple stem pitting virus
gi|165935066|gb|ABY74783.1| RNA-dependent RNA polymerase partial Apple stem pitting virus
gi|189232039|emb|CAN88913.1| RNA dependent RNA polymerase partial Apple stem pitting virus
gi|308153784|gb|ADO15064.1| RNA-dependent RNA polymerase partial Apple stem pitting virus
gi|321156779|emb|CBL51503.1| RNA dependent RNA polymerase partial Apple stem pitting virus
gi|308153790|gb|ADO15067.1| RNA-dependent RNA polymerase partial Apple stem pitting virus
gi|238652686|emb|CAY39859.1| RNA dependent RNA polymerase partial Apple stem pitting virus
gi|308153792|gb|ADO15068.1| RNA-dependent RNA polymerase partial Apple stem pitting virus
gi|308153786|gb|ADO15065.1| RNA-dependent RNA polymerase partial Apple stem pitting virus
gi|308153788|gb|ADO15066.1| RNA-dependent RNA polymerase partial Apple stem pitting virus
gi|308153769|gb|ADO15057.1| RNA-dependent RNA polymerase partial Apple stem pitting virus
gi|308153761|gb|ADO15053.1| RNA-dependent RNA polymerase partial Apple stem pitting virus
gi|189232037|emb|CAN88912.1| RNA dependent RNA polymerase partial Apple stem pitting virus
gi|321156775|emb|CBL51501.1| RNA dependent RNA polymerase partial Apple stem pitting virus
gi|308153755|gb|ADO15050.1| RNA-dependent RNA polymerase partial Apple stem pitting virus
gi|256574559|emb|CBA11525.1| RNA dependent RNA polymerase partial Apple stem pitting virus
gi|321156783|emb|CBL51505.1| RNA dependent RNA polymerase partial Apple stem pitting virus
gi|238652690|emb|CAY39861.1| RNA dependent RNA polymerase partial Apple stem pitting virus
gi|165935076|gb|ABY74788.1| RNA-dependent RNA polymerase partial Apple stem pitting virus
gi|238652688|emb|CAY39860.1| RNA dependent RNA polymerase partial Apple stem pitting virus
gi|321156777|emb|CBL51502.1| RNA dependent RNA polymerase partial Apple stem pitting virus
gi|308153778|gb|ADO15061.1| RNA-dependent RNA polymerase partial Apple stem pitting virus
gi|238652684|emb|CAY39858.1| RNA dependent RNA polymerase partial Apple stem pitting virus
gi|308153772|gb|ADO15058.1| RNA-dependent RNA polymerase partial Apple stem pitting virus
gi|308153776|gb|ADO15060.1| RNA-dependent RNA polymerase partial Apple stem pitting virus
gi|308153774|gb|ADO15059.1| RNA-dependent RNA polymerase partial Apple stem pitting virus
gi|308153780|gb|ADO15062.1| RNA-dependent RNA polymerase partial Apple stem pitting virus
gi|308153782|gb|ADO15063.1| RNA-dependent RNA polymerase partial Apple stem pitting virus
0.05

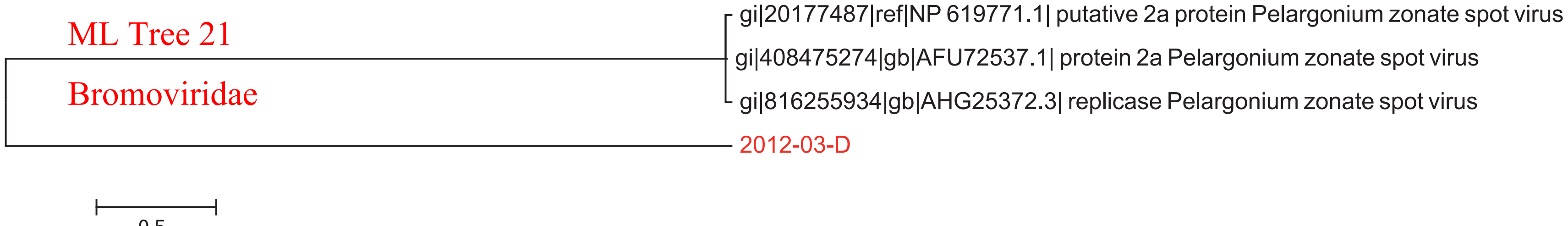
ML Tree 21
Bromoviridae
gi|20177487|ref|NP 619771.1| putative 2a protein Pelargonium zonate spot virus
gi|408475274|gb|AFU72537.1| protein 2a Pelargonium zonate spot virus
gi|816255934|gb|AHG25372.3| replicase Pelargonium zonate spot virus
2012-03-D
0.5

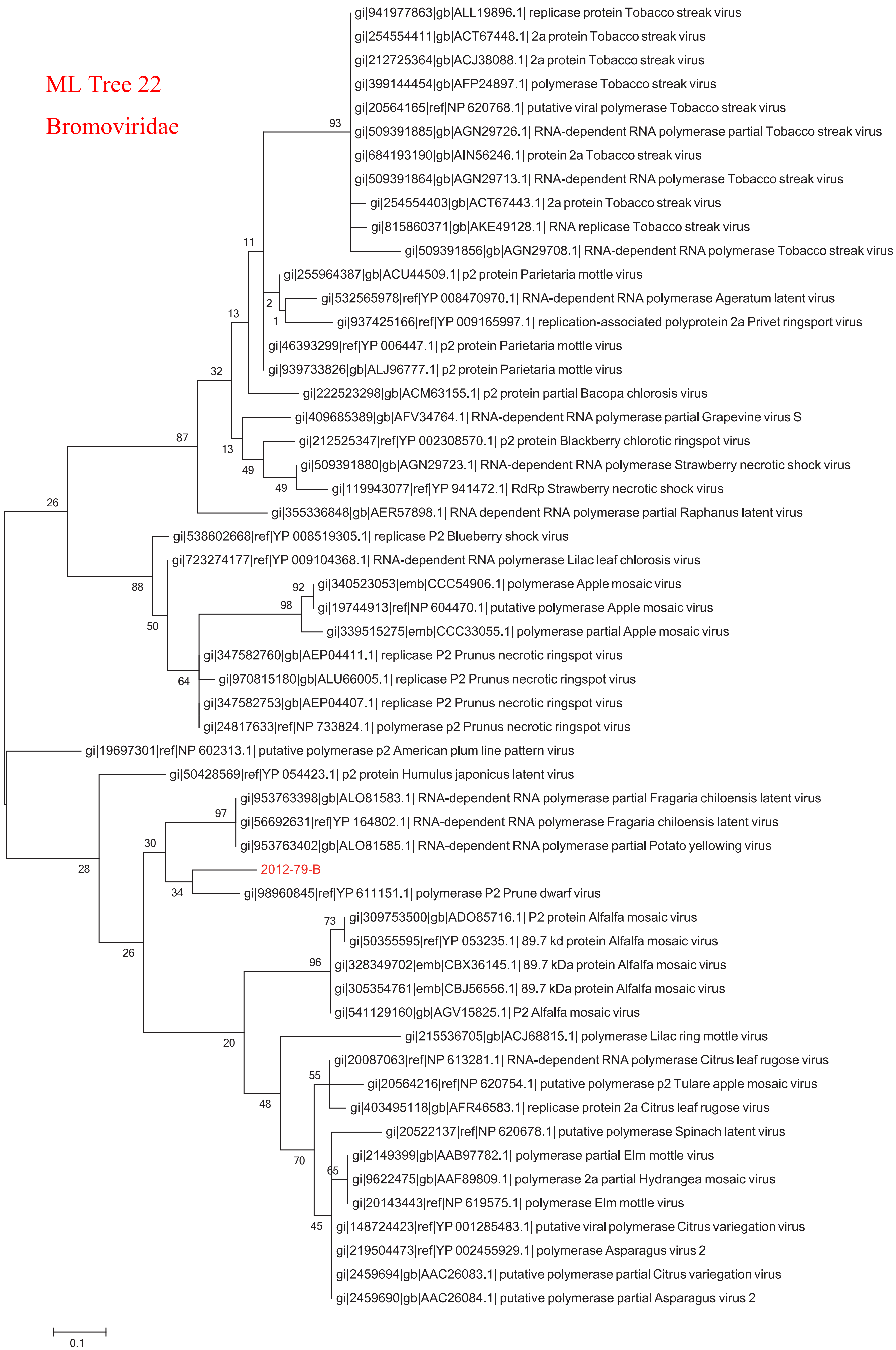
ML Tree 22
Bromoviridae
gi|941977863|gb|ALL19896.1| replicase protein Tobacco streak virus
gi|254554411|gb|ACT67448.1| 2a protein Tobacco streak virus
gi|212725364|gb|ACJ38088.1| 2a protein Tobacco streak virus
gi|399144454|gb|AFP24897.1| polymerase Tobacco streak virus
gi|20564165|ref|NP 620768.1| putative viral polymerase Tobacco streak virus
gi|509391885|gb|AGN29726.1| RNA-dependent RNA polymerase partial Tobacco streak virus
gi|684193190|gb|AIN56246.1| protein 2a Tobacco streak virus
gi|509391864|gb|AGN29713.1| RNA-dependent RNA polymerase Tobacco streak virus
gi|254554403|gb|ACT67443.1| 2a protein Tobacco streak virus
gi|815860371|gb|AKE49128.1| RNA replicase Tobacco streak virus
gi|509391856|gb|AGN29708.1| RNA-dependent RNA polymerase Tobacco streak virus
gi|255964387|gb|ACU44509.1| p2 protein Parietaria mottle virus
gi|532565978|ref|YP 008470970.1| RNA-dependent RNA polymerase Ageratum latent virus
gi|937425166|ref|YP 009165997.1| replication-associated polyprotein 2a Privet ringspot virus
gi|46393299|ref|YP 006447.1| p2 protein Parietaria mottle virus
gi|939733826|gb|ALJ96777.1| p2 protein Parietaria mottle virus
gi|222523298|gb|ACM63155.1| p2 protein partial Bacopa chlorosis virus
gi|409685389|gb|AFV34764.1| RNA-dependent RNA polymerase partial Grapevine virus S
gi|212525347|ref|YP 002308570.1| p2 protein Blackberry chlorotic ringspot virus
gi|509391880|gb|AGN29723.1| RNA-dependent RNA polymerase Strawberry necrotic shock virus
gi|119943077|ref|YP 941472.1| RdRp Strawberry necrotic shock virus
gi|355336848|gb|AER57898.1| RNA dependent RNA polymerase partial Raphanus latent virus
gi|538602668|ref|YP 008519305.1| replicase P2 Blueberry shock virus
gi|723274177|ref|YP 009104368.1| RNA-dependent RNA polymerase Lilac leaf chlorosis virus
gi|340523053|emb|CCC54906.1| polymerase Apple mosaic virus
gi|19744913|ref|NP 604470.1| putative polymerase Apple mosaic virus
gi|339515275|emb|CCC33055.1| polymerase partial Apple mosaic virus
gi|347582760|gb|AEP04411.1| replicase P2 Prunus necrotic ringspot virus
gi|970815180|gb|ALU66005.1| replicase P2 Prunus necrotic ringspot virus
gi|347582753|gb|AEP04407.1| replicase P2 Prunus necrotic ringspot virus
gi|24817633|ref|NP 733824.1| polymerase p2 Prunus necrotic ringspot virus
gi|19697301|ref|NP 602313.1| putative polymerase p2 American plum line pattern virus
gi|50428569|ref|YP 054423.1| p2 protein Humulus japonicus latent virus
gi|953763398|gb|ALO81583.1| RNA-dependent RNA polymerase partial Fragaria chiloensis latent virus
gi|56692631|ref|YP 164802.1| RNA-dependent RNA polymerase Fragaria chiloensis latent virus
gi|953763402|gb|ALO81585.1| RNA-dependent RNA polymerase partial Potato yellowing virus
2012-79-B
gi|98960845|ref|YP 611151.1| polymerase P2 Prune dwarf virus
gi|309753500|gb|ADO85716.1| P2 protein Alfalfa mosaic virus
gi|50355595|ref|YP 053235.1| 89.7 kd protein Alfalfa mosaic virus
gi|328349702|emb|CBX36145.1| 89.7 kDa protein Alfalfa mosaic virus
gi|305354761|emb|CBJ56556.1| 89.7 kDa protein Alfalfa mosaic virus
gi|541129160|gb|AGV15825.1| P2 Alfalfa mosaic virus
gi|215536705|gb|ACJ68815.1| polymerase Lilac ring mottle virus
gi|20087063|ref|NP 613281.1| RNA-dependent RNA polymerase Citrus leaf rugose virus
gi|20564216|ref|NP 620754.1| putative polymerase p2 Tulare apple mosaic virus
gi|403495118|gb|AFR46583.1| replicase protein 2a Citrus leaf rugose virus
gi|20522137|ref|NP 620678.1| putative polymerase Spinach latent virus
gi|2149399|gb|AAB97782.1| polymerase partial Elm mottle virus
gi|9622475|gb|AAF89809.1| polymerase 2a partial Hydrangea mosaic virus
gi|20143443|ref|NP 619575.1| polymerase Elm mottle virus
gi|148724423|ref|YP 001285483.1| putative viral polymerase Citrus variegation virus
gi|219504473|ref|YP 002455929.1| polymerase Asparagus virus 2
gi|2459694|gb|AAC26083.1| putative polymerase partial Citrus variegation virus
gi|2459690|gb|AAC26084.1| putative polymerase partial Asparagus virus 2
93
11
2
1
13
32
87
13
49
49
26
88
50
92
98
64
97
30
34
28
26
73
96
20
55
48
70
65
45
0.1

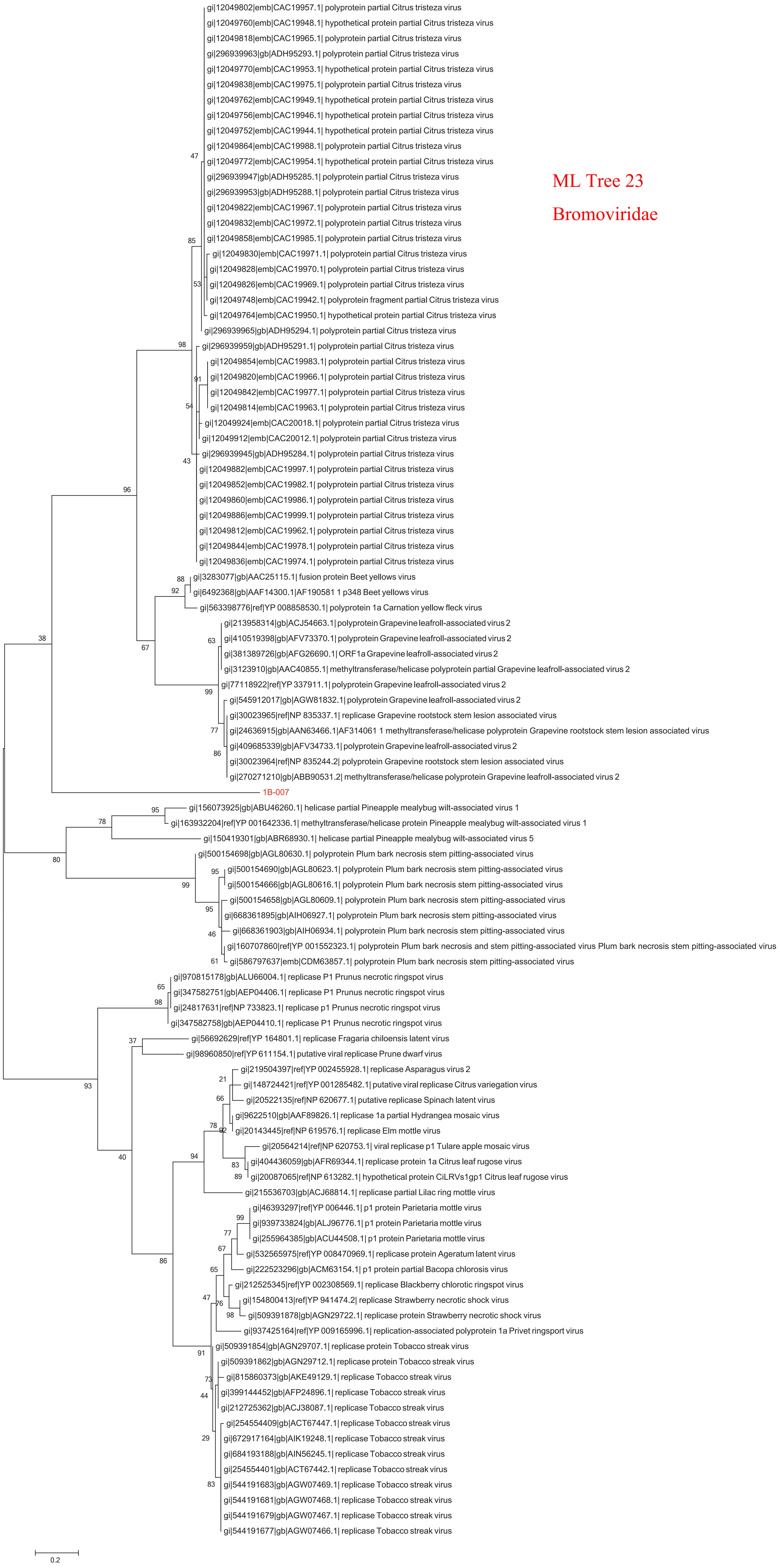

ML Tree 23
Bromoviridae
1B-007
0.2

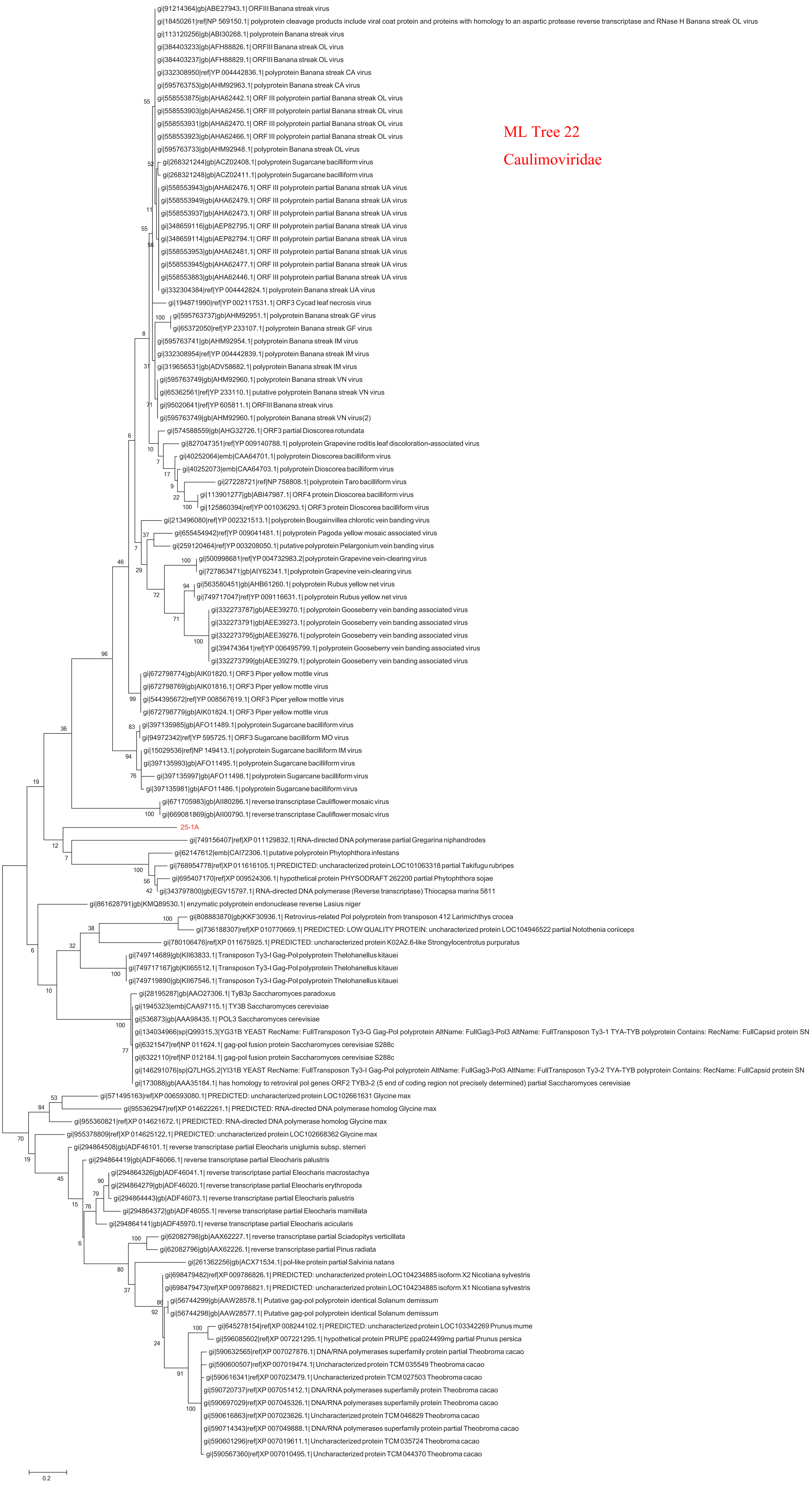
ML Tree 22
Caulimoviridae
gi|91214364|gb|ABE27943.1| ORFIII Banana streak virus
gi|18450261|ref|NP 569150.1| polyprotein cleavage products include viral coat protein and proteins with homology to an aspartic protease reverse transcriptase and RNase H Banana streak OL virus
gi|113120256|gb|ABI30268.1| polyprotein Banana streak virus
gi|384403233|gb|AFH88826.1| ORFIII Banana streak OL virus
gi|384403237|gb|AFH88829.1| ORFIII Banana streak OL virus
gi|332308950|ref|YP 004442836.1| polyprotein Banana streak CA virus
gi|595763753|gb|AHM92963.1| polyprotein Banana streak CA virus
gi|558553875|gb|AHA62442.1| ORF III polyprotein partial Banana streak OL virus
gi|558553903|gb|AHA62456.1| ORF III polyprotein partial Banana streak OL virus
gi|558553931|gb|AHA62470.1| ORF III polyprotein partial Banana streak OL virus
gi|558553923|gb|AHA62466.1| ORF III polyprotein partial Banana streak OL virus
gi|595763733|gb|AHM92948.1| polyprotein Banana streak OL virus
gi|268321244|gb|ACZ02408.1| polyprotein Sugarcane bacilliform virus
gi|268321248|gb|ACZ02411.1| polyprotein Sugarcane bacilliform virus
gi|558553943|gb|AHA62476.1| ORF III polyprotein partial Banana streak UA virus
gi|558553949|gb|AHA62479.1| ORF III polyprotein partial Banana streak UA virus
gi|558553937|gb|AHA62473.1| ORF III polyprotein partial Banana streak UA virus
gi|348659116|gb|AEP82795.1| ORF III polyprotein partial Banana streak UA virus
gi|348659114|gb|AEP82794.1| ORF III polyprotein partial Banana streak UA virus
gi|558553953|gb|AHA62481.1| ORF III polyprotein partial Banana streak UA virus
gi|558553945|gb|AHA62477.1| ORF III polyprotein partial Banana streak UA virus
gi|558553883|gb|AHA62446.1| ORF III polyprotein partial Banana streak UA virus
gi|332304384|ref|YP 004442824.1| polyprotein Banana streak UA virus
gi|194871990|ref|YP 002117531.1| ORF3 Cycad leaf necrosis virus
gi|595763737|gb|AHM92951.1| polyprotein Banana streak GF virus
gi|65372050|ref|YP 233107.1| polyprotein Banana streak GF virus
gi|595763741|gb|AHM92954.1| polyprotein Banana streak IM virus
gi|332308954|ref|YP 004442839.1| polyprotein Banana streak IM virus
gi|319656531|gb|ADV58682.1| polyprotein Banana streak IM virus
gi|595763749|gb|AHM92960.1| polyprotein Banana streak VN virus
gi|65362561|ref|YP 233110.1| putative polyprotein Banana streak VN virus
gi|95020641|ref|YP 605811.1| ORFIII Banana streak virus
gi|595763749|gb|AHM92960.1| polyprotein Banana streak VN virus(2)
gi|574588559|gb|AHG32726.1| ORF3 partial Dioscorea rotundata
gi|827047351|ref|YP 009140788.1| polyprotein Grapevine roditis leaf discoloration-associated virus
gi|40252064|emb|CAA64701.1| polyprotein Dioscorea bacilliform virus
gi|40252073|emb|CAA64703.1| polyprotein Dioscorea bacilliform virus
gi|27228721|ref|NP 758808.1| polyprotein Taro bacilliform virus
gi|113901277|gb|ABI47987.1| ORF4 protein Dioscorea bacilliform virus
gi|125860394|ref|YP 001036293.1| ORF3 protein Dioscorea bacilliform virus
gi|213496080|ref|YP 002321513.1| polyprotein Bougainvillea chlorotic vein banding virus
gi|655454942|ref|YP 009041481.1| polyprotein Pagoda yellow mosaic associated virus
gi|259120464|ref|YP 003208050.1| putative polyprotein Pelargonium vein banding virus
gi|500998681|ref|YP 004732983.2| polyprotein Grapevine vein-clearing virus
gi|727863471|gb|AIY62341.1| polyprotein Grapevine vein-clearing virus
gi|563580451|gb|AHB61260.1| polyprotein Rubus yellow net virus
gi|749717047|ref|YP 009116631.1| polyprotein Rubus yellow net virus
gi|332273787|gb|AEE39270.1| polyprotein Gooseberry vein banding associated virus
gi|332273791|gb|AEE39273.1| polyprotein Gooseberry vein banding associated virus
gi|332273795|gb|AEE39276.1| polyprotein Gooseberry vein banding associated virus
gi|394743641|ref|YP 006495799.1| polyprotein Gooseberry vein banding associated virus
gi|332273799|gb|AEE39279.1| polyprotein Gooseberry vein banding associated virus
gi|672798774|gb|AIK01820.1| ORF3 Piper yellow mottle virus
gi|672798769|gb|AIK01816.1| ORF3 Piper yellow mottle virus
gi|544395672|ref|YP 008567619.1| ORF3 Piper yellow mottle virus
gi|672798779|gb|AIK01824.1| ORF3 Piper yellow mottle virus
gi|397135985|gb|AFO11489.1| polyprotein Sugarcane bacilliform virus
gi|94972342|ref|YP 595725.1| ORF3 Sugarcane bacilliform MO virus
gi|15029536|ref|NP 149413.1| polyprotein Sugarcane bacilliform IM virus
gi|397135993|gb|AFO11495.1| polyprotein Sugarcane bacilliform virus
gi|397135997|gb|AFO11498.1| polyprotein Sugarcane bacilliform virus
gi|397135981|gb|AFO11486.1| polyprotein Sugarcane bacilliform virus
gi|671705983|gb|AII80286.1| reverse transcriptase Cauliflower mosaic virus
gi|669081869|gb|AII00790.1| reverse transcriptase Cauliflower mosaic virus
25-1A
gi|749156407|ref|XP 011129832.1| RNA-directed DNA polymerase partial Gregarina niphandrodes
gi|62147612|emb|CAI72306.1| putative polyprotein Phytophthora infestans
gi|768954778|ref|XP 011616105.1| PREDICTED: uncharacterized protein LOC101063318 partial Takifugu rubripes
gi|695407170|ref|XP 009524306.1| hypothetical protein PHYSODRAFT 262200 partial Phytophthora sojae
gi|343797800|gb|EGV15797.1| RNA-directed DNA polymerase (Reverse transcriptase) Thiocapsa marina 5811
gi|861628791|gb|KMQ89530.1| enzymatic polyprotein endonuclease reverse Lasius niger
gi|808883870|gb|KKF30936.1| Retrovirus-related Pol polyprotein from transposon 412 Larimichthys crocea
gi|736188307|ref|XP 010770669.1| PREDICTED: LOW QUALITY PROTEIN: uncharacterized protein LOC104946522 partial Notothenia coriiceps
gi|780106476|ref|XP 011675925.1| PREDICTED: uncharacterized protein K02A2.6-like Strongylocentrotus purpuratus
gi|749714689|gb|KII63833.1| Transposon Ty3-I Gag-Pol polyprotein Thelohanellus kitauei
gi|749717167|gb|KII65512.1| Transposon Ty3-I Gag-Pol polyprotein Thelohanellus kitauei
gi|749719890|gb|KII67546.1| Transposon Ty3-I Gag-Pol polyprotein Thelohanellus kitauei
gi|28195287|gb|AAO27306.1| TyB3p Saccharomyces paradoxus
gi|1945323|emb|CAA97115.1| TY3B Saccharomyces cerevisiae
gi|536873|gb|AAA98435.1| POL3 Saccharomyces cerevisiae
gi|134034966|sp|Q99315.3|YG31B YEAST RecName: FullTransposon Ty3-G Gag-Pol polyprotein AltName: FullGag3-Pol3 AltName: FullTransposon Ty3-1 TYA-TYB polyprotein Contains: RecName: FullCapsid protein SN
gi|6321547|ref|NP 011624.1| gag-pol fusion protein Saccharomyces cerevisiae S288c
gi|6322110|ref|NP 012184.1| gag-pol fusion protein Saccharomyces cerevisiae S288c
gi|146291076|sp|Q7LHG5.2|YI31B YEAST RecName: FullTransposon Ty3-I Gag-Pol polyprotein AltName: FullGag3-Pol3 AltName: FullTransposon Ty3-2 TYA-TYB polyprotein Contains: RecName: FullCapsid protein SN
gi|173088|gb|AAA35184.1| has homology to retroviral pol genes ORF2 TYB3-2 (5 end of coding region not precisely determined) partial Saccharomyces cerevisiae
gi|571495163|ref|XP 006593080.1| PREDICTED: uncharacterized protein LOC102661631 Glycine max
gi|955362947|ref|XP 014622261.1| PREDICTED: RNA-directed DNA polymerase homolog Glycine max
gi|955360821|ref|XP 014621672.1| PREDICTED: RNA-directed DNA polymerase homolog Glycine max
gi|955378809|ref|XP 014625122.1| PREDICTED: uncharacterized protein LOC102668362 Glycine max
gi|294864508|gb|ADF46101.1| reverse transcriptase partial Eleocharis uniglumis subsp. sterneri
gi|294864419|gb|ADF46066.1| reverse transcriptase partial Eleocharis palustris
gi|294864326|gb|ADF46041.1| reverse transcriptase partial Eleocharis macrostachya
gi|294864279|gb|ADF46020.1| reverse transcriptase partial Eleocharis erythropoda
gi|294864443|gb|ADF46073.1| reverse transcriptase partial Eleocharis palustris
gi|294864372|gb|ADF46055.1| reverse transcriptase partial Eleocharis mamillata
gi|294864141|gb|ADF45970.1| reverse transcriptase partial Eleocharis acicularis
gi|62082798|gb|AAX62227.1| reverse transcriptase partial Sciadopitys verticillata
gi|62082796|gb|AAX62226.1| reverse transcriptase partial Pinus radiata
gi|261362256|gb|ACX71534.1| pol-like protein partial Salvinia natans
gi|698479482|ref|XP 009786826.1| PREDICTED: uncharacterized protein LOC104234885 isoform X2 Nicotiana sylvestris
gi|698479473|ref|XP 009786821.1| PREDICTED: uncharacterized protein LOC104234885 isoform X1 Nicotiana sylvestris
gi|56744299|gb|AAW28578.1| Putative gag-pol polyprotein identical Solanum demissum
gi|56744298|gb|AAW28577.1| Putative gag-pol polyprotein identical Solanum demissum
gi|645278154|ref|XP 008244102.1| PREDICTED: uncharacterized protein LOC103342269 Prunus mume
gi|596085602|ref|XP 007221295.1| hypothetical protein PRUPE ppa024499mg partial Prunus persica
gi|590632565|ref|XP 007027876.1| DNA/RNA polymerases superfamily protein partial Theobroma cacao
gi|590600507|ref|XP 007019474.1| Uncharacterized protein TCM 035549 Theobroma cacao
gi|590616341|ref|XP 007023479.1| Uncharacterized protein TCM 027503 Theobroma cacao
gi|590720737|ref|XP 007051412.1| DNA/RNA polymerases superfamily protein Theobroma cacao
gi|590697029|ref|XP 007045326.1| DNA/RNA polymerases superfamily protein Theobroma cacao
gi|590616863|ref|XP 007023626.1| Uncharacterized protein TCM 046829 Theobroma cacao
gi|590714343|ref|XP 007049888.1| DNA/RNA polymerases superfamily protein partial Theobroma cacao
gi|590601296|ref|XP 007019611.1| Uncharacterized protein TCM 035724 Theobroma cacao
gi|590567360|ref|XP 007010495.1| Uncharacterized protein TCM 044370 Theobroma cacao
0.2

ML Tree 25

Caulimoviridae

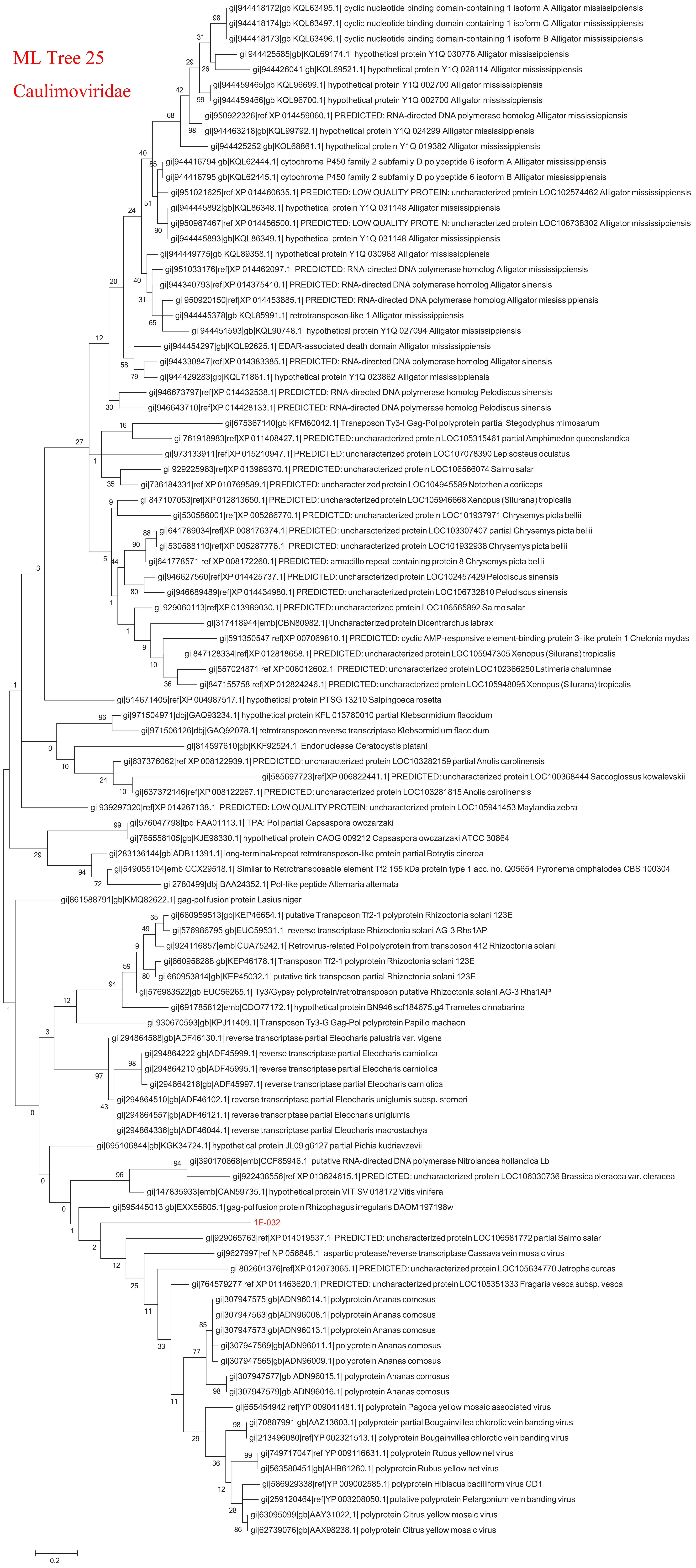

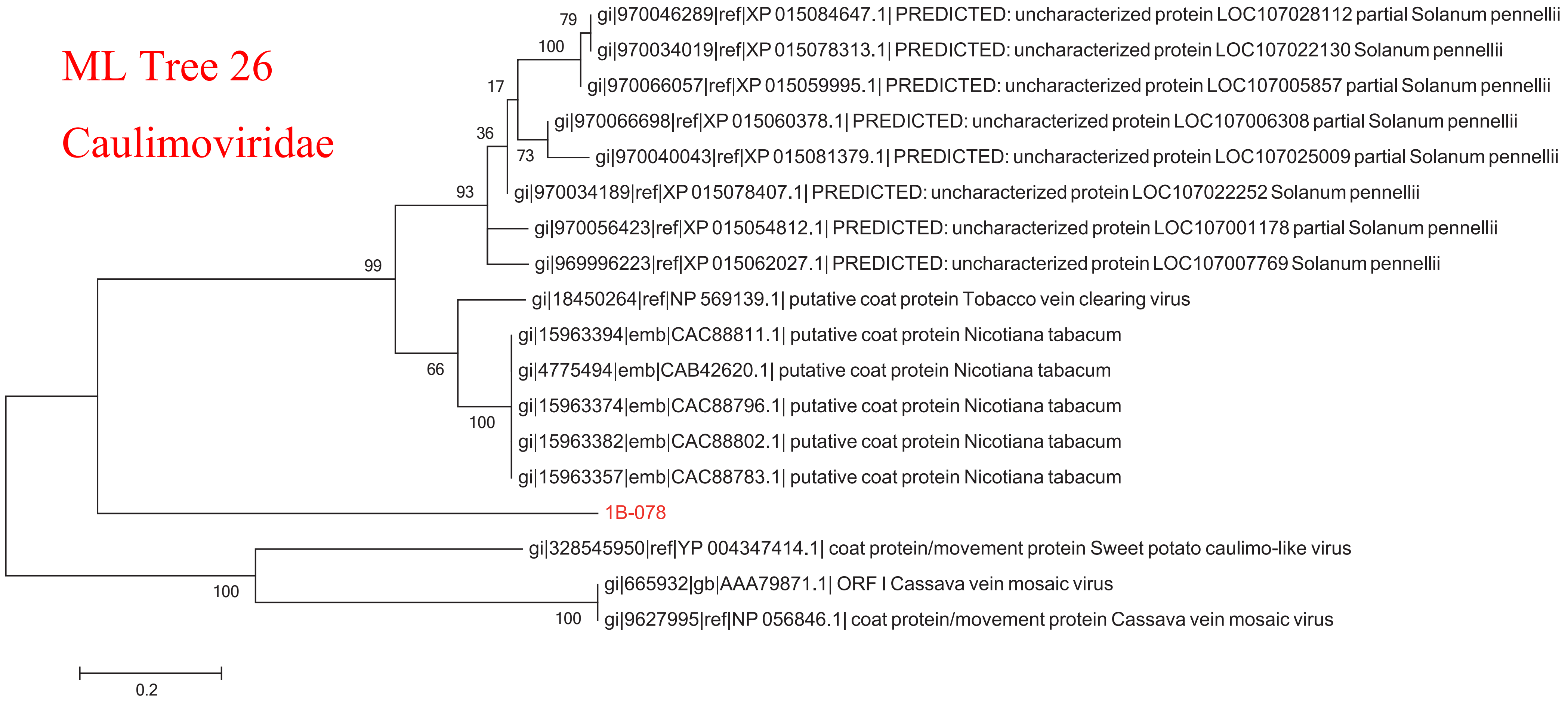
ML Tree 26
Caulimoviridae
gi|970046289|ref|XP 015084647.1| PREDICTED: uncharacterized protein LOC107028112 partial Solanum pennellii
gi|970034019|ref|XP 015078313.1| PREDICTED: uncharacterized protein LOC107022130 Solanum pennellii
gi|970066057|ref|XP 015059995.1| PREDICTED: uncharacterized protein LOC107005857 partial Solanum pennellii
gi|970066698|ref|XP 015060378.1| PREDICTED: uncharacterized protein LOC107006308 partial Solanum pennellii
gi|970040043|ref|XP 015081379.1| PREDICTED: uncharacterized protein LOC107025009 partial Solanum pennellii
gi|970034189|ref|XP 015078407.1| PREDICTED: uncharacterized protein LOC107022252 Solanum pennellii
gi|970056423|ref|XP 015054812.1| PREDICTED: uncharacterized protein LOC107001178 partial Solanum pennellii
gi|969996223|ref|XP 015062027.1| PREDICTED: uncharacterized protein LOC107007769 Solanum pennellii
gi|18450264|ref|NP 569139.1| putative coat protein Tobacco vein clearing virus
gi|15963394|emb|CAC88811.1| putative coat protein Nicotiana tabacum
gi|4775494|emb|CAB42620.1| putative coat protein Nicotiana tabacum
gi|15963374|emb|CAC88796.1| putative coat protein Nicotiana tabacum
gi|15963382|emb|CAC88802.1| putative coat protein Nicotiana tabacum
gi|15963357|emb|CAC88783.1| putative coat protein Nicotiana tabacum
1B-078
gi|328545950|ref|YP 004347414.1| coat protein/movement protein Sweet potato caulimo-like virus
gi|665932|gb|AAA79871.1| ORF I Cassava vein mosaic virus
gi|9627995|ref|NP 056846.1| coat protein/movement protein Cassava vein mosaic virus
79
100
17
36
73
93
99
66
100
100
100
0.2

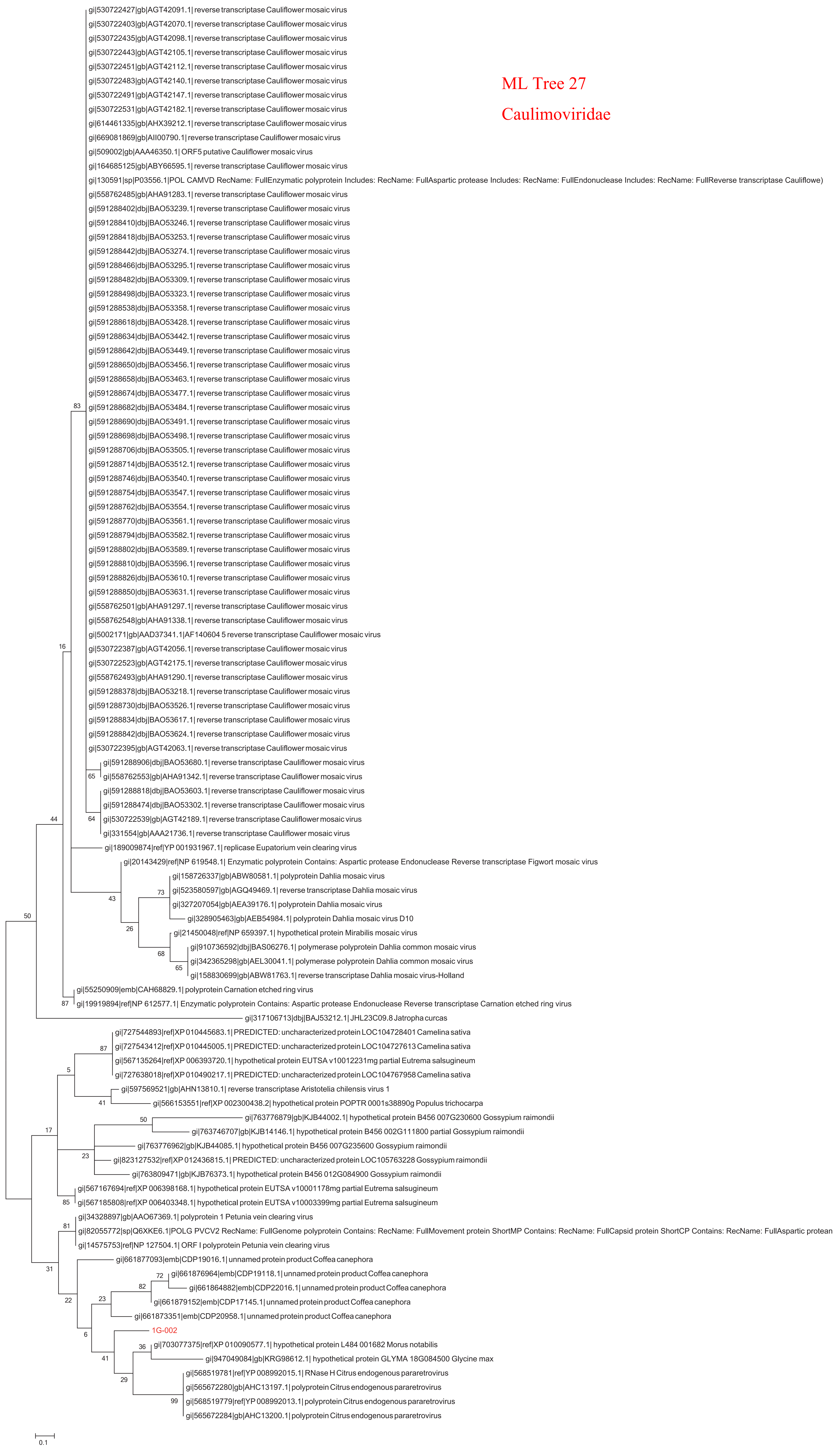
ML Tree 27
Caulimoviridae
gi|530722427|gb|AGT42091.1| reverse transcriptase Cauliflower mosaic virus
gi|530722403|gb|AGT42070.1| reverse transcriptase Cauliflower mosaic virus
gi|530722435|gb|AGT42098.1| reverse transcriptase Cauliflower mosaic virus
gi|530722443|gb|AGT42105.1| reverse transcriptase Cauliflower mosaic virus
gi|530722451|gb|AGT42112.1| reverse transcriptase Cauliflower mosaic virus
gi|530722483|gb|AGT42140.1| reverse transcriptase Cauliflower mosaic virus
gi|530722491|gb|AGT42147.1| reverse transcriptase Cauliflower mosaic virus
gi|530722531|gb|AGT42182.1| reverse transcriptase Cauliflower mosaic virus
gi|614461335|gb|AHX39212.1| reverse transcriptase Cauliflower mosaic virus
gi|669081869|gb|AII00790.1| reverse transcriptase Cauliflower mosaic virus
gi|509002|gb|AAA46350.1| ORF5 putative Cauliflower mosaic virus
gi|164685125|gb|ABY66595.1| reverse transcriptase Cauliflower mosaic virus
gi|130591|sp|P03556.1|POL CAMVD RecName: FullEnzymatic polyprotein Includes: RecName: FullAspartic protease Includes: RecName: FullEndonuclease Includes: RecName: FullReverse transcriptase Cauliflowe)
gi|558762485|gb|AHA91283.1| reverse transcriptase Cauliflower mosaic virus
gi|591288402|dbj|BAO53239.1| reverse transcriptase Cauliflower mosaic virus
gi|591288410|dbj|BAO53246.1| reverse transcriptase Cauliflower mosaic virus
gi|591288418|dbj|BAO53253.1| reverse transcriptase Cauliflower mosaic virus
gi|591288442|dbj|BAO53274.1| reverse transcriptase Cauliflower mosaic virus
gi|591288466|dbj|BAO53295.1| reverse transcriptase Cauliflower mosaic virus
gi|591288482|dbj|BAO53309.1| reverse transcriptase Cauliflower mosaic virus
gi|591288498|dbj|BAO53323.1| reverse transcriptase Cauliflower mosaic virus
gi|591288538|dbj|BAO53358.1| reverse transcriptase Cauliflower mosaic virus
gi|591288618|dbj|BAO53428.1| reverse transcriptase Cauliflower mosaic virus
gi|591288634|dbj|BAO53442.1| reverse transcriptase Cauliflower mosaic virus
gi|591288642|dbj|BAO53449.1| reverse transcriptase Cauliflower mosaic virus
gi|591288650|dbj|BAO53456.1| reverse transcriptase Cauliflower mosaic virus
gi|591288658|dbj|BAO53463.1| reverse transcriptase Cauliflower mosaic virus
gi|591288674|dbj|BAO53477.1| reverse transcriptase Cauliflower mosaic virus
gi|591288682|dbj|BAO53484.1| reverse transcriptase Cauliflower mosaic virus
gi|591288690|dbj|BAO53491.1| reverse transcriptase Cauliflower mosaic virus
gi|591288698|dbj|BAO53498.1| reverse transcriptase Cauliflower mosaic virus
gi|591288706|dbj|BAO53505.1| reverse transcriptase Cauliflower mosaic virus
gi|591288714|dbj|BAO53512.1| reverse transcriptase Cauliflower mosaic virus
gi|591288746|dbj|BAO53540.1| reverse transcriptase Cauliflower mosaic virus
gi|591288754|dbj|BAO53547.1| reverse transcriptase Cauliflower mosaic virus
gi|591288762|dbj|BAO53554.1| reverse transcriptase Cauliflower mosaic virus
gi|591288770|dbj|BAO53561.1| reverse transcriptase Cauliflower mosaic virus
gi|591288794|dbj|BAO53582.1| reverse transcriptase Cauliflower mosaic virus
gi|591288802|dbj|BAO53589.1| reverse transcriptase Cauliflower mosaic virus
gi|591288810|dbj|BAO53596.1| reverse transcriptase Cauliflower mosaic virus
gi|591288826|dbj|BAO53610.1| reverse transcriptase Cauliflower mosaic virus
gi|591288850|dbj|BAO53631.1| reverse transcriptase Cauliflower mosaic virus
gi|558762501|gb|AHA91297.1| reverse transcriptase Cauliflower mosaic virus
gi|558762548|gb|AHA91338.1| reverse transcriptase Cauliflower mosaic virus
gi|5002171|gb|AAD37341.1|AF140604 5 reverse transcriptase Cauliflower mosaic virus
gi|530722387|gb|AGT42056.1| reverse transcriptase Cauliflower mosaic virus
gi|530722523|gb|AGT42175.1| reverse transcriptase Cauliflower mosaic virus
gi|558762493|gb|AHA91290.1| reverse transcriptase Cauliflower mosaic virus
gi|591288378|dbj|BAO53218.1| reverse transcriptase Cauliflower mosaic virus
gi|591288730|dbj|BAO53526.1| reverse transcriptase Cauliflower mosaic virus
gi|591288834|dbj|BAO53617.1| reverse transcriptase Cauliflower mosaic virus
gi|591288842|dbj|BAO53624.1| reverse transcriptase Cauliflower mosaic virus
gi|530722395|gb|AGT42063.1| reverse transcriptase Cauliflower mosaic virus
gi|591288906|dbj|BAO53680.1| reverse transcriptase Cauliflower mosaic virus
gi|558762553|gb|AHA91342.1| reverse transcriptase Cauliflower mosaic virus
gi|591288818|dbj|BAO53603.1| reverse transcriptase Cauliflower mosaic virus
gi|591288474|dbj|BAO53302.1| reverse transcriptase Cauliflower mosaic virus
gi|530722539|gb|AGT42189.1| reverse transcriptase Cauliflower mosaic virus
gi|331554|gb|AAA21736.1| reverse transcriptase Cauliflower mosaic virus
gi|189009874|ref|YP 001931967.1| replicase Eupatorium vein clearing virus
gi|20143429|ref|NP 619548.1| Enzymatic polyprotein Contains: Aspartic protease Endonuclease Reverse transcriptase Figwort mosaic virus
gi|158726337|gb|ABW80581.1| polyprotein Dahlia mosaic virus
gi|523580597|gb|AGQ49469.1| reverse transcriptase Dahlia mosaic virus
gi|327207054|gb|AEA39176.1| polyprotein Dahlia mosaic virus
gi|328905463|gb|AEB54984.1| polyprotein Dahlia mosaic virus D10
gi|21450048|ref|NP 659397.1| hypothetical protein Mirabilis mosaic virus
gi|910736592|dbj|BAS06276.1| polymerase polyprotein Dahlia common mosaic virus
gi|342365298|gb|AEL30041.1| polymerase polyprotein Dahlia common mosaic virus
gi|158830699|gb|ABW81763.1| reverse transcriptase Dahlia mosaic virus-Holland
gi|55250909|emb|CAH68829.1| polyprotein Carnation etched ring virus
gi|19919894|ref|NP 612577.1| Enzymatic polyprotein Contains: Aspartic protease Endonuclease Reverse transcriptase Carnation etched ring virus
gi|317106713|dbj|BAJ53212.1| JHL23C09.8 Jatropha curcas
gi|727544893|ref|XP 010445683.1| PREDICTED: uncharacterized protein LOC104728401 Camelina sativa
gi|727543412|ref|XP 010445005.1| PREDICTED: uncharacterized protein LOC104727613 Camelina sativa
gi|567135264|ref|XP 006393720.1| hypothetical protein EUTSA v10012231mg partial Eutrema salsugineum
gi|727638018|ref|XP 010490217.1| PREDICTED: uncharacterized protein LOC104767958 Camelina sativa
gi|597569521|gb|AHN13810.1| reverse transcriptase Aristotelia chilensis virus 1
gi|566153551|ref|XP 002300438.2| hypothetical protein POPTR 0001s38890g Populus trichocarpa
gi|763776879|gb|KJB44002.1| hypothetical protein B456 007G230600 Gossypium raimondii
gi|763746707|gb|KJB14146.1| hypothetical protein B456 002G111800 partial Gossypium raimondii
gi|763776962|gb|KJB44085.1| hypothetical protein B456 007G235600 Gossypium raimondii
gi|823127532|ref|XP 012436815.1| PREDICTED: uncharacterized protein LOC105763228 Gossypium raimondii
gi|763809471|gb|KJB76373.1| hypothetical protein B456 012G084900 Gossypium raimondii
gi|567167694|ref|XP 006398168.1| hypothetical protein EUTSA v10001178mg partial Eutrema salsugineum
gi|567185808|ref|XP 006403348.1| hypothetical protein EUTSA v10003399mg partial Eutrema salsugineum
gi|34328897|gb|AAO67369.1| polyprotein 1 Petunia vein clearing virus
gi|82055772|sp|Q6XKE6.1|POLG PVCV2 RecName: FullGenome polyprotein Contains: RecName: FullMovement protein ShortMP Contains: RecName: FullCapsid protein ShortCP Contains: RecName: FullAspartic protean
gi|14575753|ref|NP 127504.1| ORF I polyprotein Petunia vein clearing virus
gi|661877093|emb|CDP19016.1| unnamed protein product Coffea canephora
gi|661876964|emb|CDP19118.1| unnamed protein product Coffea canephora
gi|661864882|emb|CDP22016.1| unnamed protein product Coffea canephora
gi|661879152|emb|CDP17145.1| unnamed protein product Coffea canephora
gi|661873351|emb|CDP20958.1| unnamed protein product Coffea canephora
1G-002
gi|703077375|ref|XP 010090577.1| hypothetical protein L484 001682 Morus notabilis
gi|947049084|gb|KRG98612.1| hypothetical protein GLYMA 18G084500 Glycine max
gi|568519781|ref|YP 008992015.1| RNase H Citrus endogenous pararetrovirus
gi|565672280|gb|AHC13197.1| polyprotein Citrus endogenous pararetrovirus
gi|568519779|ref|YP 008992013.1| polyprotein Citrus endogenous pararetrovirus
gi|565672284|gb|AHC13200.1| polyprotein Citrus endogenous pararetrovirus
0.1

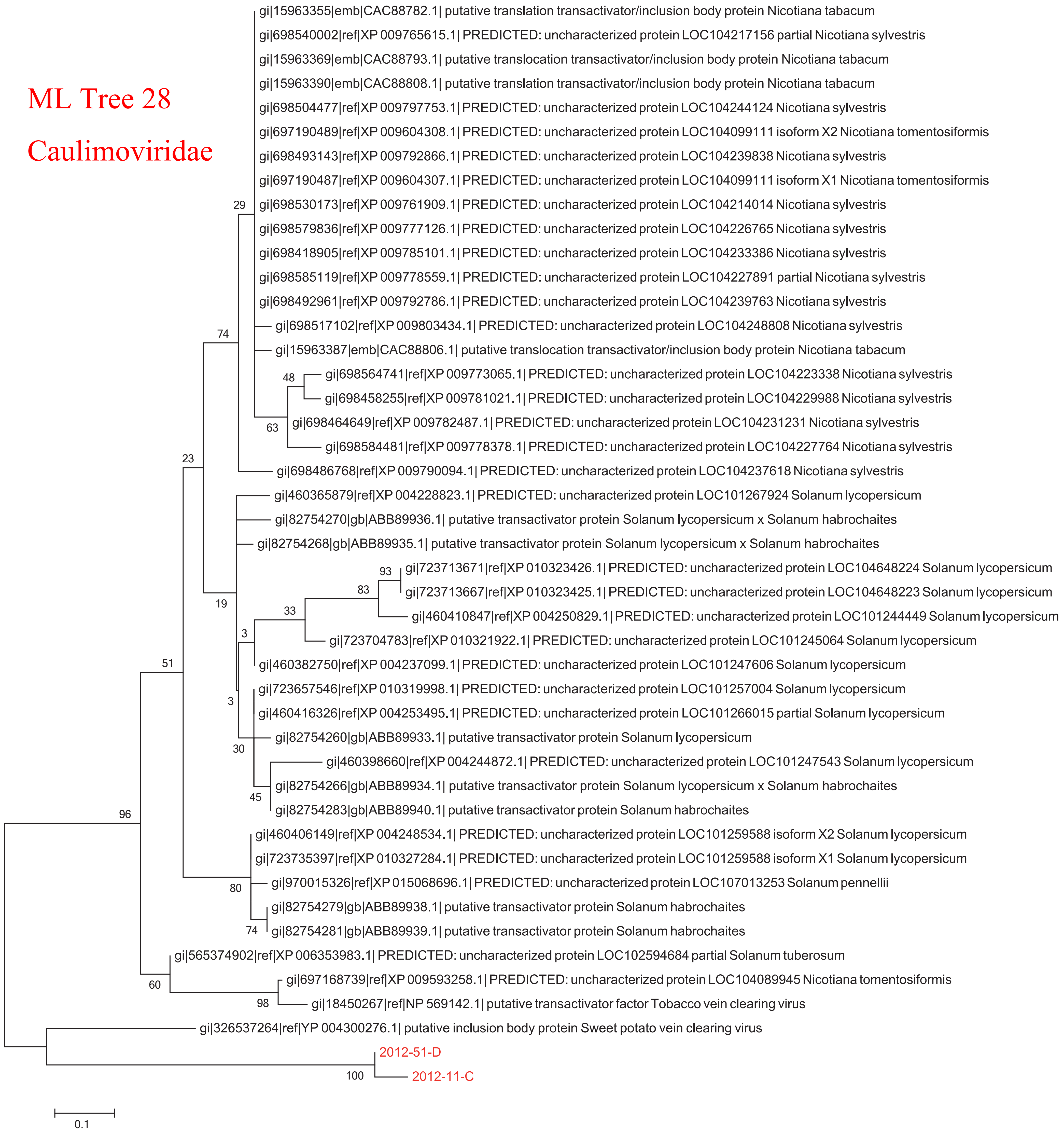
ML Tree 28
Caulimoviridae
gi|15963355|emb|CAC88782.1| putative translation transactivator/inclusion body protein Nicotiana tabacum
gi|698540002|ref|XP 009765615.1| PREDICTED: uncharacterized protein LOC104217156 partial Nicotiana sylvestris
gi|15963369|emb|CAC88793.1| putative translocation transactivator/inclusion body protein Nicotiana tabacum
gi|15963390|emb|CAC88808.1| putative translation transactivator/inclusion body protein Nicotiana tabacum
gi|698504477|ref|XP 009797753.1| PREDICTED: uncharacterized protein LOC104244124 Nicotiana sylvestris
gi|697190489|ref|XP 009604308.1| PREDICTED: uncharacterized protein LOC104099111 isoform X2 Nicotiana tomentosiformis
gi|698493143|ref|XP 009792866.1| PREDICTED: uncharacterized protein LOC104239838 Nicotiana sylvestris
gi|697190487|ref|XP 009604307.1| PREDICTED: uncharacterized protein LOC104099111 isoform X1 Nicotiana tomentosiformis
gi|698530173|ref|XP 009761909.1| PREDICTED: uncharacterized protein LOC104214014 Nicotiana sylvestris
gi|698579836|ref|XP 009777126.1| PREDICTED: uncharacterized protein LOC104226765 Nicotiana sylvestris
gi|698418905|ref|XP 009785101.1| PREDICTED: uncharacterized protein LOC104233386 Nicotiana sylvestris
gi|698585119|ref|XP 009778559.1| PREDICTED: uncharacterized protein LOC104227891 partial Nicotiana sylvestris
gi|698492961|ref|XP 009792786.1| PREDICTED: uncharacterized protein LOC104239763 Nicotiana sylvestris
gi|698517102|ref|XP 009803434.1| PREDICTED: uncharacterized protein LOC104248808 Nicotiana sylvestris
gi|15963387|emb|CAC88806.1| putative translocation transactivator/inclusion body protein Nicotiana tabacum
gi|698564741|ref|XP 009773065.1| PREDICTED: uncharacterized protein LOC104223338 Nicotiana sylvestris
gi|698458255|ref|XP 009781021.1| PREDICTED: uncharacterized protein LOC104229988 Nicotiana sylvestris
gi|698464649|ref|XP 009782487.1| PREDICTED: uncharacterized protein LOC104231231 Nicotiana sylvestris
gi|698584481|ref|XP 009778378.1| PREDICTED: uncharacterized protein LOC104227764 Nicotiana sylvestris
gi|698486768|ref|XP 009790094.1| PREDICTED: uncharacterized protein LOC104237618 Nicotiana sylvestris
gi|460365879|ref|XP 004228823.1| PREDICTED: uncharacterized protein LOC101267924 Solanum lycopersicum
gi|82754270|gb|ABB89936.1| putative transactivator protein Solanum lycopersicum x Solanum habrochaites
gi|82754268|gb|ABB89935.1| putative transactivator protein Solanum lycopersicum x Solanum habrochaites
gi|723713671|ref|XP 010323426.1| PREDICTED: uncharacterized protein LOC104648224 Solanum lycopersicum
gi|723713667|ref|XP 010323425.1| PREDICTED: uncharacterized protein LOC104648223 Solanum lycopersicum
gi|460410847|ref|XP 004250829.1| PREDICTED: uncharacterized protein LOC101244449 Solanum lycopersicum
gi|723704783|ref|XP 010321922.1| PREDICTED: uncharacterized protein LOC101245064 Solanum lycopersicum
gi|460382750|ref|XP 004237099.1| PREDICTED: uncharacterized protein LOC101247606 Solanum lycopersicum
gi|723657546|ref|XP 010319998.1| PREDICTED: uncharacterized protein LOC101257004 Solanum lycopersicum
gi|460416326|ref|XP 004253495.1| PREDICTED: uncharacterized protein LOC101266015 partial Solanum lycopersicum
gi|82754260|gb|ABB89933.1| putative transactivator protein Solanum lycopersicum
gi|460398660|ref|XP 004244872.1| PREDICTED: uncharacterized protein LOC101247543 Solanum lycopersicum
gi|82754266|gb|ABB89934.1| putative transactivator protein Solanum lycopersicum x Solanum habrochaites
gi|82754283|gb|ABB89940.1| putative transactivator protein Solanum habrochaites
gi|460406149|ref|XP 004248534.1| PREDICTED: uncharacterized protein LOC101259588 isoform X2 Solanum lycopersicum
gi|723735397|ref|XP 010327284.1| PREDICTED: uncharacterized protein LOC101259588 isoform X1 Solanum lycopersicum
gi|970015326|ref|XP 015068696.1| PREDICTED: uncharacterized protein LOC107013253 Solanum pennellii
gi|82754279|gb|ABB89938.1| putative transactivator protein Solanum habrochaites
gi|82754281|gb|ABB89939.1| putative transactivator protein Solanum habrochaites
gi|565374902|ref|XP 006353983.1| PREDICTED: uncharacterized protein LOC102594684 partial Solanum tuberosum
gi|697168739|ref|XP 009593258.1| PREDICTED: uncharacterized protein LOC104089945 Nicotiana tomentosiformis
gi|18450267|ref|NP 569142.1| putative transactivator factor Tobacco vein clearing virus
gi|326537264|ref|YP 004300276.1| putative inclusion body protein Sweet potato vein clearing virus
2012-51-D
2012-11-C
29
74
48
63
23
19
93
83
33
3
3
30
45
51
96
80
74
60
98
100
0.1

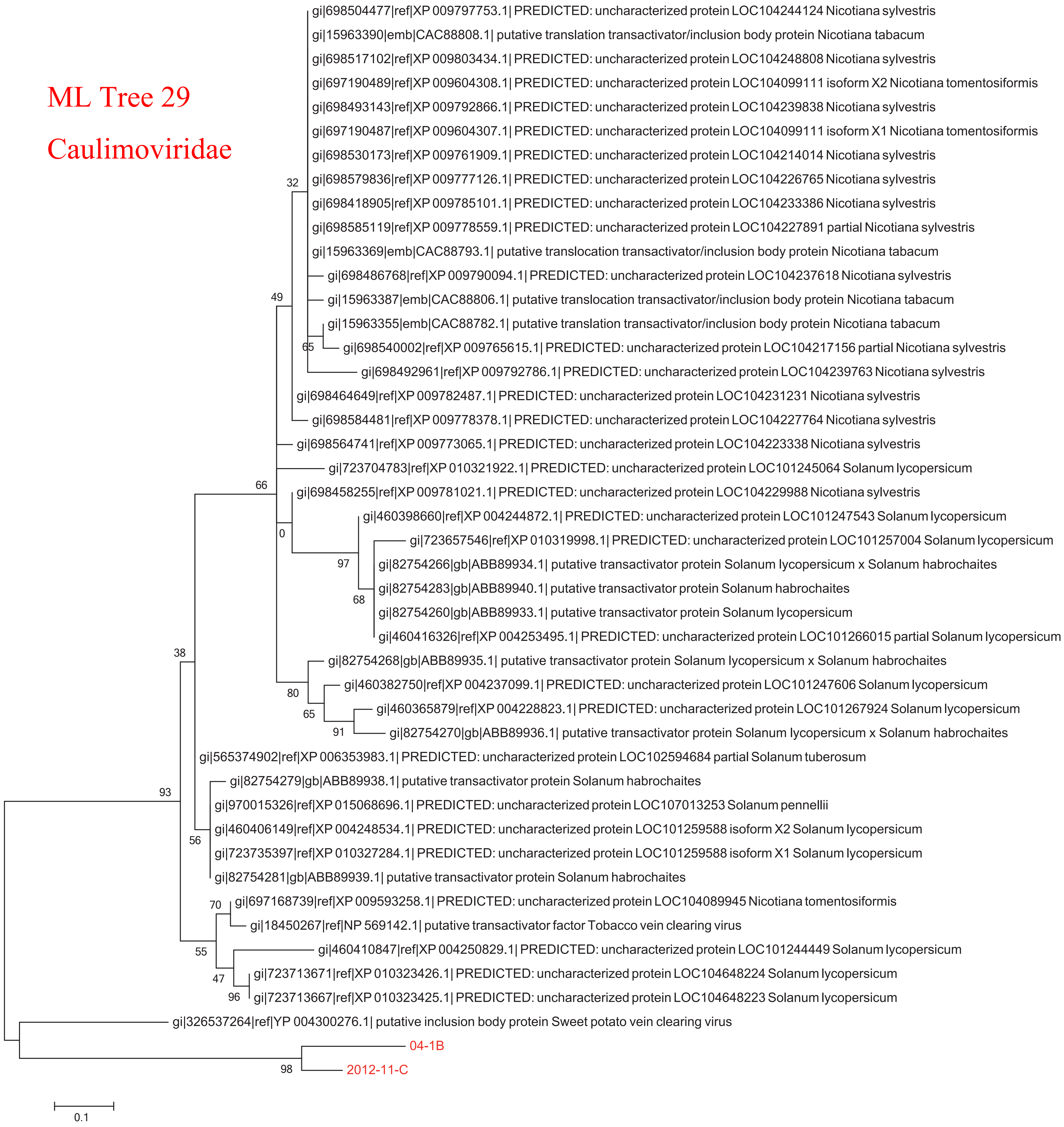
ML Tree 29
Caulimoviridae
gi|698504477|ref|XP 009797753.1| PREDICTED: uncharacterized protein LOC104244124 Nicotiana sylvestris
gi|15963390|emb|CAC88808.1| putative translation transactivator/inclusion body protein Nicotiana tabacum
gi|698517102|ref|XP 009803434.1| PREDICTED: uncharacterized protein LOC104248808 Nicotiana sylvestris
gi|697190489|ref|XP 009604308.1| PREDICTED: uncharacterized protein LOC104099111 isoform X2 Nicotiana tomentosiformis
gi|698493143|ref|XP 009792866.1| PREDICTED: uncharacterized protein LOC104239838 Nicotiana sylvestris
gi|697190487|ref|XP 009604307.1| PREDICTED: uncharacterized protein LOC104099111 isoform X1 Nicotiana tomentosiformis
gi|698530173|ref|XP 009761909.1| PREDICTED: uncharacterized protein LOC104214014 Nicotiana sylvestris
gi|698579836|ref|XP 009777126.1| PREDICTED: uncharacterized protein LOC104226765 Nicotiana sylvestris
gi|698418905|ref|XP 009785101.1| PREDICTED: uncharacterized protein LOC104233386 Nicotiana sylvestris
gi|698585119|ref|XP 009778559.1| PREDICTED: uncharacterized protein LOC104227891 partial Nicotiana sylvestris
gi|15963369|emb|CAC88793.1| putative translocation transactivator/inclusion body protein Nicotiana tabacum
gi|698486768|ref|XP 009790094.1| PREDICTED: uncharacterized protein LOC104237618 Nicotiana sylvestris
gi|15963387|emb|CAC88806.1| putative translocation transactivator/inclusion body protein Nicotiana tabacum
gi|15963355|emb|CAC88782.1| putative translation transactivator/inclusion body protein Nicotiana tabacum
gi|698540002|ref|XP 009765615.1| PREDICTED: uncharacterized protein LOC104217156 partial Nicotiana sylvestris
gi|698492961|ref|XP 009792786.1| PREDICTED: uncharacterized protein LOC104239763 Nicotiana sylvestris
gi|698464649|ref|XP 009782487.1| PREDICTED: uncharacterized protein LOC104231231 Nicotiana sylvestris
gi|698584481|ref|XP 009778378.1| PREDICTED: uncharacterized protein LOC104227764 Nicotiana sylvestris
gi|698564741|ref|XP 009773065.1| PREDICTED: uncharacterized protein LOC104223338 Nicotiana sylvestris
gi|723704783|ref|XP 010321922.1| PREDICTED: uncharacterized protein LOC101245064 Solanum lycopersicum
gi|698458255|ref|XP 009781021.1| PREDICTED: uncharacterized protein LOC104229988 Nicotiana sylvestris
gi|460398660|ref|XP 004244872.1| PREDICTED: uncharacterized protein LOC101247543 Solanum lycopersicum
gi|723657546|ref|XP 010319998.1| PREDICTED: uncharacterized protein LOC101257004 Solanum lycopersicum
gi|82754266|gb|ABB89934.1| putative transactivator protein Solanum lycopersicum x Solanum habrochaites
gi|82754283|gb|ABB89940.1| putative transactivator protein Solanum habrochaites
gi|82754260|gb|ABB89933.1| putative transactivator protein Solanum lycopersicum
gi|460416326|ref|XP 004253495.1| PREDICTED: uncharacterized protein LOC101266015 partial Solanum lycopersicum
gi|82754268|gb|ABB89935.1| putative transactivator protein Solanum lycopersicum x Solanum habrochaites
gi|460382750|ref|XP 004237099.1| PREDICTED: uncharacterized protein LOC101247606 Solanum lycopersicum
gi|460365879|ref|XP 004228823.1| PREDICTED: uncharacterized protein LOC101267924 Solanum lycopersicum
gi|82754270|gb|ABB89936.1| putative transactivator protein Solanum lycopersicum x Solanum habrochaites
gi|565374902|ref|XP 006353983.1| PREDICTED: uncharacterized protein LOC102594684 partial Solanum tuberosum
gi|82754279|gb|ABB89938.1| putative transactivator protein Solanum habrochaites
gi|970015326|ref|XP 015068696.1| PREDICTED: uncharacterized protein LOC107013253 Solanum pennellii
gi|460406149|ref|XP 004248534.1| PREDICTED: uncharacterized protein LOC101259588 isoform X2 Solanum lycopersicum
gi|723735397|ref|XP 010327284.1| PREDICTED: uncharacterized protein LOC101259588 isoform X1 Solanum lycopersicum
gi|82754281|gb|ABB89939.1| putative transactivator protein Solanum habrochaites
gi|697168739|ref|XP 009593258.1| PREDICTED: uncharacterized protein LOC104089945 Nicotiana tomentosiformis
gi|18450267|ref|NP 569142.1| putative transactivator factor Tobacco vein clearing virus
gi|460410847|ref|XP 004250829.1| PREDICTED: uncharacterized protein LOC101244449 Solanum lycopersicum
gi|723713671|ref|XP 010323426.1| PREDICTED: uncharacterized protein LOC104648224 Solanum lycopersicum
gi|723713667|ref|XP 010323425.1| PREDICTED: uncharacterized protein LOC104648223 Solanum lycopersicum
gi|326537264|ref|YP 004300276.1| putative inclusion body protein Sweet potato vein clearing virus
04-1B
2012-11-C
0.1

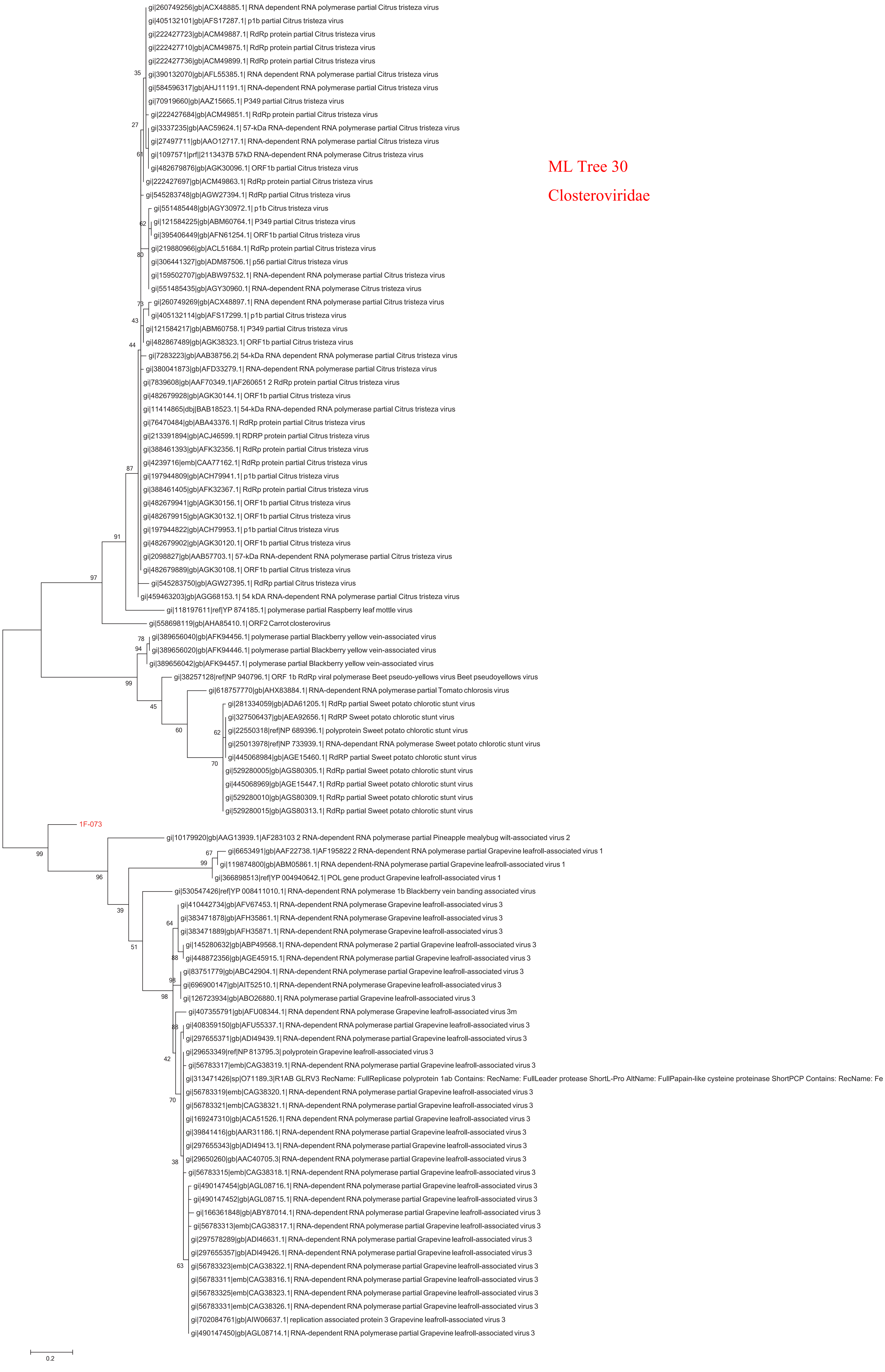
ML Tree 30
Closteroviridae
gi|260749256|gb|ACX48885.1| RNA dependent RNA polymerase partial Citrus tristeza virus
gi|405132101|gb|AFS17287.1| p1b partial Citrus tristeza virus
gi|222427723|gb|ACM49887.1| RdRp protein partial Citrus tristeza virus
gi|222427710|gb|ACM49875.1| RdRp protein partial Citrus tristeza virus
gi|222427736|gb|ACM49899.1| RdRp protein partial Citrus tristeza virus
gi|390132070|gb|AFL55385.1| RNA dependent RNA polymerase partial Citrus tristeza virus
gi|584596317|gb|AHJ11191.1| RNA-dependent RNA polymerase partial Citrus tristeza virus
gi|70919660|gb|AAZ15665.1| P349 partial Citrus tristeza virus
gi|222427684|gb|ACM49851.1| RdRp protein partial Citrus tristeza virus
gi|3337235|gb|AAC59624.1| 57-kDa RNA-dependent RNA polymerase partial Citrus tristeza virus
gi|27497711|gb|AAO12717.1| RNA-dependent RNA polymerase partial Citrus tristeza virus
gi|1097571|prf||2113437B 57kD RNA-dependent RNA polymerase Citrus tristeza virus
gi|482679876|gb|AGK30096.1| ORF1b partial Citrus tristeza virus
gi|222427697|gb|ACM49863.1| RdRp protein partial Citrus tristeza virus
gi|545283748|gb|AGW27394.1| RdRp partial Citrus tristeza virus
gi|551485448|gb|AGY30972.1| p1b Citrus tristeza virus
gi|121584225|gb|ABM60764.1| P349 partial Citrus tristeza virus
gi|395406449|gb|AFN61254.1| ORF1b partial Citrus tristeza virus
gi|219880966|gb|ACL51684.1| RdRp protein partial Citrus tristeza virus
gi|306441327|gb|ADM87506.1| p56 partial Citrus tristeza virus
gi|159502707|gb|ABW97532.1| RNA-dependent RNA polymerase partial Citrus tristeza virus
gi|551485435|gb|AGY30960.1| RNA-dependent RNA polymerase Citrus tristeza virus
gi|260749269|gb|ACX48897.1| RNA dependent RNA polymerase partial Citrus tristeza virus
gi|405132114|gb|AFS17299.1| p1b partial Citrus tristeza virus
gi|121584217|gb|ABM60758.1| P349 partial Citrus tristeza virus
gi|482867489|gb|AGK38323.1| ORF1b partial Citrus tristeza virus
gi|7283223|gb|AAB38756.2| 54-kDa RNA dependent RNA polymerase partial Citrus tristeza virus
gi|380041873|gb|AFD33279.1| RNA-dependent RNA polymerase partial Citrus tristeza virus
gi|7839608|gb|AAF70349.1|AF260651 2 RdRp protein partial Citrus tristeza virus
gi|482679928|gb|AGK30144.1| ORF1b partial Citrus tristeza virus
gi|11414865|dbj|BAB18523.1| 54-kDa RNA-depended RNA polymerase partial Citrus tristeza virus
gi|76470484|gb|ABA43376.1| RdRp protein partial Citrus tristeza virus
gi|213391894|gb|ACJ46599.1| RDRP protein partial Citrus tristeza virus
gi|388461393|gb|AFK32356.1| RdRp protein partial Citrus tristeza virus
gi|4239716|emb|CAA77162.1| RdRp protein partial Citrus tristeza virus
gi|197944809|gb|ACH79941.1| p1b partial Citrus tristeza virus
gi|388461405|gb|AFK32367.1| RdRp protein partial Citrus tristeza virus
gi|482679941|gb|AGK30156.1| ORF1b partial Citrus tristeza virus
gi|482679915|gb|AGK30132.1| ORF1b partial Citrus tristeza virus
gi|197944822|gb|ACH79953.1| p1b partial Citrus tristeza virus
gi|482679902|gb|AGK30120.1| ORF1b partial Citrus tristeza virus
gi|2098827|gb|AAB57703.1| 57-kDa RNA-dependent RNA polymerase partial Citrus tristeza virus
gi|482679889|gb|AGK30108.1| ORF1b partial Citrus tristeza virus
gi|545283750|gb|AGW27395.1| RdRp partial Citrus tristeza virus
gi|459463203|gb|AGG68153.1| 54 kDA RNA-dependent RNA polymerase partial Citrus tristeza virus
gi|118197611|ref|YP 874185.1| polymerase partial Raspberry leaf mottle virus
gi|558698119|gb|AHA85410.1| ORF2 Carrot closterovirus
gi|389656040|gb|AFK94456.1| polymerase partial Blackberry yellow vein-associated virus
gi|389656020|gb|AFK94446.1| polymerase partial Blackberry yellow vein-associated virus
gi|389656042|gb|AFK94457.1| polymerase partial Blackberry yellow vein-associated virus
gi|38257128|ref|NP 940796.1| ORF 1b RdRp viral polymerase Beet pseudo-yellows virus Beet pseudoyellows virus
gi|618757770|gb|AHX83884.1| RNA-dependent RNA polymerase partial Tomato chlorosis virus
gi|281334059|gb|ADA61205.1| RdRp partial Sweet potato chlorotic stunt virus
gi|327506437|gb|AEA92656.1| RdRP Sweet potato chlorotic stunt virus
gi|22550318|ref|NP 689396.1| polyprotein Sweet potato chlorotic stunt virus
gi|25013978|ref|NP 733939.1| RNA-dependant RNA polymerase Sweet potato chlorotic stunt virus
gi|445068984|gb|AGE15460.1| RdRP partial Sweet potato chlorotic stunt virus
gi|529280005|gb|AGS80305.1| RdRp partial Sweet potato chlorotic stunt virus
gi|445068969|gb|AGE15447.1| RdRp partial Sweet potato chlorotic stunt virus
gi|529280010|gb|AGS80309.1| RdRp partial Sweet potato chlorotic stunt virus
gi|529280015|gb|AGS80313.1| RdRp partial Sweet potato chlorotic stunt virus
1F-073
gi|10179920|gb|AAG13939.1|AF283103 2 RNA-dependent RNA polymerase partial Pineapple mealybug wilt-associated virus 2
gi|6653491|gb|AAF22738.1|AF195822 2 RNA-dependent RNA polymerase partial Grapevine leafroll-associated virus 1
gi|119874800|gb|ABM05861.1| RNA dependent-RNA polymerase partial Grapevine leafroll-associated virus 1
gi|366898513|ref|YP 004940642.1| POL gene product Grapevine leafroll-associated virus 1
gi|530547426|ref|YP 008411010.1| RNA-dependent RNA polymerase 1b Blackberry vein banding associated virus
gi|410442734|gb|AFV67453.1| RNA-dependent RNA polymerase Grapevine leafroll-associated virus 3
gi|383471878|gb|AFH35861.1| RNA-dependent RNA polymerase Grapevine leafroll-associated virus 3
gi|383471889|gb|AFH35871.1| RNA-dependent RNA polymerase Grapevine leafroll-associated virus 3
gi|145280632|gb|ABP49568.1| RNA-dependent RNA polymerase 2 partial Grapevine leafroll-associated virus 3
gi|448872356|gb|AGE45915.1| RNA-dependent RNA polymerase partial Grapevine leafroll-associated virus 3
gi|83751779|gb|ABC42904.1| RNA-dependent RNA polymerase partial Grapevine leafroll-associated virus 3
gi|696900147|gb|AIT52510.1| RNA-dependent RNA polymerase Grapevine leafroll-associated virus 3
gi|126723934|gb|ABO26880.1| RNA polymerase partial Grapevine leafroll-associated virus 3
gi|407355791|gb|AFU08344.1| RNA dependent RNA polymerase Grapevine leafroll-associated virus 3m
gi|408359150|gb|AFU55337.1| RNA-dependent RNA polymerase partial Grapevine leafroll-associated virus 3
gi|297655371|gb|ADI49439.1| RNA-dependent RNA polymerase partial Grapevine leafroll-associated virus 3
gi|29653349|ref|NP 813795.3| polyprotein Grapevine leafroll-associated virus 3
gi|56783317|emb|CAG38319.1| RNA-dependent RNA polymerase partial Grapevine leafroll-associated virus 3
gi|313471426|sp|O71189.3|R1AB GLRV3 RecName: FullReplicase polyprotein 1ab Contains: RecName: FullLeader protease ShortL-Pro AltName: FullPapain-like cysteine proteinase ShortPCP Contains: RecName: Fe
gi|56783319|emb|CAG38320.1| RNA-dependent RNA polymerase partial Grapevine leafroll-associated virus 3
gi|56783321|emb|CAG38321.1| RNA-dependent RNA polymerase partial Grapevine leafroll-associated virus 3
gi|169247310|gb|ACA51526.1| RNA dependent RNA polymerase partial Grapevine leafroll-associated virus 3
gi|39841416|gb|AAR31186.1| RNA-dependent RNA polymerase partial Grapevine leafroll-associated virus 3
gi|297655343|gb|ADI49413.1| RNA-dependent RNA polymerase partial Grapevine leafroll-associated virus 3
gi|29650260|gb|AAC40705.3| RNA-dependent RNA polymerase partial Grapevine leafroll-associated virus 3
gi|56783315|emb|CAG38318.1| RNA-dependent RNA polymerase partial Grapevine leafroll-associated virus 3
gi|490147454|gb|AGL08716.1| RNA-dependent RNA polymerase partial Grapevine leafroll-associated virus 3
gi|490147452|gb|AGL08715.1| RNA-dependent RNA polymerase partial Grapevine leafroll-associated virus 3
gi|166361848|gb|ABY87014.1| RNA-dependent RNA polymerase partial Grapevine leafroll-associated virus 3
gi|56783313|emb|CAG38317.1| RNA-dependent RNA polymerase partial Grapevine leafroll-associated virus 3
gi|297578289|gb|ADI46631.1| RNA-dependent RNA polymerase partial Grapevine leafroll-associated virus 3
gi|297655357|gb|ADI49426.1| RNA-dependent RNA polymerase partial Grapevine leafroll-associated virus 3
gi|56783323|emb|CAG38322.1| RNA-dependent RNA polymerase partial Grapevine leafroll-associated virus 3
gi|56783311|emb|CAG38316.1| RNA-dependent RNA polymerase partial Grapevine leafroll-associated virus 3
gi|56783325|emb|CAG38323.1| RNA-dependent RNA polymerase partial Grapevine leafroll-associated virus 3
gi|56783331|emb|CAG38326.1| RNA-dependent RNA polymerase partial Grapevine leafroll-associated virus 3
gi|702084761|gb|AIW06637.1| replication associated protein 3 Grapevine leafroll-associated virus 3
gi|490147450|gb|AGL08714.1| RNA-dependent RNA polymerase partial Grapevine leafroll-associated virus 3
0.2

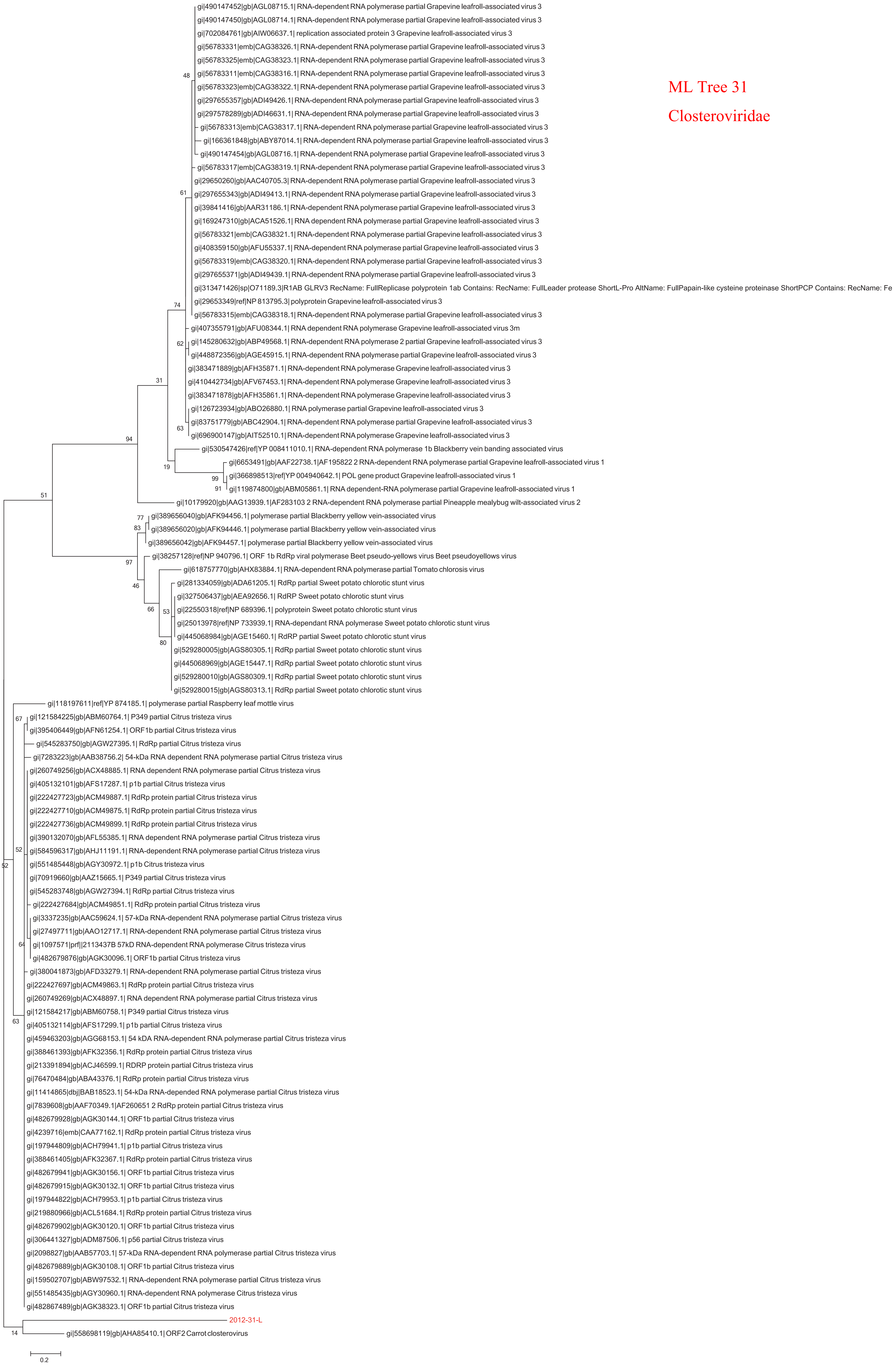

ML Tree 31
Closteroviridae
gi|490147452|gb|AGL08715.1| RNA-dependent RNA polymerase partial Grapevine leafroll-associated virus 3
gi|490147450|gb|AGL08714.1| RNA-dependent RNA polymerase partial Grapevine leafroll-associated virus 3
gi|702084761|gb|AIW06637.1| replication associated protein 3 Grapevine leafroll-associated virus 3
gi|56783331|emb|CAG38326.1| RNA-dependent RNA polymerase partial Grapevine leafroll-associated virus 3
gi|56783325|emb|CAG38323.1| RNA-dependent RNA polymerase partial Grapevine leafroll-associated virus 3
gi|56783311|emb|CAG38316.1| RNA-dependent RNA polymerase partial Grapevine leafroll-associated virus 3
48
gi|56783323|emb|CAG38322.1| RNA-dependent RNA polymerase partial Grapevine leafroll-associated virus 3
gi|297655357|gb|ADI49426.1| RNA-dependent RNA polymerase partial Grapevine leafroll-associated virus 3
gi|297578289|gb|ADI46631.1| RNA-dependent RNA polymerase partial Grapevine leafroll-associated virus 3
gi|56783313|emb|CAG38317.1| RNA-dependent RNA polymerase partial Grapevine leafroll-associated virus 3
gi|166361848|gb|ABY87014.1| RNA-dependent RNA polymerase partial Grapevine leafroll-associated virus 3
gi|490147454|gb|AGL08716.1| RNA-dependent RNA polymerase partial Grapevine leafroll-associated virus 3
gi|56783317|emb|CAG38319.1| RNA-dependent RNA polymerase partial Grapevine leafroll-associated virus 3
gi|29650260|gb|AAC40705.3| RNA-dependent RNA polymerase partial Grapevine leafroll-associated virus 3
61
gi|297655343|gb|ADI49413.1| RNA-dependent RNA polymerase partial Grapevine leafroll-associated virus 3
gi|39841416|gb|AAR31186.1| RNA-dependent RNA polymerase partial Grapevine leafroll-associated virus 3
gi|169247310|gb|ACA51526.1| RNA dependent RNA polymerase partial Grapevine leafroll-associated virus 3
gi|56783321|emb|CAG38321.1| RNA-dependent RNA polymerase partial Grapevine leafroll-associated virus 3
gi|408359150|gb|AFU55337.1| RNA-dependent RNA polymerase partial Grapevine leafroll-associated virus 3
gi|56783319|emb|CAG38320.1| RNA-dependent RNA polymerase partial Grapevine leafroll-associated virus 3
gi|297655371|gb|ADI49439.1| RNA-dependent RNA polymerase partial Grapevine leafroll-associated virus 3
gi|313471426|sp|O71189.3|R1AB GLRV3 RecName: FullReplicase polyprotein 1ab Contains: RecName: FullLeader protease ShortL-Pro AltName: FullPapain-like cysteine proteinase ShortPCP Contains: RecName: Fe
gi|29653349|ref|NP 813795.3| polyprotein Grapevine leafroll-associated virus 3
74
gi|56783315|emb|CAG38318.1| RNA-dependent RNA polymerase partial Grapevine leafroll-associated virus 3
gi|407355791|gb|AFU08344.1| RNA dependent RNA polymerase Grapevine leafroll-associated virus 3m
62
gi|145280632|gb|ABP49568.1| RNA-dependent RNA polymerase 2 partial Grapevine leafroll-associated virus 3
gi|448872356|gb|AGE45915.1| RNA-dependent RNA polymerase partial Grapevine leafroll-associated virus 3
gi|383471889|gb|AFH35871.1| RNA-dependent RNA polymerase Grapevine leafroll-associated virus 3
31
gi|410442734|gb|AFV67453.1| RNA-dependent RNA polymerase Grapevine leafroll-associated virus 3
gi|383471878|gb|AFH35861.1| RNA-dependent RNA polymerase Grapevine leafroll-associated virus 3
gi|126723934|gb|ABO26880.1| RNA polymerase partial Grapevine leafroll-associated virus 3
63
gi|83751779|gb|ABC42904.1| RNA-dependent RNA polymerase partial Grapevine leafroll-associated virus 3
gi|696900147|gb|AIT52510.1| RNA-dependent RNA polymerase Grapevine leafroll-associated virus 3
94
gi|530547426|ref|YP 008411010.1| RNA-dependent RNA polymerase 1b Blackberry vein banding associated virus
gi|6653491|gb|AAF22738.1|AF195822 2 RNA-dependent RNA polymerase partial Grapevine leafroll-associated virus 1
19
99
gi|366898513|ref|YP 004940642.1| POL gene product Grapevine leafroll-associated virus 1
91
gi|119874800|gb|ABM05861.1| RNA dependent-RNA polymerase partial Grapevine leafroll-associated virus 1
51
gi|10179920|gb|AAG13939.1|AF283103 2 RNA-dependent RNA polymerase partial Pineapple mealybug wilt-associated virus 2
77
gi|389656040|gb|AFK94456.1| polymerase partial Blackberry yellow vein-associated virus
83
gi|389656020|gb|AFK94446.1| polymerase partial Blackberry yellow vein-associated virus
gi|389656042|gb|AFK94457.1| polymerase partial Blackberry yellow vein-associated virus
97
gi|38257128|ref|NP 940796.1| ORF 1b RdRp viral polymerase Beet pseudo-yellows virus Beet pseudoyellows virus
gi|618757770|gb|AHX83884.1| RNA-dependent RNA polymerase partial Tomato chlorosis virus
46
gi|281334059|gb|ADA61205.1| RdRp partial Sweet potato chlorotic stunt virus
gi|327506437|gb|AEA92656.1| RdRP Sweet potato chlorotic stunt virus
66
53
gi|22550318|ref|NP 689396.1| polyprotein Sweet potato chlorotic stunt virus
gi|25013978|ref|NP 733939.1| RNA-dependant RNA polymerase Sweet potato chlorotic stunt virus
80
gi|445068984|gb|AGE15460.1| RdRP partial Sweet potato chlorotic stunt virus
gi|529280005|gb|AGS80305.1| RdRp partial Sweet potato chlorotic stunt virus
gi|445068969|gb|AGE15447.1| RdRp partial Sweet potato chlorotic stunt virus
gi|529280010|gb|AGS80309.1| RdRp partial Sweet potato chlorotic stunt virus
gi|529280015|gb|AGS80313.1| RdRp partial Sweet potato chlorotic stunt virus
gi|118197611|ref|YP 874185.1| polymerase partial Raspberry leaf mottle virus
67
gi|121584225|gb|ABM60764.1| P349 partial Citrus tristeza virus
gi|395406449|gb|AFN61254.1| ORF1b partial Citrus tristeza virus
gi|545283750|gb|AGW27395.1| RdRp partial Citrus tristeza virus
gi|7283223|gb|AAB38756.2| 54-kDa RNA dependent RNA polymerase partial Citrus tristeza virus
gi|260749256|gb|ACX48885.1| RNA dependent RNA polymerase partial Citrus tristeza virus
gi|405132101|gb|AFS17287.1| p1b partial Citrus tristeza virus
gi|222427723|gb|ACM49887.1| RdRp protein partial Citrus tristeza virus
gi|222427710|gb|ACM49875.1| RdRp protein partial Citrus tristeza virus
gi|222427736|gb|ACM49899.1| RdRp protein partial Citrus tristeza virus
gi|390132070|gb|AFL55385.1| RNA dependent RNA polymerase partial Citrus tristeza virus
52
gi|584596317|gb|AHJ11191.1| RNA-dependent RNA polymerase partial Citrus tristeza virus
52
gi|551485448|gb|AGY30972.1| p1b Citrus tristeza virus
gi|70919660|gb|AAZ15665.1| P349 partial Citrus tristeza virus
gi|545283748|gb|AGW27394.1| RdRp partial Citrus tristeza virus
gi|222427684|gb|ACM49851.1| RdRp protein partial Citrus tristeza virus
gi|3337235|gb|AAC59624.1| 57-kDa RNA-dependent RNA polymerase partial Citrus tristeza virus
gi|27497711|gb|AAO12717.1| RNA-dependent RNA polymerase partial Citrus tristeza virus
64
gi|1097571|prf||2113437B 57kD RNA-dependent RNA polymerase Citrus tristeza virus
gi|482679876|gb|AGK30096.1| ORF1b partial Citrus tristeza virus
gi|380041873|gb|AFD33279.1| RNA-dependent RNA polymerase partial Citrus tristeza virus
gi|222427697|gb|ACM49863.1| RdRp protein partial Citrus tristeza virus
gi|260749269|gb|ACX48897.1| RNA dependent RNA polymerase partial Citrus tristeza virus
gi|121584217|gb|ABM60758.1| P349 partial Citrus tristeza virus
63
gi|405132114|gb|AFS17299.1| p1b partial Citrus tristeza virus
gi|459463203|gb|AGG68153.1| 54 kDA RNA-dependent RNA polymerase partial Citrus tristeza virus
gi|388461393|gb|AFK32356.1| RdRp protein partial Citrus tristeza virus
gi|213391894|gb|ACJ46599.1| RDRP protein partial Citrus tristeza virus
gi|76470484|gb|ABA43376.1| RdRp protein partial Citrus tristeza virus
gi|11414865|dbj|BAB18523.1| 54-kDa RNA-depended RNA polymerase partial Citrus tristeza virus
gi|7839608|gb|AAF70349.1|AF260651 2 RdRp protein partial Citrus tristeza virus
gi|482679928|gb|AGK30144.1| ORF1b partial Citrus tristeza virus
gi|4239716|emb|CAA77162.1| RdRp protein partial Citrus tristeza virus
gi|197944809|gb|ACH79941.1| p1b partial Citrus tristeza virus
gi|388461405|gb|AFK32367.1| RdRp protein partial Citrus tristeza virus
gi|482679941|gb|AGK30156.1| ORF1b partial Citrus tristeza virus
gi|482679915|gb|AGK30132.1| ORF1b partial Citrus tristeza virus
gi|197944822|gb|ACH79953.1| p1b partial Citrus tristeza virus
gi|219880966|gb|ACL51684.1| RdRp protein partial Citrus tristeza virus
gi|482679902|gb|AGK30120.1| ORF1b partial Citrus tristeza virus
gi|306441327|gb|ADM87506.1| p56 partial Citrus tristeza virus
gi|2098827|gb|AAB57703.1| 57-kDa RNA-dependent RNA polymerase partial Citrus tristeza virus
gi|482679889|gb|AGK30108.1| ORF1b partial Citrus tristeza virus
gi|159502707|gb|ABW97532.1| RNA-dependent RNA polymerase partial Citrus tristeza virus
gi|551485435|gb|AGY30960.1| RNA-dependent RNA polymerase Citrus tristeza virus
gi|482867489|gb|AGK38323.1| ORF1b partial Citrus tristeza virus
2012-31-L
14
gi|558698119|gb|AHA85410.1| ORF2 Carrot closterovirus
0.2

ML Tree 32

Closteroviridae

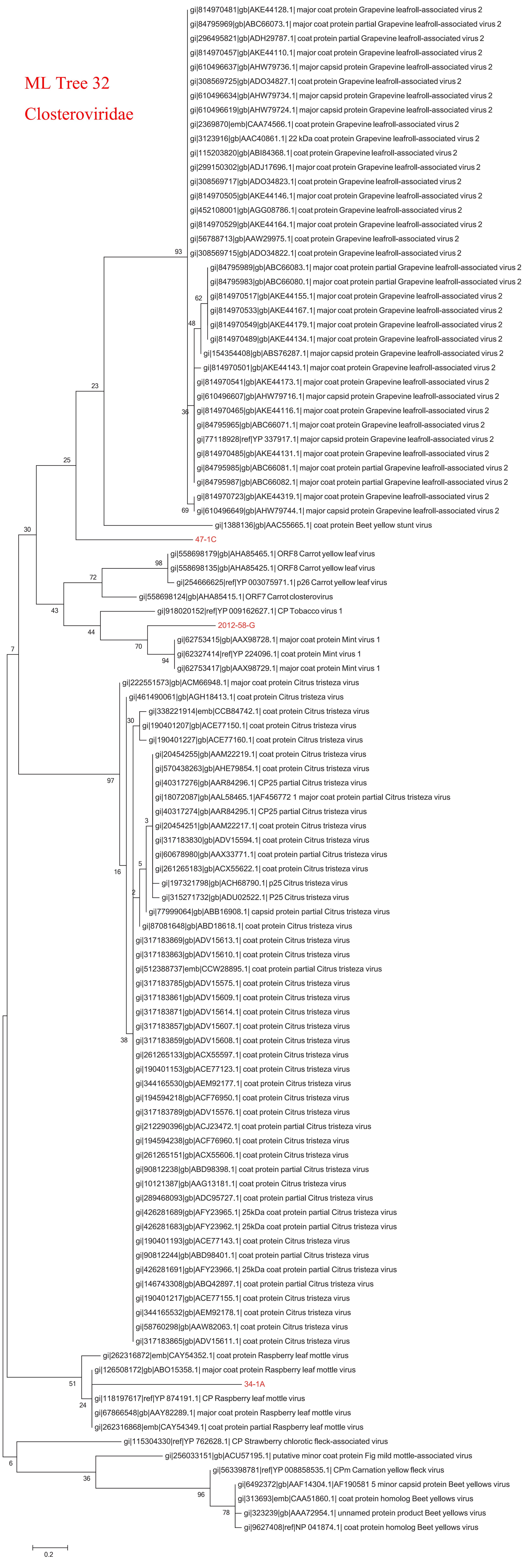

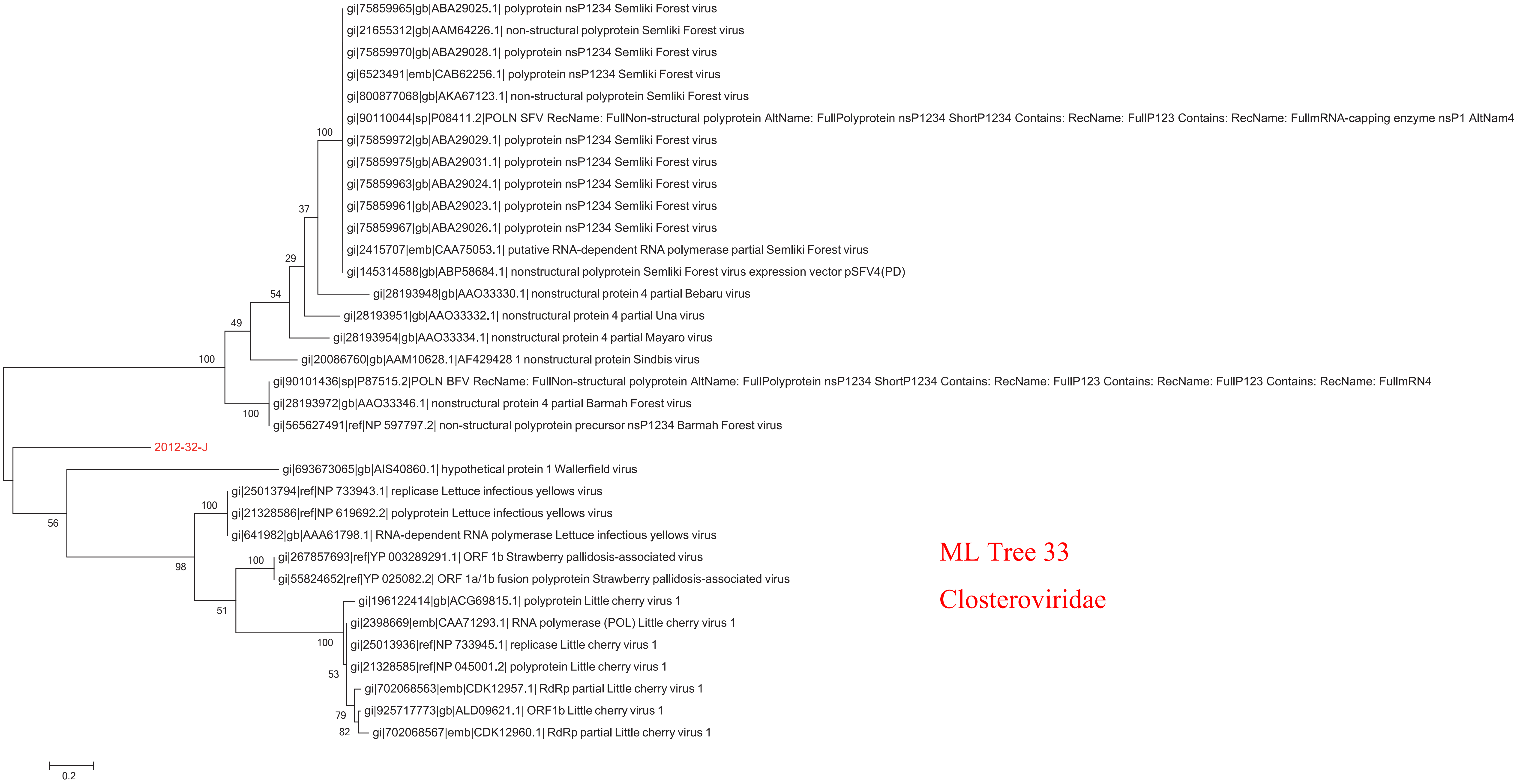

ML Tree 33

Closteroviridae

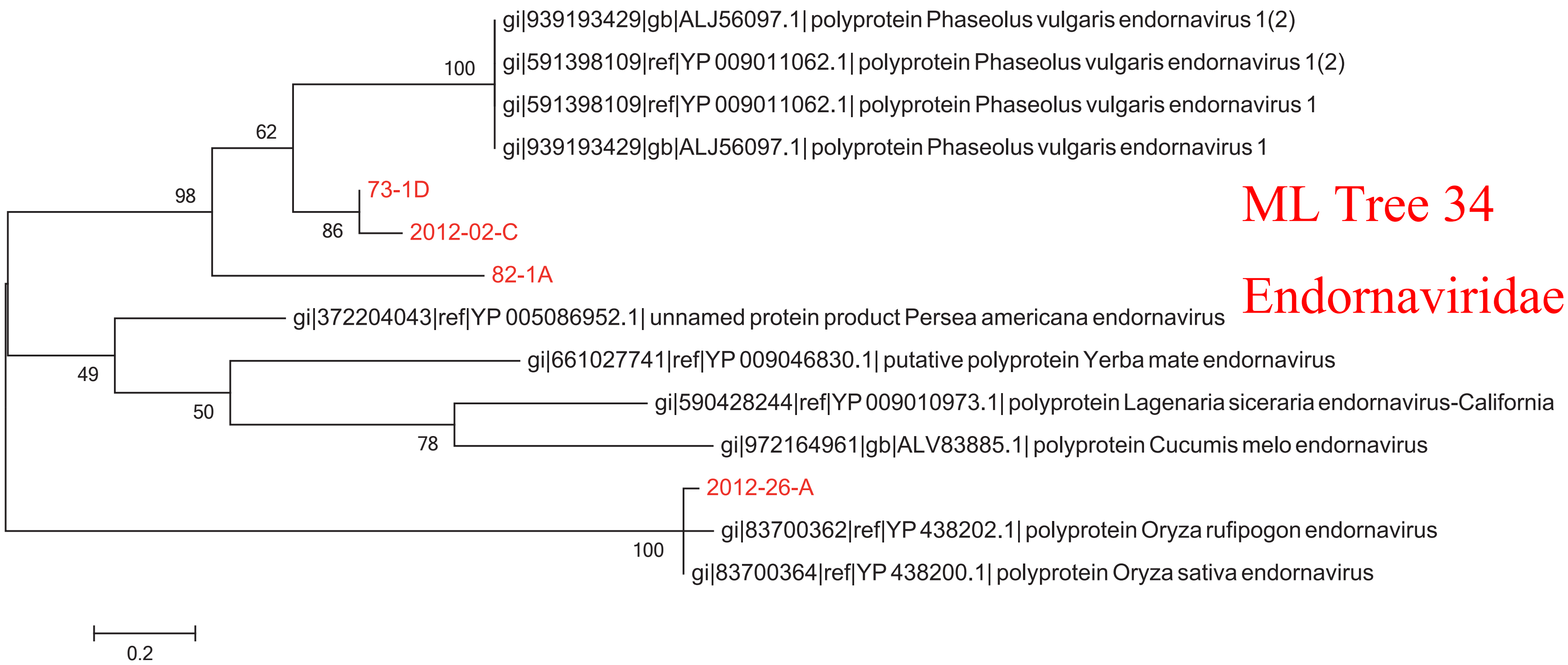
gi|939193429|gb|ALJ56097.1| polyprotein Phaseolus vulgaris endornavirus 1(2)
gi|591398109|ref|YP 009011062.1| polyprotein Phaseolus vulgaris endornavirus 1(2)
gi|591398109|ref|YP 009011062.1| polyprotein Phaseolus vulgaris endornavirus 1
gi|939193429|gb|ALJ56097.1| polyprotein Phaseolus vulgaris endornavirus 1
100
62
98
73-1D
86
2012-02-C
82-1A
gi|372204043|ref|YP 005086952.1| unnamed protein product Persea americana endornavirus
gi|661027741|ref|YP 009046830.1| putative polyprotein Yerba mate endornavirus
49
50
gi|590428244|ref|YP 009010973.1| polyprotein Lagenaria siceraria endornavirus-California
78
gi|972164961|gb|ALV83885.1| polyprotein Cucumis melo endornavirus
2012-26-A
gi|83700362|ref|YP 438202.1| polyprotein Oryza rufipogon endornavirus
100
gi|83700364|ref|YP 438200.1| polyprotein Oryza sativa endornavirus
0.2
ML Tree 34
Endornaviridae

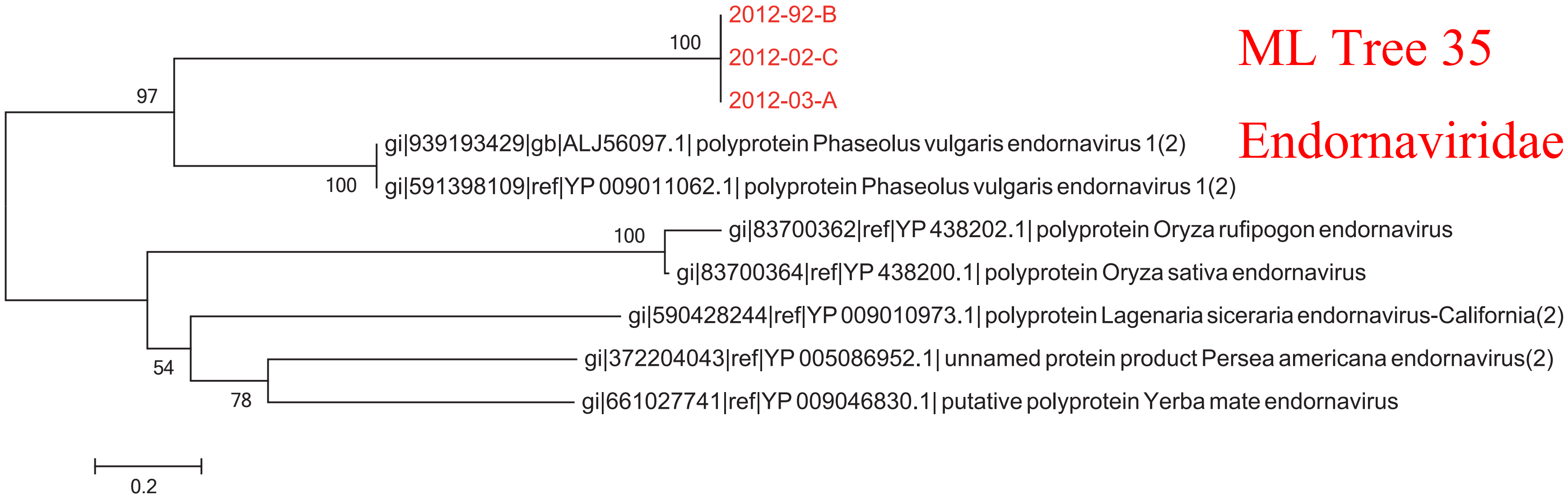
ML Tree 35
Endornaviridae
2012-92-B
2012-02-C
2012-03-A
gi|939193429|gb|ALJ56097.1| polyprotein Phaseolus vulgaris endornavirus 1(2)
gi|591398109|ref|YP 009011062.1| polyprotein Phaseolus vulgaris endornavirus 1(2)
gi|83700362|ref|YP 438202.1| polyprotein Oryza rufipogon endornavirus
gi|83700364|ref|YP 438200.1| polyprotein Oryza sativa endornavirus
gi|590428244|ref|YP 009010973.1| polyprotein Lagenaria siceraria endornavirus-California(2)
gi|372204043|ref|YP 005086952.1| unnamed protein product Persea americana endornavirus(2)
gi|661027741|ref|YP 009046830.1| putative polyprotein Yerba mate endornavirus
100
97
100
100
54
78
0.2

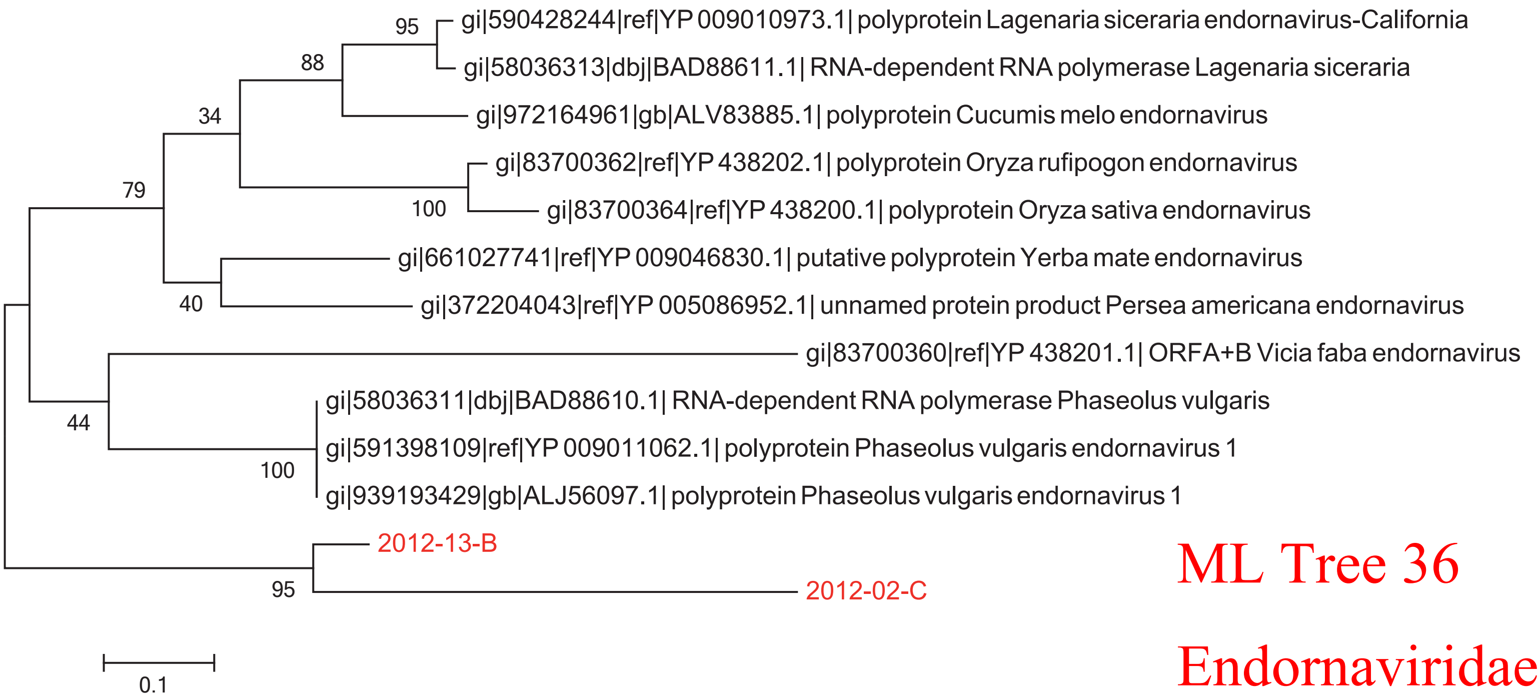

ML Tree 36

Endornaviridae

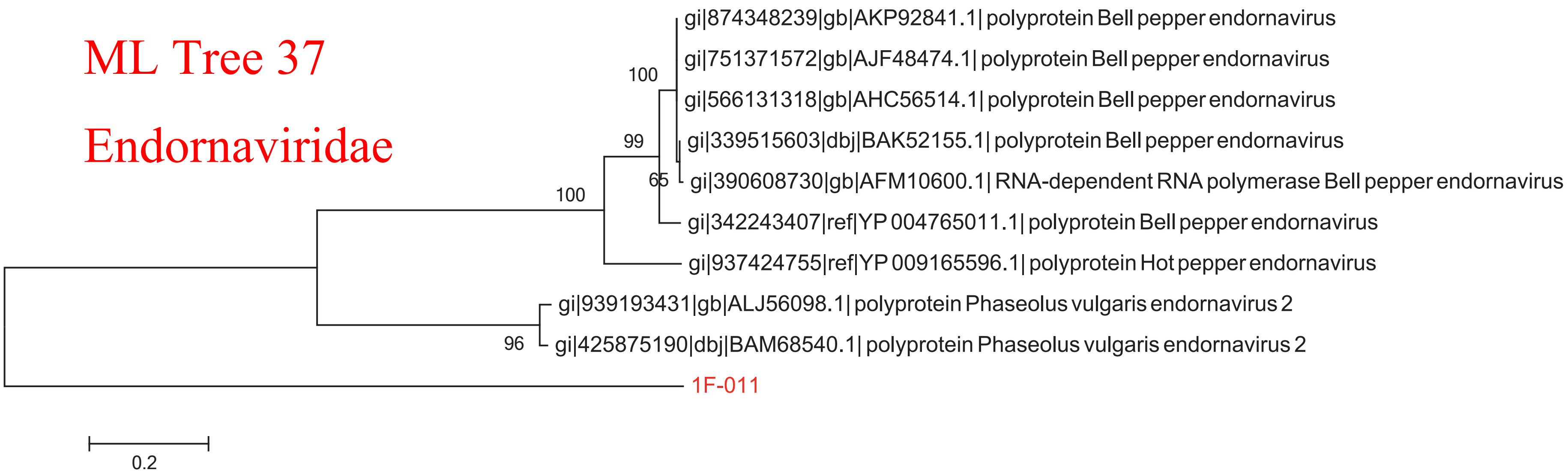
ML Tree 37
Endornaviridae
gi|874348239|gb|AKP92841.1| polyprotein Bell pepper endornavirus
gi|751371572|gb|AJF48474.1| polyprotein Bell pepper endornavirus
gi|566131318|gb|AHC56514.1| polyprotein Bell pepper endornavirus
gi|339515603|dbj|BAK52155.1| polyprotein Bell pepper endornavirus
gi|390608730|gb|AFM10600.1| RNA-dependent RNA polymerase Bell pepper endornavirus
gi|342243407|ref|YP 004765011.1| polyprotein Bell pepper endornavirus
gi|937424755|ref|YP 009165596.1| polyprotein Hot pepper endornavirus
gi|939193431|gb|ALJ56098.1| polyprotein Phaseolus vulgaris endornavirus 2
gi|425875190|dbj|BAM68540.1| polyprotein Phaseolus vulgaris endornavirus 2
1F-011
100
99
65
100
96
0.2

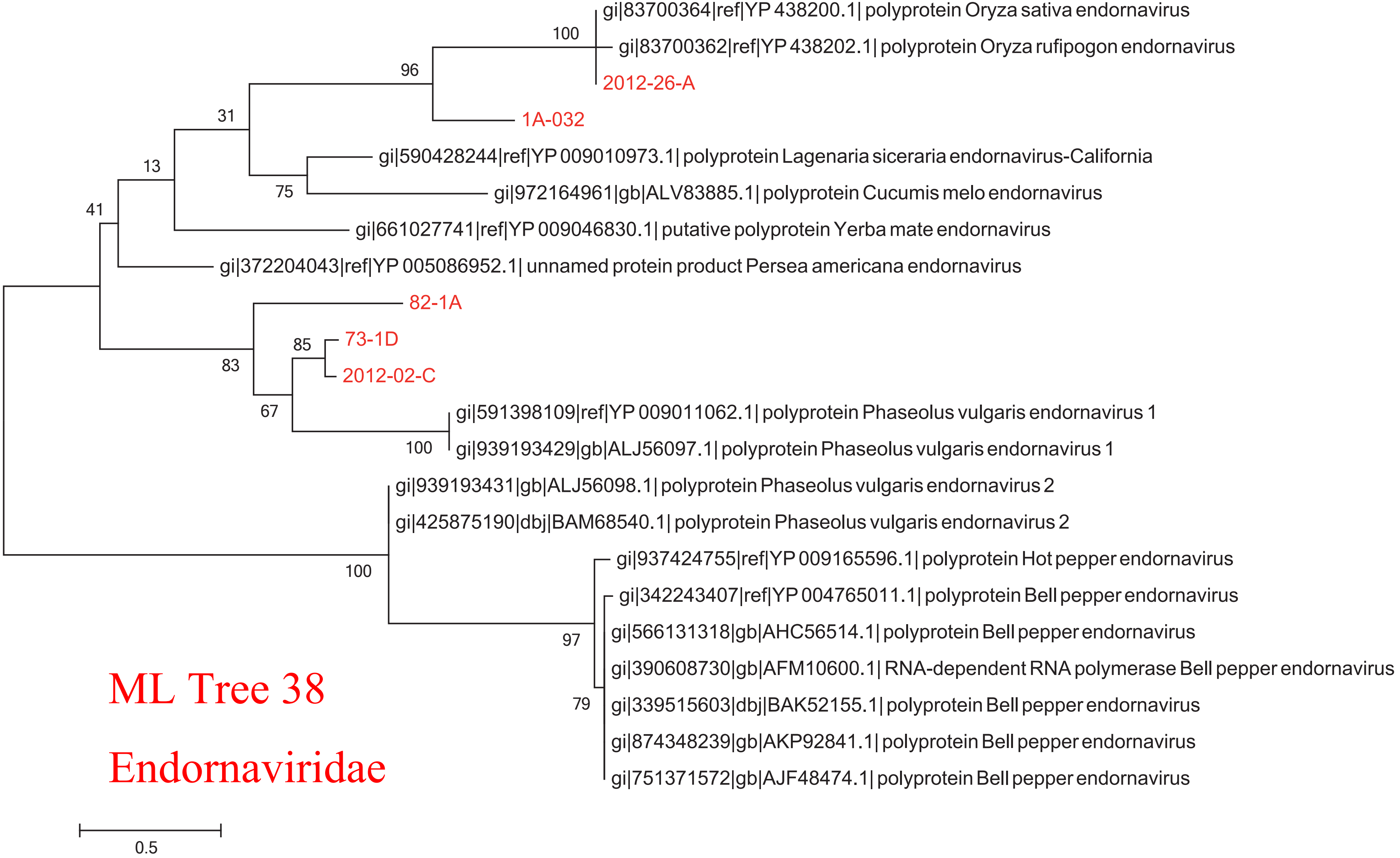

ML Tree 38

Endornaviridae

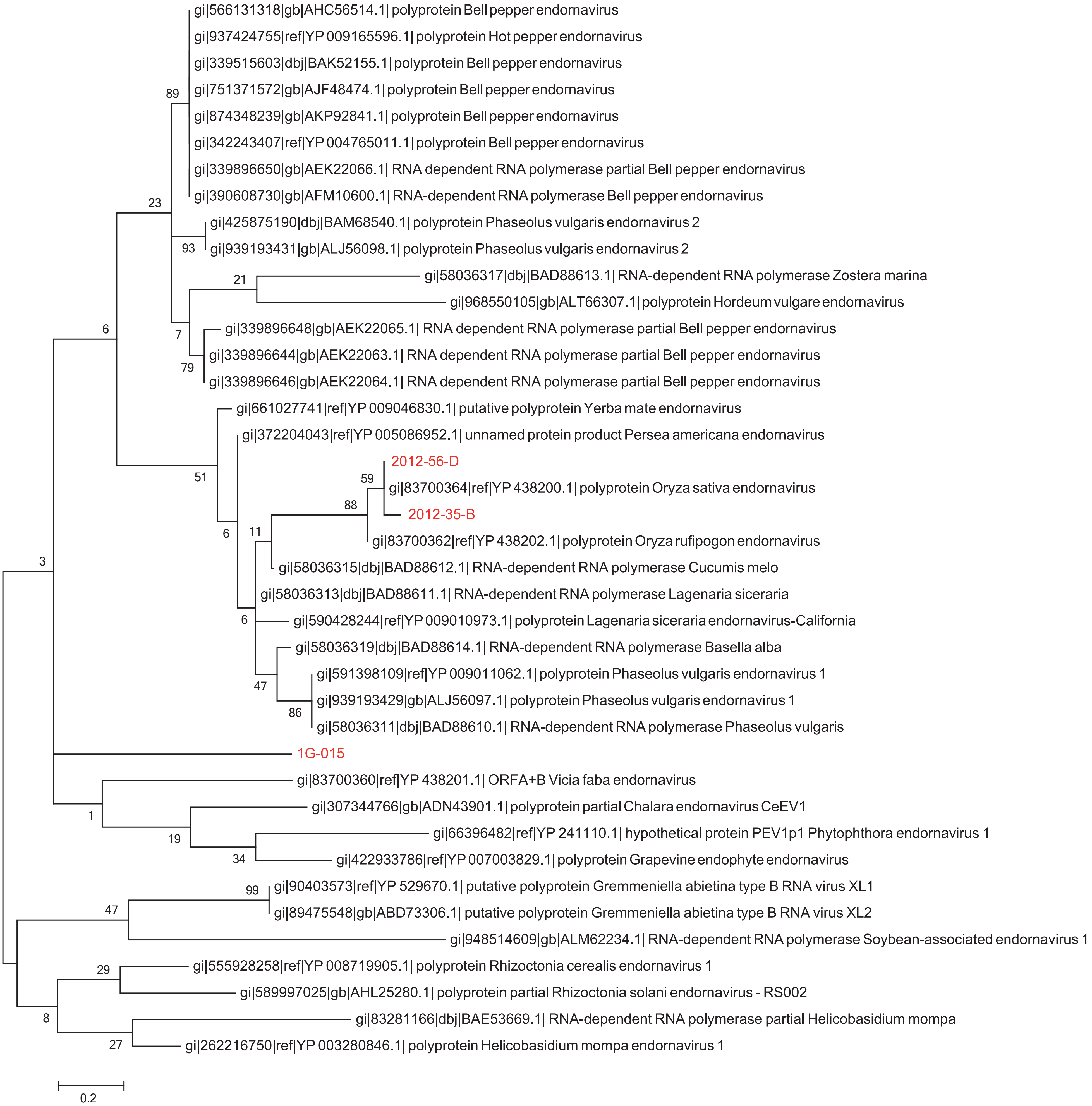

gi|566131318|gb|AHC56514.1| polyprotein Bell pepper endornavirus
gi|937424755|ref|YP 009165596.1| polyprotein Hot pepper endornavirus
gi|339515603|dbj|BAK52155.1| polyprotein Bell pepper endornavirus
gi|751371572|gb|AJF48474.1| polyprotein Bell pepper endornavirus
gi|874348239|gb|AKP92841.1| polyprotein Bell pepper endornavirus
gi|342243407|ref|YP 004765011.1| polyprotein Bell pepper endornavirus
gi|339896650|gb|AEK22066.1| RNA dependent RNA polymerase partial Bell pepper endornavirus
gi|390608730|gb|AFM10600.1| RNA-dependent RNA polymerase Bell pepper endornavirus
gi|425875190|dbj|BAM68540.1| polyprotein Phaseolus vulgaris endornavirus 2
gi|939193431|gb|ALJ56098.1| polyprotein Phaseolus vulgaris endornavirus 2
gi|58036317|dbj|BAD88613.1| RNA-dependent RNA polymerase Zostera marina
gi|968550105|gb|ALT66307.1| polyprotein Hordeum vulgare endornavirus
gi|339896648|gb|AEK22065.1| RNA dependent RNA polymerase partial Bell pepper endornavirus
gi|339896644|gb|AEK22063.1| RNA dependent RNA polymerase partial Bell pepper endornavirus
gi|339896646|gb|AEK22064.1| RNA dependent RNA polymerase partial Bell pepper endornavirus
gi|661027741|ref|YP 009046830.1| putative polyprotein Yerba mate endornavirus
gi|372204043|ref|YP 005086952.1| unnamed protein product Persea americana endornavirus
2012-56-D
gi|83700364|ref|YP 438200.1| polyprotein Oryza sativa endornavirus
2012-35-B
gi|83700362|ref|YP 438202.1| polyprotein Oryza rufipogon endornavirus
gi|58036315|dbj|BAD88612.1| RNA-dependent RNA polymerase Cucumis melo
gi|58036313|dbj|BAD88611.1| RNA-dependent RNA polymerase Lagenaria siceraria
gi|590428244|ref|YP 009010973.1| polyprotein Lagenaria siceraria endornavirus-California
gi|58036319|dbj|BAD88614.1| RNA-dependent RNA polymerase Basella alba
gi|591398109|ref|YP 009011062.1| polyprotein Phaseolus vulgaris endornavirus 1
gi|939193429|gb|ALJ56097.1| polyprotein Phaseolus vulgaris endornavirus 1
gi|58036311|dbj|BAD88610.1| RNA-dependent RNA polymerase Phaseolus vulgaris
1G-015
gi|83700360|ref|YP 438201.1| ORFA+B Vicia faba endornavirus
gi|307344766|gb|ADN43901.1| polyprotein partial Chalara endornavirus CeEV1
gi|66396482|ref|YP 241110.1| hypothetical protein PEV1p1 Phytophthora endornavirus 1
gi|422933786|ref|YP 007003829.1| polyprotein Grapevine endophyte endornavirus
gi|90403573|ref|YP 529670.1| putative polyprotein Gremmeniella abietina type B RNA virus XL1
gi|89475548|gb|ABD73306.1| putative polyprotein Gremmeniella abietina type B RNA virus XL2
gi|948514609|gb|ALM62234.1| RNA-dependent RNA polymerase Soybean-associated endornavirus 1
gi|555928258|ref|YP 008719905.1| polyprotein Rhizoctonia cerealis endornavirus 1
gi|589997025|gb|AHL25280.1| polyprotein partial Rhizoctonia solani endornavirus - RS002
gi|83281166|dbj|BAE53669.1| RNA-dependent RNA polymerase partial Helicobasidium mompa
gi|262216750|ref|YP 003280846.1| polyprotein Helicobasidium mompa endornavirus 1
89
23
93
21
7
79
6
51
59
88
6
11
3
6
47
86
1
19
34
99
47
29
8
27
0.2

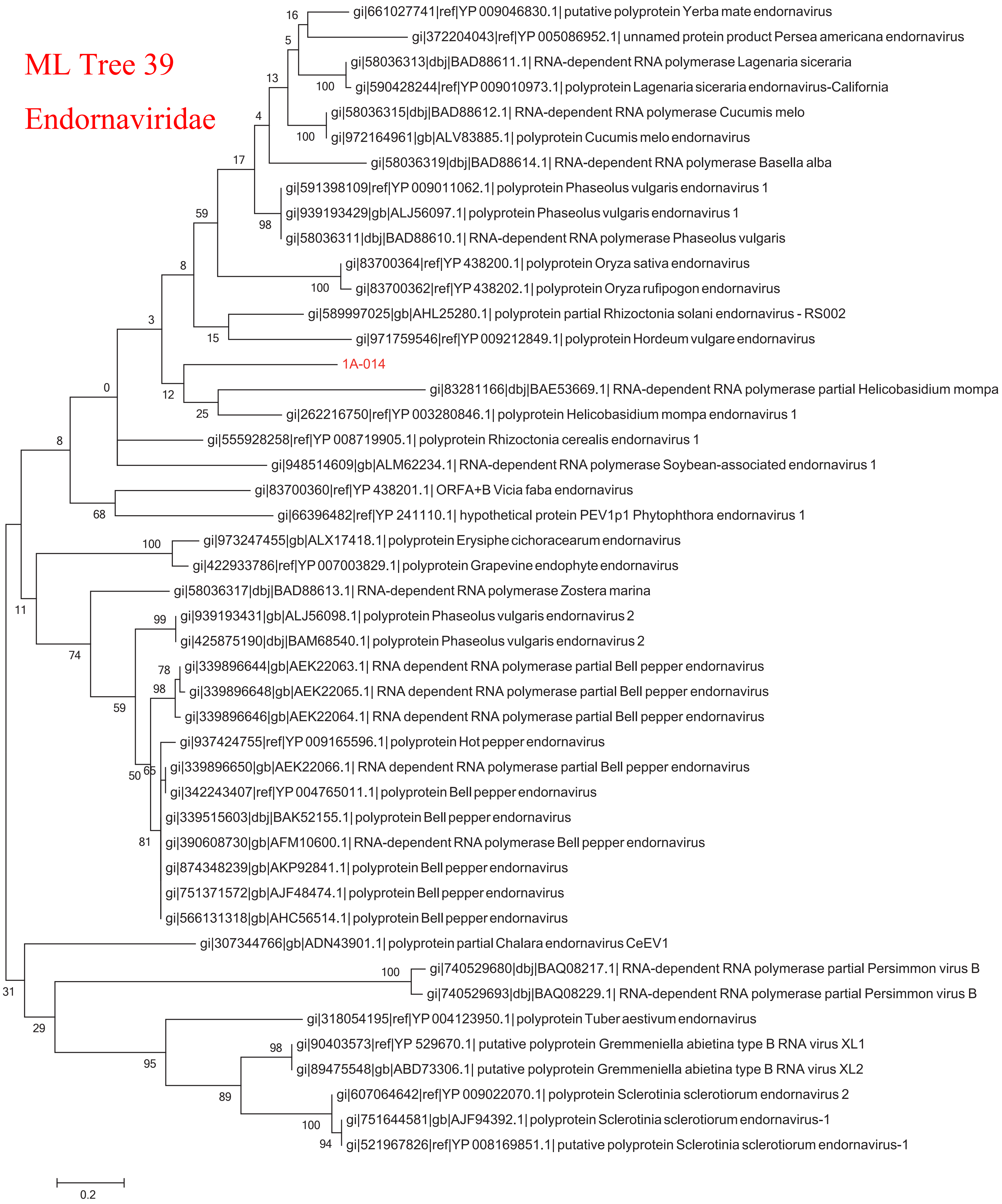
ML Tree 39
Endornaviridae
gi|661027741|ref|YP 009046830.1| putative polyprotein Yerba mate endornavirus
gi|372204043|ref|YP 005086952.1| unnamed protein product Persea americana endornavirus
gi|58036313|dbj|BAD88611.1| RNA-dependent RNA polymerase Lagenaria siceraria
gi|590428244|ref|YP 009010973.1| polyprotein Lagenaria siceraria endornavirus-California
gi|58036315|dbj|BAD88612.1| RNA-dependent RNA polymerase Cucumis melo
gi|972164961|gb|ALV83885.1| polyprotein Cucumis melo endornavirus
gi|58036319|dbj|BAD88614.1| RNA-dependent RNA polymerase Basella alba
gi|591398109|ref|YP 009011062.1| polyprotein Phaseolus vulgaris endornavirus 1
gi|939193429|gb|ALJ56097.1| polyprotein Phaseolus vulgaris endornavirus 1
gi|58036311|dbj|BAD88610.1| RNA-dependent RNA polymerase Phaseolus vulgaris
gi|83700364|ref|YP 438200.1| polyprotein Oryza sativa endornavirus
gi|83700362|ref|YP 438202.1| polyprotein Oryza rufipogon endornavirus
gi|589997025|gb|AHL25280.1| polyprotein partial Rhizoctonia solani endornavirus - RS002
gi|971759546|ref|YP 009212849.1| polyprotein Hordeum vulgare endornavirus
1A-014
gi|83281166|dbj|BAE53669.1| RNA-dependent RNA polymerase partial Helicobasidium mompa
gi|262216750|ref|YP 003280846.1| polyprotein Helicobasidium mompa endornavirus 1
gi|555928258|ref|YP 008719905.1| polyprotein Rhizoctonia cerealis endornavirus 1
gi|948514609|gb|ALM62234.1| RNA-dependent RNA polymerase Soybean-associated endornavirus 1
gi|83700360|ref|YP 438201.1| ORFA+B Vicia faba endornavirus
gi|66396482|ref|YP 241110.1| hypothetical protein PEV1p1 Phytophthora endornavirus 1
gi|973247455|gb|ALX17418.1| polyprotein Erysiphe cichoracearum endornavirus
gi|422933786|ref|YP 007003829.1| polyprotein Grapevine endophyte endornavirus
gi|58036317|dbj|BAD88613.1| RNA-dependent RNA polymerase Zostera marina
gi|939193431|gb|ALJ56098.1| polyprotein Phaseolus vulgaris endornavirus 2
gi|425875190|dbj|BAM68540.1| polyprotein Phaseolus vulgaris endornavirus 2
gi|339896644|gb|AEK22063.1| RNA dependent RNA polymerase partial Bell pepper endornavirus
gi|339896648|gb|AEK22065.1| RNA dependent RNA polymerase partial Bell pepper endornavirus
gi|339896646|gb|AEK22064.1| RNA dependent RNA polymerase partial Bell pepper endornavirus
gi|937424755|ref|YP 009165596.1| polyprotein Hot pepper endornavirus
gi|339896650|gb|AEK22066.1| RNA dependent RNA polymerase partial Bell pepper endornavirus
gi|342243407|ref|YP 004765011.1| polyprotein Bell pepper endornavirus
gi|339515603|dbj|BAK52155.1| polyprotein Bell pepper endornavirus
gi|390608730|gb|AFM10600.1| RNA-dependent RNA polymerase Bell pepper endornavirus
gi|874348239|gb|AKP92841.1| polyprotein Bell pepper endornavirus
gi|751371572|gb|AJF48474.1| polyprotein Bell pepper endornavirus
gi|566131318|gb|AHC56514.1| polyprotein Bell pepper endornavirus
gi|307344766|gb|ADN43901.1| polyprotein partial Chalara endornavirus CeEV1
gi|740529680|dbj|BAQ08217.1| RNA-dependent RNA polymerase partial Persimmon virus B
gi|740529693|dbj|BAQ08229.1| RNA-dependent RNA polymerase partial Persimmon virus B
gi|318054195|ref|YP 004123950.1| polyprotein Tuber aestivum endornavirus
gi|90403573|ref|YP 529670.1| putative polyprotein Gremmeniella abietina type B RNA virus XL1
gi|89475548|gb|ABD73306.1| putative polyprotein Gremmeniella abietina type B RNA virus XL2
gi|607064642|ref|YP 009022070.1| polyprotein Sclerotinia sclerotiorum endornavirus 2
gi|751644581|gb|AJF94392.1| polyprotein Sclerotinia sclerotiorum endornavirus-1
gi|521967826|ref|YP 008169851.1| putative polyprotein Sclerotinia sclerotiorum endornavirus-1
16
5
13
100
4
100
17
59
98
8
100
3
15
0
12
25
8
68
100
11
74
99
78
98
59
50
65
81
31
100
29
95
98
89
100
94
0.2

ML Tree 41
Endornaviridae

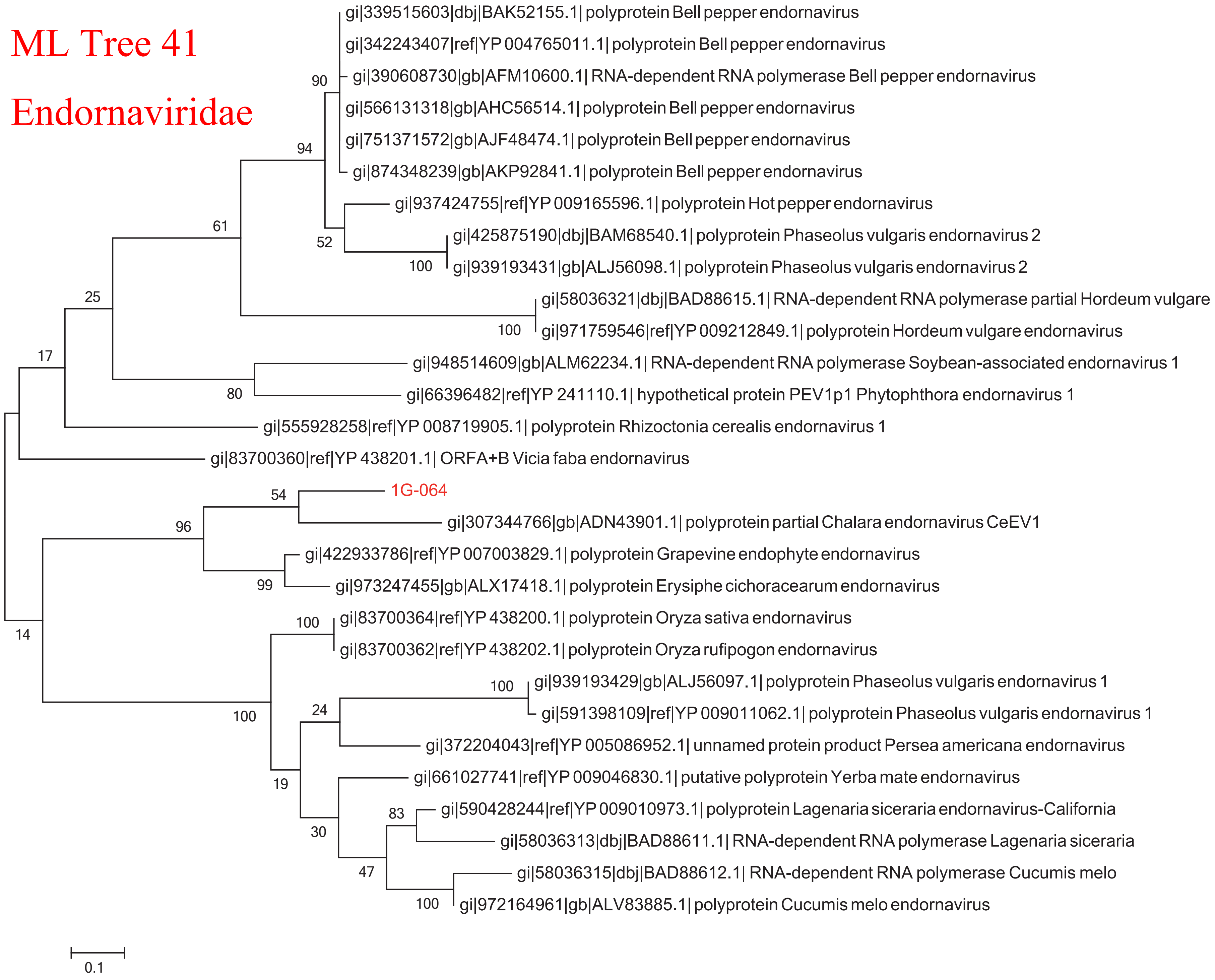

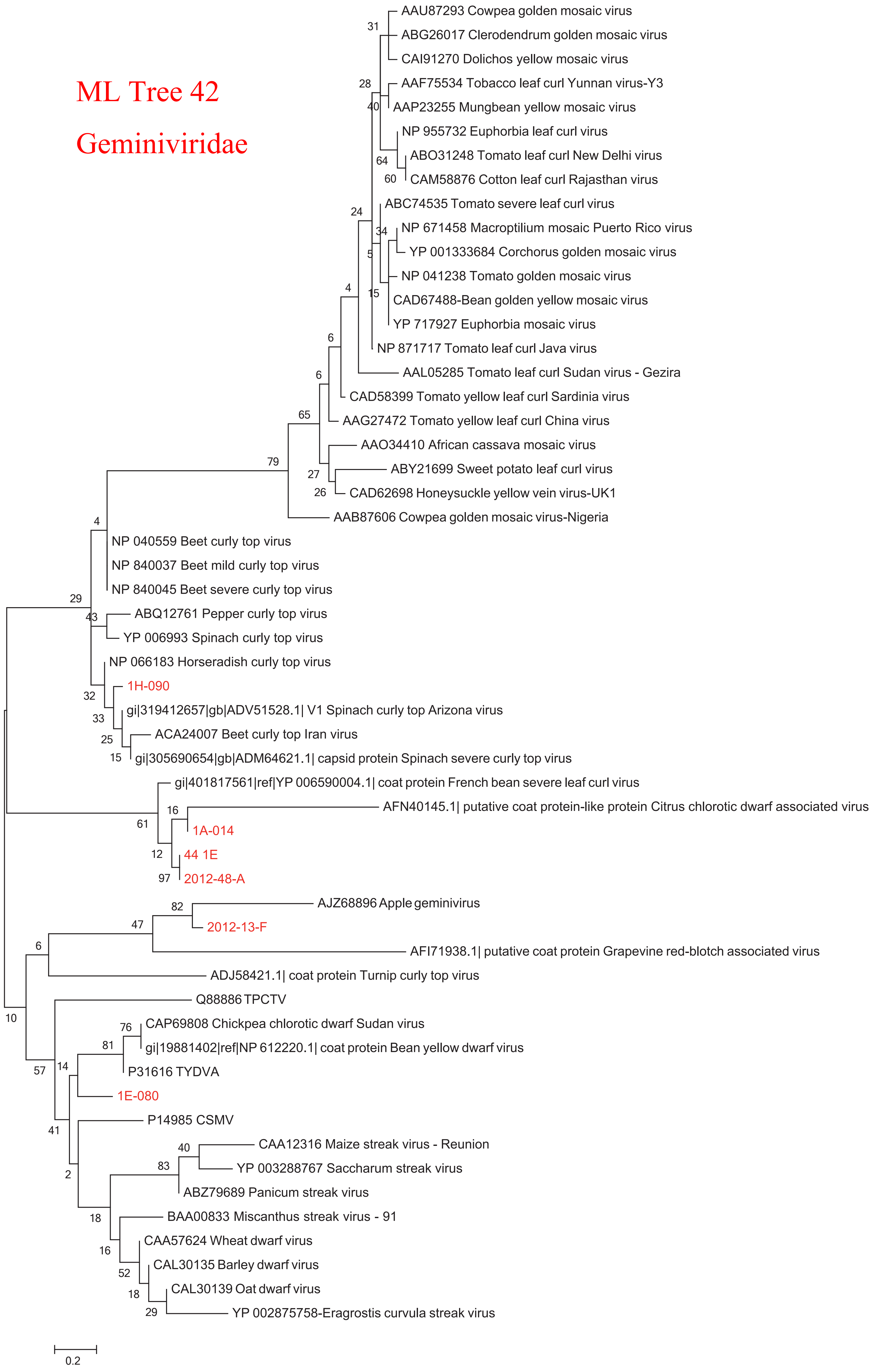
ML Tree 42
Geminiviridae
AAU87293 Cowpea golden mosaic virus
ABG26017 Clerodendrum golden mosaic virus
CAI91270 Dolichos yellow mosaic virus
AAF75534 Tobacco leaf curl Yunnan virus-Y3
AAP23255 Mungbean yellow mosaic virus
NP 955732 Euphorbia leaf curl virus
ABO31248 Tomato leaf curl New Delhi virus
CAM58876 Cotton leaf curl Rajasthan virus
ABC74535 Tomato severe leaf curl virus
NP 671458 Macroptilium mosaic Puerto Rico virus
YP 001333684 Corchorus golden mosaic virus
NP 041238 Tomato golden mosaic virus
CAD67488-Bean golden yellow mosaic virus
YP 717927 Euphorbia mosaic virus
NP 871717 Tomato leaf curl Java virus
AAL05285 Tomato leaf curl Sudan virus - Gezira
CAD58399 Tomato yellow leaf curl Sardinia virus
AAG27472 Tomato yellow leaf curl China virus
AAO34410 African cassava mosaic virus
ABY21699 Sweet potato leaf curl virus
CAD62698 Honeysuckle yellow vein virus-UK1
AAB87606 Cowpea golden mosaic virus-Nigeria
NP 040559 Beet curly top virus
NP 840037 Beet mild curly top virus
NP 840045 Beet severe curly top virus
ABQ12761 Pepper curly top virus
YP 006993 Spinach curly top virus
NP 066183 Horseradish curly top virus
1H-090
gi|319412657|gb|ADV51528.1| V1 Spinach curly top Arizona virus
ACA24007 Beet curly top Iran virus
gi|305690654|gb|ADM64621.1| capsid protein Spinach severe curly top virus
gi|401817561|ref|YP 006590004.1| coat protein French bean severe leaf curl virus
AFN40145.1| putative coat protein-like protein Citrus chlorotic dwarf associated virus
1A-014
44 1E
2012-48-A
AJZ68896 Apple geminivirus
2012-13-F
AFI71938.1| putative coat protein Grapevine red-blotch associated virus
ADJ58421.1| coat protein Turnip curly top virus
Q88886 TPCTV
CAP69808 Chickpea chlorotic dwarf Sudan virus
gi|19881402|ref|NP 612220.1| coat protein Bean yellow dwarf virus
P31616 TYDVA
1E-080
P14985 CSMV
CAA12316 Maize streak virus - Reunion
YP 003288767 Saccharum streak virus
ABZ79689 Panicum streak virus
BAA00833 Miscanthus streak virus - 91
CAA57624 Wheat dwarf virus
CAL30135 Barley dwarf virus
CAL30139 Oat dwarf virus
YP 002875758-Eragrostis curvula streak virus
0.2

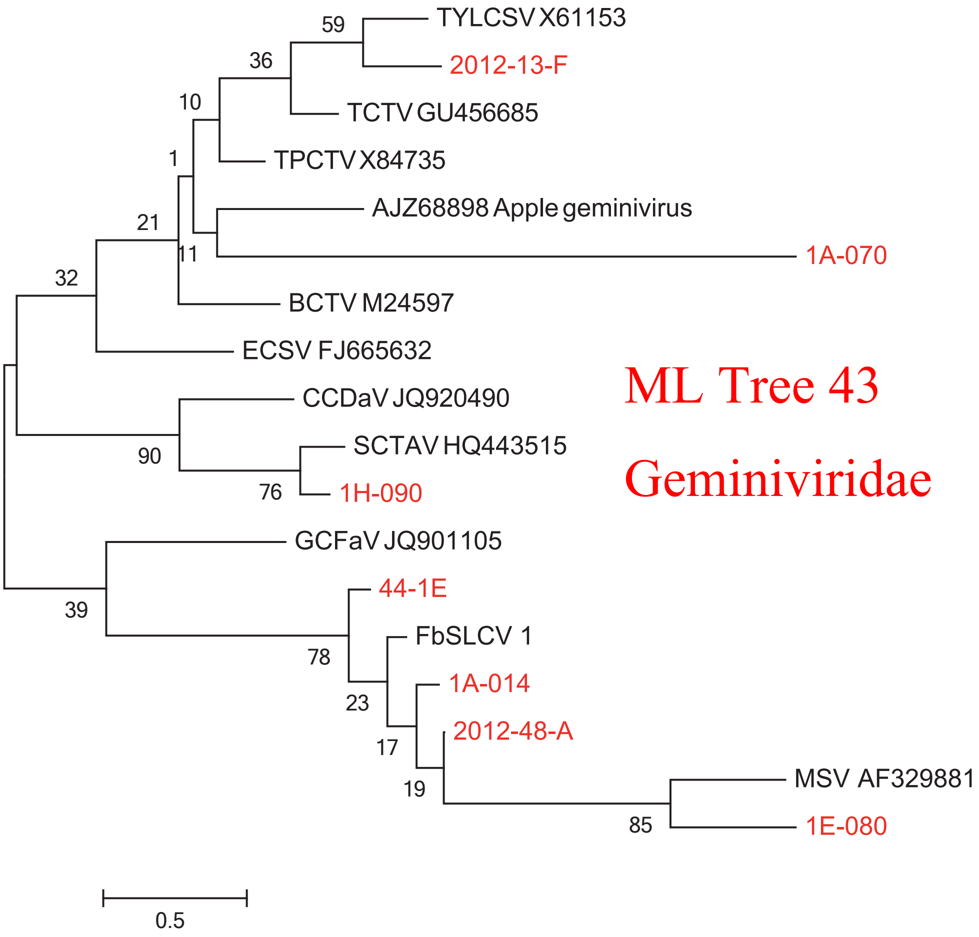

TYLCSV X61153
2012-13-F
TCTV GU456685
TPCTV X84735
AJZ68898 Apple geminivirus
1A-070
BCTV M24597
ECSV FJ665632
CCDaV JQ920490
SCTAV HQ443515
1H-090
GCFaV JQ901105
44-1E
FbSLCV 1
1A-014
2012-48-A
MSV AF329881
1E-080
59
36
10
1
21
11
32
90
76
39
78
23
17
19
85
0.5
ML Tree 43
Geminiviridae

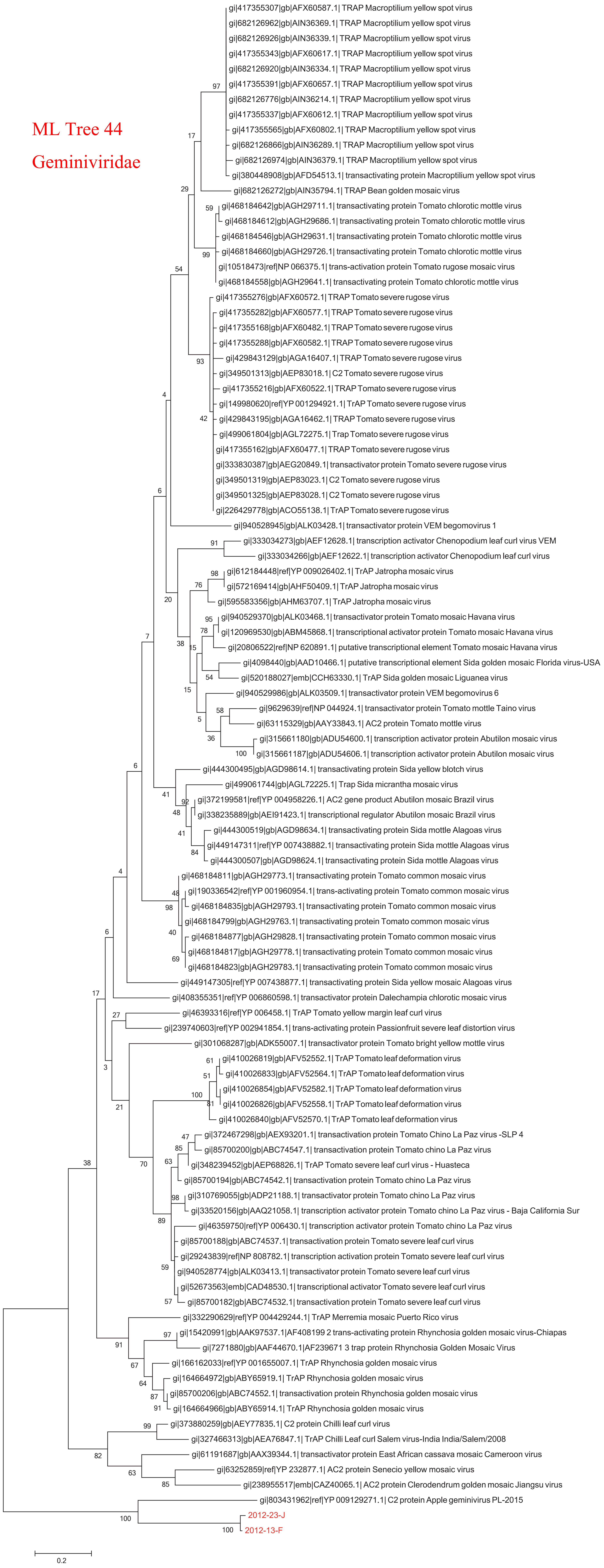

ML Tree 44
Geminiviridae
gi|417355307|gb|AFX60587.1| TRAP Macroptilium yellow spot virus
gi|682126962|gb|AIN36369.1| TRAP Macroptilium yellow spot virus
gi|682126926|gb|AIN36339.1| TRAP Macroptilium yellow spot virus
gi|417355343|gb|AFX60617.1| TRAP Macroptilium yellow spot virus
gi|682126920|gb|AIN36334.1| TRAP Macroptilium yellow spot virus
gi|417355391|gb|AFX60657.1| TRAP Macroptilium yellow spot virus
gi|682126776|gb|AIN36214.1| TRAP Macroptilium yellow spot virus
gi|417355337|gb|AFX60612.1| TRAP Macroptilium yellow spot virus
gi|417355565|gb|AFX60802.1| TRAP Macroptilium yellow spot virus
gi|682126866|gb|AIN36289.1| TRAP Macroptilium yellow spot virus
gi|682126974|gb|AIN36379.1| TRAP Macroptilium yellow spot virus
gi|380448908|gb|AFD54513.1| transactivating protein Macroptilium yellow spot virus
gi|682126272|gb|AIN35794.1| TRAP Bean golden mosaic virus
gi|468184642|gb|AGH29711.1| transactivating protein Tomato chlorotic mottle virus
gi|468184612|gb|AGH29686.1| transactivating protein Tomato chlorotic mottle virus
gi|468184546|gb|AGH29631.1| transactivating protein Tomato chlorotic mottle virus
gi|468184660|gb|AGH29726.1| transactivating protein Tomato chlorotic mottle virus
gi|10518473|ref|NP 066375.1| trans-activation protein Tomato rugose mosaic virus
gi|468184558|gb|AGH29641.1| transactivating protein Tomato chlorotic mottle virus
gi|417355276|gb|AFX60572.1| TRAP Tomato severe rugose virus
gi|417355282|gb|AFX60577.1| TRAP Tomato severe rugose virus
gi|417355168|gb|AFX60482.1| TRAP Tomato severe rugose virus
gi|417355288|gb|AFX60582.1| TRAP Tomato severe rugose virus
gi|429843129|gb|AGA16407.1| TRAP Tomato severe rugose virus
gi|349501313|gb|AEP83018.1| C2 Tomato severe rugose virus
gi|417355216|gb|AFX60522.1| TRAP Tomato severe rugose virus
gi|149980620|ref|YP 001294921.1| TrAP Tomato severe rugose virus
gi|429843195|gb|AGA16462.1| TRAP Tomato severe rugose virus
gi|499061804|gb|AGL72275.1| Trap Tomato severe rugose virus
gi|417355162|gb|AFX60477.1| TRAP Tomato severe rugose virus
gi|333830387|gb|AEG20849.1| transactivator protein Tomato severe rugose virus
gi|349501319|gb|AEP83023.1| C2 Tomato severe rugose virus
gi|349501325|gb|AEP83028.1| C2 Tomato severe rugose virus
gi|226429778|gb|ACO55138.1| TrAP Tomato severe rugose virus
gi|940528945|gb|ALK03428.1| transactivator protein VEM begomovirus 1
gi|333034273|gb|AEF12628.1| transcription activator Chenopodium leaf curl virus VEM
gi|333034266|gb|AEF12622.1| transcription activator Chenopodium leaf curl virus
gi|612184448|ref|YP 009026402.1| TrAP Jatropha mosaic virus
gi|572169414|gb|AHF50409.1| TrAP Jatropha mosaic virus
gi|595583356|gb|AHM63707.1| TrAP Jatropha mosaic virus
gi|940529370|gb|ALK03468.1| transactivator protein Tomato mosaic Havana virus
gi|120969530|gb|ABM45868.1| transcriptional activator protein Tomato mosaic Havana virus
gi|20806522|ref|NP 620891.1| putative transcriptional element Tomato mosaic Havana virus
gi|4098440|gb|AAD10466.1| putative transcriptional element Sida golden mosaic Florida virus-USA
gi|520188027|emb|CCH63330.1| TrAP Sida golden mosaic Liguanea virus
gi|940529986|gb|ALK03509.1| transactivator protein VEM begomovirus 6
gi|9629639|ref|NP 044924.1| transactivator protein Tomato mottle Taino virus
gi|63115329|gb|AAY33843.1| AC2 protein Tomato mottle virus
gi|315661180|gb|ADU54600.1| transcription activator protein Abutilon mosaic virus
gi|315661187|gb|ADU54606.1| transcription activator protein Abutilon mosaic virus
gi|444300495|gb|AGD98614.1| transactivating protein Sida yellow blotch virus
gi|499061744|gb|AGL72225.1| Trap Sida micrantha mosaic virus
gi|372199581|ref|YP 004958226.1| AC2 gene product Abutilon mosaic Brazil virus
gi|338235889|gb|AEI91423.1| transcriptional regulator Abutilon mosaic Brazil virus
gi|444300519|gb|AGD98634.1| transactivating protein Sida mottle Alagoas virus
gi|449147311|ref|YP 007438882.1| transactivating protein Sida mottle Alagoas virus
gi|444300507|gb|AGD98624.1| transactivating protein Sida mottle Alagoas virus
gi|468184811|gb|AGH29773.1| transactivating protein Tomato common mosaic virus
gi|190336542|ref|YP 001960954.1| trans-activating protein Tomato common mosaic virus
gi|468184835|gb|AGH29793.1| transactivating protein Tomato common mosaic virus
gi|468184799|gb|AGH29763.1| transactivating protein Tomato common mosaic virus
gi|468184877|gb|AGH29828.1| transactivating protein Tomato common mosaic virus
gi|468184817|gb|AGH29778.1| transactivating protein Tomato common mosaic virus
gi|468184823|gb|AGH29783.1| transactivating protein Tomato common mosaic virus
gi|449147305|ref|YP 007438877.1| transactivating protein Sida yellow mosaic Alagoas virus
gi|408355351|ref|YP 006860598.1| transactivator protein Dalechampia chlorotic mosaic virus
gi|46393316|ref|YP 006458.1| TrAP Tomato yellow margin leaf curl virus
gi|239740603|ref|YP 002941854.1| trans-activating protein Passionfruit severe leaf distortion virus
gi|301068287|gb|ADK55007.1| transactivator protein Tomato bright yellow mottle virus
gi|410026819|gb|AFV52552.1| TrAP Tomato leaf deformation virus
gi|410026833|gb|AFV52564.1| TrAP Tomato leaf deformation virus
gi|410026854|gb|AFV52582.1| TrAP Tomato leaf deformation virus
gi|410026826|gb|AFV52558.1| TrAP Tomato leaf deformation virus
gi|410026840|gb|AFV52570.1| TrAP Tomato leaf deformation virus
gi|372467298|gb|AEX93201.1| transactivation protein Tomato Chino La Paz virus -SLP 4
gi|85700200|gb|ABC74547.1| transactivation protein Tomato chino La Paz virus
gi|348239452|gb|AEP68826.1| TrAP Tomato severe leaf curl virus - Huasteca
gi|85700194|gb|ABC74542.1| transactivation protein Tomato chino La Paz virus
gi|310769055|gb|ADP21188.1| transactivator protein Tomato chino La Paz virus
gi|33520156|gb|AAQ21058.1| transcription activator protein Tomato chino La Paz virus - Baja California Sur
gi|46359750|ref|YP 006430.1| transcription activator protein Tomato chino La Paz virus
gi|85700188|gb|ABC74537.1| transactivation protein Tomato severe leaf curl virus
gi|29243839|ref|NP 808782.1| transcription activation protein Tomato severe leaf curl virus
gi|940528774|gb|ALK03413.1| transactivator protein Tomato severe leaf curl virus
gi|52673563|emb|CAD48530.1| transcriptional activator Tomato severe leaf curl virus
gi|85700182|gb|ABC74532.1| transactivation protein Tomato severe leaf curl virus
gi|332290629|ref|YP 004429244.1| TrAP Merremia mosaic Puerto Rico virus
gi|15420991|gb|AAK97537.1|AF408199 2 trans-activating protein Rhynchosia golden mosaic virus-Chiapas
gi|7271880|gb|AAF44670.1|AF239671 3 trap protein Rhynchosia Golden Mosaic Virus
gi|166162033|ref|YP 001655007.1| TrAP Rhynchosia golden mosaic virus
gi|164664972|gb|ABY65919.1| TrAP Rhynchosia golden mosaic virus
gi|85700206|gb|ABC74552.1| transactivation protein Rhynchosia golden mosaic virus
gi|164664966|gb|ABY65914.1| TrAP Rhynchosia golden mosaic virus
gi|373880259|gb|AEY77835.1| C2 protein Chilli leaf curl virus
gi|327466313|gb|AEA76847.1| TrAP Chilli Leaf curl Salem virus-India India/Salem/2008
gi|61191687|gb|AAX39344.1| transactivator protein East African cassava mosaic Cameroon virus
gi|63252859|ref|YP 232877.1| AC2 protein Senecio yellow mosaic virus
gi|238955517|emb|CAZ40065.1| AC2 protein Clerodendrum golden mosaic Jiangsu virus
gi|803431962|ref|YP 009129271.1| C2 protein Apple geminivirus PL-2015
2012-23-J
2012-13-F
0.2

ML Tree 45

Geminiviridae

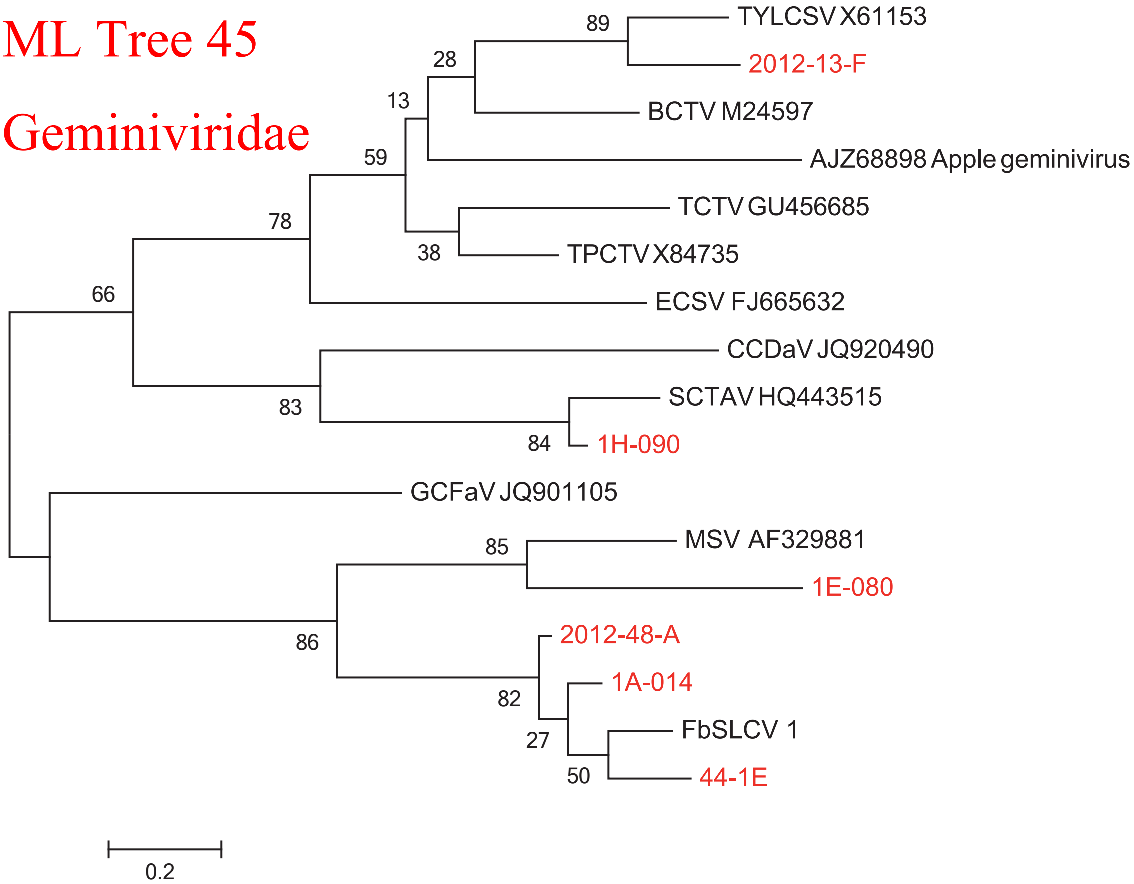

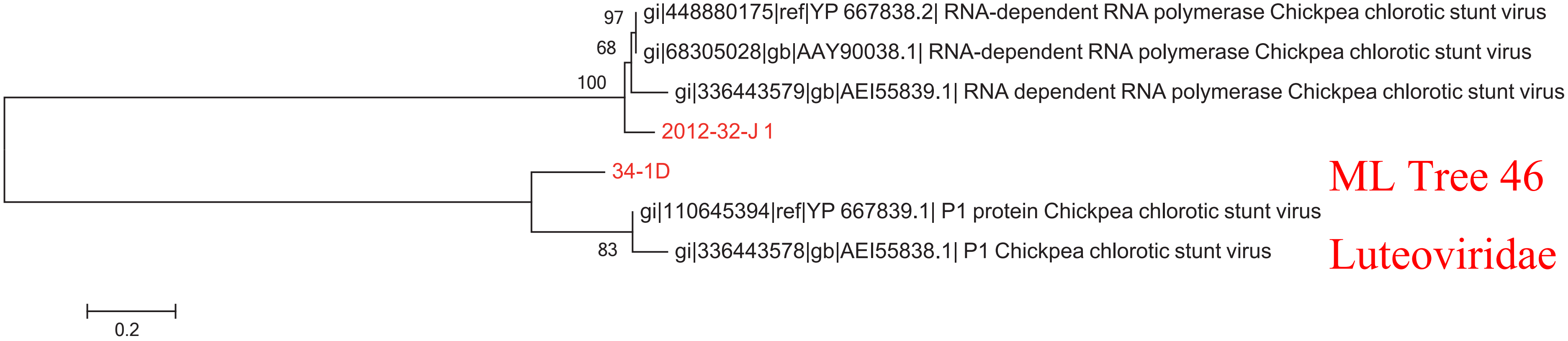

97
gi|448880175|ref|YP 667838.2| RNA-dependent RNA polymerase Chickpea chlorotic stunt virus
68
gi|68305028|gb|AAY90038.1| RNA-dependent RNA polymerase Chickpea chlorotic stunt virus
100
gi|336443579|gb|AEI55839.1| RNA dependent RNA polymerase Chickpea chlorotic stunt virus
2012-32-J 1
34-1D
gi|110645394|ref|YP 667839.1| P1 protein Chickpea chlorotic stunt virus
83
gi|336443578|gb|AEI55838.1| P1 Chickpea chlorotic stunt virus
0.2
ML Tree 46
Luteoviridae

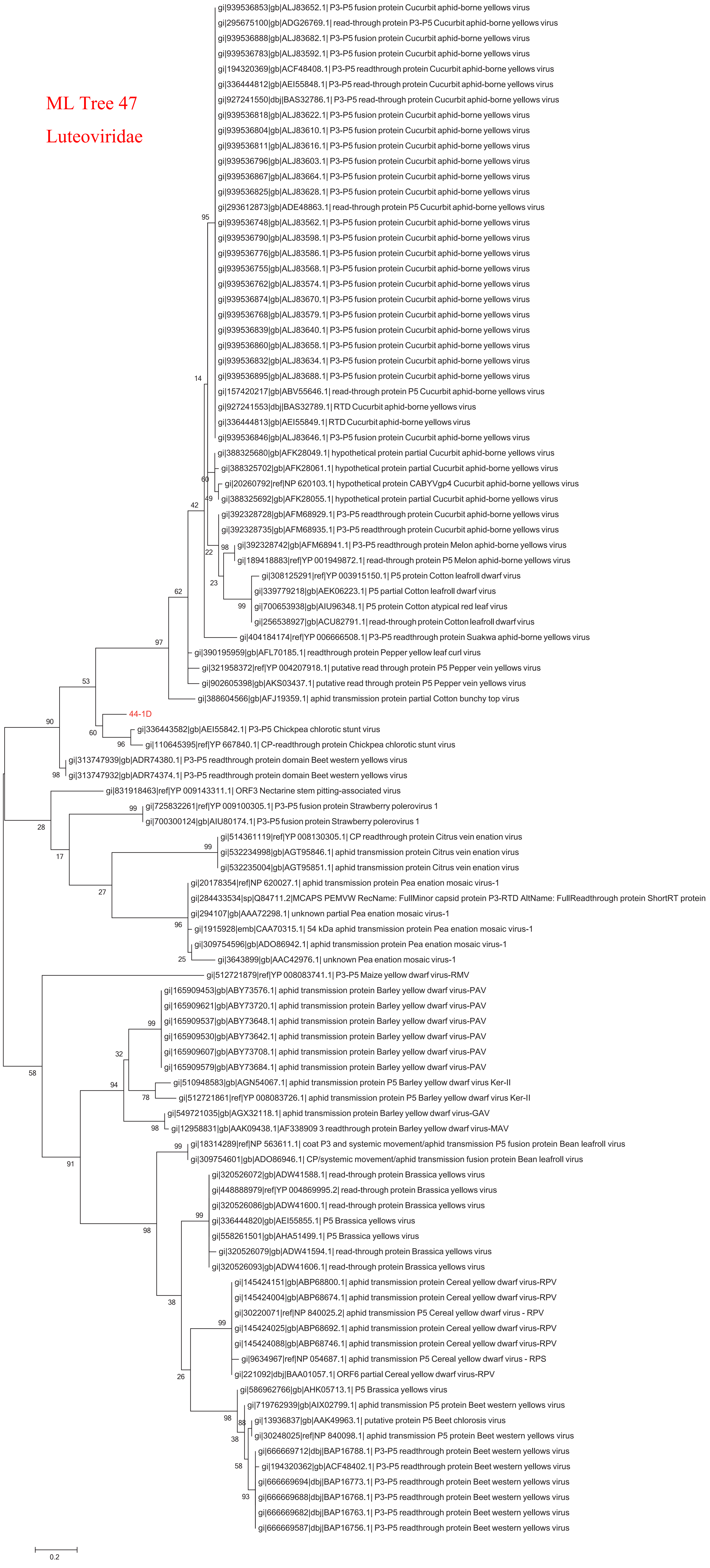
ML Tree 47
Luteoviridae
gi|939536853|gb|ALJ83652.1| P3-P5 fusion protein Cucurbit aphid-borne yellows virus
gi|295675100|gb|ADG26769.1| read-through protein P3-P5 Cucurbit aphid-borne yellows virus
gi|939536888|gb|ALJ83682.1| P3-P5 fusion protein Cucurbit aphid-borne yellows virus
gi|939536783|gb|ALJ83592.1| P3-P5 fusion protein Cucurbit aphid-borne yellows virus
gi|194320369|gb|ACF48408.1| P3-P5 readthrough protein Cucurbit aphid-borne yellows virus
gi|336444812|gb|AEI55848.1| P3-P5 read-through protein Cucurbit aphid-borne yellows virus
gi|927241550|dbj|BAS32786.1| P3-P5 read-through protein Cucurbit aphid-borne yellows virus
gi|939536818|gb|ALJ83622.1| P3-P5 fusion protein Cucurbit aphid-borne yellows virus
gi|939536804|gb|ALJ83610.1| P3-P5 fusion protein Cucurbit aphid-borne yellows virus
gi|939536811|gb|ALJ83616.1| P3-P5 fusion protein Cucurbit aphid-borne yellows virus
gi|939536796|gb|ALJ83603.1| P3-P5 fusion protein Cucurbit aphid-borne yellows virus
gi|939536867|gb|ALJ83664.1| P3-P5 fusion protein Cucurbit aphid-borne yellows virus
gi|939536825|gb|ALJ83628.1| P3-P5 fusion protein Cucurbit aphid-borne yellows virus
gi|293612873|gb|ADE48863.1| read-through protein P5 Cucurbit aphid-borne yellows virus
gi|939536748|gb|ALJ83562.1| P3-P5 fusion protein Cucurbit aphid-borne yellows virus
gi|939536790|gb|ALJ83598.1| P3-P5 fusion protein Cucurbit aphid-borne yellows virus
gi|939536776|gb|ALJ83586.1| P3-P5 fusion protein Cucurbit aphid-borne yellows virus
gi|939536755|gb|ALJ83568.1| P3-P5 fusion protein Cucurbit aphid-borne yellows virus
gi|939536762|gb|ALJ83574.1| P3-P5 fusion protein Cucurbit aphid-borne yellows virus
gi|939536874|gb|ALJ83670.1| P3-P5 fusion protein Cucurbit aphid-borne yellows virus
gi|939536768|gb|ALJ83579.1| P3-P5 fusion protein Cucurbit aphid-borne yellows virus
gi|939536839|gb|ALJ83640.1| P3-P5 fusion protein Cucurbit aphid-borne yellows virus
gi|939536860|gb|ALJ83658.1| P3-P5 fusion protein Cucurbit aphid-borne yellows virus
gi|939536832|gb|ALJ83634.1| P3-P5 fusion protein Cucurbit aphid-borne yellows virus
gi|939536895|gb|ALJ83688.1| P3-P5 fusion protein Cucurbit aphid-borne yellows virus
gi|157420217|gb|ABV55646.1| read-through protein P5 Cucurbit aphid-borne yellows virus
gi|927241553|dbj|BAS32789.1| RTD Cucurbit aphid-borne yellows virus
gi|336444813|gb|AEI55849.1| RTD Cucurbit aphid-borne yellows virus
gi|939536846|gb|ALJ83646.1| P3-P5 fusion protein Cucurbit aphid-borne yellows virus
gi|388325680|gb|AFK28049.1| hypothetical protein partial Cucurbit aphid-borne yellows virus
gi|388325702|gb|AFK28061.1| hypothetical protein partial Cucurbit aphid-borne yellows virus
gi|20260792|ref|NP 620103.1| hypothetical protein CABYVgp4 Cucurbit aphid-borne yellows virus
gi|388325692|gb|AFK28055.1| hypothetical protein partial Cucurbit aphid-borne yellows virus
gi|392328728|gb|AFM68929.1| P3-P5 readthrough protein Cucurbit aphid-borne yellows virus
gi|392328735|gb|AFM68935.1| P3-P5 readthrough protein Cucurbit aphid-borne yellows virus
gi|392328742|gb|AFM68941.1| P3-P5 readthrough protein Melon aphid-borne yellows virus
gi|189418883|ref|YP 001949872.1| read-through protein P5 Melon aphid-borne yellows virus
gi|308125291|ref|YP 003915150.1| P5 protein Cotton leafroll dwarf virus
gi|339779218|gb|AEK06223.1| P5 partial Cotton leafroll dwarf virus
gi|700653938|gb|AIU96348.1| P5 protein Cotton atypical red leaf virus
gi|256538927|gb|ACU82791.1| read-through protein Cotton leafroll dwarf virus
gi|404184174|ref|YP 006666508.1| P3-P5 readthrough protein Suakwa aphid-borne yellows virus
gi|390195959|gb|AFL70185.1| readthrough protein Pepper yellow leaf curl virus
gi|321958372|ref|YP 004207918.1| putative read through protein P5 Pepper vein yellows virus
gi|902605398|gb|AKS03437.1| putative read through protein P5 Pepper vein yellows virus
gi|388604566|gb|AFJ19359.1| aphid transmission protein partial Cotton bunchy top virus
44-1D
gi|336443582|gb|AEI55842.1| P3-P5 Chickpea chlorotic stunt virus
gi|110645395|ref|YP 667840.1| CP-readthrough protein Chickpea chlorotic stunt virus
gi|313747939|gb|ADR74380.1| P3-P5 readthrough protein domain Beet western yellows virus
gi|313747932|gb|ADR74374.1| P3-P5 readthrough protein domain Beet western yellows virus
gi|831918463|ref|YP 009143311.1| ORF3 Nectarine stem pitting-associated virus
gi|725832261|ref|YP 009100305.1| P3-P5 fusion protein Strawberry polerovirus 1
gi|700300124|gb|AIU80174.1| P3-P5 fusion protein Strawberry polerovirus 1
gi|514361119|ref|YP 008130305.1| CP readthrough protein Citrus vein enation virus
gi|532234998|gb|AGT95846.1| aphid transmission protein Citrus vein enation virus
gi|532235004|gb|AGT95851.1| aphid transmission protein Citrus vein enation virus
gi|20178354|ref|NP 620027.1| aphid transmission protein Pea enation mosaic virus-1
gi|284433534|sp|Q84711.2|MCAPS PEMVW RecName: FullMinor capsid protein P3-RTD AltName: FullReadthrough protein ShortRT protein
gi|294107|gb|AAA72298.1| unknown partial Pea enation mosaic virus-1
gi|1915928|emb|CAA70315.1| 54 kDa aphid transmission protein Pea enation mosaic virus-1
gi|309754596|gb|ADO86942.1| aphid transmission protein Pea enation mosaic virus-1
gi|3643899|gb|AAC42976.1| unknown Pea enation mosaic virus-1
gi|512721879|ref|YP 008083741.1| P3-P5 Maize yellow dwarf virus-RMV
gi|165909453|gb|ABY73576.1| aphid transmission protein Barley yellow dwarf virus-PAV
gi|165909621|gb|ABY73720.1| aphid transmission protein Barley yellow dwarf virus-PAV
gi|165909537|gb|ABY73648.1| aphid transmission protein Barley yellow dwarf virus-PAV
gi|165909530|gb|ABY73642.1| aphid transmission protein Barley yellow dwarf virus-PAV
gi|165909607|gb|ABY73708.1| aphid transmission protein Barley yellow dwarf virus-PAV
gi|165909579|gb|ABY73684.1| aphid transmission protein Barley yellow dwarf virus-PAV
gi|510948583|gb|AGN54067.1| aphid transmission protein P5 Barley yellow dwarf virus Ker-II
gi|512721861|ref|YP 008083726.1| aphid transmission protein P5 Barley yellow dwarf virus Ker-II
gi|549721035|gb|AGX32118.1| aphid transmission protein Barley yellow dwarf virus-GAV
gi|12958831|gb|AAK09438.1|AF338909 3 readthrough protein Barley yellow dwarf virus-MAV
gi|18314289|ref|NP 563611.1| coat P3 and systemic movement/aphid transmission P5 fusion protein Bean leafroll virus
gi|309754601|gb|ADO86946.1| CP/systemic movement/aphid transmission fusion protein Bean leafroll virus
gi|320526072|gb|ADW41588.1| read-through protein Brassica yellows virus
gi|448888979|ref|YP 004869995.2| read-through protein Brassica yellows virus
gi|320526086|gb|ADW41600.1| read-through protein Brassica yellows virus
gi|336444820|gb|AEI55855.1| P5 Brassica yellows virus
gi|558261501|gb|AHA51499.1| P5 Brassica yellows virus
gi|320526079|gb|ADW41594.1| read-through protein Brassica yellows virus
gi|320526093|gb|ADW41606.1| read-through protein Brassica yellows virus
gi|145424151|gb|ABP68800.1| aphid transmission protein Cereal yellow dwarf virus-RPV
gi|145424004|gb|ABP68674.1| aphid transmission protein Cereal yellow dwarf virus-RPV
gi|30220071|ref|NP 840025.2| aphid transmission P5 Cereal yellow dwarf virus - RPV
gi|145424025|gb|ABP68692.1| aphid transmission protein Cereal yellow dwarf virus-RPV
gi|145424088|gb|ABP68746.1| aphid transmission protein Cereal yellow dwarf virus-RPV
gi|9634967|ref|NP 054687.1| aphid transmission P5 Cereal yellow dwarf virus - RPS
gi|221092|dbj|BAA01057.1| ORF6 partial Cereal yellow dwarf virus-RPV
gi|586962766|gb|AHK05713.1| P5 Brassica yellows virus
gi|719762939|gb|AIX02799.1| aphid transmission P5 protein Beet western yellows virus
gi|13936837|gb|AAK49963.1| putative protein P5 Beet chlorosis virus
gi|30248025|ref|NP 840098.1| aphid transmission P5 protein Beet western yellows virus
gi|666669712|dbj|BAP16788.1| P3-P5 readthrough protein Beet western yellows virus
gi|194320362|gb|ACF48402.1| P3-P5 readthrough protein Beet western yellows virus
gi|666669694|dbj|BAP16773.1| P3-P5 readthrough protein Beet western yellows virus
gi|666669688|dbj|BAP16768.1| P3-P5 readthrough protein Beet western yellows virus
gi|666669682|dbj|BAP16763.1| P3-P5 readthrough protein Beet western yellows virus
gi|666669587|dbj|BAP16756.1| P3-P5 readthrough protein Beet western yellows virus
0.2

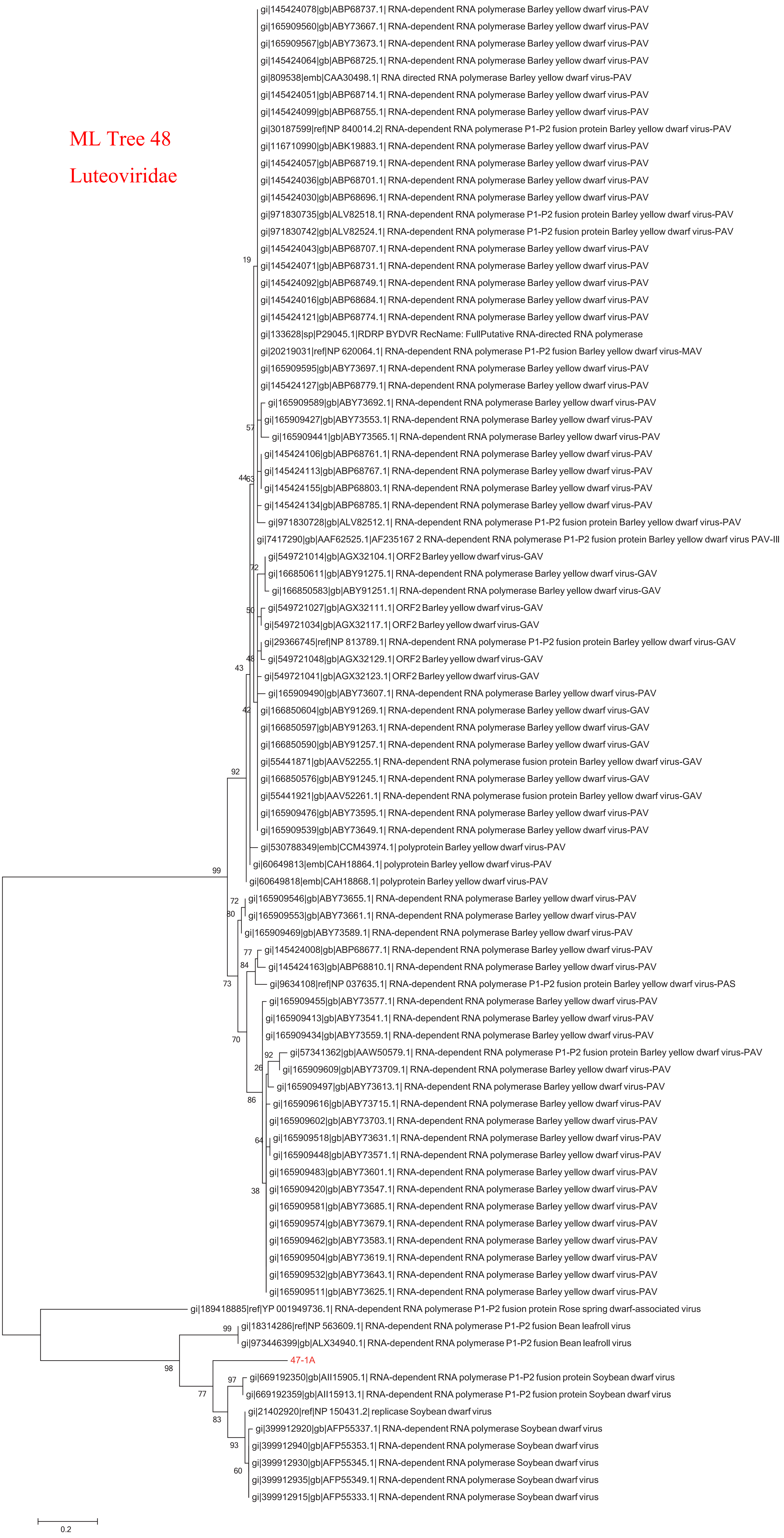
ML Tree 48
Luteoviridae
gi|145424078|gb|ABP68737.1| RNA-dependent RNA polymerase Barley yellow dwarf virus-PAV
gi|165909560|gb|ABY73667.1| RNA-dependent RNA polymerase Barley yellow dwarf virus-PAV
gi|165909567|gb|ABY73673.1| RNA-dependent RNA polymerase Barley yellow dwarf virus-PAV
gi|145424064|gb|ABP68725.1| RNA-dependent RNA polymerase Barley yellow dwarf virus-PAV
gi|809538|emb|CAA30498.1| RNA directed RNA polymerase Barley yellow dwarf virus-PAV
gi|145424051|gb|ABP68714.1| RNA-dependent RNA polymerase Barley yellow dwarf virus-PAV
gi|145424099|gb|ABP68755.1| RNA-dependent RNA polymerase Barley yellow dwarf virus-PAV
gi|30187599|ref|NP 840014.2| RNA-dependent RNA polymerase P1-P2 fusion protein Barley yellow dwarf virus-PAV
gi|116710990|gb|ABK19883.1| RNA-dependent RNA polymerase Barley yellow dwarf virus-PAV
gi|145424057|gb|ABP68719.1| RNA-dependent RNA polymerase Barley yellow dwarf virus-PAV
gi|145424036|gb|ABP68701.1| RNA-dependent RNA polymerase Barley yellow dwarf virus-PAV
gi|145424030|gb|ABP68696.1| RNA-dependent RNA polymerase Barley yellow dwarf virus-PAV
gi|971830735|gb|ALV82518.1| RNA-dependent RNA polymerase P1-P2 fusion protein Barley yellow dwarf virus-PAV
gi|971830742|gb|ALV82524.1| RNA-dependent RNA polymerase P1-P2 fusion protein Barley yellow dwarf virus-PAV
gi|145424043|gb|ABP68707.1| RNA-dependent RNA polymerase Barley yellow dwarf virus-PAV
gi|145424071|gb|ABP68731.1| RNA-dependent RNA polymerase Barley yellow dwarf virus-PAV
gi|145424092|gb|ABP68749.1| RNA-dependent RNA polymerase Barley yellow dwarf virus-PAV
gi|145424016|gb|ABP68684.1| RNA-dependent RNA polymerase Barley yellow dwarf virus-PAV
gi|145424121|gb|ABP68774.1| RNA-dependent RNA polymerase Barley yellow dwarf virus-PAV
gi|133628|sp|P29045.1|RDRP BYDVR RecName: FullPutative RNA-directed RNA polymerase
gi|20219031|ref|NP 620064.1| RNA-dependent RNA polymerase P1-P2 fusion Barley yellow dwarf virus-MAV
gi|165909595|gb|ABY73697.1| RNA-dependent RNA polymerase Barley yellow dwarf virus-PAV
gi|145424127|gb|ABP68779.1| RNA-dependent RNA polymerase Barley yellow dwarf virus-PAV
gi|165909589|gb|ABY73692.1| RNA-dependent RNA polymerase Barley yellow dwarf virus-PAV
gi|165909427|gb|ABY73553.1| RNA-dependent RNA polymerase Barley yellow dwarf virus-PAV
gi|165909441|gb|ABY73565.1| RNA-dependent RNA polymerase Barley yellow dwarf virus-PAV
gi|145424106|gb|ABP68761.1| RNA-dependent RNA polymerase Barley yellow dwarf virus-PAV
gi|145424113|gb|ABP68767.1| RNA-dependent RNA polymerase Barley yellow dwarf virus-PAV
gi|145424155|gb|ABP68803.1| RNA-dependent RNA polymerase Barley yellow dwarf virus-PAV
gi|145424134|gb|ABP68785.1| RNA-dependent RNA polymerase Barley yellow dwarf virus-PAV
gi|971830728|gb|ALV82512.1| RNA-dependent RNA polymerase P1-P2 fusion protein Barley yellow dwarf virus-PAV
gi|7417290|gb|AAF62525.1|AF235167 2 RNA-dependent RNA polymerase P1-P2 fusion protein Barley yellow dwarf virus PAV-III
gi|549721014|gb|AGX32104.1| ORF2 Barley yellow dwarf virus-GAV
gi|166850611|gb|ABY91275.1| RNA-dependent RNA polymerase Barley yellow dwarf virus-GAV
gi|166850583|gb|ABY91251.1| RNA-dependent RNA polymerase Barley yellow dwarf virus-GAV
gi|549721027|gb|AGX32111.1| ORF2 Barley yellow dwarf virus-GAV
gi|549721034|gb|AGX32117.1| ORF2 Barley yellow dwarf virus-GAV
gi|29366745|ref|NP 813789.1| RNA-dependent RNA polymerase P1-P2 fusion protein Barley yellow dwarf virus-GAV
gi|549721048|gb|AGX32129.1| ORF2 Barley yellow dwarf virus-GAV
gi|549721041|gb|AGX32123.1| ORF2 Barley yellow dwarf virus-GAV
gi|165909490|gb|ABY73607.1| RNA-dependent RNA polymerase Barley yellow dwarf virus-PAV
gi|166850604|gb|ABY91269.1| RNA-dependent RNA polymerase Barley yellow dwarf virus-GAV
gi|166850597|gb|ABY91263.1| RNA-dependent RNA polymerase Barley yellow dwarf virus-GAV
gi|166850590|gb|ABY91257.1| RNA-dependent RNA polymerase Barley yellow dwarf virus-GAV
gi|55441871|gb|AAV52255.1| RNA-dependent RNA polymerase fusion protein Barley yellow dwarf virus-GAV
gi|166850576|gb|ABY91245.1| RNA-dependent RNA polymerase Barley yellow dwarf virus-GAV
gi|55441921|gb|AAV52261.1| RNA-dependent RNA polymerase fusion protein Barley yellow dwarf virus-GAV
gi|165909476|gb|ABY73595.1| RNA-dependent RNA polymerase Barley yellow dwarf virus-PAV
gi|165909539|gb|ABY73649.1| RNA-dependent RNA polymerase Barley yellow dwarf virus-PAV
gi|530788349|emb|CCM43974.1| polyprotein Barley yellow dwarf virus-PAV
gi|60649813|emb|CAH18864.1| polyprotein Barley yellow dwarf virus-PAV
gi|60649818|emb|CAH18868.1| polyprotein Barley yellow dwarf virus-PAV
gi|165909546|gb|ABY73655.1| RNA-dependent RNA polymerase Barley yellow dwarf virus-PAV
gi|165909553|gb|ABY73661.1| RNA-dependent RNA polymerase Barley yellow dwarf virus-PAV
gi|165909469|gb|ABY73589.1| RNA-dependent RNA polymerase Barley yellow dwarf virus-PAV
gi|145424008|gb|ABP68677.1| RNA-dependent RNA polymerase Barley yellow dwarf virus-PAV
gi|145424163|gb|ABP68810.1| RNA-dependent RNA polymerase Barley yellow dwarf virus-PAV
gi|9634108|ref|NP 037635.1| RNA-dependent RNA polymerase P1-P2 fusion protein Barley yellow dwarf virus-PAS
gi|165909455|gb|ABY73577.1| RNA-dependent RNA polymerase Barley yellow dwarf virus-PAV
gi|165909413|gb|ABY73541.1| RNA-dependent RNA polymerase Barley yellow dwarf virus-PAV
gi|165909434|gb|ABY73559.1| RNA-dependent RNA polymerase Barley yellow dwarf virus-PAV
gi|57341362|gb|AAW50579.1| RNA-dependent RNA polymerase P1-P2 fusion protein Barley yellow dwarf virus-PAV
gi|165909609|gb|ABY73709.1| RNA-dependent RNA polymerase Barley yellow dwarf virus-PAV
gi|165909497|gb|ABY73613.1| RNA-dependent RNA polymerase Barley yellow dwarf virus-PAV
gi|165909616|gb|ABY73715.1| RNA-dependent RNA polymerase Barley yellow dwarf virus-PAV
gi|165909602|gb|ABY73703.1| RNA-dependent RNA polymerase Barley yellow dwarf virus-PAV
gi|165909518|gb|ABY73631.1| RNA-dependent RNA polymerase Barley yellow dwarf virus-PAV
gi|165909448|gb|ABY73571.1| RNA-dependent RNA polymerase Barley yellow dwarf virus-PAV
gi|165909483|gb|ABY73601.1| RNA-dependent RNA polymerase Barley yellow dwarf virus-PAV
gi|165909420|gb|ABY73547.1| RNA-dependent RNA polymerase Barley yellow dwarf virus-PAV
gi|165909581|gb|ABY73685.1| RNA-dependent RNA polymerase Barley yellow dwarf virus-PAV
gi|165909574|gb|ABY73679.1| RNA-dependent RNA polymerase Barley yellow dwarf virus-PAV
gi|165909462|gb|ABY73583.1| RNA-dependent RNA polymerase Barley yellow dwarf virus-PAV
gi|165909504|gb|ABY73619.1| RNA-dependent RNA polymerase Barley yellow dwarf virus-PAV
gi|165909532|gb|ABY73643.1| RNA-dependent RNA polymerase Barley yellow dwarf virus-PAV
gi|165909511|gb|ABY73625.1| RNA-dependent RNA polymerase Barley yellow dwarf virus-PAV
gi|189418885|ref|YP 001949736.1| RNA-dependent RNA polymerase P1-P2 fusion protein Rose spring dwarf-associated virus
gi|18314286|ref|NP 563609.1| RNA-dependent RNA polymerase P1-P2 fusion Bean leafroll virus
gi|973446399|gb|ALX34940.1| RNA-dependent RNA polymerase P1-P2 fusion Bean leafroll virus
47-1A
gi|669192350|gb|AII15905.1| RNA-dependent RNA polymerase P1-P2 fusion protein Soybean dwarf virus
gi|669192359|gb|AII15913.1| RNA-dependent RNA polymerase P1-P2 fusion protein Soybean dwarf virus
gi|21402920|ref|NP 150431.2| replicase Soybean dwarf virus
gi|399912920|gb|AFP55337.1| RNA-dependent RNA polymerase Soybean dwarf virus
gi|399912940|gb|AFP55353.1| RNA-dependent RNA polymerase Soybean dwarf virus
gi|399912930|gb|AFP55345.1| RNA-dependent RNA polymerase Soybean dwarf virus
gi|399912935|gb|AFP55349.1| RNA-dependent RNA polymerase Soybean dwarf virus
gi|399912915|gb|AFP55333.1| RNA-dependent RNA polymerase Soybean dwarf virus
0.2

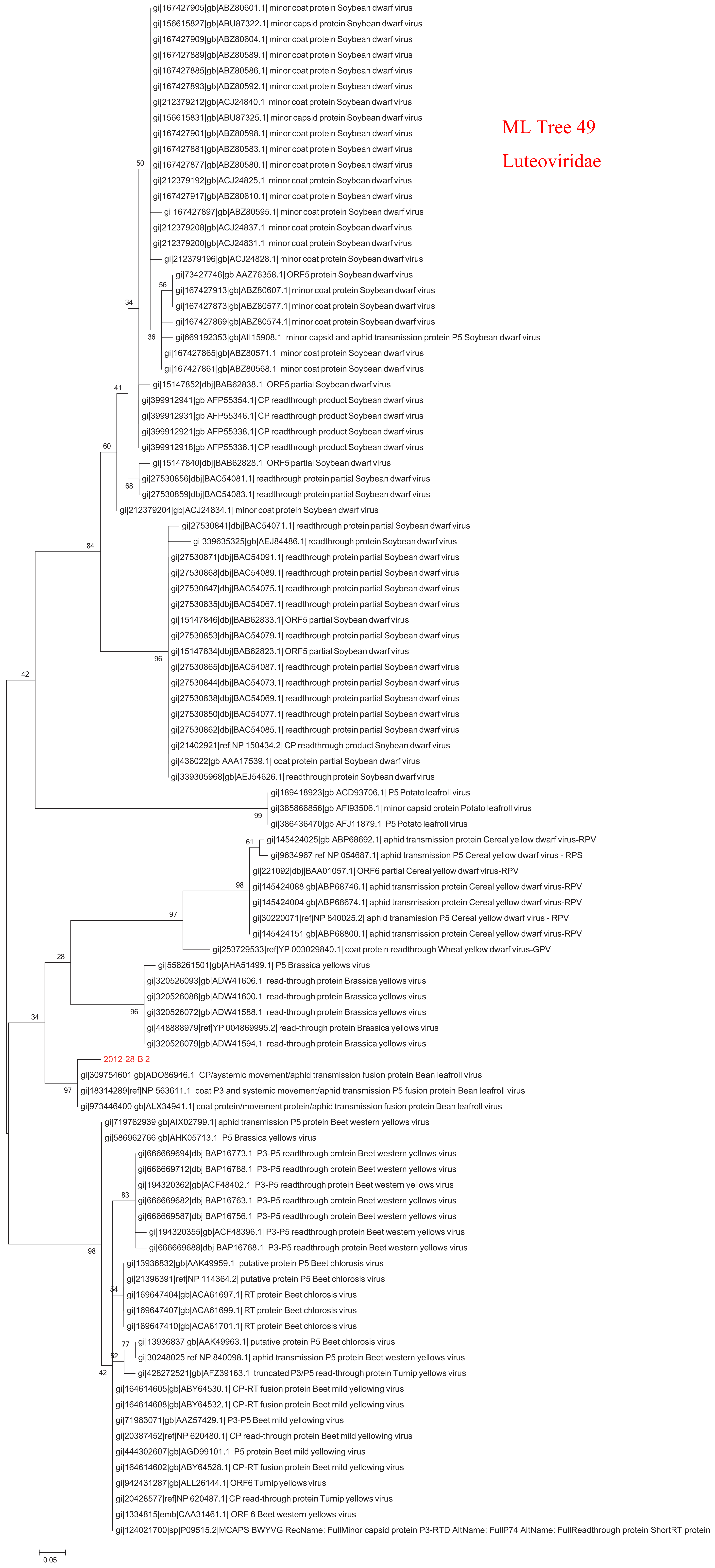

gi|167427905|gb|ABZ80601.1| minor coat protein Soybean dwarf virus
gi|156615827|gb|ABU87322.1| minor capsid protein Soybean dwarf virus
gi|167427909|gb|ABZ80604.1| minor coat protein Soybean dwarf virus
gi|167427889|gb|ABZ80589.1| minor coat protein Soybean dwarf virus
gi|167427885|gb|ABZ80586.1| minor coat protein Soybean dwarf virus
gi|167427893|gb|ABZ80592.1| minor coat protein Soybean dwarf virus
gi|212379212|gb|ACJ24840.1| minor coat protein Soybean dwarf virus
gi|156615831|gb|ABU87325.1| minor capsid protein Soybean dwarf virus
ML Tree 49
gi|167427901|gb|ABZ80598.1| minor coat protein Soybean dwarf virus
gi|167427881|gb|ABZ80583.1| minor coat protein Soybean dwarf virus
Luteoviridae
50
gi|167427877|gb|ABZ80580.1| minor coat protein Soybean dwarf virus
gi|212379192|gb|ACJ24825.1| minor coat protein Soybean dwarf virus
gi|167427917|gb|ABZ80610.1| minor coat protein Soybean dwarf virus
gi|167427897|gb|ABZ80595.1| minor coat protein Soybean dwarf virus
gi|212379208|gb|ACJ24837.1| minor coat protein Soybean dwarf virus
gi|212379200|gb|ACJ24831.1| minor coat protein Soybean dwarf virus
gi|212379196|gb|ACJ24828.1| minor coat protein Soybean dwarf virus
gi|73427746|gb|AAZ76358.1| ORF5 protein Soybean dwarf virus
56
gi|167427913|gb|ABZ80607.1| minor coat protein Soybean dwarf virus
34
gi|167427873|gb|ABZ80577.1| minor coat protein Soybean dwarf virus
gi|167427869|gb|ABZ80574.1| minor coat protein Soybean dwarf virus
36
gi|669192353|gb|AII15908.1| minor capsid and aphid transmission protein P5 Soybean dwarf virus
gi|167427865|gb|ABZ80571.1| minor coat protein Soybean dwarf virus
gi|167427861|gb|ABZ80568.1| minor coat protein Soybean dwarf virus
gi|15147852|dbj|BAB62838.1| ORF5 partial Soybean dwarf virus
41
gi|399912941|gb|AFP55354.1| CP readthrough product Soybean dwarf virus
gi|399912931|gb|AFP55346.1| CP readthrough product Soybean dwarf virus
gi|399912921|gb|AFP55338.1| CP readthrough product Soybean dwarf virus
60
gi|399912918|gb|AFP55336.1| CP readthrough product Soybean dwarf virus
gi|15147840|dbj|BAB62828.1| ORF5 partial Soybean dwarf virus
gi|27530856|dbj|BAC54081.1| readthrough protein partial Soybean dwarf virus
68
gi|27530859|dbj|BAC54083.1| readthrough protein partial Soybean dwarf virus
gi|212379204|gb|ACJ24834.1| minor coat protein Soybean dwarf virus
gi|27530841|dbj|BAC54071.1| readthrough protein partial Soybean dwarf virus
gi|339635325|gb|AEJ84486.1| readthrough protein Soybean dwarf virus
84
gi|27530871|dbj|BAC54091.1| readthrough protein partial Soybean dwarf virus
gi|27530868|dbj|BAC54089.1| readthrough protein partial Soybean dwarf virus
gi|27530847|dbj|BAC54075.1| readthrough protein partial Soybean dwarf virus
gi|27530835|dbj|BAC54067.1| readthrough protein partial Soybean dwarf virus
gi|15147846|dbj|BAB62833.1| ORF5 partial Soybean dwarf virus
gi|27530853|dbj|BAC54079.1| readthrough protein partial Soybean dwarf virus
gi|15147834|dbj|BAB62823.1| ORF5 partial Soybean dwarf virus
96
gi|27530865|dbj|BAC54087.1| readthrough protein partial Soybean dwarf virus
42
gi|27530844|dbj|BAC54073.1| readthrough protein partial Soybean dwarf virus
gi|27530838|dbj|BAC54069.1| readthrough protein partial Soybean dwarf virus
gi|27530850|dbj|BAC54077.1| readthrough protein partial Soybean dwarf virus
gi|27530862|dbj|BAC54085.1| readthrough protein partial Soybean dwarf virus
gi|21402921|ref|NP 150434.2| CP readthrough product Soybean dwarf virus
gi|436022|gb|AAA17539.1| coat protein partial Soybean dwarf virus
gi|339305968|gb|AEJ54626.1| readthrough protein Soybean dwarf virus
gi|189418923|gb|ACD93706.1| P5 Potato leafroll virus
gi|385866856|gb|AFI93506.1| minor capsid protein Potato leafroll virus
99
gi|386436470|gb|AFJ11879.1| P5 Potato leafroll virus
61
gi|145424025|gb|ABP68692.1| aphid transmission protein Cereal yellow dwarf virus-RPV
gi|9634967|ref|NP 054687.1| aphid transmission P5 Cereal yellow dwarf virus - RPS
gi|221092|dbj|BAA01057.1| ORF6 partial Cereal yellow dwarf virus-RPV
98
gi|145424088|gb|ABP68746.1| aphid transmission protein Cereal yellow dwarf virus-RPV
gi|145424004|gb|ABP68674.1| aphid transmission protein Cereal yellow dwarf virus-RPV
97
gi|30220071|ref|NP 840025.2| aphid transmission P5 Cereal yellow dwarf virus - RPV
gi|145424151|gb|ABP68800.1| aphid transmission protein Cereal yellow dwarf virus-RPV
gi|253729533|ref|YP 003029840.1| coat protein readthrough Wheat yellow dwarf virus-GPV
28
gi|558261501|gb|AHA51499.1| P5 Brassica yellows virus
gi|320526093|gb|ADW41606.1| read-through protein Brassica yellows virus
gi|320526086|gb|ADW41600.1| read-through protein Brassica yellows virus
96
gi|320526072|gb|ADW41588.1| read-through protein Brassica yellows virus
34
gi|448888979|ref|YP 004869995.2| read-through protein Brassica yellows virus
gi|320526079|gb|ADW41594.1| read-through protein Brassica yellows virus
2012-28-B 2
gi|309754601|gb|ADO86946.1| CP/systemic movement/aphid transmission fusion protein Bean leafroll virus
97
gi|18314289|ref|NP 563611.1| coat P3 and systemic movement/aphid transmission P5 fusion protein Bean leafroll virus
gi|973446400|gb|ALX34941.1| coat protein/movement protein/aphid transmission fusion protein Bean leafroll virus
gi|719762939|gb|AIX02799.1| aphid transmission P5 protein Beet western yellows virus
gi|586962766|gb|AHK05713.1| P5 Brassica yellows virus
gi|666669694|dbj|BAP16773.1| P3-P5 readthrough protein Beet western yellows virus
gi|666669712|dbj|BAP16788.1| P3-P5 readthrough protein Beet western yellows virus
gi|194320362|gb|ACF48402.1| P3-P5 readthrough protein Beet western yellows virus
83
gi|666669682|dbj|BAP16763.1| P3-P5 readthrough protein Beet western yellows virus
gi|666669587|dbj|BAP16756.1| P3-P5 readthrough protein Beet western yellows virus
gi|194320355|gb|ACF48396.1| P3-P5 readthrough protein Beet western yellows virus
gi|666669688|dbj|BAP16768.1| P3-P5 readthrough protein Beet western yellows virus
98
gi|13936832|gb|AAK49959.1| putative protein P5 Beet chlorosis virus
gi|21396391|ref|NP 114364.2| putative protein P5 Beet chlorosis virus
54
gi|169647404|gb|ACA61697.1| RT protein Beet chlorosis virus
gi|169647407|gb|ACA61699.1| RT protein Beet chlorosis virus
gi|169647410|gb|ACA61701.1| RT protein Beet chlorosis virus
77
gi|13936837|gb|AAK49963.1| putative protein P5 Beet chlorosis virus
52
gi|30248025|ref|NP 840098.1| aphid transmission P5 protein Beet western yellows virus
42
gi|428272521|gb|AFZ39163.1| truncated P3/P5 read-through protein Turnip yellows virus
gi|164614605|gb|ABY64530.1| CP-RT fusion protein Beet mild yellowing virus
gi|164614608|gb|ABY64532.1| CP-RT fusion protein Beet mild yellowing virus
gi|71983071|gb|AAZ57429.1| P3-P5 Beet mild yellowing virus
gi|20387452|ref|NP 620480.1| CP read-through protein Beet mild yellowing virus
gi|444302607|gb|AGD99101.1| P5 protein Beet mild yellowing virus
gi|164614602|gb|ABY64528.1| CP-RT fusion protein Beet mild yellowing virus
gi|942431287|gb|ALL26144.1| ORF6 Turnip yellows virus
gi|20428577|ref|NP 620487.1| CP read-through protein Turnip yellows virus
gi|1334815|emb|CAA31461.1| ORF 6 Beet western yellows virus
gi|124021700|sp|P09515.2|MCAPS BWYVG RecName: FullMinor capsid protein P3-RTD AltName: FullP74 AltName: FullReadthrough protein ShortRT protein
0.05

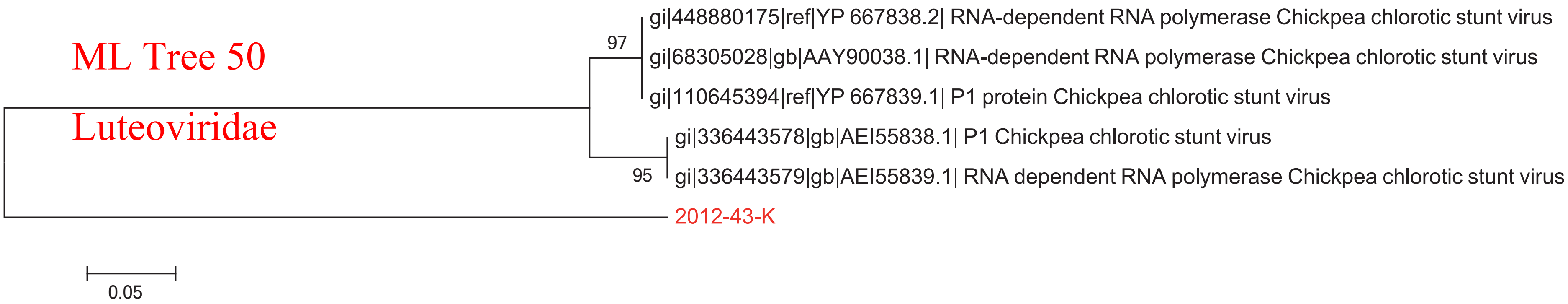
ML Tree 50
Luteoviridae
97
gi|448880175|ref|YP 667838.2| RNA-dependent RNA polymerase Chickpea chlorotic stunt virus
gi|68305028|gb|AAY90038.1| RNA-dependent RNA polymerase Chickpea chlorotic stunt virus
gi|110645394|ref|YP 667839.1| P1 protein Chickpea chlorotic stunt virus
gi|336443578|gb|AEI55838.1| P1 Chickpea chlorotic stunt virus
95
gi|336443579|gb|AEI55839.1| RNA dependent RNA polymerase Chickpea chlorotic stunt virus
2012-43-K
0.05

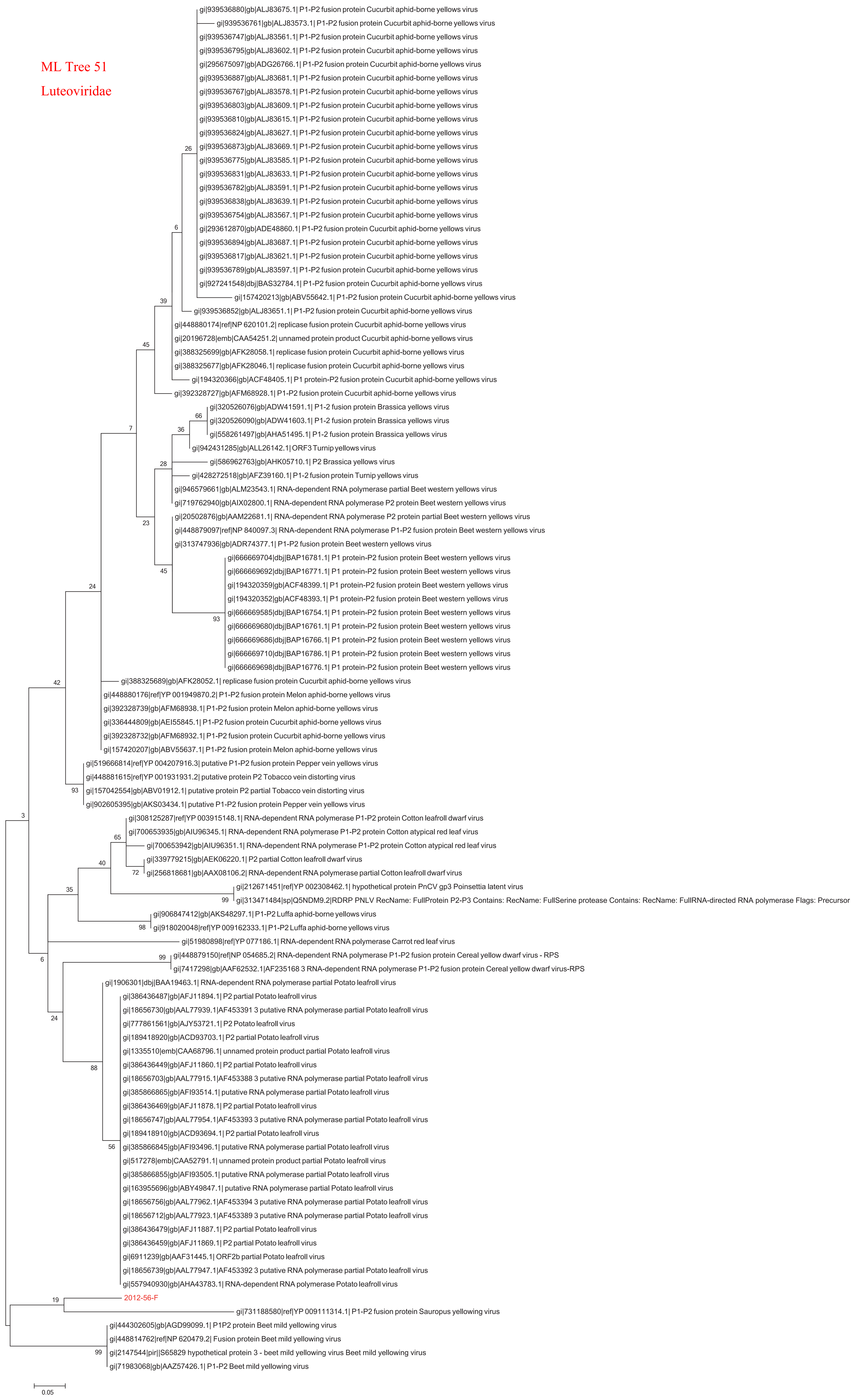
ML Tree 51
Luteoviridae
gi|939536880|gb|ALJ83675.1| P1-P2 fusion protein Cucurbit aphid-borne yellows virus
gi|939536761|gb|ALJ83573.1| P1-P2 fusion protein Cucurbit aphid-borne yellows virus
gi|939536747|gb|ALJ83561.1| P1-P2 fusion protein Cucurbit aphid-borne yellows virus
gi|939536795|gb|ALJ83602.1| P1-P2 fusion protein Cucurbit aphid-borne yellows virus
gi|295675097|gb|ADG26766.1| P1-P2 fusion protein Cucurbit aphid-borne yellows virus
gi|939536887|gb|ALJ83681.1| P1-P2 fusion protein Cucurbit aphid-borne yellows virus
gi|939536767|gb|ALJ83578.1| P1-P2 fusion protein Cucurbit aphid-borne yellows virus
gi|939536803|gb|ALJ83609.1| P1-P2 fusion protein Cucurbit aphid-borne yellows virus
gi|939536810|gb|ALJ83615.1| P1-P2 fusion protein Cucurbit aphid-borne yellows virus
gi|939536824|gb|ALJ83627.1| P1-P2 fusion protein Cucurbit aphid-borne yellows virus
gi|939536873|gb|ALJ83669.1| P1-P2 fusion protein Cucurbit aphid-borne yellows virus
gi|939536775|gb|ALJ83585.1| P1-P2 fusion protein Cucurbit aphid-borne yellows virus
gi|939536831|gb|ALJ83633.1| P1-P2 fusion protein Cucurbit aphid-borne yellows virus
gi|939536782|gb|ALJ83591.1| P1-P2 fusion protein Cucurbit aphid-borne yellows virus
gi|939536838|gb|ALJ83639.1| P1-P2 fusion protein Cucurbit aphid-borne yellows virus
gi|939536754|gb|ALJ83567.1| P1-P2 fusion protein Cucurbit aphid-borne yellows virus
gi|293612870|gb|ADE48860.1| P1-P2 fusion protein Cucurbit aphid-borne yellows virus
gi|939536894|gb|ALJ83687.1| P1-P2 fusion protein Cucurbit aphid-borne yellows virus
gi|939536817|gb|ALJ83621.1| P1-P2 fusion protein Cucurbit aphid-borne yellows virus
gi|939536789|gb|ALJ83597.1| P1-P2 fusion protein Cucurbit aphid-borne yellows virus
gi|927241548|dbj|BAS32784.1| P1-P2 fusion protein Cucurbit aphid-borne yellows virus
gi|157420213|gb|ABV55642.1| P1-P2 fusion protein Cucurbit aphid-borne yellows virus
gi|939536852|gb|ALJ83651.1| P1-P2 fusion protein Cucurbit aphid-borne yellows virus
gi|448880174|ref|NP 620101.2| replicase fusion protein Cucurbit aphid-borne yellows virus
gi|20196728|emb|CAA54251.2| unnamed protein product Cucurbit aphid-borne yellows virus
gi|388325699|gb|AFK28058.1| replicase fusion protein Cucurbit aphid-borne yellows virus
gi|388325677|gb|AFK28046.1| replicase fusion protein Cucurbit aphid-borne yellows virus
gi|194320366|gb|ACF48405.1| P1 protein-P2 fusion protein Cucurbit aphid-borne yellows virus
gi|392328727|gb|AFM68928.1| P1-P2 fusion protein Cucurbit aphid-borne yellows virus
gi|320526076|gb|ADW41591.1| P1-2 fusion protein Brassica yellows virus
gi|320526090|gb|ADW41603.1| P1-2 fusion protein Brassica yellows virus
gi|558261497|gb|AHA51495.1| P1-2 fusion protein Brassica yellows virus
gi|942431285|gb|ALL26142.1| ORF3 Turnip yellows virus
gi|586962763|gb|AHK05710.1| P2 Brassica yellows virus
gi|428272518|gb|AFZ39160.1| P1-2 fusion protein Turnip yellows virus
gi|946579661|gb|ALM23543.1| RNA-dependent RNA polymerase partial Beet western yellows virus
gi|719762940|gb|AIX02800.1| RNA-dependent RNA polymerase P2 protein Beet western yellows virus
gi|20502876|gb|AAM22681.1| RNA-dependent RNA polymerase P2 protein partial Beet western yellows virus
gi|448879097|ref|NP 840097.3| RNA-dependent RNA polymerase P1-P2 fusion protein Beet western yellows virus
gi|313747936|gb|ADR74377.1| P1-P2 fusion protein Beet western yellows virus
gi|666669704|dbj|BAP16781.1| P1 protein-P2 fusion protein Beet western yellows virus
gi|666669692|dbj|BAP16771.1| P1 protein-P2 fusion protein Beet western yellows virus
gi|194320359|gb|ACF48399.1| P1 protein-P2 fusion protein Beet western yellows virus
gi|194320352|gb|ACF48393.1| P1 protein-P2 fusion protein Beet western yellows virus
gi|666669585|dbj|BAP16754.1| P1 protein-P2 fusion protein Beet western yellows virus
gi|666669680|dbj|BAP16761.1| P1 protein-P2 fusion protein Beet western yellows virus
gi|666669686|dbj|BAP16766.1| P1 protein-P2 fusion protein Beet western yellows virus
gi|666669710|dbj|BAP16786.1| P1 protein-P2 fusion protein Beet western yellows virus
gi|666669698|dbj|BAP16776.1| P1 protein-P2 fusion protein Beet western yellows virus
gi|388325689|gb|AFK28052.1| replicase fusion protein Cucurbit aphid-borne yellows virus
gi|448880176|ref|YP 001949870.2| P1-P2 fusion protein Melon aphid-borne yellows virus
gi|392328739|gb|AFM68938.1| P1-P2 fusion protein Melon aphid-borne yellows virus
gi|336444809|gb|AEI55845.1| P1-P2 fusion protein Cucurbit aphid-borne yellows virus
gi|392328732|gb|AFM68932.1| P1-P2 fusion protein Cucurbit aphid-borne yellows virus
gi|157420207|gb|ABV55637.1| P1-P2 fusion protein Melon aphid-borne yellows virus
gi|519666814|ref|YP 004207916.3| putative P1-P2 fusion protein Pepper vein yellows virus
gi|448881615|ref|YP 001931931.2| putative protein P2 Tobacco vein distorting virus
gi|157042554|gb|ABV01912.1| putative protein P2 partial Tobacco vein distorting virus
gi|902605395|gb|AKS03434.1| putative P1-P2 fusion protein Pepper vein yellows virus
gi|308125287|ref|YP 003915148.1| RNA-dependent RNA polymerase P1-P2 protein Cotton leafroll dwarf virus
gi|700653935|gb|AIU96345.1| RNA-dependent RNA polymerase P1-P2 protein Cotton atypical red leaf virus
gi|700653942|gb|AIU96351.1| RNA-dependent RNA polymerase P1-P2 protein Cotton atypical red leaf virus
gi|339779215|gb|AEK06220.1| P2 partial Cotton leafroll dwarf virus
gi|256818681|gb|AAX08106.2| RNA-dependent RNA polymerase partial Cotton leafroll dwarf virus
gi|212671451|ref|YP 002308462.1| hypothetical protein PnCV gp3 Poinsettia latent virus
gi|313471484|sp|Q5NDM9.2|RDRP PNLV RecName: FullProtein P2-P3 Contains: RecName: FullSerine protease Contains: RecName: FullRNA-directed RNA polymerase Flags: Precursor
gi|906847412|gb|AKS48297.1| P1-P2 Luffa aphid-borne yellows virus
gi|918020048|ref|YP 009162333.1| P1-P2 Luffa aphid-borne yellows virus
gi|51980898|ref|YP 077186.1| RNA-dependent RNA polymerase Carrot red leaf virus
gi|448879150|ref|NP 054685.2| RNA-dependent RNA polymerase P1-P2 fusion protein Cereal yellow dwarf virus - RPS
gi|7417298|gb|AAF62532.1|AF235168 3 RNA-dependent RNA polymerase P1-P2 fusion protein Cereal yellow dwarf virus-RPS
gi|1906301|dbj|BAA19463.1| RNA-dependent RNA polymerase partial Potato leafroll virus
gi|386436487|gb|AFJ11894.1| P2 partial Potato leafroll virus
gi|18656730|gb|AAL77939.1|AF453391 3 putative RNA polymerase partial Potato leafroll virus
gi|777861561|gb|AJY53721.1| P2 Potato leafroll virus
gi|189418920|gb|ACD93703.1| P2 partial Potato leafroll virus
gi|1335510|emb|CAA68796.1| unnamed protein product partial Potato leafroll virus
gi|386436449|gb|AFJ11860.1| P2 partial Potato leafroll virus
gi|18656703|gb|AAL77915.1|AF453388 3 putative RNA polymerase partial Potato leafroll virus
gi|385866865|gb|AFI93514.1| putative RNA polymerase partial Potato leafroll virus
gi|386436469|gb|AFJ11878.1| P2 partial Potato leafroll virus
gi|18656747|gb|AAL77954.1|AF453393 3 putative RNA polymerase partial Potato leafroll virus
gi|189418910|gb|ACD93694.1| P2 partial Potato leafroll virus
gi|385866845|gb|AFI93496.1| putative RNA polymerase partial Potato leafroll virus
gi|517278|emb|CAA52791.1| unnamed protein product partial Potato leafroll virus
gi|385866855|gb|AFI93505.1| putative RNA polymerase partial Potato leafroll virus
gi|163955696|gb|ABY49847.1| putative RNA polymerase partial Potato leafroll virus
gi|18656756|gb|AAL77962.1|AF453394 3 putative RNA polymerase partial Potato leafroll virus
gi|18656712|gb|AAL77923.1|AF453389 3 putative RNA polymerase partial Potato leafroll virus
gi|386436479|gb|AFJ11887.1| P2 partial Potato leafroll virus
gi|386436459|gb|AFJ11869.1| P2 partial Potato leafroll virus
gi|6911239|gb|AAF31445.1| ORF2b partial Potato leafroll virus
gi|18656739|gb|AAL77947.1|AF453392 3 putative RNA polymerase partial Potato leafroll virus
gi|557940930|gb|AHA43783.1| RNA-dependent RNA polymerase Potato leafroll virus
2012-56-F
gi|731188580|ref|YP 009111314.1| P1-P2 fusion protein Sauropus yellowing virus
gi|444302605|gb|AGD99099.1| P1P2 protein Beet mild yellowing virus
gi|448814762|ref|NP 620479.2| Fusion protein Beet mild yellowing virus
gi|2147544|pir||S65829 hypothetical protein 3 - beet mild yellowing virus Beet mild yellowing virus
gi|71983068|gb|AAZ57426.1| P1-P2 Beet mild yellowing virus
0.05

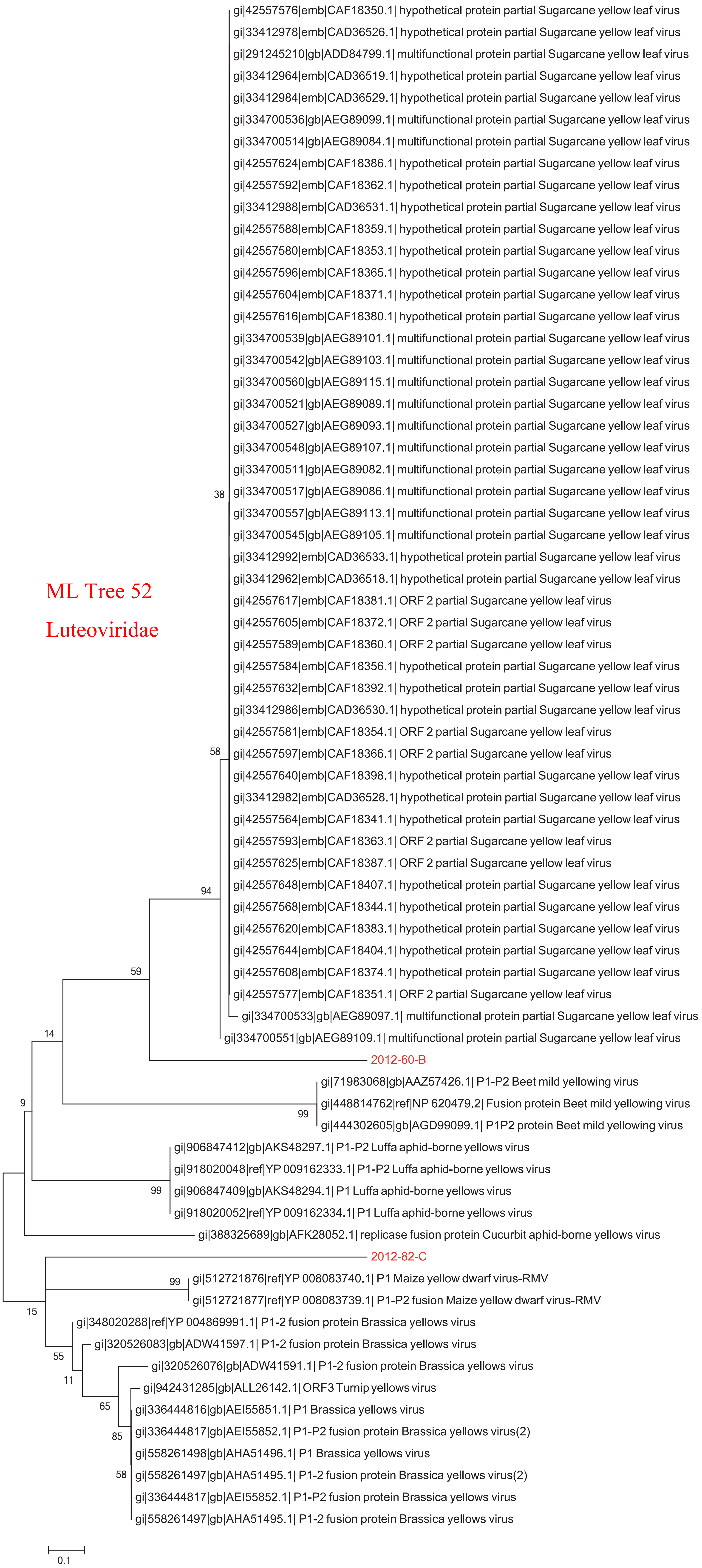

ML Tree 52
Luteoviridae
gi|42557576|emb|CAF18350.1| hypothetical protein partial Sugarcane yellow leaf virus
gi|33412978|emb|CAD36526.1| hypothetical protein partial Sugarcane yellow leaf virus
gi|291245210|gb|ADD84799.1| multifunctional protein partial Sugarcane yellow leaf virus
gi|33412964|emb|CAD36519.1| hypothetical protein partial Sugarcane yellow leaf virus
gi|33412984|emb|CAD36529.1| hypothetical protein partial Sugarcane yellow leaf virus
gi|334700536|gb|AEG89099.1| multifunctional protein partial Sugarcane yellow leaf virus
gi|334700514|gb|AEG89084.1| multifunctional protein partial Sugarcane yellow leaf virus
gi|42557624|emb|CAF18386.1| hypothetical protein partial Sugarcane yellow leaf virus
gi|42557592|emb|CAF18362.1| hypothetical protein partial Sugarcane yellow leaf virus
gi|33412988|emb|CAD36531.1| hypothetical protein partial Sugarcane yellow leaf virus
gi|42557588|emb|CAF18359.1| hypothetical protein partial Sugarcane yellow leaf virus
gi|42557580|emb|CAF18353.1| hypothetical protein partial Sugarcane yellow leaf virus
gi|42557596|emb|CAF18365.1| hypothetical protein partial Sugarcane yellow leaf virus
gi|42557604|emb|CAF18371.1| hypothetical protein partial Sugarcane yellow leaf virus
gi|42557616|emb|CAF18380.1| hypothetical protein partial Sugarcane yellow leaf virus
gi|334700539|gb|AEG89101.1| multifunctional protein partial Sugarcane yellow leaf virus
gi|334700542|gb|AEG89103.1| multifunctional protein partial Sugarcane yellow leaf virus
gi|334700560|gb|AEG89115.1| multifunctional protein partial Sugarcane yellow leaf virus
gi|334700521|gb|AEG89089.1| multifunctional protein partial Sugarcane yellow leaf virus
gi|334700527|gb|AEG89093.1| multifunctional protein partial Sugarcane yellow leaf virus
gi|334700548|gb|AEG89107.1| multifunctional protein partial Sugarcane yellow leaf virus
gi|334700511|gb|AEG89082.1| multifunctional protein partial Sugarcane yellow leaf virus
38
gi|334700517|gb|AEG89086.1| multifunctional protein partial Sugarcane yellow leaf virus
gi|334700557|gb|AEG89113.1| multifunctional protein partial Sugarcane yellow leaf virus
gi|334700545|gb|AEG89105.1| multifunctional protein partial Sugarcane yellow leaf virus
gi|33412992|emb|CAD36533.1| hypothetical protein partial Sugarcane yellow leaf virus
gi|33412962|emb|CAD36518.1| hypothetical protein partial Sugarcane yellow leaf virus
gi|42557617|emb|CAF18381.1| ORF 2 partial Sugarcane yellow leaf virus
gi|42557605|emb|CAF18372.1| ORF 2 partial Sugarcane yellow leaf virus
gi|42557589|emb|CAF18360.1| ORF 2 partial Sugarcane yellow leaf virus
gi|42557584|emb|CAF18356.1| hypothetical protein partial Sugarcane yellow leaf virus
gi|42557632|emb|CAF18392.1| hypothetical protein partial Sugarcane yellow leaf virus
gi|33412986|emb|CAD36530.1| hypothetical protein partial Sugarcane yellow leaf virus
gi|42557581|emb|CAF18354.1| ORF 2 partial Sugarcane yellow leaf virus
58
gi|42557597|emb|CAF18366.1| ORF 2 partial Sugarcane yellow leaf virus
gi|42557640|emb|CAF18398.1| hypothetical protein partial Sugarcane yellow leaf virus
gi|33412982|emb|CAD36528.1| hypothetical protein partial Sugarcane yellow leaf virus
gi|42557564|emb|CAF18341.1| hypothetical protein partial Sugarcane yellow leaf virus
gi|42557593|emb|CAF18363.1| ORF 2 partial Sugarcane yellow leaf virus
gi|42557625|emb|CAF18387.1| ORF 2 partial Sugarcane yellow leaf virus
94
gi|42557648|emb|CAF18407.1| hypothetical protein partial Sugarcane yellow leaf virus
gi|42557568|emb|CAF18344.1| hypothetical protein partial Sugarcane yellow leaf virus
gi|42557620|emb|CAF18383.1| hypothetical protein partial Sugarcane yellow leaf virus
gi|42557644|emb|CAF18404.1| hypothetical protein partial Sugarcane yellow leaf virus
59
gi|42557608|emb|CAF18374.1| hypothetical protein partial Sugarcane yellow leaf virus
gi|42557577|emb|CAF18351.1| ORF 2 partial Sugarcane yellow leaf virus
gi|334700533|gb|AEG89097.1| multifunctional protein partial Sugarcane yellow leaf virus
14
gi|334700551|gb|AEG89109.1| multifunctional protein partial Sugarcane yellow leaf virus
2012-60-B
gi|71983068|gb|AAZ57426.1| P1-P2 Beet mild yellowing virus
9
gi|448814762|ref|NP 620479.2| Fusion protein Beet mild yellowing virus
99
gi|444302605|gb|AGD99099.1| P1P2 protein Beet mild yellowing virus
gi|906847412|gb|AKS48297.1| P1-P2 Luffa aphid-borne yellows virus
gi|918020048|ref|YP 009162333.1| P1-P2 Luffa aphid-borne yellows virus
99
gi|906847409|gb|AKS48294.1| P1 Luffa aphid-borne yellows virus
gi|918020052|ref|YP 009162334.1| P1 Luffa aphid-borne yellows virus
gi|388325689|gb|AFK28052.1| replicase fusion protein Cucurbit aphid-borne yellows virus
2012-82-C
99
gi|512721876|ref|YP 008083740.1| P1 Maize yellow dwarf virus-RMV
gi|512721877|ref|YP 008083739.1| P1-P2 fusion Maize yellow dwarf virus-RMV
15
gi|348020288|ref|YP 004869991.1| P1-2 fusion protein Brassica yellows virus
gi|320526083|gb|ADW41597.1| P1-2 fusion protein Brassica yellows virus
55
11
gi|320526076|gb|ADW41591.1| P1-2 fusion protein Brassica yellows virus
gi|942431285|gb|ALL26142.1| ORF3 Turnip yellows virus
65
gi|336444816|gb|AEI55851.1| P1 Brassica yellows virus
85
gi|336444817|gb|AEI55852.1| P1-P2 fusion protein Brassica yellows virus(2)
gi|558261498|gb|AHA51496.1| P1 Brassica yellows virus
58
gi|558261497|gb|AHA51495.1| P1-2 fusion protein Brassica yellows virus(2)
gi|336444817|gb|AEI55852.1| P1-P2 fusion protein Brassica yellows virus
gi|558261497|gb|AHA51495.1| P1-2 fusion protein Brassica yellows virus
0.1

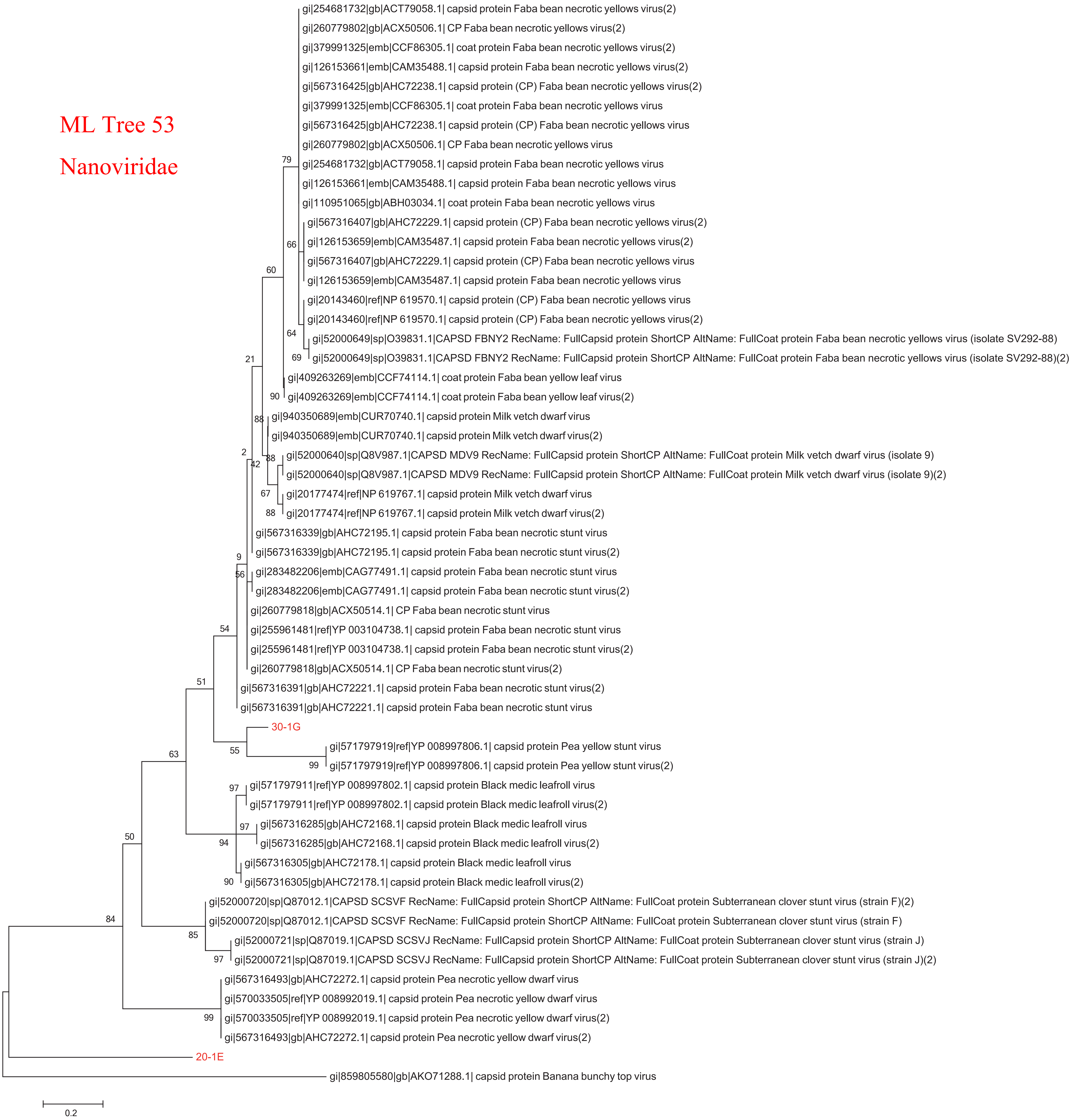
ML Tree 53
Nanoviridae
gi|254681732|gb|ACT79058.1| capsid protein Faba bean necrotic yellows virus(2)
gi|260779802|gb|ACX50506.1| CP Faba bean necrotic yellows virus(2)
gi|379991325|emb|CCF86305.1| coat protein Faba bean necrotic yellows virus(2)
gi|126153661|emb|CAM35488.1| capsid protein Faba bean necrotic yellows virus(2)
gi|567316425|gb|AHC72238.1| capsid protein (CP) Faba bean necrotic yellows virus(2)
gi|379991325|emb|CCF86305.1| coat protein Faba bean necrotic yellows virus
gi|567316425|gb|AHC72238.1| capsid protein (CP) Faba bean necrotic yellows virus
gi|260779802|gb|ACX50506.1| CP Faba bean necrotic yellows virus
gi|254681732|gb|ACT79058.1| capsid protein Faba bean necrotic yellows virus
gi|126153661|emb|CAM35488.1| capsid protein Faba bean necrotic yellows virus
gi|110951065|gb|ABH03034.1| coat protein Faba bean necrotic yellows virus
gi|567316407|gb|AHC72229.1| capsid protein (CP) Faba bean necrotic yellows virus(2)
gi|126153659|emb|CAM35487.1| capsid protein Faba bean necrotic yellows virus(2)
gi|567316407|gb|AHC72229.1| capsid protein (CP) Faba bean necrotic yellows virus
gi|126153659|emb|CAM35487.1| capsid protein Faba bean necrotic yellows virus
gi|20143460|ref|NP 619570.1| capsid protein (CP) Faba bean necrotic yellows virus
gi|20143460|ref|NP 619570.1| capsid protein (CP) Faba bean necrotic yellows virus(2)
gi|52000649|sp|O39831.1|CAPSD FBNY2 RecName: FullCapsid protein ShortCP AltName: FullCoat protein Faba bean necrotic yellows virus (isolate SV292-88)
gi|52000649|sp|O39831.1|CAPSD FBNY2 RecName: FullCapsid protein ShortCP AltName: FullCoat protein Faba bean necrotic yellows virus (isolate SV292-88)(2)
gi|409263269|emb|CCF74114.1| coat protein Faba bean yellow leaf virus
gi|409263269|emb|CCF74114.1| coat protein Faba bean yellow leaf virus(2)
gi|940350689|emb|CUR70740.1| capsid protein Milk vetch dwarf virus
gi|940350689|emb|CUR70740.1| capsid protein Milk vetch dwarf virus(2)
gi|52000640|sp|Q8V987.1|CAPSD MDV9 RecName: FullCapsid protein ShortCP AltName: FullCoat protein Milk vetch dwarf virus (isolate 9)
gi|52000640|sp|Q8V987.1|CAPSD MDV9 RecName: FullCapsid protein ShortCP AltName: FullCoat protein Milk vetch dwarf virus (isolate 9)(2)
gi|20177474|ref|NP 619767.1| capsid protein Milk vetch dwarf virus
gi|20177474|ref|NP 619767.1| capsid protein Milk vetch dwarf virus(2)
gi|567316339|gb|AHC72195.1| capsid protein Faba bean necrotic stunt virus
gi|567316339|gb|AHC72195.1| capsid protein Faba bean necrotic stunt virus(2)
gi|283482206|emb|CAG77491.1| capsid protein Faba bean necrotic stunt virus
gi|283482206|emb|CAG77491.1| capsid protein Faba bean necrotic stunt virus(2)
gi|260779818|gb|ACX50514.1| CP Faba bean necrotic stunt virus
gi|255961481|ref|YP 003104738.1| capsid protein Faba bean necrotic stunt virus
gi|255961481|ref|YP 003104738.1| capsid protein Faba bean necrotic stunt virus(2)
gi|260779818|gb|ACX50514.1| CP Faba bean necrotic stunt virus(2)
gi|567316391|gb|AHC72221.1| capsid protein Faba bean necrotic stunt virus(2)
gi|567316391|gb|AHC72221.1| capsid protein Faba bean necrotic stunt virus
30-1G
gi|571797919|ref|YP 008997806.1| capsid protein Pea yellow stunt virus
gi|571797919|ref|YP 008997806.1| capsid protein Pea yellow stunt virus(2)
gi|571797911|ref|YP 008997802.1| capsid protein Black medic leafroll virus
gi|571797911|ref|YP 008997802.1| capsid protein Black medic leafroll virus(2)
gi|567316285|gb|AHC72168.1| capsid protein Black medic leafroll virus
gi|567316285|gb|AHC72168.1| capsid protein Black medic leafroll virus(2)
gi|567316305|gb|AHC72178.1| capsid protein Black medic leafroll virus
gi|567316305|gb|AHC72178.1| capsid protein Black medic leafroll virus(2)
gi|52000720|sp|Q87012.1|CAPSD SCSVF RecName: FullCapsid protein ShortCP AltName: FullCoat protein Subterranean clover stunt virus (strain F)(2)
gi|52000720|sp|Q87012.1|CAPSD SCSVF RecName: FullCapsid protein ShortCP AltName: FullCoat protein Subterranean clover stunt virus (strain F)
gi|52000721|sp|Q87019.1|CAPSD SCSVJ RecName: FullCapsid protein ShortCP AltName: FullCoat protein Subterranean clover stunt virus (strain J)
gi|52000721|sp|Q87019.1|CAPSD SCSVJ RecName: FullCapsid protein ShortCP AltName: FullCoat protein Subterranean clover stunt virus (strain J)(2)
gi|567316493|gb|AHC72272.1| capsid protein Pea necrotic yellow dwarf virus
gi|570033505|ref|YP 008992019.1| capsid protein Pea necrotic yellow dwarf virus
gi|570033505|ref|YP 008992019.1| capsid protein Pea necrotic yellow dwarf virus(2)
gi|567316493|gb|AHC72272.1| capsid protein Pea necrotic yellow dwarf virus(2)
20-1E
gi|859805580|gb|AKO71288.1| capsid protein Banana bunchy top virus
0.2

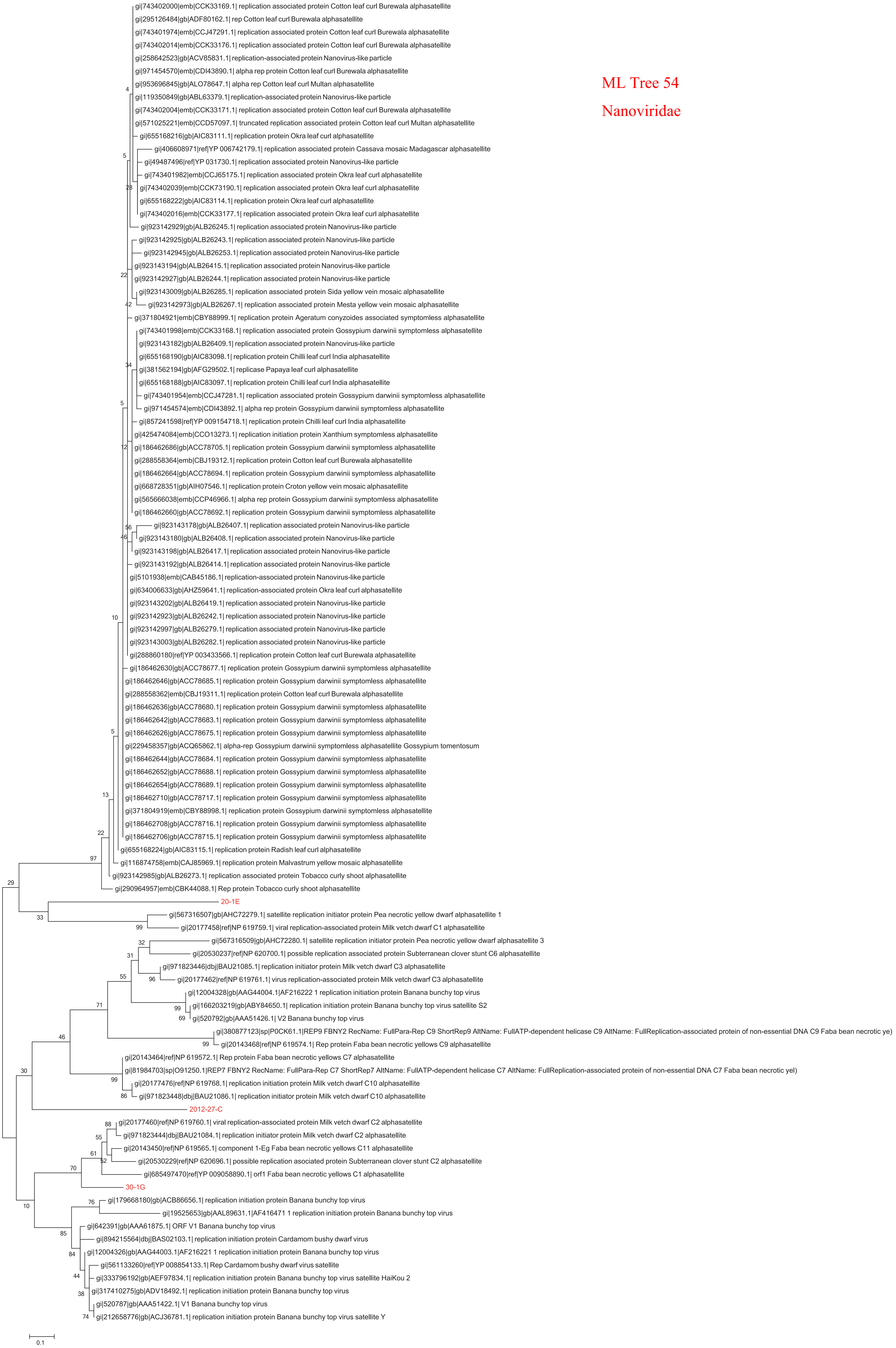
ML Tree 54
Nanoviridae
gi|743402000|emb|CCK33169.1| replication associated protein Cotton leaf curl Burewala alphasatellite
gi|295126484|gb|ADF80162.1| rep Cotton leaf curl Burewala alphasatellite
gi|743401974|emb|CCJ47291.1| replication associated protein Cotton leaf curl Burewala alphasatellite
gi|743402014|emb|CCK33176.1| replication associated protein Cotton leaf curl Burewala alphasatellite
gi|258642523|gb|ACV85831.1| replication-associated protein Nanovirus-like particle
gi|971454570|emb|CDI43890.1| alpha rep protein Cotton leaf curl Burewala alphasatellite
gi|953696845|gb|ALO78647.1| alpha rep Cotton leaf curl Multan alphasatellite
gi|119350849|gb|ABL63379.1| replication-associated protein Nanovirus-like particle
gi|743402004|emb|CCK33171.1| replication associated protein Cotton leaf curl Burewala alphasatellite
gi|571025221|emb|CCD57097.1| truncated replication associated protein Cotton leaf curl Multan alphasatellite
gi|655168216|gb|AIC83111.1| replication protein Okra leaf curl alphasatellite
gi|406608971|ref|YP 006742179.1| replication associated protein Cassava mosaic Madagascar alphasatellite
gi|49487496|ref|YP 031730.1| replication associated protein Nanovirus-like particle
gi|743401982|emb|CCJ65175.1| replication associated protein Okra leaf curl alphasatellite
gi|743402039|emb|CCK73190.1| replication associated protein Okra leaf curl alphasatellite
gi|655168222|gb|AIC83114.1| replication protein Okra leaf curl alphasatellite
gi|743402016|emb|CCK33177.1| replication associated protein Okra leaf curl alphasatellite
gi|923142929|gb|ALB26245.1| replication associated protein Nanovirus-like particle
gi|923142925|gb|ALB26243.1| replication associated protein Nanovirus-like particle
gi|923142945|gb|ALB26253.1| replication associated protein Nanovirus-like particle
gi|923143194|gb|ALB26415.1| replication associated protein Nanovirus-like particle
gi|923142927|gb|ALB26244.1| replication associated protein Nanovirus-like particle
gi|923143009|gb|ALB26285.1| replication associated protein Sida yellow vein mosaic alphasatellite
gi|923142973|gb|ALB26267.1| replication associated protein Mesta yellow vein mosaic alphasatellite
gi|371804921|emb|CBY88999.1| replication protein Ageratum conyzoides associated symptomless alphasatellite
gi|743401998|emb|CCK33168.1| replication associated protein Gossypium darwinii symptomless alphasatellite
gi|923143182|gb|ALB26409.1| replication associated protein Nanovirus-like particle
gi|655168190|gb|AIC83098.1| replication protein Chilli leaf curl India alphasatellite
gi|381562194|gb|AFG29502.1| replicase Papaya leaf curl alphasatellite
gi|655168188|gb|AIC83097.1| replication protein Chilli leaf curl India alphasatellite
gi|743401954|emb|CCJ47281.1| replication associated protein Gossypium darwinii symptomless alphasatellite
gi|971454574|emb|CDI43892.1| alpha rep protein Gossypium darwinii symptomless alphasatellite
gi|857241598|ref|YP 009154718.1| replication protein Chilli leaf curl India alphasatellite
gi|425474084|emb|CCO13273.1| replication initiation protein Xanthium symptomless alphasatellite
gi|186462686|gb|ACC78705.1| replication protein Gossypium darwinii symptomless alphasatellite
gi|288558364|emb|CBJ19312.1| replication protein Cotton leaf curl Burewala alphasatellite
gi|186462664|gb|ACC78694.1| replication protein Gossypium darwinii symptomless alphasatellite
gi|668728351|gb|AIH07546.1| replication protein Croton yellow vein mosaic alphasatellite
gi|565666038|emb|CCP46966.1| alpha rep protein Gossypium darwinii symptomless alphasatellite
gi|186462660|gb|ACC78692.1| replication protein Gossypium darwinii symptomless alphasatellite
gi|923143178|gb|ALB26407.1| replication associated protein Nanovirus-like particle
gi|923143180|gb|ALB26408.1| replication associated protein Nanovirus-like particle
gi|923143198|gb|ALB26417.1| replication associated protein Nanovirus-like particle
gi|923143192|gb|ALB26414.1| replication associated protein Nanovirus-like particle
gi|5101938|emb|CAB45186.1| replication-associated protein Nanovirus-like particle
gi|634006633|gb|AHZ59641.1| replication-associated protein Okra leaf curl alphasatellite
gi|923143202|gb|ALB26419.1| replication associated protein Nanovirus-like particle
gi|923142923|gb|ALB26242.1| replication associated protein Nanovirus-like particle
gi|923142997|gb|ALB26279.1| replication associated protein Nanovirus-like particle
gi|923143003|gb|ALB26282.1| replication associated protein Nanovirus-like particle
gi|288860180|ref|YP 003433566.1| replication protein Cotton leaf curl Burewala alphasatellite
gi|186462630|gb|ACC78677.1| replication protein Gossypium darwinii symptomless alphasatellite
gi|186462646|gb|ACC78685.1| replication protein Gossypium darwinii symptomless alphasatellite
gi|288558362|emb|CBJ19311.1| replication protein Cotton leaf curl Burewala alphasatellite
gi|186462636|gb|ACC78680.1| replication protein Gossypium darwinii symptomless alphasatellite
gi|186462642|gb|ACC78683.1| replication protein Gossypium darwinii symptomless alphasatellite
gi|186462626|gb|ACC78675.1| replication protein Gossypium darwinii symptomless alphasatellite
gi|229458357|gb|ACQ65862.1| alpha-rep Gossypium darwinii symptomless alphasatellite Gossypium tomentosum
gi|186462644|gb|ACC78684.1| replication protein Gossypium darwinii symptomless alphasatellite
gi|186462652|gb|ACC78688.1| replication protein Gossypium darwinii symptomless alphasatellite
gi|186462654|gb|ACC78689.1| replication protein Gossypium darwinii symptomless alphasatellite
gi|186462710|gb|ACC78717.1| replication protein Gossypium darwinii symptomless alphasatellite
gi|371804919|emb|CBY88998.1| replication protein Gossypium darwinii symptomless alphasatellite
gi|186462708|gb|ACC78716.1| replication protein Gossypium darwinii symptomless alphasatellite
gi|186462706|gb|ACC78715.1| replication protein Gossypium darwinii symptomless alphasatellite
gi|655168224|gb|AIC83115.1| replication protein Radish leaf curl alphasatellite
gi|116874758|emb|CAJ85969.1| replication protein Malvastrum yellow mosaic alphasatellite
gi|923142985|gb|ALB26273.1| replication associated protein Tobacco curly shoot alphasatellite
gi|290964957|emb|CBK44088.1| Rep protein Tobacco curly shoot alphasatellite
20-1E
gi|567316507|gb|AHC72279.1| satellite replication initiator protein Pea necrotic yellow dwarf alphasatellite 1
gi|20177458|ref|NP 619759.1| viral replication-associated protein Milk vetch dwarf C1 alphasatellite
gi|567316509|gb|AHC72280.1| satellite replication initiator protein Pea necrotic yellow dwarf alphasatellite 3
gi|20530237|ref|NP 620700.1| possible replication associated protein Subterranean clover stunt C6 alphasatellite
gi|971823446|dbj|BAU21085.1| replication initiator protein Milk vetch dwarf C3 alphasatellite
gi|20177462|ref|NP 619761.1| virus replication-associated protein Milk vetch dwarf C3 alphasatellite
gi|12004328|gb|AAG44004.1|AF216222 1 replication initiation protein Banana bunchy top virus
gi|166203219|gb|ABY84650.1| replication initiation protein Banana bunchy top virus satellite S2
gi|520792|gb|AAA51426.1| V2 Banana bunchy top virus
gi|380877123|sp|P0CK61.1|REP9 FBNY2 RecName: FullPara-Rep C9 ShortRep9 AltName: FullATP-dependent helicase C9 AltName: FullReplication-associated protein of non-essential DNA C9 Faba bean necrotic ye)
gi|20143468|ref|NP 619574.1| Rep protein Faba bean necrotic yellows C9 alphasatellite
gi|20143464|ref|NP 619572.1| Rep protein Faba bean necrotic yellows C7 alphasatellite
gi|81984703|sp|O91250.1|REP7 FBNY2 RecName: FullPara-Rep C7 ShortRep7 AltName: FullATP-dependent helicase C7 AltName: FullReplication-associated protein of non-essential DNA C7 Faba bean necrotic yel)
gi|20177476|ref|NP 619768.1| replication initiation protein Milk vetch dwarf C10 alphasatellite
gi|971823448|dbj|BAU21086.1| replication initiator protein Milk vetch dwarf C10 alphasatellite
2012-27-C
gi|20177460|ref|NP 619760.1| viral replication-associated protein Milk vetch dwarf C2 alphasatellite
gi|971823444|dbj|BAU21084.1| replication initiator protein Milk vetch dwarf C2 alphasatellite
gi|20143450|ref|NP 619565.1| component 1-Eg Faba bean necrotic yellows C11 alphasatellite
gi|20530229|ref|NP 620696.1| possible replication asociated protein Subterranean clover stunt C2 alphasatellite
gi|685497470|ref|YP 009058890.1| orf1 Faba bean necrotic yellows C1 alphasatellite
30-1G
gi|179668180|gb|ACB86656.1| replication initiation protein Banana bunchy top virus
gi|19525653|gb|AAL89631.1|AF416471 1 replication initiation protein Banana bunchy top virus
gi|642391|gb|AAA61875.1| ORF V1 Banana bunchy top virus
gi|894215564|dbj|BAS02103.1| replication initiation protein Cardamom bushy dwarf virus
gi|12004326|gb|AAG44003.1|AF216221 1 replication initiation protein Banana bunchy top virus
gi|561133260|ref|YP 008854133.1| Rep Cardamom bushy dwarf virus satellite
gi|333796192|gb|AEF97834.1| replication initiation protein Banana bunchy top virus satellite HaiKou 2
gi|317410275|gb|ADV18492.1| replication initiation protein Banana bunchy top virus
gi|520787|gb|AAA51422.1| V1 Banana bunchy top virus
gi|212658776|gb|ACJ36781.1| replication initiation protein Banana bunchy top virus satellite Y
0.1

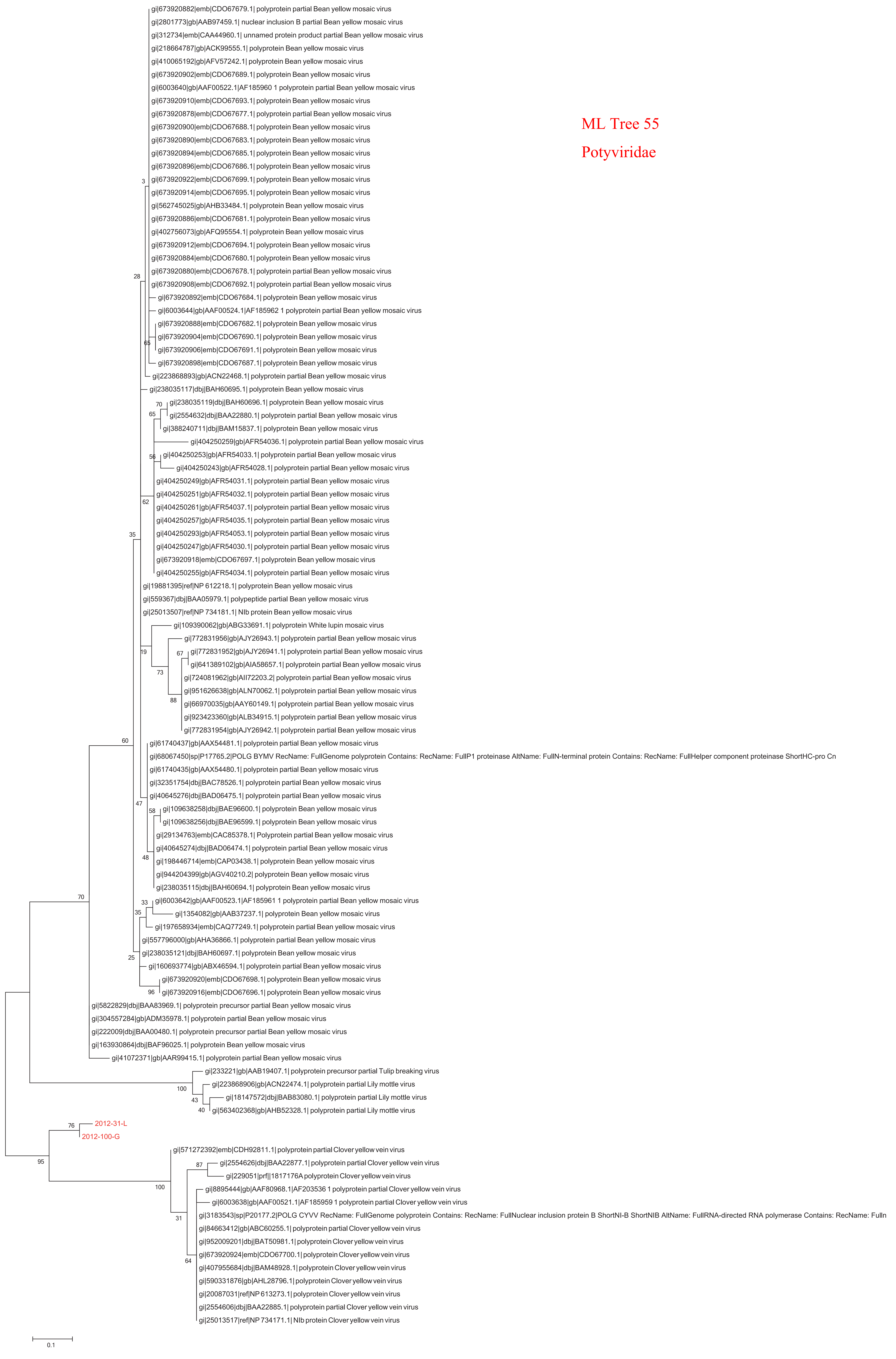
ML Tree 55
Potyviridae
gi|673920882|emb|CDO67679.1| polyprotein partial Bean yellow mosaic virus
gi|2801773|gb|AAB97459.1| nuclear inclusion B partial Bean yellow mosaic virus
gi|312734|emb|CAA44960.1| unnamed protein product partial Bean yellow mosaic virus
gi|218664787|gb|ACK99555.1| polyprotein Bean yellow mosaic virus
gi|410065192|gb|AFV57242.1| polyprotein Bean yellow mosaic virus
gi|673920902|emb|CDO67689.1| polyprotein Bean yellow mosaic virus
gi|6003640|gb|AAF00522.1|AF185960 1 polyprotein partial Bean yellow mosaic virus
gi|673920910|emb|CDO67693.1| polyprotein Bean yellow mosaic virus
gi|673920878|emb|CDO67677.1| polyprotein partial Bean yellow mosaic virus
gi|673920900|emb|CDO67688.1| polyprotein Bean yellow mosaic virus
gi|673920890|emb|CDO67683.1| polyprotein Bean yellow mosaic virus
gi|673920894|emb|CDO67685.1| polyprotein Bean yellow mosaic virus
gi|673920896|emb|CDO67686.1| polyprotein Bean yellow mosaic virus
gi|673920922|emb|CDO67699.1| polyprotein Bean yellow mosaic virus
3
gi|673920914|emb|CDO67695.1| polyprotein Bean yellow mosaic virus
gi|562745025|gb|AHB33484.1| polyprotein Bean yellow mosaic virus
gi|673920886|emb|CDO67681.1| polyprotein Bean yellow mosaic virus
gi|402756073|gb|AFQ95554.1| polyprotein Bean yellow mosaic virus
gi|673920912|emb|CDO67694.1| polyprotein Bean yellow mosaic virus
gi|673920884|emb|CDO67680.1| polyprotein Bean yellow mosaic virus
28
gi|673920880|emb|CDO67678.1| polyprotein partial Bean yellow mosaic virus
gi|673920908|emb|CDO67692.1| polyprotein partial Bean yellow mosaic virus
gi|673920892|emb|CDO67684.1| polyprotein Bean yellow mosaic virus
gi|6003644|gb|AAF00524.1|AF185962 1 polyprotein partial Bean yellow mosaic virus
gi|673920888|emb|CDO67682.1| polyprotein Bean yellow mosaic virus
gi|673920904|emb|CDO67690.1| polyprotein Bean yellow mosaic virus
65
gi|673920906|emb|CDO67691.1| polyprotein Bean yellow mosaic virus
gi|673920898|emb|CDO67687.1| polyprotein Bean yellow mosaic virus
gi|223868893|gb|ACN22468.1| polyprotein partial Bean yellow mosaic virus
gi|238035117|dbj|BAH60695.1| polyprotein Bean yellow mosaic virus
70
gi|238035119|dbj|BAH60696.1| polyprotein Bean yellow mosaic virus
65
gi|2554632|dbj|BAA22880.1| polyprotein partial Bean yellow mosaic virus
gi|388240711|dbj|BAM15837.1| polyprotein Bean yellow mosaic virus
gi|404250259|gb|AFR54036.1| polyprotein partial Bean yellow mosaic virus
56
gi|404250253|gb|AFR54033.1| polyprotein partial Bean yellow mosaic virus
gi|404250243|gb|AFR54028.1| polyprotein partial Bean yellow mosaic virus
gi|404250249|gb|AFR54031.1| polyprotein partial Bean yellow mosaic virus
gi|404250251|gb|AFR54032.1| polyprotein partial Bean yellow mosaic virus
62
gi|404250261|gb|AFR54037.1| polyprotein partial Bean yellow mosaic virus
gi|404250257|gb|AFR54035.1| polyprotein partial Bean yellow mosaic virus
35
gi|404250293|gb|AFR54053.1| polyprotein partial Bean yellow mosaic virus
gi|404250247|gb|AFR54030.1| polyprotein partial Bean yellow mosaic virus
gi|673920918|emb|CDO67697.1| polyprotein Bean yellow mosaic virus
gi|404250255|gb|AFR54034.1| polyprotein partial Bean yellow mosaic virus
gi|19881395|ref|NP 612218.1| polyprotein Bean yellow mosaic virus
gi|559367|dbj|BAA05979.1| polypeptide partial Bean yellow mosaic virus
gi|25013507|ref|NP 734181.1| NIb protein Bean yellow mosaic virus
gi|109390062|gb|ABG33691.1| polyprotein White lupin mosaic virus
gi|772831956|gb|AJY26943.1| polyprotein partial Bean yellow mosaic virus
19
67
gi|772831952|gb|AJY26941.1| polyprotein partial Bean yellow mosaic virus
gi|641389102|gb|AIA58657.1| polyprotein partial Bean yellow mosaic virus
73
gi|724081962|gb|AII72203.2| polyprotein partial Bean yellow mosaic virus
gi|951626638|gb|ALN70062.1| polyprotein partial Bean yellow mosaic virus
88
gi|66970035|gb|AAY60149.1| polyprotein partial Bean yellow mosaic virus
gi|923423360|gb|ALB34915.1| polyprotein partial Bean yellow mosaic virus
gi|772831954|gb|AJY26942.1| polyprotein partial Bean yellow mosaic virus
60
gi|61740437|gb|AAX54481.1| polyprotein partial Bean yellow mosaic virus
gi|68067450|sp|P17765.2|POLG BYMV RecName: FullGenome polyprotein Contains: RecName: FullP1 proteinase AltName: FullN-terminal protein Contains: RecName: FullHelper component proteinase ShortHC-pro Cn
gi|61740435|gb|AAX54480.1| polyprotein partial Bean yellow mosaic virus
gi|32351754|dbj|BAC78526.1| polyprotein partial Bean yellow mosaic virus
gi|40645276|dbj|BAD06475.1| polyprotein partial Bean yellow mosaic virus
47
58
gi|109638258|dbj|BAE96600.1| polyprotein Bean yellow mosaic virus
gi|109638256|dbj|BAE96599.1| polyprotein Bean yellow mosaic virus
gi|29134763|emb|CAC85378.1| Polyprotein partial Bean yellow mosaic virus
gi|40645274|dbj|BAD06474.1| polyprotein partial Bean yellow mosaic virus
48
gi|198446714|emb|CAP03438.1| polyprotein Bean yellow mosaic virus
gi|944204399|gb|AGV40210.2| polyprotein Bean yellow mosaic virus
gi|238035115|dbj|BAH60694.1| polyprotein Bean yellow mosaic virus
70
33
gi|6003642|gb|AAF00523.1|AF185961 1 polyprotein partial Bean yellow mosaic virus
35
gi|1354082|gb|AAB37237.1| polyprotein Bean yellow mosaic virus
gi|197658934|emb|CAQ77249.1| polyprotein partial Bean yellow mosaic virus
gi|557796000|gb|AHA36866.1| polyprotein partial Bean yellow mosaic virus
25
gi|238035121|dbj|BAH60697.1| polyprotein Bean yellow mosaic virus
gi|160693774|gb|ABX46594.1| polyprotein partial Bean yellow mosaic virus
gi|673920920|emb|CDO67698.1| polyprotein Bean yellow mosaic virus
96
gi|673920916|emb|CDO67696.1| polyprotein Bean yellow mosaic virus
gi|5822829|dbj|BAA83969.1| polyprotein precursor partial Bean yellow mosaic virus
gi|304557284|gb|ADM35978.1| polyprotein partial Bean yellow mosaic virus
gi|222009|dbj|BAA00480.1| polyprotein precursor partial Bean yellow mosaic virus
gi|163930864|dbj|BAF96025.1| polyprotein Bean yellow mosaic virus
gi|41072371|gb|AAR99415.1| polyprotein partial Bean yellow mosaic virus
gi|233221|gb|AAB19407.1| polyprotein precursor partial Tulip breaking virus
100
gi|223868906|gb|ACN22474.1| polyprotein partial Lily mottle virus
43
gi|18147572|dbj|BAB83080.1| polyprotein partial Lily mottle virus
40
gi|563402368|gb|AHB52328.1| polyprotein partial Lily mottle virus
76
2012-31-L
2012-100-G
95
gi|571272392|emb|CDH92811.1| polyprotein partial Clover yellow vein virus
87
gi|2554626|dbj|BAA22877.1| polyprotein partial Clover yellow vein virus
gi|229051|prf||1817176A polyprotein Clover yellow vein virus
100
gi|8895444|gb|AAF80968.1|AF203536 1 polyprotein partial Clover yellow vein virus
gi|6003638|gb|AAF00521.1|AF185959 1 polyprotein partial Clover yellow vein virus
31
gi|3183543|sp|P20177.2|POLG CYVV RecName: FullGenome polyprotein Contains: RecName: FullNuclear inclusion protein B ShortNI-B ShortNIB AltName: FullRNA-directed RNA polymerase Contains: RecName: FullIn
gi|84663412|gb|ABC60255.1| polyprotein partial Clover yellow vein virus
gi|952009201|dbj|BAT50981.1| polyprotein Clover yellow vein virus
gi|673920924|emb|CDO67700.1| polyprotein Clover yellow vein virus
64
gi|407955684|dbj|BAM48928.1| polyprotein Clover yellow vein virus
gi|590331876|gb|AHL28796.1| polyprotein Clover yellow vein virus
gi|20087031|ref|NP 613273.1| polyprotein Clover yellow vein virus
gi|2554606|dbj|BAA22885.1| polyprotein partial Clover yellow vein virus
gi|25013517|ref|NP 734171.1| NIb protein Clover yellow vein virus
0.1

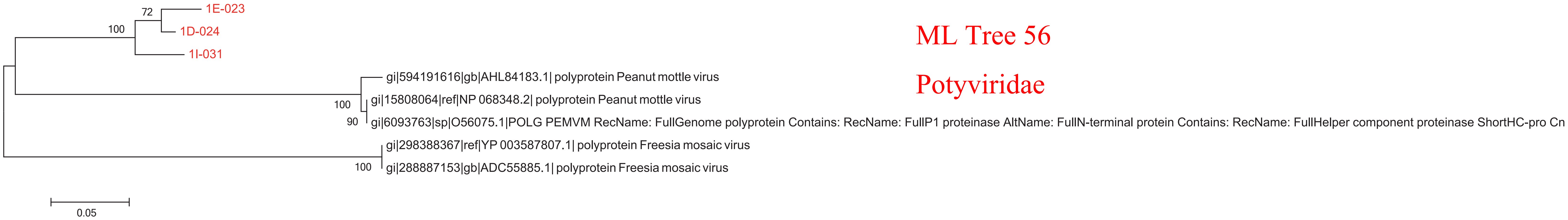

ML Tree 56

Potyviridae

ML Tree 57

Potyviridae

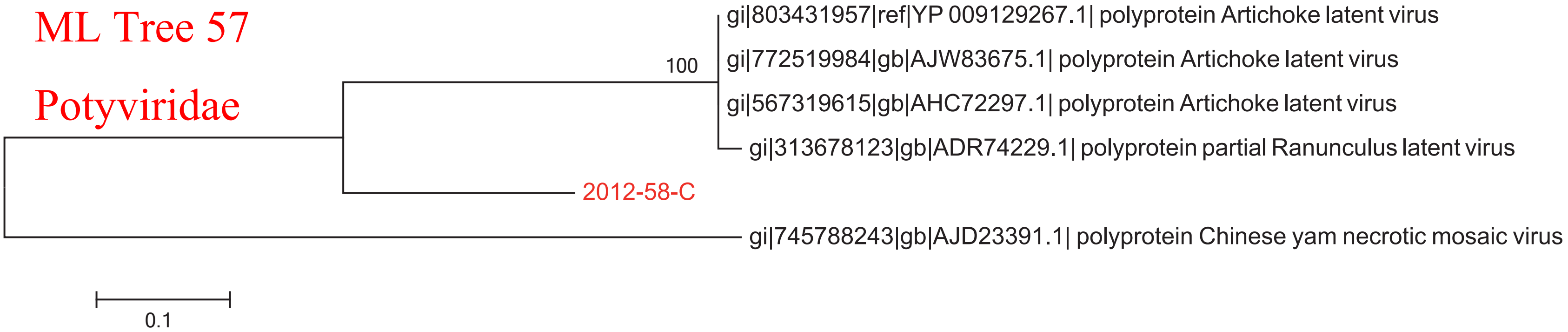

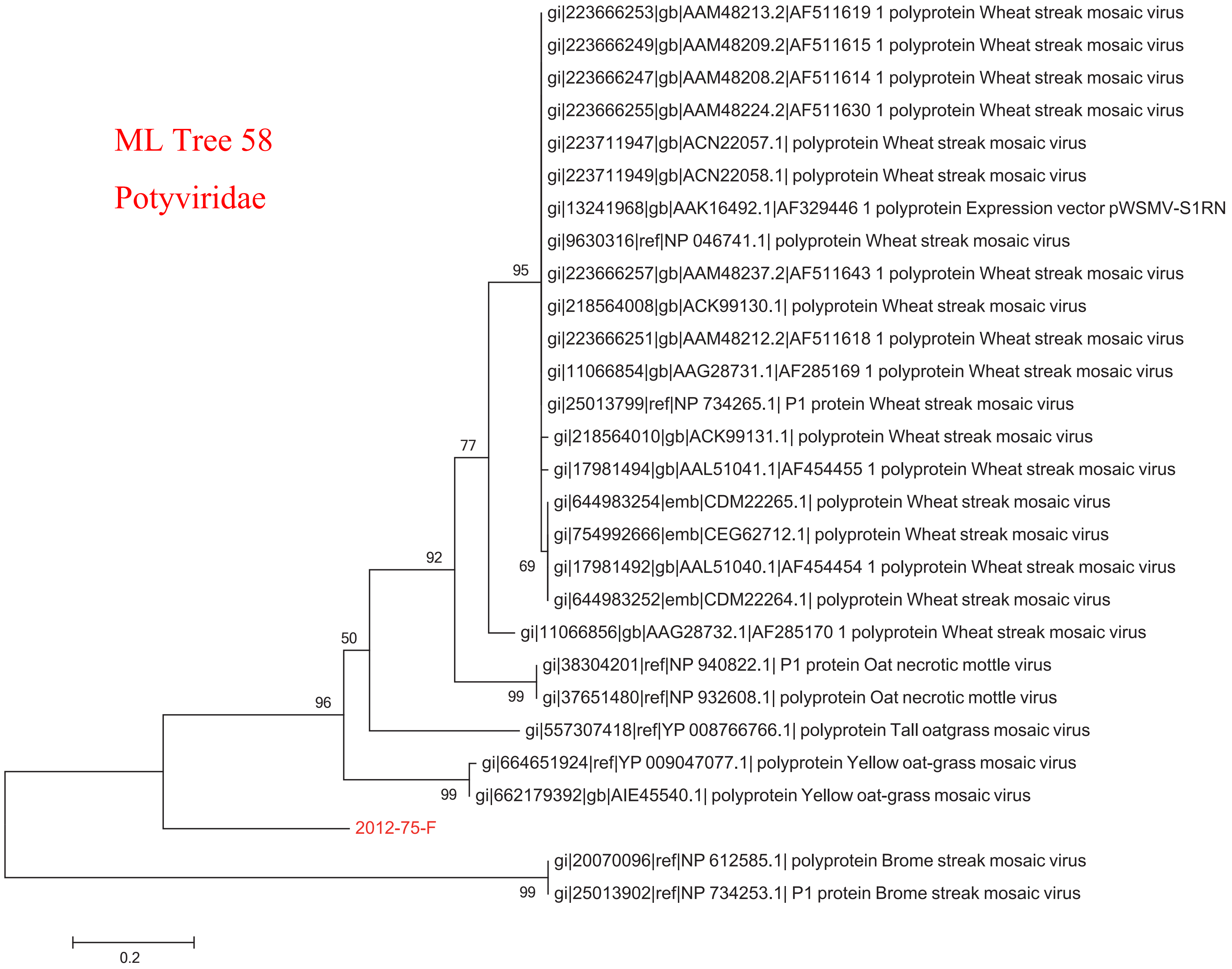
ML Tree 58
Potyviridae
gi|223666253|gb|AAM48213.2|AF511619 1 polyprotein Wheat streak mosaic virus
gi|223666249|gb|AAM48209.2|AF511615 1 polyprotein Wheat streak mosaic virus
gi|223666247|gb|AAM48208.2|AF511614 1 polyprotein Wheat streak mosaic virus
gi|223666255|gb|AAM48224.2|AF511630 1 polyprotein Wheat streak mosaic virus
gi|223711947|gb|ACN22057.1| polyprotein Wheat streak mosaic virus
gi|223711949|gb|ACN22058.1| polyprotein Wheat streak mosaic virus
gi|13241968|gb|AAK16492.1|AF329446 1 polyprotein Expression vector pWSMV-S1RN
gi|9630316|ref|NP 046741.1| polyprotein Wheat streak mosaic virus
95
gi|223666257|gb|AAM48237.2|AF511643 1 polyprotein Wheat streak mosaic virus
gi|218564008|gb|ACK99130.1| polyprotein Wheat streak mosaic virus
gi|223666251|gb|AAM48212.2|AF511618 1 polyprotein Wheat streak mosaic virus
gi|11066854|gb|AAG28731.1|AF285169 1 polyprotein Wheat streak mosaic virus
gi|25013799|ref|NP 734265.1| P1 protein Wheat streak mosaic virus
gi|218564010|gb|ACK99131.1| polyprotein Wheat streak mosaic virus
77
gi|17981494|gb|AAL51041.1|AF454455 1 polyprotein Wheat streak mosaic virus
gi|644983254|emb|CDM22265.1| polyprotein Wheat streak mosaic virus
gi|754992666|emb|CEG62712.1| polyprotein Wheat streak mosaic virus
92
69
gi|17981492|gb|AAL51040.1|AF454454 1 polyprotein Wheat streak mosaic virus
gi|644983252|emb|CDM22264.1| polyprotein Wheat streak mosaic virus
50
gi|11066856|gb|AAG28732.1|AF285170 1 polyprotein Wheat streak mosaic virus
gi|38304201|ref|NP 940822.1| P1 protein Oat necrotic mottle virus
99
gi|37651480|ref|NP 932608.1| polyprotein Oat necrotic mottle virus
96
gi|557307418|ref|YP 008766766.1| polyprotein Tall oatgrass mosaic virus
gi|664651924|ref|YP 009047077.1| polyprotein Yellow oat-grass mosaic virus
99
gi|662179392|gb|AIE45540.1| polyprotein Yellow oat-grass mosaic virus
2012-75-F
gi|20070096|ref|NP 612585.1| polyprotein Brome streak mosaic virus
99
gi|25013902|ref|NP 734253.1| P1 protein Brome streak mosaic virus
0.2

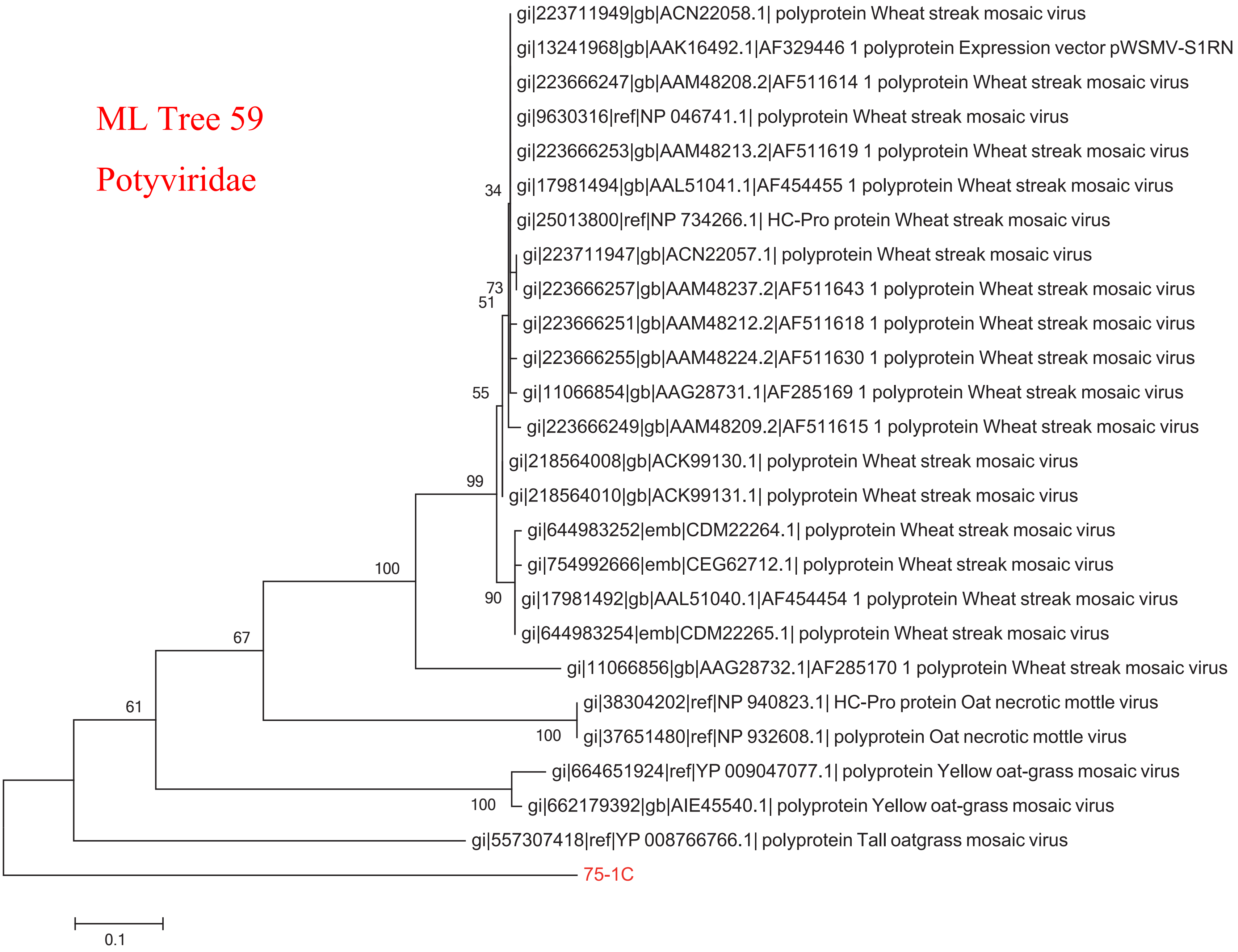
ML Tree 59
Potyviridae
gi|223711949|gb|ACN22058.1| polyprotein Wheat streak mosaic virus
gi|13241968|gb|AAK16492.1|AF329446 1 polyprotein Expression vector pWSMV-S1RN
gi|223666247|gb|AAM48208.2|AF511614 1 polyprotein Wheat streak mosaic virus
gi|9630316|ref|NP 046741.1| polyprotein Wheat streak mosaic virus
gi|223666253|gb|AAM48213.2|AF511619 1 polyprotein Wheat streak mosaic virus
gi|17981494|gb|AAL51041.1|AF454455 1 polyprotein Wheat streak mosaic virus
gi|25013800|ref|NP 734266.1| HC-Pro protein Wheat streak mosaic virus
gi|223711947|gb|ACN22057.1| polyprotein Wheat streak mosaic virus
gi|223666257|gb|AAM48237.2|AF511643 1 polyprotein Wheat streak mosaic virus
gi|223666251|gb|AAM48212.2|AF511618 1 polyprotein Wheat streak mosaic virus
gi|223666255|gb|AAM48224.2|AF511630 1 polyprotein Wheat streak mosaic virus
gi|11066854|gb|AAG28731.1|AF285169 1 polyprotein Wheat streak mosaic virus
gi|223666249|gb|AAM48209.2|AF511615 1 polyprotein Wheat streak mosaic virus
gi|218564008|gb|ACK99130.1| polyprotein Wheat streak mosaic virus
gi|218564010|gb|ACK99131.1| polyprotein Wheat streak mosaic virus
gi|644983252|emb|CDM22264.1| polyprotein Wheat streak mosaic virus
gi|754992666|emb|CEG62712.1| polyprotein Wheat streak mosaic virus
gi|17981492|gb|AAL51040.1|AF454454 1 polyprotein Wheat streak mosaic virus
gi|644983254|emb|CDM22265.1| polyprotein Wheat streak mosaic virus
gi|11066856|gb|AAG28732.1|AF285170 1 polyprotein Wheat streak mosaic virus
gi|38304202|ref|NP 940823.1| HC-Pro protein Oat necrotic mottle virus
gi|37651480|ref|NP 932608.1| polyprotein Oat necrotic mottle virus
gi|664651924|ref|YP 009047077.1| polyprotein Yellow oat-grass mosaic virus
gi|662179392|gb|AIE45540.1| polyprotein Yellow oat-grass mosaic virus
gi|557307418|ref|YP 008766766.1| polyprotein Tall oatgrass mosaic virus
75-1C
34
73
51
55
99
90
100
67
100
61
100
0.1

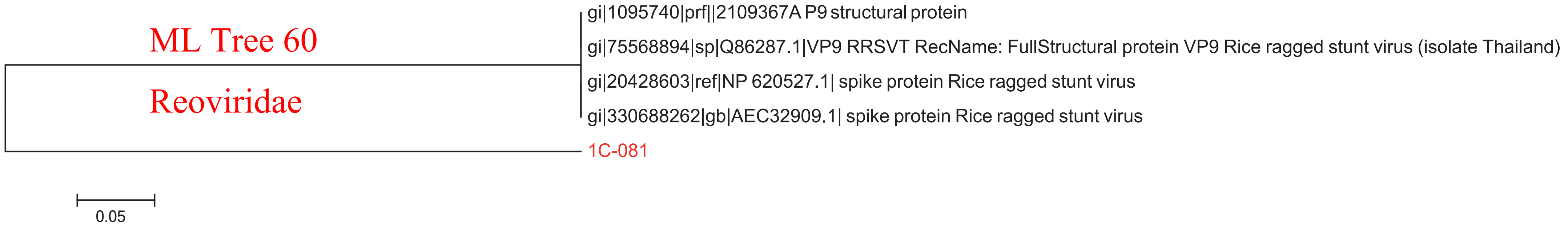
ML Tree 60
Reoviridae
gi|1095740|prf||2109367A P9 structural protein
gi|75568894|sp|Q86287.1|VP9 RRSVT RecName: FullStructural protein VP9 Rice ragged stunt virus (isolate Thailand)
gi|20428603|ref|NP 620527.1| spike protein Rice ragged stunt virus
gi|330688262|gb|AEC32909.1| spike protein Rice ragged stunt virus
1C-081
0.05

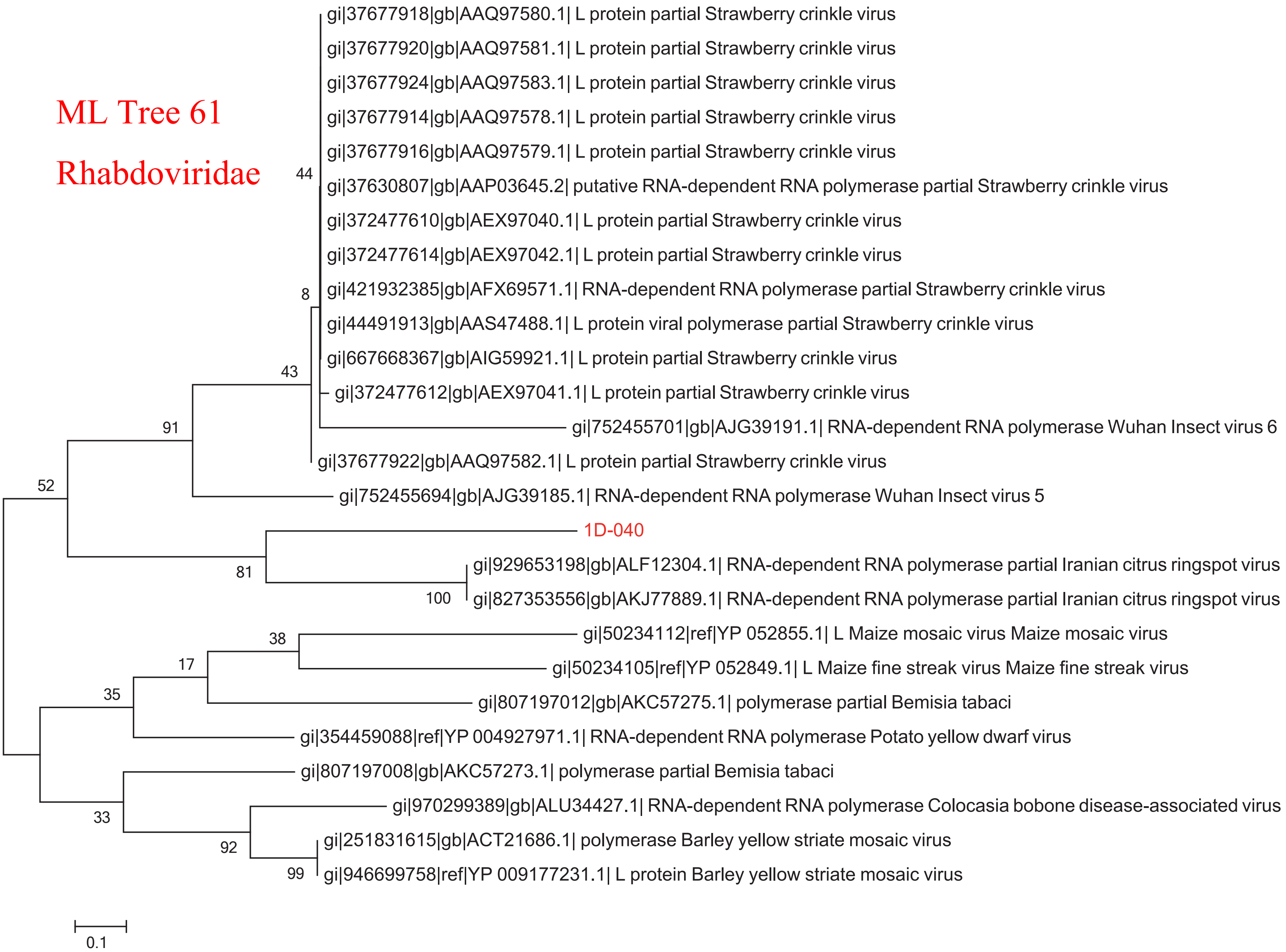

ML Tree 61
Rhabdoviridae
gi|37677918|gb|AAQ97580.1| L protein partial Strawberry crinkle virus
gi|37677920|gb|AAQ97581.1| L protein partial Strawberry crinkle virus
gi|37677924|gb|AAQ97583.1| L protein partial Strawberry crinkle virus
gi|37677914|gb|AAQ97578.1| L protein partial Strawberry crinkle virus
gi|37677916|gb|AAQ97579.1| L protein partial Strawberry crinkle virus
44
gi|37630807|gb|AAP03645.2| putative RNA-dependent RNA polymerase partial Strawberry crinkle virus
gi|372477610|gb|AEX97040.1| L protein partial Strawberry crinkle virus
gi|372477614|gb|AEX97042.1| L protein partial Strawberry crinkle virus
8
gi|421932385|gb|AFX69571.1| RNA-dependent RNA polymerase partial Strawberry crinkle virus
gi|44491913|gb|AAS47488.1| L protein viral polymerase partial Strawberry crinkle virus
gi|667668367|gb|AIG59921.1| L protein partial Strawberry crinkle virus
43
gi|372477612|gb|AEX97041.1| L protein partial Strawberry crinkle virus
91
gi|752455701|gb|AJG39191.1| RNA-dependent RNA polymerase Wuhan Insect virus 6
gi|37677922|gb|AAQ97582.1| L protein partial Strawberry crinkle virus
52
gi|752455694|gb|AJG39185.1| RNA-dependent RNA polymerase Wuhan Insect virus 5
1D-040
81
gi|929653198|gb|ALF12304.1| RNA-dependent RNA polymerase partial Iranian citrus ringspot virus
100
gi|827353556|gb|AKJ77889.1| RNA-dependent RNA polymerase partial Iranian citrus ringspot virus
38
gi|50234112|ref|YP 052855.1| L Maize mosaic virus Maize mosaic virus
17
gi|50234105|ref|YP 052849.1| L Maize fine streak virus Maize fine streak virus
35
gi|807197012|gb|AKC57275.1| polymerase partial Bemisia tabaci
gi|354459088|ref|YP 004927971.1| RNA-dependent RNA polymerase Potato yellow dwarf virus
gi|807197008|gb|AKC57273.1| polymerase partial Bemisia tabaci
gi|970299389|gb|ALU34427.1| RNA-dependent RNA polymerase Colocasia bobone disease-associated virus
33
gi|251831615|gb|ACT21686.1| polymerase Barley yellow striate mosaic virus
92
99
gi|946699758|ref|YP 009177231.1| L protein Barley yellow striate mosaic virus
0.1

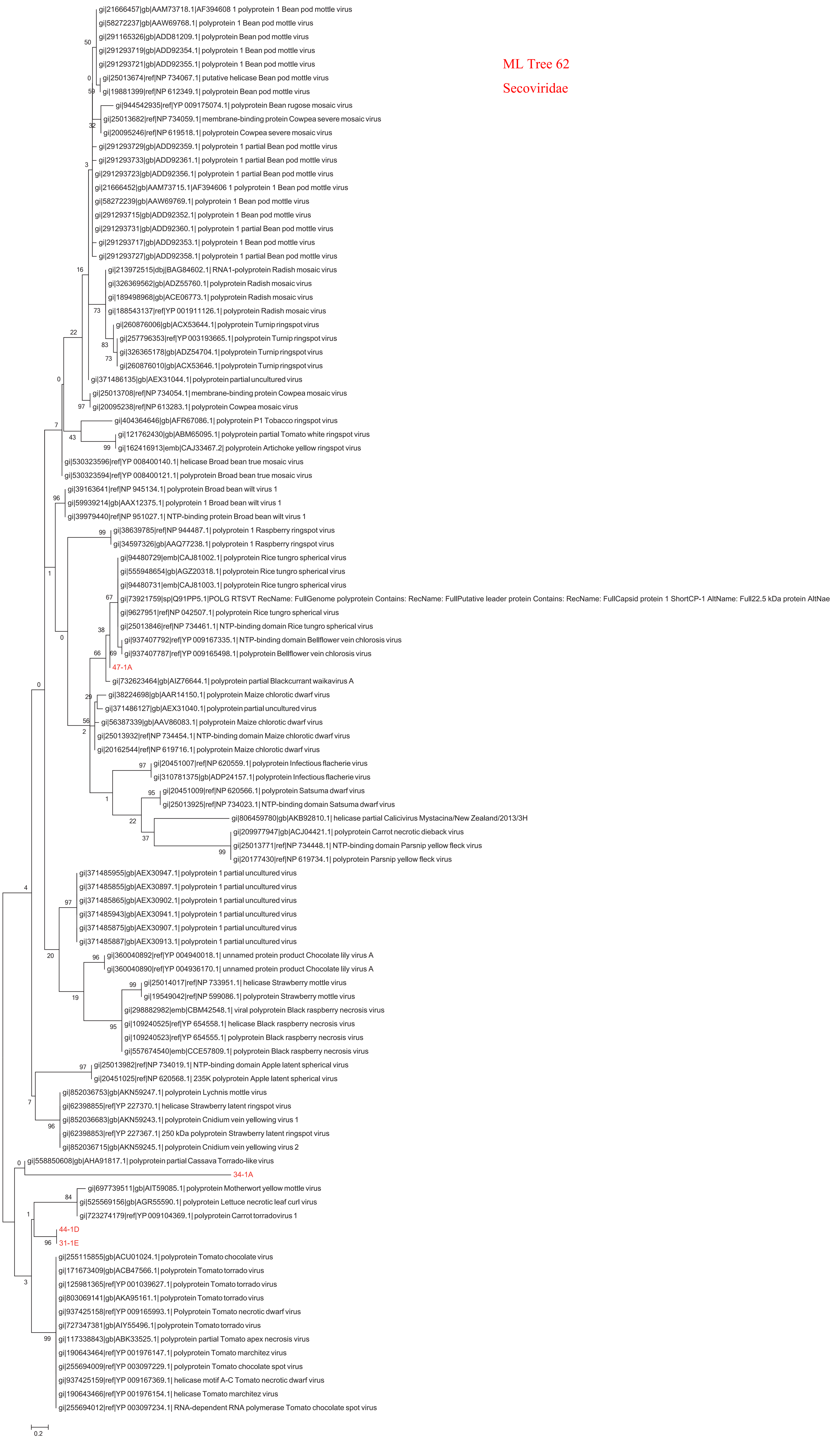

ML Tree 62
Secoviridae
gi|21666457|gb|AAM73718.1|AF394608 1 polyprotein 1 Bean pod mottle virus
gi|58272237|gb|AAW69768.1| polyprotein 1 Bean pod mottle virus
gi|291165326|gb|ADD81209.1| polyprotein Bean pod mottle virus
gi|291293719|gb|ADD92354.1| polyprotein 1 Bean pod mottle virus
gi|291293721|gb|ADD92355.1| polyprotein 1 Bean pod mottle virus
gi|25013674|ref|NP 734067.1| putative helicase Bean pod mottle virus
gi|19881399|ref|NP 612349.1| polyprotein Bean pod mottle virus
gi|944542935|ref|YP 009175074.1| polyprotein Bean rugose mosaic virus
gi|25013682|ref|NP 734059.1| membrane-binding protein Cowpea severe mosaic virus
gi|20095246|ref|NP 619518.1| polyprotein Cowpea severe mosaic virus
gi|291293729|gb|ADD92359.1| polyprotein 1 partial Bean pod mottle virus
gi|291293733|gb|ADD92361.1| polyprotein 1 partial Bean pod mottle virus
gi|291293723|gb|ADD92356.1| polyprotein 1 partial Bean pod mottle virus
gi|21666452|gb|AAM73715.1|AF394606 1 polyprotein 1 Bean pod mottle virus
gi|58272239|gb|AAW69769.1| polyprotein 1 Bean pod mottle virus
gi|291293715|gb|ADD92352.1| polyprotein 1 Bean pod mottle virus
gi|291293731|gb|ADD92360.1| polyprotein 1 partial Bean pod mottle virus
gi|291293717|gb|ADD92353.1| polyprotein 1 Bean pod mottle virus
gi|291293727|gb|ADD92358.1| polyprotein 1 partial Bean pod mottle virus
gi|213972515|dbj|BAG84602.1| RNA1-polyprotein Radish mosaic virus
gi|326369562|gb|ADZ55760.1| polyprotein Radish mosaic virus
gi|189498968|gb|ACE06773.1| polyprotein Radish mosaic virus
gi|188543137|ref|YP 001911126.1| polyprotein Radish mosaic virus
gi|260876006|gb|ACX53644.1| polyprotein Turnip ringspot virus
gi|257796353|ref|YP 003193665.1| polyprotein Turnip ringspot virus
gi|326365178|gb|ADZ54704.1| polyprotein Turnip ringspot virus
gi|260876010|gb|ACX53646.1| polyprotein Turnip ringspot virus
gi|371486135|gb|AEX31044.1| polyprotein partial uncultured virus
gi|25013708|ref|NP 734054.1| membrane-binding protein Cowpea mosaic virus
gi|20095238|ref|NP 613283.1| polyprotein Cowpea mosaic virus
gi|404364646|gb|AFR67086.1| polyprotein P1 Tobacco ringspot virus
gi|121762430|gb|ABM65095.1| polyprotein partial Tomato white ringspot virus
gi|162416913|emb|CAJ33467.2| polyprotein Artichoke yellow ringspot virus
gi|530323596|ref|YP 008400140.1| helicase Broad bean true mosaic virus
gi|530323594|ref|YP 008400121.1| polyprotein Broad bean true mosaic virus
gi|39163641|ref|NP 945134.1| polyprotein Broad bean wilt virus 1
gi|59939214|gb|AAX12375.1| polyprotein 1 Broad bean wilt virus 1
gi|39979440|ref|NP 951027.1| NTP-binding protein Broad bean wilt virus 1
gi|38639785|ref|NP 944487.1| polyprotein 1 Raspberry ringspot virus
gi|34597326|gb|AAQ77238.1| polyprotein 1 Raspberry ringspot virus
gi|94480729|emb|CAJ81002.1| polyprotein Rice tungro spherical virus
gi|555948654|gb|AGZ20318.1| polyprotein Rice tungro spherical virus
gi|94480731|emb|CAJ81003.1| polyprotein Rice tungro spherical virus
gi|73921759|sp|Q91PP5.1|POLG RTSVT RecName: FullGenome polyprotein Contains: RecName: FullPutative leader protein Contains: RecName: FullCapsid protein 1 ShortCP-1 AltName: Full22.5 kDa protein AltNae
gi|9627951|ref|NP 042507.1| polyprotein Rice tungro spherical virus
gi|25013846|ref|NP 734461.1| NTP-binding domain Rice tungro spherical virus
gi|937407792|ref|YP 009167335.1| NTP-binding domain Bellflower vein chlorosis virus
gi|937407787|ref|YP 009165498.1| polyprotein Bellflower vein chlorosis virus
47-1A
gi|732623464|gb|AIZ76644.1| polyprotein partial Blackcurrant waikavirus A
gi|38224698|gb|AAR14150.1| polyprotein Maize chlorotic dwarf virus
gi|371486127|gb|AEX31040.1| polyprotein partial uncultured virus
gi|56387339|gb|AAV86083.1| polyprotein Maize chlorotic dwarf virus
gi|25013932|ref|NP 734454.1| NTP-binding domain Maize chlorotic dwarf virus
gi|20162544|ref|NP 619716.1| polyprotein Maize chlorotic dwarf virus
gi|20451007|ref|NP 620559.1| polyprotein Infectious flacherie virus
gi|310781375|gb|ADP24157.1| polyprotein Infectious flacherie virus
gi|20451009|ref|NP 620566.1| polyprotein Satsuma dwarf virus
gi|25013925|ref|NP 734023.1| NTP-binding domain Satsuma dwarf virus
gi|806459780|gb|AKB92810.1| helicase partial Calicivirus Mystacina/New Zealand/2013/3H
gi|209977947|gb|ACJ04421.1| polyprotein Carrot necrotic dieback virus
gi|25013771|ref|NP 734448.1| NTP-binding domain Parsnip yellow fleck virus
gi|20177430|ref|NP 619734.1| polyprotein Parsnip yellow fleck virus
gi|371485955|gb|AEX30947.1| polyprotein 1 partial uncultured virus
gi|371485855|gb|AEX30897.1| polyprotein 1 partial uncultured virus
gi|371485865|gb|AEX30902.1| polyprotein 1 partial uncultured virus
gi|371485943|gb|AEX30941.1| polyprotein 1 partial uncultured virus
gi|371485875|gb|AEX30907.1| polyprotein 1 partial uncultured virus
gi|371485887|gb|AEX30913.1| polyprotein 1 partial uncultured virus
gi|360040892|ref|YP 004940018.1| unnamed protein product Chocolate lily virus A
gi|360040890|ref|YP 004936170.1| unnamed protein product Chocolate lily virus A
gi|25014017|ref|NP 733951.1| helicase Strawberry mottle virus
gi|19549042|ref|NP 599086.1| polyprotein Strawberry mottle virus
gi|298882982|emb|CBM42548.1| viral polyprotein Black raspberry necrosis virus
gi|109240525|ref|YP 654558.1| helicase Black raspberry necrosis virus
gi|109240523|ref|YP 654555.1| polyprotein Black raspberry necrosis virus
gi|557674540|emb|CCE57809.1| polyprotein Black raspberry necrosis virus
gi|25013982|ref|NP 734019.1| NTP-binding domain Apple latent spherical virus
gi|20451025|ref|NP 620568.1| 235K polyprotein Apple latent spherical virus
gi|852036753|gb|AKN59247.1| polyprotein Lychnis mottle virus
gi|62398855|ref|YP 227370.1| helicase Strawberry latent ringspot virus
gi|852036683|gb|AKN59243.1| polyprotein Cnidium vein yellowing virus 1
gi|62398853|ref|YP 227367.1| 250 kDa polyprotein Strawberry latent ringspot virus
gi|852036715|gb|AKN59245.1| polyprotein Cnidium vein yellowing virus 2
gi|558850608|gb|AHA91817.1| polyprotein partial Cassava Torrado-like virus
34-1A
gi|697739511|gb|AIT59085.1| polyprotein Motherwort yellow mottle virus
gi|525569156|gb|AGR55590.1| polyprotein Lettuce necrotic leaf curl virus
gi|723274179|ref|YP 009104369.1| polyprotein Carrot torradovirus 1
44-1D
31-1E
gi|255115855|gb|ACU01024.1| polyprotein Tomato chocolate virus
gi|171673409|gb|ACB47566.1| polyprotein Tomato torrado virus
gi|125981365|ref|YP 001039627.1| polyprotein Tomato torrado virus
gi|803069141|gb|AKA95161.1| polyprotein Tomato torrado virus
gi|937425158|ref|YP 009165993.1| Polyprotein Tomato necrotic dwarf virus
gi|727347381|gb|AIY55496.1| polyprotein Tomato torrado virus
gi|117338843|gb|ABK33525.1| polyprotein partial Tomato apex necrosis virus
gi|190643464|ref|YP 001976147.1| polyprotein Tomato marchitez virus
gi|255694009|ref|YP 003097229.1| polyprotein Tomato chocolate spot virus
gi|937425159|ref|YP 009167369.1| helicase motif A-C Tomato necrotic dwarf virus
gi|190643466|ref|YP 001976154.1| helicase Tomato marchitez virus
gi|255694012|ref|YP 003097234.1| RNA-dependent RNA polymerase Tomato chocolate spot virus
0.2

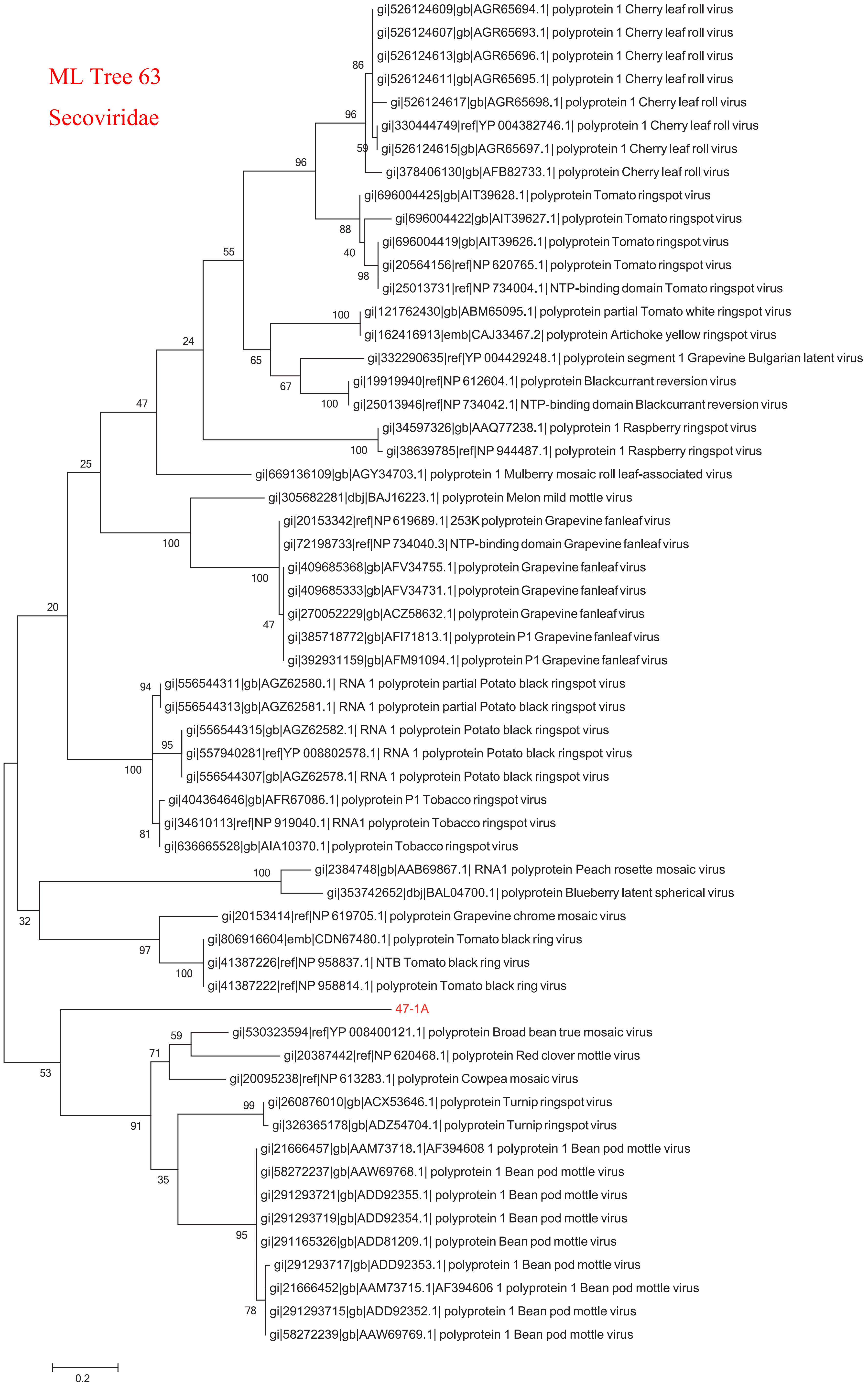

ML Tree 63
Secoviridae
gi|526124609|gb|AGR65694.1| polyprotein 1 Cherry leaf roll virus
gi|526124607|gb|AGR65693.1| polyprotein 1 Cherry leaf roll virus
gi|526124613|gb|AGR65696.1| polyprotein 1 Cherry leaf roll virus
gi|526124611|gb|AGR65695.1| polyprotein 1 Cherry leaf roll virus
gi|526124617|gb|AGR65698.1| polyprotein 1 Cherry leaf roll virus
gi|330444749|ref|YP 004382746.1| polyprotein 1 Cherry leaf roll virus
gi|526124615|gb|AGR65697.1| polyprotein 1 Cherry leaf roll virus
gi|378406130|gb|AFB82733.1| polyprotein Cherry leaf roll virus
gi|696004425|gb|AIT39628.1| polyprotein Tomato ringspot virus
gi|696004422|gb|AIT39627.1| polyprotein Tomato ringspot virus
gi|696004419|gb|AIT39626.1| polyprotein Tomato ringspot virus
gi|20564156|ref|NP 620765.1| polyprotein Tomato ringspot virus
gi|25013731|ref|NP 734004.1| NTP-binding domain Tomato ringspot virus
gi|121762430|gb|ABM65095.1| polyprotein partial Tomato white ringspot virus
gi|162416913|emb|CAJ33467.2| polyprotein Artichoke yellow ringspot virus
gi|332290635|ref|YP 004429248.1| polyprotein segment 1 Grapevine Bulgarian latent virus
gi|19919940|ref|NP 612604.1| polyprotein Blackcurrant reversion virus
gi|25013946|ref|NP 734042.1| NTP-binding domain Blackcurrant reversion virus
gi|34597326|gb|AAQ77238.1| polyprotein 1 Raspberry ringspot virus
gi|38639785|ref|NP 944487.1| polyprotein 1 Raspberry ringspot virus
gi|669136109|gb|AGY34703.1| polyprotein 1 Mulberry mosaic roll leaf-associated virus
gi|305682281|dbj|BAJ16223.1| polyprotein Melon mild mottle virus
gi|20153342|ref|NP 619689.1| 253K polyprotein Grapevine fanleaf virus
gi|72198733|ref|NP 734040.3| NTP-binding domain Grapevine fanleaf virus
gi|409685368|gb|AFV34755.1| polyprotein Grapevine fanleaf virus
gi|409685333|gb|AFV34731.1| polyprotein Grapevine fanleaf virus
gi|270052229|gb|ACZ58632.1| polyprotein Grapevine fanleaf virus
gi|385718772|gb|AFI71813.1| polyprotein P1 Grapevine fanleaf virus
gi|392931159|gb|AFM91094.1| polyprotein P1 Grapevine fanleaf virus
gi|556544311|gb|AGZ62580.1| RNA 1 polyprotein partial Potato black ringspot virus
gi|556544313|gb|AGZ62581.1| RNA 1 polyprotein partial Potato black ringspot virus
gi|556544315|gb|AGZ62582.1| RNA 1 polyprotein Potato black ringspot virus
gi|557940281|ref|YP 008802578.1| RNA 1 polyprotein Potato black ringspot virus
gi|556544307|gb|AGZ62578.1| RNA 1 polyprotein Potato black ringspot virus
gi|404364646|gb|AFR67086.1| polyprotein P1 Tobacco ringspot virus
gi|34610113|ref|NP 919040.1| RNA1 polyprotein Tobacco ringspot virus
gi|636665528|gb|AIA10370.1| polyprotein Tobacco ringspot virus
gi|2384748|gb|AAB69867.1| RNA1 polyprotein Peach rosette mosaic virus
gi|353742652|dbj|BAL04700.1| polyprotein Blueberry latent spherical virus
gi|20153414|ref|NP 619705.1| polyprotein Grapevine chrome mosaic virus
gi|806916604|emb|CDN67480.1| polyprotein Tomato black ring virus
gi|41387226|ref|NP 958837.1| NTB Tomato black ring virus
gi|41387222|ref|NP 958814.1| polyprotein Tomato black ring virus
47-1A
gi|530323594|ref|YP 008400121.1| polyprotein Broad bean true mosaic virus
gi|20387442|ref|NP 620468.1| polyprotein Red clover mottle virus
gi|20095238|ref|NP 613283.1| polyprotein Cowpea mosaic virus
gi|260876010|gb|ACX53646.1| polyprotein Turnip ringspot virus
gi|326365178|gb|ADZ54704.1| polyprotein Turnip ringspot virus
gi|21666457|gb|AAM73718.1|AF394608 1 polyprotein 1 Bean pod mottle virus
gi|58272237|gb|AAW69768.1| polyprotein 1 Bean pod mottle virus
gi|291293721|gb|ADD92355.1| polyprotein 1 Bean pod mottle virus
gi|291293719|gb|ADD92354.1| polyprotein 1 Bean pod mottle virus
gi|291165326|gb|ADD81209.1| polyprotein Bean pod mottle virus
gi|291293717|gb|ADD92353.1| polyprotein 1 Bean pod mottle virus
gi|21666452|gb|AAM73715.1|AF394606 1 polyprotein 1 Bean pod mottle virus
gi|291293715|gb|ADD92352.1| polyprotein 1 Bean pod mottle virus
gi|58272239|gb|AAW69769.1| polyprotein 1 Bean pod mottle virus
0.2

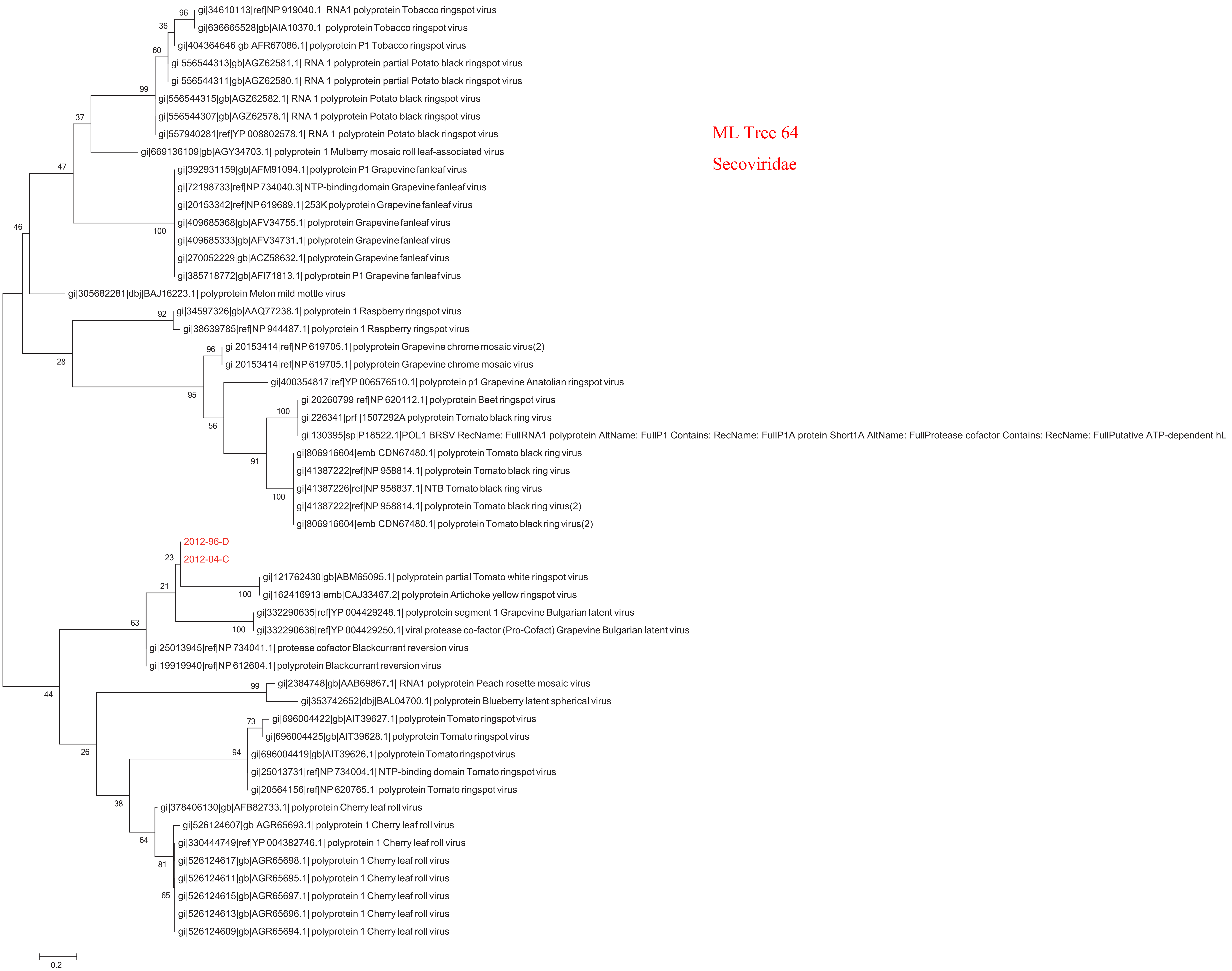
ML Tree 64
Secoviridae
gi|34610113|ref|NP 919040.1| RNA1 polyprotein Tobacco ringspot virus
gi|636665528|gb|AIA10370.1| polyprotein Tobacco ringspot virus
gi|404364646|gb|AFR67086.1| polyprotein P1 Tobacco ringspot virus
gi|556544313|gb|AGZ62581.1| RNA 1 polyprotein partial Potato black ringspot virus
gi|556544311|gb|AGZ62580.1| RNA 1 polyprotein partial Potato black ringspot virus
gi|556544315|gb|AGZ62582.1| RNA 1 polyprotein Potato black ringspot virus
gi|556544307|gb|AGZ62578.1| RNA 1 polyprotein Potato black ringspot virus
gi|557940281|ref|YP 008802578.1| RNA 1 polyprotein Potato black ringspot virus
gi|669136109|gb|AGY34703.1| polyprotein 1 Mulberry mosaic roll leaf-associated virus
gi|392931159|gb|AFM91094.1| polyprotein P1 Grapevine fanleaf virus
gi|72198733|ref|NP 734040.3| NTP-binding domain Grapevine fanleaf virus
gi|20153342|ref|NP 619689.1| 253K polyprotein Grapevine fanleaf virus
gi|409685368|gb|AFV34755.1| polyprotein Grapevine fanleaf virus
gi|409685333|gb|AFV34731.1| polyprotein Grapevine fanleaf virus
gi|270052229|gb|ACZ58632.1| polyprotein Grapevine fanleaf virus
gi|385718772|gb|AFI71813.1| polyprotein P1 Grapevine fanleaf virus
gi|305682281|dbj|BAJ16223.1| polyprotein Melon mild mottle virus
gi|34597326|gb|AAQ77238.1| polyprotein 1 Raspberry ringspot virus
gi|38639785|ref|NP 944487.1| polyprotein 1 Raspberry ringspot virus
gi|20153414|ref|NP 619705.1| polyprotein Grapevine chrome mosaic virus(2)
gi|20153414|ref|NP 619705.1| polyprotein Grapevine chrome mosaic virus
gi|400354817|ref|YP 006576510.1| polyprotein p1 Grapevine Anatolian ringspot virus
gi|20260799|ref|NP 620112.1| polyprotein Beet ringspot virus
gi|226341|prf||1507292A polyprotein Tomato black ring virus
gi|130395|sp|P18522.1|POL1 BRSV RecName: FullRNA1 polyprotein AltName: FullP1 Contains: RecName: FullP1A protein Short1A AltName: FullProtease cofactor Contains: RecName: FullPutative ATP-dependent hL
gi|806916604|emb|CDN67480.1| polyprotein Tomato black ring virus
gi|41387222|ref|NP 958814.1| polyprotein Tomato black ring virus
gi|41387226|ref|NP 958837.1| NTB Tomato black ring virus
gi|41387222|ref|NP 958814.1| polyprotein Tomato black ring virus(2)
gi|806916604|emb|CDN67480.1| polyprotein Tomato black ring virus(2)
2012-96-D
2012-04-C
gi|121762430|gb|ABM65095.1| polyprotein partial Tomato white ringspot virus
gi|162416913|emb|CAJ33467.2| polyprotein Artichoke yellow ringspot virus
gi|332290635|ref|YP 004429248.1| polyprotein segment 1 Grapevine Bulgarian latent virus
gi|332290636|ref|YP 004429250.1| viral protease co-factor (Pro-Cofact) Grapevine Bulgarian latent virus
gi|25013945|ref|NP 734041.1| protease cofactor Blackcurrant reversion virus
gi|19919940|ref|NP 612604.1| polyprotein Blackcurrant reversion virus
gi|2384748|gb|AAB69867.1| RNA1 polyprotein Peach rosette mosaic virus
gi|353742652|dbj|BAL04700.1| polyprotein Blueberry latent spherical virus
gi|696004422|gb|AIT39627.1| polyprotein Tomato ringspot virus
gi|696004425|gb|AIT39628.1| polyprotein Tomato ringspot virus
gi|696004419|gb|AIT39626.1| polyprotein Tomato ringspot virus
gi|25013731|ref|NP 734004.1| NTP-binding domain Tomato ringspot virus
gi|20564156|ref|NP 620765.1| polyprotein Tomato ringspot virus
gi|378406130|gb|AFB82733.1| polyprotein Cherry leaf roll virus
gi|526124607|gb|AGR65693.1| polyprotein 1 Cherry leaf roll virus
gi|330444749|ref|YP 004382746.1| polyprotein 1 Cherry leaf roll virus
gi|526124617|gb|AGR65698.1| polyprotein 1 Cherry leaf roll virus
gi|526124611|gb|AGR65695.1| polyprotein 1 Cherry leaf roll virus
gi|526124615|gb|AGR65697.1| polyprotein 1 Cherry leaf roll virus
gi|526124613|gb|AGR65696.1| polyprotein 1 Cherry leaf roll virus
gi|526124609|gb|AGR65694.1| polyprotein 1 Cherry leaf roll virus
0.2

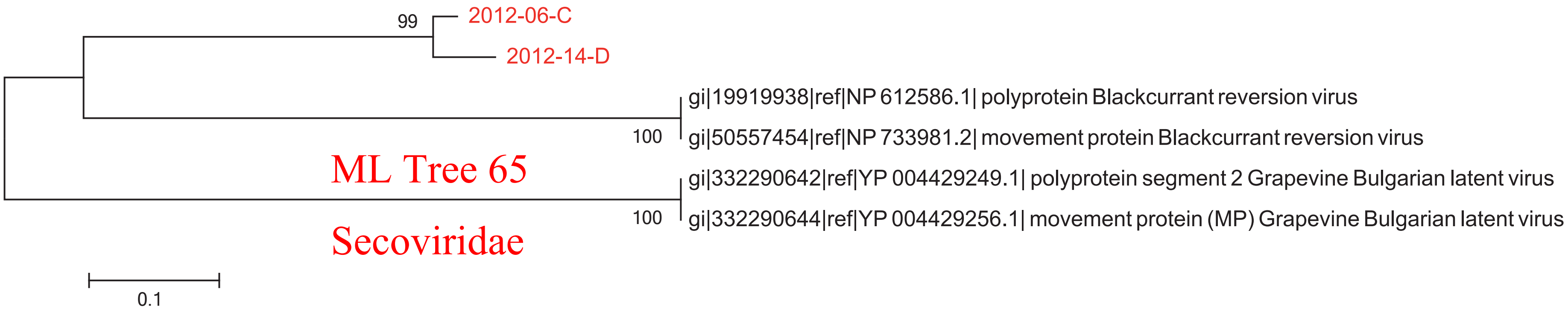

99
2012-06-C
2012-14-D
gi|19919938|ref|NP 612586.1| polyprotein Blackcurrant reversion virus
100
gi|50557454|ref|NP 733981.2| movement protein Blackcurrant reversion virus
ML Tree 65
gi|332290642|ref|YP 004429249.1| polyprotein segment 2 Grapevine Bulgarian latent virus
100
gi|332290644|ref|YP 004429256.1| movement protein (MP) Grapevine Bulgarian latent virus
Secoviridae
0.1

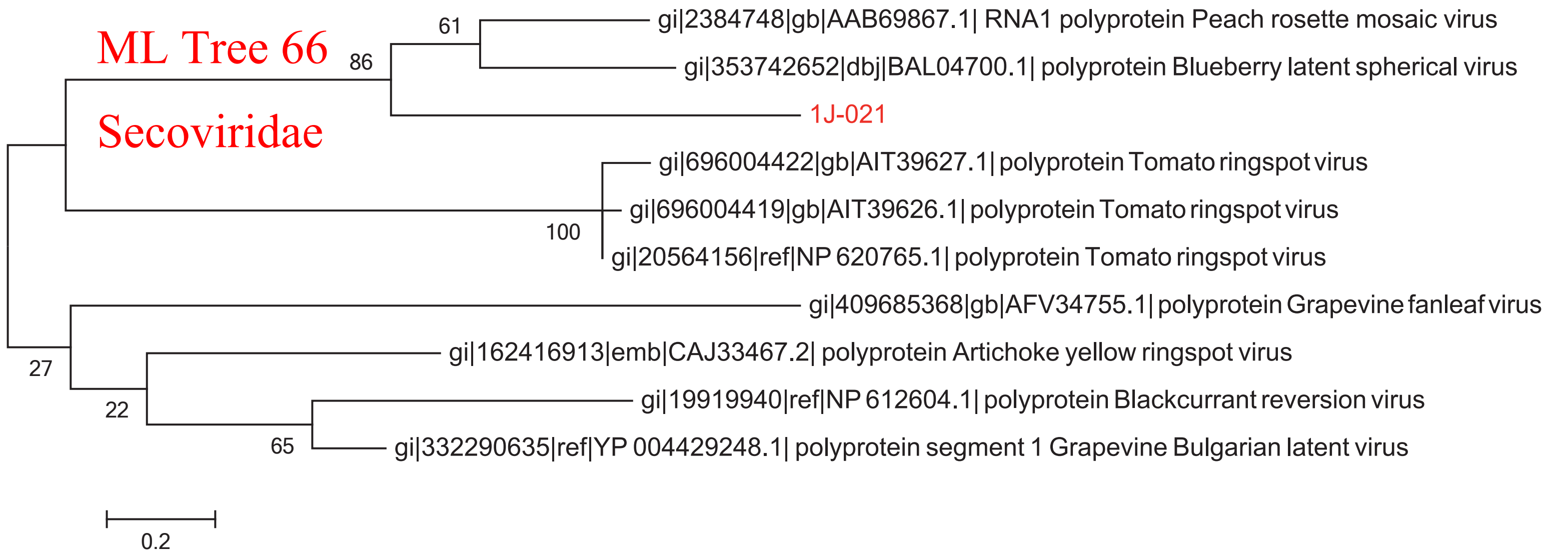
ML Tree 66
Secoviridae
gi|2384748|gb|AAB69867.1| RNA1 polyprotein Peach rosette mosaic virus
gi|353742652|dbj|BAL04700.1| polyprotein Blueberry latent spherical virus
1J-021
gi|696004422|gb|AIT39627.1| polyprotein Tomato ringspot virus
gi|696004419|gb|AIT39626.1| polyprotein Tomato ringspot virus
gi|20564156|ref|NP 620765.1| polyprotein Tomato ringspot virus
gi|409685368|gb|AFV34755.1| polyprotein Grapevine fanleaf virus
gi|162416913|emb|CAJ33467.2| polyprotein Artichoke yellow ringspot virus
gi|19919940|ref|NP 612604.1| polyprotein Blackcurrant reversion virus
gi|332290635|ref|YP 004429248.1| polyprotein segment 1 Grapevine Bulgarian latent virus
61
86
100
27
22
65
0.2

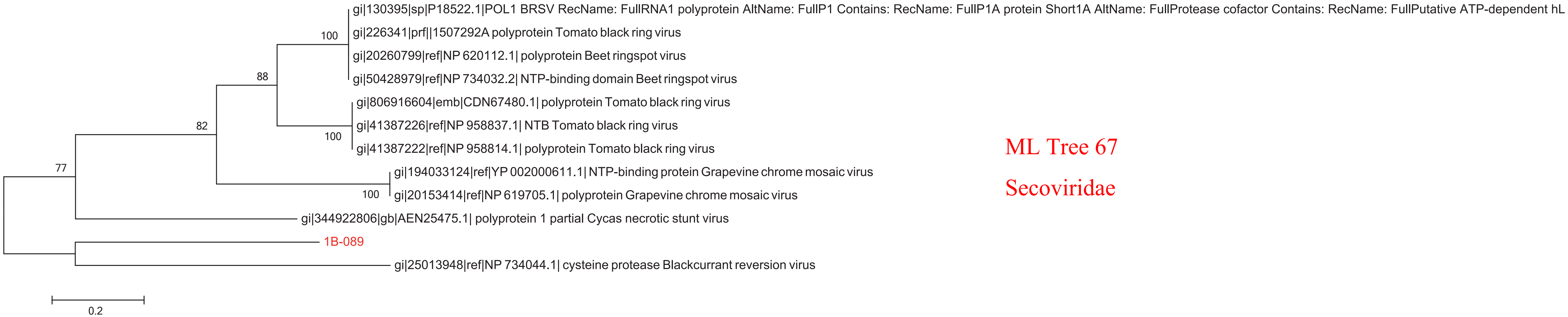
gi|130395|sp|P18522.1|POL1 BRSV RecName: FullRNA1 polyprotein AltName: FullP1 Contains: RecName: FullP1A protein Short1A AltName: FullProtease cofactor Contains: RecName: FullPutative ATP-dependent hL
gi|226341|prf||1507292A polyprotein Tomato black ring virus
gi|20260799|ref|NP 620112.1| polyprotein Beet ringspot virus
gi|50428979|ref|NP 734032.2| NTP-binding domain Beet ringspot virus
gi|806916604|emb|CDN67480.1| polyprotein Tomato black ring virus
gi|41387226|ref|NP 958837.1| NTB Tomato black ring virus
gi|41387222|ref|NP 958814.1| polyprotein Tomato black ring virus
gi|194033124|ref|YP 002000611.1| NTP-binding protein Grapevine chrome mosaic virus
gi|20153414|ref|NP 619705.1| polyprotein Grapevine chrome mosaic virus
gi|344922806|gb|AEN25475.1| polyprotein 1 partial Cycas necrotic stunt virus
1B-089
gi|25013948|ref|NP 734044.1| cysteine protease Blackcurrant reversion virus
100
88
100
82
100
77
0.2
ML Tree 67
Secoviridae

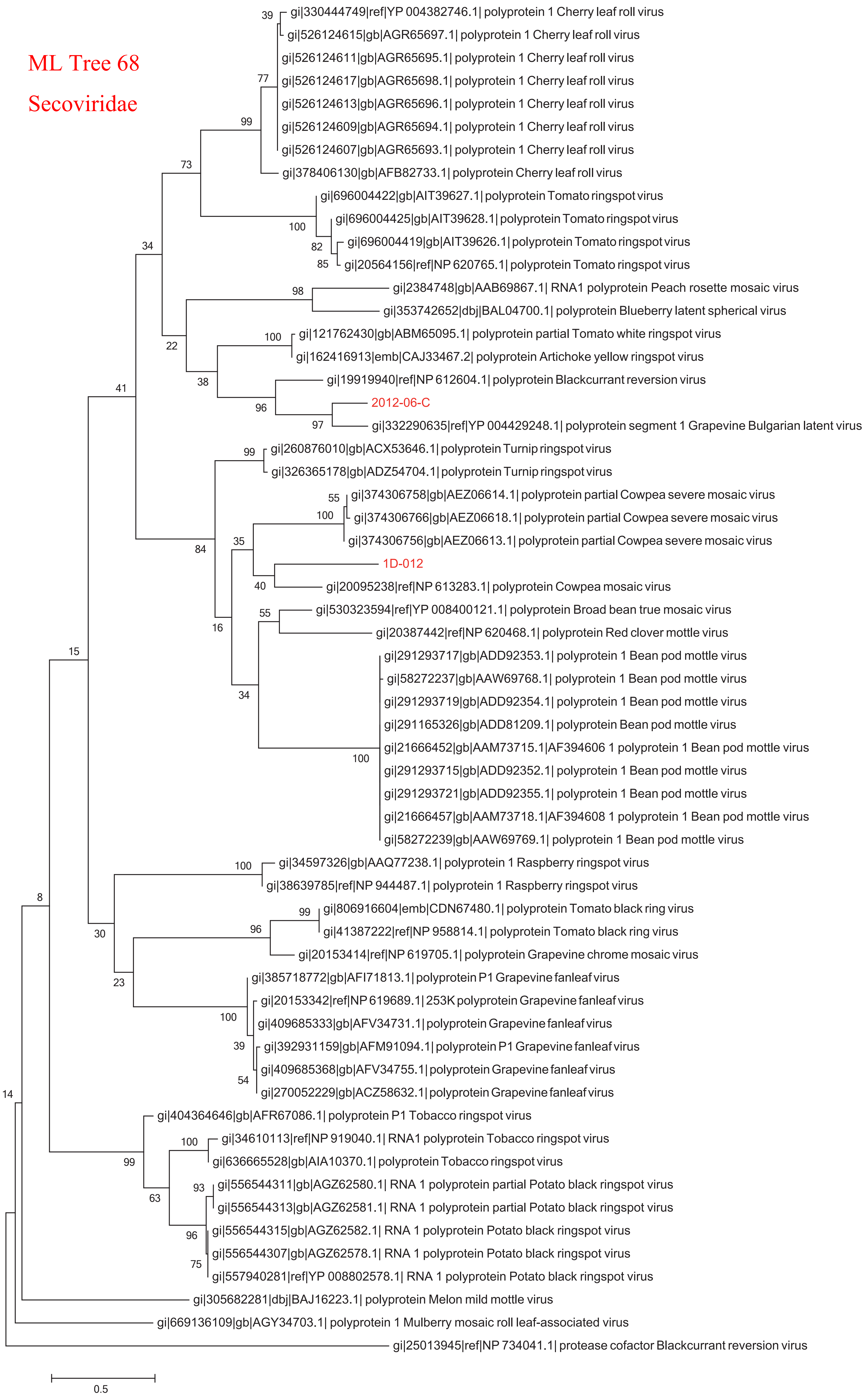

ML Tree 68
Secoviridae
gi|330444749|ref|YP 004382746.1| polyprotein 1 Cherry leaf roll virus
gi|526124615|gb|AGR65697.1| polyprotein 1 Cherry leaf roll virus
gi|526124611|gb|AGR65695.1| polyprotein 1 Cherry leaf roll virus
gi|526124617|gb|AGR65698.1| polyprotein 1 Cherry leaf roll virus
gi|526124613|gb|AGR65696.1| polyprotein 1 Cherry leaf roll virus
gi|526124609|gb|AGR65694.1| polyprotein 1 Cherry leaf roll virus
gi|526124607|gb|AGR65693.1| polyprotein 1 Cherry leaf roll virus
gi|378406130|gb|AFB82733.1| polyprotein Cherry leaf roll virus
gi|696004422|gb|AIT39627.1| polyprotein Tomato ringspot virus
gi|696004425|gb|AIT39628.1| polyprotein Tomato ringspot virus
gi|696004419|gb|AIT39626.1| polyprotein Tomato ringspot virus
gi|20564156|ref|NP 620765.1| polyprotein Tomato ringspot virus
gi|2384748|gb|AAB69867.1| RNA1 polyprotein Peach rosette mosaic virus
gi|353742652|dbj|BAL04700.1| polyprotein Blueberry latent spherical virus
gi|121762430|gb|ABM65095.1| polyprotein partial Tomato white ringspot virus
gi|162416913|emb|CAJ33467.2| polyprotein Artichoke yellow ringspot virus
gi|19919940|ref|NP 612604.1| polyprotein Blackcurrant reversion virus
2012-06-C
gi|332290635|ref|YP 004429248.1| polyprotein segment 1 Grapevine Bulgarian latent virus
gi|260876010|gb|ACX53646.1| polyprotein Turnip ringspot virus
gi|326365178|gb|ADZ54704.1| polyprotein Turnip ringspot virus
gi|374306758|gb|AEZ06614.1| polyprotein partial Cowpea severe mosaic virus
gi|374306766|gb|AEZ06618.1| polyprotein partial Cowpea severe mosaic virus
gi|374306756|gb|AEZ06613.1| polyprotein partial Cowpea severe mosaic virus
1D-012
gi|20095238|ref|NP 613283.1| polyprotein Cowpea mosaic virus
gi|530323594|ref|YP 008400121.1| polyprotein Broad bean true mosaic virus
gi|20387442|ref|NP 620468.1| polyprotein Red clover mottle virus
gi|291293717|gb|ADD92353.1| polyprotein 1 Bean pod mottle virus
gi|58272237|gb|AAW69768.1| polyprotein 1 Bean pod mottle virus
gi|291293719|gb|ADD92354.1| polyprotein 1 Bean pod mottle virus
gi|291165326|gb|ADD81209.1| polyprotein Bean pod mottle virus
gi|21666452|gb|AAM73715.1|AF394606 1 polyprotein 1 Bean pod mottle virus
gi|291293715|gb|ADD92352.1| polyprotein 1 Bean pod mottle virus
gi|291293721|gb|ADD92355.1| polyprotein 1 Bean pod mottle virus
gi|21666457|gb|AAM73718.1|AF394608 1 polyprotein 1 Bean pod mottle virus
gi|58272239|gb|AAW69769.1| polyprotein 1 Bean pod mottle virus
gi|34597326|gb|AAQ77238.1| polyprotein 1 Raspberry ringspot virus
gi|38639785|ref|NP 944487.1| polyprotein 1 Raspberry ringspot virus
gi|806916604|emb|CDN67480.1| polyprotein Tomato black ring virus
gi|41387222|ref|NP 958814.1| polyprotein Tomato black ring virus
gi|20153414|ref|NP 619705.1| polyprotein Grapevine chrome mosaic virus
gi|385718772|gb|AFI71813.1| polyprotein P1 Grapevine fanleaf virus
gi|20153342|ref|NP 619689.1| 253K polyprotein Grapevine fanleaf virus
gi|409685333|gb|AFV34731.1| polyprotein Grapevine fanleaf virus
gi|392931159|gb|AFM91094.1| polyprotein P1 Grapevine fanleaf virus
gi|409685368|gb|AFV34755.1| polyprotein Grapevine fanleaf virus
gi|270052229|gb|ACZ58632.1| polyprotein Grapevine fanleaf virus
gi|404364646|gb|AFR67086.1| polyprotein P1 Tobacco ringspot virus
gi|34610113|ref|NP 919040.1| RNA1 polyprotein Tobacco ringspot virus
gi|636665528|gb|AIA10370.1| polyprotein Tobacco ringspot virus
gi|556544311|gb|AGZ62580.1| RNA 1 polyprotein partial Potato black ringspot virus
gi|556544313|gb|AGZ62581.1| RNA 1 polyprotein partial Potato black ringspot virus
gi|556544315|gb|AGZ62582.1| RNA 1 polyprotein Potato black ringspot virus
gi|556544307|gb|AGZ62578.1| RNA 1 polyprotein Potato black ringspot virus
gi|557940281|ref|YP 008802578.1| RNA 1 polyprotein Potato black ringspot virus
gi|305682281|dbj|BAJ16223.1| polyprotein Melon mild mottle virus
gi|669136109|gb|AGY34703.1| polyprotein 1 Mulberry mosaic roll leaf-associated virus
gi|25013945|ref|NP 734041.1| protease cofactor Blackcurrant reversion virus
0.5

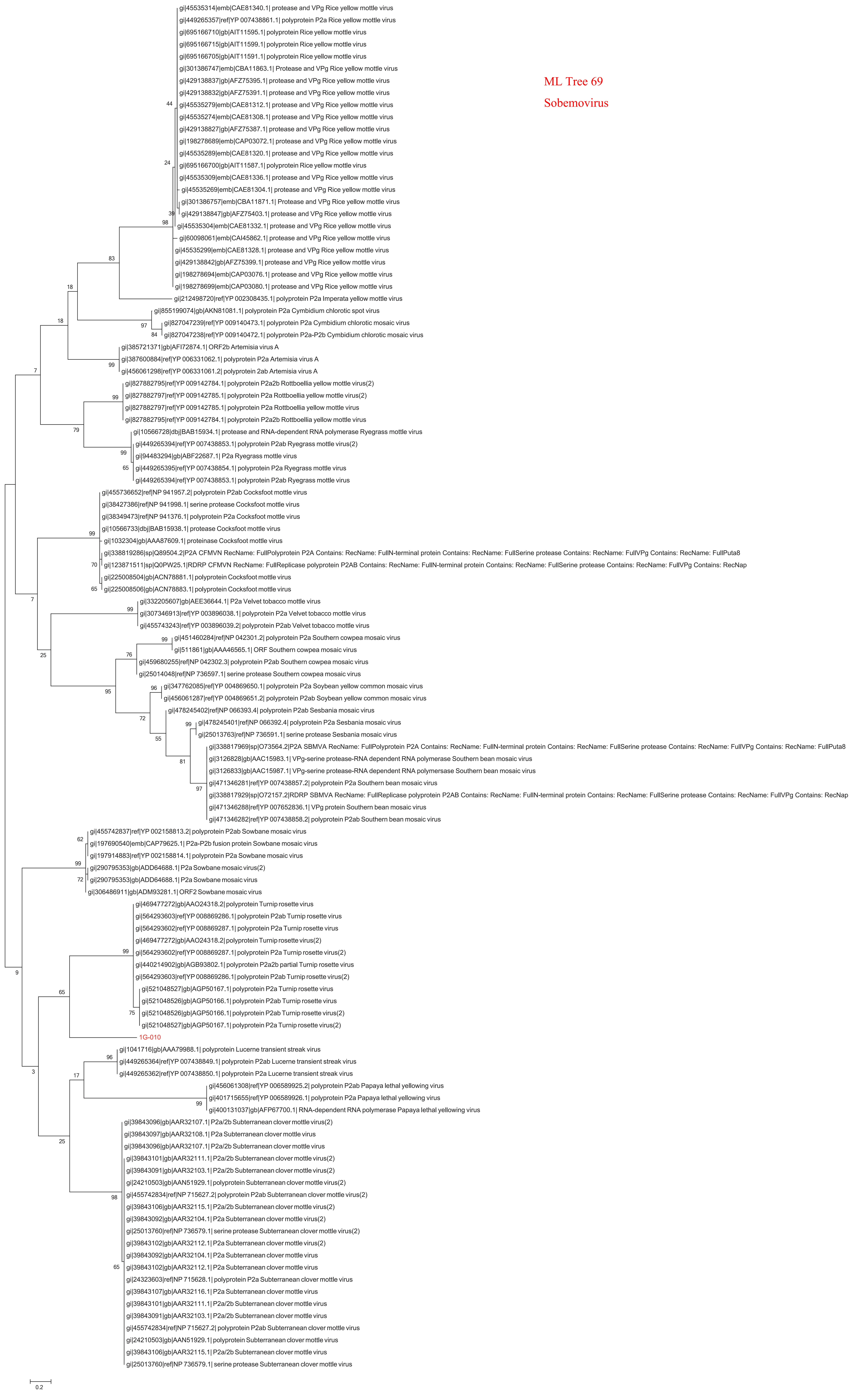
ML Tree 69
Sobemovirus
gi|45535314|emb|CAE81340.1| protease and VPg Rice yellow mottle virus
gi|449265357|ref|YP 007438861.1| polyprotein P2a Rice yellow mottle virus
gi|695166710|gb|AIT11595.1| polyprotein Rice yellow mottle virus
gi|695166715|gb|AIT11599.1| polyprotein Rice yellow mottle virus
gi|695166705|gb|AIT11591.1| polyprotein Rice yellow mottle virus
gi|301386747|emb|CBA11863.1| Protease and VPg Rice yellow mottle virus
gi|429138837|gb|AFZ75395.1| protease and VPg Rice yellow mottle virus
gi|429138832|gb|AFZ75391.1| protease and VPg Rice yellow mottle virus
gi|45535279|emb|CAE81312.1| protease and VPg Rice yellow mottle virus
gi|45535274|emb|CAE81308.1| protease and VPg Rice yellow mottle virus
gi|429138827|gb|AFZ75387.1| protease and VPg Rice yellow mottle virus
gi|198278689|emb|CAP03072.1| protease and VPg Rice yellow mottle virus
gi|45535289|emb|CAE81320.1| protease and VPg Rice yellow mottle virus
gi|695166700|gb|AIT11587.1| polyprotein Rice yellow mottle virus
gi|45535309|emb|CAE81336.1| protease and VPg Rice yellow mottle virus
gi|45535269|emb|CAE81304.1| protease and VPg Rice yellow mottle virus
gi|301386757|emb|CBA11871.1| Protease and VPg Rice yellow mottle virus
gi|429138847|gb|AFZ75403.1| protease and VPg Rice yellow mottle virus
gi|45535304|emb|CAE81332.1| protease and VPg Rice yellow mottle virus
gi|60098061|emb|CAI45862.1| protease and VPg Rice yellow mottle virus
gi|45535299|emb|CAE81328.1| protease and VPg Rice yellow mottle virus
gi|429138842|gb|AFZ75399.1| protease and VPg Rice yellow mottle virus
gi|198278694|emb|CAP03076.1| protease and VPg Rice yellow mottle virus
gi|198278699|emb|CAP03080.1| protease and VPg Rice yellow mottle virus
gi|212498720|ref|YP 002308435.1| polyprotein P2a Imperata yellow mottle virus
gi|855199074|gb|AKN81081.1| polyprotein P2a Cymbidium chlorotic spot virus
gi|827047239|ref|YP 009140473.1| polyprotein P2a Cymbidium chlorotic mosaic virus
gi|827047238|ref|YP 009140472.1| polyprotein P2a-P2b Cymbidium chlorotic mosaic virus
gi|385721371|gb|AFI72874.1| ORF2b Artemisia virus A
gi|387600884|ref|YP 006331062.1| polyprotein P2a Artemisia virus A
gi|456061298|ref|YP 006331061.2| polyprotein 2ab Artemisia virus A
gi|827882795|ref|YP 009142784.1| polyprotein P2a2b Rottboellia yellow mottle virus(2)
gi|827882797|ref|YP 009142785.1| polyprotein P2a Rottboellia yellow mottle virus(2)
gi|827882797|ref|YP 009142785.1| polyprotein P2a Rottboellia yellow mottle virus
gi|827882795|ref|YP 009142784.1| polyprotein P2a2b Rottboellia yellow mottle virus
gi|10566728|dbj|BAB15934.1| protease and RNA-dependent RNA polymerase Ryegrass mottle virus
gi|449265394|ref|YP 007438853.1| polyprotein P2ab Ryegrass mottle virus(2)
gi|94483294|gb|ABF22687.1| P2a Ryegrass mottle virus
gi|449265395|ref|YP 007438854.1| polyprotein P2a Ryegrass mottle virus
gi|449265394|ref|YP 007438853.1| polyprotein P2ab Ryegrass mottle virus
gi|455736652|ref|NP 941957.2| polyprotein P2ab Cocksfoot mottle virus
gi|38427386|ref|NP 941998.1| serine protease Cocksfoot mottle virus
gi|38349473|ref|NP 941376.1| polyprotein P2a Cocksfoot mottle virus
gi|10566733|dbj|BAB15938.1| protease Cocksfoot mottle virus
gi|1032304|gb|AAA87609.1| proteinase Cocksfoot mottle virus
gi|338819286|sp|Q89504.2|P2A CFMVN RecName: FullPolyprotein P2A Contains: RecName: FullN-terminal protein Contains: RecName: FullSerine protease Contains: RecName: FullVPg Contains: RecName: FullPuta8
gi|123871511|sp|Q0PW25.1|RDRP CFMVN RecName: FullReplicase polyprotein P2AB Contains: RecName: FullN-terminal protein Contains: RecName: FullSerine protease Contains: RecName: FullVPg Contains: RecNap
gi|225008504|gb|ACN78881.1| polyprotein Cocksfoot mottle virus
gi|225008506|gb|ACN78883.1| polyprotein Cocksfoot mottle virus
gi|332205607|gb|AEE36644.1| P2a Velvet tobacco mottle virus
gi|307346913|ref|YP 003896038.1| polyprotein P2a Velvet tobacco mottle virus
gi|455743243|ref|YP 003896039.2| polyprotein P2ab Velvet tobacco mottle virus
gi|451460284|ref|NP 042301.2| polyprotein P2a Southern cowpea mosaic virus
gi|511861|gb|AAA46565.1| ORF Southern cowpea mosaic virus
gi|459680255|ref|NP 042302.3| polyprotein P2ab Southern cowpea mosaic virus
gi|25014048|ref|NP 736597.1| serine protease Southern cowpea mosaic virus
gi|347762085|ref|YP 004869650.1| polyprotein P2a Soybean yellow common mosaic virus
gi|456061287|ref|YP 004869651.2| polyprotein P2ab Soybean yellow common mosaic virus
gi|478245402|ref|NP 066393.4| polyprotein P2ab Sesbania mosaic virus
gi|478245401|ref|NP 066392.4| polyprotein P2a Sesbania mosaic virus
gi|25013763|ref|NP 736591.1| serine protease Sesbania mosaic virus
gi|338817969|sp|O73564.2|P2A SBMVA RecName: FullPolyprotein P2A Contains: RecName: FullN-terminal protein Contains: RecName: FullSerine protease Contains: RecName: FullVPg Contains: RecName: FullPuta8
gi|3126828|gb|AAC15983.1| VPg-serine protease-RNA dependent RNA polymerase Southern bean mosaic virus
gi|3126833|gb|AAC15987.1| VPg-serine protease-RNA dependent RNA polymersase Southern bean mosaic virus
gi|471346281|ref|YP 007438857.2| polyprotein P2a Southern bean mosaic virus
gi|338817929|sp|O72157.2|RDRP SBMVA RecName: FullReplicase polyprotein P2AB Contains: RecName: FullN-terminal protein Contains: RecName: FullSerine protease Contains: RecName: FullVPg Contains: RecNap
gi|471346288|ref|YP 007652836.1| VPg protein Southern bean mosaic virus
gi|471346282|ref|YP 007438858.2| polyprotein P2ab Southern bean mosaic virus
gi|455742837|ref|YP 002158813.2| polyprotein P2ab Sowbane mosaic virus
gi|197690540|emb|CAP79625.1| P2a-P2b fusion protein Sowbane mosaic virus
gi|197914883|ref|YP 002158814.1| polyprotein P2a Sowbane mosaic virus
gi|290795353|gb|ADD64688.1| P2a Sowbane mosaic virus(2)
gi|290795353|gb|ADD64688.1| P2a Sowbane mosaic virus
gi|306486911|gb|ADM93281.1| ORF2 Sowbane mosaic virus
gi|469477272|gb|AAO24318.2| polyprotein Turnip rosette virus
gi|564293603|ref|YP 008869286.1| polyprotein P2ab Turnip rosette virus
gi|564293602|ref|YP 008869287.1| polyprotein P2a Turnip rosette virus
gi|469477272|gb|AAO24318.2| polyprotein Turnip rosette virus(2)
gi|564293602|ref|YP 008869287.1| polyprotein P2a Turnip rosette virus(2)
gi|440214902|gb|AGB93802.1| polyprotein P2a2b partial Turnip rosette virus
gi|564293603|ref|YP 008869286.1| polyprotein P2ab Turnip rosette virus(2)
gi|521048527|gb|AGP50167.1| polyprotein P2a Turnip rosette virus
gi|521048526|gb|AGP50166.1| polyprotein P2ab Turnip rosette virus
gi|521048526|gb|AGP50166.1| polyprotein P2ab Turnip rosette virus(2)
gi|521048527|gb|AGP50167.1| polyprotein P2a Turnip rosette virus(2)
1G-010
gi|1041716|gb|AAA79988.1| polyprotein Lucerne transient streak virus
gi|449265364|ref|YP 007438849.1| polyprotein P2ab Lucerne transient streak virus
gi|449265362|ref|YP 007438850.1| polyprotein P2a Lucerne transient streak virus
gi|456061308|ref|YP 006589925.2| polyprotein P2ab Papaya lethal yellowing virus
gi|401715655|ref|YP 006589926.1| polyprotein P2a Papaya lethal yellowing virus
gi|400131037|gb|AFP67700.1| RNA-dependent RNA polymerase Papaya lethal yellowing virus
gi|39843096|gb|AAR32107.1| P2a/2b Subterranean clover mottle virus(2)
gi|39843097|gb|AAR32108.1| P2a Subterranean clover mottle virus
gi|39843096|gb|AAR32107.1| P2a/2b Subterranean clover mottle virus
gi|39843101|gb|AAR32111.1| P2a/2b Subterranean clover mottle virus(2)
gi|39843091|gb|AAR32103.1| P2a/2b Subterranean clover mottle virus(2)
gi|24210503|gb|AAN51929.1| polyprotein Subterranean clover mottle virus(2)
gi|455742834|ref|NP 715627.2| polyprotein P2ab Subterranean clover mottle virus(2)
gi|39843106|gb|AAR32115.1| P2a/2b Subterranean clover mottle virus(2)
gi|39843092|gb|AAR32104.1| P2a Subterranean clover mottle virus(2)
gi|25013760|ref|NP 736579.1| serine protease Subterranean clover mottle virus(2)
gi|39843102|gb|AAR32112.1| P2a Subterranean clover mottle virus(2)
gi|39843092|gb|AAR32104.1| P2a Subterranean clover mottle virus
gi|39843102|gb|AAR32112.1| P2a Subterranean clover mottle virus
gi|24323603|ref|NP 715628.1| polyprotein P2a Subterranean clover mottle virus
gi|39843107|gb|AAR32116.1| P2a Subterranean clover mottle virus
gi|39843101|gb|AAR32111.1| P2a/2b Subterranean clover mottle virus
gi|39843091|gb|AAR32103.1| P2a/2b Subterranean clover mottle virus
gi|455742834|ref|NP 715627.2| polyprotein P2ab Subterranean clover mottle virus
gi|24210503|gb|AAN51929.1| polyprotein Subterranean clover mottle virus
gi|39843106|gb|AAR32115.1| P2a/2b Subterranean clover mottle virus
gi|25013760|ref|NP 736579.1| serine protease Subterranean clover mottle virus
0.2

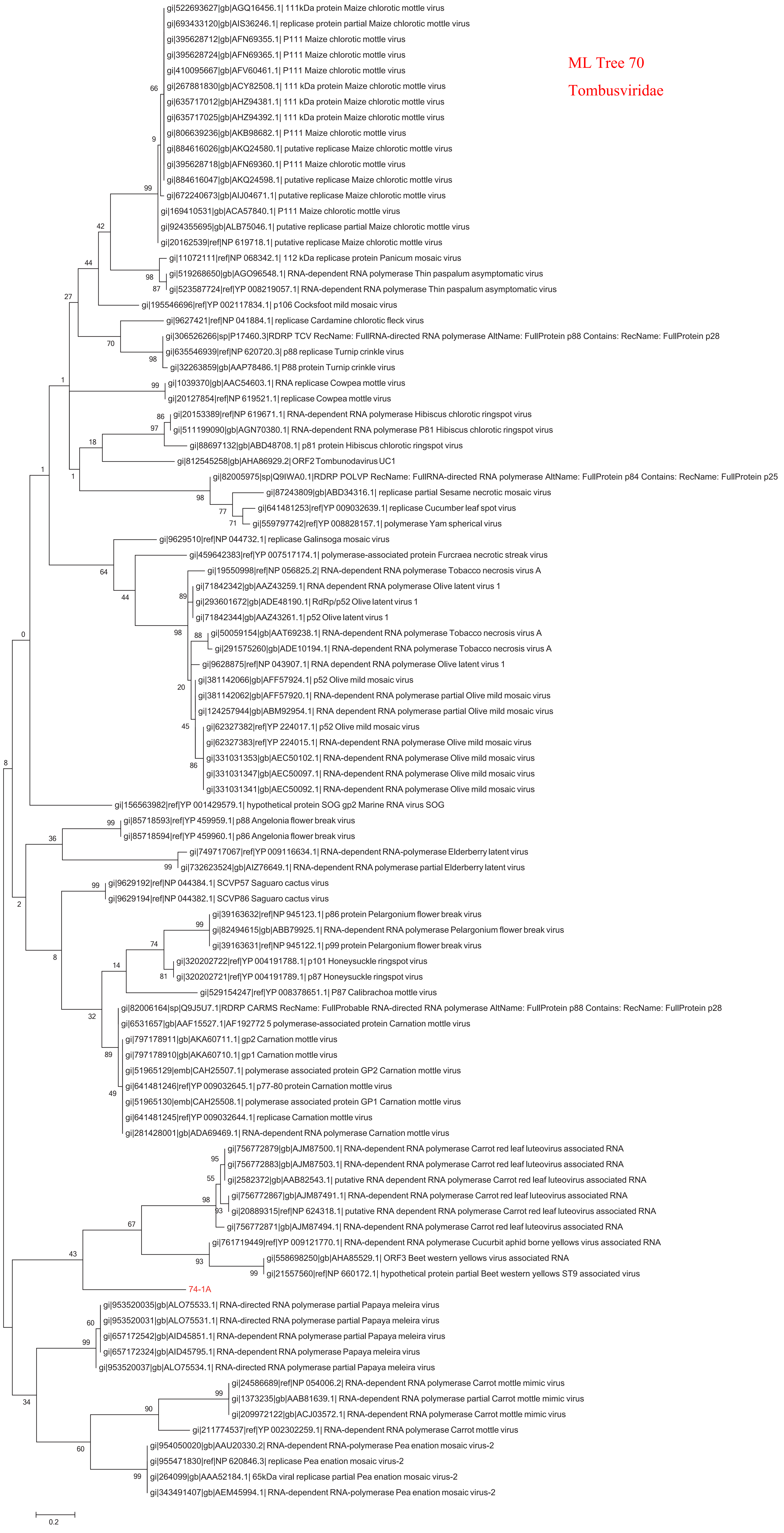
ML Tree 70
Tombusviridae
gi|522693627|gb|AGQ16456.1| 111kDa protein Maize chlorotic mottle virus
gi|693433120|gb|AIS36246.1| replicase protein partial Maize chlorotic mottle virus
gi|395628712|gb|AFN69355.1| P111 Maize chlorotic mottle virus
gi|395628724|gb|AFN69365.1| P111 Maize chlorotic mottle virus
gi|410095667|gb|AFV60461.1| P111 Maize chlorotic mottle virus
gi|267881830|gb|ACY82508.1| 111 kDa protein Maize chlorotic mottle virus
gi|635717012|gb|AHZ94381.1| 111 kDa protein Maize chlorotic mottle virus
gi|635717025|gb|AHZ94392.1| 111 kDa protein Maize chlorotic mottle virus
gi|806639236|gb|AKB98682.1| P111 Maize chlorotic mottle virus
gi|884616026|gb|AKQ24580.1| putative replicase Maize chlorotic mottle virus
gi|395628718|gb|AFN69360.1| P111 Maize chlorotic mottle virus
gi|884616047|gb|AKQ24598.1| putative replicase Maize chlorotic mottle virus
gi|672240673|gb|AIJ04671.1| putative replicase Maize chlorotic mottle virus
gi|169410531|gb|ACA57840.1| P111 Maize chlorotic mottle virus
gi|924355695|gb|ALB75046.1| putative replicase partial Maize chlorotic mottle virus
gi|20162539|ref|NP 619718.1| putative replicase Maize chlorotic mottle virus
gi|11072111|ref|NP 068342.1| 112 kDa replicase protein Panicum mosaic virus
gi|519268650|gb|AGO96548.1| RNA-dependent RNA polymerase Thin paspalum asymptomatic virus
gi|523587724|ref|YP 008219057.1| RNA-dependent RNA polymerase Thin paspalum asymptomatic virus
gi|195546696|ref|YP 002117834.1| p106 Cocksfoot mild mosaic virus
gi|9627421|ref|NP 041884.1| replicase Cardamine chlorotic fleck virus
gi|306526266|sp|P17460.3|RDRP TCV RecName: FullRNA-directed RNA polymerase AltName: FullProtein p88 Contains: RecName: FullProtein p28
gi|635546939|ref|NP 620720.3| p88 replicase Turnip crinkle virus
gi|32263859|gb|AAP78486.1| P88 protein Turnip crinkle virus
gi|1039370|gb|AAC54603.1| RNA replicase Cowpea mottle virus
gi|20127854|ref|NP 619521.1| replicase Cowpea mottle virus
gi|20153389|ref|NP 619671.1| RNA-dependent RNA polymerase Hibiscus chlorotic ringspot virus
gi|511199090|gb|AGN70380.1| RNA-dependent RNA polymerase P81 Hibiscus chlorotic ringspot virus
gi|88697132|gb|ABD48708.1| p81 protein Hibiscus chlorotic ringspot virus
gi|812545258|gb|AHA86929.2| ORF2 Tombunodavirus UC1
gi|82005975|sp|Q9IWA0.1|RDRP POLVP RecName: FullRNA-directed RNA polymerase AltName: FullProtein p84 Contains: RecName: FullProtein p25
gi|87243809|gb|ABD34316.1| replicase partial Sesame necrotic mosaic virus
gi|641481253|ref|YP 009032639.1| replicase Cucumber leaf spot virus
gi|559797742|ref|YP 008828157.1| polymerase Yam spherical virus
gi|9629510|ref|NP 044732.1| replicase Galinsoga mosaic virus
gi|459642383|ref|YP 007517174.1| polymerase-associated protein Furcraea necrotic streak virus
gi|19550998|ref|NP 056825.2| RNA-dependent RNA polymerase Tobacco necrosis virus A
gi|71842342|gb|AAZ43259.1| RNA dependent RNA polymerase Olive latent virus 1
gi|293601672|gb|ADE48190.1| RdRp/p52 Olive latent virus 1
gi|71842344|gb|AAZ43261.1| p52 Olive latent virus 1
gi|50059154|gb|AAT69238.1| RNA-dependent RNA polymerase Tobacco necrosis virus A
gi|291575260|gb|ADE10194.1| RNA-dependent RNA polymerase Tobacco necrosis virus A
gi|9628875|ref|NP 043907.1| RNA dependent RNA polymerase Olive latent virus 1
gi|381142066|gb|AFF57924.1| p52 Olive mild mosaic virus
gi|381142062|gb|AFF57920.1| RNA-dependent RNA polymerase partial Olive mild mosaic virus
gi|124257944|gb|ABM92954.1| RNA dependent RNA polymerase partial Olive mild mosaic virus
gi|62327382|ref|YP 224017.1| p52 Olive mild mosaic virus
gi|62327383|ref|YP 224015.1| RNA-dependent RNA polymerase Olive mild mosaic virus
gi|331031353|gb|AEC50102.1| RNA-dependent RNA polymerase Olive mild mosaic virus
gi|331031347|gb|AEC50097.1| RNA-dependent RNA polymerase Olive mild mosaic virus
gi|331031341|gb|AEC50092.1| RNA-dependent RNA polymerase Olive mild mosaic virus
gi|156563982|ref|YP 001429579.1| hypothetical protein SOG gp2 Marine RNA virus SOG
gi|85718593|ref|YP 459959.1| p88 Angelonia flower break virus
gi|85718594|ref|YP 459960.1| p86 Angelonia flower break virus
gi|749717067|ref|YP 009116634.1| RNA-dependent RNA-polymerase Elderberry latent virus
gi|732623524|gb|AIZ76649.1| RNA-dependent RNA polymerase partial Elderberry latent virus
gi|9629192|ref|NP 044384.1| SCVP57 Saguaro cactus virus
gi|9629194|ref|NP 044382.1| SCVP86 Saguaro cactus virus
gi|39163632|ref|NP 945123.1| p86 protein Pelargonium flower break virus
gi|82494615|gb|ABB79925.1| RNA-dependent RNA polymerase Pelargonium flower break virus
gi|39163631|ref|NP 945122.1| p99 protein Pelargonium flower break virus
gi|320202722|ref|YP 004191788.1| p101 Honeysuckle ringspot virus
gi|320202721|ref|YP 004191789.1| p87 Honeysuckle ringspot virus
gi|529154247|ref|YP 008378651.1| P87 Calibrachoa mottle virus
gi|82006164|sp|Q9J5U7.1|RDRP CARMS RecName: FullProbable RNA-directed RNA polymerase AltName: FullProtein p88 Contains: RecName: FullProtein p28
gi|6531657|gb|AAF15527.1|AF192772 5 polymerase-associated protein Carnation mottle virus
gi|797178911|gb|AKA60711.1| gp2 Carnation mottle virus
gi|797178910|gb|AKA60710.1| gp1 Carnation mottle virus
gi|51965129|emb|CAH25507.1| polymerase associated protein GP2 Carnation mottle virus
gi|641481246|ref|YP 009032645.1| p77-80 protein Carnation mottle virus
gi|51965130|emb|CAH25508.1| polymerase associated protein GP1 Carnation mottle virus
gi|641481245|ref|YP 009032644.1| replicase Carnation mottle virus
gi|281428001|gb|ADA69469.1| RNA-dependent RNA polymerase Carnation mottle virus
gi|756772879|gb|AJM87500.1| RNA-dependent RNA polymerase Carrot red leaf luteovirus associated RNA
gi|756772883|gb|AJM87503.1| RNA-dependent RNA polymerase Carrot red leaf luteovirus associated RNA
gi|2582372|gb|AAB82543.1| putative RNA dependent RNA polymerase Carrot red leaf luteovirus associated RNA
gi|756772867|gb|AJM87491.1| RNA-dependent RNA polymerase Carrot red leaf luteovirus associated RNA
gi|20889315|ref|NP 624318.1| putative RNA dependent RNA polymerase Carrot red leaf luteovirus associated RNA
gi|756772871|gb|AJM87494.1| RNA-dependent RNA polymerase Carrot red leaf luteovirus associated RNA
gi|761719449|ref|YP 009121770.1| RNA-dependent RNA polymerase Cucurbit aphid borne yellows virus associated RNA
gi|558698250|gb|AHA85529.1| ORF3 Beet western yellows virus associated RNA
gi|21557560|ref|NP 660172.1| hypothetical protein partial Beet western yellows ST9 associated virus
74-1A
gi|953520035|gb|ALO75533.1| RNA-directed RNA polymerase partial Papaya meleira virus
gi|953520031|gb|ALO75531.1| RNA-directed RNA polymerase partial Papaya meleira virus
gi|657172542|gb|AID45851.1| RNA-dependent RNA polymerase partial Papaya meleira virus
gi|657172324|gb|AID45795.1| RNA-dependent RNA polymerase Papaya meleira virus
gi|953520037|gb|ALO75534.1| RNA-directed RNA polymerase partial Papaya meleira virus
gi|24586689|ref|NP 054006.2| RNA-dependent RNA polymerase Carrot mottle mimic virus
gi|1373235|gb|AAB81639.1| RNA-dependent RNA polymerase partial Carrot mottle mimic virus
gi|209972122|gb|ACJ03572.1| RNA-dependent RNA polymerase Carrot mottle mimic virus
gi|211774537|ref|YP 002302259.1| RNA-dependent RNA polymerase Carrot mottle virus
gi|954050020|gb|AAU20330.2| RNA-dependent RNA-polymerase Pea enation mosaic virus-2
gi|955471830|ref|NP 620846.3| replicase Pea enation mosaic virus-2
gi|264099|gb|AAA52184.1| 65kDa viral replicase partial Pea enation mosaic virus-2
gi|343491407|gb|AEM45994.1| RNA-dependent RNA-polymerase Pea enation mosaic virus-2
0.2

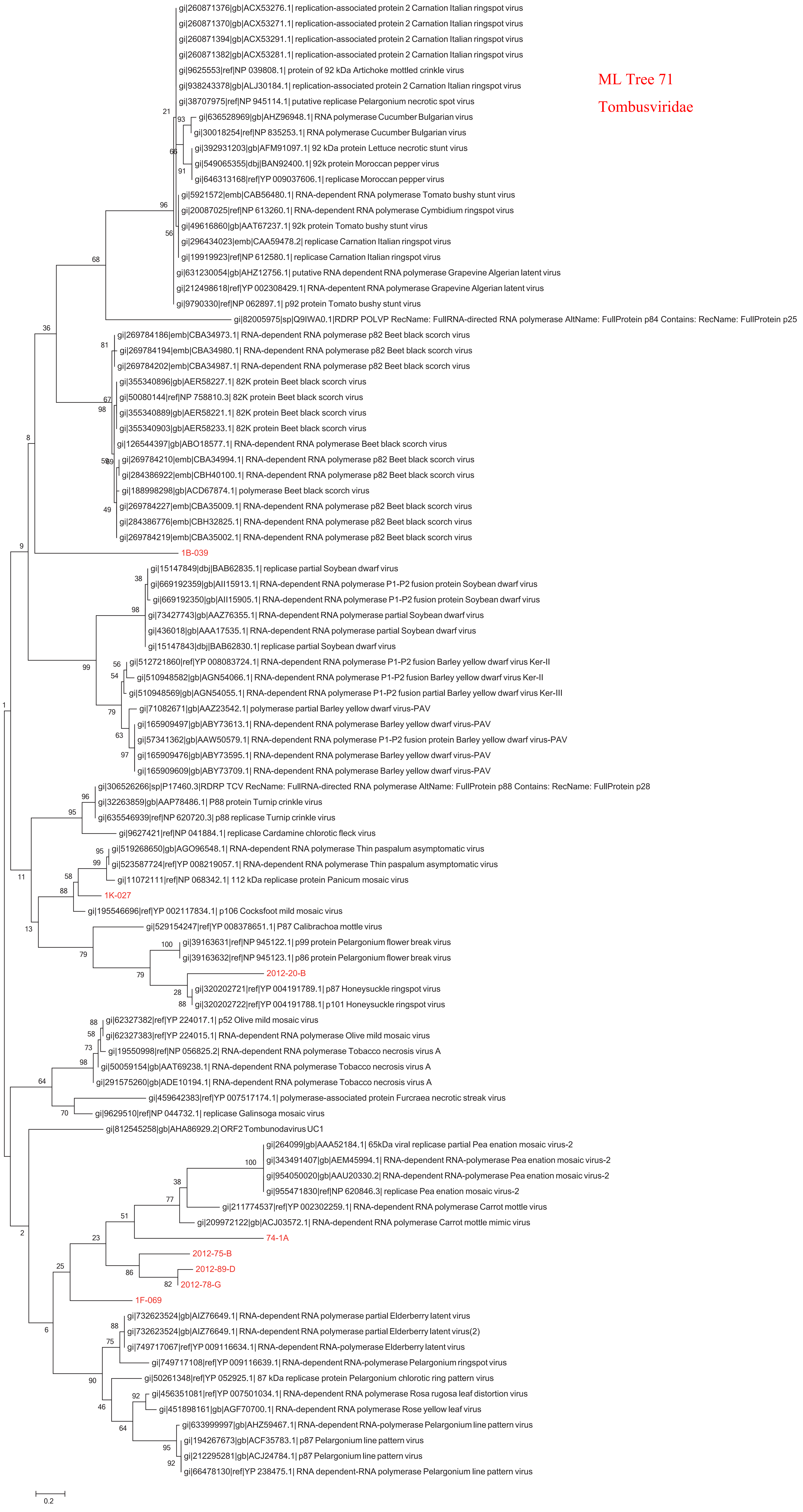

ML Tree 71
Tombusviridae
gi|260871376|gb|ACX53276.1| replication-associated protein 2 Carnation Italian ringspot virus
gi|260871370|gb|ACX53271.1| replication-associated protein 2 Carnation Italian ringspot virus
gi|260871394|gb|ACX53291.1| replication-associated protein 2 Carnation Italian ringspot virus
gi|260871382|gb|ACX53281.1| replication-associated protein 2 Carnation Italian ringspot virus
gi|9625553|ref|NP 039808.1| protein of 92 kDa Artichoke mottled crinkle virus
gi|938243378|gb|ALJ30184.1| replication-associated protein 2 Carnation Italian ringspot virus
gi|38707975|ref|NP 945114.1| putative replicase Pelargonium necrotic spot virus
gi|636528969|gb|AHZ96948.1| RNA polymerase Cucumber Bulgarian virus
gi|30018254|ref|NP 835253.1| RNA polymerase Cucumber Bulgarian virus
gi|392931203|gb|AFM91097.1| 92 kDa protein Lettuce necrotic stunt virus
gi|549065355|dbj|BAN92400.1| 92k protein Moroccan pepper virus
gi|646313168|ref|YP 009037606.1| replicase Moroccan pepper virus
gi|5921572|emb|CAB56480.1| RNA-dependent RNA polymerase Tomato bushy stunt virus
gi|20087025|ref|NP 613260.1| RNA-dependent RNA polymerase Cymbidium ringspot virus
gi|49616860|gb|AAT67237.1| 92k protein Tomato bushy stunt virus
gi|296434023|emb|CAA59478.2| replicase Carnation Italian ringspot virus
gi|19919923|ref|NP 612580.1| replicase Carnation Italian ringspot virus
gi|631230054|gb|AHZ12756.1| putative RNA dependent RNA polymerase Grapevine Algerian latent virus
gi|212498618|ref|YP 002308429.1| RNA-dependent RNA polymerase Grapevine Algerian latent virus
gi|9790330|ref|NP 062897.1| p92 protein Tomato bushy stunt virus
gi|82005975|sp|Q9IWA0.1|RDRP POLVP RecName: FullRNA-directed RNA polymerase AltName: FullProtein p84 Contains: RecName: FullProtein p25
gi|269784186|emb|CBA34973.1| RNA-dependent RNA polymerase p82 Beet black scorch virus
gi|269784194|emb|CBA34980.1| RNA-dependent RNA polymerase p82 Beet black scorch virus
gi|269784202|emb|CBA34987.1| RNA-dependent RNA polymerase p82 Beet black scorch virus
gi|355340896|gb|AER58227.1| 82K protein Beet black scorch virus
gi|50080144|ref|NP 758810.3| 82K protein Beet black scorch virus
gi|355340889|gb|AER58221.1| 82K protein Beet black scorch virus
gi|355340903|gb|AER58233.1| 82K protein Beet black scorch virus
gi|126544397|gb|ABO18577.1| RNA-dependent RNA polymerase Beet black scorch virus
gi|269784210|emb|CBA34994.1| RNA-dependent RNA polymerase p82 Beet black scorch virus
gi|284386922|emb|CBH40100.1| RNA-dependent RNA polymerase p82 Beet black scorch virus
gi|188998298|gb|ACD67874.1| polymerase Beet black scorch virus
gi|269784227|emb|CBA35009.1| RNA-dependent RNA polymerase p82 Beet black scorch virus
gi|284386776|emb|CBH32825.1| RNA-dependent RNA polymerase p82 Beet black scorch virus
gi|269784219|emb|CBA35002.1| RNA-dependent RNA polymerase p82 Beet black scorch virus
1B-039
gi|15147849|dbj|BAB62835.1| replicase partial Soybean dwarf virus
gi|669192359|gb|AII15913.1| RNA-dependent RNA polymerase P1-P2 fusion protein Soybean dwarf virus
gi|669192350|gb|AII15905.1| RNA-dependent RNA polymerase P1-P2 fusion protein Soybean dwarf virus
gi|73427743|gb|AAZ76355.1| RNA-dependent RNA polymerase partial Soybean dwarf virus
gi|436018|gb|AAA17535.1| RNA-dependent RNA polymerase partial Soybean dwarf virus
gi|15147843|dbj|BAB62830.1| replicase partial Soybean dwarf virus
gi|512721860|ref|YP 008083724.1| RNA-dependent RNA polymerase P1-P2 fusion Barley yellow dwarf virus Ker-II
gi|510948582|gb|AGN54066.1| RNA-dependent RNA polymerase P1-P2 fusion Barley yellow dwarf virus Ker-II
gi|510948569|gb|AGN54055.1| RNA-dependent RNA polymerase P1-P2 fusion partial Barley yellow dwarf virus Ker-III
gi|71082671|gb|AAZ23542.1| polymerase partial Barley yellow dwarf virus-PAV
gi|165909497|gb|ABY73613.1| RNA-dependent RNA polymerase Barley yellow dwarf virus-PAV
gi|57341362|gb|AAW50579.1| RNA-dependent RNA polymerase P1-P2 fusion protein Barley yellow dwarf virus-PAV
gi|165909476|gb|ABY73595.1| RNA-dependent RNA polymerase Barley yellow dwarf virus-PAV
gi|165909609|gb|ABY73709.1| RNA-dependent RNA polymerase Barley yellow dwarf virus-PAV
gi|306526266|sp|P17460.3|RDRP TCV RecName: FullRNA-directed RNA polymerase AltName: FullProtein p88 Contains: RecName: FullProtein p28
gi|32263859|gb|AAP78486.1| P88 protein Turnip crinkle virus
gi|635546939|ref|NP 620720.3| p88 replicase Turnip crinkle virus
gi|9627421|ref|NP 041884.1| replicase Cardamine chlorotic fleck virus
gi|519268650|gb|AGO96548.1| RNA-dependent RNA polymerase Thin paspalum asymptomatic virus
gi|523587724|ref|YP 008219057.1| RNA-dependent RNA polymerase Thin paspalum asymptomatic virus
gi|11072111|ref|NP 068342.1| 112 kDa replicase protein Panicum mosaic virus
1K-027
gi|195546696|ref|YP 002117834.1| p106 Cocksfoot mild mosaic virus
gi|529154247|ref|YP 008378651.1| P87 Calibrachoa mottle virus
gi|39163631|ref|NP 945122.1| p99 protein Pelargonium flower break virus
gi|39163632|ref|NP 945123.1| p86 protein Pelargonium flower break virus
2012-20-B
gi|320202721|ref|YP 004191789.1| p87 Honeysuckle ringspot virus
gi|320202722|ref|YP 004191788.1| p101 Honeysuckle ringspot virus
gi|62327382|ref|YP 224017.1| p52 Olive mild mosaic virus
gi|62327383|ref|YP 224015.1| RNA-dependent RNA polymerase Olive mild mosaic virus
gi|19550998|ref|NP 056825.2| RNA-dependent RNA polymerase Tobacco necrosis virus A
gi|50059154|gb|AAT69238.1| RNA-dependent RNA polymerase Tobacco necrosis virus A
gi|291575260|gb|ADE10194.1| RNA-dependent RNA polymerase Tobacco necrosis virus A
gi|459642383|ref|YP 007517174.1| polymerase-associated protein Furcraea necrotic streak virus
gi|9629510|ref|NP 044732.1| replicase Galinsoga mosaic virus
gi|812545258|gb|AHA86929.2| ORF2 Tombunodavirus UC1
gi|264099|gb|AAA52184.1| 65kDa viral replicase partial Pea enation mosaic virus-2
gi|343491407|gb|AEM45994.1| RNA-dependent RNA-polymerase Pea enation mosaic virus-2
gi|954050020|gb|AAU20330.2| RNA-dependent RNA-polymerase Pea enation mosaic virus-2
gi|955471830|ref|NP 620846.3| replicase Pea enation mosaic virus-2
gi|211774537|ref|YP 002302259.1| RNA-dependent RNA polymerase Carrot mottle virus
gi|209972122|gb|ACJ03572.1| RNA-dependent RNA polymerase Carrot mottle mimic virus
74-1A
2012-75-B
2012-89-D
2012-78-G
1F-069
gi|732623524|gb|AIZ76649.1| RNA-dependent RNA polymerase partial Elderberry latent virus
gi|732623524|gb|AIZ76649.1| RNA-dependent RNA polymerase partial Elderberry latent virus(2)
gi|749717067|ref|YP 009116634.1| RNA-dependent RNA-polymerase Elderberry latent virus
gi|749717108|ref|YP 009116639.1| RNA-dependent RNA-polymerase Pelargonium ringspot virus
gi|50261348|ref|YP 052925.1| 87 kDa replicase protein Pelargonium chlorotic ring pattern virus
gi|456351081|ref|YP 007501034.1| RNA-dependent RNA polymerase Rosa rugosa leaf distortion virus
gi|451898161|gb|AGF70700.1| RNA-dependent RNA polymerase Rose yellow leaf virus
gi|633999997|gb|AHZ59467.1| RNA-dependent RNA-polymerase Pelargonium line pattern virus
gi|194267673|gb|ACF35783.1| p87 Pelargonium line pattern virus
gi|212295281|gb|ACJ24784.1| p87 Pelargonium line pattern virus
gi|66478130|ref|YP 238475.1| RNA dependent-RNA polymerase Pelargonium line pattern virus
0.2

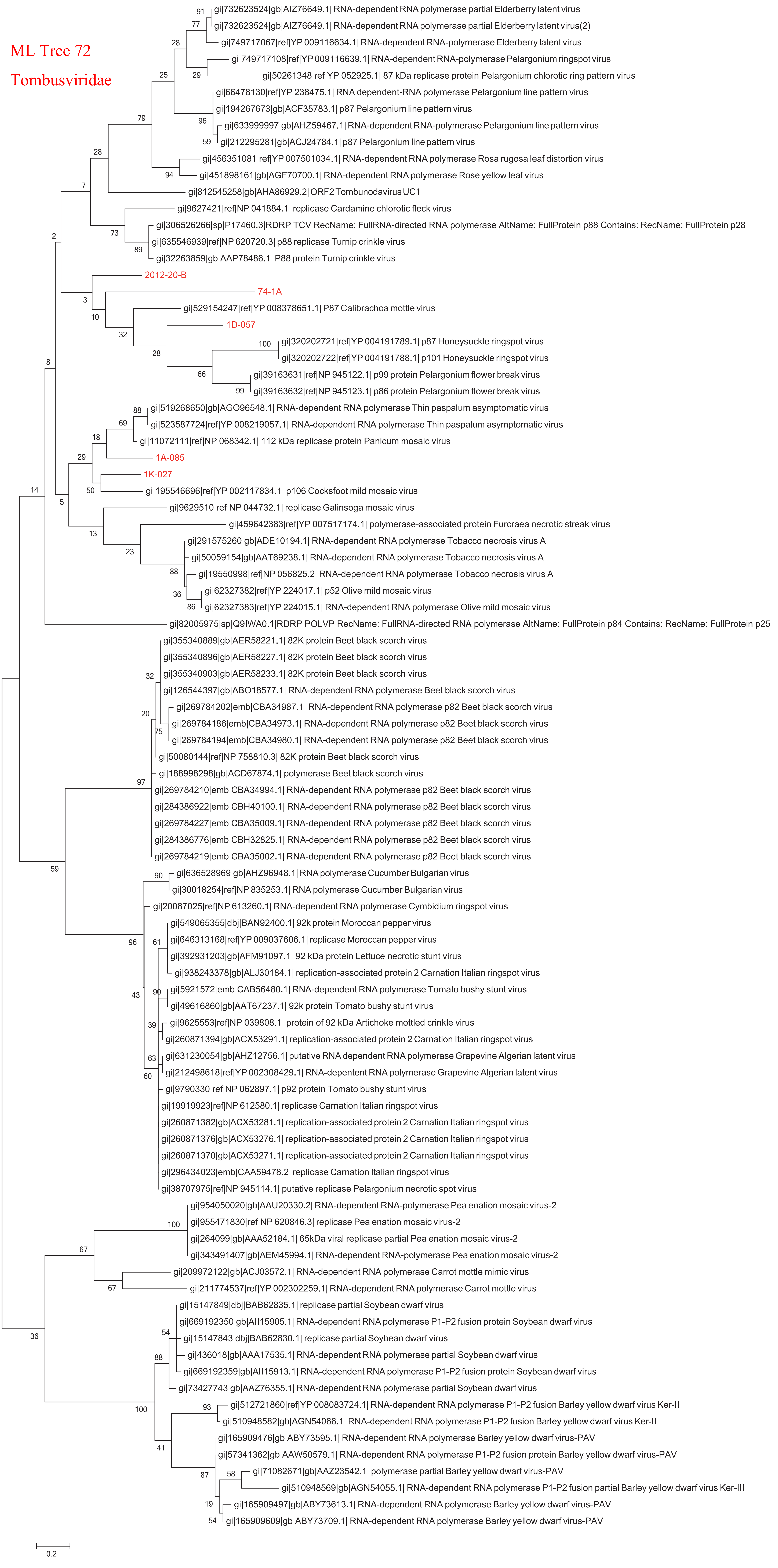
ML Tree 72
Tombusviridae
gi|732623524|gb|AIZ76649.1| RNA-dependent RNA polymerase partial Elderberry latent virus
gi|732623524|gb|AIZ76649.1| RNA-dependent RNA polymerase partial Elderberry latent virus(2)
gi|749717067|ref|YP 009116634.1| RNA-dependent RNA-polymerase Elderberry latent virus
gi|749717108|ref|YP 009116639.1| RNA-dependent RNA-polymerase Pelargonium ringspot virus
gi|50261348|ref|YP 052925.1| 87 kDa replicase protein Pelargonium chlorotic ring pattern virus
gi|66478130|ref|YP 238475.1| RNA dependent-RNA polymerase Pelargonium line pattern virus
gi|194267673|gb|ACF35783.1| p87 Pelargonium line pattern virus
gi|633999997|gb|AHZ59467.1| RNA-dependent RNA-polymerase Pelargonium line pattern virus
gi|212295281|gb|ACJ24784.1| p87 Pelargonium line pattern virus
gi|456351081|ref|YP 007501034.1| RNA-dependent RNA polymerase Rosa rugosa leaf distortion virus
gi|451898161|gb|AGF70700.1| RNA-dependent RNA polymerase Rose yellow leaf virus
gi|812545258|gb|AHA86929.2| ORF2 Tombunodavirus UC1
gi|9627421|ref|NP 041884.1| replicase Cardamine chlorotic fleck virus
gi|306526266|sp|P17460.3|RDRP TCV RecName: FullRNA-directed RNA polymerase AltName: FullProtein p88 Contains: RecName: FullProtein p28
gi|635546939|ref|NP 620720.3| p88 replicase Turnip crinkle virus
gi|32263859|gb|AAP78486.1| P88 protein Turnip crinkle virus
2012-20-B
74-1A
gi|529154247|ref|YP 008378651.1| P87 Calibrachoa mottle virus
1D-057
gi|320202721|ref|YP 004191789.1| p87 Honeysuckle ringspot virus
gi|320202722|ref|YP 004191788.1| p101 Honeysuckle ringspot virus
gi|39163631|ref|NP 945122.1| p99 protein Pelargonium flower break virus
gi|39163632|ref|NP 945123.1| p86 protein Pelargonium flower break virus
gi|519268650|gb|AGO96548.1| RNA-dependent RNA polymerase Thin paspalum asymptomatic virus
gi|523587724|ref|YP 008219057.1| RNA-dependent RNA polymerase Thin paspalum asymptomatic virus
gi|11072111|ref|NP 068342.1| 112 kDa replicase protein Panicum mosaic virus
1A-085
1K-027
gi|195546696|ref|YP 002117834.1| p106 Cocksfoot mild mosaic virus
gi|9629510|ref|NP 044732.1| replicase Galinsoga mosaic virus
gi|459642383|ref|YP 007517174.1| polymerase-associated protein Furcraea necrotic streak virus
gi|291575260|gb|ADE10194.1| RNA-dependent RNA polymerase Tobacco necrosis virus A
gi|50059154|gb|AAT69238.1| RNA-dependent RNA polymerase Tobacco necrosis virus A
gi|19550998|ref|NP 056825.2| RNA-dependent RNA polymerase Tobacco necrosis virus A
gi|62327382|ref|YP 224017.1| p52 Olive mild mosaic virus
gi|62327383|ref|YP 224015.1| RNA-dependent RNA polymerase Olive mild mosaic virus
gi|82005975|sp|Q9IWA0.1|RDRP POLVP RecName: FullRNA-directed RNA polymerase AltName: FullProtein p84 Contains: RecName: FullProtein p25
gi|355340889|gb|AER58221.1| 82K protein Beet black scorch virus
gi|355340896|gb|AER58227.1| 82K protein Beet black scorch virus
gi|355340903|gb|AER58233.1| 82K protein Beet black scorch virus
gi|126544397|gb|ABO18577.1| RNA-dependent RNA polymerase Beet black scorch virus
gi|269784202|emb|CBA34987.1| RNA-dependent RNA polymerase p82 Beet black scorch virus
gi|269784186|emb|CBA34973.1| RNA-dependent RNA polymerase p82 Beet black scorch virus
gi|269784194|emb|CBA34980.1| RNA-dependent RNA polymerase p82 Beet black scorch virus
gi|50080144|ref|NP 758810.3| 82K protein Beet black scorch virus
gi|188998298|gb|ACD67874.1| polymerase Beet black scorch virus
gi|269784210|emb|CBA34994.1| RNA-dependent RNA polymerase p82 Beet black scorch virus
gi|284386922|emb|CBH40100.1| RNA-dependent RNA polymerase p82 Beet black scorch virus
gi|269784227|emb|CBA35009.1| RNA-dependent RNA polymerase p82 Beet black scorch virus
gi|284386776|emb|CBH32825.1| RNA-dependent RNA polymerase p82 Beet black scorch virus
gi|269784219|emb|CBA35002.1| RNA-dependent RNA polymerase p82 Beet black scorch virus
gi|636528969|gb|AHZ96948.1| RNA polymerase Cucumber Bulgarian virus
gi|30018254|ref|NP 835253.1| RNA polymerase Cucumber Bulgarian virus
gi|20087025|ref|NP 613260.1| RNA-dependent RNA polymerase Cymbidium ringspot virus
gi|549065355|dbj|BAN92400.1| 92k protein Moroccan pepper virus
gi|646313168|ref|YP 009037606.1| replicase Moroccan pepper virus
gi|392931203|gb|AFM91097.1| 92 kDa protein Lettuce necrotic stunt virus
gi|938243378|gb|ALJ30184.1| replication-associated protein 2 Carnation Italian ringspot virus
gi|5921572|emb|CAB56480.1| RNA-dependent RNA polymerase Tomato bushy stunt virus
gi|49616860|gb|AAT67237.1| 92k protein Tomato bushy stunt virus
gi|9625553|ref|NP 039808.1| protein of 92 kDa Artichoke mottled crinkle virus
gi|260871394|gb|ACX53291.1| replication-associated protein 2 Carnation Italian ringspot virus
gi|631230054|gb|AHZ12756.1| putative RNA dependent RNA polymerase Grapevine Algerian latent virus
gi|212498618|ref|YP 002308429.1| RNA-depentent RNA polymerase Grapevine Algerian latent virus
gi|9790330|ref|NP 062897.1| p92 protein Tomato bushy stunt virus
gi|19919923|ref|NP 612580.1| replicase Carnation Italian ringspot virus
gi|260871382|gb|ACX53281.1| replication-associated protein 2 Carnation Italian ringspot virus
gi|260871376|gb|ACX53276.1| replication-associated protein 2 Carnation Italian ringspot virus
gi|260871370|gb|ACX53271.1| replication-associated protein 2 Carnation Italian ringspot virus
gi|296434023|emb|CAA59478.2| replicase Carnation Italian ringspot virus
gi|38707975|ref|NP 945114.1| putative replicase Pelargonium necrotic spot virus
gi|954050020|gb|AAU20330.2| RNA-dependent RNA-polymerase Pea enation mosaic virus-2
gi|955471830|ref|NP 620846.3| replicase Pea enation mosaic virus-2
gi|264099|gb|AAA52184.1| 65kDa viral replicase partial Pea enation mosaic virus-2
gi|343491407|gb|AEM45994.1| RNA-dependent RNA-polymerase Pea enation mosaic virus-2
gi|209972122|gb|ACJ03572.1| RNA-dependent RNA polymerase Carrot mottle mimic virus
gi|211774537|ref|YP 002302259.1| RNA-dependent RNA polymerase Carrot mottle virus
gi|15147849|dbj|BAB62835.1| replicase partial Soybean dwarf virus
gi|669192350|gb|AII15905.1| RNA-dependent RNA polymerase P1-P2 fusion protein Soybean dwarf virus
gi|15147843|dbj|BAB62830.1| replicase partial Soybean dwarf virus
gi|436018|gb|AAA17535.1| RNA-dependent RNA polymerase partial Soybean dwarf virus
gi|669192359|gb|AII15913.1| RNA-dependent RNA polymerase P1-P2 fusion protein Soybean dwarf virus
gi|73427743|gb|AAZ76355.1| RNA-dependent RNA polymerase partial Soybean dwarf virus
gi|512721860|ref|YP 008083724.1| RNA-dependent RNA polymerase P1-P2 fusion Barley yellow dwarf virus Ker-II
gi|510948582|gb|AGN54066.1| RNA-dependent RNA polymerase P1-P2 fusion Barley yellow dwarf virus Ker-II
gi|165909476|gb|ABY73595.1| RNA-dependent RNA polymerase Barley yellow dwarf virus-PAV
gi|57341362|gb|AAW50579.1| RNA-dependent RNA polymerase P1-P2 fusion protein Barley yellow dwarf virus-PAV
gi|71082671|gb|AAZ23542.1| polymerase partial Barley yellow dwarf virus-PAV
gi|510948569|gb|AGN54055.1| RNA-dependent RNA polymerase P1-P2 fusion partial Barley yellow dwarf virus Ker-III
gi|165909497|gb|ABY73613.1| RNA-dependent RNA polymerase Barley yellow dwarf virus-PAV
gi|165909609|gb|ABY73709.1| RNA-dependent RNA polymerase Barley yellow dwarf virus-PAV
0.2

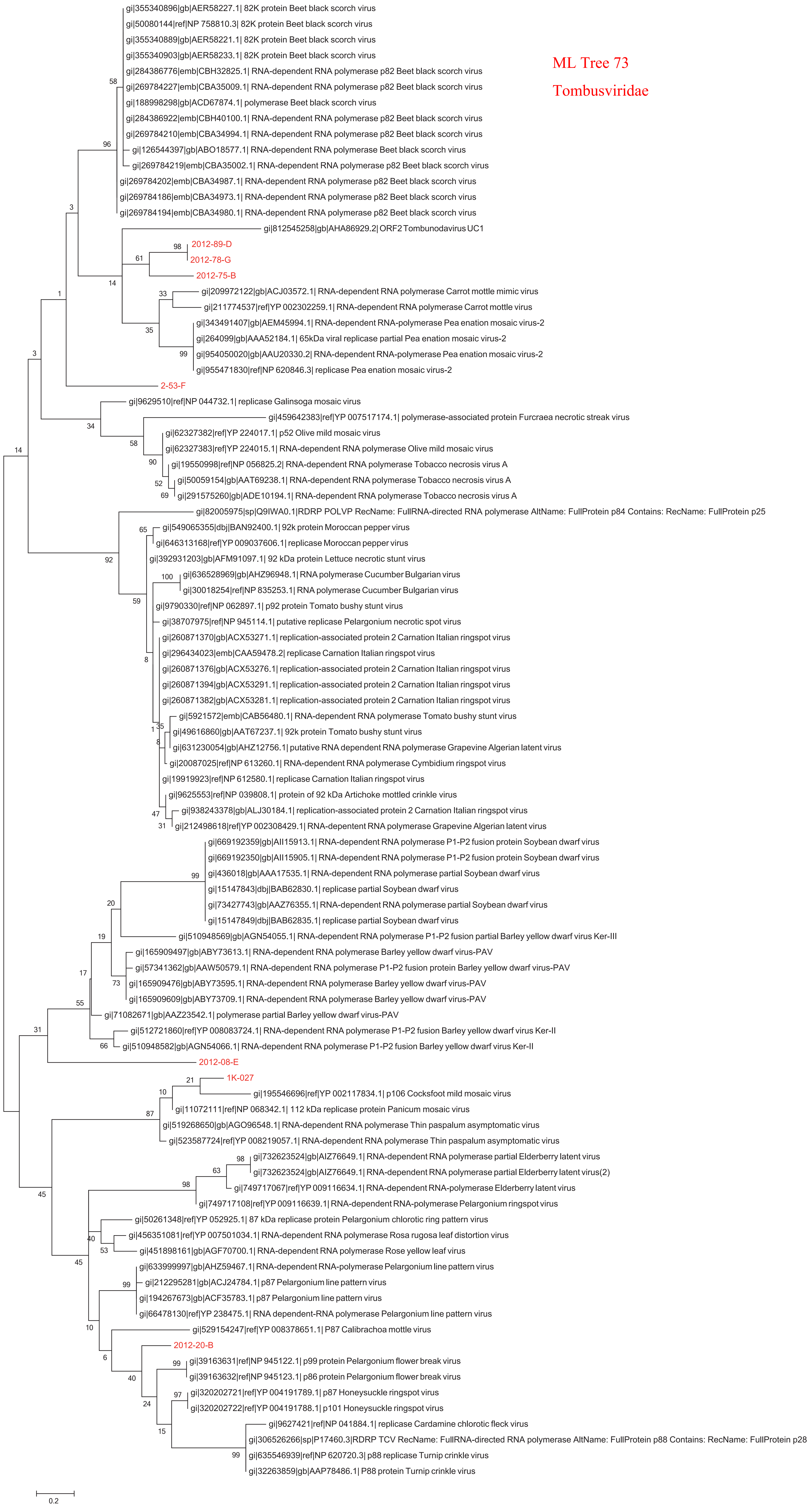
ML Tree 73
Tombusviridae
gi|355340896|gb|AER58227.1| 82K protein Beet black scorch virus
gi|50080144|ref|NP 758810.3| 82K protein Beet black scorch virus
gi|355340889|gb|AER58221.1| 82K protein Beet black scorch virus
gi|355340903|gb|AER58233.1| 82K protein Beet black scorch virus
gi|284386776|emb|CBH32825.1| RNA-dependent RNA polymerase p82 Beet black scorch virus
gi|269784227|emb|CBA35009.1| RNA-dependent RNA polymerase p82 Beet black scorch virus
gi|188998298|gb|ACD67874.1| polymerase Beet black scorch virus
gi|284386922|emb|CBH40100.1| RNA-dependent RNA polymerase p82 Beet black scorch virus
gi|269784210|emb|CBA34994.1| RNA-dependent RNA polymerase p82 Beet black scorch virus
gi|126544397|gb|ABO18577.1| RNA-dependent RNA polymerase Beet black scorch virus
gi|269784219|emb|CBA35002.1| RNA-dependent RNA polymerase p82 Beet black scorch virus
gi|269784202|emb|CBA34987.1| RNA-dependent RNA polymerase p82 Beet black scorch virus
gi|269784186|emb|CBA34973.1| RNA-dependent RNA polymerase p82 Beet black scorch virus
gi|269784194|emb|CBA34980.1| RNA-dependent RNA polymerase p82 Beet black scorch virus
gi|812545258|gb|AHA86929.2| ORF2 Tombunodavirus UC1
2012-89-D
2012-78-G
2012-75-B
gi|209972122|gb|ACJ03572.1| RNA-dependent RNA polymerase Carrot mottle mimic virus
gi|211774537|ref|YP 002302259.1| RNA-dependent RNA polymerase Carrot mottle virus
gi|343491407|gb|AEM45994.1| RNA-dependent RNA-polymerase Pea enation mosaic virus-2
gi|264099|gb|AAA52184.1| 65kDa viral replicase partial Pea enation mosaic virus-2
gi|954050020|gb|AAU20330.2| RNA-dependent RNA-polymerase Pea enation mosaic virus-2
gi|955471830|ref|NP 620846.3| replicase Pea enation mosaic virus-2
2-53-F
gi|9629510|ref|NP 044732.1| replicase Galinsoga mosaic virus
gi|459642383|ref|YP 007517174.1| polymerase-associated protein Furcraea necrotic streak virus
gi|62327382|ref|YP 224017.1| p52 Olive mild mosaic virus
gi|62327383|ref|YP 224015.1| RNA-dependent RNA polymerase Olive mild mosaic virus
gi|19550998|ref|NP 056825.2| RNA-dependent RNA polymerase Tobacco necrosis virus A
gi|50059154|gb|AAT69238.1| RNA-dependent RNA polymerase Tobacco necrosis virus A
gi|291575260|gb|ADE10194.1| RNA-dependent RNA polymerase Tobacco necrosis virus A
gi|82005975|sp|Q9IWA0.1|RDRP POLVP RecName: FullRNA-directed RNA polymerase AltName: FullProtein p84 Contains: RecName: FullProtein p25
gi|549065355|dbj|BAN92400.1| 92k protein Moroccan pepper virus
gi|646313168|ref|YP 009037606.1| replicase Moroccan pepper virus
gi|392931203|gb|AFM91097.1| 92 kDa protein Lettuce necrotic stunt virus
gi|636528969|gb|AHZ96948.1| RNA polymerase Cucumber Bulgarian virus
gi|30018254|ref|NP 835253.1| RNA polymerase Cucumber Bulgarian virus
gi|9790330|ref|NP 062897.1| p92 protein Tomato bushy stunt virus
gi|38707975|ref|NP 945114.1| putative replicase Pelargonium necrotic spot virus
gi|260871370|gb|ACX53271.1| replication-associated protein 2 Carnation Italian ringspot virus
gi|296434023|emb|CAA59478.2| replicase Carnation Italian ringspot virus
gi|260871376|gb|ACX53276.1| replication-associated protein 2 Carnation Italian ringspot virus
gi|260871394|gb|ACX53291.1| replication-associated protein 2 Carnation Italian ringspot virus
gi|260871382|gb|ACX53281.1| replication-associated protein 2 Carnation Italian ringspot virus
gi|5921572|emb|CAB56480.1| RNA-dependent RNA polymerase Tomato bushy stunt virus
gi|49616860|gb|AAT67237.1| 92k protein Tomato bushy stunt virus
gi|631230054|gb|AHZ12756.1| putative RNA dependent RNA polymerase Grapevine Algerian latent virus
gi|20087025|ref|NP 613260.1| RNA-dependent RNA polymerase Cymbidium ringspot virus
gi|19919923|ref|NP 612580.1| replicase Carnation Italian ringspot virus
gi|9625553|ref|NP 039808.1| protein of 92 kDa Artichoke mottled crinkle virus
gi|938243378|gb|ALJ30184.1| replication-associated protein 2 Carnation Italian ringspot virus
gi|212498618|ref|YP 002308429.1| RNA-depentent RNA polymerase Grapevine Algerian latent virus
gi|669192359|gb|AII15913.1| RNA-dependent RNA polymerase P1-P2 fusion protein Soybean dwarf virus
gi|669192350|gb|AII15905.1| RNA-dependent RNA polymerase P1-P2 fusion protein Soybean dwarf virus
gi|436018|gb|AAA17535.1| RNA-dependent RNA polymerase partial Soybean dwarf virus
gi|15147843|dbj|BAB62830.1| replicase partial Soybean dwarf virus
gi|73427743|gb|AAZ76355.1| RNA-dependent RNA polymerase partial Soybean dwarf virus
gi|15147849|dbj|BAB62835.1| replicase partial Soybean dwarf virus
gi|510948569|gb|AGN54055.1| RNA-dependent RNA polymerase P1-P2 fusion partial Barley yellow dwarf virus Ker-III
gi|165909497|gb|ABY73613.1| RNA-dependent RNA polymerase Barley yellow dwarf virus-PAV
gi|57341362|gb|AAW50579.1| RNA-dependent RNA polymerase P1-P2 fusion protein Barley yellow dwarf virus-PAV
gi|165909476|gb|ABY73595.1| RNA-dependent RNA polymerase Barley yellow dwarf virus-PAV
gi|165909609|gb|ABY73709.1| RNA-dependent RNA polymerase Barley yellow dwarf virus-PAV
gi|71082671|gb|AAZ23542.1| polymerase partial Barley yellow dwarf virus-PAV
gi|512721860|ref|YP 008083724.1| RNA-dependent RNA polymerase P1-P2 fusion Barley yellow dwarf virus Ker-II
gi|510948582|gb|AGN54066.1| RNA-dependent RNA polymerase P1-P2 fusion Barley yellow dwarf virus Ker-II
2012-08-E
1K-027
gi|195546696|ref|YP 002117834.1| p106 Cocksfoot mild mosaic virus
gi|11072111|ref|NP 068342.1| 112 kDa replicase protein Panicum mosaic virus
gi|519268650|gb|AGO96548.1| RNA-dependent RNA polymerase Thin paspalum asymptomatic virus
gi|523587724|ref|YP 008219057.1| RNA-dependent RNA polymerase Thin paspalum asymptomatic virus
gi|732623524|gb|AIZ76649.1| RNA-dependent RNA polymerase partial Elderberry latent virus
gi|732623524|gb|AIZ76649.1| RNA-dependent RNA polymerase partial Elderberry latent virus(2)
gi|749717067|ref|YP 009116634.1| RNA-dependent RNA-polymerase Elderberry latent virus
gi|749717108|ref|YP 009116639.1| RNA-dependent RNA-polymerase Pelargonium ringspot virus
gi|50261348|ref|YP 052925.1| 87 kDa replicase protein Pelargonium chlorotic ring pattern virus
gi|456351081|ref|YP 007501034.1| RNA-dependent RNA polymerase Rosa rugosa leaf distortion virus
gi|451898161|gb|AGF70700.1| RNA-dependent RNA polymerase Rose yellow leaf virus
gi|633999997|gb|AHZ59467.1| RNA-dependent RNA-polymerase Pelargonium line pattern virus
gi|212295281|gb|ACJ24784.1| p87 Pelargonium line pattern virus
gi|194267673|gb|ACF35783.1| p87 Pelargonium line pattern virus
gi|66478130|ref|YP 238475.1| RNA dependent-RNA polymerase Pelargonium line pattern virus
gi|529154247|ref|YP 008378651.1| P87 Calibrachoa mottle virus
2012-20-B
gi|39163631|ref|NP 945122.1| p99 protein Pelargonium flower break virus
gi|39163632|ref|NP 945123.1| p86 protein Pelargonium flower break virus
gi|320202721|ref|YP 004191789.1| p87 Honeysuckle ringspot virus
gi|320202722|ref|YP 004191788.1| p101 Honeysuckle ringspot virus
gi|9627421|ref|NP 041884.1| replicase Cardamine chlorotic fleck virus
gi|306526266|sp|P17460.3|RDRP TCV RecName: FullRNA-directed RNA polymerase AltName: FullProtein p88 Contains: RecName: FullProtein p28
gi|635546939|ref|NP 620720.3| p88 replicase Turnip crinkle virus
gi|32263859|gb|AAP78486.1| P88 protein Turnip crinkle virus
0.2

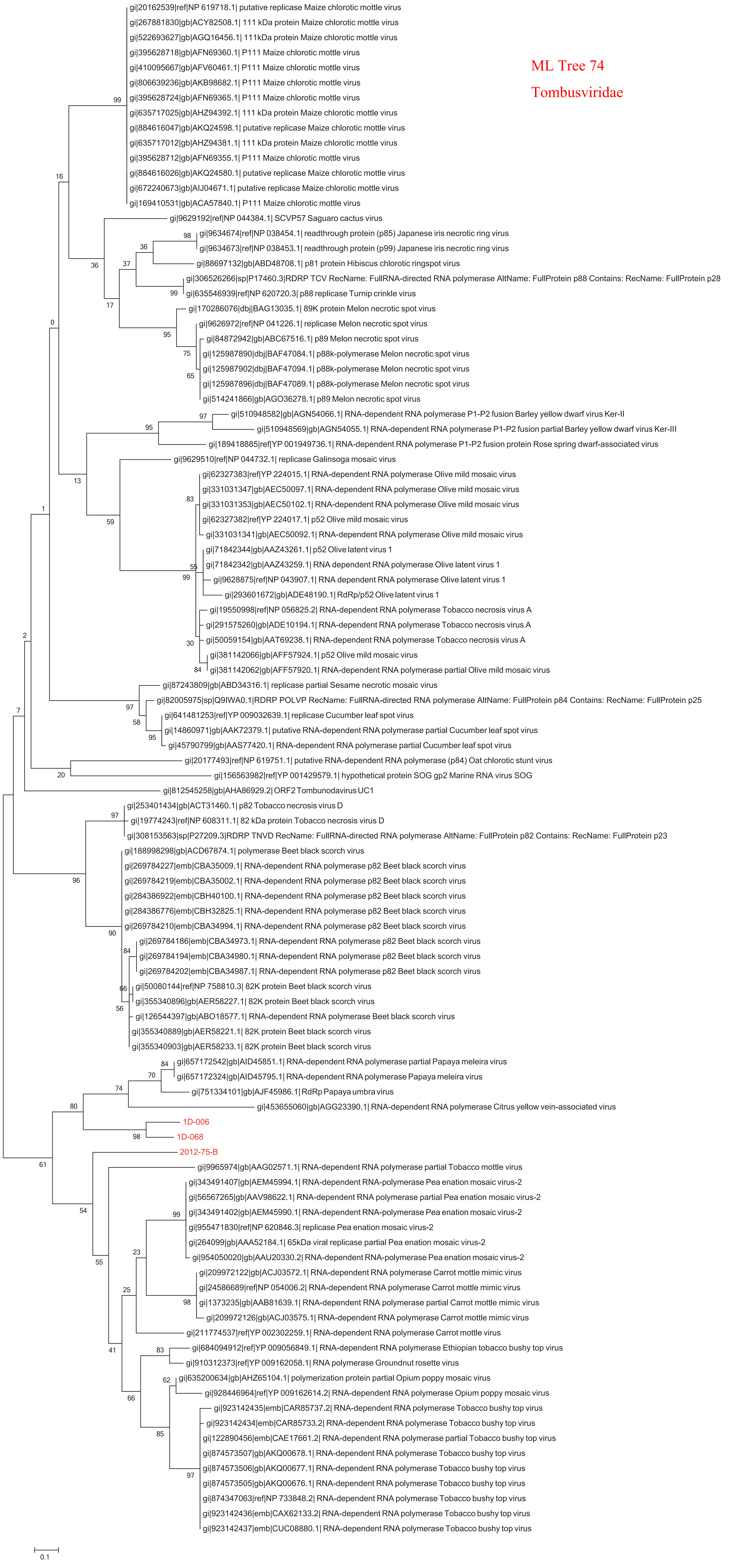
ML Tree 74
Tombusviridae
gi|20162539|ref|NP 619718.1| putative replicase Maize chlorotic mottle virus
gi|267881830|gb|ACY82508.1| 111 kDa protein Maize chlorotic mottle virus
gi|522693627|gb|AGQ16456.1| 111kDa protein Maize chlorotic mottle virus
gi|395628718|gb|AFN69360.1| P111 Maize chlorotic mottle virus
gi|410095667|gb|AFV60461.1| P111 Maize chlorotic mottle virus
gi|806639236|gb|AKB98682.1| P111 Maize chlorotic mottle virus
gi|395628724|gb|AFN69365.1| P111 Maize chlorotic mottle virus
gi|635717025|gb|AHZ94392.1| 111 kDa protein Maize chlorotic mottle virus
gi|884616047|gb|AKQ24598.1| putative replicase Maize chlorotic mottle virus
gi|635717012|gb|AHZ94381.1| 111 kDa protein Maize chlorotic mottle virus
gi|395628712|gb|AFN69355.1| P111 Maize chlorotic mottle virus
gi|884616026|gb|AKQ24580.1| putative replicase Maize chlorotic mottle virus
gi|672240673|gb|AIJ04671.1| putative replicase Maize chlorotic mottle virus
gi|169410531|gb|ACA57840.1| P111 Maize chlorotic mottle virus
gi|9629192|ref|NP 044384.1| SCVP57 Saguaro cactus virus
gi|9634674|ref|NP 038454.1| readthrough protein (p85) Japanese iris necrotic ring virus
gi|9634673|ref|NP 038453.1| readthrough protein (p99) Japanese iris necrotic ring virus
gi|88697132|gb|ABD48708.1| p81 protein Hibiscus chlorotic ringspot virus
gi|306526266|sp|P17460.3|RDRP TCV RecName: FullRNA-directed RNA polymerase AltName: FullProtein p88 Contains: RecName: FullProtein p28
gi|635546939|ref|NP 620720.3| p88 replicase Turnip crinkle virus
gi|170286076|dbj|BAG13035.1| 89K protein Melon necrotic spot virus
gi|9626972|ref|NP 041226.1| replicase Melon necrotic spot virus
gi|84872942|gb|ABC67516.1| p89 Melon necrotic spot virus
gi|125987890|dbj|BAF47084.1| p88k-polymerase Melon necrotic spot virus
gi|125987902|dbj|BAF47094.1| p88k-polymerase Melon necrotic spot virus
gi|125987896|dbj|BAF47089.1| p88k-polymerase Melon necrotic spot virus
gi|514241866|gb|AGO36278.1| p89 Melon necrotic spot virus
gi|510948582|gb|AGN54066.1| RNA-dependent RNA polymerase P1-P2 fusion Barley yellow dwarf virus Ker-II
gi|510948569|gb|AGN54055.1| RNA-dependent RNA polymerase P1-P2 fusion partial Barley yellow dwarf virus Ker-III
gi|189418885|ref|YP 001949736.1| RNA-dependent RNA polymerase P1-P2 fusion protein Rose spring dwarf-associated virus
gi|9629510|ref|NP 044732.1| replicase Galinsoga mosaic virus
gi|62327383|ref|YP 224015.1| RNA-dependent RNA polymerase Olive mild mosaic virus
gi|331031347|gb|AEC50097.1| RNA-dependent RNA polymerase Olive mild mosaic virus
gi|331031353|gb|AEC50102.1| RNA-dependent RNA polymerase Olive mild mosaic virus
gi|62327382|ref|YP 224017.1| p52 Olive mild mosaic virus
gi|331031341|gb|AEC50092.1| RNA-dependent RNA polymerase Olive mild mosaic virus
gi|71842344|gb|AAZ43261.1| p52 Olive latent virus 1
gi|71842342|gb|AAZ43259.1| RNA dependent RNA polymerase Olive latent virus 1
gi|9628875|ref|NP 043907.1| RNA dependent RNA polymerase Olive latent virus 1
gi|293601672|gb|ADE48190.1| RdRp/p52 Olive latent virus 1
gi|19550998|ref|NP 056825.2| RNA-dependent RNA polymerase Tobacco necrosis virus A
gi|291575260|gb|ADE10194.1| RNA-dependent RNA polymerase Tobacco necrosis virus A
gi|50059154|gb|AAT69238.1| RNA-dependent RNA polymerase Tobacco necrosis virus A
gi|381142066|gb|AFF57924.1| p52 Olive mild mosaic virus
gi|381142062|gb|AFF57920.1| RNA-dependent RNA polymerase partial Olive mild mosaic virus
gi|87243809|gb|ABD34316.1| replicase partial Sesame necrotic mosaic virus
gi|82005975|sp|Q9IWA0.1|RDRP POLVP RecName: FullRNA-directed RNA polymerase AltName: FullProtein p84 Contains: RecName: FullProtein p25
gi|641481253|ref|YP 009032639.1| replicase Cucumber leaf spot virus
gi|14860971|gb|AAK72379.1| putative RNA-dependent RNA polymerase partial Cucumber leaf spot virus
gi|45790799|gb|AAS77420.1| RNA-dependent RNA polymerase partial Cucumber leaf spot virus
gi|20177493|ref|NP 619751.1| putative RNA-dependent RNA polymerase (p84) Oat chlorotic stunt virus
gi|156563982|ref|YP 001429579.1| hypothetical protein SOG gp2 Marine RNA virus SOG
gi|812545258|gb|AHA86929.2| ORF2 Tombunodavirus UC1
gi|253401434|gb|ACT31460.1| p82 Tobacco necrosis virus D
gi|19774243|ref|NP 608311.1| 82 kDa protein Tobacco necrosis virus D
gi|308153563|sp|P27209.3|RDRP TNVD RecName: FullRNA-directed RNA polymerase AltName: FullProtein p82 Contains: RecName: FullProtein p23
gi|188998298|gb|ACD67874.1| polymerase Beet black scorch virus
gi|269784227|emb|CBA35009.1| RNA-dependent RNA polymerase p82 Beet black scorch virus
gi|269784219|emb|CBA35002.1| RNA-dependent RNA polymerase p82 Beet black scorch virus
gi|284386922|emb|CBH40100.1| RNA-dependent RNA polymerase p82 Beet black scorch virus
gi|284386776|emb|CBH32825.1| RNA-dependent RNA polymerase p82 Beet black scorch virus
gi|269784210|emb|CBA34994.1| RNA-dependent RNA polymerase p82 Beet black scorch virus
gi|269784186|emb|CBA34973.1| RNA-dependent RNA polymerase p82 Beet black scorch virus
gi|269784194|emb|CBA34980.1| RNA-dependent RNA polymerase p82 Beet black scorch virus
gi|269784202|emb|CBA34987.1| RNA-dependent RNA polymerase p82 Beet black scorch virus
gi|50080144|ref|NP 758810.3| 82K protein Beet black scorch virus
gi|355340896|gb|AER58227.1| 82K protein Beet black scorch virus
gi|126544397|gb|ABO18577.1| RNA-dependent RNA polymerase Beet black scorch virus
gi|355340889|gb|AER58221.1| 82K protein Beet black scorch virus
gi|355340903|gb|AER58233.1| 82K protein Beet black scorch virus
gi|657172542|gb|AID45851.1| RNA-dependent RNA polymerase partial Papaya meleira virus
gi|657172324|gb|AID45795.1| RNA-dependent RNA polymerase Papaya meleira virus
gi|751334101|gb|AJF45986.1| RdRp Papaya umbra virus
gi|453655060|gb|AGG23390.1| RNA-dependent RNA polymerase Citrus yellow vein-associated virus
1D-006
1D-068
2012-75-B
gi|9965974|gb|AAG02571.1| RNA-dependent RNA polymerase partial Tobacco mottle virus
gi|343491407|gb|AEM45994.1| RNA-dependent RNA-polymerase Pea enation mosaic virus-2
gi|56567265|gb|AAV98622.1| RNA-dependent RNA polymerase partial Pea enation mosaic virus-2
gi|343491402|gb|AEM45990.1| RNA-dependent RNA-polymerase Pea enation mosaic virus-2
gi|955471830|ref|NP 620846.3| replicase Pea enation mosaic virus-2
gi|264099|gb|AAA52184.1| 65kDa viral replicase partial Pea enation mosaic virus-2
gi|954050020|gb|AAU20330.2| RNA-dependent RNA-polymerase Pea enation mosaic virus-2
gi|209972122|gb|ACJ03572.1| RNA-dependent RNA polymerase Carrot mottle mimic virus
gi|24586689|ref|NP 054006.2| RNA-dependent RNA polymerase Carrot mottle mimic virus
gi|1373235|gb|AAB81639.1| RNA-dependent RNA polymerase partial Carrot mottle mimic virus
gi|209972126|gb|ACJ03575.1| RNA-dependent RNA polymerase Carrot mottle mimic virus
gi|211774537|ref|YP 002302259.1| RNA-dependent RNA polymerase Carrot mottle virus
gi|684094912|ref|YP 009056849.1| RNA-dependent RNA polymerase Ethiopian tobacco bushy top virus
gi|910312373|ref|YP 009162058.1| RNA polymerase Groundnut rosette virus
gi|635200634|gb|AHZ65104.1| polymerization protein partial Opium poppy mosaic virus
gi|928446964|ref|YP 009162614.2| RNA-dependent RNA polymerase Opium poppy mosaic virus
gi|923142435|emb|CAR85737.2| RNA-dependent RNA polymerase Tobacco bushy top virus
gi|923142434|emb|CAR85733.2| RNA-dependent RNA polymerase Tobacco bushy top virus
gi|122890456|emb|CAE17661.2| RNA-dependent RNA polymerase partial Tobacco bushy top virus
gi|874573507|gb|AKQ00678.1| RNA-dependent RNA polymerase Tobacco bushy top virus
gi|874573506|gb|AKQ00677.1| RNA-dependent RNA polymerase Tobacco bushy top virus
gi|874573505|gb|AKQ00676.1| RNA-dependent RNA polymerase Tobacco bushy top virus
gi|874347063|ref|NP 733848.2| RNA-dependent RNA polymerase Tobacco bushy top virus
gi|923142436|emb|CAX62133.2| RNA-dependent RNA polymerase Tobacco bushy top virus
gi|923142437|emb|CUC08880.1| RNA-dependent RNA polymerase Tobacco bushy top virus
0.1

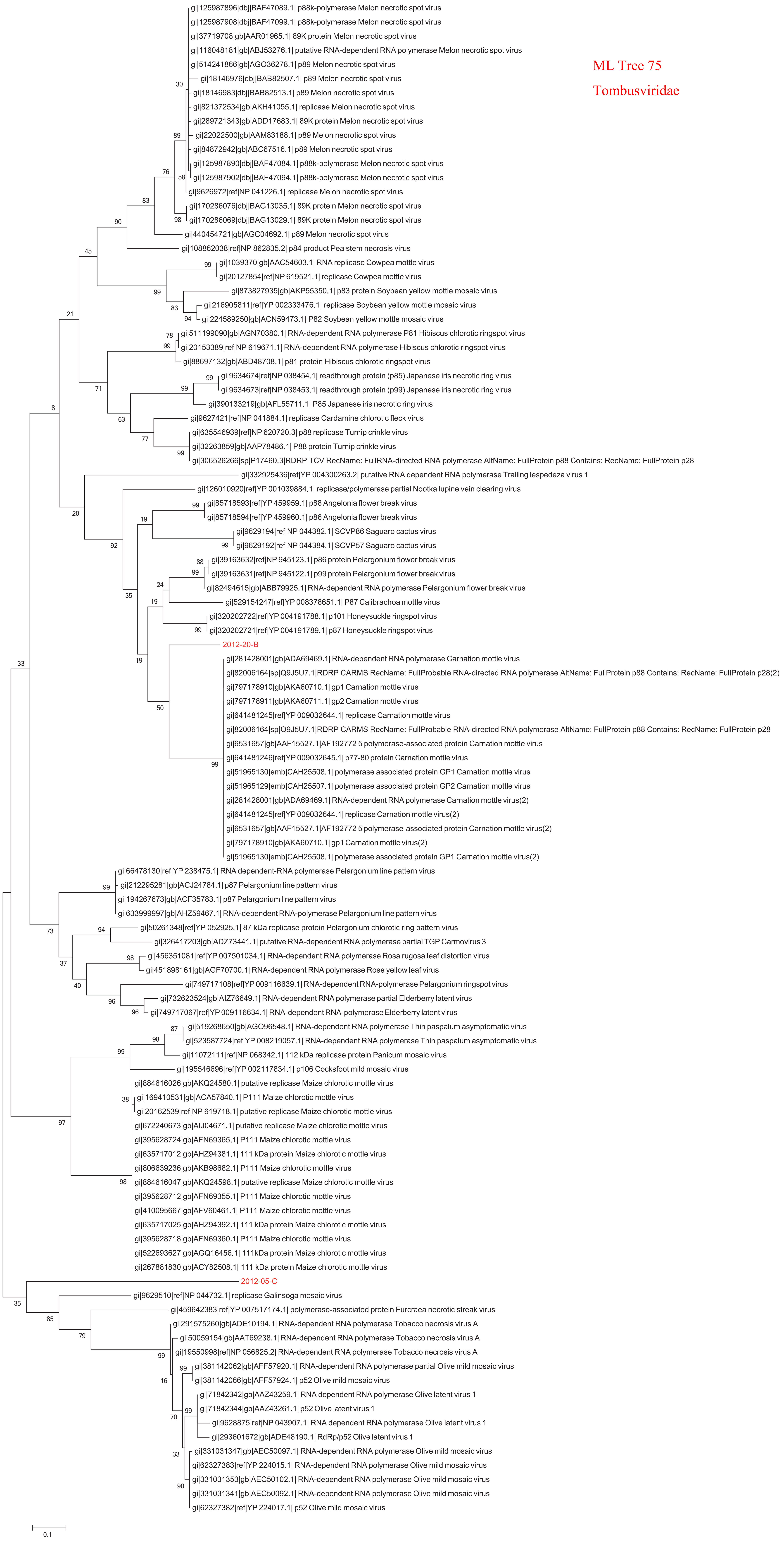
ML Tree 75
Tombusviridae
gi|125987896|dbj|BAF47089.1| p88k-polymerase Melon necrotic spot virus
gi|125987908|dbj|BAF47099.1| p88k-polymerase Melon necrotic spot virus
gi|37719708|gb|AAR01965.1| 89K protein Melon necrotic spot virus
gi|116048181|gb|ABJ53276.1| putative RNA-dependent RNA polymerase Melon necrotic spot virus
gi|514241866|gb|AGO36278.1| p89 Melon necrotic spot virus
gi|18146976|dbj|BAB82507.1| p89 Melon necrotic spot virus
gi|18146983|dbj|BAB82513.1| p89 Melon necrotic spot virus
gi|821372534|gb|AKH41055.1| replicase Melon necrotic spot virus
gi|289721343|gb|ADD17683.1| 89K protein Melon necrotic spot virus
gi|22022500|gb|AAM83188.1| p89 Melon necrotic spot virus
gi|84872942|gb|ABC67516.1| p89 Melon necrotic spot virus
gi|125987890|dbj|BAF47084.1| p88k-polymerase Melon necrotic spot virus
gi|125987902|dbj|BAF47094.1| p88k-polymerase Melon necrotic spot virus
gi|9626972|ref|NP 041226.1| replicase Melon necrotic spot virus
gi|170286076|dbj|BAG13035.1| 89K protein Melon necrotic spot virus
gi|170286069|dbj|BAG13029.1| 89K protein Melon necrotic spot virus
gi|440454721|gb|AGC04692.1| p89 Melon necrotic spot virus
gi|108862038|ref|NP 862835.2| p84 product Pea stem necrosis virus
gi|1039370|gb|AAC54603.1| RNA replicase Cowpea mottle virus
gi|20127854|ref|NP 619521.1| replicase Cowpea mottle virus
gi|873827935|gb|AKP55350.1| p83 protein Soybean yellow mottle mosaic virus
gi|216905811|ref|YP 002333476.1| replicase Soybean yellow mottle mosaic virus
gi|224589250|gb|ACN59473.1| P82 Soybean yellow mottle mosaic virus
gi|511199090|gb|AGN70380.1| RNA-dependent RNA polymerase P81 Hibiscus chlorotic ringspot virus
gi|20153389|ref|NP 619671.1| RNA-dependent RNA polymerase Hibiscus chlorotic ringspot virus
gi|88697132|gb|ABD48708.1| p81 protein Hibiscus chlorotic ringspot virus
gi|9634674|ref|NP 038454.1| readthrough protein (p85) Japanese iris necrotic ring virus
gi|9634673|ref|NP 038453.1| readthrough protein (p99) Japanese iris necrotic ring virus
gi|390133219|gb|AFL55711.1| P85 Japanese iris necrotic ring virus
gi|9627421|ref|NP 041884.1| replicase Cardamine chlorotic fleck virus
gi|635546939|ref|NP 620720.3| p88 replicase Turnip crinkle virus
gi|32263859|gb|AAP78486.1| P88 protein Turnip crinkle virus
gi|306526266|sp|P17460.3|RDRP TCV RecName: FullRNA-directed RNA polymerase AltName: FullProtein p88 Contains: RecName: FullProtein p28
gi|332925436|ref|YP 004300263.2| putative RNA dependent RNA polymerase Trailing lespedeza virus 1
gi|126010920|ref|YP 001039884.1| replicase/polymerase partial Nootka lupine vein clearing virus
gi|85718593|ref|YP 459959.1| p88 Angelonia flower break virus
gi|85718594|ref|YP 459960.1| p86 Angelonia flower break virus
gi|9629194|ref|NP 044382.1| SCVP86 Saguaro cactus virus
gi|9629192|ref|NP 044384.1| SCVP57 Saguaro cactus virus
gi|39163632|ref|NP 945123.1| p86 protein Pelargonium flower break virus
gi|39163631|ref|NP 945122.1| p99 protein Pelargonium flower break virus
gi|82494615|gb|ABB79925.1| RNA-dependent RNA polymerase Pelargonium flower break virus
gi|529154247|ref|YP 008378651.1| P87 Calibrachoa mottle virus
gi|320202722|ref|YP 004191788.1| p101 Honeysuckle ringspot virus
gi|320202721|ref|YP 004191789.1| p87 Honeysuckle ringspot virus
2012-20-B
gi|281428001|gb|ADA69469.1| RNA-dependent RNA polymerase Carnation mottle virus
gi|82006164|sp|Q9J5U7.1|RDRP CARMS RecName: FullProbable RNA-directed RNA polymerase AltName: FullProtein p88 Contains: RecName: FullProtein p28(2)
gi|797178910|gb|AKA60710.1| gp1 Carnation mottle virus
gi|797178911|gb|AKA60711.1| gp2 Carnation mottle virus
gi|641481245|ref|YP 009032644.1| replicase Carnation mottle virus
gi|82006164|sp|Q9J5U7.1|RDRP CARMS RecName: FullProbable RNA-directed RNA polymerase AltName: FullProtein p88 Contains: RecName: FullProtein p28
gi|6531657|gb|AAF15527.1|AF192772 5 polymerase-associated protein Carnation mottle virus
gi|641481246|ref|YP 009032645.1| p77-80 protein Carnation mottle virus
gi|51965130|emb|CAH25508.1| polymerase associated protein GP1 Carnation mottle virus
gi|51965129|emb|CAH25507.1| polymerase associated protein GP2 Carnation mottle virus
gi|281428001|gb|ADA69469.1| RNA-dependent RNA polymerase Carnation mottle virus(2)
gi|641481245|ref|YP 009032644.1| replicase Carnation mottle virus(2)
gi|6531657|gb|AAF15527.1|AF192772 5 polymerase-associated protein Carnation mottle virus(2)
gi|797178910|gb|AKA60710.1| gp1 Carnation mottle virus(2)
gi|51965130|emb|CAH25508.1| polymerase associated protein GP1 Carnation mottle virus(2)
gi|66478130|ref|YP 238475.1| RNA dependent-RNA polymerase Pelargonium line pattern virus
gi|212295281|gb|ACJ24784.1| p87 Pelargonium line pattern virus
gi|194267673|gb|ACF35783.1| p87 Pelargonium line pattern virus
gi|633999997|gb|AHZ59467.1| RNA-dependent RNA-polymerase Pelargonium line pattern virus
gi|50261348|ref|YP 052925.1| 87 kDa replicase protein Pelargonium chlorotic ring pattern virus
gi|326417203|gb|ADZ73441.1| putative RNA-dependent RNA polymerase partial TGP Carmovirus 3
gi|456351081|ref|YP 007501034.1| RNA-dependent RNA polymerase Rosa rugosa leaf distortion virus
gi|451898161|gb|AGF70700.1| RNA-dependent RNA polymerase Rose yellow leaf virus
gi|749717108|ref|YP 009116639.1| RNA-dependent RNA-polymerase Pelargonium ringspot virus
gi|732623524|gb|AIZ76649.1| RNA-dependent RNA polymerase partial Elderberry latent virus
gi|749717067|ref|YP 009116634.1| RNA-dependent RNA-polymerase Elderberry latent virus
gi|519268650|gb|AGO96548.1| RNA-dependent RNA polymerase Thin paspalum asymptomatic virus
gi|523587724|ref|YP 008219057.1| RNA-dependent RNA polymerase Thin paspalum asymptomatic virus
gi|11072111|ref|NP 068342.1| 112 kDa replicase protein Panicum mosaic virus
gi|195546696|ref|YP 002117834.1| p106 Cocksfoot mild mosaic virus
gi|884616026|gb|AKQ24580.1| putative replicase Maize chlorotic mottle virus
gi|169410531|gb|ACA57840.1| P111 Maize chlorotic mottle virus
gi|20162539|ref|NP 619718.1| putative replicase Maize chlorotic mottle virus
gi|672240673|gb|AIJ04671.1| putative replicase Maize chlorotic mottle virus
gi|395628724|gb|AFN69365.1| P111 Maize chlorotic mottle virus
gi|635717012|gb|AHZ94381.1| 111 kDa protein Maize chlorotic mottle virus
gi|806639236|gb|AKB98682.1| P111 Maize chlorotic mottle virus
gi|884616047|gb|AKQ24598.1| putative replicase Maize chlorotic mottle virus
gi|395628712|gb|AFN69355.1| P111 Maize chlorotic mottle virus
gi|410095667|gb|AFV60461.1| P111 Maize chlorotic mottle virus
gi|635717025|gb|AHZ94392.1| 111 kDa protein Maize chlorotic mottle virus
gi|395628718|gb|AFN69360.1| P111 Maize chlorotic mottle virus
gi|522693627|gb|AGQ16456.1| 111kDa protein Maize chlorotic mottle virus
gi|267881830|gb|ACY82508.1| 111 kDa protein Maize chlorotic mottle virus
2012-05-C
gi|9629510|ref|NP 044732.1| replicase Galinsoga mosaic virus
gi|459642383|ref|YP 007517174.1| polymerase-associated protein Furcraea necrotic streak virus
gi|291575260|gb|ADE10194.1| RNA-dependent RNA polymerase Tobacco necrosis virus A
gi|50059154|gb|AAT69238.1| RNA-dependent RNA polymerase Tobacco necrosis virus A
gi|19550998|ref|NP 056825.2| RNA-dependent RNA polymerase Tobacco necrosis virus A
gi|381142062|gb|AFF57920.1| RNA-dependent RNA polymerase partial Olive mild mosaic virus
gi|381142066|gb|AFF57924.1| p52 Olive mild mosaic virus
gi|71842342|gb|AAZ43259.1| RNA dependent RNA polymerase Olive latent virus 1
gi|71842344|gb|AAZ43261.1| p52 Olive latent virus 1
gi|9628875|ref|NP 043907.1| RNA dependent RNA polymerase Olive latent virus 1
gi|293601672|gb|ADE48190.1| RdRp/p52 Olive latent virus 1
gi|331031347|gb|AEC50097.1| RNA-dependent RNA polymerase Olive mild mosaic virus
gi|62327383|ref|YP 224015.1| RNA-dependent RNA polymerase Olive mild mosaic virus
gi|331031353|gb|AEC50102.1| RNA-dependent RNA polymerase Olive mild mosaic virus
gi|331031341|gb|AEC50092.1| RNA-dependent RNA polymerase Olive mild mosaic virus
gi|62327382|ref|YP 224017.1| p52 Olive mild mosaic virus
0.1

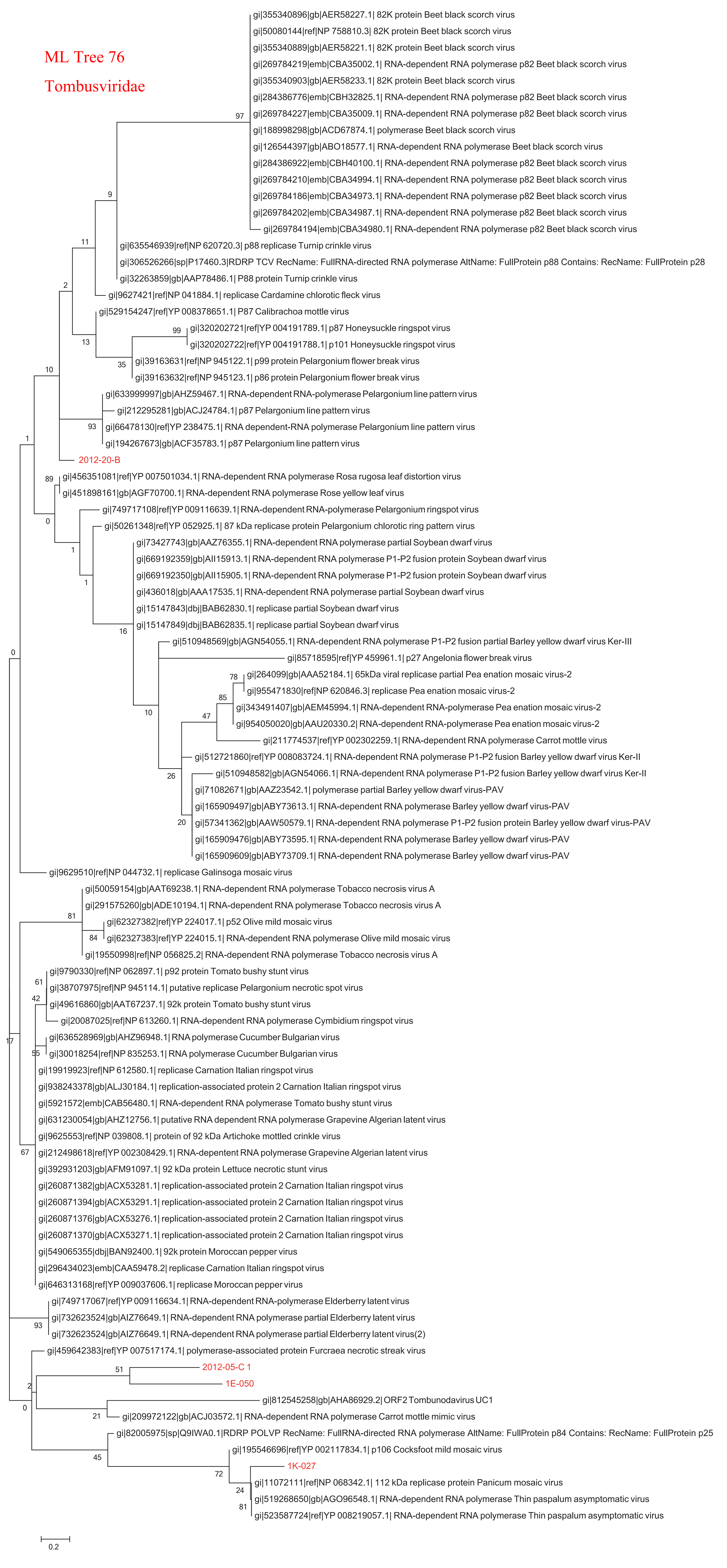
ML Tree 76
Tombusviridae
gi|355340896|gb|AER58227.1| 82K protein Beet black scorch virus
gi|50080144|ref|NP 758810.3| 82K protein Beet black scorch virus
gi|355340889|gb|AER58221.1| 82K protein Beet black scorch virus
gi|269784219|emb|CBA35002.1| RNA-dependent RNA polymerase p82 Beet black scorch virus
gi|355340903|gb|AER58233.1| 82K protein Beet black scorch virus
gi|284386776|emb|CBH32825.1| RNA-dependent RNA polymerase p82 Beet black scorch virus
gi|269784227|emb|CBA35009.1| RNA-dependent RNA polymerase p82 Beet black scorch virus
gi|188998298|gb|ACD67874.1| polymerase Beet black scorch virus
gi|126544397|gb|ABO18577.1| RNA-dependent RNA polymerase Beet black scorch virus
gi|284386922|emb|CBH40100.1| RNA-dependent RNA polymerase p82 Beet black scorch virus
gi|269784210|emb|CBA34994.1| RNA-dependent RNA polymerase p82 Beet black scorch virus
gi|269784186|emb|CBA34973.1| RNA-dependent RNA polymerase p82 Beet black scorch virus
gi|269784202|emb|CBA34987.1| RNA-dependent RNA polymerase p82 Beet black scorch virus
gi|269784194|emb|CBA34980.1| RNA-dependent RNA polymerase p82 Beet black scorch virus
gi|635546939|ref|NP 620720.3| p88 replicase Turnip crinkle virus
gi|306526266|sp|P17460.3|RDRP TCV RecName: FullRNA-directed RNA polymerase AltName: FullProtein p88 Contains: RecName: FullProtein p28
gi|32263859|gb|AAP78486.1| P88 protein Turnip crinkle virus
gi|9627421|ref|NP 041884.1| replicase Cardamine chlorotic fleck virus
gi|529154247|ref|YP 008378651.1| P87 Calibrachoa mottle virus
gi|320202721|ref|YP 004191789.1| p87 Honeysuckle ringspot virus
gi|320202722|ref|YP 004191788.1| p101 Honeysuckle ringspot virus
gi|39163631|ref|NP 945122.1| p99 protein Pelargonium flower break virus
gi|39163632|ref|NP 945123.1| p86 protein Pelargonium flower break virus
gi|633999997|gb|AHZ59467.1| RNA-dependent RNA-polymerase Pelargonium line pattern virus
gi|212295281|gb|ACJ24784.1| p87 Pelargonium line pattern virus
gi|66478130|ref|YP 238475.1| RNA dependent-RNA polymerase Pelargonium line pattern virus
gi|194267673|gb|ACF35783.1| p87 Pelargonium line pattern virus
2012-20-B
gi|456351081|ref|YP 007501034.1| RNA-dependent RNA polymerase Rosa rugosa leaf distortion virus
gi|451898161|gb|AGF70700.1| RNA-dependent RNA polymerase Rose yellow leaf virus
gi|749717108|ref|YP 009116639.1| RNA-dependent RNA-polymerase Pelargonium ringspot virus
gi|50261348|ref|YP 052925.1| 87 kDa replicase protein Pelargonium chlorotic ring pattern virus
gi|73427743|gb|AAZ76355.1| RNA-dependent RNA polymerase partial Soybean dwarf virus
gi|669192359|gb|AII15913.1| RNA-dependent RNA polymerase P1-P2 fusion protein Soybean dwarf virus
gi|669192350|gb|AII15905.1| RNA-dependent RNA polymerase P1-P2 fusion protein Soybean dwarf virus
gi|436018|gb|AAA17535.1| RNA-dependent RNA polymerase partial Soybean dwarf virus
gi|15147843|dbj|BAB62830.1| replicase partial Soybean dwarf virus
gi|15147849|dbj|BAB62835.1| replicase partial Soybean dwarf virus
gi|510948569|gb|AGN54055.1| RNA-dependent RNA polymerase P1-P2 fusion partial Barley yellow dwarf virus Ker-III
gi|85718595|ref|YP 459961.1| p27 Angelonia flower break virus
gi|264099|gb|AAA52184.1| 65kDa viral replicase partial Pea enation mosaic virus-2
gi|955471830|ref|NP 620846.3| replicase Pea enation mosaic virus-2
gi|343491407|gb|AEM45994.1| RNA-dependent RNA-polymerase Pea enation mosaic virus-2
gi|954050020|gb|AAU20330.2| RNA-dependent RNA-polymerase Pea enation mosaic virus-2
gi|211774537|ref|YP 002302259.1| RNA-dependent RNA polymerase Carrot mottle virus
gi|512721860|ref|YP 008083724.1| RNA-dependent RNA polymerase P1-P2 fusion Barley yellow dwarf virus Ker-II
gi|510948582|gb|AGN54066.1| RNA-dependent RNA polymerase P1-P2 fusion Barley yellow dwarf virus Ker-II
gi|71082671|gb|AAZ23542.1| polymerase partial Barley yellow dwarf virus-PAV
gi|165909497|gb|ABY73613.1| RNA-dependent RNA polymerase Barley yellow dwarf virus-PAV
gi|57341362|gb|AAW50579.1| RNA-dependent RNA polymerase P1-P2 fusion protein Barley yellow dwarf virus-PAV
gi|165909476|gb|ABY73595.1| RNA-dependent RNA polymerase Barley yellow dwarf virus-PAV
gi|165909609|gb|ABY73709.1| RNA-dependent RNA polymerase Barley yellow dwarf virus-PAV
gi|9629510|ref|NP 044732.1| replicase Galinsoga mosaic virus
gi|50059154|gb|AAT69238.1| RNA-dependent RNA polymerase Tobacco necrosis virus A
gi|291575260|gb|ADE10194.1| RNA-dependent RNA polymerase Tobacco necrosis virus A
gi|62327382|ref|YP 224017.1| p52 Olive mild mosaic virus
gi|62327383|ref|YP 224015.1| RNA-dependent RNA polymerase Olive mild mosaic virus
gi|19550998|ref|NP 056825.2| RNA-dependent RNA polymerase Tobacco necrosis virus A
gi|9790330|ref|NP 062897.1| p92 protein Tomato bushy stunt virus
gi|38707975|ref|NP 945114.1| putative replicase Pelargonium necrotic spot virus
gi|49616860|gb|AAT67237.1| 92k protein Tomato bushy stunt virus
gi|20087025|ref|NP 613260.1| RNA-dependent RNA polymerase Cymbidium ringspot virus
gi|636528969|gb|AHZ96948.1| RNA polymerase Cucumber Bulgarian virus
gi|30018254|ref|NP 835253.1| RNA polymerase Cucumber Bulgarian virus
gi|19919923|ref|NP 612580.1| replicase Carnation Italian ringspot virus
gi|938243378|gb|ALJ30184.1| replication-associated protein 2 Carnation Italian ringspot virus
gi|5921572|emb|CAB56480.1| RNA-dependent RNA polymerase Tomato bushy stunt virus
gi|631230054|gb|AHZ12756.1| putative RNA dependent RNA polymerase Grapevine Algerian latent virus
gi|9625553|ref|NP 039808.1| protein of 92 kDa Artichoke mottled crinkle virus
gi|212498618|ref|YP 002308429.1| RNA-depentent RNA polymerase Grapevine Algerian latent virus
gi|392931203|gb|AFM91097.1| 92 kDa protein Lettuce necrotic stunt virus
gi|260871382|gb|ACX53281.1| replication-associated protein 2 Carnation Italian ringspot virus
gi|260871394|gb|ACX53291.1| replication-associated protein 2 Carnation Italian ringspot virus
gi|260871376|gb|ACX53276.1| replication-associated protein 2 Carnation Italian ringspot virus
gi|260871370|gb|ACX53271.1| replication-associated protein 2 Carnation Italian ringspot virus
gi|549065355|dbj|BAN92400.1| 92k protein Moroccan pepper virus
gi|296434023|emb|CAA59478.2| replicase Carnation Italian ringspot virus
gi|646313168|ref|YP 009037606.1| replicase Moroccan pepper virus
gi|749717067|ref|YP 009116634.1| RNA-dependent RNA-polymerase Elderberry latent virus
gi|732623524|gb|AIZ76649.1| RNA-dependent RNA polymerase partial Elderberry latent virus
gi|732623524|gb|AIZ76649.1| RNA-dependent RNA polymerase partial Elderberry latent virus(2)
gi|459642383|ref|YP 007517174.1| polymerase-associated protein Furcraea necrotic streak virus
2012-05-C 1
1E-050
gi|812545258|gb|AHA86929.2| ORF2 Tombunodavirus UC1
gi|209972122|gb|ACJ03572.1| RNA-dependent RNA polymerase Carrot mottle mimic virus
gi|82005975|sp|Q9IWA0.1|RDRP POLVP RecName: FullRNA-directed RNA polymerase AltName: FullProtein p84 Contains: RecName: FullProtein p25
gi|195546696|ref|YP 002117834.1| p106 Cocksfoot mild mosaic virus
1K-027
gi|11072111|ref|NP 068342.1| 112 kDa replicase protein Panicum mosaic virus
gi|519268650|gb|AGO96548.1| RNA-dependent RNA polymerase Thin paspalum asymptomatic virus
gi|523587724|ref|YP 008219057.1| RNA-dependent RNA polymerase Thin paspalum asymptomatic virus
0.2

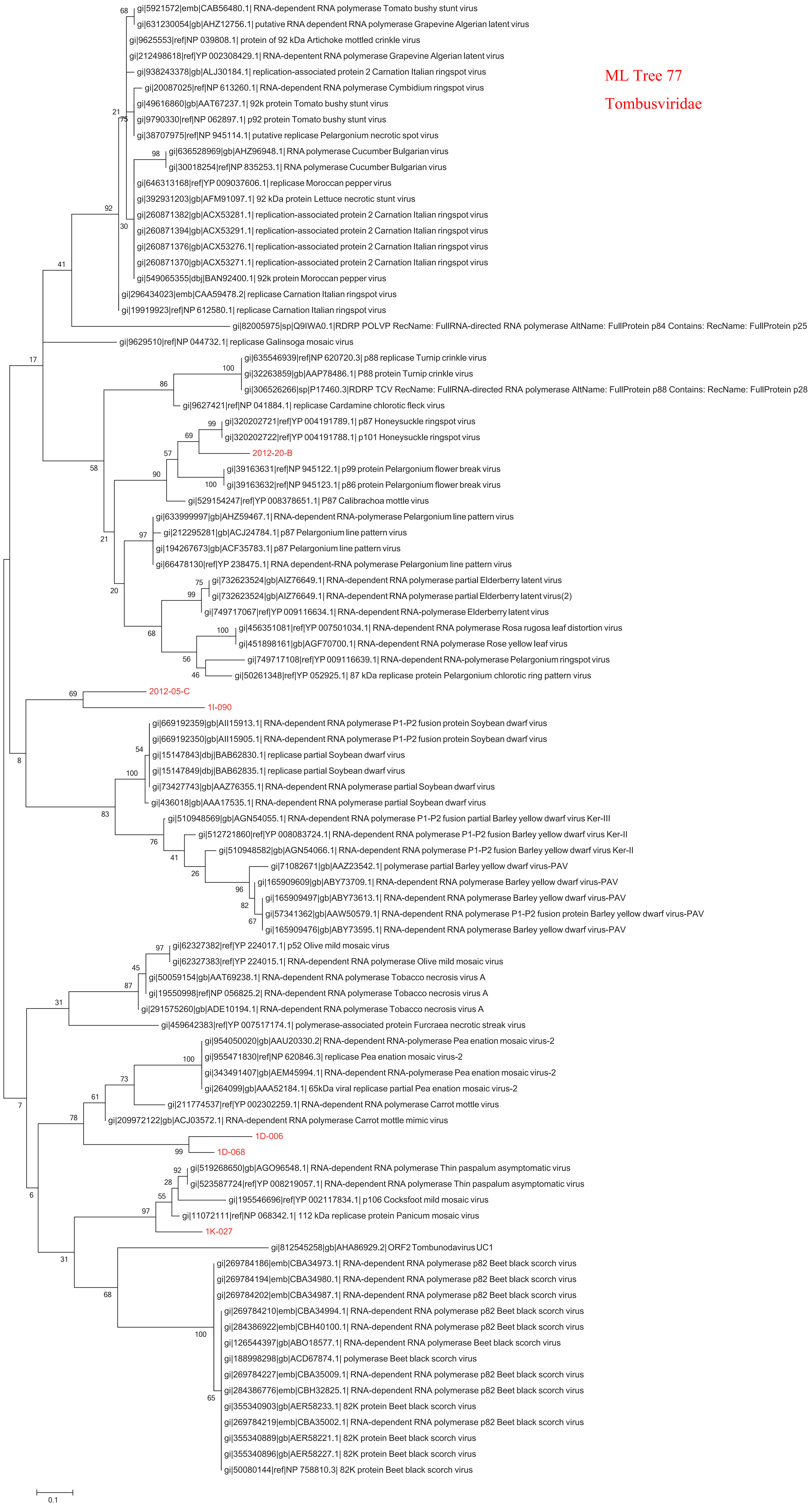
ML Tree 77
Tombusviridae
gi|5921572|emb|CAB56480.1| RNA-dependent RNA polymerase Tomato bushy stunt virus
gi|631230054|gb|AHZ12756.1| putative RNA dependent RNA polymerase Grapevine Algerian latent virus
gi|9625553|ref|NP 039808.1| protein of 92 kDa Artichoke mottled crinkle virus
gi|212498618|ref|YP 002308429.1| RNA-depentent RNA polymerase Grapevine Algerian latent virus
gi|938243378|gb|ALJ30184.1| replication-associated protein 2 Carnation Italian ringspot virus
gi|20087025|ref|NP 613260.1| RNA-dependent RNA polymerase Cymbidium ringspot virus
gi|49616860|gb|AAT67237.1| 92k protein Tomato bushy stunt virus
gi|9790330|ref|NP 062897.1| p92 protein Tomato bushy stunt virus
gi|38707975|ref|NP 945114.1| putative replicase Pelargonium necrotic spot virus
gi|636528969|gb|AHZ96948.1| RNA polymerase Cucumber Bulgarian virus
gi|30018254|ref|NP 835253.1| RNA polymerase Cucumber Bulgarian virus
gi|646313168|ref|YP 009037606.1| replicase Moroccan pepper virus
gi|392931203|gb|AFM91097.1| 92 kDa protein Lettuce necrotic stunt virus
gi|260871382|gb|ACX53281.1| replication-associated protein 2 Carnation Italian ringspot virus
gi|260871394|gb|ACX53291.1| replication-associated protein 2 Carnation Italian ringspot virus
gi|260871376|gb|ACX53276.1| replication-associated protein 2 Carnation Italian ringspot virus
gi|260871370|gb|ACX53271.1| replication-associated protein 2 Carnation Italian ringspot virus
gi|549065355|dbj|BAN92400.1| 92k protein Moroccan pepper virus
gi|296434023|emb|CAA59478.2| replicase Carnation Italian ringspot virus
gi|19919923|ref|NP 612580.1| replicase Carnation Italian ringspot virus
gi|82005975|sp|Q9IWA0.1|RDRP POLVP RecName: FullRNA-directed RNA polymerase AltName: FullProtein p84 Contains: RecName: FullProtein p25
gi|9629510|ref|NP 044732.1| replicase Galinsoga mosaic virus
gi|635546939|ref|NP 620720.3| p88 replicase Turnip crinkle virus
gi|32263859|gb|AAP78486.1| P88 protein Turnip crinkle virus
gi|306526266|sp|P17460.3|RDRP TCV RecName: FullRNA-directed RNA polymerase AltName: FullProtein p88 Contains: RecName: FullProtein p28
gi|9627421|ref|NP 041884.1| replicase Cardamine chlorotic fleck virus
gi|320202721|ref|YP 004191789.1| p87 Honeysuckle ringspot virus
gi|320202722|ref|YP 004191788.1| p101 Honeysuckle ringspot virus
2012-20-B
gi|39163631|ref|NP 945122.1| p99 protein Pelargonium flower break virus
gi|39163632|ref|NP 945123.1| p86 protein Pelargonium flower break virus
gi|529154247|ref|YP 008378651.1| P87 Calibrachoa mottle virus
gi|633999997|gb|AHZ59467.1| RNA-dependent RNA-polymerase Pelargonium line pattern virus
gi|212295281|gb|ACJ24784.1| p87 Pelargonium line pattern virus
gi|194267673|gb|ACF35783.1| p87 Pelargonium line pattern virus
gi|66478130|ref|YP 238475.1| RNA dependent-RNA polymerase Pelargonium line pattern virus
gi|732623524|gb|AIZ76649.1| RNA-dependent RNA polymerase partial Elderberry latent virus
gi|732623524|gb|AIZ76649.1| RNA-dependent RNA polymerase partial Elderberry latent virus(2)
gi|749717067|ref|YP 009116634.1| RNA-dependent RNA-polymerase Elderberry latent virus
gi|456351081|ref|YP 007501034.1| RNA-dependent RNA polymerase Rosa rugosa leaf distortion virus
gi|451898161|gb|AGF70700.1| RNA-dependent RNA polymerase Rose yellow leaf virus
gi|749717108|ref|YP 009116639.1| RNA-dependent RNA-polymerase Pelargonium ringspot virus
gi|50261348|ref|YP 052925.1| 87 kDa replicase protein Pelargonium chlorotic ring pattern virus
2012-05-C
1I-090
gi|669192359|gb|AII15913.1| RNA-dependent RNA polymerase P1-P2 fusion protein Soybean dwarf virus
gi|669192350|gb|AII15905.1| RNA-dependent RNA polymerase P1-P2 fusion protein Soybean dwarf virus
gi|15147843|dbj|BAB62830.1| replicase partial Soybean dwarf virus
gi|15147849|dbj|BAB62835.1| replicase partial Soybean dwarf virus
gi|73427743|gb|AAZ76355.1| RNA-dependent RNA polymerase partial Soybean dwarf virus
gi|436018|gb|AAA17535.1| RNA-dependent RNA polymerase partial Soybean dwarf virus
gi|510948569|gb|AGN54055.1| RNA-dependent RNA polymerase P1-P2 fusion partial Barley yellow dwarf virus Ker-III
gi|512721860|ref|YP 008083724.1| RNA-dependent RNA polymerase P1-P2 fusion Barley yellow dwarf virus Ker-II
gi|510948582|gb|AGN54066.1| RNA-dependent RNA polymerase P1-P2 fusion Barley yellow dwarf virus Ker-II
gi|71082671|gb|AAZ23542.1| polymerase partial Barley yellow dwarf virus-PAV
gi|165909609|gb|ABY73709.1| RNA-dependent RNA polymerase Barley yellow dwarf virus-PAV
gi|165909497|gb|ABY73613.1| RNA-dependent RNA polymerase Barley yellow dwarf virus-PAV
gi|57341362|gb|AAW50579.1| RNA-dependent RNA polymerase P1-P2 fusion protein Barley yellow dwarf virus-PAV
gi|165909476|gb|ABY73595.1| RNA-dependent RNA polymerase Barley yellow dwarf virus-PAV
gi|62327382|ref|YP 224017.1| p52 Olive mild mosaic virus
gi|62327383|ref|YP 224015.1| RNA-dependent RNA polymerase Olive mild mosaic virus
gi|50059154|gb|AAT69238.1| RNA-dependent RNA polymerase Tobacco necrosis virus A
gi|19550998|ref|NP 056825.2| RNA-dependent RNA polymerase Tobacco necrosis virus A
gi|291575260|gb|ADE10194.1| RNA-dependent RNA polymerase Tobacco necrosis virus A
gi|459642383|ref|YP 007517174.1| polymerase-associated protein Furcraea necrotic streak virus
gi|954050020|gb|AAU20330.2| RNA-dependent RNA-polymerase Pea enation mosaic virus-2
gi|955471830|ref|NP 620846.3| replicase Pea enation mosaic virus-2
gi|343491407|gb|AEM45994.1| RNA-dependent RNA-polymerase Pea enation mosaic virus-2
gi|264099|gb|AAA52184.1| 65kDa viral replicase partial Pea enation mosaic virus-2
gi|211774537|ref|YP 002302259.1| RNA-dependent RNA polymerase Carrot mottle virus
gi|209972122|gb|ACJ03572.1| RNA-dependent RNA polymerase Carrot mottle mimic virus
1D-006
1D-068
gi|519268650|gb|AGO96548.1| RNA-dependent RNA polymerase Thin paspalum asymptomatic virus
gi|523587724|ref|YP 008219057.1| RNA-dependent RNA polymerase Thin paspalum asymptomatic virus
gi|195546696|ref|YP 002117834.1| p106 Cocksfoot mild mosaic virus
gi|11072111|ref|NP 068342.1| 112 kDa replicase protein Panicum mosaic virus
1K-027
gi|812545258|gb|AHA86929.2| ORF2 Tombunodavirus UC1
gi|269784186|emb|CBA34973.1| RNA-dependent RNA polymerase p82 Beet black scorch virus
gi|269784194|emb|CBA34980.1| RNA-dependent RNA polymerase p82 Beet black scorch virus
gi|269784202|emb|CBA34987.1| RNA-dependent RNA polymerase p82 Beet black scorch virus
gi|269784210|emb|CBA34994.1| RNA-dependent RNA polymerase p82 Beet black scorch virus
gi|284386922|emb|CBH40100.1| RNA-dependent RNA polymerase p82 Beet black scorch virus
gi|126544397|gb|ABO18577.1| RNA-dependent RNA polymerase Beet black scorch virus
gi|188998298|gb|ACD67874.1| polymerase Beet black scorch virus
gi|269784227|emb|CBA35009.1| RNA-dependent RNA polymerase p82 Beet black scorch virus
gi|284386776|emb|CBH32825.1| RNA-dependent RNA polymerase p82 Beet black scorch virus
gi|355340903|gb|AER58233.1| 82K protein Beet black scorch virus
gi|269784219|emb|CBA35002.1| RNA-dependent RNA polymerase p82 Beet black scorch virus
gi|355340889|gb|AER58221.1| 82K protein Beet black scorch virus
gi|355340896|gb|AER58227.1| 82K protein Beet black scorch virus
gi|50080144|ref|NP 758810.3| 82K protein Beet black scorch virus
0.1

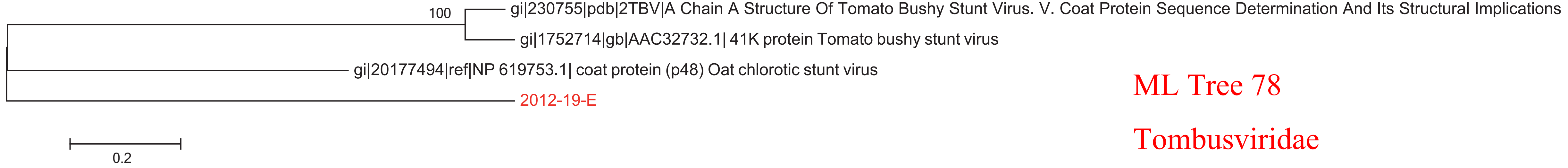
100
gi|230755|pdb|2TBV|A Chain A Structure Of Tomato Bushy Stunt Virus. V. Coat Protein Sequence Determination And Its Structural Implications
gi|1752714|gb|AAC32732.1| 41K protein Tomato bushy stunt virus
gi|20177494|ref|NP 619753.1| coat protein (p48) Oat chlorotic stunt virus
2012-19-E
0.2
ML Tree 78
Tombusviridae

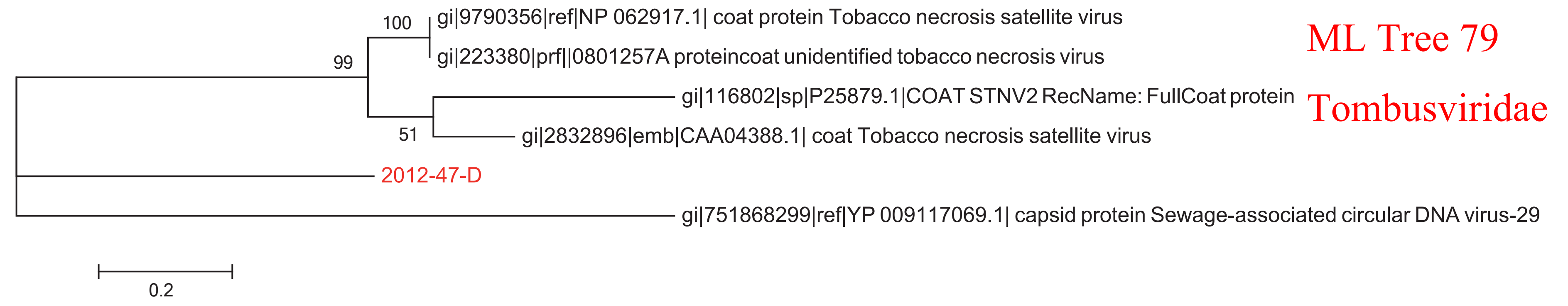
100
gi|9790356|ref|NP 062917.1| coat protein Tobacco necrosis satellite virus
99
gi|223380|prf||0801257A proteincoat unidentified tobacco necrosis virus
gi|116802|sp|P25879.1|COAT STNV2 RecName: FullCoat protein
51
gi|2832896|emb|CAA04388.1| coat Tobacco necrosis satellite virus
2012-47-D
gi|751868299|ref|YP 009117069.1| capsid protein Sewage-associated circular DNA virus-29
0.2
ML Tree 79
Tombusviridae

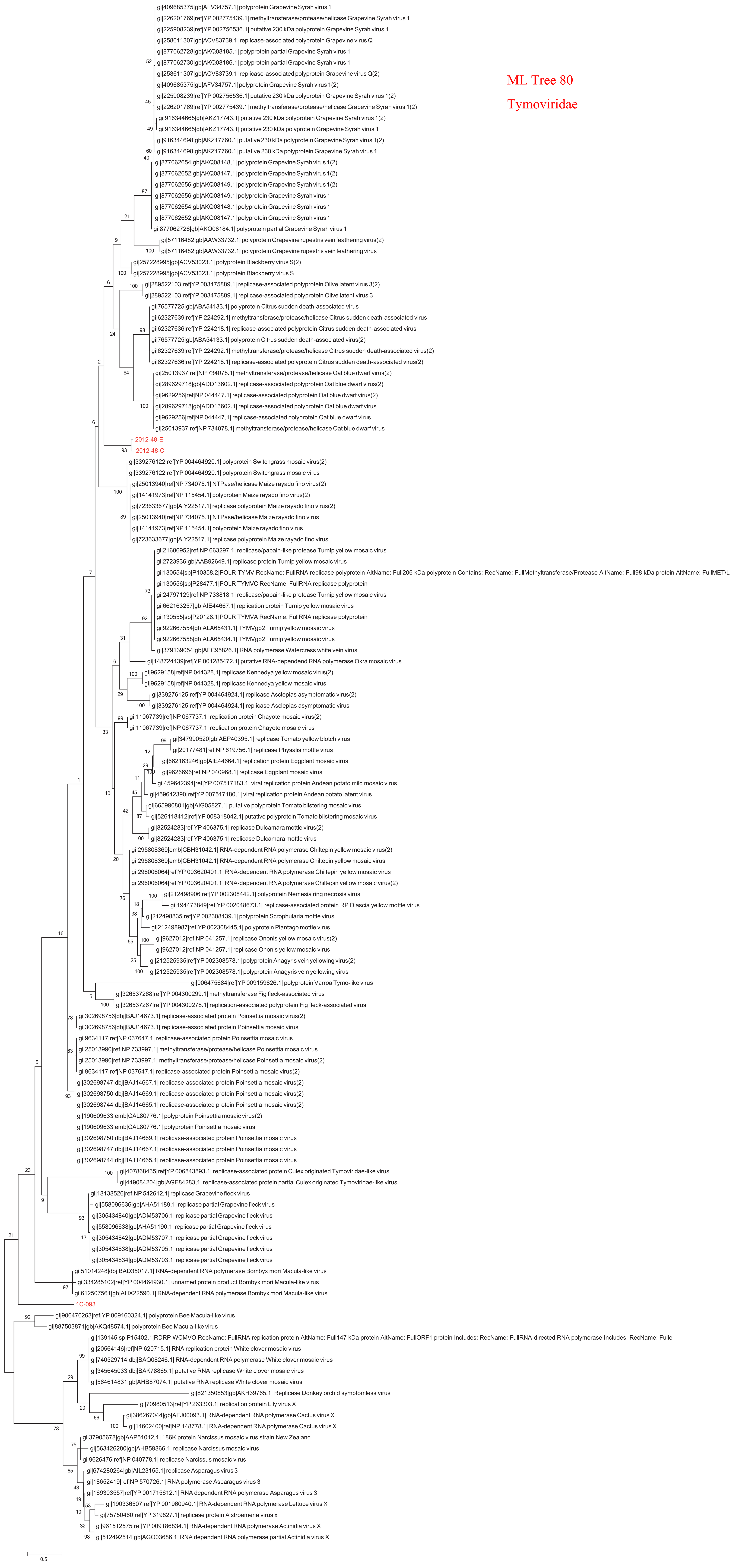
ML Tree 80
Tymoviridae
gi|409685375|gb|AFV34757.1| polyprotein Grapevine Syrah virus 1
gi|226201769|ref|YP 002775439.1| methyltransferase/protease/helicase Grapevine Syrah virus 1
gi|225908239|ref|YP 002756536.1| putative 230 kDa polyprotein Grapevine Syrah virus 1
gi|258611307|gb|ACV83739.1| replicase-associated polyprotein Grapevine virus Q
gi|877062728|gb|AKQ08185.1| polyprotein partial Grapevine Syrah virus 1
gi|877062730|gb|AKQ08186.1| polyprotein partial Grapevine Syrah virus 1
gi|258611307|gb|ACV83739.1| replicase-associated polyprotein Grapevine virus Q(2)
gi|409685375|gb|AFV34757.1| polyprotein Grapevine Syrah virus 1(2)
gi|225908239|ref|YP 002756536.1| putative 230 kDa polyprotein Grapevine Syrah virus 1(2)
gi|226201769|ref|YP 002775439.1| methyltransferase/protease/helicase Grapevine Syrah virus 1(2)
gi|916344665|gb|AKZ17743.1| putative 230 kDa polyprotein Grapevine Syrah virus 1(2)
gi|916344665|gb|AKZ17743.1| putative 230 kDa polyprotein Grapevine Syrah virus 1
gi|916344698|gb|AKZ17760.1| putative 230 kDa polyprotein Grapevine Syrah virus 1(2)
gi|916344698|gb|AKZ17760.1| putative 230 kDa polyprotein Grapevine Syrah virus 1
gi|877062654|gb|AKQ08148.1| polyprotein Grapevine Syrah virus 1(2)
gi|877062652|gb|AKQ08147.1| polyprotein Grapevine Syrah virus 1(2)
gi|877062656|gb|AKQ08149.1| polyprotein Grapevine Syrah virus 1(2)
gi|877062656|gb|AKQ08149.1| polyprotein Grapevine Syrah virus 1
gi|877062654|gb|AKQ08148.1| polyprotein Grapevine Syrah virus 1
gi|877062652|gb|AKQ08147.1| polyprotein Grapevine Syrah virus 1
gi|877062726|gb|AKQ08184.1| polyprotein partial Grapevine Syrah virus 1
gi|57116482|gb|AAW33732.1| polyprotein Grapevine rupestris vein feathering virus(2)
gi|57116482|gb|AAW33732.1| polyprotein Grapevine rupestris vein feathering virus
gi|257228995|gb|ACV53023.1| polyprotein Blackberry virus S(2)
gi|257228995|gb|ACV53023.1| polyprotein Blackberry virus S
gi|289522103|ref|YP 003475889.1| replicase-associated polyprotein Olive latent virus 3(2)
gi|289522103|ref|YP 003475889.1| replicase-associated polyprotein Olive latent virus 3
gi|76577725|gb|ABA54133.1| polyprotein Citrus sudden death-associated virus
gi|62327639|ref|YP 224292.1| methyltransferase/protease/helicase Citrus sudden death-associated virus
gi|62327636|ref|YP 224218.1| replicase-associated polyprotein Citrus sudden death-associated virus
gi|76577725|gb|ABA54133.1| polyprotein Citrus sudden death-associated virus(2)
gi|62327639|ref|YP 224292.1| methyltransferase/protease/helicase Citrus sudden death-associated virus(2)
gi|62327636|ref|YP 224218.1| replicase-associated polyprotein Citrus sudden death-associated virus(2)
gi|25013937|ref|NP 734078.1| methyltransferase/protease/helicase Oat blue dwarf virus(2)
gi|289629718|gb|ADD13602.1| replicase-associated polyprotein Oat blue dwarf virus(2)
gi|9629256|ref|NP 044447.1| replicase-associated polyprotein Oat blue dwarf virus(2)
gi|289629718|gb|ADD13602.1| replicase-associated polyprotein Oat blue dwarf virus
gi|9629256|ref|NP 044447.1| replicase-associated polyprotein Oat blue dwarf virus
gi|25013937|ref|NP 734078.1| methyltransferase/protease/helicase Oat blue dwarf virus
2012-48-E
2012-48-C
gi|339276122|ref|YP 004464920.1| polyprotein Switchgrass mosaic virus(2)
gi|339276122|ref|YP 004464920.1| polyprotein Switchgrass mosaic virus
gi|25013940|ref|NP 734075.1| NTPase/helicase Maize rayado fino virus(2)
gi|14141973|ref|NP 115454.1| polyprotein Maize rayado fino virus(2)
gi|723633677|gb|AIY22517.1| replicase polyprotein Maize rayado fino virus(2)
gi|25013940|ref|NP 734075.1| NTPase/helicase Maize rayado fino virus
gi|14141973|ref|NP 115454.1| polyprotein Maize rayado fino virus
gi|723633677|gb|AIY22517.1| replicase polyprotein Maize rayado fino virus
gi|21686952|ref|NP 663297.1| replicase/papain-like protease Turnip yellow mosaic virus
gi|2723936|gb|AAB92649.1| replicase protein Turnip yellow mosaic virus
gi|130554|sp|P10358.2|POLR TYMV RecName: FullRNA replicase polyprotein AltName: Full206 kDa polyprotein Contains: RecName: FullMethyltransferase/Protease AltName: Full98 kDa protein AltName: FullMET/L
gi|130556|sp|P28477.1|POLR TYMVC RecName: FullRNA replicase polyprotein
gi|24797129|ref|NP 733818.1| replicase/papain-like protease Turnip yellow mosaic virus
gi|662163257|gb|AIE44667.1| replication protein Turnip yellow mosaic virus
gi|130555|sp|P20128.1|POLR TYMVA RecName: FullRNA replicase polyprotein
gi|922667554|gb|ALA65431.1| TYMVgp2 Turnip yellow mosaic virus
gi|922667558|gb|ALA65434.1| TYMVgp2 Turnip yellow mosaic virus
gi|379139054|gb|AFC95826.1| RNA polymerase Watercress white vein virus
gi|148724439|ref|YP 001285472.1| putative RNA-dependend RNA polymerase Okra mosaic virus
gi|9629158|ref|NP 044328.1| replicase Kennedya yellow mosaic virus(2)
gi|9629158|ref|NP 044328.1| replicase Kennedya yellow mosaic virus
gi|339276125|ref|YP 004464924.1| replicase Asclepias asymptomatic virus(2)
gi|339276125|ref|YP 004464924.1| replicase Asclepias asymptomatic virus
gi|11067739|ref|NP 067737.1| replication protein Chayote mosaic virus(2)
gi|11067739|ref|NP 067737.1| replication protein Chayote mosaic virus
gi|347990520|gb|AEP40395.1| replicase Tomato yellow blotch virus
gi|20177481|ref|NP 619756.1| replicase Physalis mottle virus
gi|662163246|gb|AIE44664.1| replication protein Eggplant mosaic virus
gi|9626696|ref|NP 040968.1| replicase Eggplant mosaic virus
gi|459642394|ref|YP 007517183.1| viral replication protein Andean potato mild mosaic virus
gi|459642390|ref|YP 007517180.1| viral replication protein Andean potato latent virus
gi|665990801|gb|AIG05827.1| putative polyprotein Tomato blistering mosaic virus
gi|526118412|ref|YP 008318042.1| putative polyprotein Tomato blistering mosaic virus
gi|82524283|ref|YP 406375.1| replicase Dulcamara mottle virus(2)
gi|82524283|ref|YP 406375.1| replicase Dulcamara mottle virus
gi|295808369|emb|CBH31042.1| RNA-dependent RNA polymerase Chiltepin yellow mosaic virus(2)
gi|295808369|emb|CBH31042.1| RNA-dependent RNA polymerase Chiltepin yellow mosaic virus
gi|296006064|ref|YP 003620401.1| RNA-dependent RNA polymerase Chiltepin yellow mosaic virus
gi|296006064|ref|YP 003620401.1| RNA-dependent RNA polymerase Chiltepin yellow mosaic virus(2)
gi|212498906|ref|YP 002308442.1| polyprotein Nemesia ring necrosis virus
gi|194473849|ref|YP 002048673.1| replicase-associated protein RP Diascia yellow mottle virus
gi|212498835|ref|YP 002308439.1| polyprotein Scrophularia mottle virus
gi|212498987|ref|YP 002308445.1| polyprotein Plantago mottle virus
gi|9627012|ref|NP 041257.1| replicase Ononis yellow mosaic virus(2)
gi|9627012|ref|NP 041257.1| replicase Ononis yellow mosaic virus
gi|212525935|ref|YP 002308578.1| polyprotein Anagyris vein yellowing virus(2)
gi|212525935|ref|YP 002308578.1| polyprotein Anagyris vein yellowing virus
gi|906475684|ref|YP 009159826.1| polyprotein Varroa Tymo-like virus
gi|326537268|ref|YP 004300299.1| methyltransferase Fig fleck-associated virus
gi|326537267|ref|YP 004300278.1| replication-associated polyprotein Fig fleck-associated virus
gi|302698756|dbj|BAJ14673.1| replicase-associated protein Poinsettia mosaic virus(2)
gi|302698756|dbj|BAJ14673.1| replicase-associated protein Poinsettia mosaic virus
gi|9634117|ref|NP 037647.1| replicase-associated protein Poinsettia mosaic virus
gi|25013990|ref|NP 733997.1| methyltransferase/protease/helicase Poinsettia mosaic virus
gi|25013990|ref|NP 733997.1| methyltransferase/protease/helicase Poinsettia mosaic virus(2)
gi|9634117|ref|NP 037647.1| replicase-associated protein Poinsettia mosaic virus(2)
gi|302698747|dbj|BAJ14667.1| replicase-associated protein Poinsettia mosaic virus(2)
gi|302698750|dbj|BAJ14669.1| replicase-associated protein Poinsettia mosaic virus(2)
gi|302698744|dbj|BAJ14665.1| replicase-associated protein Poinsettia mosaic virus(2)
gi|190609633|emb|CAL80776.1| polyprotein Poinsettia mosaic virus(2)
gi|190609633|emb|CAL80776.1| polyprotein Poinsettia mosaic virus
gi|302698750|dbj|BAJ14669.1| replicase-associated protein Poinsettia mosaic virus
gi|302698747|dbj|BAJ14667.1| replicase-associated protein Poinsettia mosaic virus
gi|302698744|dbj|BAJ14665.1| replicase-associated protein Poinsettia mosaic virus
gi|407868435|ref|YP 006843893.1| replicase-associated protein Culex originated Tymoviridae-like virus
gi|449084204|gb|AGE84283.1| replicase-associated protein partial Culex originated Tymoviridae-like virus
gi|18138526|ref|NP 542612.1| replicase Grapevine fleck virus
gi|558096636|gb|AHA51189.1| replicase partial Grapevine fleck virus
gi|305434840|gb|ADM53706.1| replicase partial Grapevine fleck virus
gi|558096638|gb|AHA51190.1| replicase partial Grapevine fleck virus
gi|305434842|gb|ADM53707.1| replicase partial Grapevine fleck virus
gi|305434838|gb|ADM53705.1| replicase partial Grapevine fleck virus
gi|305434834|gb|ADM53703.1| replicase partial Grapevine fleck virus
gi|51014248|dbj|BAD35017.1| RNA-dependent RNA polymerase Bombyx mori Macula-like virus
gi|334285102|ref|YP 004464930.1| unnamed protein product Bombyx mori Macula-like virus
gi|612507561|gb|AHX22590.1| RNA-dependent RNA polymerase Bombyx mori Macula-like virus
1C-093
gi|906476263|ref|YP 009160324.1| polyprotein Bee Macula-like virus
gi|887503871|gb|AKQ48574.1| polyprotein Bee Macula-like virus
gi|139145|sp|P15402.1|RDRP WCMVO RecName: FullRNA replication protein AltName: Full147 kDa protein AltName: FullORF1 protein Includes: RecName: FullRNA-directed RNA polymerase Includes: RecName: Fulle
gi|20564146|ref|NP 620715.1| RNA replication protein White clover mosaic virus
gi|740529714|dbj|BAQ08246.1| RNA-dependent RNA polymerase White clover mosaic virus
gi|345645033|dbj|BAK78865.1| putative RNA replicase White clover mosaic virus
gi|564614831|gb|AHB87074.1| putative RNA replicase White clover mosaic virus
gi|821350853|gb|AKH39765.1| Replicase Donkey orchid symptomless virus
gi|70980513|ref|YP 263303.1| replication protein Lily virus X
gi|386267044|gb|AFJ00093.1| RNA-dependent RNA polymerase Cactus virus X
gi|14602400|ref|NP 148778.1| RNA-dependent RNA polymerase Cactus virus X
gi|37905678|gb|AAP51012.1| 186K protein Narcissus mosaic virus strain New Zealand
gi|563426280|gb|AHB59866.1| replicase Narcissus mosaic virus
gi|9626476|ref|NP 040778.1| replicase Narcissus mosaic virus
gi|674280264|gb|AIL23155.1| replicase Asparagus virus 3
gi|18652419|ref|NP 570726.1| RNA polymerase Asparagus virus 3
gi|169303557|ref|YP 001715612.1| RNA dependent RNA polymerase Asparagus virus 3
gi|190336507|ref|YP 001960940.1| RNA-dependent RNA polymerase Lettuce virus X
gi|75750460|ref|YP 319827.1| replicase protein Alstroemeria virus x
gi|961512575|ref|YP 009186834.1| RNA-dependent RNA polymerase Actinidia virus X
gi|512492514|gb|AGO03686.1| RNA dependent RNA polymerase partial Actinidia virus X
0.5

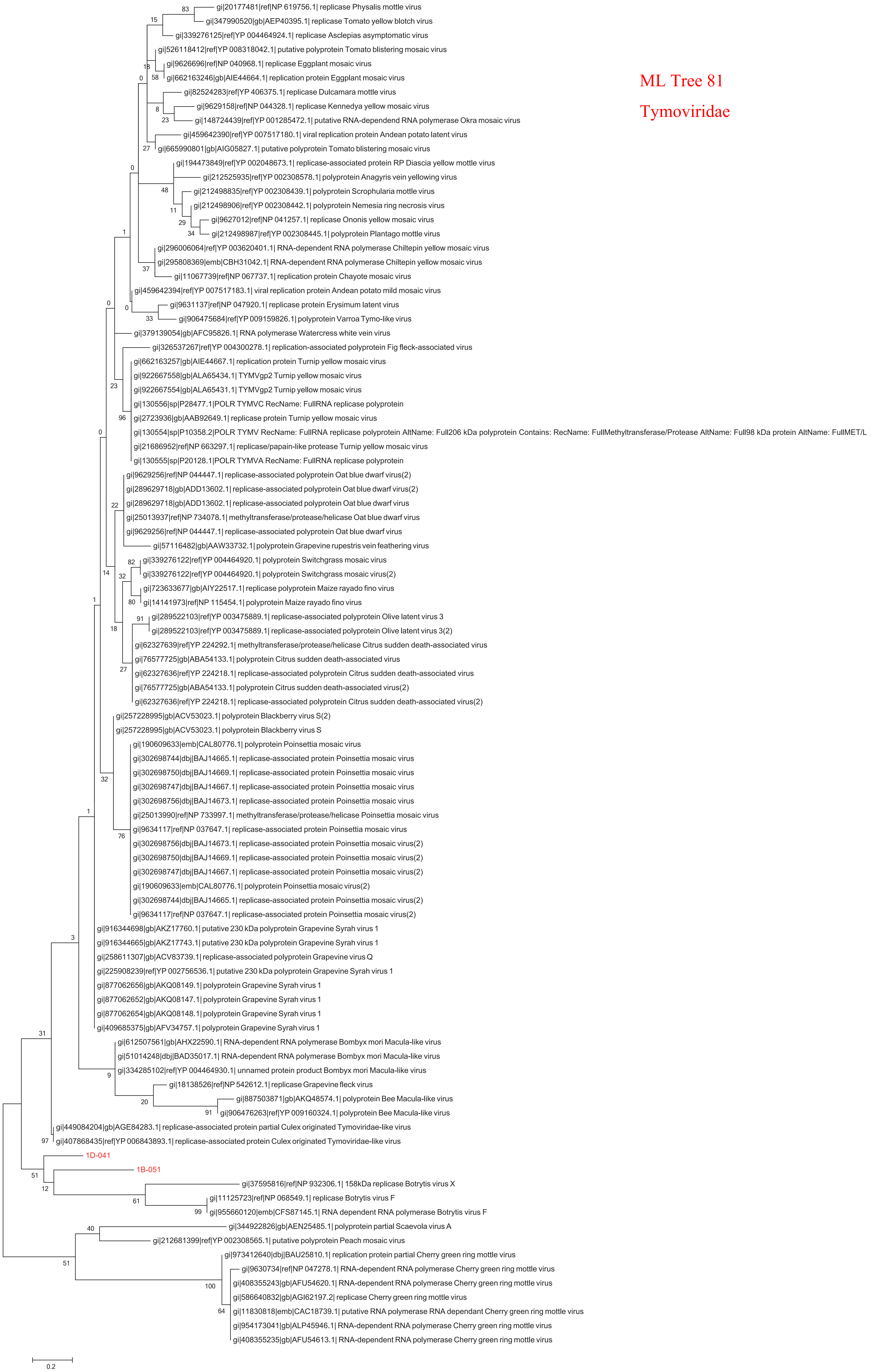
ML Tree 81
Tymoviridae
gi|20177481|ref|NP 619756.1| replicase Physalis mottle virus
gi|347990520|gb|AEP40395.1| replicase Tomato yellow blotch virus
gi|339276125|ref|YP 004464924.1| replicase Asclepias asymptomatic virus
gi|526118412|ref|YP 008318042.1| putative polyprotein Tomato blistering mosaic virus
gi|9626696|ref|NP 040968.1| replicase Eggplant mosaic virus
gi|662163246|gb|AIE44664.1| replication protein Eggplant mosaic virus
gi|82524283|ref|YP 406375.1| replicase Dulcamara mottle virus
gi|9629158|ref|NP 044328.1| replicase Kennedya yellow mosaic virus
gi|148724439|ref|YP 001285472.1| putative RNA-dependend RNA polymerase Okra mosaic virus
gi|459642390|ref|YP 007517180.1| viral replication protein Andean potato latent virus
gi|665990801|gb|AIG05827.1| putative polyprotein Tomato blistering mosaic virus
gi|194473849|ref|YP 002048673.1| replicase-associated protein RP Diascia yellow mottle virus
gi|212525935|ref|YP 002308578.1| polyprotein Anagyris vein yellowing virus
gi|212498835|ref|YP 002308439.1| polyprotein Scrophularia mottle virus
gi|212498906|ref|YP 002308442.1| polyprotein Nemesia ring necrosis virus
gi|9627012|ref|NP 041257.1| replicase Ononis yellow mosaic virus
gi|212498987|ref|YP 002308445.1| polyprotein Plantago mottle virus
gi|296006064|ref|YP 003620401.1| RNA-dependent RNA polymerase Chiltepin yellow mosaic virus
gi|295808369|emb|CBH31042.1| RNA-dependent RNA polymerase Chiltepin yellow mosaic virus
gi|11067739|ref|NP 067737.1| replication protein Chayote mosaic virus
gi|459642394|ref|YP 007517183.1| viral replication protein Andean potato mild mosaic virus
gi|9631137|ref|NP 047920.1| replicase protein Erysimum latent virus
gi|906475684|ref|YP 009159826.1| polyprotein Varroa Tymo-like virus
gi|379139054|gb|AFC95826.1| RNA polymerase Watercress white vein virus
gi|326537267|ref|YP 004300278.1| replication-associated polyprotein Fig fleck-associated virus
gi|662163257|gb|AIE44667.1| replication protein Turnip yellow mosaic virus
gi|922667558|gb|ALA65434.1| TYMVgp2 Turnip yellow mosaic virus
gi|922667554|gb|ALA65431.1| TYMVgp2 Turnip yellow mosaic virus
gi|130556|sp|P28477.1|POLR TYMVC RecName: FullRNA replicase polyprotein
gi|2723936|gb|AAB92649.1| replicase protein Turnip yellow mosaic virus
gi|130554|sp|P10358.2|POLR TYMV RecName: FullRNA replicase polyprotein AltName: Full206 kDa polyprotein Contains: RecName: FullMethyltransferase/Protease AltName: Full98 kDa protein AltName: FullMET/L
gi|21686952|ref|NP 663297.1| replicase/papain-like protease Turnip yellow mosaic virus
gi|130555|sp|P20128.1|POLR TYMVA RecName: FullRNA replicase polyprotein
gi|9629256|ref|NP 044447.1| replicase-associated polyprotein Oat blue dwarf virus(2)
gi|289629718|gb|ADD13602.1| replicase-associated polyprotein Oat blue dwarf virus(2)
gi|289629718|gb|ADD13602.1| replicase-associated polyprotein Oat blue dwarf virus
gi|25013937|ref|NP 734078.1| methyltransferase/protease/helicase Oat blue dwarf virus
gi|9629256|ref|NP 044447.1| replicase-associated polyprotein Oat blue dwarf virus
gi|57116482|gb|AAW33732.1| polyprotein Grapevine rupestris vein feathering virus
gi|339276122|ref|YP 004464920.1| polyprotein Switchgrass mosaic virus
gi|339276122|ref|YP 004464920.1| polyprotein Switchgrass mosaic virus(2)
gi|723633677|gb|AIY22517.1| replicase polyprotein Maize rayado fino virus
gi|14141973|ref|NP 115454.1| polyprotein Maize rayado fino virus
gi|289522103|ref|YP 003475889.1| replicase-associated polyprotein Olive latent virus 3
gi|289522103|ref|YP 003475889.1| replicase-associated polyprotein Olive latent virus 3(2)
gi|62327639|ref|YP 224292.1| methyltransferase/protease/helicase Citrus sudden death-associated virus
gi|76577725|gb|ABA54133.1| polyprotein Citrus sudden death-associated virus
gi|62327636|ref|YP 224218.1| replicase-associated polyprotein Citrus sudden death-associated virus
gi|76577725|gb|ABA54133.1| polyprotein Citrus sudden death-associated virus(2)
gi|62327636|ref|YP 224218.1| replicase-associated polyprotein Citrus sudden death-associated virus(2)
gi|257228995|gb|ACV53023.1| polyprotein Blackberry virus S(2)
gi|257228995|gb|ACV53023.1| polyprotein Blackberry virus S
gi|190609633|emb|CAL80776.1| polyprotein Poinsettia mosaic virus
gi|302698744|dbj|BAJ14665.1| replicase-associated protein Poinsettia mosaic virus
gi|302698750|dbj|BAJ14669.1| replicase-associated protein Poinsettia mosaic virus
gi|302698747|dbj|BAJ14667.1| replicase-associated protein Poinsettia mosaic virus
gi|302698756|dbj|BAJ14673.1| replicase-associated protein Poinsettia mosaic virus
gi|25013990|ref|NP 733997.1| methyltransferase/protease/helicase Poinsettia mosaic virus
gi|9634117|ref|NP 037647.1| replicase-associated protein Poinsettia mosaic virus
gi|302698756|dbj|BAJ14673.1| replicase-associated protein Poinsettia mosaic virus(2)
gi|302698750|dbj|BAJ14669.1| replicase-associated protein Poinsettia mosaic virus(2)
gi|302698747|dbj|BAJ14667.1| replicase-associated protein Poinsettia mosaic virus(2)
gi|190609633|emb|CAL80776.1| polyprotein Poinsettia mosaic virus(2)
gi|302698744|dbj|BAJ14665.1| replicase-associated protein Poinsettia mosaic virus(2)
gi|9634117|ref|NP 037647.1| replicase-associated protein Poinsettia mosaic virus(2)
gi|916344698|gb|AKZ17760.1| putative 230 kDa polyprotein Grapevine Syrah virus 1
gi|916344665|gb|AKZ17743.1| putative 230 kDa polyprotein Grapevine Syrah virus 1
gi|258611307|gb|ACV83739.1| replicase-associated polyprotein Grapevine virus Q
gi|225908239|ref|YP 002756536.1| putative 230 kDa polyprotein Grapevine Syrah virus 1
gi|877062656|gb|AKQ08149.1| polyprotein Grapevine Syrah virus 1
gi|877062652|gb|AKQ08147.1| polyprotein Grapevine Syrah virus 1
gi|877062654|gb|AKQ08148.1| polyprotein Grapevine Syrah virus 1
gi|409685375|gb|AFV34757.1| polyprotein Grapevine Syrah virus 1
gi|612507561|gb|AHX22590.1| RNA-dependent RNA polymerase Bombyx mori Macula-like virus
gi|51014248|dbj|BAD35017.1| RNA-dependent RNA polymerase Bombyx mori Macula-like virus
gi|334285102|ref|YP 004464930.1| unnamed protein product Bombyx mori Macula-like virus
gi|18138526|ref|NP 542612.1| replicase Grapevine fleck virus
gi|887503871|gb|AKQ48574.1| polyprotein Bee Macula-like virus
gi|906476263|ref|YP 009160324.1| polyprotein Bee Macula-like virus
gi|449084204|gb|AGE84283.1| replicase-associated protein partial Culex originated Tymoviridae-like virus
gi|407868435|ref|YP 006843893.1| replicase-associated protein Culex originated Tymoviridae-like virus
1D-041
1B-051
gi|37595816|ref|NP 932306.1| 158kDa replicase Botrytis virus X
gi|11125723|ref|NP 068549.1| replicase Botrytis virus F
gi|955660120|emb|CFS87145.1| RNA dependent RNA polymerase Botrytis virus F
gi|344922826|gb|AEN25485.1| polyprotein partial Scaevola virus A
gi|212681399|ref|YP 002308565.1| putative polyprotein Peach mosaic virus
gi|973412640|dbj|BAU25810.1| replication protein partial Cherry green ring mottle virus
gi|9630734|ref|NP 047278.1| RNA-dependent RNA polymerase Cherry green ring mottle virus
gi|408355243|gb|AFU54620.1| RNA-dependent RNA polymerase Cherry green ring mottle virus
gi|586640832|gb|AGI62197.2| replicase Cherry green ring mottle virus
gi|11830818|emb|CAC18739.1| putative RNA polymerase RNA dependant Cherry green ring mottle virus
gi|954173041|gb|ALP45946.1| RNA-dependent RNA polymerase Cherry green ring mottle virus
gi|408355235|gb|AFU54613.1| RNA-dependent RNA polymerase Cherry green ring mottle virus
0.2

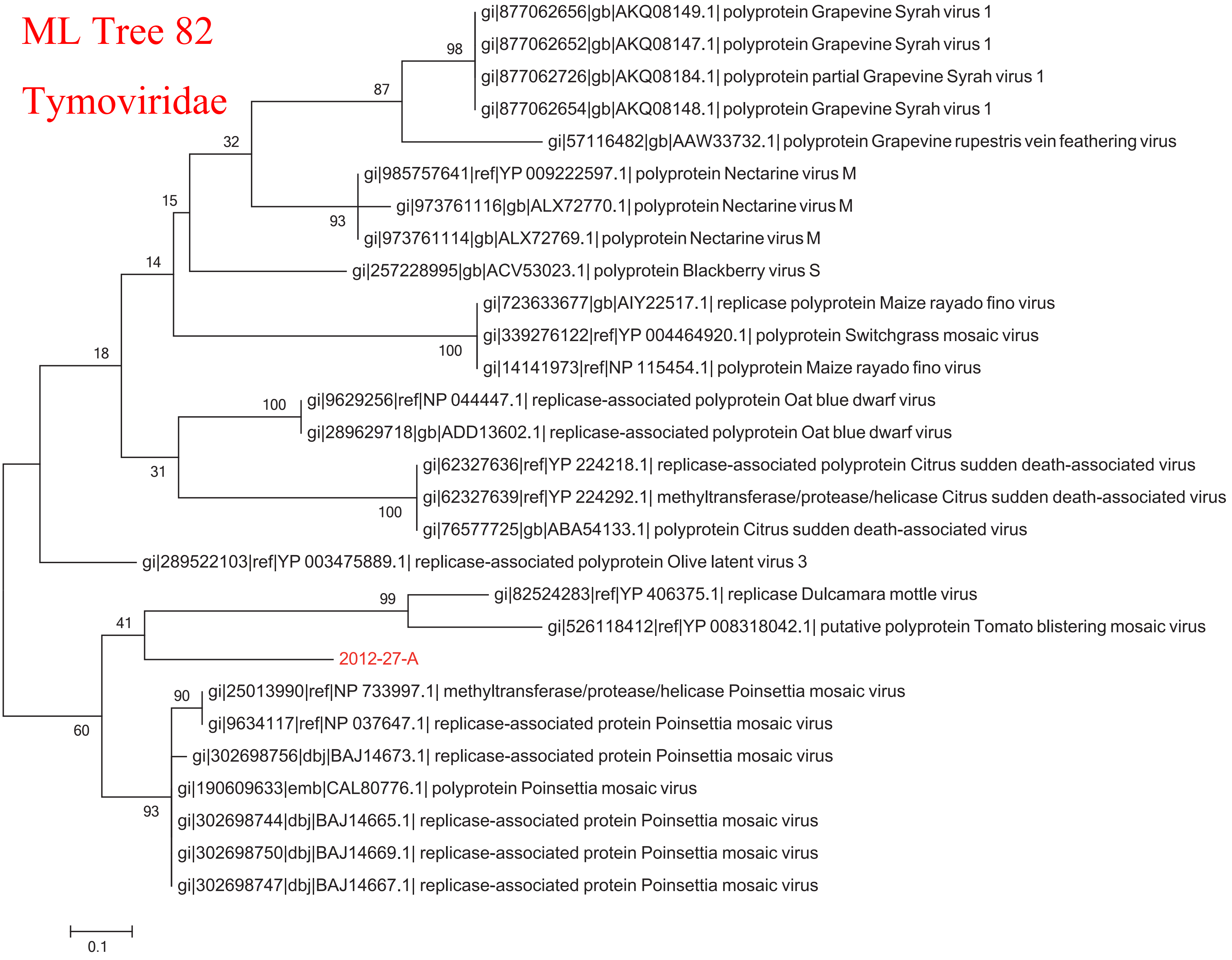
ML Tree 82
Tymoviridae
gi|877062656|gb|AKQ08149.1| polyprotein Grapevine Syrah virus 1
gi|877062652|gb|AKQ08147.1| polyprotein Grapevine Syrah virus 1
gi|877062726|gb|AKQ08184.1| polyprotein partial Grapevine Syrah virus 1
gi|877062654|gb|AKQ08148.1| polyprotein Grapevine Syrah virus 1
gi|57116482|gb|AAW33732.1| polyprotein Grapevine rupestris vein feathering virus
gi|985757641|ref|YP 009222597.1| polyprotein Nectarine virus M
gi|973761116|gb|ALX72770.1| polyprotein Nectarine virus M
gi|973761114|gb|ALX72769.1| polyprotein Nectarine virus M
gi|257228995|gb|ACV53023.1| polyprotein Blackberry virus S
gi|723633677|gb|AIY22517.1| replicase polyprotein Maize rayado fino virus
gi|339276122|ref|YP 004464920.1| polyprotein Switchgrass mosaic virus
gi|14141973|ref|NP 115454.1| polyprotein Maize rayado fino virus
gi|9629256|ref|NP 044447.1| replicase-associated polyprotein Oat blue dwarf virus
gi|289629718|gb|ADD13602.1| replicase-associated polyprotein Oat blue dwarf virus
gi|62327636|ref|YP 224218.1| replicase-associated polyprotein Citrus sudden death-associated virus
gi|62327639|ref|YP 224292.1| methyltransferase/protease/helicase Citrus sudden death-associated virus
gi|76577725|gb|ABA54133.1| polyprotein Citrus sudden death-associated virus
gi|289522103|ref|YP 003475889.1| replicase-associated polyprotein Olive latent virus 3
gi|82524283|ref|YP 406375.1| replicase Dulcamara mottle virus
gi|526118412|ref|YP 008318042.1| putative polyprotein Tomato blistering mosaic virus
2012-27-A
gi|25013990|ref|NP 733997.1| methyltransferase/protease/helicase Poinsettia mosaic virus
gi|9634117|ref|NP 037647.1| replicase-associated protein Poinsettia mosaic virus
gi|302698756|dbj|BAJ14673.1| replicase-associated protein Poinsettia mosaic virus
gi|190609633|emb|CAL80776.1| polyprotein Poinsettia mosaic virus
gi|302698744|dbj|BAJ14665.1| replicase-associated protein Poinsettia mosaic virus
gi|302698750|dbj|BAJ14669.1| replicase-associated protein Poinsettia mosaic virus
gi|302698747|dbj|BAJ14667.1| replicase-associated protein Poinsettia mosaic virus
98
87
32
15
93
14
100
18
100
31
100
99
41
90
60
93
0.1

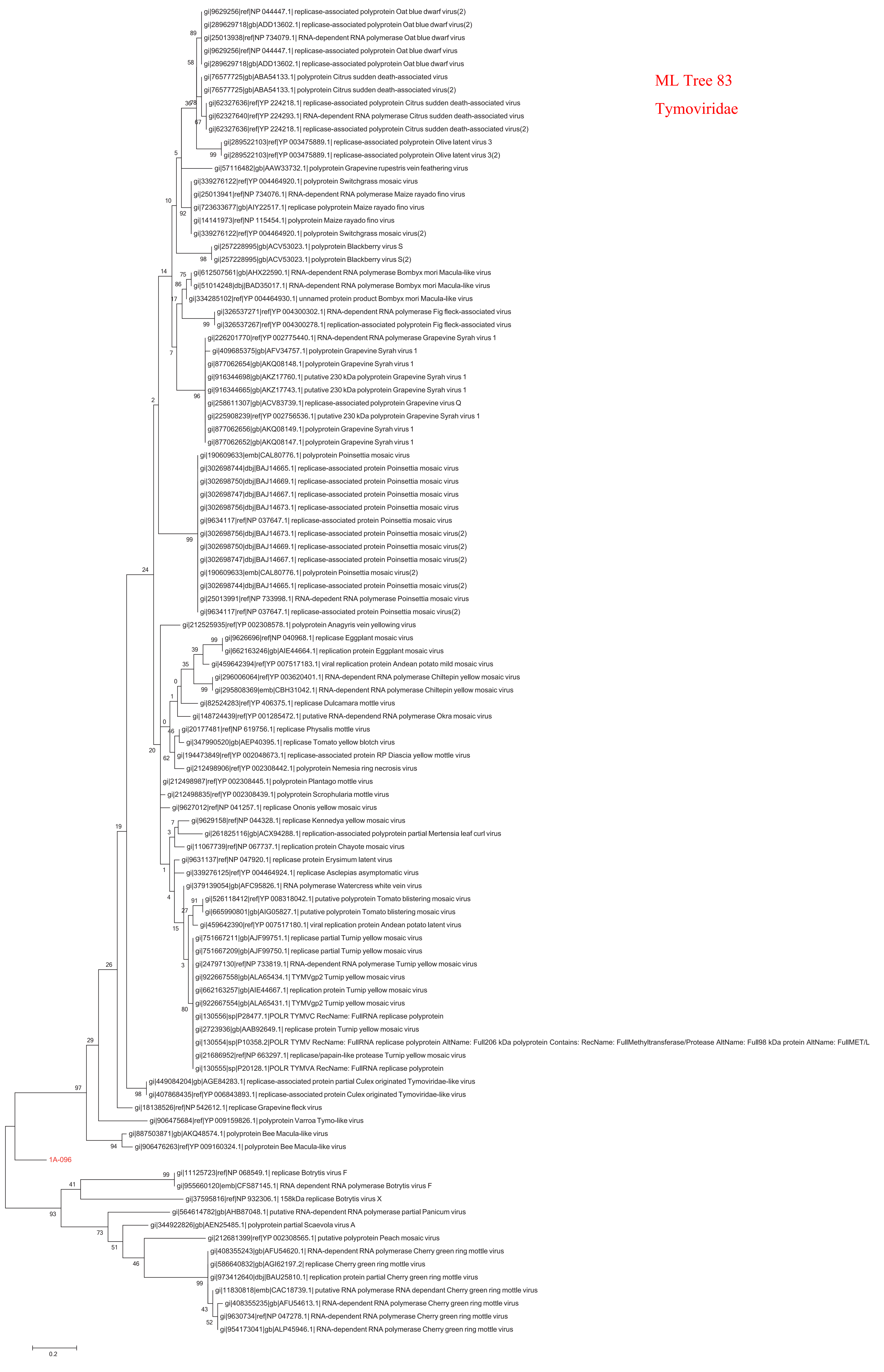
ML Tree 83
Tymoviridae
gi|9629256|ref|NP 044447.1| replicase-associated polyprotein Oat blue dwarf virus(2)
gi|289629718|gb|ADD13602.1| replicase-associated polyprotein Oat blue dwarf virus(2)
gi|25013938|ref|NP 734079.1| RNA-dependent RNA polymerase Oat blue dwarf virus
gi|9629256|ref|NP 044447.1| replicase-associated polyprotein Oat blue dwarf virus
gi|289629718|gb|ADD13602.1| replicase-associated polyprotein Oat blue dwarf virus
gi|76577725|gb|ABA54133.1| polyprotein Citrus sudden death-associated virus
gi|76577725|gb|ABA54133.1| polyprotein Citrus sudden death-associated virus(2)
gi|62327636|ref|YP 224218.1| replicase-associated polyprotein Citrus sudden death-associated virus
gi|62327640|ref|YP 224293.1| RNA-dependent RNA polymerase Citrus sudden death-associated virus
gi|62327636|ref|YP 224218.1| replicase-associated polyprotein Citrus sudden death-associated virus(2)
gi|289522103|ref|YP 003475889.1| replicase-associated polyprotein Olive latent virus 3
gi|289522103|ref|YP 003475889.1| replicase-associated polyprotein Olive latent virus 3(2)
gi|57116482|gb|AAW33732.1| polyprotein Grapevine rupestris vein feathering virus
gi|339276122|ref|YP 004464920.1| polyprotein Switchgrass mosaic virus
gi|25013941|ref|NP 734076.1| RNA-dependent RNA polymerase Maize rayado fino virus
gi|723633677|gb|AIY22517.1| replicase polyprotein Maize rayado fino virus
gi|14141973|ref|NP 115454.1| polyprotein Maize rayado fino virus
gi|339276122|ref|YP 004464920.1| polyprotein Switchgrass mosaic virus(2)
gi|257228995|gb|ACV53023.1| polyprotein Blackberry virus S
gi|257228995|gb|ACV53023.1| polyprotein Blackberry virus S(2)
gi|612507561|gb|AHX22590.1| RNA-dependent RNA polymerase Bombyx mori Macula-like virus
gi|51014248|dbj|BAD35017.1| RNA-dependent RNA polymerase Bombyx mori Macula-like virus
gi|334285102|ref|YP 004464930.1| unnamed protein product Bombyx mori Macula-like virus
gi|326537271|ref|YP 004300302.1| RNA-dependent RNA polymerase Fig fleck-associated virus
gi|326537267|ref|YP 004300278.1| replication-associated polyprotein Fig fleck-associated virus
gi|226201770|ref|YP 002775440.1| RNA-dependent RNA polymerase Grapevine Syrah virus 1
gi|409685375|gb|AFV34757.1| polyprotein Grapevine Syrah virus 1
gi|877062654|gb|AKQ08148.1| polyprotein Grapevine Syrah virus 1
gi|916344698|gb|AKZ17760.1| putative 230 kDa polyprotein Grapevine Syrah virus 1
gi|916344665|gb|AKZ17743.1| putative 230 kDa polyprotein Grapevine Syrah virus 1
gi|258611307|gb|ACV83739.1| replicase-associated polyprotein Grapevine virus Q
gi|225908239|ref|YP 002756536.1| putative 230 kDa polyprotein Grapevine Syrah virus 1
gi|877062656|gb|AKQ08149.1| polyprotein Grapevine Syrah virus 1
gi|877062652|gb|AKQ08147.1| polyprotein Grapevine Syrah virus 1
gi|190609633|emb|CAL80776.1| polyprotein Poinsettia mosaic virus
gi|302698744|dbj|BAJ14665.1| replicase-associated protein Poinsettia mosaic virus
gi|302698750|dbj|BAJ14669.1| replicase-associated protein Poinsettia mosaic virus
gi|302698747|dbj|BAJ14667.1| replicase-associated protein Poinsettia mosaic virus
gi|302698756|dbj|BAJ14673.1| replicase-associated protein Poinsettia mosaic virus
gi|9634117|ref|NP 037647.1| replicase-associated protein Poinsettia mosaic virus
gi|302698756|dbj|BAJ14673.1| replicase-associated protein Poinsettia mosaic virus(2)
gi|302698750|dbj|BAJ14669.1| replicase-associated protein Poinsettia mosaic virus(2)
gi|302698747|dbj|BAJ14667.1| replicase-associated protein Poinsettia mosaic virus(2)
gi|190609633|emb|CAL80776.1| polyprotein Poinsettia mosaic virus(2)
gi|302698744|dbj|BAJ14665.1| replicase-associated protein Poinsettia mosaic virus(2)
gi|25013991|ref|NP 733998.1| RNA-depedent RNA polymerase Poinsettia mosaic virus
gi|9634117|ref|NP 037647.1| replicase-associated protein Poinsettia mosaic virus(2)
gi|212525935|ref|YP 002308578.1| polyprotein Anagyris vein yellowing virus
gi|9626696|ref|NP 040968.1| replicase Eggplant mosaic virus
gi|662163246|gb|AIE44664.1| replication protein Eggplant mosaic virus
gi|459642394|ref|YP 007517183.1| viral replication protein Andean potato mild mosaic virus
gi|296006064|ref|YP 003620401.1| RNA-dependent RNA polymerase Chiltepin yellow mosaic virus
gi|295808369|emb|CBH31042.1| RNA-dependent RNA polymerase Chiltepin yellow mosaic virus
gi|82524283|ref|YP 406375.1| replicase Dulcamara mottle virus
gi|148724439|ref|YP 001285472.1| putative RNA-dependend RNA polymerase Okra mosaic virus
gi|20177481|ref|NP 619756.1| replicase Physalis mottle virus
gi|347990520|gb|AEP40395.1| replicase Tomato yellow blotch virus
gi|194473849|ref|YP 002048673.1| replicase-associated protein RP Diascia yellow mottle virus
gi|212498906|ref|YP 002308442.1| polyprotein Nemesia ring necrosis virus
gi|212498987|ref|YP 002308445.1| polyprotein Plantago mottle virus
gi|212498835|ref|YP 002308439.1| polyprotein Scrophularia mottle virus
gi|9627012|ref|NP 041257.1| replicase Ononis yellow mosaic virus
gi|9629158|ref|NP 044328.1| replicase Kennedya yellow mosaic virus
gi|261825116|gb|ACX94288.1| replication-associated polyprotein partial Mertensia leaf curl virus
gi|11067739|ref|NP 067737.1| replication protein Chayote mosaic virus
gi|9631137|ref|NP 047920.1| replicase protein Erysimum latent virus
gi|339276125|ref|YP 004464924.1| replicase Asclepias asymptomatic virus
gi|379139054|gb|AFC95826.1| RNA polymerase Watercress white vein virus
gi|526118412|ref|YP 008318042.1| putative polyprotein Tomato blistering mosaic virus
gi|665990801|gb|AIG05827.1| putative polyprotein Tomato blistering mosaic virus
gi|459642390|ref|YP 007517180.1| viral replication protein Andean potato latent virus
gi|751667211|gb|AJF99751.1| replicase partial Turnip yellow mosaic virus
gi|751667209|gb|AJF99750.1| replicase partial Turnip yellow mosaic virus
gi|24797130|ref|NP 733819.1| RNA-dependent RNA polymerase Turnip yellow mosaic virus
gi|922667558|gb|ALA65434.1| TYMVgp2 Turnip yellow mosaic virus
gi|662163257|gb|AIE44667.1| replication protein Turnip yellow mosaic virus
gi|922667554|gb|ALA65431.1| TYMVgp2 Turnip yellow mosaic virus
gi|130556|sp|P28477.1|POLR TYMVC RecName: FullRNA replicase polyprotein
gi|2723936|gb|AAB92649.1| replicase protein Turnip yellow mosaic virus
gi|130554|sp|P10358.2|POLR TYMV RecName: FullRNA replicase polyprotein AltName: Full206 kDa polyprotein Contains: RecName: FullMethyltransferase/Protease AltName: Full98 kDa protein AltName: FullMET/L
gi|21686952|ref|NP 663297.1| replicase/papain-like protease Turnip yellow mosaic virus
gi|130555|sp|P20128.1|POLR TYMVA RecName: FullRNA replicase polyprotein
gi|449084204|gb|AGE84283.1| replicase-associated protein partial Culex originated Tymoviridae-like virus
gi|407868435|ref|YP 006843893.1| replicase-associated protein Culex originated Tymoviridae-like virus
gi|18138526|ref|NP 542612.1| replicase Grapevine fleck virus
gi|906475684|ref|YP 009159826.1| polyprotein Varroa Tymo-like virus
gi|887503871|gb|AKQ48574.1| polyprotein Bee Macula-like virus
gi|906476263|ref|YP 009160324.1| polyprotein Bee Macula-like virus
1A-096
gi|11125723|ref|NP 068549.1| replicase Botrytis virus F
gi|955660120|emb|CFS87145.1| RNA dependent RNA polymerase Botrytis virus F
gi|37595816|ref|NP 932306.1| 158kDa replicase Botrytis virus X
gi|564614782|gb|AHB87048.1| putative RNA-dependent RNA polymerase partial Panicum virus
gi|344922826|gb|AEN25485.1| polyprotein partial Scaevola virus A
gi|212681399|ref|YP 002308565.1| putative polyprotein Peach mosaic virus
gi|408355243|gb|AFU54620.1| RNA-dependent RNA polymerase Cherry green ring mottle virus
gi|586640832|gb|AGI62197.2| replicase Cherry green ring mottle virus
gi|973412640|dbj|BAU25810.1| replication protein partial Cherry green ring mottle virus
gi|11830818|emb|CAC18739.1| putative RNA polymerase RNA dependant Cherry green ring mottle virus
gi|408355235|gb|AFU54613.1| RNA-dependent RNA polymerase Cherry green ring mottle virus
gi|9630734|ref|NP 047278.1| RNA-dependent RNA polymerase Cherry green ring mottle virus
gi|954173041|gb|ALP45946.1| RNA-dependent RNA polymerase Cherry green ring mottle virus
0.2

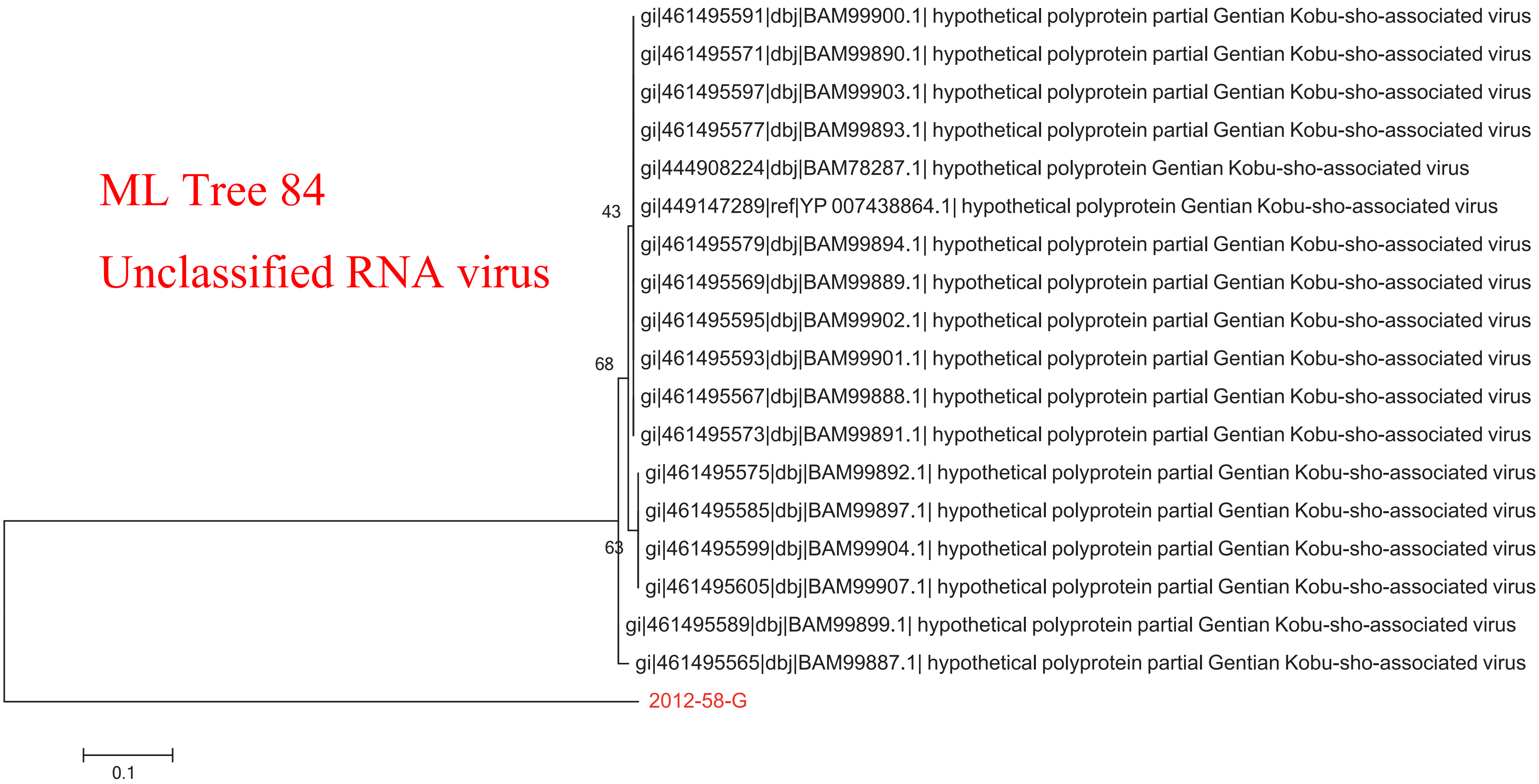

ML Tree 84
Unclassified RNA virus
gi|461495591|dbj|BAM99900.1| hypothetical polyprotein partial Gentian Kobu-sho-associated virus
gi|461495571|dbj|BAM99890.1| hypothetical polyprotein partial Gentian Kobu-sho-associated virus
gi|461495597|dbj|BAM99903.1| hypothetical polyprotein partial Gentian Kobu-sho-associated virus
gi|461495577|dbj|BAM99893.1| hypothetical polyprotein partial Gentian Kobu-sho-associated virus
gi|444908224|dbj|BAM78287.1| hypothetical polyprotein Gentian Kobu-sho-associated virus
43
gi|449147289|ref|YP 007438864.1| hypothetical polyprotein Gentian Kobu-sho-associated virus
gi|461495579|dbj|BAM99894.1| hypothetical polyprotein partial Gentian Kobu-sho-associated virus
gi|461495569|dbj|BAM99889.1| hypothetical polyprotein partial Gentian Kobu-sho-associated virus
gi|461495595|dbj|BAM99902.1| hypothetical polyprotein partial Gentian Kobu-sho-associated virus
68
gi|461495593|dbj|BAM99901.1| hypothetical polyprotein partial Gentian Kobu-sho-associated virus
gi|461495567|dbj|BAM99888.1| hypothetical polyprotein partial Gentian Kobu-sho-associated virus
gi|461495573|dbj|BAM99891.1| hypothetical polyprotein partial Gentian Kobu-sho-associated virus
gi|461495575|dbj|BAM99892.1| hypothetical polyprotein partial Gentian Kobu-sho-associated virus
gi|461495585|dbj|BAM99897.1| hypothetical polyprotein partial Gentian Kobu-sho-associated virus
63
gi|461495599|dbj|BAM99904.1| hypothetical polyprotein partial Gentian Kobu-sho-associated virus
gi|461495605|dbj|BAM99907.1| hypothetical polyprotein partial Gentian Kobu-sho-associated virus
gi|461495589|dbj|BAM99899.1| hypothetical polyprotein partial Gentian Kobu-sho-associated virus
gi|461495565|dbj|BAM99887.1| hypothetical polyprotein partial Gentian Kobu-sho-associated virus
2012-58-G
0.1

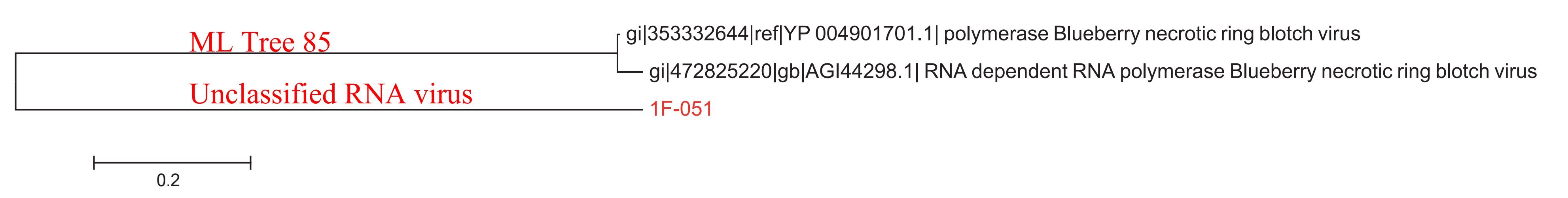

ML Tree 85
Unclassified RNA virus
gi|353332644|ref|YP 004901701.1| polymerase Blueberry necrotic ring blotch virus
gi|472825220|gb|AGI44298.1| RNA dependent RNA polymerase Blueberry necrotic ring blotch virus
1F-051
0.2

ML Tree 86

Virgaviridae

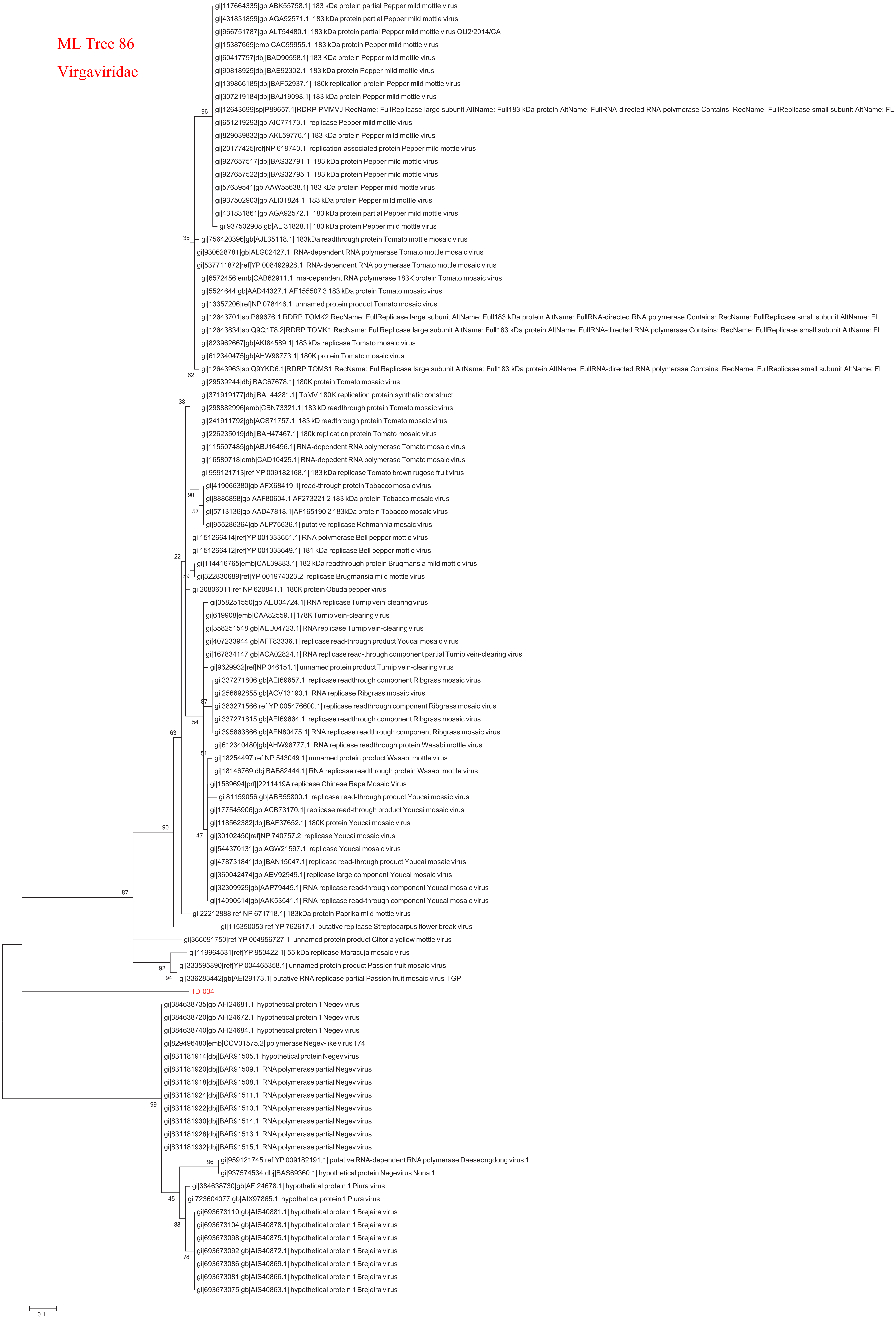

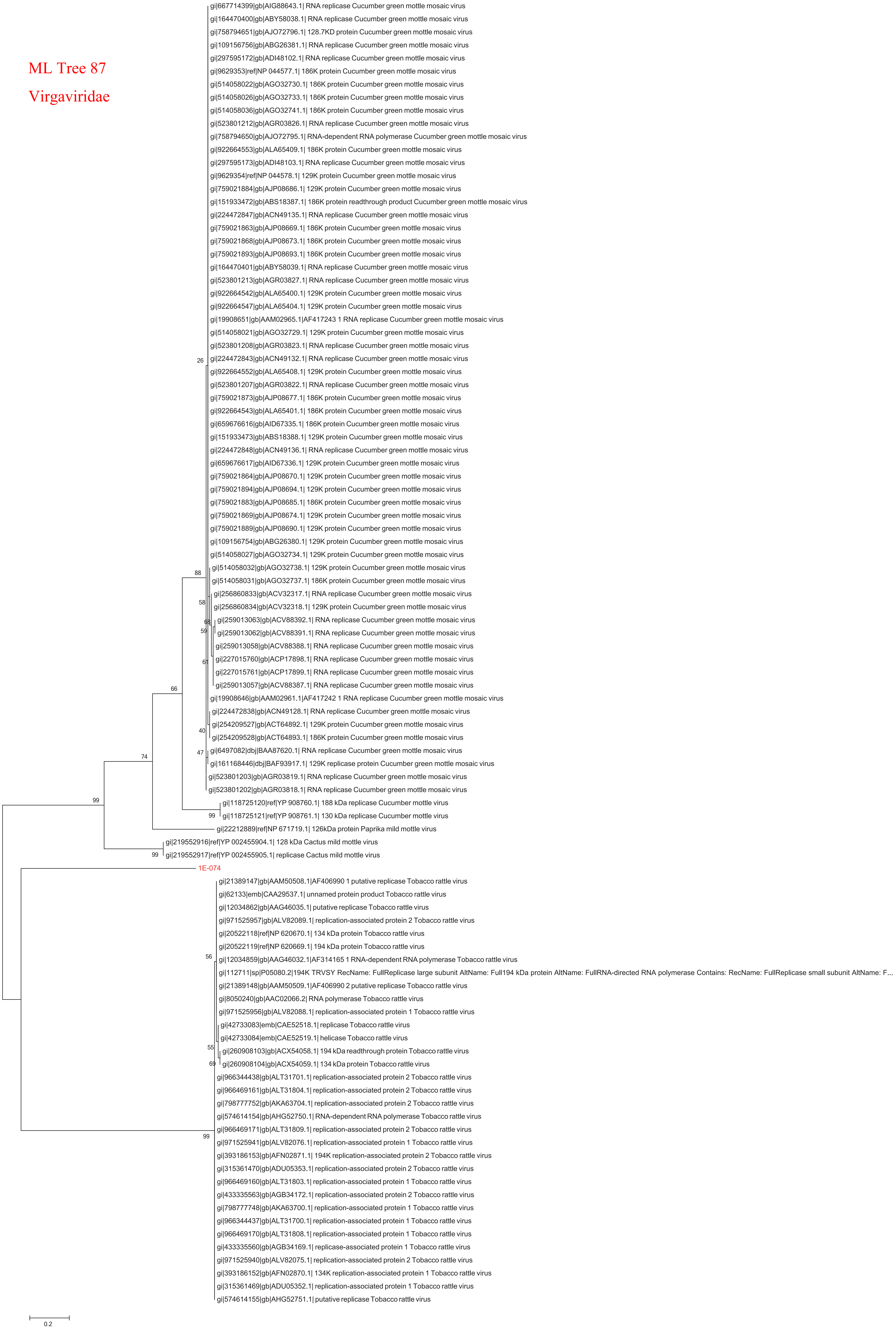

ML Tree 87
Virgaviridae
gi|667714399|gb|AIG88643.1| RNA replicase Cucumber green mottle mosaic virus
gi|164470400|gb|ABY58038.1| RNA replicase Cucumber green mottle mosaic virus
gi|758794651|gb|AJO72796.1| 128.7KD protein Cucumber green mottle mosaic virus
gi|109156756|gb|ABG26381.1| RNA replicase Cucumber green mottle mosaic virus
gi|297595172|gb|ADI48102.1| RNA replicase Cucumber green mottle mosaic virus
gi|9629353|ref|NP 044577.1| 186K protein Cucumber green mottle mosaic virus
gi|514058022|gb|AGO32730.1| 186K protein Cucumber green mottle mosaic virus
gi|514058026|gb|AGO32733.1| 186K protein Cucumber green mottle mosaic virus
gi|514058036|gb|AGO32741.1| 186K protein Cucumber green mottle mosaic virus
gi|523801212|gb|AGR03826.1| RNA replicase Cucumber green mottle mosaic virus
gi|758794650|gb|AJO72795.1| RNA-dependent RNA polymerase Cucumber green mottle mosaic virus
gi|922664553|gb|ALA65409.1| 186K protein Cucumber green mottle mosaic virus
gi|297595173|gb|ADI48103.1| RNA replicase Cucumber green mottle mosaic virus
gi|9629354|ref|NP 044578.1| 129K protein Cucumber green mottle mosaic virus
gi|759021884|gb|AJP08686.1| 129K protein Cucumber green mottle mosaic virus
gi|151933472|gb|ABS18387.1| 186K protein readthrough product Cucumber green mottle mosaic virus
gi|224472847|gb|ACN49135.1| RNA replicase Cucumber green mottle mosaic virus
gi|759021863|gb|AJP08669.1| 186K protein Cucumber green mottle mosaic virus
gi|759021868|gb|AJP08673.1| 186K protein Cucumber green mottle mosaic virus
gi|759021893|gb|AJP08693.1| 186K protein Cucumber green mottle mosaic virus
gi|164470401|gb|ABY58039.1| RNA replicase Cucumber green mottle mosaic virus
gi|523801213|gb|AGR03827.1| RNA replicase Cucumber green mottle mosaic virus
gi|922664542|gb|ALA65400.1| 129K protein Cucumber green mottle mosaic virus
gi|922664547|gb|ALA65404.1| 129K protein Cucumber green mottle mosaic virus
gi|19908651|gb|AAM02965.1|AF417243 1 RNA replicase Cucumber green mottle mosaic virus
gi|514058021|gb|AGO32729.1| 129K protein Cucumber green mottle mosaic virus
gi|523801208|gb|AGR03823.1| RNA replicase Cucumber green mottle mosaic virus
26
gi|224472843|gb|ACN49132.1| RNA replicase Cucumber green mottle mosaic virus
gi|922664552|gb|ALA65408.1| 129K protein Cucumber green mottle mosaic virus
gi|523801207|gb|AGR03822.1| RNA replicase Cucumber green mottle mosaic virus
gi|759021873|gb|AJP08677.1| 186K protein Cucumber green mottle mosaic virus
gi|922664543|gb|ALA65401.1| 186K protein Cucumber green mottle mosaic virus
gi|659676616|gb|AID67335.1| 186K protein Cucumber green mottle mosaic virus
gi|151933473|gb|ABS18388.1| 129K protein Cucumber green mottle mosaic virus
gi|224472848|gb|ACN49136.1| RNA replicase Cucumber green mottle mosaic virus
gi|659676617|gb|AID67336.1| 129K protein Cucumber green mottle mosaic virus
gi|759021864|gb|AJP08670.1| 129K protein Cucumber green mottle mosaic virus
gi|759021894|gb|AJP08694.1| 129K protein Cucumber green mottle mosaic virus
gi|759021883|gb|AJP08685.1| 186K protein Cucumber green mottle mosaic virus
gi|759021869|gb|AJP08674.1| 129K protein Cucumber green mottle mosaic virus
gi|759021889|gb|AJP08690.1| 129K protein Cucumber green mottle mosaic virus
gi|109156754|gb|ABG26380.1| 129K protein Cucumber green mottle mosaic virus
gi|514058027|gb|AGO32734.1| 129K protein Cucumber green mottle mosaic virus
88
gi|514058032|gb|AGO32738.1| 129K protein Cucumber green mottle mosaic virus
gi|514058031|gb|AGO32737.1| 186K protein Cucumber green mottle mosaic virus
gi|256860833|gb|ACV32317.1| RNA replicase Cucumber green mottle mosaic virus
58
gi|256860834|gb|ACV32318.1| 129K protein Cucumber green mottle mosaic virus
68
gi|259013063|gb|ACV88392.1| RNA replicase Cucumber green mottle mosaic virus
59
gi|259013062|gb|ACV88391.1| RNA replicase Cucumber green mottle mosaic virus
gi|259013058|gb|ACV88388.1| RNA replicase Cucumber green mottle mosaic virus
61
gi|227015760|gb|ACP17898.1| RNA replicase Cucumber green mottle mosaic virus
gi|227015761|gb|ACP17899.1| RNA replicase Cucumber green mottle mosaic virus
gi|259013057|gb|ACV88387.1| RNA replicase Cucumber green mottle mosaic virus
66
gi|19908646|gb|AAM02961.1|AF417242 1 RNA replicase Cucumber green mottle mosaic virus
gi|224472838|gb|ACN49128.1| RNA replicase Cucumber green mottle mosaic virus
gi|254209527|gb|ACT64892.1| 129K protein Cucumber green mottle mosaic virus
40
gi|254209528|gb|ACT64893.1| 186K protein Cucumber green mottle mosaic virus
47
gi|6497082|dbj|BAA87620.1| RNA replicase Cucumber green mottle mosaic virus
74
gi|161168446|dbj|BAF93917.1| 129K replicase protein Cucumber green mottle mosaic virus
gi|523801203|gb|AGR03819.1| RNA replicase Cucumber green mottle mosaic virus
gi|523801202|gb|AGR03818.1| RNA replicase Cucumber green mottle mosaic virus
99
gi|118725120|ref|YP 908760.1| 188 kDa replicase Cucumber mottle virus
99
gi|118725121|ref|YP 908761.1| 130 kDa replicase Cucumber mottle virus
gi|22212889|ref|NP 671719.1| 126kDa protein Paprika mild mottle virus
gi|219552916|ref|YP 002455904.1| 128 kDa Cactus mild mottle virus
99
gi|219552917|ref|YP 002455905.1| replicase Cactus mild mottle virus
1E-074
gi|21389147|gb|AAM50508.1|AF406990 1 putative replicase Tobacco rattle virus
gi|62133|emb|CAA29537.1| unnamed protein product Tobacco rattle virus
gi|12034862|gb|AAG46035.1| putative replicase Tobacco rattle virus
gi|971525957|gb|ALV82089.1| replication-associated protein 2 Tobacco rattle virus
gi|20522118|ref|NP 620670.1| 134 kDa protein Tobacco rattle virus
gi|20522119|ref|NP 620669.1| 194 kDa protein Tobacco rattle virus
56
gi|12034859|gb|AAG46032.1|AF314165 1 RNA-dependent RNA polymerase Tobacco rattle virus
gi|112711|sp|P05080.2|194K TRVSY RecName: FullReplicase large subunit AltName: Full194 kDa protein AltName: FullRNA-directed RNA polymerase Contains: RecName: FullReplicase small subunit AltName: F...
gi|21389148|gb|AAM50509.1|AF406990 2 putative replicase Tobacco rattle virus
gi|8050240|gb|AAC02066.2| RNA polymerase Tobacco rattle virus
gi|971525956|gb|ALV82088.1| replication-associated protein 1 Tobacco rattle virus
gi|42733083|emb|CAE52518.1| replicase Tobacco rattle virus
gi|42733084|emb|CAE52519.1| helicase Tobacco rattle virus
55
gi|260908103|gb|ACX54058.1| 194 kDa readthrough protein Tobacco rattle virus
69
gi|260908104|gb|ACX54059.1| 134 kDa protein Tobacco rattle virus
gi|966344438|gb|ALT31701.1| replication-associated protein 2 Tobacco rattle virus
gi|966469161|gb|ALT31804.1| replication-associated protein 2 Tobacco rattle virus
gi|798777752|gb|AKA63704.1| replication-associated protein 2 Tobacco rattle virus
gi|574614154|gb|AHG52750.1| RNA-dependent RNA polymerase Tobacco rattle virus
gi|966469171|gb|ALT31809.1| replication-associated protein 2 Tobacco rattle virus
99
gi|971525941|gb|ALV82076.1| replication-associated protein 1 Tobacco rattle virus
gi|393186153|gb|AFN02871.1| 194K replication-associated protein 2 Tobacco rattle virus
gi|315361470|gb|ADU05353.1| replication-associated protein 2 Tobacco rattle virus
gi|966469160|gb|ALT31803.1| replication-associated protein 1 Tobacco rattle virus
gi|433335563|gb|AGB34172.1| replication-associated protein 2 Tobacco rattle virus
gi|798777748|gb|AKA63700.1| replication-associated protein 1 Tobacco rattle virus
gi|966344437|gb|ALT31700.1| replication-associated protein 1 Tobacco rattle virus
gi|966469170|gb|ALT31808.1| replication-associated protein 1 Tobacco rattle virus
gi|433335560|gb|AGB34169.1| replicase-associated protein 1 Tobacco rattle virus
gi|971525940|gb|ALV82075.1| replication-associated protein 2 Tobacco rattle virus
gi|393186152|gb|AFN02870.1| 134K replication-associated protein 1 Tobacco rattle virus
gi|315361469|gb|ADU05352.1| replication-associated protein 1 Tobacco rattle virus
gi|574614155|gb|AHG52751.1| putative replicase Tobacco rattle virus
0.2

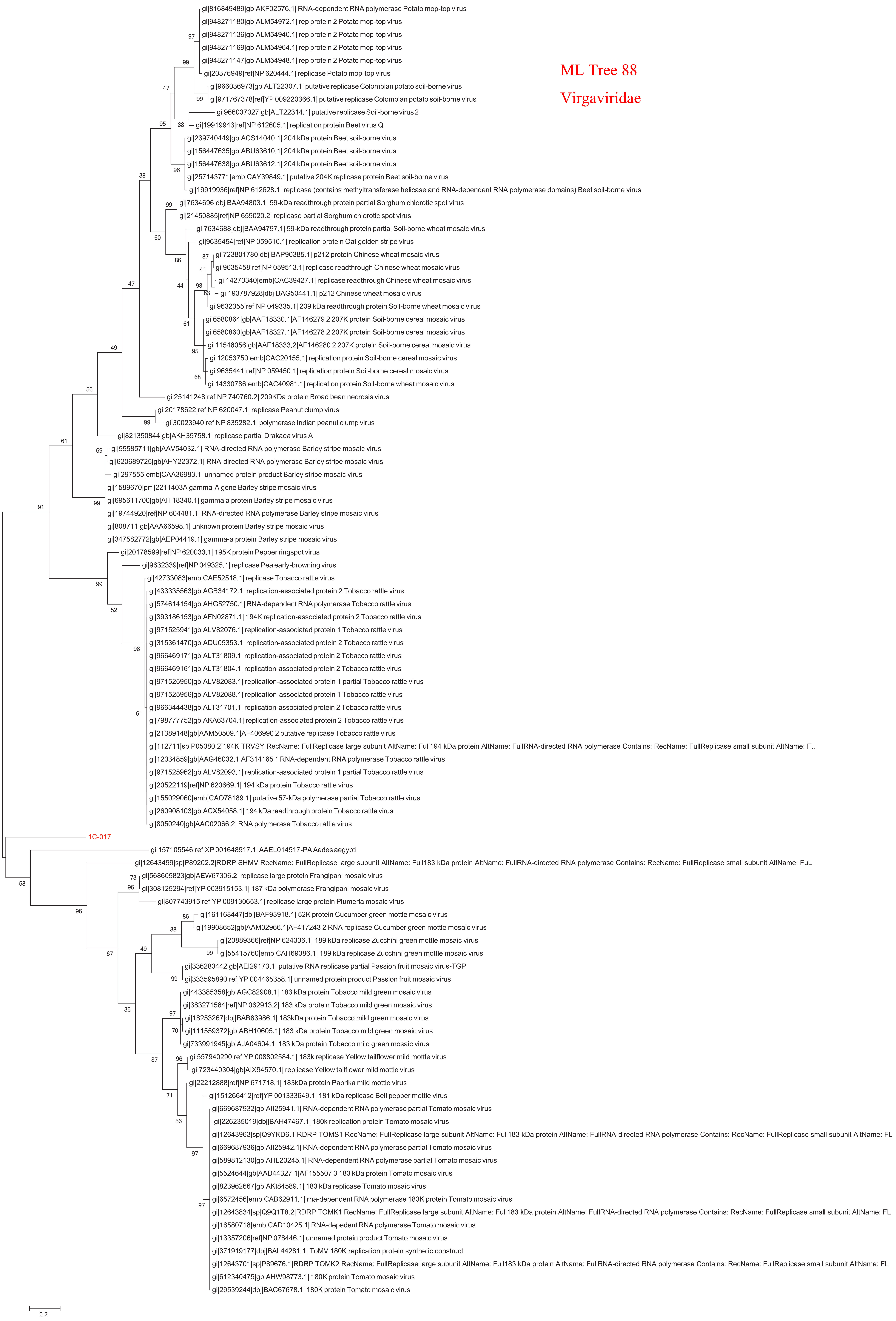
ML Tree 88
Virgaviridae
gi|816849489|gb|AKF02576.1| RNA-dependent RNA polymerase Potato mop-top virus
gi|948271180|gb|ALM54972.1| rep protein 2 Potato mop-top virus
gi|948271136|gb|ALM54940.1| rep protein 2 Potato mop-top virus
gi|948271169|gb|ALM54964.1| rep protein 2 Potato mop-top virus
gi|948271147|gb|ALM54948.1| rep protein 2 Potato mop-top virus
gi|20376949|ref|NP 620444.1| replicase Potato mop-top virus
gi|966036973|gb|ALT22307.1| putative replicase Colombian potato soil-borne virus
gi|971767378|ref|YP 009220366.1| putative replicase Colombian potato soil-borne virus
gi|966037027|gb|ALT22314.1| putative replicase Soil-borne virus 2
gi|19919943|ref|NP 612605.1| replication protein Beet virus Q
gi|239740449|gb|ACS14040.1| 204 kDa protein Beet soil-borne virus
gi|156447635|gb|ABU63610.1| 204 kDa protein Beet soil-borne virus
gi|156447638|gb|ABU63612.1| 204 kDa protein Beet soil-borne virus
gi|257143771|emb|CAY39849.1| putative 204K replicase protein Beet soil-borne virus
gi|19919936|ref|NP 612628.1| replicase (contains methyltransferase helicase and RNA-dependent RNA polymerase domains) Beet soil-borne virus
gi|7634696|dbj|BAA94803.1| 59-kDa readthrough protein partial Sorghum chlorotic spot virus
gi|21450885|ref|NP 659020.2| replicase partial Sorghum chlorotic spot virus
gi|7634688|dbj|BAA94797.1| 59-kDa readthrough protein partial Soil-borne wheat mosaic virus
gi|9635454|ref|NP 059510.1| replication protein Oat golden stripe virus
gi|723801780|dbj|BAP90385.1| p212 protein Chinese wheat mosaic virus
gi|9635458|ref|NP 059513.1| replicase readthrough Chinese wheat mosaic virus
gi|14270340|emb|CAC39427.1| replicase readthrough Chinese wheat mosaic virus
gi|193787928|dbj|BAG50441.1| p212 Chinese wheat mosaic virus
gi|9632355|ref|NP 049335.1| 209 kDa readthrough protein Soil-borne wheat mosaic virus
gi|6580864|gb|AAF18330.1|AF146279 2 207K protein Soil-borne cereal mosaic virus
gi|6580860|gb|AAF18327.1|AF146278 2 207K protein Soil-borne cereal mosaic virus
gi|11546056|gb|AAF18333.2|AF146280 2 207K protein Soil-borne cereal mosaic virus
gi|12053750|emb|CAC20155.1| replication protein Soil-borne cereal mosaic virus
gi|9635441|ref|NP 059450.1| replication protein Soil-borne cereal mosaic virus
gi|14330786|emb|CAC40981.1| replication protein Soil-borne wheat mosaic virus
gi|25141248|ref|NP 740760.2| 209KDa protein Broad bean necrosis virus
gi|20178622|ref|NP 620047.1| replicase Peanut clump virus
gi|30023940|ref|NP 835282.1| polymerase Indian peanut clump virus
gi|821350844|gb|AKH39758.1| replicase partial Drakaea virus A
gi|55585711|gb|AAV54032.1| RNA-directed RNA polymerase Barley stripe mosaic virus
gi|620689725|gb|AHY22372.1| RNA-directed RNA polymerase Barley stripe mosaic virus
gi|297555|emb|CAA36983.1| unnamed protein product Barley stripe mosaic virus
gi|1589670|prf||2211403A gamma-A gene Barley stripe mosaic virus
gi|695611700|gb|AIT18340.1| gamma a protein Barley stripe mosaic virus
gi|19744920|ref|NP 604481.1| RNA-directed RNA polymerase Barley stripe mosaic virus
gi|808711|gb|AAA66598.1| unknown protein Barley stripe mosaic virus
gi|347582772|gb|AEP04419.1| gamma-a protein Barley stripe mosaic virus
gi|20178599|ref|NP 620033.1| 195K protein Pepper ringspot virus
gi|9632339|ref|NP 049325.1| replicase Pea early-browning virus
gi|42733083|emb|CAE52518.1| replicase Tobacco rattle virus
gi|433335563|gb|AGB34172.1| replication-associated protein 2 Tobacco rattle virus
gi|574614154|gb|AHG52750.1| RNA-dependent RNA polymerase Tobacco rattle virus
gi|393186153|gb|AFN02871.1| 194K replication-associated protein 2 Tobacco rattle virus
gi|971525941|gb|ALV82076.1| replication-associated protein 1 Tobacco rattle virus
gi|315361470|gb|ADU05353.1| replication-associated protein 2 Tobacco rattle virus
gi|966469171|gb|ALT31809.1| replication-associated protein 2 Tobacco rattle virus
gi|966469161|gb|ALT31804.1| replication-associated protein 2 Tobacco rattle virus
gi|971525950|gb|ALV82083.1| replication-associated protein 1 partial Tobacco rattle virus
gi|971525956|gb|ALV82088.1| replication-associated protein 1 Tobacco rattle virus
gi|966344438|gb|ALT31701.1| replication-associated protein 2 Tobacco rattle virus
gi|798777752|gb|AKA63704.1| replication-associated protein 2 Tobacco rattle virus
gi|21389148|gb|AAM50509.1|AF406990 2 putative replicase Tobacco rattle virus
gi|112711|sp|P05080.2|194K TRVSY RecName: FullReplicase large subunit AltName: Full194 kDa protein AltName: FullRNA-directed RNA polymerase Contains: RecName: FullReplicase small subunit AltName: F...
gi|12034859|gb|AAG46032.1|AF314165 1 RNA-dependent RNA polymerase Tobacco rattle virus
gi|971525962|gb|ALV82093.1| replication-associated protein 1 partial Tobacco rattle virus
gi|20522119|ref|NP 620669.1| 194 kDa protein Tobacco rattle virus
gi|155029060|emb|CAO78189.1| putative 57-kDa polymerase partial Tobacco rattle virus
gi|260908103|gb|ACX54058.1| 194 kDa readthrough protein Tobacco rattle virus
gi|8050240|gb|AAC02066.2| RNA polymerase Tobacco rattle virus
1C-017
gi|157105546|ref|XP 001648917.1| AAEL014517-PA Aedes aegypti
gi|12643499|sp|P89202.2|RDRP SHMV RecName: FullReplicase large subunit AltName: Full183 kDa protein AltName: FullRNA-directed RNA polymerase Contains: RecName: FullReplicase small subunit AltName: FuL
gi|568605823|gb|AEW67306.2| replicase large protein Frangipani mosaic virus
gi|308125294|ref|YP 003915153.1| 187 kDa polymerase Frangipani mosaic virus
gi|807743915|ref|YP 009130653.1| replicase large protein Plumeria mosaic virus
gi|161168447|dbj|BAF93918.1| 52K protein Cucumber green mottle mosaic virus
gi|19908652|gb|AAM02966.1|AF417243 2 RNA replicase Cucumber green mottle mosaic virus
gi|20889366|ref|NP 624336.1| 189 kDa replicase Zucchini green mottle mosaic virus
gi|55415760|emb|CAH69386.1| 189 kDa replicase Zucchini green mottle mosaic virus
gi|336283442|gb|AEI29173.1| putative RNA replicase partial Passion fruit mosaic virus-TGP
gi|333595890|ref|YP 004465358.1| unnamed protein product Passion fruit mosaic virus
gi|443385358|gb|AGC82908.1| 183 kDa protein Tobacco mild green mosaic virus
gi|383271564|ref|NP 062913.2| 183 kDa protein Tobacco mild green mosaic virus
gi|18253267|dbj|BAB83986.1| 183kDa protein Tobacco mild green mosaic virus
gi|111559372|gb|ABH10605.1| 183 kDa protein Tobacco mild green mosaic virus
gi|733991945|gb|AJA04604.1| 183 kDa protein Tobacco mild green mosaic virus
gi|557940290|ref|YP 008802584.1| 183k replicase Yellow tailflower mild mottle virus
gi|723440304|gb|AIX94570.1| replicase Yellow tailflower mild mottle virus
gi|22212888|ref|NP 671718.1| 183kDa protein Paprika mild mottle virus
gi|151266412|ref|YP 001333649.1| 181 kDa replicase Bell pepper mottle virus
gi|669687932|gb|AII25941.1| RNA-dependent RNA polymerase partial Tomato mosaic virus
gi|226235019|dbj|BAH47467.1| 180k replication protein Tomato mosaic virus
gi|12643963|sp|Q9YKD6.1|RDRP TOMS1 RecName: FullReplicase large subunit AltName: Full183 kDa protein AltName: FullRNA-directed RNA polymerase Contains: RecName: FullReplicase small subunit AltName: FL
gi|669687936|gb|AII25942.1| RNA-dependent RNA polymerase partial Tomato mosaic virus
gi|589812130|gb|AHL20245.1| RNA-dependent RNA polymerase partial Tomato mosaic virus
gi|5524644|gb|AAD44327.1|AF155507 3 183 kDa protein Tomato mosaic virus
gi|823962667|gb|AKI84589.1| 183 kDa replicase Tomato mosaic virus
gi|6572456|emb|CAB62911.1| rna-dependent RNA polymerase 183K protein Tomato mosaic virus
gi|12643834|sp|Q9Q1T8.2|RDRP TOMK1 RecName: FullReplicase large subunit AltName: Full183 kDa protein AltName: FullRNA-directed RNA polymerase Contains: RecName: FullReplicase small subunit AltName: FL
gi|16580718|emb|CAD10425.1| RNA-depedent RNA polymerase Tomato mosaic virus
gi|13357206|ref|NP 078446.1| unnamed protein product Tomato mosaic virus
gi|371919177|dbj|BAL44281.1| ToMV 180K replication protein synthetic construct
gi|12643701|sp|P89676.1|RDRP TOMK2 RecName: FullReplicase large subunit AltName: Full183 kDa protein AltName: FullRNA-directed RNA polymerase Contains: RecName: FullReplicase small subunit AltName: FL
gi|612340475|gb|AHW98773.1| 180K protein Tomato mosaic virus
gi|29539244|dbj|BAC67678.1| 180K protein Tomato mosaic virus
0.2

ML Tree 89

Potyviridae

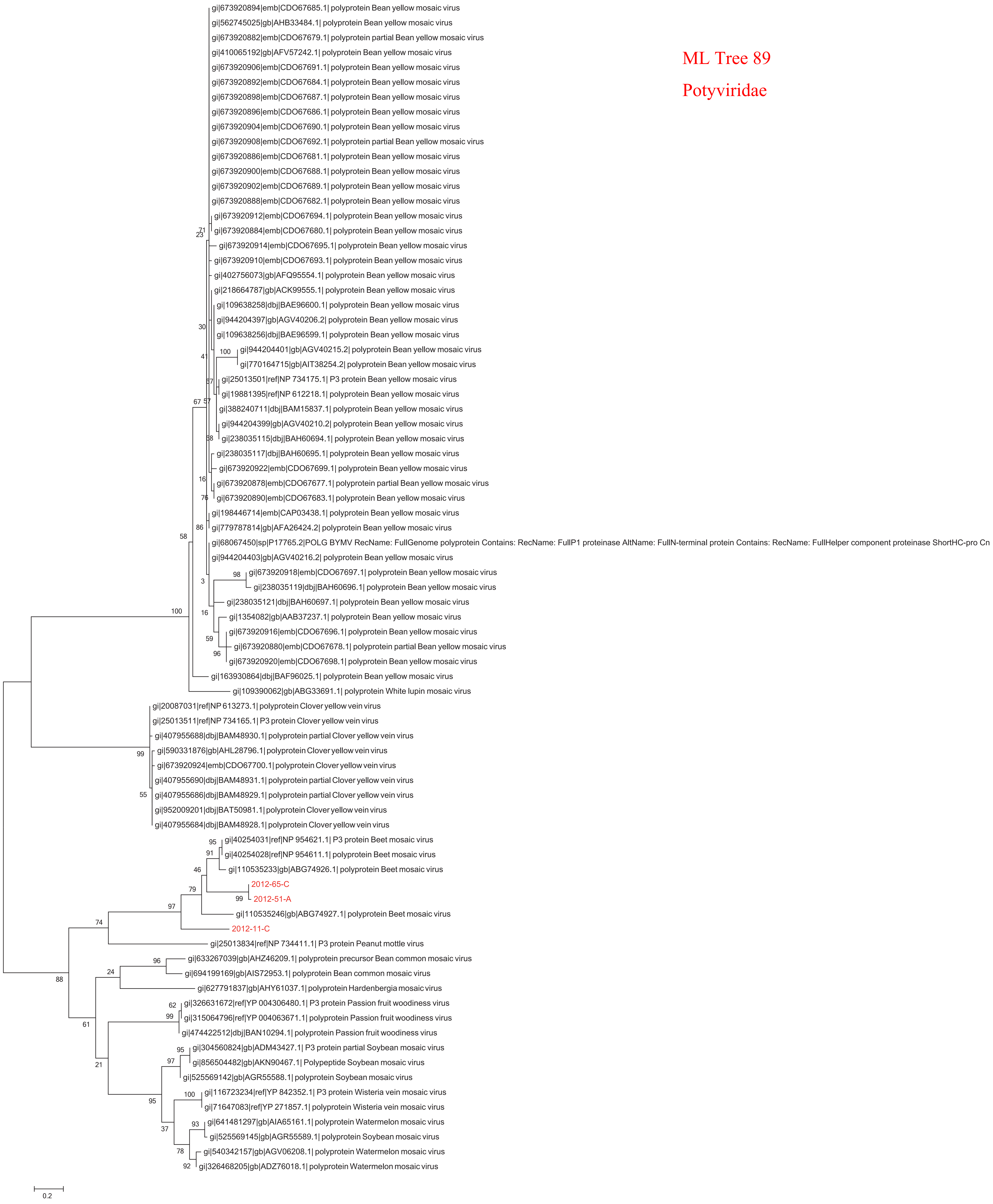

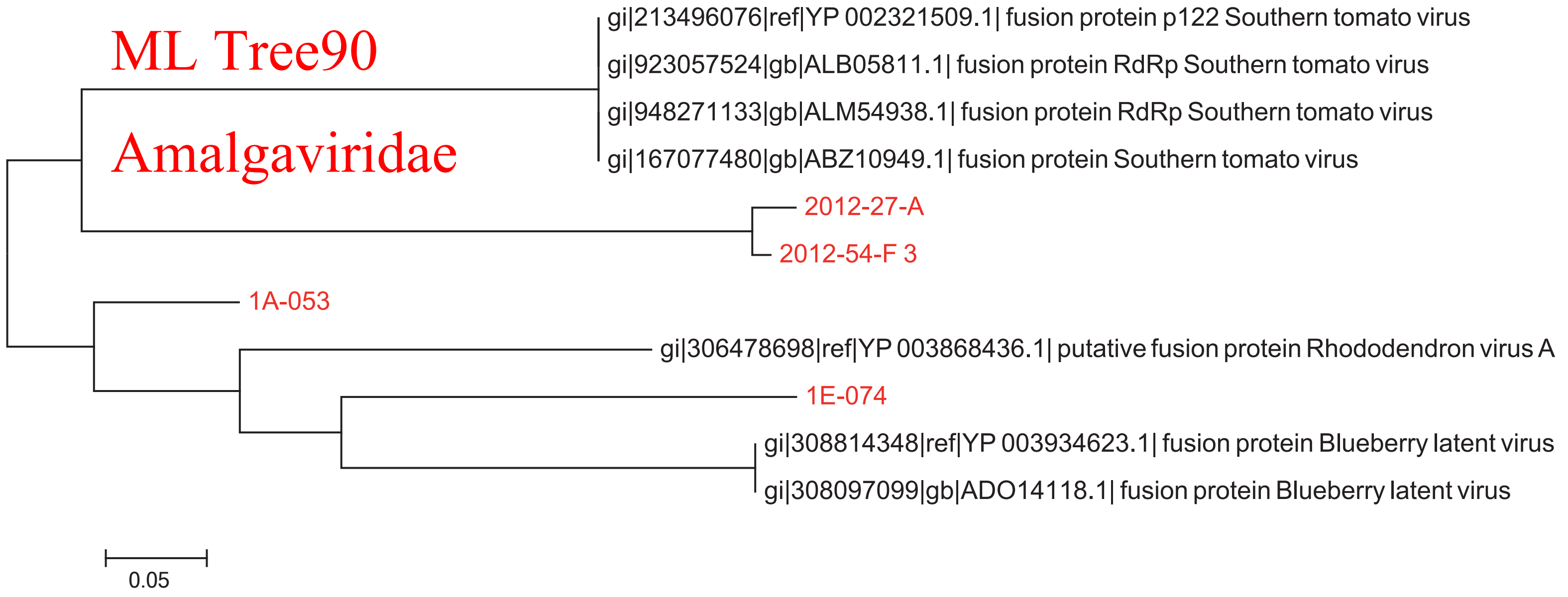
ML Tree90
Amalgaviridae
gi|213496076|ref|YP 002321509.1| fusion protein p122 Southern tomato virus
gi|923057524|gb|ALB05811.1| fusion protein RdRp Southern tomato virus
gi|948271133|gb|ALM54938.1| fusion protein RdRp Southern tomato virus
gi|167077480|gb|ABZ10949.1| fusion protein Southern tomato virus
2012-27-A
2012-54-F 3
1A-053
gi|306478698|ref|YP 003868436.1| putative fusion protein Rhododendron virus A
1E-074
gi|308814348|ref|YP 003934623.1| fusion protein Blueberry latent virus
gi|308097099|gb|ADO14118.1| fusion protein Blueberry latent virus
0.05

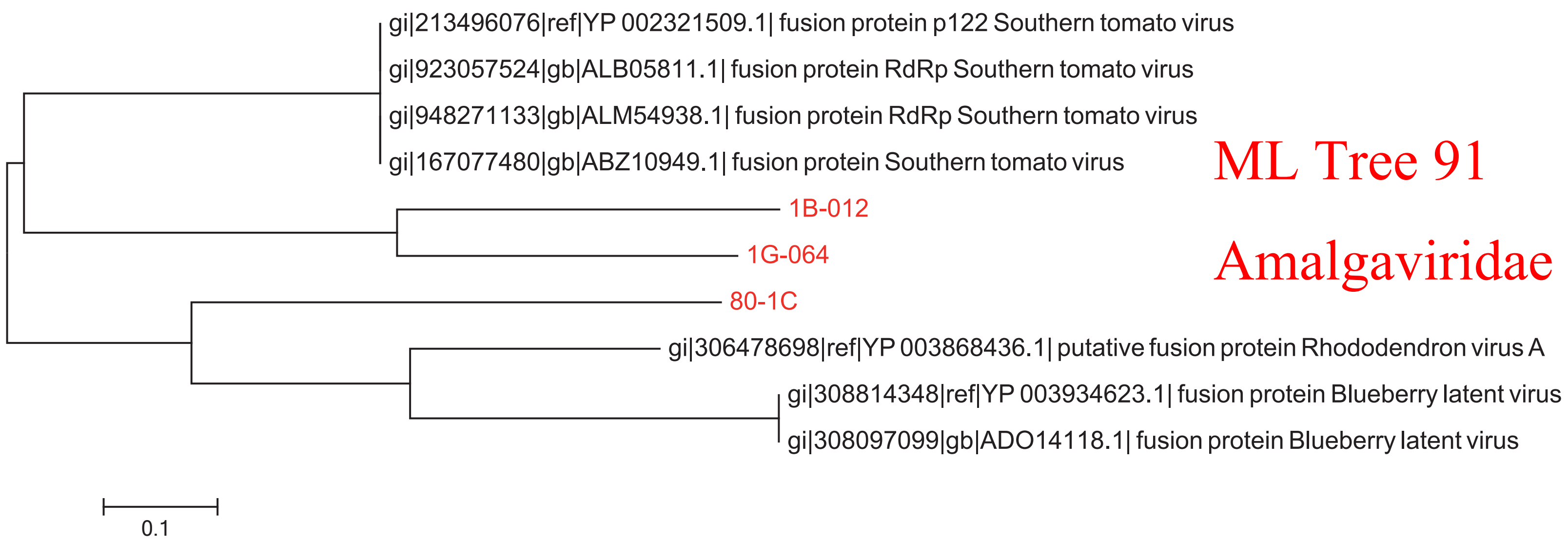
gi|213496076|ref|YP 002321509.1| fusion protein p122 Southern tomato virus
gi|923057524|gb|ALB05811.1| fusion protein RdRp Southern tomato virus
gi|948271133|gb|ALM54938.1| fusion protein RdRp Southern tomato virus
gi|167077480|gb|ABZ10949.1| fusion protein Southern tomato virus
1B-012
1G-064
80-1C
gi|306478698|ref|YP 003868436.1| putative fusion protein Rhododendron virus A
gi|308814348|ref|YP 003934623.1| fusion protein Blueberry latent virus
gi|308097099|gb|ADO14118.1| fusion protein Blueberry latent virus
0.1
ML Tree 91
Amalgaviridae

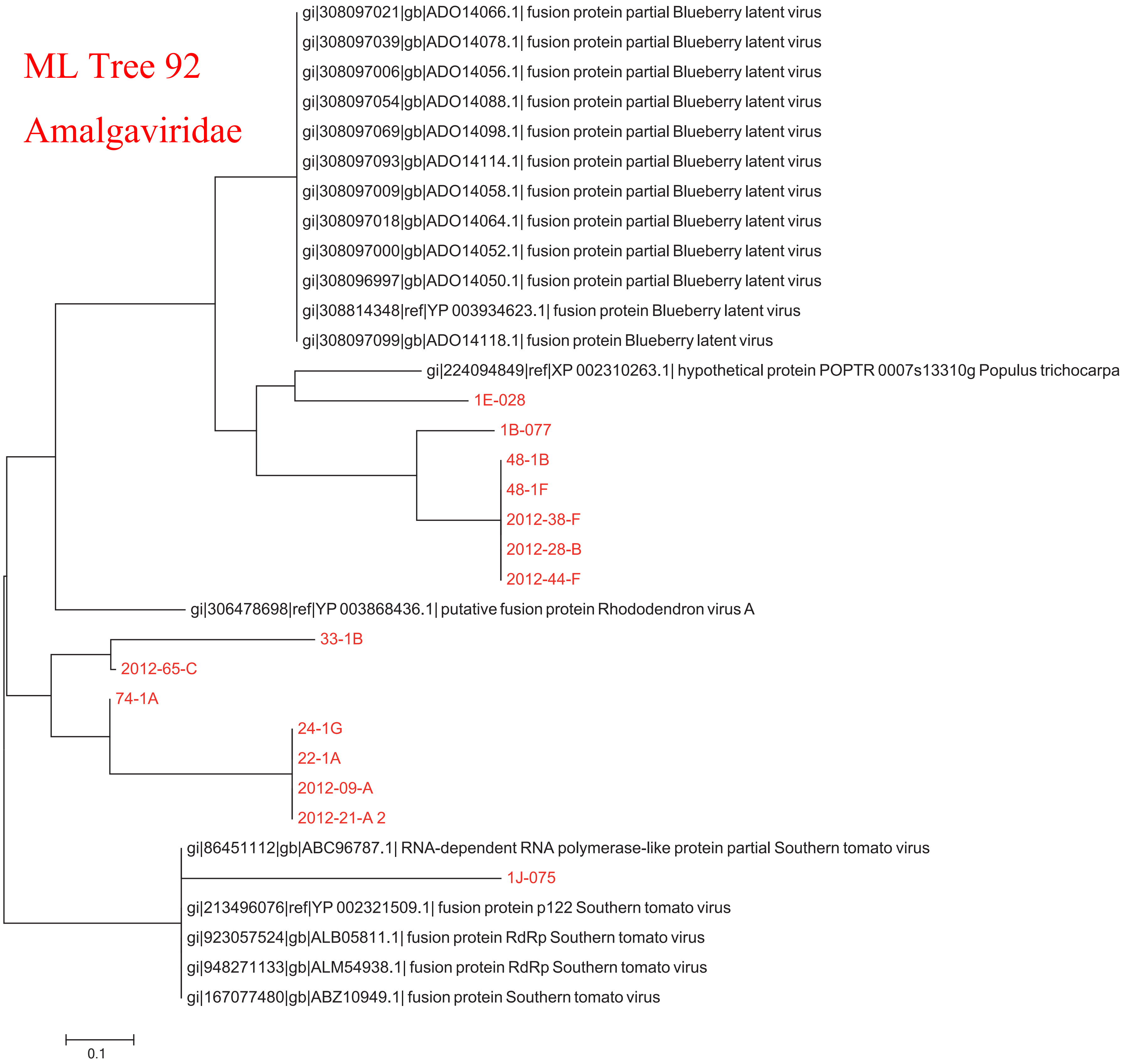
ML Tree 92
Amalgaviridae
gi|308097021|gb|ADO14066.1| fusion protein partial Blueberry latent virus
gi|308097039|gb|ADO14078.1| fusion protein partial Blueberry latent virus
gi|308097006|gb|ADO14056.1| fusion protein partial Blueberry latent virus
gi|308097054|gb|ADO14088.1| fusion protein partial Blueberry latent virus
gi|308097069|gb|ADO14098.1| fusion protein partial Blueberry latent virus
gi|308097093|gb|ADO14114.1| fusion protein partial Blueberry latent virus
gi|308097009|gb|ADO14058.1| fusion protein partial Blueberry latent virus
gi|308097018|gb|ADO14064.1| fusion protein partial Blueberry latent virus
gi|308097000|gb|ADO14052.1| fusion protein partial Blueberry latent virus
gi|308096997|gb|ADO14050.1| fusion protein partial Blueberry latent virus
gi|308814348|ref|YP 003934623.1| fusion protein Blueberry latent virus
gi|308097099|gb|ADO14118.1| fusion protein Blueberry latent virus
gi|224094849|ref|XP 002310263.1| hypothetical protein POPTR 0007s13310g Populus trichocarpa
1E-028
1B-077
48-1B
48-1F
2012-38-F
2012-28-B
2012-44-F
gi|306478698|ref|YP 003868436.1| putative fusion protein Rhododendron virus A
33-1B
2012-65-C
74-1A
24-1G
22-1A
2012-09-A
2012-21-A 2
gi|86451112|gb|ABC96787.1| RNA-dependent RNA polymerase-like protein partial Southern tomato virus
1J-075
gi|213496076|ref|YP 002321509.1| fusion protein p122 Southern tomato virus
gi|923057524|gb|ALB05811.1| fusion protein RdRp Southern tomato virus
gi|948271133|gb|ALM54938.1| fusion protein RdRp Southern tomato virus
gi|167077480|gb|ABZ10949.1| fusion protein Southern tomato virus
0.1

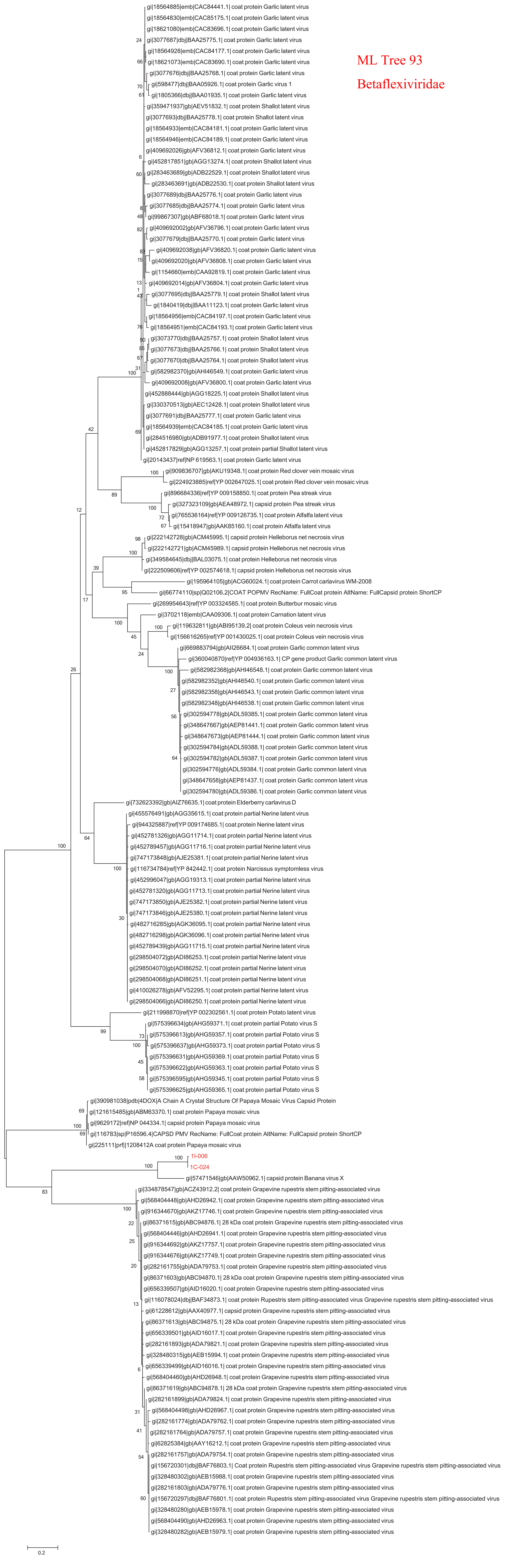
ML Tree 93
Betaflexiviridae
gi|18564885|emb|CAC84441.1| coat protein Garlic latent virus
gi|18564830|emb|CAC85175.1| coat protein Garlic latent virus
gi|18621080|emb|CAC83696.1| coat protein Garlic latent virus
gi|3077687|dbj|BAA25775.1| coat protein Garlic latent virus
gi|18564928|emb|CAC84177.1| coat protein Garlic latent virus
gi|18621073|emb|CAC83690.1| coat protein Garlic latent virus
gi|3077676|dbj|BAA25768.1| coat protein Garlic latent virus
gi|598477|dbj|BAA05926.1| coat protein Garlic virus 1
gi|1805366|dbj|BAA01935.1| coat protein Garlic latent virus
gi|359471937|gb|AEV51832.1| coat protein Shallot latent virus
gi|3077693|dbj|BAA25778.1| coat protein Shallot latent virus
gi|18564933|emb|CAC84181.1| coat protein Garlic latent virus
gi|18564946|emb|CAC84189.1| coat protein Garlic latent virus
gi|409692026|gb|AFV36812.1| coat protein Garlic latent virus
gi|452817851|gb|AGG13274.1| coat protein Shallot latent virus
gi|283463689|gb|ADB22529.1| coat protein Shallot latent virus
gi|283463691|gb|ADB22530.1| coat protein Shallot latent virus
gi|3077689|dbj|BAA25776.1| coat protein Garlic latent virus
gi|3077685|dbj|BAA25774.1| coat protein Garlic latent virus
gi|99867307|gb|ABF68018.1| coat protein Garlic latent virus
gi|409692002|gb|AFV36796.1| coat protein Garlic latent virus
gi|3077679|dbj|BAA25770.1| coat protein Garlic latent virus
gi|409692038|gb|AFV36820.1| coat protein Garlic latent virus
gi|409692020|gb|AFV36808.1| coat protein Garlic latent virus
gi|1154660|emb|CAA92819.1| coat protein Garlic latent virus
gi|409692014|gb|AFV36804.1| coat protein Garlic latent virus
gi|3077695|dbj|BAA25779.1| coat protein Shallot latent virus
gi|1840419|dbj|BAA11123.1| coat protein Garlic latent virus
gi|18564956|emb|CAC84197.1| coat protein Garlic latent virus
gi|18564951|emb|CAC84193.1| coat protein Garlic latent virus
gi|3073770|dbj|BAA25757.1| coat protein Shallot latent virus
gi|3077673|dbj|BAA25766.1| coat protein Shallot latent virus
gi|3077670|dbj|BAA25764.1| coat protein Shallot latent virus
gi|582982370|gb|AHI46549.1| coat protein Garlic latent virus
gi|409692008|gb|AFV36800.1| coat protein Garlic latent virus
gi|452888444|gb|AGG18225.1| coat protein Shallot latent virus
gi|330370513|gb|AEC12428.1| coat protein Shallot latent virus
gi|3077691|dbj|BAA25777.1| coat protein Garlic latent virus
gi|18564939|emb|CAC84185.1| coat protein Garlic latent virus
gi|284516980|gb|ADB91977.1| coat protein Shallot latent virus
gi|452817829|gb|AGG13257.1| coat protein partial Shallot latent virus
gi|20143437|ref|NP 619563.1| coat protein Garlic latent virus
gi|909836707|gb|AKU19348.1| coat protein Red clover vein mosaic virus
gi|224923885|ref|YP 002647025.1| coat protein Red clover vein mosaic virus
gi|896684336|ref|YP 009158850.1| coat protein Pea streak virus
gi|327323109|gb|AEA48972.1| capsid protein Pea streak virus
gi|765536164|ref|YP 009126735.1| coat protein Alfalfa latent virus
gi|15418947|gb|AAK85160.1| coat protein Alfalfa latent virus
gi|222142728|gb|ACM45995.1| capsid protein Helleborus net necrosis virus
gi|222142721|gb|ACM45989.1| capsid protein Helleborus net necrosis virus
gi|349584645|dbj|BAL03075.1| coat protein Helleborus net necrosis virus
gi|222509606|ref|YP 002574618.1| capsid protein Helleborus net necrosis virus
gi|195964105|gb|ACG60024.1| coat protein Carrot carlavirus WM-2008
gi|66774110|sp|Q02106.2|COAT POPMV RecName: FullCoat protein AltName: FullCapsid protein ShortCP
gi|269954643|ref|YP 003324585.1| coat protein Butterbur mosaic virus
gi|3702118|emb|CAA09306.1| coat protein Carnation latent virus
gi|119632811|gb|ABI95139.2| coat protein Coleus vein necrosis virus
gi|156616265|ref|YP 001430025.1| coat protein Coleus vein necrosis virus
gi|669883794|gb|AII26684.1| coat protein Garlic common latent virus
gi|360040870|ref|YP 004936163.1| CP gene product Garlic common latent virus
gi|582982368|gb|AHI46548.1| coat protein Garlic common latent virus
gi|582982352|gb|AHI46540.1| coat protein Garlic common latent virus
gi|582982358|gb|AHI46543.1| coat protein Garlic common latent virus
gi|582982348|gb|AHI46538.1| coat protein Garlic common latent virus
gi|302594778|gb|ADL59385.1| coat protein Garlic common latent virus
gi|348647667|gb|AEP81441.1| coat protein Garlic common latent virus
gi|348647673|gb|AEP81444.1| coat protein Garlic common latent virus
gi|302594784|gb|ADL59388.1| coat protein Garlic common latent virus
gi|302594782|gb|ADL59387.1| coat protein Garlic common latent virus
gi|302594776|gb|ADL59384.1| coat protein Garlic common latent virus
gi|348647658|gb|AEP81437.1| coat protein Garlic common latent virus
gi|302594780|gb|ADL59386.1| coat protein Garlic common latent virus
gi|732623392|gb|AIZ76635.1| coat protein Elderberry carlavirus D
gi|455576491|gb|AGG35615.1| coat protein partial Nerine latent virus
gi|944325887|ref|YP 009174685.1| coat protein Nerine latent virus
gi|452781326|gb|AGG11714.1| coat protein partial Nerine latent virus
gi|452789457|gb|AGG11716.1| coat protein partial Nerine latent virus
gi|747173848|gb|AJE25381.1| coat protein partial Nerine latent virus
gi|116734784|ref|YP 842442.1| coat protein Narcissus symptomless virus
gi|452996047|gb|AGG19313.1| coat protein partial Nerine latent virus
gi|452781320|gb|AGG11713.1| coat protein partial Nerine latent virus
gi|747173850|gb|AJE25382.1| coat protein partial Nerine latent virus
gi|747173846|gb|AJE25380.1| coat protein partial Nerine latent virus
gi|482716285|gb|AGK36095.1| coat protein partial Nerine latent virus
gi|482716298|gb|AGK36096.1| coat protein partial Nerine latent virus
gi|452789439|gb|AGG11715.1| coat protein partial Nerine latent virus
gi|298504072|gb|ADI86253.1| coat protein Nerine latent virus
gi|298504070|gb|ADI86252.1| coat protein Nerine latent virus
gi|298504068|gb|ADI86251.1| coat protein Nerine latent virus
gi|410026278|gb|AFV52295.1| coat protein Nerine latent virus
gi|298504066|gb|ADI86250.1| coat protein Nerine latent virus
gi|211998870|ref|YP 002302561.1| coat protein Potato latent virus
gi|575396634|gb|AHG59371.1| coat protein partial Potato virus S
gi|575396613|gb|AHG59357.1| coat protein partial Potato virus S
gi|575396637|gb|AHG59373.1| coat protein partial Potato virus S
gi|575396631|gb|AHG59369.1| coat protein partial Potato virus S
gi|575396622|gb|AHG59363.1| coat protein partial Potato virus S
gi|575396595|gb|AHG59345.1| coat protein partial Potato virus S
gi|575396625|gb|AHG59365.1| coat protein partial Potato virus S
gi|390981038|pdb|4DOX|A Chain A Crystal Structure Of Papaya Mosaic Virus Capsid Protein
gi|121615485|gb|ABM63370.1| coat protein Papaya mosaic virus
gi|9629172|ref|NP 044334.1| capsid protein Papaya mosaic virus
gi|116783|sp|P16596.4|CAPSD PMV RecName: FullCoat protein AltName: FullCapsid protein ShortCP
gi|225111|prf||1208412A coat protein Papaya mosaic virus
1I-006
1C-024
gi|57471546|gb|AAW50962.1| capsid protein Banana virus X
gi|334878547|gb|ACZ43912.2| coat protein Grapevine rupestris stem pitting-associated virus
gi|568404448|gb|AHD26942.1| coat protein Grapevine rupestris stem pitting-associated virus
gi|916344670|gb|AKZ17746.1| coat protein Grapevine rupestris stem pitting-associated virus
gi|86371615|gb|ABC94876.1| 28 kDa coat protein Grapevine rupestris stem pitting-associated virus
gi|568404446|gb|AHD26941.1| coat protein Grapevine rupestris stem pitting-associated virus
gi|916344692|gb|AKZ17757.1| coat protein Grapevine rupestris stem pitting-associated virus
gi|916344676|gb|AKZ17749.1| coat protein Grapevine rupestris stem pitting-associated virus
gi|282161755|gb|ADA79753.1| coat protein Grapevine rupestris stem pitting-associated virus
gi|86371603|gb|ABC94870.1| 28 kDa coat protein Grapevine rupestris stem pitting-associated virus
gi|656339507|gb|AID16020.1| coat protein Grapevine rupestris stem pitting-associated virus
gi|116078024|dbj|BAF34873.1| coat protein Rupestris stem pitting-associated virus Grapevine rupestris stem pitting-associated virus
gi|61228612|gb|AAX40977.1| capsid protein Grapevine rupestris stem pitting-associated virus
gi|86371613|gb|ABC94875.1| 28 kDa coat protein Grapevine rupestris stem pitting-associated virus
gi|656339501|gb|AID16017.1| coat protein Grapevine rupestris stem pitting-associated virus
gi|282161893|gb|ADA79821.1| coat protein Grapevine rupestris stem pitting-associated virus
gi|328480315|gb|AEB15994.1| coat protein Grapevine rupestris stem pitting-associated virus
gi|656339499|gb|AID16016.1| coat protein Grapevine rupestris stem pitting-associated virus
gi|568404460|gb|AHD26948.1| coat protein Grapevine rupestris stem pitting-associated virus
gi|86371619|gb|ABC94878.1| 28 kDa coat protein Grapevine rupestris stem pitting-associated virus
gi|282161899|gb|ADA79824.1| coat protein Grapevine rupestris stem pitting-associated virus
gi|568404498|gb|AHD26967.1| coat protein Grapevine rupestris stem pitting-associated virus
gi|282161774|gb|ADA79762.1| coat protein Grapevine rupestris stem pitting-associated virus
gi|282161764|gb|ADA79757.1| coat protein Grapevine rupestris stem pitting-associated virus
gi|62825384|gb|AAY16212.1| coat protein Grapevine rupestris stem pitting-associated virus
gi|282161757|gb|ADA79754.1| coat protein Grapevine rupestris stem pitting-associated virus
gi|156720301|dbj|BAF76803.1| Coat protein Rupestris stem pitting-associated virus Grapevine rupestris stem pitting-associated virus
gi|328480302|gb|AEB15988.1| coat protein Grapevine rupestris stem pitting-associated virus
gi|282161803|gb|ADA79776.1| coat protein Grapevine rupestris stem pitting-associated virus
gi|156720297|dbj|BAF76801.1| coat protein Rupestris stem pitting-associated virus Grapevine rupestris stem pitting-associated virus
gi|328480280|gb|AEB15978.1| coat protein Grapevine rupestris stem pitting-associated virus
gi|568404490|gb|AHD26963.1| coat protein Grapevine rupestris stem pitting-associated virus
gi|328480282|gb|AEB15979.1| coat protein Grapevine rupestris stem pitting-associated virus
0.2

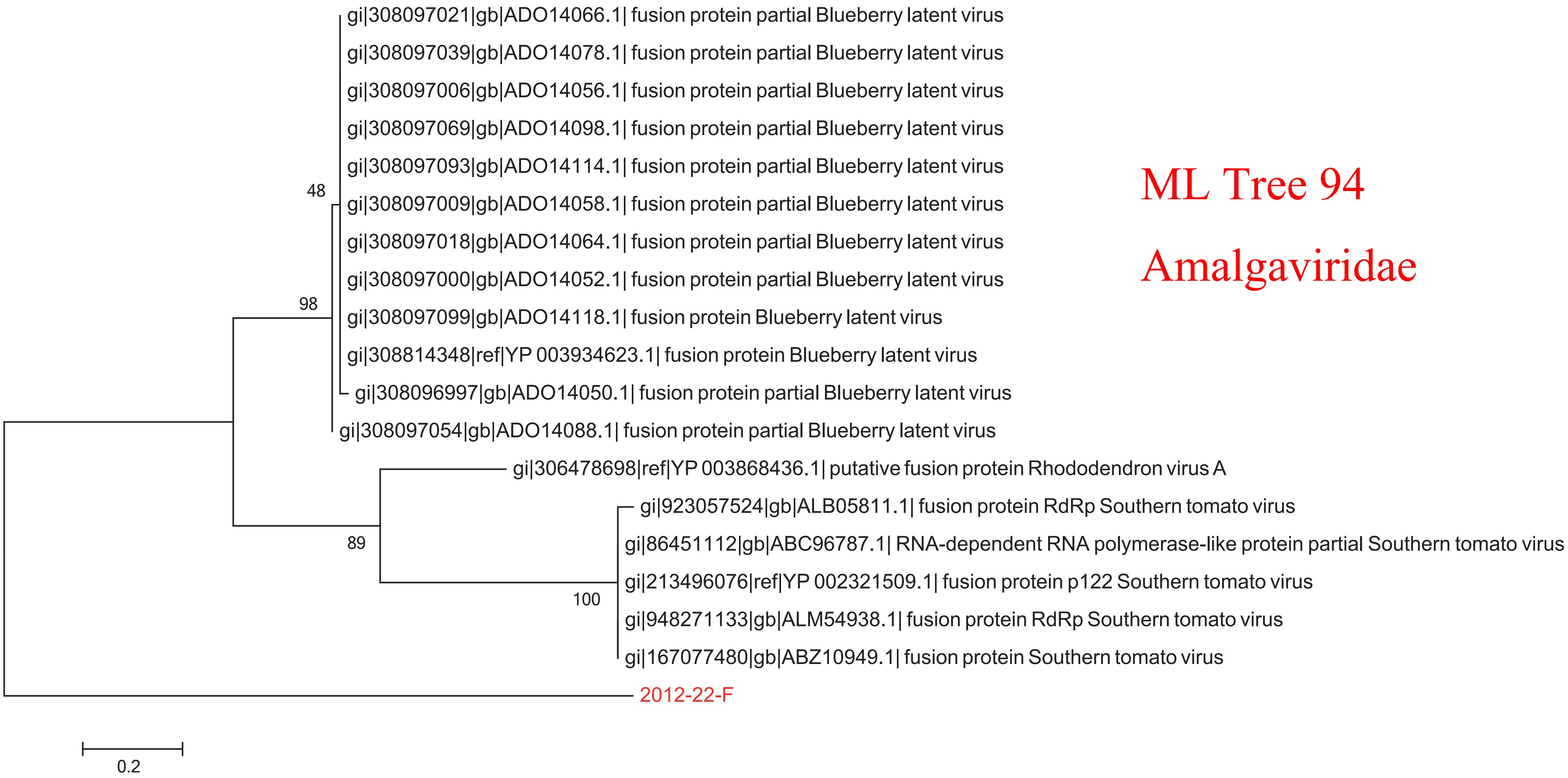

ML Tree 94
Amalgaviridae
gi|308097021|gb|ADO14066.1| fusion protein partial Blueberry latent virus
gi|308097039|gb|ADO14078.1| fusion protein partial Blueberry latent virus
gi|308097006|gb|ADO14056.1| fusion protein partial Blueberry latent virus
gi|308097069|gb|ADO14098.1| fusion protein partial Blueberry latent virus
gi|308097093|gb|ADO14114.1| fusion protein partial Blueberry latent virus
48
gi|308097009|gb|ADO14058.1| fusion protein partial Blueberry latent virus
gi|308097018|gb|ADO14064.1| fusion protein partial Blueberry latent virus
gi|308097000|gb|ADO14052.1| fusion protein partial Blueberry latent virus
98
gi|308097099|gb|ADO14118.1| fusion protein Blueberry latent virus
gi|308814348|ref|YP 003934623.1| fusion protein Blueberry latent virus
gi|308096997|gb|ADO14050.1| fusion protein partial Blueberry latent virus
gi|308097054|gb|ADO14088.1| fusion protein partial Blueberry latent virus
gi|306478698|ref|YP 003868436.1| putative fusion protein Rhododendron virus A
gi|923057524|gb|ALB05811.1| fusion protein RdRp Southern tomato virus
89
gi|86451112|gb|ABC96787.1| RNA-dependent RNA polymerase-like protein partial Southern tomato virus
gi|213496076|ref|YP 002321509.1| fusion protein p122 Southern tomato virus
100
gi|948271133|gb|ALM54938.1| fusion protein RdRp Southern tomato virus
gi|167077480|gb|ABZ10949.1| fusion protein Southern tomato virus
2012-22-F
0.2

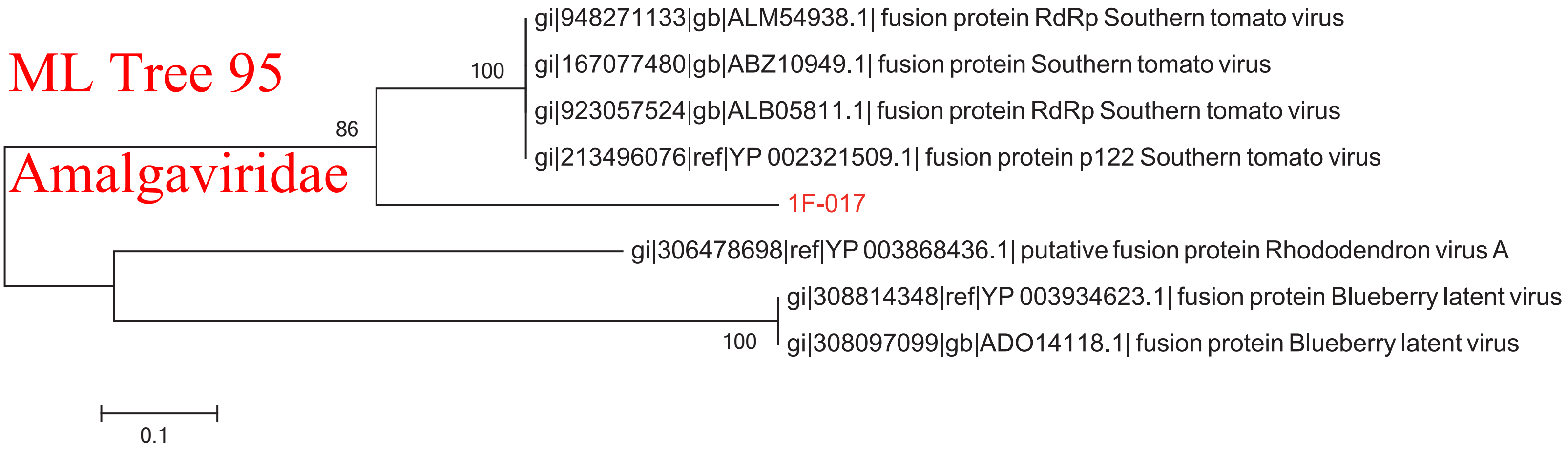
ML Tree 95
Amalgaviridae
gi|948271133|gb|ALM54938.1| fusion protein RdRp Southern tomato virus
gi|167077480|gb|ABZ10949.1| fusion protein Southern tomato virus
gi|923057524|gb|ALB05811.1| fusion protein RdRp Southern tomato virus
gi|213496076|ref|YP 002321509.1| fusion protein p122 Southern tomato virus
1F-017
gi|306478698|ref|YP 003868436.1| putative fusion protein Rhododendron virus A
gi|308814348|ref|YP 003934623.1| fusion protein Blueberry latent virus
gi|308097099|gb|ADO14118.1| fusion protein Blueberry latent virus
100
86
100
0.1
