# Supplementary material for: Geometagenomics illuminates the impact of agriculture on the distribution and prevalence of plant viruses at the ecosystem scale
Source: ISME J. 2017 Oct 20;12(1):173–84. doi: 10.1038/ismej.2017.155 (PMC5739011; doi:10.1038/ismej.2017.155)
Supplement: Supplementary Table 1 [file ismej2017155x6.docx]

**Supplementary Table S1**. Annual precipitation (mm) at the study sites. For the French site, totals for Yr_n_ are calculated as July Yr_n-1_– June Yr_n_. For the South African site, totals for totals for Yr_n_ are calculated as October Yr_n-1_– September Yr_n_. Winters in both regions are cool and wet while summers are warmer and dry, with mean annual precipitation of 530 mm (Yzerfontein, Western Cape, South Africa) – 625 mm (Arles, France). The natural growing season for most vegetation is fall–early summer.

|  | 2010 | 2012 | Mean |
| --- | --- | --- | --- |
| France (Arles) | 724 mm | 631 mm | ca. 625 mm |
| South Africa (Yzerfontein) | 932 mm | 810 mm | ca. 530 mm |
