# Supplementary material for: Geometagenomics illuminates the impact of agriculture on the distribution and prevalence of plant viruses at the ecosystem scale
Source: ISME J. 2017 Oct 20;12(1):173–84. doi: 10.1038/ismej.2017.155 (PMC5739011; doi:10.1038/ismej.2017.155)
Supplement: Supplementary Table 2 [file ismej2017155x7.docx]

**Supplementary Table S2**. Number of 454 reads (within brackets) that were obtained from sixty-two different viruses from eleven viral families with a range of genomic nucleic acid types (RNA and DNA). These viruses were in both single and co-infections available *in vivo* from the CIRAD quarantine station collection (Montpellier, France).

| **Host plant** | **Expected viruses** | **Genome** | **France2010** | **France2012** | **South-Africa2010** |
| --- | --- | --- | --- | --- | --- |
| Arabidopsis thaliana | CaMV (Caulimovirus) | Monopartite open circular dsDNA | + (1693) |  | + (29) |
| Arabidopsis thaliana | TUMV (Potyvirus) | Monopartite linear ssRNA(+) | + (43) |  | + (10) |
|  | CaMV (Caulimovirus) | Monopartite open circular dsDNA | + (192) |  | + (1) |
| *Brassica rapa* | TUMV (Potyvirus) | Monopartite linear ssRNA(+) |  | + (1810) |  |
| Datura stramonium | ToLCKMV (Begomovirus) | Monopartite circular ssDNA | + (343) |  | + (17) |
| Datura stramonium | TYLCV (Begomovirus) | Monopartite circular ssDNA | + (367) |  | + (6) |
| *Dioscorea alata* | YMV (Potyvirus) | Monopartite linear ssRNA(+) | + (49) | + (7) | - (not detected) |
| *Dioscorea alata* | YVX (Potexvirus) | Monopartite linear ssRNA(+) |  | + (1744) | + (1) |
| *Euphorbia caput-medusae* | EcmLV (Capulavirus) | Monopartite circular ssDNA | + (1) | - (not detected) |  |
| Musa acuminata | BSOLV (Badnavirus) | Monopartite open circular dsDNA | + (15) | - (not detected) | + (1) |
| *Nicotiana benthamiana* | PPV (Potyvirus) | Monopartite linear ssRNA(+) |  | + (224) |  |
| *Nicotiana benthamiana* | TUMV (Potyvirus) | Monopartite linear ssRNA(+) | + (50) |  | - (not detected) |
| *Nicotiana benthamiana* | TUMV (Potyvirus) | Monopartite linear ssRNA(+) | - (not detected) |  | - (not detected) |
|  | TYLCV (Begomovirus) | Monopartite circular ssDNA | + (1648) |  | + (1) |
| *Saccharum officinarum* | SCYLV (Polerovirus) | Monopartite linear ssRNA(+) |  | + (2) | + (1) |
| *Saccharum officinarum* | SCBV (Badnavirus) | Monopartite open circular dsDNA |  | + (25) |  |
| *Saccharum officinarum* | PCV (Pecluvirus) | Segmented linear ssRNA(+) |  | + (16) |  |
| *Saccharum officinarum* | SCBV (Badnavirus) | Monopartite open circular dsDNA | + (856) | + (184) | + (5) |
|  | SCSMV (Poacevirus) | Monopartite linear ssRNA(+) | - (not detected) | + (311) | + (39) |
| *Saccharum officinarum* | SCBV (Badnavirus) | Monopartite open circular dsDNA | + (222) |  |  |
|  | SCSMV (Poacevirus) | Monopartite linear ssRNA(+) | + (4) |  |  |
| *Saccharum officinarum* | SCBV (Badnavirus) | Monopartite open circular dsDNA | + (13) |  |  |
|  | SCYLV (Polerovirus) | Monopartite linear ssRNA(+) | - (not detected) |  |  |
| *Saccharum officinarum* | SCBV (Badnavirus) | Monopartite open circular dsDNA | + (16) |  |  |
|  | SRMV (Potyvirus) | Monopartite linear ssRNA(+) | - (not detected) |  |  |
| *Saccharum officinarum* | SCBV (Badnavirus) | Monopartite open circular dsDNA | + (25) | + (1) | - (not detected) |
|  | SWSV (Mastrevirus) | Monopartite circular ssDNA | + (1111) | - (not detected) | + (145) |
|  | SSEV (Mastrevirus) | Monopartite circular ssDNA | + (292) | + (1810) | + (21) |
| *Saccharum officinarum* | SCBV (Badnavirus) | Monopartite open circular dsDNA |  | + (195) | + (55) |
|  | SCMV (Potyvirus) | Monopartite linear ssRNA(+) |  | + (2) | + (2) |
|  | SCYLV (Polerovirus) | Monopartite linear ssRNA(+) |  | + (14) | + (14) |
| *Saccharum officinarum* | SRMV (Potyvirus) | Monopartite linear ssRNA(+) |  |  | + (115) |
|  | SCYLV (Polerovirus) | Monopartite linear ssRNA(+) |  |  | + (14) |
| *Saccharum officinarum* | SRMV (Potyvirus) | Monopartite linear ssRNA(+) |  | + (6) | + (115) |
|  | SCYLV (Polerovirus) | Monopartite linear ssRNA(+) |  | + (79) | + (14) |
|  | SCBV (Badnavirus) | Monopartite open circular dsDNA |  | + (45) |  |
|  | Novel Ampelovirus | Monopartite linear ssRNA(+) |  | + (2) |  |
| *Solanum lycopersicum* | TYLCV (Begomovirus) | Monopartite circular ssDNA |  | + (376) |  |
| *Vicia faba* | FBNSV (Nanovirus) | Multipartite circular ssDNA |  | + (685) |  |
|  | VCV (Alphapartitivirus) | Segmented linear dsRNA |  | + (787) |  |
