# Supplementary material for: Geometagenomics illuminates the impact of agriculture on the distribution and prevalence of plant viruses at the ecosystem scale
Source: ISME J. 2017 Oct 20;12(1):173–84. doi: 10.1038/ismej.2017.155 (PMC5739011; doi:10.1038/ismej.2017.155)
Supplement: Supplementary Table 3 [file ismej2017155x8.docx]

**Supplementary Table S3**: Seven hundred and fifty-five 454 pyrosequencing single reads and contigs with detectable similarity to plant-associated viruses found from 511 bulked plant samples from France (2010 and 2012) and South Africa (2010). Uncultivated plants that contained OTU closely related to known crop pathogens are indicated in bold.

**Novel plant viruses (94 OTUs, 121 plants)**

| **OTU** | **Family** | **Genus** | **Coding region-related sequence** | **Host plant** | **Plant Family** | **Location** | **Sample number** | **Identity** | **Phylogenetic**  **tree number*** |
| --- | --- | --- | --- | --- | --- | --- | --- | --- | --- |
| 1 | Alphaflexiviridae | Potexvirus | Triple gene block protein | *Eriocephalus africanus* | Asteraceae | SA2010 | 1B-023 | 37.93 | 1 |
| 1 |  |  | Triple gene block protein | *Not identified* |  | SA2010 | 1J-021 | 38.46 | 1 |
| 2 | Alphaflexiviridae | Potexvirus | RNA dependent RNA polymerase | *Carex cuprina* | Cyperaceae | FR2012 | 2012-75-D | 52.94 | 2 |
| 3 | Alphaflexiviridae | Potexvirus | Replicase protein | *Lebeckia sepiaria* | Fabaceae | SA2010 | 1F-073 | 61.22 | 3 |
| 4 | Alphaflexiviridae | Potexvirus | Replication-associated polyprotein | *Avena byzantina* | Poaceae | SA2010 | 1B-007 | 39.34 | 4 |
| 5 | Alphaflexiviridae | Unclassified | Putative RNA replicase | *Tetragonia sp.* | Aizoaceae | SA2010 | 1F-010 | 47.44 | 4 |
| 6 | Alphaflexiviridae | Unclassified | Replication-associated polyprotein | *Salvia sp.* | Lamiaceae | SA2010 | 1G-092 | 72.88 | 5 |
| 7 | Amalgaviridae | Amalgavirus | Putative fusion protein | *Lotus glaber* | Fabaceae | FR2010 | 44-1F | 54.19 | 7-9, 13-14 |
| 7 |  |  | Putative fusion protein | *Lotus corniculatus* | Fabaceae | FR2010 | 33-1B | 52.97 | 7-9, 13-14 |
| 8 | Amalgaviridae | Amalgavirus | Fusion protein | *Melilotus alba* | Fabaceae | FR2010 | 48-1F | 55.56 | 7-9, 11-14 |
| 8 |  |  | RNA-dependent RNA polymerase | *Medicago sativa* | Fabaceae | FR2010 | 48-1B | 56.04 | 7 |
| 8 |  |  | RNA-dependent RNA polymerase | *Medicago sativa* | Fabaceae | FR2012 | 2012-48-E | 62.86 | 8, 9, 14 |
| 8 |  |  | Fusion protein | *Trifolium angustifolium* | Fabaceae | FR2012 | 2012-28-B | 53.69 | 7-15 |
| 8 |  |  | Putative fusion protein | *Medicago sativa* | Fabaceae | FR2012 | 2012-27-A | 41.27 | 8-12, 14, 15 |
| 8 |  |  | RNA-dependent RNA polymerase | *Medicago sativa* | Fabaceae | FR2012 | 2012-44-F | 54.19 | 7-9, 14 |
| 8 |  |  | RNA-dependent RNA polymerase | *Medicago sativa* | Fabaceae | FR2012 | 2012-48-C | 51.39 | 8, 9, 14 |
| 8 |  |  | RNA-dependent RNA polymerase | *Medicago sativa* | Fabaceae | FR2012 | 2012-54-F | 59.06 | 10-12, 15 |
| 8 |  |  | Fusion protein | *Trifolium resupinatum* | Fabaceae | FR2012 | 2012-32-J | 60.61 | 9, 11, 12 |
| 8 |  |  | RNA-dependent RNA polymerase | *Oxalis sp.* | Oxalidaceae | FR2012 | 2012-38-F | 51.87 | 9, 13, 14 |
| 9 | Amalgaviridae | Amalgavirus | Putative fusion protein | *Schedonorus arundinaceus* | Poaceae | FR2010 | 24-1G | 47.17 | 7, 13 |
| 9 |  |  | Putative fusion protein | *Puccinellia sp.* | Poaceae | FR2010 | 72-1A | 49.57 | 7, 8, 13 |
| 9 |  |  | Putative fusion protein | *Schedonorus arundinaceus* | Poaceae | FR2010 | 22-1A | 52.12 | 7-9, 13, 14 |
| 9 |  |  | Putative fusion protein | *Puccinellia festuciformis* | Poaceae | FR2010 | 74-1A | 29.44 | 7-9, 11-14 |
| 9 |  |  | RNA-dependent RNA polymerase | *Puccinellia festuciformis* | Poaceae | FR2010 | 41-1E | 57.14 | 12, 15 |
| 9 |  |  | Putative fusion protein | *Puccinellia festuciformis* | Poaceae | FR2010 | 53-1D | 32.29 | 6 |
| 9 |  |  | Fusion protein | *Avena sp.* | Poaceae | FR2012 | 2012-09-A | 47.67 | 7, 13 |
| 9 |  |  | Putative fusion protein | *Schedonorus arundinaceus* | Poaceae | FR2012 | 2012-89-B | 51.47 | 7, 8, 13 |
| 9 |  |  | Putative fusion protein | *Sorghum bicolor* | Poaceae | FR2012 | 2012-76-D | 47.39 | 8, 9, 11, 12, 14 |
| 9 |  |  | Fusion protein | *Schedonorus arundinaceus* | Poaceae | FR2012 | 2012-21-A | 60.45 | 7-9, 11, 12, 14 |
| 9 |  |  | Putative fusion protein | *Schedonorus arundinaceus* | Poaceae | FR2012 | 2012-44-E | 60.00 | 7, 10-12 |
| 9 |  |  | Putative fusion protein | *Echinochloa sp.* | Poaceae | FR2012 | 2012-49-A | 60.87 | 11, 12 |
| 9 |  |  | Fusion protein | *Schedonorus arundinaceus* | Poaceae | FR2012 | 2012-33-E | 56.10 | 10, 15 |
| 9 |  |  | Putative fusion protein | *Staberoha distachyos* | Restionaceae | SA2010 | 1G-001 | 51.82 | 8-10, 14 |
| 9 |  |  | Fusion protein | *Staberoha distachyos* | Restionaceae | SA2010 | 1D-013 | 59.78 | 10,15 |
| 9 |  |  | Putative fusion protein | *Thamnochortus spicigerus* | Restionaceae | SA2010 | 1G-063 | 70.23 | 14 |
| 9 |  |  | Fusion protein | *Not identified* |  | SA2010 | 1I-083 | 31.40 | 6, 13 |
| 9 |  |  | Putative fusion protein | *Not identified* |  | SA2010 | 1G-039 | 29.38 | 7, 8, 12-14 |
| 10 | Amalgaviridae | Amalgavirus | RNA-dependent RNA polymerase | *Asparagus maritimus* | Asparagaceae | FR2012 | 2012-41-E | 67.54 | 11, 12, 15 |
| 10 |  |  | Fusion protein | *Schedonorus arundinaceus* | Poaceae | FR2012 | 2012-36-A | 54.17 | 9, 11, 12 |
| 11  11 | Amalgaviridae | Amalgavirus | Fusion protein | *Not identified* | Cucurbitaceae | SA2010 | 1B-077 | 56.52 | 8, 9, 11, 12, 14, 15 |
| 12 | Amalgaviridae | Amalgavirus | Putative fusion protein | *Galenia africana* | Aizoaceae | SA2010 | 1D-012 | 62.63 | 11, 12, 14, 15 |
| 12 |  |  | Fusion protein | *Ammocharis longifolia* | Alliaceae | SA2010 | 1E-028 | 60.47 | 7, 13, 14 |
| 13 | Amalgaviridae | Amalgavirus | Putative fusion protein | *Ehrharta calycina* | Poaceae | SA2010 | 1J-075 | 49.49 | 7, 10-12, 15 |
| 14 | Amalgaviridae | Amalgavirus | Putative fusion protein | *Not identified* |  | SA2010 | 1G-064 | 57.58 | 15 |
| 15 | Benyviridae | Benyvirus | Replication-associated protein | *Lotus tenuis* | Fabaceae | FR2010 | 34-1A | 27.19 | 16 |
| 16 | Betaflexiviridae | Capillovirus | Polyprotein | *Vicia cracca* | Fabaceae | FR2010 | 30-1G | 66.29 | 17 |
| 17 | Betaflexiviridae | Carlavirus | RNA-dependent RNA polymerase | *Trifolium repens* | Fabaceae | FR2010 | 20-1E | 61.34 | 18 |
| 18 | Betaflexiviridae | Carlavirus | Triple gene block protein | *Lotus corniculatus* | Fabaceae | FR2010 | 47-1A | 60.53 | 18 |
| 18 |  |  | Triple gene block protein | *Lotus glaber* | Fabaceae | FR2010 | 34-1A | 60.53 | 19 |
| 19 | Flexiviridae | Unclassified | RNA-dependent RNA polymerase | *Euphorbia mauritanica* | Euphorbiaceae | SA2010 | 1G-031 | 71.43 | 20 |
| 20 | Bromoviridae | Anulavirus | Putative 2a protein | *Suaeda vera* | Chenopodiaceae | FR2012 | 2012-03-D | 63.46 | 21 |
| 21 | Bromoviridae | Ilarvirus | Replicase | *Avena byzantina* | Poaceae | SA2010 | 1B-007 | 45.10 | 23 |
| 22 | Caulimoviridae | Badnavirus | Polyprotein | *Sorghum sp.* | Poaceae | FR2010 | 25-1A | 38.28 | 24 |
| 23 | Caulimoviridae | Badnavirus | Poplyprotein | *Not identified* |  | SA2010 | 1E-032 | 52.50 | 25 |
| 24 | Caulimoviridae | Cavemovirus | Putative coat protein | *Exomis sp.* | Amaranthaceae | SA2010 | 1B-078 | 38.96 | 26 |
| 25 | Caulimoviridae | Petuvirus | Polyprotein 1 | *Lebeckia sepiaria* | Fabaceae | SA2010 | 1G-002 | 60.00 | 27 |
| 26 | Caulimoviridae | Solendovirus | Putative inclusion body protein | *Halimione portulacoides* | Chenopodiaceae | FR2012 | 2012-51-D | 52.63 | 28 |
| 26 |  |  | Putative inclusion body protein | *Halimione portulacoides* | Chenopodiaceae | FR2012 | 2012-11-C | 41.60 | 28, 29 |
| 26 |  |  | Putative inclusion body protein | *Puccinellia festuciformis* | Poaceae | FR2010 | 04-1B | 47.00 | 29 |
| 27 | Closteroviridae | Ampelovirus | RNA polymerase | *Lebeckia sepiaria* | Fabaceae | SA2010 | 1F-073 | 59.65 | 30 |
| 28 | Closteroviridae | Ampelovirus | Methyl transferase/helicase | *Trifolium pratense* | Fabaceae | FR2012 | 2012-31-L | 33.33 | 31 |
| 29 | Closteroviridae | Closterovirus | Coat protein | *Verbena officinalis* | Verbenaceae | FR2012 | 2012-58-G | 55.36 | 32 |
| 30 | Closteroviridae | Closterovirus | Coat protein | *Lotus glaber* | Fabaceae | FR2010 | 34-1A | 35.95 | 32 |
| 31 | Closteroviridae | Closterovirus | Major coat protein | *Trifolium repens* | Fabaceae | FR2010 | 47-1C | 34.78 | 32 |
| 32 | Closteroviridae | Crinivirus | RNA-dependent RNA polymerase | *Trifolium resupinatum* | Fabaceae | FR2012 | 2012-32-J | 34.34 | 33 |
| 33 | Endornaviridae | Endornavirus | Polyprotein] | *Ranunculus peltatus* | Ranunculaceae | FR2010 | 82-1A | 38.67 | 34, 38 |
| 34 | Endornaviridae | Endornavirus | ORFA+B | *Trifolium repens* | Fabaceae | FR2010 | 43-1D | 35.29 | - |
| 35 | Endornaviridae | Endornavirus | Polyprotein | *Atriplex halimus* | Chenopodiaceae | FR2010 | 73-1D | 25.39 | 34, 38 |
| 35 |  |  | Polyprotein | *Halimione portulacoides* | Chenopodiaceae | FR2012 | 2012-92-B | 36.71 | 35 |
| 35 |  |  | Polyprotein | *Halimione portulacoides* | Chenopodiaceae | FR2012 | 2012-03-A | 29.92 | 35 |
| 35 |  |  | Polyprotein | *Halimione portulacoides* | Chenopodiaceae | FR2012 | 2012-02-C | 28.68 | 34-36, 38 |
| 35 |  |  | Polyprotein | *Halimione portulacoides* | Chenopodiaceae | FR2012 | 2012-13-B | 33.60 | 36 |
| 36 | Endornaviridae | Endornavirus | Polyprotein | *Microcodon sp.* | Campanulaceae | SA2010 | 1F-011 | 27.49 | 37 |
| 37 | Endornaviridae | Endornavirus | Polyprotein | *Aspalathus sp.* | Fabaceae | SA2010 | 1G-015 | 49.12 | 39 |
| 38 | Endornaviridae | Endornavirus | Polyprotein | *Euphorbia caput-medusae* | Euphorbiaceae | SA2010 | 1A-014 | 50.54 | 40 |
| 39 | Endornaviridae | Endornavirus | Polyprotein | *Not identified* |  | SA2010 | 1G-064 | 65.79 | 41 |
| 40 | Geminiviridae | Begomovirus | Replication associated protein | *Juncus maritimus* | Juncaceae | FR2012 | 2012-13-F | 40.00 | 42-45 |
| 41 | Geminiviridae | Begomovirus | Replication associated protein | *Not identified* |  | SA2010 | 1A-070 | 33.68 | 43 |
| 42 | Geminiviridae | Becurtovirus | Replication associated protein | *Exomis sp.* | Amaranthaceae | SA2010 | 1H-090 | 70.30 | 42, 43, 45 |
| 43 | Geminiviridae | Mastrevirus | Coat protein | *Asparagus rubicundus* | Asparagaceae | SA2010 | 1E-080 | 52.88 | 42, 43, 45 |
| 44 | Geminiviridae | Unclassified | C2 protein | *Plantago coronopus* | Plantaginaceae | FR2012 | 2012-23-J | 53.19 | 44 |
| 45 | Geminiviridae | Unclassified | Replication associated protein | *Medicago sativa* | Fabaceae | FR2010 | 44-1E | 76.36 | 42, 43, 45 |
| 45 |  |  | Replication associated protein | *Medicago sativa* | Fabaceae | FR2012 | 2012-48-A | 74.77 | 42, 43, 45 |
| 46 | Geminiviridae | Unclassified | Coat protein | *Euphorbia caput-medusae* | Euphorbiaceae | SA2010 | 1A-014 | 100.00 | 42, 43, 45 |
| 47 | Luteoviridae | Polerovirus | Multifunctional protein | *Echinochloa sp.* | Poaceae | FR2012 | 2012-60-B | 45.83 | 52 |
| 48 | Luteoviridae | Polerovirus | Putative protein P2 | *Not identified* | Poaceae | FR2012 | 2012-56-F | 65.88 | 51 |
| 49 | Nanoviridae | Unclassified | Replication-associated protein | *Trifolium repens* | Fabaceae | FR2010 | 20-1E | 49.31 | 53, 54 |
| 50 | Nanoviridae | Unclassified | Coat protein | *Vicia cracca* | Fabaceae | FR2010 | 30-1G | 68.02 | 53, 54 |
| 51 | Nanoviridae | Unclassified | Replication initiation protein | *Medicago sativa* | Fabaceae | FR2012 | 2012-27-A | 48.96 | 54 |
| 52 | Potyviridae | Potyvirus | Polyprotein | *Trifolium pratense* | Fabaceae | FR2012 | 2012-31-L | 72.52 | 55 |
| 52 |  |  | P3 protein | *Medicago lupulina* | Fabaceae | FR2012 | 2012-100-G | 42.61 | 55 |
| 53 | Potyviridae | Potyvirus | Polyprotein | *Dipogon lignosus* | Fabaceae | SA2010 | 1I-031 | 75.84 | 56 |
| 53 |  |  | Polyprotein | *Dipogon lignosus* | Fabaceae | SA2010 | 1E-023 | 75.41 | 56 |
| 53 |  |  | Polyprotein | *Dipogon lignosus* | Fabaceae | SA2010 | 1D-024 | 74.04 | 56 |
| 54 | Potyviridae | Macluravirus | Polyprotein | *Cirsium arvense* | Asteraceae | FR2012 | 2012-58-C | 70.27 | 57 |
| 55 | Potyviridae | Tritimovirus | Polyprotein | *Schedonorus arundinaceus* | Poaceae | FR2010 | 75-1C | 61.54 | 59 |
| 56 | Potyviridae | Tritimovirus | Polyprotein | *Brachypodium phoenicoides* | Poaceae | FR2012 | 2012-75-F | 39.80 | 58 |
| 57 | Reoviridae | Oryzavirus | P9 structural protein | *Calendula sp.* | Asteraceae | SA2010 | 1C-081 | 47.22 | 60 |
| 58 | Rhabdoviridae | Cytorhabdovirus | Putative polymerase | *Lamprocephalus sp.* | Asteraceae | SA2010 | 1D-040 | 73.64 | 61 |
| 59 | Secoviridae | Waikavirus | Polyprotein | *Trifolium repens* | Fabaceae | FR2010 | 44-1D | 25.35 | 62 |
| 59 |  |  | Polyprotein | *Not identified* |  | FR2010 | 31-1E | 47.19 | 62 |
| 60 | Secoviridae | Waikavirus | Polyprotein | *Lotus glaber* | Fabaceae | FR2010 | 34-1A | 37.93 | 62 |
| 61 | Secoviridae | Waikavirus | 3C-like protease | *Lotus corniculatus* | Fabaceae | FR2010 | 47-1A | 60.38 | 63 |
| 62 | Secoviridae | Nepovirus | Movement protein | *Salicornia fruticosa* | Amaranthaceae | FR2012 | 2012-14-D | 53.27 | 65 |
| 62 |  |  | Polyprotein segment 1 | *Arthrocnemum macrostachyum* | Amaranthaceae | FR2012 | 2012-06-C | 76.52 | 65, 68 |
| 62 |  |  | Polyprotein segment 1 | *Salicornia fruticosa* | Amaranthaceae | FR2012 | 2012-04-C | 53.23 | 64 |
| 62 |  |  | Polyprotein segment 1 | *Arthrocnemum macrostachyum* | Amaranthaceae | FR2012 | 2012-96-D | 48.48 | 64 |
| 63 | Secoviridae | Nepovirus | RNA1 polyprotein | *Not identified* |  | SA2010 | 1J-021 | 47.00 | 66 |
| 64 | Secoviridae | Nepovirus | Polyprotein 1 | *Cysticapnos vesicaria* | Papaveraceae | SA2010 | 1B-089 | 50.10 | 67 |
| 65 | Secoviridae | Comovirus | Polyprotein | *Galenia africana* | Aizoaceae | SA2010 | 1D-012 | 46.77 | 68 |
| 66 | Unclassified | Sobemovirus | Polyprotein | *Staberoha distachyos* | Restionaceae | SA2010 | 1G-010 | 46.55 | 69 |
| 67 | Tombusviridae | Alphanecrovirus | RNA-dependent RNA polymerase | *Puccinellia festuciformis* | Poaceae | FR2010 | 74-1A | 37.01 | 70-72 |
| 68 | Tombusviridae | Avenavirus | RNA-dependent RNA polymerase | *Oryza sativa* | Poaceae | FR2012 | 2012-08-E | 47.37 | 73 |
| 69 | Tombusviridae | Avenavirus | RNA-dependent RNA polymerase | *Briza maxima* | Poaceae | SA2010 | 1E-050 | 41.51 | 76 |
| 70 | Tombusviridae | Carmovirus | RNA-dependent RNA polymerase | *Plantago sp.* | Plantaginaceae | FR2012 | 2012-20-B | 57.41 | 71-73, 75-77 |
| 71 | Tombusviridae | Carmovirus | p88 replicase | *Oryza sativa* | Poaceae | FR2012 | 2012-05-C | 53.90 | 75-77 |
| 72 | Tombusviridae | Carmovirus | RNA-dependent RNA polymerase | *Clutia alaternoides* | Euphorbiaceae | SA2010 | 1D-057 | 53.57 | 72 |
| 73 | Tombusviridae | Unclassified | Coat protein | *Trifolium sp.* | Fabaceae | FR2012 | 2012-47-D | 45.61 | 79 |
| 74 | Tombusviridae | Unclassified | 41K protein | *Phragmites australis* | Poaceae | FR2012 | 2012-19-E | 41.67 | 78 |
| 75 | Tombusviridae | Umbravirus | RNA-dependent RNA polymerase | *Elytrigia acuta* | Poaceae | FR2012 | 2012-75-B | 56.20 | 71, 73, 74 |
| 75 |  |  | RNA-dependent RNA polymerase | *Puccinellia fasciculata* | Poaceae | FR2012 | 2012-78-G | 50.00 | 71, 73 |
| 76 | Tombusviridae | Umbravirus | RNA-dependent RNA polymerase | *Rhus laevigata* | Anacardiaceae | SA2010 | 1D-006 | 56.38 | 74, 77 |
| 77 | Tombusviridae | Umbravirus | RNA-dependent RNA polymerase | *Ehrharta calycina* | Poaceae | SA2010 | 1D-068 | 55.07 | 74, 77 |
| 78 | Tombusviridae | Betanecrovirus | RNA-dependent RNA polymerase | *Pennisetum sp.* | Poaceae | SA2010 | 1I-090 | 40.98 | 77 |
| 79 | Tombusviridae | Macanavirus | Polymerase-associated protein | *Avena byzantina* | Poaceae | SA2010 | 1F-069 | 41.13 | 71 |
| 80 | Tombusviridae | Panicovirus | RNA-dependent RNA polymerase | *Avena fatua* | Poaceae | SA2010 | 1K-027 | 64.06 | 71-73, 76, 77 |
| 81 | Tombusviridae | Panicovirus | p106 | *Briza maxima* | Poaceae | SA2010 | 1A-085 | 52.22 | 72 |
| 82 | Tymoviridae | Marafivirus | Polyprotein | *Medicago sativa* | Fabaceae | FR2012 | 2012-48-E | 52.83 | 80 |
| 82 |  |  | Polyprotein | *Medicago sativa* | Fabaceae | FR2012 | 2012-48-C | 40.35 | 80 |
| 83 | Tymoviridae | Maculavirus | Helicase | *Capnophyllum africanum* | Apiaceae | SA2010 | 1B-051 | 45.16 | 81 |
| 84 | Tymoviridae | Maculavirus | Replicase-associated protein | *Willdenowia incurvata* | Restionaceae | SA2010 | 1D-041 | 39.33 | 81 |
| 85 | Tymoviridae | Unclassified | Replicase-associated protein | *Medicago sativa* | Fabaceae | FR2012 | 2012-27-A | 63.16 | 82 |
| 86 | Tymoviridae | Maculavirus | Replicase | *Raphanus sp.* | Brassicaceae | SA2010 | 1A-096 | 57.58 | 83 |
| 87 | Tymoviridae | Tymovirus | Replicase | *Phalaris minor* | Poaceae | SA2010 | 1C-093 | 37.65 | 80 |
| 88 | Unclassified viruses^b^ |  | ORF2 | *Oryza sativa* | Poaceae | FR2012 | 2012-09-D | 70.59 | - |
| 89 | Unclassified viruses^c^ |  | Polyprotein | *Verbena officinalis* | Verbenaceae | FR2012 | 2012-58-G | 36.96 | 84 |
| 90 | Unclassified viruses^d^ |  | p9 | *Salicornia fruticosa* | Amaranthaceae | FR2012 | 2012-74-F | 46.30 | - |
| 91 | Unclassified viruses^e^ |  | Polymerase | *Not identified* |  | SA2010 | 1F-051 | 40.85 | 85 |
| 92 | Virgaviridae | Tobamovirus | Replicase | *Microloma sp.* | Apocynaceae | SA2010 | 1D-034 | 55.00 | 86 |
| 93 | Virgaviridae | Tobravirus | RNA polymerase | *Lolium perenne* | Poaceae | SA2010 | 1E-074 | 29.52 | 87 |
| 94 | Virgaviridae | Pomovirus | Replicase | *Gazania pectinata* | Asteraceae | SA2010 | 1C-017 | 36.19 | 88 |

^a^Supplementary Figure 2

^b^Tombunodavirus UC1

^c^Gentian Kobu-sho-associated virus

^d^Hibiscus green spot virus

^e^Blueberry necrotic ring blotch virus

**Variants of known plant virus species (26 OTUs, 151 plants)**

| **OTU** | **Family** | **Genus** | **Species** | **Coding region** | **Host plant** | **Plant Family** | **Location** | **Sample number** | **Identity** |
| --- | --- | --- | --- | --- | --- | --- | --- | --- | --- |
| 95 | Alphaflexiviridae | Potexvirus | White clover mosaic virus | Putative RNA replicase | *Trifolium repens* | Fabaceae | FR2010 | 20-1E | 97.91 |
| 95 |  |  | White clover mosaic virus | Putative RNA replicase | *Lotus corniculatus* | Fabaceae | FR2010 | 33-1B | 95.45 |
| 95 |  |  | White clover mosaic virus | Putative triple gene block protein | ***Trifolium pratense*** | Fabaceae | FR2010 | 48-1D | 95.00 |
| 96 | Amalgaviridae | Amalgavirus | Southern tomato virus | Putative coat protein | *Solanum lycopersicum* | Solanaceae | FR2010 | 49-1D | 99.24 |
| 96 |  |  | Southern tomato virus | Fusion protein | ***Solanum cf villosum*** | Solanaceae | FR2012 | 2012-99-E | 98.36 |
| 96 |  |  | Southern tomato virus | Putative coat protein | ***Bolboschoenus maritimus*** | Cyperaceae | FR2012 | 2012-89-E | 99.28 |
| 96 |  |  | Southern tomato virus | Putative coat protein | ***Typha domingensis*** | Typhaceae | FR2012 | 2012-57-E | 98.82 |
| 96 |  |  | Southern tomato virus | Putative coat protein | ***Chenopodium album*** | Amaranthaceae | FR2012 | 2012-99-D | 98.71 |
| 97 | Betaflexiviridae | Unclassified | Carrot carlavirus WM-2008 | Coat protein | *Foeniculum vulgare* | Apiaceae | FR2012 | 2012-17-F | 88.00 |
| 98 | Betaflexiviridae | Carlavirus | Red clover vein mosaic virus | Coat protein | *Oryza sativa* | Poaceae | FR2012 | 2012-39-A | 97.01 |
| 99 | Bromoviridae | Alfamovirus | Alfalfa mosaic virus | 126 kDa protein | *Medicago sativa* | Fabaceae | FR2012 | 2012-48-E | 100.00 |
| 99 |  |  | Alfalfa mosaic virus | 126 kDa protein | *Medicago sativa* | Fabaceae | FR2012 | 2012-48-C | 100.00 |
| 100 | Bromoviridae | Cucumovirus | Cucumber mosaic virus | Replicase | *Lupinus sp.* | Fabaceae | SA2010 | 1D-018 | 99.19 |
| 100 |  |  | Cucumber mosaic virus | Replicase | ***Avena byzantina*** | Poaceae | SA2010 | 1C-088 | 98.67 |
| 101 | Caulimoviridae | Caulimovirus | Soybean Putnam virus | Aphid transmission factor | ***Circium arvense*** | Asteraceae | FR2012 | 2012-29-D | 94.87 |
| 102 | Endornaviridae | Endornavirus | Oryza sativa endornavirus | Polyprotein | ***Trifolium resupinatum*** | Fabaceae | FR2012 | 2012-47-C | 87.50 |
| 102 |  |  | Oryza sativa endornavirus | Polyprotein | *Oryza sativa* | Poaceae | FR2010 | 46-1B | 96.97 |
| 102 |  |  | Oryza sativa endornavirus | Polyprotein | *Oryza sativa* | Poaceae | FR2010 | 37-1C | 98.47 |
| 102 |  |  | Oryza sativa endornavirus | Polyprotein | *Oryza sativa* | Poaceae | FR2010 | 39-1A | 95.83 |
| 102 |  |  | Oryza sativa endornavirus | Polyprotein | *Oryza sativa* | Poaceae | FR2010 | 46-1E | 90.00 |
| 102 |  |  | Oryza sativa endornavirus | Polyprotein | *Oryza sativa* | Poaceae | FR2010 | 38-1E | 98.57 |
| 102 |  |  | Oryza sativa endornavirus | Polyprotein | *Oryza sativa* | Poaceae | FR2010 | 19-1A | 98.14 |
| 102 |  |  | Oryza sativa endornavirus | Polyprotein | *Oryza sativa* | Poaceae | FR2010 | 38-1C | 98.32 |
| 102 |  |  | Oryza sativa endornavirus | Polyprotein | *Oryza sativa* | Poaceae | FR2010 | 40-1B | 95.24 |
| 102 |  |  | Oryza sativa endornavirus | Polyprotein | *Oryza sativa* | Poaceae | FR2010 | 38-1D | 98.94 |
| 102 |  |  | Oryza sativa endornavirus | Polyprotein | *Oryza sativa* | Poaceae | FR2010 | 70-1C | 97.44 |
| 102 |  |  | Oryza sativa endornavirus | Polyprotein | *Oryza sativa* | Poaceae | FR2010 | 70-1D | 94.89 |
| 102 |  |  | Oryza sativa endornavirus | Polyprotein | *Oryza sativa* | Poaceae | FR2010 | 88-1E | 96.67 |
| 102 |  |  | Oryza sativa endornavirus | Polyprotein | *Oryza sativa* | Poaceae | FR2010 | 60-1D | 98.20 |
| 102 |  |  | Oryza sativa endornavirus | Polyprotein | *Oryza sativa* | Poaceae | FR2010 | 60-1A | 95.20 |
| 102 |  |  | Oryza sativa endornavirus | Polyprotein | *Oryza sativa* | Poaceae | FR2010 | 60-1B | 98.73 |
| 102 |  |  | Oryza sativa endornavirus | Polyprotein | *Oryza sativa* | Poaceae | FR2010 | 88-1C | 88.61 |
| 102 |  |  | Oryza sativa endornavirus | Polyprotein | *Oryza sativa* | Poaceae | FR2010 | 88-1A | 97.44 |
| 102 |  |  | Oryza sativa endornavirus | Polyprotein | *Oryza sativa* | Poaceae | FR2010 | 70-1A | 96.55 |
| 102 |  |  | Oryza sativa endornavirus | Polyprotein | *Oryza sativa* | Poaceae | FR2010 | 97-1B | 97.30 |
| 102 |  |  | Oryza sativa endornavirus | Polyprotein | *Oryza sativa* | Poaceae | FR2010 | 97-1E | 97.64 |
| 102 |  |  | Oryza sativa endornavirus | Polyprotein | *Oryza sativa* | Poaceae | FR2010 | 46-1C | 93.33 |
| 102 |  |  | Oryza sativa endornavirus | Polyprotein | *Oryza sativa* | Poaceae | FR2012 | 2012-08-D | 88.46 |
| 102 |  |  | Oryza sativa endornavirus | Polyprotein | *Oryza sativa* | Poaceae | FR2012 | 2012-16-C | 100.00 |
| 102 |  |  | Oryza sativa endornavirus | Polyprotein | *Oryza sativa* | Poaceae | FR2012 | 2012-05-D | 97.93 |
| 102 |  |  | Oryza sativa endornavirus | Polyprotein | *Oryza sativa* | Poaceae | FR2012 | 2012-16-B | 94.76 |
| 102 |  |  | Oryza sativa endornavirus | Polyprotein | *Oryza sativa* | Poaceae | FR2012 | 2012-05-B | 95.90 |
| 102 |  |  | Oryza sativa endornavirus | Polyprotein | *Oryza sativa* | Poaceae | FR2012 | 2012-08-E | 91.78 |
| 102 |  |  | Oryza sativa endornavirus | Polyprotein | *Oryza sativa* | Poaceae | FR2012 | 2012-16-A | 98.80 |
| 102 |  |  | Oryza sativa endornavirus | Polyprotein | *Oryza sativa* | Poaceae | FR2012 | 2012-19-G | 98.99 |
| 102 |  |  | Oryza sativa endornavirus | Polyprotein | *Oryza sativa* | Poaceae | FR2012 | 2012-18-D | 97.87 |
| 102 |  |  | Oryza sativa endornavirus | Polyprotein | *Oryza sativa* | Poaceae | FR2012 | 2012-18-B | 97.28 |
| 102 |  |  | Oryza sativa endornavirus | Polyprotein | *Oryza sativa* | Poaceae | FR2012 | 2012-08-A | 90.32 |
| 102 |  |  | Oryza sativa endornavirus | Polyprotein | *Oryza sativa* | Poaceae | FR2012 | 2012-05-C | 91.04 |
| 102 |  |  | Oryza sativa endornavirus | Polyprotein | *Oryza sativa* | Poaceae | FR2012 | 2012-18-E | 98.85 |
| 102 |  |  | Oryza sativa endornavirus | Polyprotein | *Oryza sativa* | Poaceae | FR2012 | 2012-90-B | 98.31 |
| 102 |  |  | Oryza sativa endornavirus | Polyprotein | *Oryza sativa* | Poaceae | FR2012 | 2012-66-D | 97.35 |
| 102 |  |  | Oryza sativa endornavirus | Polyprotein | *Oryza sativa* | Poaceae | FR2012 | 2012-46-B | 99.26 |
| 102 |  |  | Oryza sativa endornavirus | Polyprotein | *Oryza sativa* | Poaceae | FR2012 | 2012-56-D | 96.88 |
| 102 |  |  | Oryza sativa endornavirus | Polyprotein | *Oryza sativa* | Poaceae | FR2012 | 2012-57-A | 81.25 |
| 102 |  |  | Oryza sativa endornavirus | Polyprotein | *Oryza sativa* | Poaceae | FR2012 | 2012-39-F | 98.65 |
| 102 |  |  | Oryza sativa endornavirus | Polyprotein | *Oryza sativa* | Poaceae | FR2012 | 2012-66-B | 97.59 |
| 102 |  |  | Oryza sativa endornavirus | Polyprotein | *Oryza sativa* | Poaceae | FR2012 | 2012-90-C | 100.00 |
| 102 |  |  | Oryza sativa endornavirus | Polyprotein | *Oryza sativa* | Poaceae | FR2012 | 2012-66-C | 100.00 |
| 102 |  |  | Oryza sativa endornavirus | Polyprotein | *Oryza sativa* | Poaceae | FR2012 | 2012-79-C | 97.74 |
| 102 |  |  | Oryza sativa endornavirus | Polyprotein | *Oryza sativa* | Poaceae | FR2012 | 2012-80-C | 96.46 |
| 102 |  |  | Oryza sativa endornavirus | Polyprotein | *Oryza sativa* | Poaceae | FR2012 | 2012-37-D | 95.25 |
| 102 |  |  | Oryza sativa endornavirus | Polyprotein | *Oryza sativa* | Poaceae | FR2012 | 2012-35-A | 94.55 |
| 102 |  |  | Oryza sativa endornavirus | Polyprotein | *Oryza sativa* | Poaceae | FR2012 | 2012-39-B | 93.62 |
| 102 |  |  | Oryza sativa endornavirus | Polyprotein | *Oryza sativa* | Poaceae | FR2012 | 2012-69-C | 96.55 |
| 102 |  |  | Oryza sativa endornavirus | Polyprotein | *Oryza sativa* | Poaceae | FR2012 | 2012-37-F | 93.09 |
| 102 |  |  | Oryza sativa endornavirus | Polyprotein | *Oryza sativa* | Poaceae | FR2012 | 2012-84-E | 95.61 |
| 102 |  |  | Oryza sativa endornavirus | Polyprotein | *Oryza sativa* | Poaceae | FR2012 | 2012-80-E | 90.41 |
| 102 |  |  | Oryza sativa endornavirus | Polyprotein | *Oryza sativa* | Poaceae | FR2012 | 2012-46-C | 92.19 |
| 102 |  |  | Oryza sativa endornavirus | Polyprotein | *Oryza sativa* | Poaceae | FR2012 | 2012-37-E | 98.59 |
| 102 |  |  | Oryza sativa endornavirus | Polyprotein | *Oryza sativa* | Poaceae | FR2012 | 2012-35-C | 99.25 |
| 102 |  |  | Oryza sativa endornavirus | Polyprotein | *Oryza sativa* | Poaceae | FR2012 | 2012-95-C | 82.04 |
| 102 |  |  | Oryza sativa endornavirus | Polyprotein | *Oryza sativa* | Poaceae | FR2012 | 2012-26-B | 85.93 |
| 102 |  |  | Oryza sativa endornavirus | Polyprotein | *Oryza sativa* | Poaceae | FR2012 | 2012-97-A | 98.48 |
| 102 |  |  | Oryza sativa endornavirus | Polyprotein | *Oryza sativa* | Poaceae | FR2012 | 2012-94-B | 95.83 |
| 102 |  |  | Oryza sativa endornavirus | Polyprotein | *Oryza sativa* | Poaceae | FR2012 | 2012-97-B | 98.51 |
| 102 |  |  | Oryza sativa endornavirus | Polyprotein | *Oryza sativa* | Poaceae | FR2012 | 2012-26-A | 99.11 |
| 102 |  |  | Oryza sativa endornavirus | Polyprotein | *Oryza sativa* | Poaceae | FR2012 | 2012-84-D | 93.01 |
| 102 |  |  | Oryza sativa endornavirus | Polyprotein | *Oryza sativa* | Poaceae | FR2012 | 2012-94-A | 92.70 |
| 102 |  |  | Oryza sativa endornavirus | Polyprotein | *Oryza sativa* | Poaceae | FR2012 | 2012-46-A | 97.96 |
| 102 |  |  | Oryza sativa endornavirus | Polyprotein | *Oryza sativa* | Poaceae | FR2012 | 2012-94-C | 91.23 |
| 102 |  |  | Oryza sativa endornavirus | Polyprotein | *Oryza sativa* | Poaceae | FR2012 | 2012-35-B | 98.11 |
| 102 |  |  | Oryza sativa endornavirus | Polyprotein | *Oryza sativa* | Poaceae | FR2012 | 2012-97-F | 86.36 |
| 102 |  |  | Oryza sativa endornavirus | Polyprotein | ***Hordeum marinum*** | Poaceae | FR2010 | 15-1C | 92.07 |
| 102 |  |  | Oryza sativa endornavirus | Polyprotein | ***Echinochloa sp.*** | Poaceae | FR2012 | 2012-88-A | 96.15 |
| 102 |  |  | Oryza sativa endornavirus | Polyprotein | ***Echinochloa sp.*** | Poaceae | FR2012 | 2012-49-A | 81.48 |
| 102 |  |  | Oryza sativa endornavirus | Polyprotein | *Not identified* |  | FR2012 | 2012-80-D | 94.69 |
| 103 | Luteoviridae | Luteovirus | Soybean dwarf virus | Movement protein | *Trifolium repens* | Fabaceae | FR2010 | 43-1D | 88.37 |
| 103 | Luteoviridae | Luteovirus | Soybean dwarf virus | Replicase | ***Trifolium angustifolium*** | Fabaceae | FR2012 | 2012-28-B | 75.00 |
| 104 | Luteoviridae | Luteovirus | Bean leafroll virus | Coat protein | *Medicago sativa* | Fabaceae | FR2012 | 2012-27-A | 96.77 |
| 105 | Luteoviridae | Luteovirus | Barley yellow dwarf virus-PAV | RNA-dependent RNA polymerase | *Not identified* | Poaceae | FR2010 | 78-1E | 97.47 |
| 105 |  |  | Barley yellow dwarf virus-PAV | Genome-linked protein | *Triticum sp.* | Poaceae | FR2010 | 67-1C | 94.70 |
| 105 |  |  | Barley yellow dwarf virus-PAV | Coat protein | *Hordeum sp.* | Poaceae | FR2010 | 27-1B | 88.89 |
| 105 |  |  | Barley yellow dwarf virus-PAV | RNA-dependent RNA polymerase | *Triticum sp.* | Poaceae | FR2010 | 10-1A | 84.78 |
| 105 |  |  | Barley yellow dwarf virus-PAV | RNA-dependent RNA polymerase | *Triticum sp.* | Poaceae | FR2010 | 10-1C | 84.07 |
| 105 |  |  | Barley yellow dwarf virus-PAV | RNA-dependent RNA polymerase | *Triticum sp.* | Poaceae | FR2010 | 49-1C | 100.00 |
| 105 |  |  | Barley yellow dwarf virus-PAV | RNA-dependent RNA polymerase | *Triticum sp.* | Poaceae | FR2010 | 99-1B | 96.71 |
| 105 |  |  | Barley yellow dwarf virus-PAV | RNA-dependent RNA polymerase | *Triticum sp.* | Poaceae | FR2010 | 10-1E | 94.44 |
| 105 |  |  | Barley yellow dwarf virus-PAV | RNA-dependent RNA polymerase | *Triticum sp.* | Poaceae | FR2010 | 68-1B | 94.29 |
| 105 |  |  | Barley yellow dwarf virus-PAV | Readthrough domain | *Triticum sp.* | Poaceae | FR2010 | 48-1E | 98.90 |
| 105 |  |  | Barley yellow dwarf virus-PAV | Polymerase | ***Puccinellia festuciformis*** | Poaceae | FR2010 | 74-1A | 97.81 |
| 105 |  |  | Barley yellow dwarf virus-PAV | Not identified | *Triticum turgidum* | Poaceae | FR2012 | 2012-38-C | 98.23 |
| 105 |  |  | Barley yellow dwarf virus-PAV | P1 | *Not identified* | Poaceae | FR2012 | 2012-38-G | 96.04 |
| 105 |  |  | Barley yellow dwarf virus-PAV | P6 protein | ***Hordeum marinum*** | Poaceae | FR2012 | 2012-15-F | 96.15 |
| 105 |  |  | Barley yellow dwarf virus-PAV | RNA-dependent RNA polymerase | *Trticum sp.* | Poaceae | FR2012 | 2012-10-A | 97.13 |
| 105 |  |  | Barley yellow dwarf virus-PAS | RNA-dependent RNA polymerase | *Triticum sp.* | Poaceae | FR2010 | 67-1E | 97.70 |
| 105 |  |  | Barley yellow dwarf virus-PAS | Not identified | ***Schedonorus arundinaceus*** | Poaceae | FR2012 | 2012-20-C | 99.06 |
| 105 |  |  | Barley yellow dwarf virus-PAV | RNA-dependent RNA polymerase | ***Schedonorus arundinaceus*** | Poaceae | FR2012 | 2012-43-H | 98.07 |
| 105 |  |  | Barley yellow dwarf virus-PAV | P1 | *Triticum turgidum* | Poaceae | FR2012 | 2012-38-D | 97.70 |
| 105 |  |  | Barley yellow dwarf virus-PAV | P1 | *Triticum turgidum* | Poaceae | FR2012 | 2012-10-B | 100.00 |
| 105 |  |  | Barley yellow dwarf virus-PAV | RNA-dependent RNA polymerase | *Triticum turgidum* | Poaceae | FR2012 | 2012-10-F | 100.00 |
| 105 |  |  | Barley yellow dwarf virus-PAV | RNA-dependent RNA polymerase | *Triticum turgidum* | Poaceae | FR2012 | 2012-28-A | 100.00 |
| 105 |  |  | Barley yellow dwarf virus-PAV | Orf5 | ***Lolium perenne*** | Poaceae | FR2012 | 2012-78-B | 99.12 |
| 105 |  |  | Barley yellow dwarf virus-PAV | Orf5 | *Triticum turgidum* | Poaceae | SA2010 | 1F-093 | 95.74 |
| 105 |  |  | Barley yellow dwarf virus-PAV | Coat protein | ***Avena byzantina*** | Poaceae | SA2010 | 1G-097 | 96.50 |
| 105 |  |  | Barley yellow dwarf virus-PAV | P1 | ***Avena byzantina*** | Poaceae | SA2010 | 1G-099 | 92.80 |
| 105 |  |  | Barley yellow dwarf virus-PAV | P1 | *Avena sp.* | Poaceae | SA2010 | 1C-089 | 95.00 |
| 105 |  |  | Barley yellow dwarf virus-PAV | P1 | *Triticum turgidum* | Poaceae | SA2010 | 1B-094 | 98.40 |
| 105 |  |  | Barley yellow dwarf virus-PAV | RNA-dependent RNA polymerase | *Triticum turgidum* | Poaceae | SA2010 | 1E-094 | 98.85 |
| 105 |  |  | Barley yellow dwarf virus-PAV | P1 | ***Lolium perenne*** | Poaceae | SA2010 | 1E-074 | 100.00 |
| 105 |  |  | Barley yellow dwarf virus-PAV | ORF2 | ***Anisantha diandra*** | Poaceae | SA2010 | 1G-086 | 100.00 |
| 106 | Luteoviridae | Polerovirus | Chickpea chlorotic stunt virus | RNA-dependent RNA polymerase | ***Trifolium resupinatum*** | Fabaceae | FR2012 | 2012-32-J | 99.11 |
| 107 | Luteoviridae | Polerovirus | Cereal yellow dwarf virus-RPS | RNA-dependent RNA polymerase | ***Willdenowia incurvata*** | Restionaceae | SA2010 | 1I-083 | 86.49 |
| 108 | Luteoviridae | Polerovirus | Beet chlorosis virus | Putative protein P5 | *Not identified* | Brassicaceae | SA2010 | 1C-069 | 95.45 |
| 109 | Luteoviridae | Polerovirus | Turnip yellows virus | CP read-through protein | ***Cysticapnos vesicaria*** | Papaveraceae | SA2010 | 1E-100 | 96.74 |
| 109 |  |  | Turnip yellows virus | Hypothetical protein | ***Raphanus sp.*** | Brassicaceae | SA2010 | 1A-096 | 100.00 |
| 109 |  |  | Turnip yellows virus | Hypothetical protein | ***Raphanus sp.*** | Brassicaceae | SA2010 | 1B-100 | 95.51 |
| 110 | Luteoviridae | Polerovirus | Sugarcane yellow leaf virus | Putative movement protein p17 | ***Aeluropus littoralis*** | Poaceae | FR2012 | 2012-82-C | 86.17 |
| 111 | Potyviridae | Potyvirus | Clover yellow vein virus | Polyprotein | *Trifolium repens* | Fabaceae | FR2010 | 18-1B | 96.27 |
| 112 | Potyviridae | Potyvirus | Beet mosaic virus | NIa-VPg protein | ***Salicornia fruticosa*** | Amaranthaceae | FR2010 | 63-1D | 94.59 |
| 112 | Potyviridae | Potyvirus | Beet mosaic virus | P3 protein | ***Arthrocnemum macrostachyum*** | Amaranthaceae | FR2012 | 2012-65-C | 70.59 |
| 112 | Potyviridae | Potyvirus | Beet mosaic virus | P3 protein | ***Salicornia fruticosa*** | Amaranthaceae | FR2012 | 2012-51-A | 69.53 |
| 112 | Potyviridae | Potyvirus | Beet mosaic virus | Polyprotein | ***Salicornia fruticosa*** | Amaranthaceae | FR2012 | 2012-64-E | 93.48 |
| 113 | Potyviridae | Tritimovirus | Brome streak mosaic virus | P1 protein | ***Avena barbata*** | Poaceae | FR2012 | 2012-17-A | 98.78 |
| 113 | Potyviridae | Tritimovirus | Brome streak mosaic virus | P1 | ***avena barbata*** | Poaceae | FR2012 | 2012-39-C | 98.78 |
| 113 | Potyviridae | Tritimovirus | Brome streak mosaic virus | NIb protein | ***Hordeum murinum*** | Poaceae | FR2012 | 2012-48-B | 98.32 |
| 114 | Potyviridae | Tritimovirus | Wheat streak mosaic virus | NIb protein | *Hordeum sp.* | Poaceae | FR2010 | 27-1B | 99.08 |
| 114 | Potyviridae | Tritimovirus | Wheat streak mosaic virus | Polyprotein | ***Avena byzantina*** | Poaceae | SA2010 | 1E-068 | 92.05 |
| 115 | Secoviridae | Nepovirus | Tomato black ring virus | Polyprotein | ***Lolium perenne*** | Poaceae | FR2012 | 2012-78-B | 95.30 |
| 116 | Unclassified | Sobemovirus | Sowbane mosaic virus | P2b | ***Atriplex prostrata*** | Chenopodiaceae | FR2012 | 2012-27-C | 95.30 |
| 116 |  |  | Sowbane mosaic virus | P2b | ***Atriplex prostrata*** | Chenopodiaceae | FR2012 | 2012-45-H | 86.79 |
| 117 | Tombusviridae | Unclassified | TGP Tombusvirid 1 | Putative replicase | *Microloma sp.* | Apocynaceae | SA2010 | 1B-039 | 87.36 |
| 118 | Tombusviridae | Carmovirus | Pelargonium line pattern virus | p87 | ***Tamarix gallica*** | Tamaricaceae | FR2012 | 2012-74-G | 89.29 |
| 119 | Virgaviridae | Hordeivirus | Barley stripe mosaic virus | Alpha protein | ***Schedonorus arundinaceus*** | Poaceae | FR2010 | 33-1C | 87.43 |
| 119 |  |  | Barley stripe mosaic virus | Alpha protein | ***Schedonorus arundinaceus*** | Poaceae | FR2010 | 34-1E | 78.49 |
| 119 |  |  | Barley stripe mosaic virus | Alpha protein | ***Schedonorus arundinaceus*** | Poaceae | FR2010 | 36-1A | 88.19 |
| 119 |  |  | Barley stripe mosaic virus | Alpha protein | ***Festuca arundinacea*** | Poaceae | FR2012 | 2012-05-E | 84.00 |
| 119 |  |  | Barley stripe mosaic virus | Alpha protein | *Avena sp.* | Poaceae | FR2012 | 2012-09-A | 79.21 |
| 119 |  |  | Barley stripe mosaic virus | Alpha protein | ***Schedonorus arundinaceus*** | Poaceae | FR2012 | 2012-58-A | 85.15 |
| 119 |  |  | Barley stripe mosaic virus | Alpha protein | ***Schedonorus arundinaceus*** | Poaceae | FR2012 | 2012-44-B | 81.98 |
| 119 |  |  | Barley stripe mosaic virus | Beta-B protein | ***Schedonorus arundinaceus*** | Poaceae | FR2012 | 2012-33-E | 91.89 |
| 119 |  |  | Barley stripe mosaic virus | Alpha protein | ***Schedonorus arundinaceus*** | Poaceae | FR2012 | 2012-44-E | 93.12 |
| 120 | Virgaviridae | Tobravirus | Tobacco rattle virus | 57-kDa polymerase | *Lotus sp.* | Fabaceae | SA2010 | 1C-099 | 99.15 |

Bold: uncultivated plant species containing agriculturally important viral pathogens

**Unclassified Plant viruses (21 single reads and contigs, 21 plants)**

| **Family** | **Genus** | **Coding region** | **Host plant** | **Plant Family** | **Location** | **Sample number** | **Identity** | **Phylogenetic tree number*** |
| --- | --- | --- | --- | --- | --- | --- | --- | --- |
| Amalgaviridae | Amalgavirus | Fusion protein | *Lolium perenne* | Poaceae | SA2010 | 1E-074 | 53.85 | 90 |
| Amalgaviridae | Amalgavirus | Fusion protein | *Passerina corymbosa* | Thymelaceae | SA2010 | 1A-053 | 64.84 | 90 |
| Amalgaviridae | Amalgavirus | Fusion protein | *Phragmites australis* | Poaceae | FR2010 | 80-1C | 50.00 | 91 |
| Amalgaviridae | Amalgavirus | RNA-dependent RNA polymerase | *Arthrocnemum macrostachyum* | Amaranthaceae | FR2012 | 2012-65-C | 62.79 | 92 |
| Amalgaviridae | Amalgavirus | Fusion protein | *Oryza sativa* | Poaceae | FR2012 | 2012-39-A | 52.27 | 7 |
| Amalgaviridae | Amalgavirus | Putative fusion protein | *Schedonorus arundinaceus* | Poaceae | FR2012 | 2012-16-D | 53.66 | 14 |
| Amalgaviridae | Amalgavirus | Fusion protein | *Schedonorus arundinaceus* | Poaceae | FR2012 | 2012-58-A | 58.18 | 15 |
| Amalgaviridae | Amalgavirus | Fusion protein | *Iris pseudacorus* | Iridaceae | FR2012 | 2012-22-F | 35.42 | 94 |
| Amalgaviridae | Amalgavirus | Putative fusion protein | *Galenia africana* | Aizoaceae | SA2010 | 1B-012 | 55.81 | 91 |
| Amalgaviridae | Amalgavirus | Putative fusion protein | *Nemesia sp.* | Scrophulariaceae | SA2010 | 1H-002 | 75.23 | 9 |
| Amalgaviridae | Amalgavirus | RNA-dependent RNA polymerase | *Schedonorus arundinaceus* | Poaceae | FR2012 | 2012-44-B | 77.97 | 8 |
| Amalgaviridae | Amalgavirus | Fusion protein | *Gymnodiscus sp.* | Asteraceae | SA2010 | 1F-017 | 58.06 | 95 |
| Betaflexiviridae | Unclassified | Coat protein | *Indigofera heterophylla* | Fabaceae | SA2010 | 1C-024 | 65.09 | 93 |
|  |  |  | *Not identified* |  | SA2010 | 1I-006 | 64.62 | 93 |
| Bromoviridae | Ilarvirus | Polymerase P2 | *Bolboschoenus maritimus* | Cyperaceae | FR2012 | 2012-79-B | 73.58 | 22 |
| Endornaviridae | Endornavirus | Polyprotein | *Ammophyla sp.* | Poaceae | SA2010 | 1A-032 | 77.48 | 38 |
| Luteoviridae | Polerovirus | P1 protein | *Trifolium repens* | Fabaceae | FR2012 | 2012-43-K | 68.75 | 50 |
| Luteoviridae | Luteovirus | RNA-dependent RNA polymerase | *Lotus corniculatus* | Fabaceae | FR2010 | 47-1A | 74.02 | 48 |
|  |  | P3-P5 | *Trifolium repens* | Fabaceae | FR2010 | 44-1D | 77.62 | 47 |
| Luteoviridae | Polerovirus | RNA-dependent RNA polymerase | *Trifolium repens* | Fabaceae | FR2010 | 34-1D | 65.33 | 46 |
| Potyviridae | Potyvirus | P3 protein | *Halimione portulacoides* | Chenopodiaceae | FR2012 | 2012-11-C | 59.20 | 89 |

**Partitiviridae (166 single reads and contigs)**

| **Family** | **Genus** | **Coding region** | **Host plant** | **Plant Family** | **Location** | **Sample number** |
| --- | --- | --- | --- | --- | --- | --- |
| Partitiviridae | Alphapartitivirus | Coat protein | *Galenia africana* | Aizoaceae | SA2010 | 1B-012 |
| Partitiviridae | Alphapartitivirus | RNA-dependent RNA polymerase | *Galenia africana* | Aizoaceae | SA2010 | 1D-012 |
| Partitiviridae | Alphapartitivirus | RNA-dependent RNA polymerase | *Salicornia fruticosa* | Amaranthaceae | FR2012 | 2012-65-D |
| Partitiviridae | Alphapartitivirus | Coat protein | *Foeniculum vulgare* | Apiaceae | FR2012 | 2012-17-F |
| Partitiviridae | Alphapartitivirus | Coat protein | *Capnophyllum africanum* | Apiaceae | SA2010 | 1E-005 |
| Partitiviridae | Alphapartitivirus | RNA-dependent RNA polymerase | *Raphanus sp.* | Brassicaceae | SA2010 | 1B-100 |
| Partitiviridae | Alphapartitivirus | RNA-dependent RNA polymerase | *Halimione portulacoides* | Chenopodiaceae | FR2012 | 2012-51-Bbis |
| Partitiviridae | Alphapartitivirus | Coat protein | *Trifolium repens* | Fabaceae | FR2010 | 34-1D |
| Partitiviridae | Alphapartitivirus | Coat protein | *Trifolium repens* | Fabaceae | FR2010 | 20-1E |
| Partitiviridae | Alphapartitivirus | RNA-dependent RNA polymerase | *Melilotus alba* | Fabaceae | FR2010 | 48-1F |
| Partitiviridae | Alphapartitivirus | RNA-dependent RNA polymerase | *Medicago sativa* | Fabaceae | FR2010 | 48-1A |
| Partitiviridae | Alphapartitivirus | RNA-dependent RNA polymerase | *Medicago sativa* | Fabaceae | FR2010 | 48-1B |
| Partitiviridae | Alphapartitivirus | RNA-dependent RNA polymerase | *Medicago sativa* | Fabaceae | FR2012 | 2012-48-E |
| Partitiviridae | Alphapartitivirus | RNA-dependent RNA polymerase | *Medicago sativa* | Fabaceae | FR2012 | 2012-48-C |
| Partitiviridae | Alphapartitivirus | RNA-dependent RNA polymerase | *Medicago sativa* | Fabaceae | FR2012 | 2012-68-A |
| Partitiviridae | Alphapartitivirus | RNA-dependent RNA polymerase | *Trifolium resupinatum* | Fabaceae | FR2012 | 2012-32-J |
| Partitiviridae | Alphapartitivirus | RNA-dependent RNA polymerase | *Adenogramma sp.* | Molluginaceae | SA2010 | 1H-044 |
| Partitiviridae | Alphapartitivirus | RNA-dependent RNA polymerase | *Oxalis sp.* | Oxalidaceae | FR2012 | 2012-38-F |
| Partitiviridae | Alphapartitivirus | RNA-dependent RNA polymerase | *Schedonorus arundinaceus* | Poaceae | FR2010 | 33-1C |
| Partitiviridae | Alphapartitivirus | RNA-dependent RNA polymerase | *Schedonorus arundinaceus* | Poaceae | FR2010 | 36-1A |
| Partitiviridae | Alphapartitivirus | RNA-dependent RNA polymerase | *Schedonorus arundinaceus* | Poaceae | FR2010 | 32-1C |
| Partitiviridae | Alphapartitivirus | RNA-dependent RNA polymerase | *Triticum turgidum* | Poaceae | FR2012 | 2012-38-C |
| Partitiviridae | Alphapartitivirus | RNA-dependent RNA polymerase | *Schedonorus arundinaceus* | Poaceae | FR2012 | 2012-36-C |
| Partitiviridae | Alphapartitivirus | RNA-dependent RNA polymerase | *Oryza sativa* | Poaceae | FR2012 | 2012-39-A |
| Partitiviridae | Alphapartitivirus | RNA-dependent RNA polymerase | *Schedonorus arundinaceus* | Poaceae | FR2012 | 2012-33-E |
| Partitiviridae | Alphapartitivirus | RNA-dependent RNA polymerase | *Schedonorus arundinaceus* | Poaceae | FR2012 | 2012-21-A |
| Partitiviridae | Alphapartitivirus | RNA-dependent RNA polymerase | *Schedonorus arundinaceus* | Poaceae | FR2012 | 2012-44-E |
| Partitiviridae | Alphapartitivirus | RNA-dependent RNA polymerase | *Avena sp.* | Poaceae | SA2010 | 1C-089 |
| Partitiviridae | Alphapartitivirus | RNA-dependent RNA polymerase | *Briza maxima* | Poaceae | SA2010 | 1D-058 |
| Partitiviridae | Alphapartitivirus | RNA-dependent RNA polymerase | *Avena byzantina* | Poaceae | SA2010 | 1C-096 |
| Partitiviridae | Alphapartitivirus | RNA-dependent RNA polymerase | *Avena byzantina* | Poaceae | SA2010 | 1F-099 |
| Partitiviridae | Alphapartitivirus | RNA-dependent RNA polymerase | *Avena byzantina* | Poaceae | SA2010 | 1D-096 |
| Partitiviridae | Alphapartitivirus | RNA-dependent RNA polymerase | *Ehrharta calycina* | Poaceae | SA2010 | 1E-058 |
| Partitiviridae | Alphapartitivirus | RNA-dependent RNA polymerase | *Bromus sp.* | Poaceae | SA2010 | 1A-073 |
| Partitiviridae | Alphapartitivirus | Coat protein | *Stipagrostis sp.* | Poaceae | SA2010 | 1B-008 |
| Partitiviridae | Alphapartitivirus | RNA-dependent RNA polymerase | *Avena byzantina* | Poaceae | SA2010 | 1A-086 |
| Partitiviridae | Alphapartitivirus | RNA-dependent RNA polymerase | *Anisantha diandra* | Poaceae | SA2010 | 1A-027 |
| Partitiviridae | Alphapartitivirus | RNA-dependent RNA polymerase | *Lolium perenne* | Poaceae | SA2010 | 1E-074 |
| Partitiviridae | Alphapartitivirus | RNA-dependent RNA polymerase | *Willdenowia incurvata* | Restionaceae | SA2010 | 1A-052 |
| Partitiviridae | Alphapartitivirus | RNA-dependent RNA polymerase | *Willdenowia incurvata* | Restionaceae | SA2010 | 1D-041 |
| Partitiviridae | Alphapartitivirus | RNA-dependent RNA polymerase | *Nemesia sp.* | Scrophulariaceae | SA2010 | 1C-044 |
| Partitiviridae | Alphapartitivirus | Coat protein | *Bulbinella sp.* | [Xanthorrhoeaceae](http://en.wikipedia.org/wiki/Xanthorrhoeaceae) | SA2010 | 1C-005 |
| Partitiviridae | Alphapartitivirus | RNA-dependent RNA polymerase | *Not identified* |  | FR2012 | 2012-38-G |
| Partitiviridae | Alphapartitivirus | RNA-dependent RNA polymerase | *Not identified* |  | SA2010 | 1I-006 |
| Partitiviridae | Alphapartitivirus | RNA-dependent RNA polymerase | *Not identified* |  | SA2010 | 1G-062 |
| Partitiviridae | Betapartitivirus | Coat protein | *Ruschia sp.* | Aizoaceae | SA2010 | 1H-027 |
| Partitiviridae | Betapartitivirus | Coat protein | *Tulbaghia capensis* | Alliaceae | SA2010 | 1G-028 |
| Partitiviridae | Betapartitivirus | Coat protein | *Arctopus echinatus* | Apiaceae | SA2010 | 1A-058 |
| Partitiviridae | Betapartitivirus | RNA-dependent RNA polymerase | *Capnophyllum africanum* | Apiaceae | SA2010 | 1F-092 |
| Partitiviridae | Betapartitivirus | RNA-dependent RNA polymerase | *Capnophyllum africanum* | Apiaceae | SA2010 | 1B-051 |
| Partitiviridae | Betapartitivirus | Coat protein | *Asparagus maritimus* | Asparagaceae | FR2012 | 2012-41-E |
| Partitiviridae | Betapartitivirus | Coat protein | *Bidens sp.* | Asteraceae | FR2012 | 2012-09-C |
| Partitiviridae | Betapartitivirus | RNA-dependent RNA polymerase | *Lobularia sp.* | Brassicaceae | SA2010 | 1C-085 |
| Partitiviridae | Betapartitivirus | Coat protein | *Microcodon sp.* | Campanulaceae | SA2010 | 1F-011 |
| Partitiviridae | Betapartitivirus | Coat protein | *Trifolium pratense* | Fabaceae | FR2012 | 2012-48-F |
| Partitiviridae | Betapartitivirus | Coat protein | *Dipogon lignosus* | Fabaceae | SA2010 | 1H-084 |
| Partitiviridae | Betapartitivirus | Coat protein | *Ixia dubia* | Iridaceae | SA2010 | 1A-092 |
| Partitiviridae | Betapartitivirus | Coat protein | *Juncus gerardii* | Juncaceae | FR2010 | 25-1B |
| Partitiviridae | Betapartitivirus | Coat protein | *Lolium rigidum* | Poaceae | FR2010 | 72-1C |
| Partitiviridae | Betapartitivirus | Coat protein | *Bromus hordeaceus* | Poaceae | FR2010 | 61-1G |
| Partitiviridae | Betapartitivirus | RNA-dependent RNA polymerase | *Anisantha madritensis* | Poaceae | FR2010 | 14-1G |
| Partitiviridae | Betapartitivirus | Coat protein | *Triticum turgidum* | Poaceae | FR2012 | 2012-38-D |
| Partitiviridae | Betapartitivirus | Coat protein | *Puccinellia festuciformis* | Poaceae | FR2012 | 2012-87-C |
| Partitiviridae | Betapartitivirus | Coat protein | *Phragmites australis* | Poaceae | FR2012 | 2012-35-D |
| Partitiviridae | Betapartitivirus | Coat protein | *Cynodon dactylon* | Poaceae | FR2012 | 2012-68-B |
| Partitiviridae | Betapartitivirus | Coat protein | *Oryza sativa* | Poaceae | FR2012 | 2012-94-C |
| Partitiviridae | Betapartitivirus | RNA-dependent RNA polymerase | *Elytrigia sp.* | Poaceae | FR2012 | 2012-93-C |
| Partitiviridae | Betapartitivirus | Coat protein | *Phragmites australis* | Poaceae | FR2012 | 2012-22-G |
| Partitiviridae | Betapartitivirus | Coat protein | *Cynodon dactylon* | Poaceae | SA2010 | 1G-078 |
| Partitiviridae | Betapartitivirus | RNA-dependent RNA polymerase | *Triticum turgidum* | Poaceae | SA2010 | 1B-094 |
| Partitiviridae | Betapartitivirus | RNA-dependent RNA polymerase | *Ehrharta calycina* | Poaceae | SA2010 | 1D-068 |
| Partitiviridae | Betapartitivirus | Coat protein | *Phalaris minor* | Poaceae | SA2010 | 1C-093 |
| Partitiviridae | Betapartitivirus | RNA-dependent RNA polymerase | *Avena byzantina* | Poaceae | SA2010 | 1F-069 |
| Partitiviridae | Betapartitivirus | Coat protein | *Avena byzantina* | Poaceae | SA2010 | 1B-096 |
| Partitiviridae | Betapartitivirus | Coat protein | *Panicum sp.* | Poaceae | SA2010 | 1K-051 |
| Partitiviridae | Betapartitivirus | RNA-dependent RNA polymerase | *Lolium perenne* | Poaceae | SA2010 | 1D-086 |
| Partitiviridae | Betapartitivirus | Coat protein | *Phalaris minor* | Poaceae | SA2010 | 1C-094 |
| Partitiviridae | Betapartitivirus | Coat protein | *Panicum sp.* | Poaceae | SA2010 | 1G-061 |
| Partitiviridae | Betapartitivirus | Coat protein | *Anisantha diandra* | Poaceae | SA2010 | 1A-099 |
| Partitiviridae | Betapartitivirus | RNA-dependent RNA polymerase | *Ehrharta calycina* | Poaceae | SA2010 | 1J-075 |
| Partitiviridae | Betapartitivirus | RNA-dependent RNA polymerase | *Anisantha diandra* | Poaceae | SA2010 | 1F-068 |
| Partitiviridae | Betapartitivirus | RNA-dependent RNA polymerase | *Ranunculus sp.* | Ranunculaceae | FR2010 | 18-1H_ |
| Partitiviridae | Betapartitivirus | RNA-dependent RNA polymerase | *Thamnochortus spicigerus* | Restionaceae | SA2010 | 1F-006 |
| Partitiviridae | Betapartitivirus | RNA-dependent RNA polymerase | *Zaluzianskya sp.* | Scrophulariaceae | SA2010 | 1F-031 |
| Partitiviridae | Betapartitivirus | Coat protein | *Not identified* |  | FR2010 | 17-1E |
| Partitiviridae | Betapartitivirus | RNA-dependent RNA polymerase | *Not identified* |  | FR2010 | 32-1A |
| Partitiviridae | Betapartitivirus | RNA-dependent RNA polymerase | *Not identified* |  | FR2010 | 20-1D |
| Partitiviridae | Betapartitivirus | Coat protein | *Not identified* |  | SA2010 | 1B-068 |
| Partitiviridae | Deltapartitivirus | Coat protein | *Arthrocnemum macrostachyum* | Amaranthaceae | FR2012 | 2012-06-C |
| Partitiviridae | Deltapartitivirus | Coat protein | *Arthrocnemum macrostachyum* | Amaranthaceae | FR2012 | 2012-82-A |
| Partitiviridae | Deltapartitivirus | RNA-dependent RNA polymerase | *Arthrocnemum macrostachyum* | Amaranthaceae | FR2012 | 2012-12-A |
| Partitiviridae | Deltapartitivirus | RNA-dependent RNA polymerase | *Capnophyllum africanum* | Apiaceae | SA2010 | 1D-073 |
| Partitiviridae | Deltapartitivirus | RNA-dependent RNA polymerase | *Chenopodium sp.* | Chenopodiaceae | FR2010 | 29-1B |
| Partitiviridae | Deltapartitivirus | Coat protein | *Atriplex prostrata* | Chenopodiaceae | FR2012 | 2012-27-C |
| Partitiviridae | Deltapartitivirus | RNA-dependent RNA polymerase | *Atriplex prostrata* | Chenopodiaceae | FR2012 | 2012-45-E |
| Partitiviridae | Deltapartitivirus | RNA-dependent RNA polymerase | *Trifolium sp.* | Fabaceae | FR2012 | 2012-47-D |
| Partitiviridae | Deltapartitivirus | RNA-dependent RNA polymerase | *Avena sp.* | Poaceae | FR2012 | 2012-09-A |
| Partitiviridae | Deltapartitivirus | RNA-dependent RNA polymerase | *Verbascum sinuatum* | Scrophulariaceae | FR2012 | 2012-30-B |
| Partitiviridae | Deltapartitivirus | RNA-dependent RNA polymerase | *Not identified* |  | FR2010 | 81-1A |
| Partitiviridae | Deltapartitivirus | Coat protein | *Not identified* |  | SA2010 | 1B-077 |
| Partitiviridae | Deltapartitivirus | RNA-dependent RNA polymerase | *Not identified* |  | SA2010 | 1A-090 |
| Partitiviridae | Gammapartitivirus | RNA-dependent RNA polymerase | *Stachys aethiopica* | Lamiaceae | SA2010 | 1C-091 |
| Partitiviridae | Gammapartitivirus | RNA-dependent RNA polymerase | *Phragmites australis* | Poaceae | FR2010 | 2012-75-A |
| Partitiviridae | Gammapartitivirus | RNA-dependent RNA polymerase | *Aeluropus littoralis* | Poaceae | FR2012 | 2012-81-B |
| Partitiviridae | Gammapartitivirus | RNA-dependent RNA polymerase | *Staberoha distachyos* | Restionaceae | SA2010 | 1G-001 |
| Partitiviridae | Gammapartitivirus | RNA-dependent RNA polymerase | *Not identified* |  | SA2010 | 1D-028 |
| Partitiviridae | Gammapartitivirus | Coat protein | *Not identified* |  | SA2010 | 1D-074 |
| Partitiviridae | Unclassified | RNA-dependent RNA polymerase | *Schedonorus arundinaceus* | Poaceae | FR2010 | 31-1A |
| Partitiviridae | Unclassified | RNA-dependent RNA polymerase | *Arthrocnemum macrostachyum* | Amaranthaceae | FR2012 | 2012-96-D |
| Partitiviridae | Unclassified | RNA-dependent RNA polymerase | *Arthrocnemum macrostachyum* | Amaranthaceae | FR2012 | 2012-01-B |
| Partitiviridae | Unclassified | RNA-dependent RNA polymerase | *Capnophyllum africanum* | Apiaceae | SA2010 | 1B-035 |
| Partitiviridae | Unclassified | Coat protein | *Asparagus acutifolius* | Asparagaceae | FR2010 | 53-1A |
| Partitiviridae | Unclassified | Coat protein | *Asparagus acutifolius* | Asparagaceae | FR2010 | 61-1E |
| Partitiviridae | Unclassified | RNA-dependent RNA polymerase | *Gymnodiscus sp.* | Asteraceae | SA2010 | 1E-044 |
| Partitiviridae | Unclassified | RNA-dependent RNA polymerase | *Cineraria sp.* | Asteraceae | SA2010 | 1H-051 |
| Partitiviridae | Unclassified | Coat protein | *Gymnodiscus sp.* | Asteraceae | SA2010 | 1F-017 |
| Partitiviridae | Unclassified | RNA-dependent RNA polymerase | *Oncosiphon grandiflorum* | Asteraceae | SA2010 | 1E-037 |
| Partitiviridae | Unclassified | RNA-dependent RNA polymerase | *Raphanus sp.* | Brassicaceae | SA2010 | 1A-096 |
| Partitiviridae | Unclassified | RNA-dependent RNA polymerase | *Microcodon sp.* | Campanulaceae | SA2010 | 1A-037 |
| Partitiviridae | Unclassified | Coat protein | *Atriplex prostrata* | Chenopodiaceae | FR2012 | 2012-15-B |
| Partitiviridae | Unclassified | RNA-dependent RNA polymerase | *Carex cuprina* | Cyperaceae | FR2010 | 21-1A |
| Partitiviridae | Unclassified | Coat protein | *Euphorbia caput-medusae* | Euphorbiaceae | SA2010 | 1J-072 |
| Partitiviridae | Unclassified | Coat protein | *Trifolium nigrescens* | Fabaceae | FR2010 | 02-1B |
| Partitiviridae | Unclassified | Hypothetical protein | *Trifolium resupinatum* | Fabaceae | FR2010 | 51-1A |
| Partitiviridae | Unclassified | Coat protein | *Medicago sativa* | Fabaceae | FR2012 | 2012-27-A |
| Partitiviridae | Unclassified | RNA-dependent RNA polymerase | *Medicago sativa* | Fabaceae | FR2012 | 2012-44-F |
| Partitiviridae | Unclassified | RNA-dependent RNA polymerase | *Medicago sativa* | Fabaceae | FR2012 | 2012-54-F |
| Partitiviridae | Unclassified | Coat protein | *Trifolium pratense* | Fabaceae | FR2012 | 2012-31-L |
| Partitiviridae | Unclassified | RNA-dependent RNA polymerase | *Aspalathus sp.* | Fabaceae | SA2010 | 1G-079 |
| Partitiviridae | Unclassified | Coat protein | *Dipogon lignosus* | Fabaceae | SA2010 | 1E-023 |
| Partitiviridae | Unclassified | Hypothetical protein | *Pharnaceum incanum* | Molluginaceae | SA2010 | 1A-074 |
| Partitiviridae | Unclassified | RNA-dependent RNA polymerase | *Cysticapnos vesicaria* | Papaveraceae | SA2010 | 1E-027 |
| Partitiviridae | Unclassified | RNA-dependent RNA polymerase | *Limonium narbonense* | Plumbaginaceae | FR2012 | 2012-87-E |
| Partitiviridae | Unclassified | Hypothetical protein | *Oryza sativa* | Poaceae | FR2010 | 94-1B |
| Partitiviridae | Unclassified | Coat protein | *Triticum sp.* | Poaceae | FR2010 | 68-1A |
| Partitiviridae | Unclassified | RNA-dependent RNA polymerase | *Phragmites australis* | Poaceae | FR2010 | 66-1E |
| Partitiviridae | Unclassified | RNA-dependent RNA polymerase | *Schedonorus arundinaceus* | Poaceae | FR2012 | 2012-20-C |
| Partitiviridae | Unclassified | Coat protein | *Puccinellia distans* | Poaceae | FR2012 | 2012-15-H |
| Partitiviridae | Unclassified | Coat protein | *Avena barbata* | Poaceae | FR2012 | 2012-17-A |
| Partitiviridae | Unclassified | RNA-dependent RNA polymerase | *Schedonorus arundinaceus* | Poaceae | FR2012 | 2012-16-D |
| Partitiviridae | Unclassified | Coat protein | *Oryza sativa* | Poaceae | FR2012 | 2012-09-D |
| Partitiviridae | Unclassified | Coat protein | *Triticum sp.* | Poaceae | FR2012 | 2012-10-A |
| Partitiviridae | Unclassified | Replicase | *Oryza sativa* | Poaceae | FR2012 | 2012-90-B |
| Partitiviridae | Unclassified | Coat protein | *Elytrigia sp.* | Poaceae | FR2012 | 2012-92-F |
| Partitiviridae | Unclassified | RNA-dependent RNA polymerase | *Schedonorus arundinaceus* | Poaceae | FR2012 | 2012-44-B |
| Partitiviridae | Unclassified | Coat protein | *Hordeum marinum* | Poaceae | FR2012 | 2012-74-I |
| Partitiviridae | Unclassified | RNA-dependent RNA polymerase | *Anisantha diandra* | Poaceae | SA2010 | 1D-087 |
| Partitiviridae | Unclassified | RNA-dependent RNA polymerase | *Lolium perenne* | Poaceae | SA2010 | 1B-090 |
| Partitiviridae | Unclassified | RNA-dependent RNA polymerase | *Briza maxima* | Poaceae | SA2010 | 1A-049 |
| Partitiviridae | Unclassified | Coat protein | *Pennisetum sp.* | Poaceae | SA2010 | 1I-090 |
| Partitiviridae | Unclassified | RNA-dependent RNA polymerase | *Triticum turgidum* | Poaceae | SA2010 | 1E-094 |
| Partitiviridae | Unclassified | Coat protein | *Triticum turgidum* | Poaceae | SA2010 | 1D-047 |
| Partitiviridae | Unclassified | RNA-dependent RNA polymerase | *Cynodon dactylon* | Poaceae | SA2010 | 1A-038 |
| Partitiviridae | Unclassified | Coat protein | *Anisantha diandra* | Poaceae | SA2010 | 1G-052 |
| Partitiviridae | Unclassified | RNA-dependent RNA polymerase | *Bromus pectinatus* | Poaceae | SA2010 | 1C-052 |
| Partitiviridae | Unclassified | Coat protein | *Avena fatua* | Poaceae | SA2010 | 1F-059 |
| Partitiviridae | Unclassified | RNA-dependent RNA polymerase | *Briza maxima* | Poaceae | SA2010 | 1E-050 |
| Partitiviridae | Unclassified | RNA-dependent RNA polymerase | *Leucospermum hypophyllocarpodendron* | Proteaceae | SA2010 | 1A-004 |
| Partitiviridae | Unclassified | RNA-dependent RNA polymerase | *Ranunculus bulbosus* | Ranunculaceae | FR2012 | 2012-32-F |
| Partitiviridae | Unclassified | Coat protein | *Ranunculus parviflora* | Ranunculaceae | FR2012 | 2012-62-D |
| Partitiviridae | Unclassified | Coat protein | *Not identified* |  | SA2010 | 1D-036 |
| Partitiviridae | Unclassified | RNA-dependent RNA polymerase | *Not identified* |  | SA2010 | 1I-083 |
| Partitiviridae | Unclassified | RNA-dependent RNA polymerase | *Not identified* |  | SA2010 | 1F-051 |
| Partitiviridae | Unclassified | Coat protein | *Not identified* |  | SA2010 | 1E-089 |
| Partitiviridae | Unclassified | RNA-dependent RNA polymerase | *Not identified* |  | SA2010 | 1B-086 |
| Partitiviridae | Unclassified | RNA-dependent RNA polymerase | *Not identified* |  | SA2010 | 1C-023 |

**Chrysoviridae (57 single reads and contigs)**

| **Family** | **Genus** | **Coding region** | **Host plant** | **Plant Family** | **Location** | **Sample number** |
| --- | --- | --- | --- | --- | --- | --- |
| Chrysoviridae | Chrysovirus | RNA-dependent RNA polymerase | *Conicosia sp.* | Aizoaceae | SA2010 | 1F-082 |
| Chrysoviridae | Chrysovirus | Putative protease | *Capnophyllum africanum* | Apiaceae | SA2010 | 1E-052 |
| Chrysoviridae | Chrysovirus | RNA-dependent RNA polymerase | *Capnophyllum africanum* | Apiaceae | SA2010 | 1D-073 |
| Chrysoviridae | Chrysovirus | Hv145SV-protein 3 | *Capnophyllum africanum* | Apiaceae | SA2010 | 1F-092 |
| Chrysoviridae | Chrysovirus | Hv145SV-protein 3 | *Asparagus rubicundus* | Asparagaceae | SA2010 | 1E-080 |
| Chrysoviridae | Chrysovirus | RNA-dependent RNA polymerase | *Eriocephalus racemosa* | Asteraceae | SA2010 | 1B-091 |
| Chrysoviridae | Chrysovirus | Putative coat protein | *Berkheya sp.* | Asteraceae | SA2010 | 1H-032 |
| Chrysoviridae | Chrysovirus | Putative coat protein | *Helichrysum sp.* | Asteraceae | SA2010 | 1B-034 |
| Chrysoviridae | Chrysovirus | Hv145SV-protein 4 | *Spergularia salina* | Caryophyllaceae | FR2010 | 78-1A |
| Chrysoviridae | Chrysovirus | Hypothetical protein | *Atriplex prostrata* | Chenopodiaceae | FR2012 | 2012-15-B |
| Chrysoviridae | Chrysovirus | RNA-dependent RNA polymerase | *Halimione portulacoides* | Chenopodiaceae | FR2012 | 2012-14-A |
| Chrysoviridae | Chrysovirus | Hv145SV-protein 3 | *Halimione portulacoides* | Chenopodiaceae | FR2012 | 2012-74-C |
| Chrysoviridae | Chrysovirus | Putative protease | *Trifolium resupinatum* | Fabaceae | FR2010 | 33-1A |
| Chrysoviridae | Chrysovirus | RNA-dependent RNA polymerase | *Vicia cracca* | Fabaceae | FR2010 | 30-1G |
| Chrysoviridae | Chrysovirus | RNA-dependent RNA polymerase | *Medicago sativa* | Fabaceae | FR2012 | 2012-44-F |
| Chrysoviridae | Chrysovirus | RNA-dependent RNA polymerase | *Trifolium resupinatum* | Fabaceae | FR2012 | 2012-32-J |
| Chrysoviridae | Chrysovirus | RNA-dependent RNA polymerase | *Aspalathus sp.* | Fabaceae | SA2010 | 1G-015 |
| Chrysoviridae | Chrysovirus | RNA-dependent RNA polymerase | *Stachys aethiopica* | Lamiaceae | SA2010 | 1C-091 |
| Chrysoviridae | Chrysovirus | Hv145SV-protein 3 | *Ballota africana* | Lamiaceae | SA2010 | 1J-082 |
| Chrysoviridae | Chrysovirus | RNA-dependent RNA polymerase | *Cissampelos sp.* | Menispermaceae | SA2010 | 1K-075 |
| Chrysoviridae | Chrysovirus | Hypothetical protein | *Antizoma capensis* | Menispermaceae | SA2010 | 1D-044 |
| Chrysoviridae | Chrysovirus | Hv145SV-protein 4 | *Adenogramma sp.* | Molluginaceae | SA2010 | 1E-001 |
| Chrysoviridae | Chrysovirus | Hv145SV-protein 3 | *Cysticapnos vesicaria* | Papaveraceae | SA2010 | 1G-033 |
| Chrysoviridae | Chrysovirus | Putative coat protein | *Cysticapnos vesicaria* | Papaveraceae | SA2010 | 1F-023 |
| Chrysoviridae | Chrysovirus | RNA-dependent RNA polymerase | *Schedonorus arundinaceus* | Poaceae | FR2010 | 34-1E |
| Chrysoviridae | Chrysovirus | RNA-dependent RNA polymerase | *Schedonorus arundinaceus* | Poaceae | FR2010 | 36-1A |
| Chrysoviridae | Chrysovirus | RNA-dependent RNA polymerase | *Schedonorus arundinaceus* | Poaceae | FR2010 | 47-1E |
| Chrysoviridae | Chrysovirus | RNA-dependent RNA polymerase | *Schedonorus arundinaceus* | Poaceae | FR2012 | 2012-58-A |
| Chrysoviridae | Chrysovirus | RNA-dependent RNA polymerase | *Schedonorus arundinaceus* | Poaceae | FR2012 | 2012-36-A |
| Chrysoviridae | Chrysovirus | RNA-dependent RNA polymerase | *Schedonorus arundinaceus* | Poaceae | FR2012 | 2012-36-C |
| Chrysoviridae | Chrysovirus | RNA-dependent RNA polymerase | *Parapholis filiformis* | Poaceae | FR2012 | 2012-83-C |
| Chrysoviridae | Chrysovirus | Putative coat protein | *Hordeum marinum* | Poaceae | FR2012 | 2012-11-B |
| Chrysoviridae | Chrysovirus | RNA-dependent RNA polymerase | *Schedonorus arundinaceus* | Poaceae | FR2012 | 2012-44-B |
| Chrysoviridae | Chrysovirus | Putative coat protein | *Avena byzantina* | Poaceae | SA2010 | 1B-098 |
| Chrysoviridae | Chrysovirus | Hv145SV-protein 3 | *Pennisetum sp.* | Poaceae | SA2010 | 1I-090 |
| Chrysoviridae | Chrysovirus | Hv145SV-protein 3 | *Avena byzantina* | Poaceae | SA2010 | 1B-018 |
| Chrysoviridae | Chrysovirus | Hv145SV-protein 4 | *Avena byzantina* | Poaceae | SA2010 | 1D-096 |
| Chrysoviridae | Chrysovirus | Putative coat protein | *Avena byzantina* | Poaceae | SA2010 | 1A-097 |
| Chrysoviridae | Chrysovirus | RNA-dependent RNA polymerase | *Avena byzantina* | Poaceae | SA2010 | 1B-096 |
| Chrysoviridae | Chrysovirus | Hv145SV-protein 3 | *Stipagrostis sp.* | Poaceae | SA2010 | 1B-008 |
| Chrysoviridae | Chrysovirus | RNA-dependent RNA polymerase | *Lolium perenne* | Poaceae | SA2010 | 1D-086 |
| Chrysoviridae | Chrysovirus | Hv145SV-protein 3 | *Phalaris minor* | Poaceae | SA2010 | 1C-094 |
| Chrysoviridae | Chrysovirus | Putative coat protein | *Phalaris minor* | Poaceae | SA2010 | 1I-021 |
| Chrysoviridae | Chrysovirus | Putative protease | *Nylandtia spinosa* | Polygalaceae | SA2010 | 1G-041 |
| Chrysoviridae | Chrysovirus | Hypothetical protein | *Polypogon sp.* | Polygonaceae | FR2012 | 2012-36-D |
| Chrysoviridae | Chrysovirus | Hypothetical protein | *Rumex lativalvis* | Polygonaceae | SA2010 | 1C-063 |
| Chrysoviridae | Chrysovirus | Hv145SV-protein 3 | *Galium debile* | Rubiaceae | FR2012 | 2012-31-E |
| Chrysoviridae | Chrysovirus | Hv145SV-protein 3 | *Nemesia sp.* | Scrophulariaceae | SA2010 | 1H-002 |
| Chrysoviridae | Chrysovirus | Putative coat protein | *Not identified* |  | FR2010 | 32-1B |
| Chrysoviridae | Chrysovirus | RNA-dependent RNA polymerase | *Not identified* |  | SA2010 | 1J-044 |
| Chrysoviridae | Chrysovirus | Putative protease | *Not identified* |  | SA2010 | 1I-006 |
| Chrysoviridae | Chrysovirus | Hv145SV-protein 3 | *Not identified* |  | SA2010 | 1F-087 |
| Chrysoviridae | Chrysovirus | Putative coat protein | *Not identified* |  | SA2010 | 1A-006 |
| Chrysoviridae | Chrysovirus | Hv145SV-protein 3 | *Not identified* |  | SA2010 | 1B-086 |
| Chrysoviridae | Chrysovirus | Hv145SV-protein 4 | *Not identified* |  | SA2010 | 1B-011 |
| Chrysoviridae | Chrysovirus | Putative protease | *Not identified* |  | SA2010 | 1B-083 |
| Chrysoviridae | Chrysovirus | Putative coat protein | *Not identified* |  | SA2010 | 1G-062 |

**Totiviridae (88 single reads and contigs)**

| **Family** | **Genus** | **Coding region** | **Host plant** | **Plant Family** | **Location** | **Sample number** |
| --- | --- | --- | --- | --- | --- | --- |
| Totiviridae | Totivirus | RNA-dependent RNA polymerase | *Capnophyllum africana* | Apiaceae | SA2010 | 1D-073 |
| Totiviridae | Totivirus | RNA-dependent RNA polymerase | *Capnophyllum africana* | Apiaceae | SA2010 | 1F-071 |
| Totiviridae | Totivirus | Polyprotein | *Limbarda cithmoides* | Asteraceae | FR2012 | 2012-06-A |
| Totiviridae | Totivirus | RNA-dependent RNA polymerase | *Cirsium arvense* | Asteraceae | FR2012 | 2012-58-C |
| Totiviridae | Totivirus | RNA-dependent RNA polymerase | *Gymnodiscus sp.* | Asteraceae | SA2010 | 1H-019 |
| Totiviridae | Totivirus | RNA-dependent RNA polymerase | *Cotula turbinata* | Asteraceae | SA2010 | 1C-086 |
| Totiviridae | Totivirus | RNA-dependent RNA polymerase | *Helichrysum sp.* | Asteraceae | SA2010 | 1B-034 |
| Totiviridae | Totivirus | RNA-dependent RNA polymerase | *Lobularia sp.* | Brassicaceae | SA2010 | 1C-085 |
| Totiviridae | Totivirus | RNA-dependent RNA polymerase | *Raphanus sp.* | Brassicaceae | SA2010 | 1B-100 |
| Totiviridae | Totivirus | RNA-dependent RNA polymerase | *Cyphia sp.* | Campanulaceae | SA2010 | 1A-044 |
| Totiviridae | Totivirus | RNA-dependent RNA polymerase | *Atriplex prostrata* | Chenopodiaceae | FR2012 | 2012-15-B |
| Totiviridae | Totivirus | RNA-dependent RNA polymerase | *Halimione portulacoides* | Chenopodiaceae | FR2012 | 2012-92-B |
| Totiviridae | Totivirus | RNA-dependent RNA polymerase | *Halimione portulacoides* | Chenopodiaceae | FR2012 | 2012-04-E |
| Totiviridae | Totivirus | RNA-dependent RNA polymerase | *Halimione portulacoides* | Chenopodiaceae | FR2012 | 2012-74-C |
| Totiviridae | Totivirus | RNA-dependent RNA polymerase | *Euphorbia cyparicias* | Euphorbiaceae | FR2010 | 17-1B |
| Totiviridae | Totivirus | RNA-dependent RNA polymerase | *Lotus glaber* | Fabaceae | FR2010 | 44-1F |
| Totiviridae | Totivirus | RNA-dependent RNA polymerase | *Trifolium repens* | Fabaceae | FR2010 | 34-1D |
| Totiviridae | Totivirus | RNA-dependent RNA polymerase | *Trifolium repens* | Fabaceae | FR2010 | 44-1D |
| Totiviridae | Totivirus | RNA-dependent RNA polymerase | *Medicago sativa* | Fabaceae | FR2012 | 2012-48-C |
| Totiviridae | Totivirus | RNA-dependent RNA polymerase | *Trifolium sp.* | Fabaceae | FR2012 | 2012-34-C |
| Totiviridae | Totivirus | RNA-dependent RNA polymerase | *Trifolium resupinatum* | Fabaceae | FR2012 | 2012-32-J |
| Totiviridae | Totivirus | RNA-dependent RNA polymerase | *Lotus corniculatus* | Fabaceae | FR2012 | 2012-33-A |
| Totiviridae | Totivirus | RNA-dependent RNA polymerase | *Trifolium pratense* | Fabaceae | FR2012 | 2012-31-L |
| Totiviridae | Totivirus | RNA-dependent RNA polymerase | *Cyclopia genistoides* | Fabaceae | SA2010 | 1D-053 |
| Totiviridae | Totivirus | RNA-dependent RNA polymerase | *Aspalathus sp.* | Fabaceae | SA2010 | 1G-079 |
| Totiviridae | Totivirus | RNA-dependent RNA polymerase | *Indigofera heterophylla* | Fabaceae | SA2010 | 1C-024 |
| Totiviridae | Totivirus | RNA-dependent RNA polymerase | *Aspalathus sp.* | Fabaceae | SA2010 | 1G-015 |
| Totiviridae | Totivirus | RNA-dependent RNA polymerase | *Dipogon lignosus* | Fabaceae | SA2010 | 1H-084 |
| Totiviridae | Totivirus | RNA-dependent RNA polymerase | *Dipogon lignosus* | Fabaceae | SA2010 | 1I-031 |
| Totiviridae | Totivirus | RNA-dependent RNA polymerase | *Cyclopia genistoides* | Fabaceae | SA2010 | 1A-001 |
| Totiviridae | Totivirus | RNA-dependent RNA polymerase | *Pelargonium sp.* | Geraniaceae | SA2010 | 1A-033 |
| Totiviridae | Totivirus | RNA-dependent RNA polymerase | *Stachys aethiopica* | Lamiaceae | SA2010 | 1C-091 |
| Totiviridae | Totivirus | Capsid protein | *Pharnaceum incanum* | Molluginaceae | SA2010 | 1F-086 |
| Totiviridae | Totivirus | RNA-dependent RNA polymerase | *Cysticapnos vesicaria* | Papaveraceae | SA2010 | 1E-100 |
| Totiviridae | Totivirus | RNA-dependent RNA polymerase | *Pinus halepensis* | Pinaceae | FR2012 | 2012-17-G |
| Totiviridae | Totivirus | RNA-dependent RNA polymerase | *Elytrigia elongata* | Poaceae | FR2010 | 53-1B |
| Totiviridae | Totivirus | RNA-dependent RNA polymerase | *Polypogon nonspeliensis* | Poaceae | FR2010 | 67-1D |
| Totiviridae | Totivirus | Capsid protein | *Triticum turgidum* | Poaceae | FR2012 | 2012-07-A |
| Totiviridae | Totivirus | RNA-dependent RNA polymerase | *Avena sp.* | Poaceae | FR2012 | 2012-09-A |
| Totiviridae | Totivirus | RNA-dependent RNA polymerase | *Phragmites australis* | Poaceae | FR2012 | 2012-94-F |
| Totiviridae | Totivirus | Capsid protein | *Phragmites australis* | Poaceae | FR2012 | 2012-75-A |
| Totiviridae | Totivirus | RNA-dependent RNA polymerase | *Phragmites australis* | Poaceae | FR2012 | 2012-76-A |
| Totiviridae | Totivirus | gag-pol fusion protein [ | *Aeluropus littoralis* | Poaceae | FR2012 | 2012-82-C |
| Totiviridae | Totivirus | RNA-dependent RNA polymerase | *Aeluropus littoralis* | Poaceae | FR2012 | 2012-81-B |
| Totiviridae | Totivirus | RNA-dependent RNA polymerase | *Elytrigia sp.* | Poaceae | FR2012 | 2012-92-F |
| Totiviridae | Totivirus | RNA-dependent RNA polymerase | *Lolium perenne* | Poaceae | SA2010 | 1B-090 |
| Totiviridae | Totivirus | RNA-dependent RNA polymerase | *Pentaschistis sp.* | Poaceae | SA2010 | 1E-010 |
| Totiviridae | Totivirus | RNA-dependent RNA polymerase | *Avena fatua* | Poaceae | SA2010 | 1D-100 |
| Totiviridae | Totivirus | RNA-dependent RNA polymerase | *Avena sp.* | Poaceae | SA2010 | 1C-089 |
| Totiviridae | Totivirus | RNA-dependent RNA polymerase | *Anisantha diandra* | Poaceae | SA2010 | 1H-028 |
| Totiviridae | Totivirus | RNA-dependent RNA polymerase | *Avena byzantina* | Poaceae | SA2010 | 1C-096 |
| Totiviridae | Totivirus | RNA-dependent RNA polymerase | *Phalaris minor* | Poaceae | SA2010 | 1C-093 |
| Totiviridae | Totivirus | RNA-dependent RNA polymerase | *Avena fatua* | Poaceae | SA2010 | 1K-027 |
| Totiviridae | Totivirus | RNA-dependent RNA polymerase | *Avena byzantina* | Poaceae | SA2010 | 1B-007 |
| Totiviridae | Totivirus | RNA polymerase | *Anisantha diandra* | Poaceae | SA2010 | 1G-052 |
| Totiviridae | Totivirus | RNA-dependent RNA polymerase | *Lolium perenne* | Poaceae | SA2010 | 1D-086 |
| Totiviridae | Totivirus | RNA-dependent RNA polymerase | *Briza maxima* | Poaceae | SA2010 | 1A-085 |
| Totiviridae | Totivirus | RNA-dependent RNA polymerase | *Anagallis sp.* | Primulaceae | SA2010 | 1F-038 |
| Totiviridae | Totivirus | RNA-dependent RNA polymerase | *Willdenowia incurvata* | Restionaceae | SA2010 | 1F-024 |
| Totiviridae | Totivirus | RNA-dependent RNA polymerase | *Willdenowia incurvata* | Restionaceae | SA2010 | 1E-021 |
| Totiviridae | Totivirus | RNA-dependent RNA polymerase | *Nemesia sp.* | Scrophulariaceae | SA2010 | 1H-002 |
| Totiviridae | Totivirus | RNA-dependent RNA polymerase | *Nemesia sp.* | Scrophulariaceae | SA2010 | 1C-044 |
| Totiviridae | Totivirus | RNA-dependent RNA polymerase | *Hemimeris sp.* | Scrophulariaceae | SA2010 | 1G-013 |
| Totiviridae | Totivirus | RNA-dependent RNA polymerase | *Nemesia sp.* | Scrophulariaceae | SA2010 | 1B-060 |
| Totiviridae | Totivirus | Capsid protein | *Not identified* |  | FR2012 | 2012-10-A |
| Totiviridae | Totivirus | RNA-dependent RNA polymerase | *Not identified* |  | FR2012 | 2012-56-E |
| Totiviridae | Totivirus | RNA-dependent RNA polymerase | *Not identified* |  | SA2010 | 1D-028 |
| Totiviridae | Totivirus | RNA-dependent RNA polymerase | *Not identified* |  | SA2010 | 1H-090 |
| Totiviridae | Totivirus | RNA-dependent RNA polymerase | *Not identified* |  | SA2010 | 1I-006 |
| Totiviridae | Totivirus | RNA-dependent RNA polymerase | *Not identified* |  | SA2010 | 1C-069 |
| Totiviridae | Totivirus | RNA-dependent RNA polymerase | *Not identified* |  | SA2010 | 1E-089 |
| Totiviridae | Totivirus | RNA-dependent RNA polymerase | *Not identified* |  | SA2010 | 1B-086 |
| Totiviridae | Totivirus | RNA-dependent RNA polymerase | *Not identified* |  | SA2010 | 1B-011 |
| Totiviridae | Totivirus | RNA-dependent RNA polymerase | *Not identified* |  | SA2010 | 1B-083 |
| Totiviridae | Unclassified | RNA-dependent RNA polymerase | *Cineraria sp.* | Asteraceae | SA2010 | 1H-051 |
| Totiviridae | Unclassified | RNA-dependent RNA polymerase | *Cineraria sp.* | Asteraceae | SA2010 | 1G-072 |
| Totiviridae | Unclassified | RNA-dependent RNA polymerase | *Limonium narbonense* | Plumbaginaceae | FR2012 | 2012-87-E |
| Totiviridae | Unclassified | RNA-dependent RNA polymerase | *Schedonorus arundinaceus* | Poaceae | FR2012 | 2012-16-D |
| Totiviridae | Unclassified | RNA-dependent RNA polymerase | *Lolium perenne* | Poaceae | FR2012 | 2012-78-B |
| Totiviridae | Unclassified | RNA-dependent RNA polymerase | *Avena fatua* | Poaceae | SA2010 | 1B-057 |
| Totiviridae | Unclassified | RNA-dependent RNA polymerase | *Lolium perenne* | Poaceae | SA2010 | 1E-074 |
| Totiviridae | Unclassified | RNA-dependent RNA polymerase | *Not identified* |  | FR2010 | 32-1A |
| Totiviridae | Victorivirus | RNA-dependent RNA polymerase | *Arctopus echinatus* | Apiaceae | SA2010 | 1A-058 |
| Totiviridae | Victorivirus | RNA-dependent RNA polymerase | *Lebeckia sepiaria* | Fabaceae | SA2010 | 1F-073 |
| Totiviridae | Victorivirus | RNA-dependent RNA polymerase | *Ehrharta calycina* | Poaceae | SA2010 | 1E-058 |
| Totiviridae | Victorivirus | RNA-dependent RNA polymerase | *Ammophyla sp.* | Poaceae | SA2010 | 1A-032 |
| Totiviridae | Victorivirus | RNA-dependent RNA polymerase | *Not identified* |  | SA2010 | 1D-048 |

**ssDNA viruses (128 single reads and contigs)**

| **Taxonomy** | **Coding region** | **Host plant** | **Plant Family** | **Location** | **Sample number** |
| --- | --- | --- | --- | --- | --- |
| Unclassified ssDNA viruses | Replication-associated protein | *Conicosia sp.* | Aizoaceae | SA2010 | 1A-068 |
| Unclassified ssDNA viruses | Replication-associated protein | *Salicornia fruticosa* | Amaranthaceae | FR2010 | 02-1C |
| Unclassified ssDNA viruses | Replication-associated protein | *Halimione portulacoides* | Amaranthaceae | FR2010 | 03-1B |
| Unclassified ssDNA viruses | Capsid protein | *Salicornia fruticosa* | Amaranthaceae | FR2010 | 63-1D |
| Unclassified ssDNA viruses | Capsid protein | *Halimione portulacoides* | Amaranthaceae | FR2010 | 96-1C |
| Unclassified ssDNA viruses | Capsid protein | *Salicornia fructosa* | Amaranthaceae | FR2010 | 96-1E |
| Unclassified ssDNA viruses | Capsid protein | *Chenopodium album* | Amaranthaceae | FR2012 | 2012-54-A |
| Unclassified ssDNA viruses | Capsid protein | *Rhus laevigata* | Anacardiaceae | SA2010 | 1D-006 |
| Unclassified ssDNA viruses | Capsid protein | *Torilis nodosa* | Apiaceae | FR2012 | 2012-24-B |
| Unclassified ssDNA viruses | Replication-associated protein | *Arctopus echinatus* | Apiaceae | SA2010 | 1A-058 |
| Unclassified ssDNA viruses | Capsid protein | *Sonchus bulbosus* | Asteraceae | FR2010 | 14-1F |
| Unclassified ssDNA viruses | Capsid protein | *Helminthotheca echioides* | Asteraceae | FR2010 | 18-1J |
| Unclassified ssDNA viruses | Capsid protein | *Crepis vesicaria subsp. taraxifolia* | Asteraceae | FR2010 | 30-1E |
| Unclassified ssDNA viruses | Replication-associated protein | *Sonchus asper* | Asteraceae | FR2010 | 55-1B |
| Unclassified ssDNA viruses | Capsid protein | *Scorzonera laciniata* | Asteraceae | FR2010 | 64-1E |
| Unclassified ssDNA viruses | Capsid protein | *Bidens sp.* | Asteraceae | FR2012 | 2012-09-C |
| Unclassified ssDNA viruses | Replication-associated protein | *Chrysanthemum sp.* | Asteraceae | FR2012 | 2012-36-E |
| Unclassified ssDNA viruses | Replication-associated protein | *Lamprocephalus sp.* | Asteraceae | SA2010 | 1D-040 |
| Unclassified ssDNA viruses | Replication-associated protein | *Cotula turbinata* | Asteraceae | SA2010 | 1C-086 |
| Unclassified ssDNA viruses | Capsid protein | *Raphanus sp.* | Brassicaceae | SA2010 | 1A-096 |
| Unclassified ssDNA viruses | Capsid protein | *Not identified* | Brassicaceae | SA2010 | 1E-089 |
| Unclassified ssDNA viruses | Replication-associated protein | *Campanula rapunculoides* | Campanulaceae | FR2010 | 71-1A |
| Unclassified ssDNA viruses | Replication-associated protein | *Spergularia sp.* | Caryophyllaceae | FR2012 | 2012-27-D |
| Unclassified ssDNA viruses | Capsid protein | *Suaeda vera* | Chenopodiaceae | FR2010 | 15-1B |
| Unclassified ssDNA viruses | Capsid protein | *Halimione portulacoides* | Chenopodiaceae | FR2012 | 2012-02-C |
| Unclassified ssDNA viruses | Capsid protein | *Halimione portulacoides* | Chenopodiaceae | FR2012 | 2012-04-E |
| Unclassified ssDNA viruses | Capsid protein | *Bolboschoenus maritimus* | Cyperaceae | FR2012 | 2012-50-A |
| Unclassified ssDNA viruses | Replication-associated protein | *Bolboschoenus maritimus* | Cyperaceae | FR2012 | 2012-95-B |
| Unclassified ssDNA viruses | Capsid protein | *Elaeagnus angustifolia* | Elaeagnaceae | FR2012 | 2012-22-A |
| Unclassified ssDNA viruses | Capsid protein | *Lotus glaber* | Fabaceae | FR2010 | 34-1A |
| Unclassified ssDNA viruses | Replication-associated protein | *Trifolium resupinatum* | Fabaceae | FR2010 | 55-1D |
| Unclassified ssDNA viruses | Capsid protein | *Medicago truncatula* | Fabaceae | FR2010 | 92-1B |
| Unclassified ssDNA viruses | Replication-associated protein | *Trifolium pratense* | Fabaceae | FR2012 | 2012-31-L |
| Unclassified ssDNA viruses | Replication-associated protein | *Trifolium resupinatum* | Fabaceae | FR2012 | 2012-32-J |
| Unclassified ssDNA viruses | Capsid protein | *Trifolium sp.* | Fabaceae | FR2012 | 2012-48-D |
| Unclassified ssDNA viruses | Capsid protein | *Cyclopia genistoides* | Fabaceae | SA2010 | 1D-053 |
| Unclassified ssDNA viruses | Capsid protein | *Lotus sp.* | Fabaceae | SA2010 | 1C-099 |
| Unclassified ssDNA viruses | Capsid protein | *Aspalathus sp.* | Fabaceae | SA2010 | 1B-038 |
| Unclassified ssDNA viruses | Capsid protein | *Babiana sp.* | Iridaceae | SA2010 | 1H-079 |
| Unclassified ssDNA viruses | Capsid protein | *Juncus gerardii* | Juncaceae | FR2010 | 81-1D |
| Unclassified ssDNA viruses | Replication-associated protein | *Juncus gerardii* | Juncaceae | FR2012 | 2012-36-G |
| Unclassified ssDNA viruses | Replication-associated protein | *Pharnaceum incanum* | Molluginaceae | SA2010 | 1D-094 |
| Unclassified ssDNA viruses | Capsid protein | *Phillyrea angustifolia* | Oleaceae | FR2010 | 71-1D |
| Unclassified ssDNA viruses | Capsid protein | *Phillyrea angustifolia* | Oleaceae | FR2012 | 2012-23-F |
| Unclassified ssDNA viruses | Capsid protein | *Epilobium hirsutum* | Onagraceae | FR2010 | 73-1C |
| Unclassified ssDNA viruses | Capsid protein | *Cysticapnos vesicaria* | Papaveraceae | SA2010 | 1B-089 |
| Unclassified ssDNA viruses | Capsid protein | *Cysticapnos vesicaria* | Papaveraceae | SA2010 | 1E-100 |
| Unclassified ssDNA viruses | Capsid protein | *Plantago major* | Plantaginaceae | FR2010 | 31-1B |
| Unclassified ssDNA viruses | Capsid protein | *Limonium narbonense* | Plumbaginaceae | FR2012 | 2012-51-C |
| Unclassified ssDNA viruses | Capsid protein | *Rostraria cristata* | Poaceae | FR2010 | 03-1D |
| Unclassified ssDNA viruses | Replication-associated protein | *Bromus hordeaceus* | Poaceae | FR2010 | 03-1C |
| Unclassified ssDNA viruses | Capsid protein | *Triticum sp.* | Poaceae | FR2010 | 09-1C |
| Unclassified ssDNA viruses | Capsid protein | *Dactylis hispanica* | Poaceae | FR2010 | 13-1F |
| Unclassified ssDNA viruses | Capsid protein | *Bromus hordeaceus* | Poaceae | FR2010 | 18-1A |
| Unclassified ssDNA viruses | Capsid protein | *Puccinellia festuciformis* | Poaceae | FR2010 | 25-1C |
| Unclassified ssDNA viruses | Capsid protein | *Sorghum sp.* | Poaceae | FR2010 | 25-1A |
| Unclassified ssDNA viruses | Replication-associated protein | *Hordeum marinum* | Poaceae | FR2010 | 45-1C |
| Unclassified ssDNA viruses | Capsid protein | *Hordeum marinum* | Poaceae | FR2010 | 55-1F |
| Unclassified ssDNA viruses | Capsid protein | *Lolium sp.* | Poaceae | FR2010 | 57-1E |
| Unclassified ssDNA viruses | Capsid protein | *Bromus hordeaceus* | Poaceae | FR2010 | 61-1G |
| Unclassified ssDNA viruses | Capsid protein | *Schedonorus arundinaceus* | Poaceae | FR2010 | 75-1C |
| Unclassified ssDNA viruses | Capsid protein | *Echinochloa crus-galli* | Poaceae | FR2010 | 79-1B |
| Unclassified ssDNA viruses | Capsid protein | *Bromus hordeaceus* | Poaceae | FR2010 | 83-1C |
| Unclassified ssDNA viruses | Replication-associated protein | *Hordeum marinum* | Poaceae | FR2010 | 86-1A |
| Unclassified ssDNA viruses | Capsid protein | *Phragmites australis* | Poaceae | FR2010 | 66-1E |
| Unclassified ssDNA viruses | Capsid protein | *Oryza sativa* | Poaceae | FR2010 | 80-1E |
| Unclassified ssDNA viruses | Replication-associated protein | *Elytrigia acuta* | Poaceae | FR2012 | 2012-02-F |
| Unclassified ssDNA viruses | Replication-associated protein | *Hordeum murinum* | Poaceae | FR2012 | 2012-23-E |
| Unclassified ssDNA viruses | Capsid protein | *Hordeum murinum* | Poaceae | FR2012 | 2012-24-C |
| Unclassified ssDNA viruses | Replication-associated protein | *Schedonorus arundinaceus* | Poaceae | FR2012 | 2012-31-H |
| Unclassified ssDNA viruses | Replication-associated protein | *Dactylis glomerata* | Poaceae | FR2012 | 2012-32-G |
| Unclassified ssDNA viruses | Capsid protein | *Hordeum marinum* | Poaceae | FR2012 | 2012-33-D |
| Unclassified ssDNA viruses | Replication-associated protein | *Schedonorus arundinaceus* | Poaceae | FR2012 | 2012-36-A |
| Unclassified ssDNA viruses | Capsid protein | *Oryza sativa* | Poaceae | FR2012 | 2012-39-A |
| Unclassified ssDNA viruses | Replication-associated protein | *Hordeum murinum* | Poaceae | FR2012 | 2012-48-B |
| Unclassified ssDNA viruses | Capsid protein | *Festuca arundinacea* | Poaceae | FR2012 | 2012-58-A |
| Unclassified ssDNA viruses | Replication-associated protein | *Cynodon dactylon* | Poaceae | FR2012 | 2012-66-G |
| Unclassified ssDNA viruses | Replication-associated protein | *Dactylis hispanica* | Poaceae | FR2012 | 2012-71-D |
| Unclassified ssDNA viruses | Capsid protein | *Avena byzantina* | Poaceae | SA2010 | 1B-097 |
| Unclassified ssDNA viruses | Replication-associated protein | *Anisantha diandra* | Poaceae | SA2010 | 1D-087 |
| Unclassified ssDNA viruses | Replication-associated protein | *Lolium perenne* | Poaceae | SA2010 | 1B-090 |
| Unclassified ssDNA viruses | Capsid protein | *Lolium sp.* | Poaceae | SA2010 | 1B-069 |
| Unclassified ssDNA viruses | Replication-associated protein | *Avena fatua* | Poaceae | SA2010 | 1F-070 |
| Unclassified ssDNA viruses | Capsid protein | *Anisantha diandra* | Poaceae | SA2010 | 1F-078 |
| Unclassified ssDNA viruses | Replication-associated protein | *Anisantha diandra* | Poaceae | SA2010 | 1F-028 |
| Unclassified ssDNA viruses | Capsid protein | *Avena byzantina* | Poaceae | SA2010 | 1B-098 |
| Unclassified ssDNA viruses | Capsid protein | *Avena fatua* | Poaceae | SA2010 | 1D-100 |
| Unclassified ssDNA viruses | Replication-associated protein | *Avena byzantina* | Poaceae | SA2010 | 1E-099 |
| Unclassified ssDNA viruses | Replication-associated protein | *Avena byzantina* | Poaceae | SA2010 | 1G-097 |
| Unclassified ssDNA viruses | Capsid protein | *Avena byzantina* | Poaceae | SA2010 | 1G-099 |
| Unclassified ssDNA viruses | Capsid protein | *Avena sp.* | Poaceae | SA2010 | 1C-089 |
| Unclassified ssDNA viruses | Replication-associated protein | *Pennisetum sp.* | Poaceae | SA2010 | 1I-090 |
| Unclassified ssDNA viruses | Replication-associated protein | *Avena byzantina* | Poaceae | SA2010 | 1E-078 |
| Unclassified ssDNA viruses | Capsid protein | *Anisantha diandra* | Poaceae | SA2010 | 1H-028 |
| Unclassified ssDNA viruses | Replication-associated protein | *Avena byzantina* | Poaceae | SA2010 | 1D-098 |
| Unclassified ssDNA viruses | Capsid protein | *Avena byzantina* | Poaceae | SA2010 | 1C-096 |
| Unclassified ssDNA viruses | Capsid protein | *Avena byzantina* | Poaceae | SA2010 | 1F-099 |
| Unclassified ssDNA viruses | Replication-associated protein | *Avena byzantina* | Poaceae | SA2010 | 1E-098 |
| Unclassified ssDNA viruses | Replication-associated protein | *Avena byzantina* | Poaceae | SA2010 | 1D-096 |
| Unclassified ssDNA viruses | Capsid protein | *Avena byzantina* | Poaceae | SA2010 | 1A-097 |
| Unclassified ssDNA viruses | Replication-associated protein | *Phalaris minor* | Poaceae | SA2010 | 1C-093 |
| Unclassified ssDNA viruses | Capsid protein | *Anisantha diandra* | Poaceae | SA2010 | 1E-087 |
| Unclassified ssDNA viruses | Replication-associated protein | *Avena byzantina* | Poaceae | SA2010 | 1F-069 |
| Unclassified ssDNA viruses | Capsid protein | *Avena byzantina* | Poaceae | SA2010 | 1B-096 |
| Unclassified ssDNA viruses | Replication-associated protein | *Anisantha diandra* | Poaceae | SA2010 | 1A-007 |
| Unclassified ssDNA viruses | Capsid protein | *Hordeum murinum* | Poaceae | SA2010 | 1K-090 |
| Unclassified ssDNA viruses | Capsid protein | *Stipagrostis sp.* | Poaceae | SA2010 | 1B-008 |
| Unclassified ssDNA viruses | Capsid protein | *Anisantha diandra* | Poaceae | SA2010 | 1A-099 |
| Unclassified ssDNA viruses | Capsid protein | *Ranunculus sardous* | Ranunculaceae | FR2010 | 62-1A |
| Unclassified ssDNA viruses | Capsid protein | *Ranunculus sp.* | Ranunculaceae | FR2012 | 2012-47-G |
| Unclassified ssDNA viruses | Capsid protein | *Thamnochortus spicigerus* | Restionaceae | SA2010 | 1G-063 |
| Unclassified ssDNA viruses | Capsid protein | *Thamnochortus spicigerus* | Restionaceae | SA2010 | 1A-009 |
| Unclassified ssDNA viruses | Capsid protein | *Schizaea pectinata* | Schizaeaceae | SA2010 | 1L-082 |
| Unclassified ssDNA viruses | Capsid protein | *Lyperia tristis* | Scrophulariaceae | SA2010 | 1C-008 |
| Unclassified ssDNA viruses | Capsid protein | *Solanum sp.* | Solanaceae | SA2010 | 1D-099 |
| Unclassified ssDNA viruses | Capsid protein | *Verbena officinalis* | Verbenaceae | FR2012 | 2012-58-G |
| Unclassified ssDNA viruses | Capsid protein | *Corynotheca sp.* | Xanthorrhoeaceae | SA2010 | 1A-089 |
| Unclassified ssDNA viruses | Capsid protein | *Not identified* |  | FR2010 | 17-1E |
| Unclassified ssDNA viruses | Capsid protein | *Not identified* |  | FR2010 | 32-1A |
| Unclassified ssDNA viruses | Replication-associated protein | *Not identified* |  | SA2010 | 1D-028 |
| Unclassified ssDNA viruses | Capsid protein | *Not identified* |  | SA2010 | 1G-039 |
| Unclassified ssDNA viruses | Capsid protein | *Not identified* |  | SA2010 | 1D-036 |
| Unclassified ssDNA viruses | Capsid protein | *Not identified* |  | SA2010 | 1F-051 |
| Unclassified ssDNA viruses | Capsid protein | *Not identified* |  | SA2010 | 1B-077 |
| Unclassified ssDNA viruses | Replication-associated protein | *Not identified* |  | SA2010 | 1D-048 |
| Unclassified ssDNA viruses | Replication-associated protein | *Not identified* |  | SA2010 | 1G-096 |
| Unclassified ssDNA viruses | Capsid protein | *Not identified* |  | SA2010 | 1C-038 |
| Unclassified ssDNA viruses | Capsid protein | *Not identified* |  | SA2010 | 1B-068 |
